# Pericyclic Umpolung in a Catalytic Asymmetric Diels–Alder Reaction of Tropone with Enol Ethers

**DOI:** 10.1021/jacs.5c05709

**Published:** 2025-07-10

**Authors:** Tianyu Zheng, Zikuan Wang, Benjamin Mitschke, Nils Nöthling, Markus Leutzsch, Frank Neese, Benjamin List

**Affiliations:** Max-Planck-Institut für Kohlenforschung, Mülheim an der Ruhr 45470, Germany

## Abstract

One remarkable feature of catalysis in chemical synthesis is its capacity to override substrate-imposed reactivity and selectivity. The inversion of normal reaction patterns, commonly known as Umpolung, can be divided into (1) functional group Umpolung, where electrophilic groups are rendered nucleophilic (or vice versa), and (2) pericyclic Umpolung, in which the regioselectivity of pericyclic reactions is reversed relative to the predictions of frontier molecular orbital (FMO) theory. Although catalytic functional group Umpolung has been extensively investigated, the highly organized, concerted nature of pericyclic reactions makes inverting their conventional regioselectivity particularly challenging. To date, such inversion has been achieved only using engineered substrates or near-stoichiometric amounts of molecular cages. Here, we report an example of a chiral confined acid-catalyzed, asymmetric Umpolung of the Diels–Alder reaction. In our system, tropone reacts with enol ethers to deliver “contra-FMO” products with high yield, stereoselectivity, and regioselectivity. Mechanistic and computational studies indicate that a network of attractive noncovalent interactions, including π–π-interactions, nonclassical hydrogen bonding, and dispersion, governs the inverted regioselectivity. We anticipate that confined acid catalysis will open new avenues for addressing challenges in pericyclic Umpolung and regioselectivity control.

## Graphical abstract

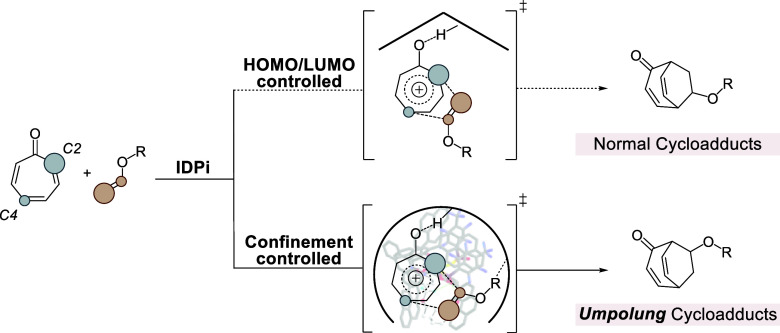

## Introduction

The Diels–Alder reaction is one of the most invaluable transformations in chemical synthesis, enabling the efficient and regioselective construction of six-membered rings. It has served as a pivotal connection strategy in the synthesis of numerous natural products and pharmaceuticals.^1^ According to FMO theory, the regioselectivity of cycloadditions such as the Diels–Alder reaction is governed by the maximum overlap between the reactants’ lowest unoccupied molecular orbital (LUMO) and highest occupied molecular orbital (HOMO).^2^ Numerous studies have confirmed this reaction pattern, even when catalysts are employed to either lower the LUMO or raise the HOMO energy of the reactants (Figure 1A).^3^

**Figure 1:**
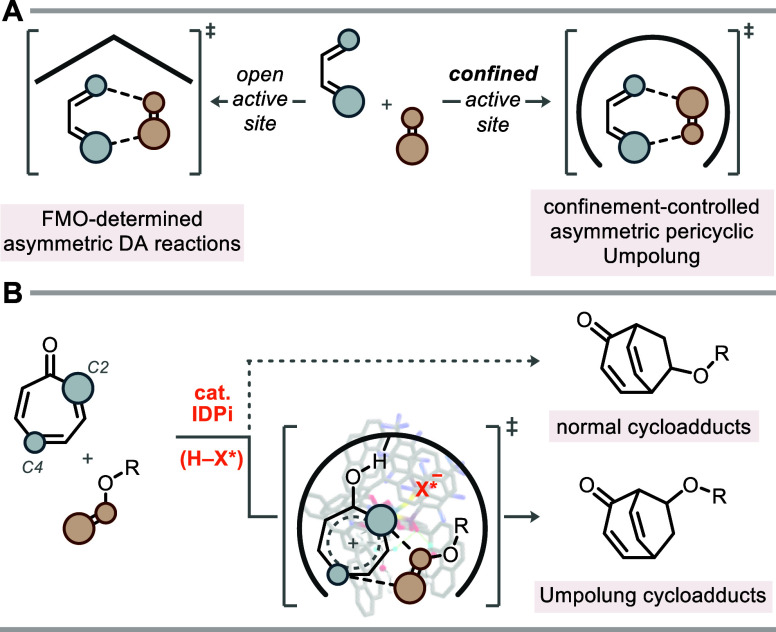
Regioselectivity of the Diels–Alder reaction. (A) The divergent regioselectivity of FMO- and confinement-controlled Diels–Alder reactions. (B) This work: Umpolung Diels–Alder reaction of tropone with enol ethers.

Reversing the inherent regiochemical preference predicted by FMO theory, a strategy referred to as pericyclic Umpolung, presents a significant synthetic challenge. Nevertheless, achieving such inversion would enrich the synthetic toolbox by simplifying synthetic routes and offering alternative retrosynthetic disconnections. To date, only a few approaches to Umpolung Diels–Alder reactions have been reported, and most require either extensive prefunctionalization or substrates with unusual electronic and steric properties.^4–11^ In one elegant approach that does not solely rely on engineered substrates, a molecular host was used to address the atypical regioselectivity in the Diels–Alder reaction.^12^ In this confined environment, the 1,4-Diels–Alder adduct of 9-hydroxymethylanthracene was obtained exclusively. However, near-stoichiometric amounts of the host promoter were needed to overcome product inhibition, which limits the broader application of this strategy. Therefore, a more general catalytic approach to the Umpolung Diels–Alder reaction remains highly desirable.

Nature masterfully employs enzymes to modify molecules with surgical precision, owing largely to their confined microenvironments. Within these specialized active sites, a network of proximal noncovalent interactions synergistically binds and preorganizes substrates, stabilizes transition states, and accelerates the desired reaction pathways. Moreover, enzymes are renowned for their ability to concurrently control enantio-, diastereo-, chemo-, and regioselectivity. Based on these observations, we propose that confinement is a critical factor in achieving unconventional regioselectivity.^13^ Inspired by nature’s blueprint, we have developed a class of imidodiphosphorimidate (IDPi) catalysts that create confined microenvironments analogous to enzyme active sites. Our work with IDPi catalysts has successfully addressed a number of synthetic challenges, including asymmetric Diels–Alder reactions.^14–18^ Consequently, we speculated that these catalysts might not only govern stereoselectivities but also facilitate access to unconventional regioisomers; that is, enable pericyclic Umpolung. Specifically, the stabilization of substrates within a confined environment through multiple noncovalent interactions may override the typical regioselectivity dictated by the orbital coefficients in HOMO–LUMO interactions (Figure 1B).

## Results and Discussion

### Reaction Development

During the optimization of asymmetric [6 + 4] cycloadditions of tropone catalyzed by heterogeneous IDPi-based noncovalent organic frameworks (NCOFs), we observed the formation of [4 + 2] cycloadducts with inverted regioselectivity.^19^ Among attractive noncovalent interactions, π–π stacking and cation−π interactions are known to stabilize transition states, and therefore, facilitate the desired reaction pathways. Given the modularity of and straightforward synthetic access to the IDPi class of catalysts, we hypothesized about the importance of polycyclic aromatic substituents in the 3,3′-positions of the 1,1’-bi-2-naphthol (BINOL) backbone in order to optimize for this unconventional outcome. Therefore, we chose the inverse electron-demand Diels–Alder reaction of tropone **1** with enol ether **2a** as the model reaction. Upon activation of the carbonyl group by the acid catalyst, the aromatic tropylium ion would be generated in situ.^20^ Moreover, tropone **1** offers various regiochemical possibilities for cycloaddition reactivity, leading to multiple selectivity challenges. Conventional [4 + 2] cycloadditions were reported using metal Lewis acid catalysts and primary amine catalysts with highly reactive 2π donors such as enamines and ketene acetals. Their regioselectivity was dictated by a matching of the large LUMO coefficient at the tropone’s C2 position and the HOMO coefficient at the terminal carbon of the electron-rich alkene, additionally aided by diastereoselectivity-determining secondary orbital interactions.^21–23^ These intrinsic properties of tropone **1** make our designed pericyclic Umpolung nontrivial and represent a unique approach for synthesizing 9-substituted bicyclo[3.2.2]­nona-3,6-dien-2-ones instead of their 8-substituted analogs. More generally, the present study highlights the utility of catalyst-substrate noncovalent interactions in obtaining pericyclic products with abnormal regioselectivity.

We initiated our investigation by reacting tropone **1** and 4-trifluoromethylbenzyl vinyl ether **2a** (10 equiv) under thermal conditions, forming the conventional regioisomers in 5% yield after 2 d (Figure 2). Metal Lewis acids like Cu­(OTf)_2_, Sc­(OTf)_3_, Fe­(OTf)_3_, and AlCl_3_ were ineffective for this transformation at −20 °C. Using BF_3_·Et_2_O, we observed decomposition of the vinyl ether **2a** and no formation of any cycloadducts **3**. In contrast, the organic Lewis acid TMSNTf_2_ was efficient in promoting this transformation smoothly. We obtained cycloadducts **3** in 77% yield and close-to-conventional (thermal) regioselectivity (r.r. = 21:79). Although the strong Brønsted acid HNTf_2_ provided product **3** with similar selectivity, the yield decreased significantly due to the decomposition of enol ether **2a**. Remarkably, by using a confined Brønsted acid, **IDPi-1**, the reaction proceeded efficiently to give cycloadduct **3** in 86% yield after 2 d, forming significant quantities of the Umpolung products **3a-1** and **3a-2** (r.r. = 59:41). Meanwhile, the diastereoselectivity of the unconventional regioisomer was less affected (d.r. = 74:26), accompanied by promising enantioselectivity (e.r. = 71:29). Further optimization by increasing the π-system of the substituents at the 3,3′ positions of the BINOL skeleton revealed **IDPi-4** bearing 1-pyrenyl substituents as the best catalyst, giving **3a-1** exclusively (r.r. = 95:5) with excellent diastereoselectivity (d.r. = 97:3) and enantioselectivity (e.r. = 99:1). This indicates a truly catalyst-controlled Umpolung Diels–Alder reaction and is consistent with our assumption that enzyme-like confined IDPi catalysts are capable of reversing the intrinsic regioselectivity.

**Figure 2:**
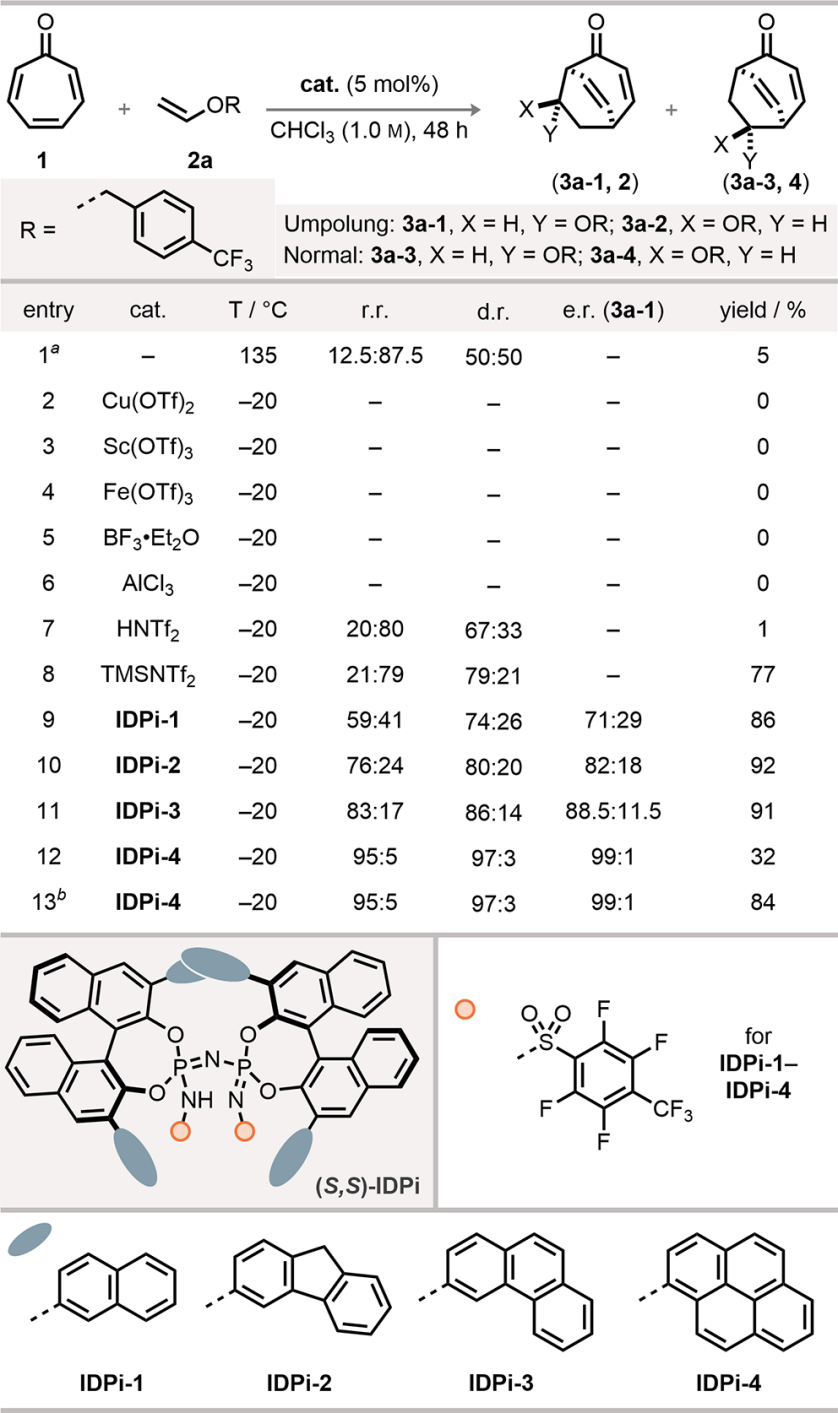
Catalyst evaluation. Conditions: the reactions were carried out with tropone **1** (0.01 mmol), **IDPi** (5 mol %), and **2a** (0.1 mmol) in chloroform (0.010 mL) at −20 °C for 48 h. The e.r. was determined by HPLC. e.r., enantiomeric ratio. a. Reaction was performed with tropone **1** (0.01 mmol), **2a** (0.1 mmol) in *m*-xylene (0.010 mL) at 135 °C for 48 h. b. Reaction was performed using tropone **1** (0.10 mmol), IDPi (2.5 mol %), and **2a** (1.0 mmol) in chloroform (0.1 mL) at – 20 °C for 7 d.

### Substrate Scope

Encouraged by these results, we investigated the scope of this Umpolung Diels–Alder reaction. While we found the Brønsted acid conditions shown above to be suited for **2a**, extensive polymerization was observed for α-substituted enol ethers. Enol ethers are less prone to be protonated under Lewis acidic conditions, which can inhibit the polymerization. Therefore, we reoptimized the reaction using silylium ion Lewis acids (see Figure S1–S2). Gratifyingly, regardless of the electronic properties of α-aryl substituents, enol ethers **2** proceeded smoothly under silylium Lewis acid conditions using **IDPi-5** as the catalyst (with the Lewis acid generated *in situ* from **IDPi-5** and BSTFA, *N*,*O*-bis­(trimethylsilyl)­trifluoroacetamide). The Umpolung products **3** were obtained exclusively in moderate to high yields (38% to 99%, Figure 3A), albeit with moderate diastereoselectivities (see Figure S6). The optimal catalyst’s **IDPi-5** inner core was decorated with a comparatively smaller CF_3_ group in place of the 2,3,5,6-tetrafluoro-4-trifluoromethylphenyl group in **IDPi-4**, reflecting the increased steric demand of TMS^+^ versus H^+^, as well as that of the α-substituent of the enol ether. Besides, excellent enantioselectivities were also obtained at the same time (e.r. = 95:5 to 98:2). Substrates **2e**–**2h** with different steric demands also underwent this transformation efficiently, giving exclusively the pericyclic Umpolung products **3e**–**3h**. An α-alkyl substituted enol ether **2p** was also tested, in which case **IDPi-6** was identified to be the optimal catalyst. The unconventional regioisomers **3p** were obtained exclusively with high enantioselectivity (e.r. = 95:5, Figure 3B). In summary, the investigation of a broad scope of enol ethers further corroborated our hypothesis of a completely catalyst-controlled Umpolung Diels–Alder reaction.

**Figure 3:**
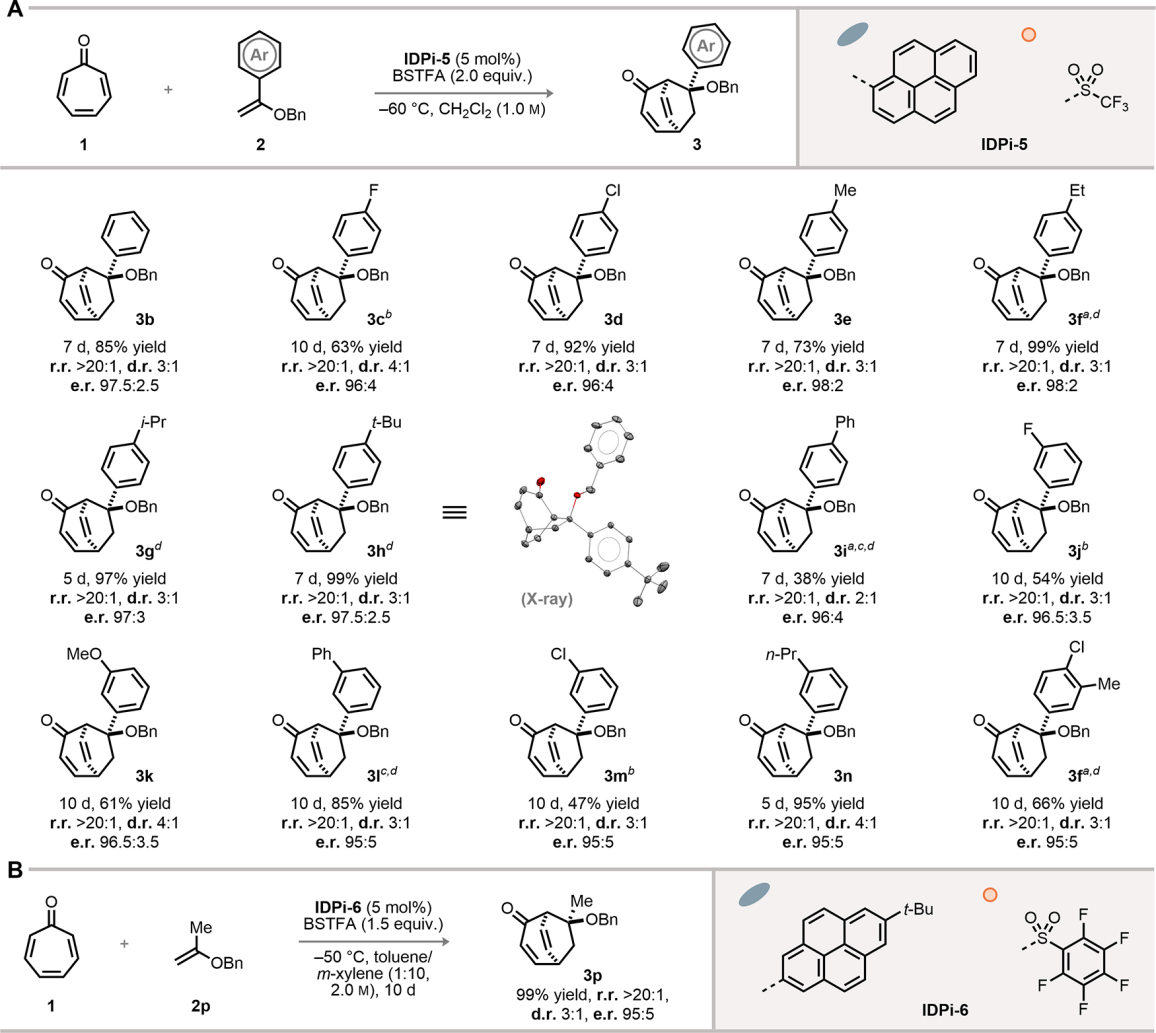
Substrate scope. (A) Substrate scope of α-aryl enol ethers. Reaction conditions: tropone **1** (0.1 mmol), **IDPi-5** (5 mol %), BSTFA (0.2 mmol) and **2** (1.0 mmol) in dichloromethane (0.1 mL) at −60 °C for 7 d. a. in chloroform, b. at −55 °C, c. at – 50 °C, d. in dichloromethane (0.2 mL). (B) Reaction of α-methyl enol ether. Reaction conditions: tropone **1** (0.1 mmol), **IDPi**-**6** (5 mol %), and **2p** (2.0 mmol) in toluene/*m*-xylene (1:10, *v*/*v*, 0.1 mL) at −50 °C for 10 d. The r.r. and d.r. values were determined by ^1^H NMR and e.r. was determined by HPLC. r.r., regiomeric ratio, d.r. diastereomeric ratio, e.r., enantiomeric ratio.

### Mechanistic Considerations

Consistent with our initial hypothesis of stabilizing π–π interactions between the catalyst and the substrate, enlarging the π-system of the substituents in the IDPi catalysts led to an increase of the yields of unconventional cycloadducts. This phenomenon suggests that the π-interactions in the confined environment might play a crucial role in controlling regioselectivity. Therefore, an NMR study was carried out to gain mechanistic insight about this Umpolung Diels–Alder reaction (Figure 4A). After mixing **IDPi-4** and tropone **1** (1:1) in CD_2_Cl_2_, the ^31^P NMR signal was deshielded, implying the spontaneous formation of an ion pair between the IDPi anion^40^ and hydroxytropylium ion (**1-H** ^**+**^). At the same time, the ^1^H NMR signals of tropone were shifted toward lower ppm values. In sharp contrast, in the 1:1 mixture of tropone **1** and the highly acidic but nonconfined acid HNTf_2_, an obvious downfield shift of all protons was observed. This result indicates a strong π-interaction between the 1-pyrenyl substituents of **IDPi-4** and **1-H** ^**+**^, which puts the tropone protons in the shielding zone of the 1-pyrenyl group.

**Figure 4:**
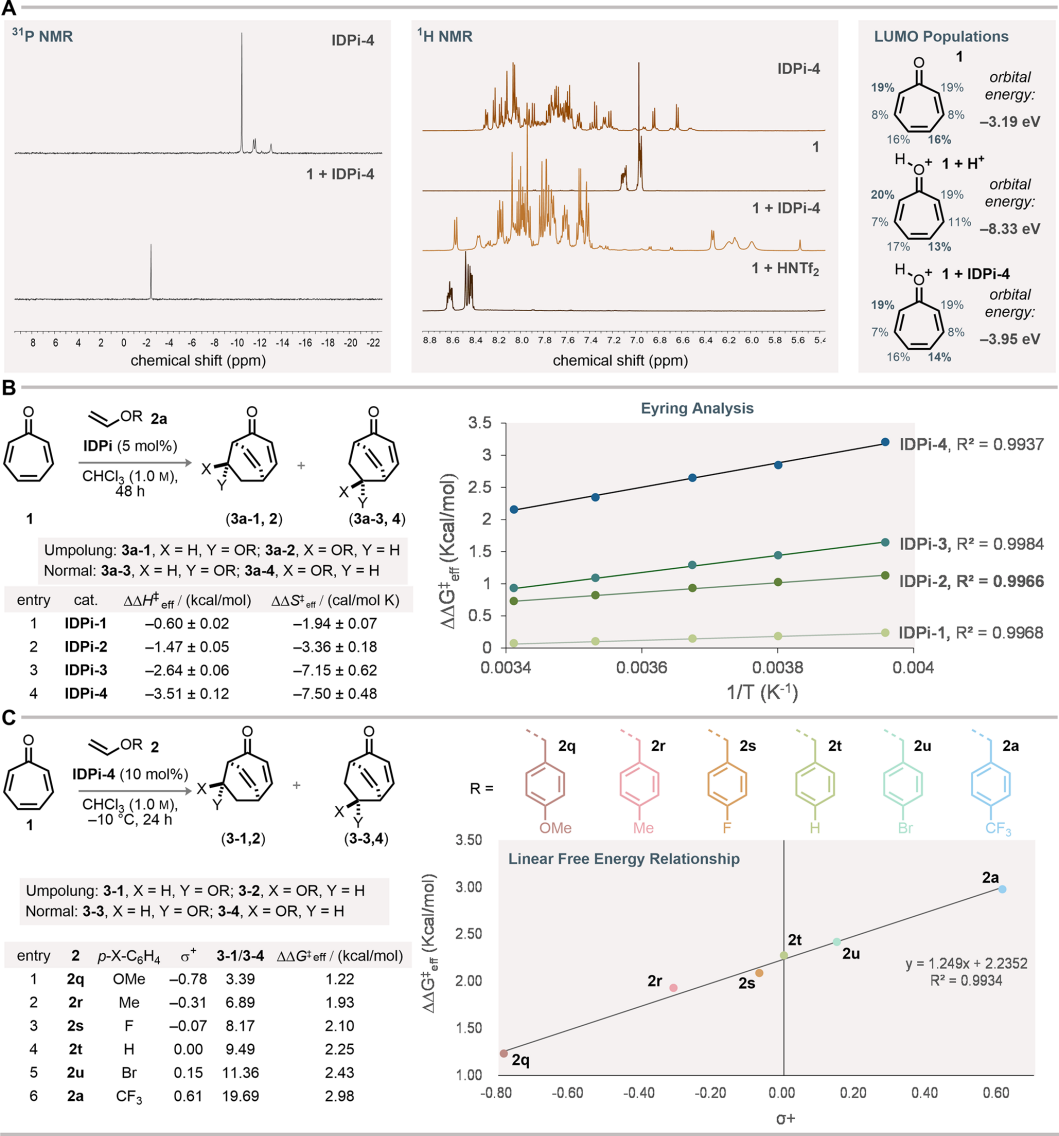
Mechanistic studies. (A) ^1^H NMR and ^31^P NMR study of tropone **1** with **IDPi-4** and HNTf_2_. LUMO energies and Hirshfeld populations (calculated from PBE/def2-SVP wave functions generated from ORCA,^26^ with the help of Multiwfn 3.8­(dev)^27^) of **1** in different chemical environments are given on the right. (B) Eyring analysis of the regioselectivity in the model reaction (tropone **1** with enol ether **2a**) using **IDPi-1–4**. (C) Linear free energy relationship study with different enol ethers **2**.

As our previous study^19^ showed the spontaneous formation of a noncovalent organic framework (NCOF) between the IDPi and tropone **1** via self-assembly, diffusion-ordered NMR spectroscopy (DOSY) was carried out to estimate the molecular weight (MW) of the complex (tropone **1**:**IDPi-4**, 1:1). The measured MW supported the formation of a simple 1:1 heterodimer instead of a NCOF (see Supporting Information). Calculated LUMO populations of this ion pair are similar to that of protonated tropone and free tropone (Figure 4A). This indicates the origin of the pericyclic Umpolung resides at the confined environment provided by the **IDPi-4** anion, instead of a change of FMO distribution of tropone. Next, Eyring analysis of the regioselectivity was performed using catalysts **IDPi-1**, **-2**, **-3**, **-4** in the model Umpolung Diels–Alder reaction (Figure 4B). Reaction pathways of the two main regioisomers **3a-1** and **3a-4** were compared using effective relative Gibbs free energy (ΔΔ*G* ^‡^_eff_). All four catalysts showed perfect linear correlation between the ΔΔ*G* ^‡^_eff_ (RTln (**3a-1**/**3a-4**)) and the inverse temperature over a range of 40 °C. As the π-system of the substituents in the IDPi catalyst series is consecutively enlarged, the effective enthalpy difference ΔΔ*H* ^‡^_eff_ in the transition states between the confinement-controlled Umpolung pathway and the FMO-controlled pathway increased dramatically. In fact, ΔΔ*H* ^‡^_eff_ in the reaction catalyzed by **IDPi-4** is 6 times higher than in the reaction of **IDPi-1**. The more favorable effective enthalpy ΔΔ*H* ^‡^_eff_ with increased π-systems suggests more favorable binding interactions in the transition state of the Umpolung pathway compared to the conventional pathway.^24^ By comparison, the binding in the transition state becomes entropically more unfavorable upon enlarging the π-system of the substituents (4 times between **IDPi-4** and **IDPi-1**), indicating a more rigid transition structure for the pericyclic Umpolung pathway. Furthermore, an apparent linear relationship between polarizabilities of different arenes in IDPi catalysts and ΔΔ*G* ^‡^_eff_ was also observed (*R* ^2^ = 0.98, see Supporting Information). This further supports that the π- or dispersion interactions in the transition state are the main factors for the reversal of regioselectivity instead of steric effects.^24^ Next, a linear free energy relationship (LFER) study was conducted by varying para-substituents (MeO–, Me–, F–, H–, Br–, CF_3_−) in the enol ethers **2** with **IDPi-4** as the catalyst (Figure 4C).^25^ The Hammett-type constant σ+ and ΔΔ*G* ^‡^_eff_ exhibit strong correlation (*R* ^2^ = 0.99), which suggests that the electronegativity of the benzylic group is crucial to regioselectivity.

### Theoretical Studies

Density functional theory (DFT) calculations (ωB97M-V^28^/def2-QZVP^29^/SMD­(CHCl_3_)^30,31^ //PBE^32^-D3 ^33,34^ /def2-SVP^29^ of the reaction between tropone **1**, **IDPi-4** and enol ether **2a** were performed using the ORCA software^26^ suite to further elucidate the mechanism of this Umpolung Diels–Alder reaction. While the usual computational workflow involves using a semiempirical method such as GFN2-xTB^35^ for conformational search, we have found that GFN2-xTB gives qualitatively wrong conformer ensembles due to inaccurate treatments of the phosphorus and sulfur atoms in **IDPi-4**. Although we refined the GFN2-xTB conformer geometries by DFT geometry optimizations, many important transition state conformers were missed at the GFN2-xTB level from the beginning, so that a low enantioselectivity (e.r. = 27:73) with a preference of the opposite enantiomer relative to experiment was observed. Therefore, we reparameterized 13 parameters of the GFN2-xTB method related to phosphorus and sulfur (in particular their atomic orbital energy levels and orbital exponents; see Table S9), against the PBE-D3/def2-SVP energies and gradients evaluated at about 900 **IDPi-4** ^–^ + **1-H** ^**+**^ + **2a** transition state conformers generated by GFN2-xTB molecular dynamics trajectories. Similar reparameterization techniques have been successfully applied by others^36,37^ and us.^38^

Unlike the unmodified GFN2-xTB method which gives a dense manifold of low-lying conformers of **IDPi-4** ^–^, the reparameterized GFN2-xTB method (combined with ORCA’s conformation search module, GOAT^39^ predicts a well-defined lowest energy conformer (DFT-refined structure shown in Figure 5A) of **IDPi-4** ^–^ that is 2.5 kcal/mol (0.4 kcal/mol at the DFT level) lower than the second most stable conformer. **IDPi-4** ^–^ exposes two negatively charged pockets, one surrounded by fluorine substituents (the “fluorous pocket”) and the other essentially without fluorine atoms nearby (the “non-fluorous pocket”). Docking **1**-H^+^ into **IDPi-4** ^–^ yields two major conformers after DFT refinement (**RC-1** and **RC-2**; Figure 5B). In both conformers, **1-H** ^**+**^ forms a hydrogen bond with the nonfluorous pocket of **IDPi-4** ^–^, but with different orientations. Independent gradient model based on Hirshfeld partition (IGMH) plots^40^ reveal that while in the lowest conformer **RC-1**, **1-H** ^**+**^ forms a π–π-stacking interaction with one of the pyrene rings of **IDPi-4** ^–^ and a C–H···π interaction with another pyrene ring, in **RC-2** (whose Gibbs free energy is, within error, the same as **RC-1**) no π–π-stacking interactions but only C–H···π interactions were found. In contrast to **RC-2** where **1-H** ^**+**^ still has considerable freedom to move, in **RC-1** the orientation of **1-H** ^**+**^ is completely fixed by three geometry constraints: (1) it has to remain coplanar with the 1-pyrene group below it; (2) it forms an O–H···O=S hydrogen bond with **IDPi-4** ^–^; and (3) the O–H···O = S hydrogen bond has to be close to linear. With the configuration of **1**-H^+^ fixed, it then becomes evident that the Umpolung pathway (**3a-1**, **3a-2**) can benefit significantly more from the attractive van der Waals interaction of the 4-trifluoromethylbenzyl group of the enol ether **2a** with **IDPi-4** ^–^, than the conventional pathway (**3a-3**, **3a-4**) can, since the other substituents of **IDPi-4** ^–^ are all located on the carbonyl side of **1-H** ^**+**^ and therefore tend to stabilize a transition state with the 4-trifluoromethylbenzyl group located close to the tropone carbonyl group. The computed conformation of **RC-1** is also consistent with the ^1^H NMR results, which indicates that the hydroxytropylium is in the shielding zones of the pyrene substituents.

**Figure 5:**
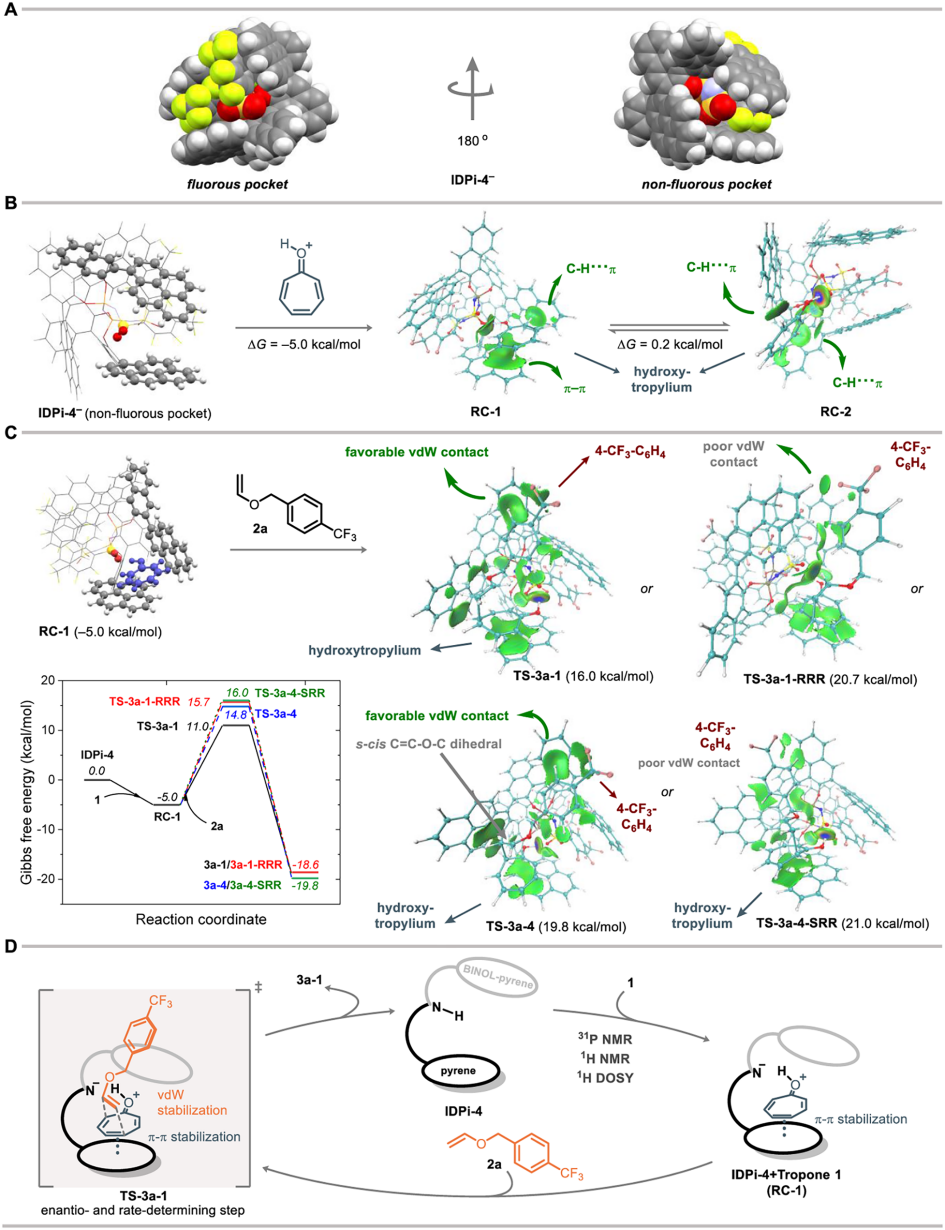
Theoretical studies. (A) The two substrate pockets of IDPi-4^–^. (B) Complexation of the hydroxytropylium cation with IDPi-4^–^ to form the reactant complexes **RC-1** and **RC-2** (IGMH plots shown, isovalue: 0.005 au). (C) Gibbs free energy profile and the structures of the lowest free energy conformers of the transition states leading to **3a-1** (**TS-3a-1**), **3a-4** (**TS-3a-4**), as well as those that lead to their enantiomers (**TS-3a-1-RRR** and **TS-3a-4-SRR**). The IGMH plots of the transition states are shown (isovalue: 0.005 au). (D) Proposed catalytic cycle.

Reaction of **RC-1**/**RC-2** with enol ether **2a** proceeds through asynchronous concerted transition states, as verified by intrinsic reaction coordinate (IRC) calculations (Figures S22–S24). The asynchronous character of the transition states is exemplified by **TS-3a-1**, where the lengths of the two forming C–C bonds are 1.959 and 3.270 Å, respectively (Figure 5C). The lowest free energy transition state conformers **TS-3a-1**, **TS-3a-4** as well as their enantiomers **TS-3a-1-RRR** and **TS-3a-4-SRR** (Figure 5C) indeed show that only **TS-3a-1** can maximize the interaction of the 4-trifluoromethylbenzyl group with the **IDPi-4** ^–^ anion, as it is situated to the right of the cavity and therefore forms strong dispersion interactions with the naphthalene and pyrene groups in the upper right part of the cavity. The 4-trifluoromethylbenzyl group in **TS-3a-1-RRR** does not have a close contact with the **IDPi-4** ^–^ anion; while in **TS-3a-4** the close contact exists, the 4-trifluoromethylbenzyl group is situated to the left of the cavity and therefore has a looser contact with the cavity. In **TS-3a-4-SRR**, the 4- trifluoromethylbenzyl group is located to the right of the cavity (resembling **TS-3a-1**), but with a high-energy *s-cis*-CH_2_=CH–O–CH_2_ dihedral angle of the enol ether substrate, and the interaction of the 4-trifluoromethylbenzyl group with IDPi is weaker than in **TS-3a-1** as reflected by the closest contacts and dispersion energies (Figure S20 and Table S12). This is consistent with the experimental finding that the conjugation lengths of the 3,3′-substituents of IDPi have a positive correlation with the enantio-, diastereo- and regioselectivity of the reaction (Figure 2, entries 9–12; Figure 4B). Our calculations therefore reproduced the experimental selectivity of the Umpolung product **3a-1**, and gave e.r. and r.r. values (>99:1 and >99:1, respectively) that agree qualitatively with experiment (99:1 and 95:5; Figure 2, entry 13). The selectivity improvement of electron-withdrawing enol ethers (Figure 5C) can be explained by two C–H···O nonclassical hydrogen bonds between the 4-trifluoromethylbenzyl group and one of the S=O groups in **TS-3a-1**, as well as the π-π interaction of the 4-trifluoromethylbenzyl group and the electron rich BINOL motif of the IDPi catalyst (Figure S3); while the other three transition states may have some of these three interactions, none of them have all three of them. To sum up, the conformational rigidity of IDPi-4, the tropone···pyrene π–π interaction, and favorable enol ether···IDPi noncovalent interactions when the 4-trifluoromethylbenzyl group is on the carbonyl side of tropone, all contribute to the high and unconventional regioselectivity of **IDPi-4**.

## Conclusions

A catalytic asymmetric Umpolung Diels–Alder reaction of tropone with enol ethers has been realized. Our detailed mechanistic investigation revealed confinement as the crucial element for enabling the overall pericyclic Umpolung with excellent enantioselectivity. Complementing previous approaches utilizing repulsive steric interactions or covalent tethering, our results highlight the preeminence of attractive noncovalent interactions within the confined active site of the catalyst to preorganize substrates, stabilize the transition state, and accelerate product formation with unconventional regioselectivity. Meanwhile, our IDPi catalysts can open new avenues to tackling fundamental issues not only in asymmetric transformations but also in various regioselective reactions to deliver significantly simplified synthetic routes.

## Supplementary Material



## Notes

Experimental details of catalyst and substrate synthesis, catalytic pericyclic Umpolung reactions, and mechanistic experiments; substrate scope using other enol ethers; experimental characterization data; computational details, including details about the reparameterization of GFN2-xTB, the conformational sampling workflow, energy decomposition analysis and CD spectrum calculation­(PDF)

## †

Laboratorium für Organische Chemie, ETH Zürich, 8093 Zürich, Switzerland

## Abbreviations

IDPi,: imidodiphosphorimidate
TMS: trimethylsilyl
Et,: ethyl
Me,: methyl
*i*Pr,: iso-propyl
*n*Pr,: normal propyl
*t*Bu,: *tert*-butyl
Ar,: aryl
Tf,: triflyl
h,: hours
d,: days
