# Supplementary material for: Pericyclic Umpolung in a Catalytic Asymmetric Diels–Alder Reaction of Tropone with Enol Ethers
Source: J Am Chem Soc. 2025 Jul 10;147(29):25489–97. doi: 10.1021/jacs.5c05709 (PMC12291457; doi:10.1021/jacs.5c05709)
Supplement: Supplementary file 1 [file ja5c05709_si_001.pdf]

Supporting Information

Pericyclic Umpolung in a Catalytic Asymmetric Diels-Alder Reaction of Tropone with Enol Ethers

Tianyu Zheng, Zikuan Wang, Benjamin Mitschke, Nils Nöthling, Markus Leutzsch, Frank Neese and Benjamin List*

Correspondence to: list@kofo.mpg.de

Content

1. General Considerations........**2**

2. Screening results of asymmetric catalytic pericyclic umpolung using enol ether 2b as substrates **4**

3. Screening results of asymmetric catalytic pericyclic umpolung using enol ether 2p as substrates **5**

4. Eyring Analysis of ratio of 3a-1/3a-4 for IDPi 1-4........**5**

5. Correlation of polarizability with ratio of 3a-1/3a-4 for IDPi 1-4........**7**

6. Linear free energy relationship of σ^+ of R with ratio of 3a-1/3a-4........**8**

7. Substrate scope of α-aryl enol ethers (minor diastereomers)........**9**

8. DOSY Analysis........**10**

9. Preparation and characterization of imidodiphosphorimidates (IDPis)........**11**

10. General procedure for the preparation of enol ethers 2a, 2q-2u........**16**

11. General procedure for the preparation of α-substituted enol ethers 2b-2o........**18**

12. Procedure for the asymmetric synthesis of 3a-1........**23**

13. General procedure for the synthesis of 3b-3o........**23**

14. X-ray Crystal Structure Analysis........**38**

15. NMR spectra........**71**

16. HPLC traces........**142**

17. Computational details........**173**

18. References........**316**

1. General considerations

Chemicals

Unless otherwise indicated, starting materials were obtained from Sigma-Aldrich, ABCR-GmbH, TCI, or Acros Co. Ltd. Moreover, commercially available reagents were used without additional purification.

Solvents

Solvents (dichloromethane, chloroform and toluene) were dried by distillation from an appropriate drying agent in the technical department of the Max-Planck-Institut für Kohlenforschung and received in Schlenk flasks under argon.

Inert Gas

Dry argon was purchased from Air Liquide with >99.5% purity.

Thin Layer Chromatography

Thin-layer chromatography (TLC) was performed using silica gel pre-coated plastic sheets (Polygram SIL G/UV254, 0.2 mm, with fluorescent indicator; Macherey-Nagel) which was visualized with a UV lamp (254 nm) and/or phosphomolybdic acid (PMA). PMA stain: PMA (20 g) in EtOH (200 mL).

Column Chromatography

Column chromatography (CC) was carried out using Merck silica gel (60 Å, 230−400 mesh, particle size 0.040−0.063 mm) using technical grade solvents. Elution was accelerated using compressed argon. All reported yields, unless otherwise specified, refer to spectroscopically and chromatographically pure compounds.

Nomenclature

Nomenclature follows the suggestions proposed by the computer program ChemBioDraw (12.0.3.1216) of CBD/cambridgesoft.

Nuclear Magnetic Resonance Spectroscopy

^{1}H, ^{13}C, ^{19}F, ^{31}P Nuclear magnetic resonance (NMR) spectra for compound characterization were recorded on Bruker AVIII-500 MHz, NMR spectrometer in a suitable deuterated solvent. The solvent employed and the respective measuring frequency are indicated for each experiment. The
resonance multiplicity is described as s (singlet), d (doublet), t (triplet), q (quadruplet), m (multiplet), and br (broad). All spectra were recorded at 298 K, processed with MestReNova 14.2.3 suits of program, and coupling constants are reported as observed. The residual deuterated solvent signal relative to tetramethylsilane was used as the internal reference in ^{1}H and ^{13}C NMR spectra (e.g. CD_2Cl_2 = 5.32 ppm in ^{1}H NMR and 53.84 ppm in ^{13}C NMR). Signals are reported as follows: chemical shift δ in ppm (multiplicity, coupling constant *J* in Hz, number of protons). All X-nuclei spectra were acquired proton decoupled unless otherwise noted.

Mass Spectrometry

Electrospray ionization (ESI) mass spectrometry was conducted on a Bruker ESQ 3000 spectrometer. High resolution mass spectra were determined on a Bruker APEX III FTMS (7 T magnet). The ionization method and mode of detection employed is indicated for the
respective experiment and all masses are reported in atomic units per elementary charge (m/z) with an intensity normalized to the most intense peak.

Specific Rotations

Specific rotations ($[\alpha]_D^{25}$) were measured with a Rudolph RA Autopol IV Automatic Polarimeter at the indicated temperature with a sodium lamp (sodium D line, λ = 589 nm). Measurements were performed in an acid resistant 1 mL cell (50 mm length) with concentrations (g/100 mL) reported in the corresponding solvent.

High Performance Liquid Chromatography

High performance liquid chromatography (HPLC) was performed on a Shimadzu LC-20AD liquid chromatograph SIL-20AC autosampler, CMB-20A using Daicel columns with a chiral stationary phase. All solvents used were HPLC-grade solvents purchased from Sigma-Aldrich. The column employed and the respective solvent mixture are indicated for each experiment.

CD (Circular Dichroism) Spectra

CD spectra were recorded at 20 °C in Hexane or MeCN (HPLC grade) using a JASCO J-1110 spectropolarimeter and precision cells (quartz suprasil, 2mm, Hellma).

Abbreviations

e.r. = enantiomeric ratio, d.r. = diastereomeric ratio, r.r. = regiomeric ratio, TLC = thin layer chromatography, TMS = $SiMe_3$, TBS = $SiMe_2tBu$, Tf = SO_2CF_3, MOM =methoxymethyl ether, BSTFA = N,O-Bis(trimethylsilyl)trifluoroacetamide, h = hours, d = days

2. Screening results of asymmetric catalytic pericyclic umpolung using enol ether 2b as substrates

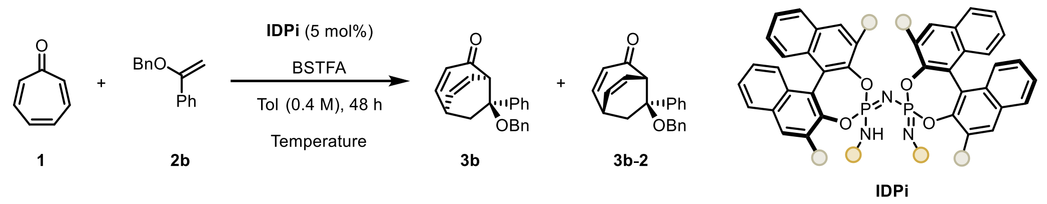

	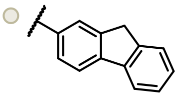	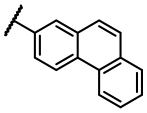	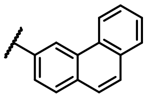	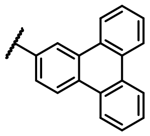	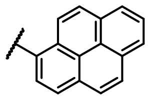
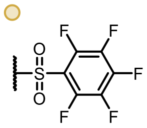	r.r. > 20: 1<br>d.r. 1: 1<br>e.r. 32: 68 (**3b**)<br>34: 66 (**3b-2**)	r.r. > 20: 1<br>d.r. 2.2: 1<br>e.r. 59.5: 40.5 (**3b**)<br>38.5: 61.5 (**3b-2**)	r.r. > 20: 1<br>d.r. 3: 1<br>e.r. 75: 25(**3b**)<br>75: 25 (**3b-2**)	r.r. > 20: 1<br>d.r. 3: 1<br>e.r. 77.5: 22.5 (**3b**)<br>59: 41 (**3b-2**)	
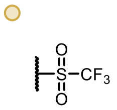		r.r. > 20: 1<br>d.r. 2.7: 1<br>e.r. 30: 70 (**3b**)<br>45: 55 (**3b-2**)	r.r. > 20: 1<br>d.r. 3: 1<br>e.r. 49: 51(**3b**)<br>60: 40 (**3b-2**)		-30°C, r.r. > 20: 1<br>d.r. 2: 1<br>e.r. 10.5: 89.5 (**3b**)<br>8.5: 91.5 (**3b-2**)
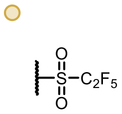				r.r. > 20: 1<br>d.r. 21: 1<br>e.r. 50: 50 (**3b**)<br>67: 33 (**3b-2**)	r.r. > 20: 1<br>d.r. 2.5: 1<br>e.r. 44.5: 55.5 (**3b**)<br>37.5: 62.5 (**3b-2**)

Figure S1: Conditions: Tropone 1 (0.01 mmol), **IDPi** (5 mol%), **BSTFA** (0.02 mmol) and **2b** (0.1 mmol) in Toluene (0.025 mL) at -20 or -30 °C for 2 days. The r.r. and d.r. were determined by ^{1}H NMR and e.r. was determined by HPLC. r.r., regiomeric ratio, d.r. diatereomeric ratio, e.r., enantiomeric ratio.

3. Screening results of asymmetric catalytic pericyclic umpolung using enol ether 2p as substrates

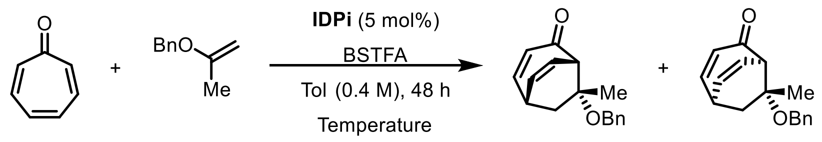

1 **2p** **3p** **3p-2**

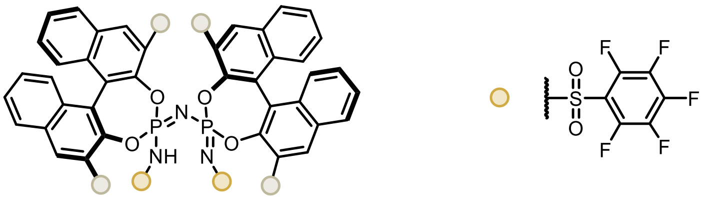

IDPi

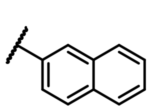	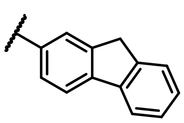	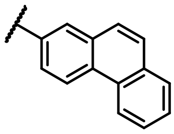	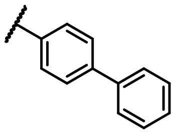
-20 °C, r.r. 7.5: 1<br>d.r. 4:1<br>e.r. 53.5: 46.5 (major)<br>62.5: 37.5 (minor)	-20 °C, r.r. 7: 1<br>d.r. 2: 1<br>e.r. 63: 37 (major)<br>87.5: 12.5 (minor)	-20 °C, r.r. 13: 1<br>d.r. 2: 1<br>e.r. 72: 28 (major)<br>92: 8 (minor)	-20 °C, r.r. 5: 1<br>d.r. 4: 1<br>e.r. 64: 36 (major)<br>69.5: 30.5 (minor)
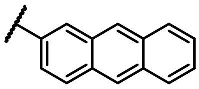	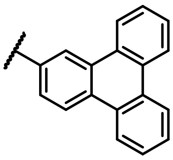	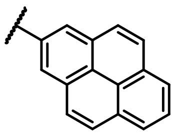	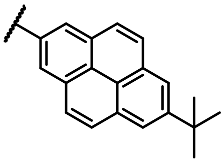
-20 °C, r.r. 5: 1<br>d.r. 2: 1<br>e.r. 68: 32 (major)<br>84.5: 15.5 (minor)	-40 °C, r.r. > 20: 1<br>d.r. 3: 1<br>e.r. 75: 25 (major)<br>82.5: 17.5 (minor)	-40 °C, r.r. > 20: 1<br>d.r. 3: 1<br>e.r. 90.5: 9.5 (major)<br>90: 10 (minor)	-40 °C, r.r. > 20: 1<br>d.r. 3: 1<br>e.r. 92.5: 7.5 (major)<br>90: 10 (minor)

Figure S2: Conditions: Tropone 1 (0.01 mmol), **IDPi** (5 mol%), **BSTFA** (0.02 mmol) and **2p** (0.1 mmol) in Toluene (0.025 mL) at indicated temperature for 2 days. The r.r. and d.r. were determined by ^{1}H NMR and e.r. was determined by HPLC. r.r., regiomeric ratio, d.r. diatereomeric ratio, e.r., enantiomeric ratio.

4. Eyring Analysis of ratio of 3a-1/3a-4 for IDPi 1-4

Procedure: An oven-dried vial was charged with tropone **1** (0.01 mmol, 1.0 equiv), IDPi (0.005 mmol, 5 mol%) and $CHCl_3$ (0.1 mL). The vial was cooled to the indicated temperature and enol ether **2a** (0.1 mmol 10.0 equiv) was added in one portion. The reaction was stirred for 24-48 h and then quenched at that temperature by addition of 0.1 mL pre-cooled triethylamine. The ratio of product **3a-1** and **3a-4** was determined by 1H NMR analysis The

differential activation parameters were calculated using the following relationship: ln (**3a-1**/**3a-4**) = –$\Delta\Delta H^{\ddagger}_{eff}$/RT + $\Delta\Delta S^{\ddagger}_{eff}$/R (where R = 1.986 cal/mol•K)

IDPi-4

1/T	(3a-1/3a-4)
0.003958045	25.54
0.003800114	19.02
0.003674444	14.83
0.003531697	11.54
0.003411223	9.85
$\Delta\Delta H^{\ddagger}_{eff}$ = -3.51 ± 0.12	
$\Delta\Delta S^{\ddagger}_{eff}$ = -7.50 ± 0.48	

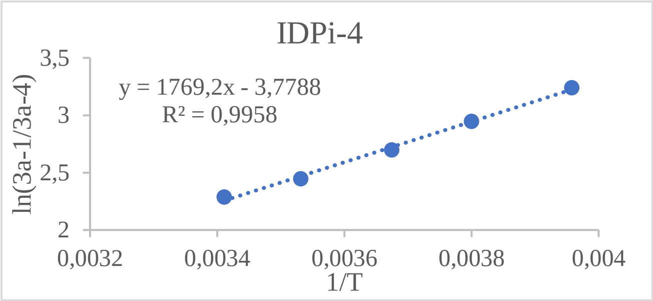

IDPi-3

1/T	(3a-1/3a-4)
0.003958045	5.21
0.003800114	4.17
0.003674444	3.64
0.003531697	2.95
0.003411223	2.51
$\Delta\Delta H^{\ddagger}_{eff}$ = -2.64± 0.06	
$\Delta\Delta S^{\ddagger}_{eff}$ = -7.15 ± 0.24	

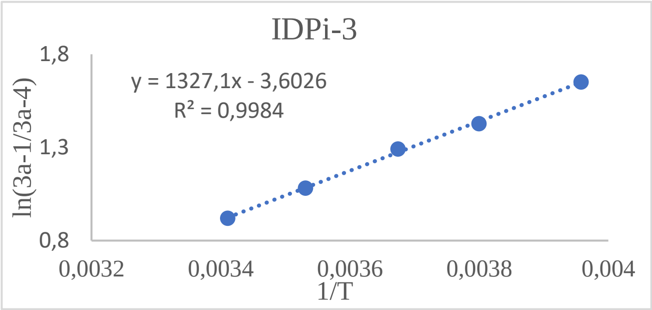

IDPi-2

1/T	(3a-1/3a-4)
0.003958045	3.07
0.003800114	2.79
0.003674444	2.54
0.003531697	2.26
0.003411223	2.06
$\Delta\Delta H^{\ddagger}_{eff}$ = -1.47± 0.05	
$\Delta\Delta S^{\ddagger}_{eff}$ = -3.36 ± 0.18	

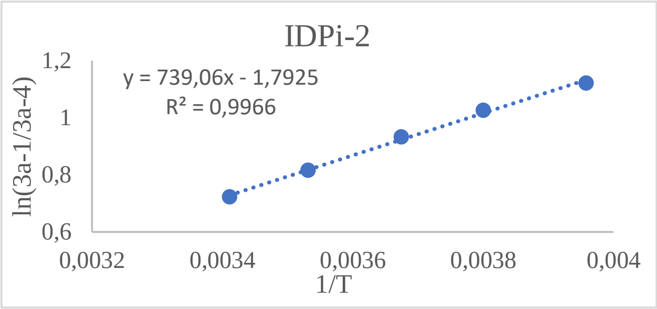

IDPi-1

1/T	(3a-1/3a-4)
0.003958045	1.26
0.003800114	1.20
0.003674444	1.15
0.003531697	1.10
0.003411223	1.07
$\Delta\Delta H^{\ddagger}_{eff}$ = -0.60 ± 0.02	
$\Delta\Delta S^{\ddagger}_{eff}$ = -1.94 ± 0.07	

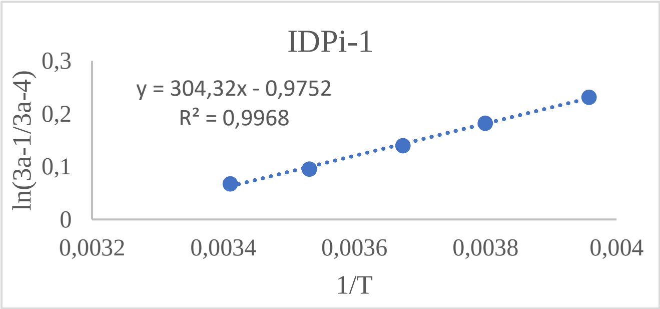

Figure S3: Eyring Analysis

5. Correlation of polarizability with ratio of 3a-1/3a-4 for IDPi 1-4

Procedure: An oven-dried vial was charged with tropone (0.01 mmol, 1.0 equiv), **IDPi** (0.005 mmol, 5 mol%) and $CHCl_3$ (0.1 mL). The vial was cooled to -20 °C and enol ether **2a** (0.1 mmol 10.0 equiv) was added in one portion. The reaction was stirred for 24-48 h and then quenched at that temperature by addition of 0.1 mL pre-cooled Triethylamine. The ratio of product **3a-1** and **3a-4** was determined by ^{1}H NMR analysis.

Arene	polarizability	3a-1/3a-4
**Napthyl**	**117**	**1.26**
**Fluorenyl**	**146**	**3.07**
**Phenanthrenyl**	**167**	**5.21**
**Pyrenyl**	**201**	**25.54**

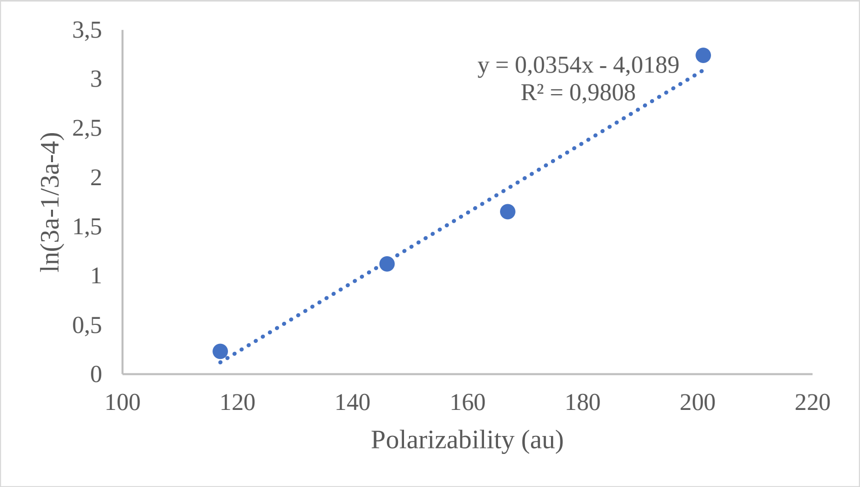

Figure S4: Correlation of polarizability with ratio of 3a-1/3a-4 for IDPi 1-4

6. Linear free energy relationship of σ^+ of R with ratio of 3a-1/3a-4

Procedure: An oven-dried vial was charged with tropone (0.01 mmol, 1.0 equiv), **IDPi** (0.005 mmol, 5 mol%) and $CHCl_3$ (0.1 mL). The vial was cooled to -10 °C and 2 (0.1 mmol 10.0 equiv) was added in one portion. The reaction was stirred for 24-48 h and then quenched at that temperature by addition of 0.1 mL pre-cooled Triethylamine. The ratio of product **3a-1** and **3a-4** was determined by ^{1}H NMR analysis.

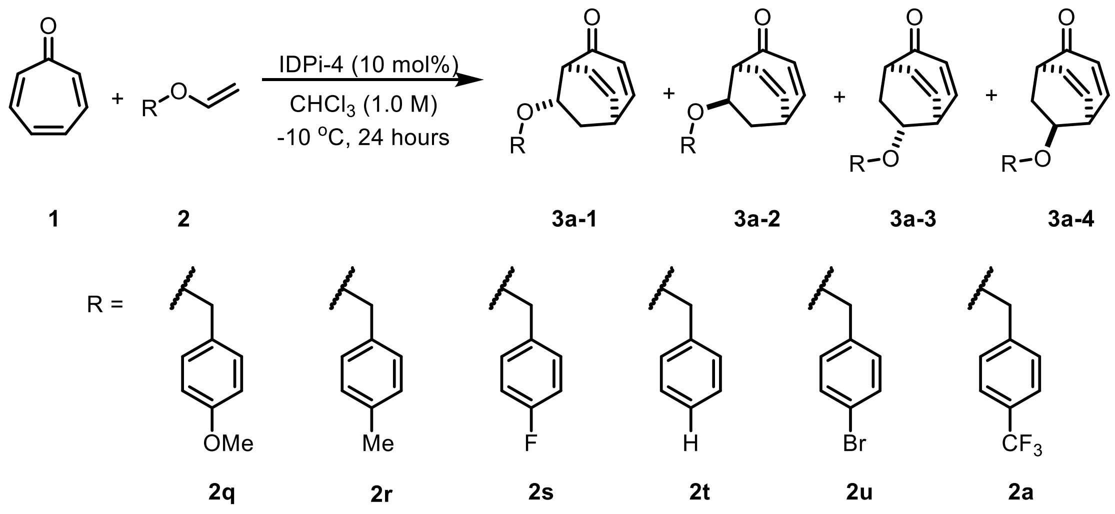

Entry	2	Substitutes	σ+	3a-1/3a-4	$\Delta\Delta G^{\ddagger}_{eff}$ (kcal/mol)
1	**2q**	OMe	-0.78	3.39	1.22
2	**2r**	Me	-0.31	6.89	1.93
3	**2s**	F	-0.07	8.17	2.10
4	**2t**	H	0.00	9.49	2.25
5	**2u**	Br	0.15	11.36	2.43
6	**2a**	CF_3	0.61	19.69	2.98

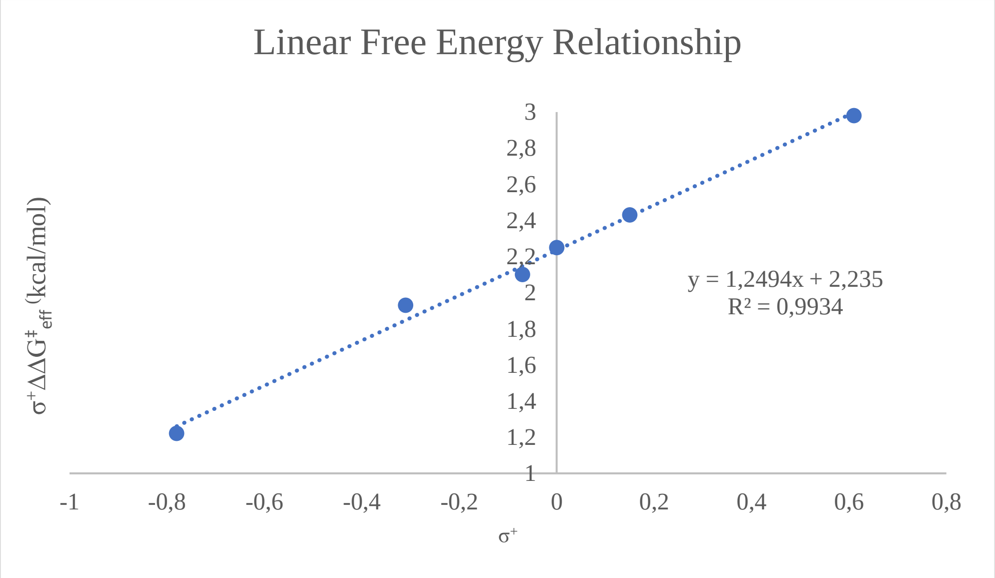

Figure S5: Linear free energy relationship of σ + of R with ratio of 3a-1/3a-4

7. Substrate scope of α-aryl enol ethers (minor diastereomers) and Substrate Limtations

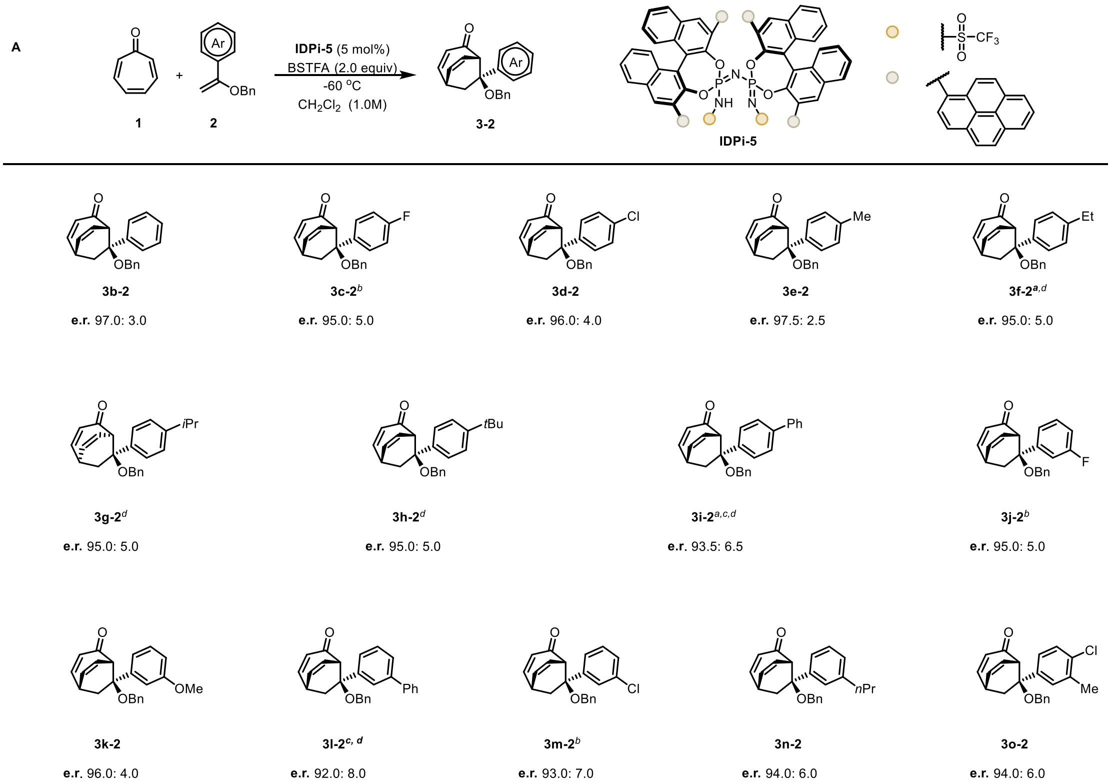

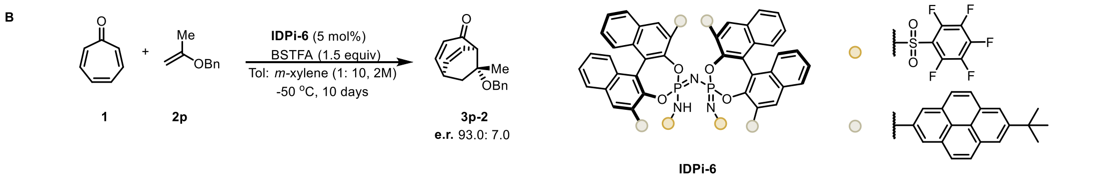

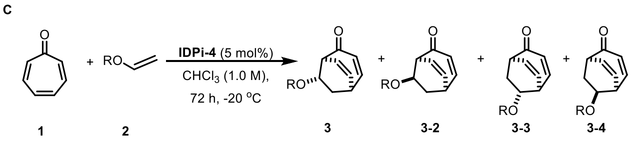

Entry	2	R	r.r. [*Umpolung*: Normal]	d.r. (**3a-1**: **3a-2**)	e.r. (**3a-1**)	Yield (%)
1	**2v**	Et	2: 1	9: 1	98: 2	45
2	**2w**	*n*Bu	4: 1	8: 1	98: 2	69
3	**2x**	*i*Bu	10: 1	11: 1	98: 2	93
4	**2y**	Cy	1: 2	1: 1	-	87

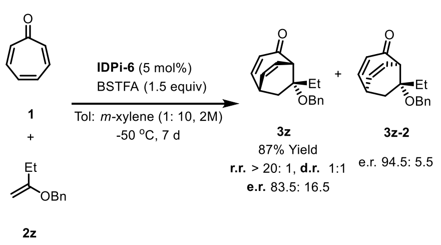

Figure S6: A. Reaction Conditions: Tropone **1** (0.1 mmol), **IDPi-5** (5 mol%), BSTFA (0.2 mmol) and **2** (1.0 mmol) in dichloromethane (0.1 mL) at -60 °C for 7 days. (a). in $CHCl_3$, (b). at -55 °C, (c). at -50 °C, (d). 0.5M **B**. Reaction of α-methyl enol ether. **B**. Reaction conditions: Tropone **1** (0.1 mmol), **IDPi-6** (5 mol%), and **2p** (2.0 mmol) in Tol/*m*-xylene (1: 10, v/v, 0.1 mL) at -50 °C for 10 days. The r.r. and d.r. were determined by ^{1}H NMR and e.r. was determined by HPLC. r.r., regiomeric ratio, d.r. diatereomeric ratio, e.r., enantiomeric ratio. **C**. Substrate Limitations

8. DOSY Analysis

Data acquisition & processing

Diffusion coefficients were obtained from a double stimulated echo sequence with bipolar gradient pulsed, convection compensation, longitudinal eddy current delay, and three spoiler gradients (Bruker sequence: dstebpgp3s). The gradient pulse strength *G* was incremented from 5% to 98% of the maximum gradient strength (G_{max}) with a linear gradient ramp in 40 steps (NMR probe: PA BBO 500S1 BBF-H-D Z PLUS; Gmax = 53.5 G·cm^{-1}). The diffusion time (delay d20) used was 85 ms and the length of a gradient pulse (δ/2) of the encoding gradient was 1.0 ms. After the measurement, the dataset was processed with the AU program *setdiffparm* in Bruker Topspin 3.6.3 and then imported into in MNOVA 15.0.0 and processed therein. The imported 2D DOSY dataset was Fourier-transformed with 1 Hz line broadening and afterwards phased and baseline corrected. 2D DOSY plots were obtained using the DOSY transform implemented in MNOVA 15.0.0. with the following options:

**Method**	**Peak Fit**
**Peak Fit Option**	
**Decay components**	**Single**
**Fit Mode**	**Normal**
**Autocorrect Peak Positions**	**No**
**Confidence interval**	**95%**
**Scaling**	
**Method**	**No scaling**
**DOSY Spectrum**	
**Units**	**m^2/sec**
**Points in diff. dim.**	**160**
**Scale**	**manual**
**min**	**3.00E-11**
**max.**	**3.00E-09**

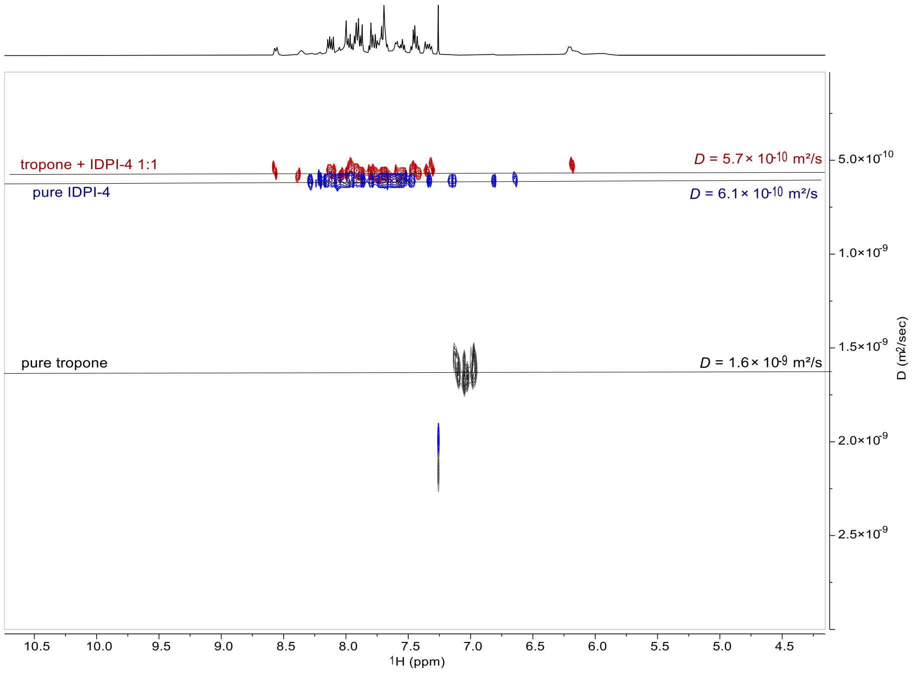

Figure S7: Stacked 2D DOSY spectra of pure tropone (black), pure IDPI-4 (blue) and the 1:1 tropone:IDPI-4 mixture in $CDCl_3$ at 298K.

The diffusion data suggests that only a monomeric 1:1 adduct is present, as the self-diffusion coefficient *D* of the IDPI-4 only reduces by only 7% upon tropon addition. A similar reduction has previously been observed by our group in the heterodimerization of chiral phosphoric acids and carboxylic acids.[1] This decrease in *D* is attributed to a slight increase of molecular weight due to the complex formation, as well as the adoption of a different conformation, which is already evident from the changes observed in the ^{1}H and ^{31}P NMR datasets.

9. Preparation and characterization of imidodiphosphorimidates (IDPis)

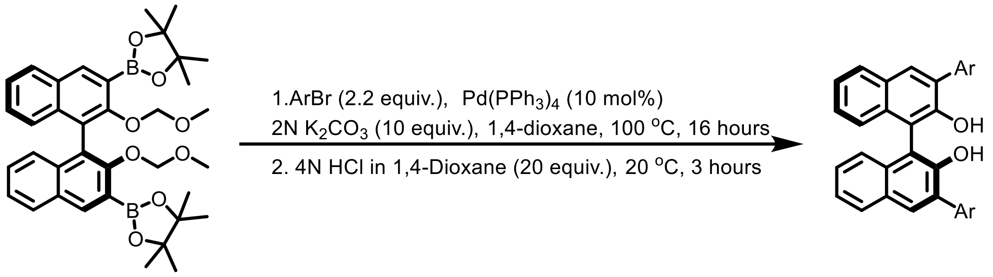

Representative procedure for 3,3'-disubstituted BINOL synthesis by Suzuki coupling and the subsequent hydrolysis:

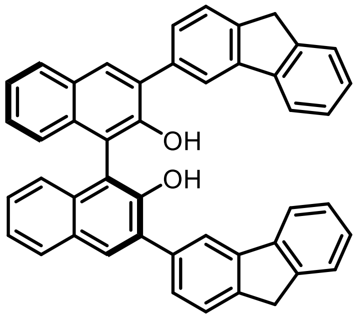

In a flame dried 2-neck round-bottom flask with a condenser, (S)-2,2'-(2,2'-bis(methoxymethoxy)-1,1'-binaphthyl-3,3'-diyl)bis(4,4,5,5-tetramethyl-1,3,2-dioxaborolane) (500 mg, 0.8 mmol, 1.0 equiv.), 3-bromo-9H-fluorene (432 mg, 2.2 equiv.), and tetrakis(triphenylphosphine)palladium (92mg, 0.08 mmol, 0.1 equiv.) were dissolved in 1,4-dioxane (10 mL). After adding aqeous K_2CO_3 solution (2.0 M, 4 mL), the resultant mixture was degassed with Argon for 10 minnutes. Then the resultant mixture was stirred at 100 °C for 12 h. After cooling to room temperature, the solvent was removed under reduced pressure. The unsoluble solids were filtered off a short pad of celite and washed with EtOAc. The solvent was removed under reduced pressure and the residue was dissolved in a small amount of DCM (1 mL). A solution of HCl (4 M in 1,4-dioxane, 3 mL) was added at R.T. and the mixture was stirred at R.T. for 3 h. The solvent was removed under reduced pressure and the residue was purified by column chromatography to afford the (S)-3,3'-di(9H-fluoren-3-yl)-[1,1'-binaphthalene]-2,2'-diol. (overall yield of the two steps: 85 %).

(S)-3,3'-di(9H-fluoren-3-yl)-[1,1'-binaphthalene]-2,2'-diol.

Yield: 86%

Appearance: light yellow solid.

^{1}H NMR (500 MHz, CD_2Cl_2) δ 8.15 (d, *J* = 17.5 Hz, 4H), 7.99 (d, *J* = 8.0 Hz, 2H), 7.87 (d, *J* = 7.4 Hz, 2H), 7.67-7.70 (m, 4H), 7.60 (d, *J* = 7.4 Hz, 2H), 7.33-7.43 (m, 8H), 7.27 (d, *J* = 8.4 Hz, 2H), 5.55 (s, 2H), 4.00 (s, 4H);

^{13}C NMR (125 MHz, CD_2Cl_2) δ 150.7, 144.1, 143.4, 142.5, 141.8, 136.6, 133.6, 131.7, 131.5, 130.0, 128.9, 128.7, 127.6, 127.4, 127.2, 125.5, 125.5, 134.7, 121.4, 120.4, 113.2, 37.2;

HRMS (ESI): m/z calcd for $C_{46}H_{29}O_2$ $[M-H]^-$]: 613.217305; Found: 613.217440.

$[\alpha]_D^{25}$ = 9.20 (c = 0.17, CH_2Cl_2)

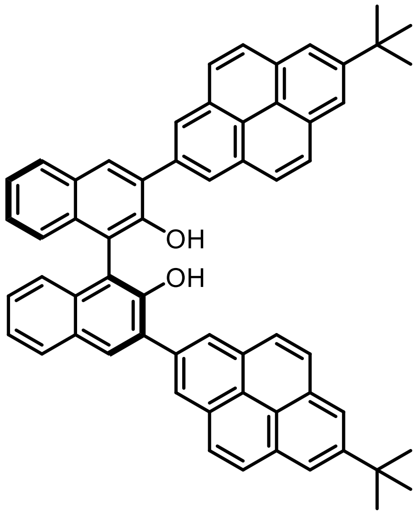

(S)-3,3'-bis(7-(tert-butyl)pyren-2-yl)-[1,1'-binaphthalene]-2,2'-diol (diol-2)

Yield: 92%

Appearance: light yellow solid.

^{1}H NMR (500 MHz, CD_2Cl_2) δ 8.56 (s, 4H), 8.30 (s, 2H), 8.29 (s, 4H), 8.4 (dd, *J* = 11.4, 8.9 Hz, 8H), 8.05 (d, *J* = 7.3 Hz, 2H), 7.47 (t, *J* = 7.4 Hz, 2H), 7.42 (t, *J* = 7.5 Hz, 2H), 7.37 (d, *J* = 7.9 Hz, 2H), 5.73 (s, 2H), 1.60 (s, 18H);

^{13}C NMR (125 MHz, $CDCl_3$) δ 150.5, 149.4, 134.8, 133.3, 132.4, 131.3, 131.2, 131.2, 129.8, 128.7, 128.2, 127.6, 127.5, 126.0, 124.2, 122.9, 122.6, 113.1, 35.4, 32.1;

HRMS (ESI): m/z calcd for $C_{60}H_{45}O_2$ [$(M-H)^-$]: 797.342505; Found: 797.343320.

$[\alpha]_D^{25}$ = 229.07 (c = 0.97, CH_2Cl_2)

General procedure for the preparation of IDPi 1-5 and IDPi-6

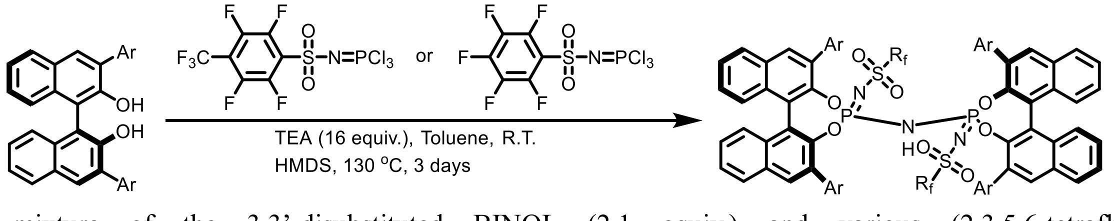

To a mixture of the 3,3'-disubstituted BINOL (2.1 equiv.) and various (2,3,5,6-tetrafluoro-4-(trifluoromethyl)phenyl)sulfonyl)phosphorimidoyl trichloride (2.1 equiv.) in toluene (0.2 M) was added triethylamine (16 equiv.) at room temperature under an argon atmosphere. After stirring for 3 hours, HMDS (1.0 equiv.) was added. After an additional 10 min at room temperature, the reaction mixture was sealed and heated to 130 °C for 3 d. The solvent was removed under reduced pressure and the crude residue was purified by column chromatography. After remove the solvent under reduced pressure, the collected solid was stirred vigorously in a biphasic solution (DCM/6 N HCl) for 15 min and extracted with DCM. Azeotropic removal of water using toluene gave IDPi 1-5 and IDPi 6 as white or light-yellow solids in their acidic form.

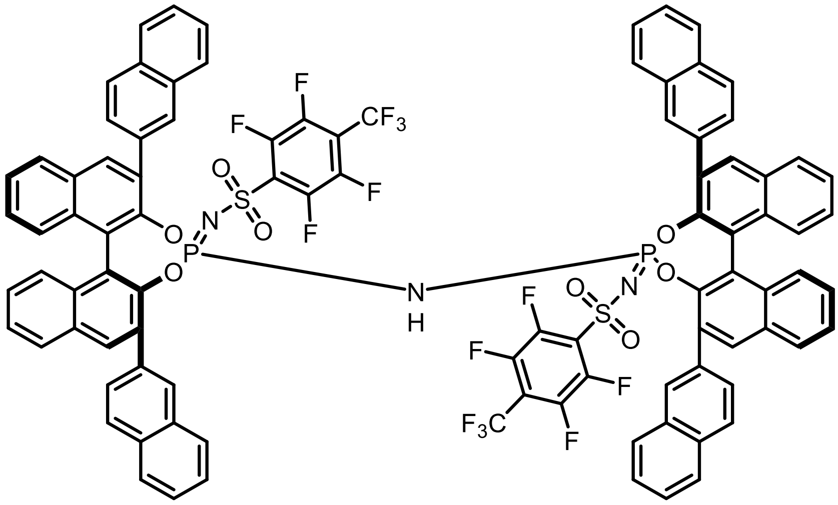

IDPi-1

Yield: 73%

Appearance: white solid;

^{1}H NMR (500 MHz, CD_2Cl_2) δ 8.07-8.14 (m, 6H), 7.85-7.90 (m, 4H), 7.74 (ddd, *J* = 8.4, 6.8, 1.3 Hz, 2H), 7.58-7.66 (m, 6H), 7.50-7.52 (m, 2H), 7.40-7.47 (m, 8H), 7.22-7.35 (m, 16H), 6.81 (dd, *J* = 8.5, 1.9 Hz, 3H), 6.24 (dd, *J* = 8.6, 2.0 Hz, 2H);

^{13}C NMR (125 MHz, CD_2Cl_2) δ 144.5, 143.6, 142.6, 133.9, 133.7, 133.7, 133.4, 133.3, 133.3, 133.1, 132.7, 132.4, 132.4, 132.3, 132.2, 132.1, 131.8, 129.8, 129.2, 129.1, 128.7, 128.6, 128.5, 128.0, 127.8, 127.6, 127.5, 127.4, 127.4, 127.3, 127.2, 127.1, 127.1, 126.9, 126.6, 126.3, 125.9, 123.8, 122.4, 119.6;

^{19}**F NMR** (471 MHz, CD_2Cl_2): δ -56.65 (t, *J* = 21.7 Hz, 3F), -135.1 (q, *J* = 13.8 Hz, 2F), -138.07 (tt, *J* = 21.2, 10.6 Hz, 2F);

^{31}**P NMR** (203 MHz, CD_2Cl_2): δ -15.71

HRMS (ESI): m/z calcd for $C_{94}H_{48}N_3O_8F_{14}S_2P_2$ [$(M-H)^-$]: 1738.214014; Found: 1738.216170.

$[\alpha]_D^{25}$ = 155.41 (c = 0.45, CH_2Cl_2)

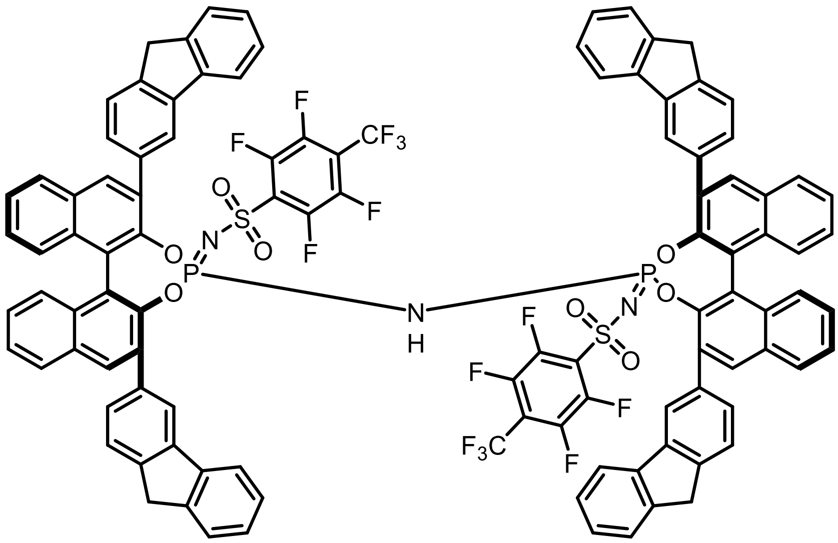

IDPi-2

Yield: 65%

Appearance: white solid;

^{1}H NMR (500 MHz, CD_2Cl_2) δ 8.05-8.12 (m, 6H), 7.94 (s, 2H), 7.82 (ddd, *J* = 8.2, 5.8, 2.1 Hz, 2H), 7.61-7.74 (m, 12H), 7.54 (s, 2H), 7.33-7.43 (m, 6H), 7.16-7.33 (m, 10H), 6.65 (d, *J* = 7.7 Hz, 2H), 6.44-6.51 (m, 4H), 6.17 (br, 1H), 5.99 (d, *J* = 7.7 Hz, 2H), 3.50-3.66 (m, 8H);

^{13}C NMR (125 MHz, CD_2Cl_2) δ 144.8, 144.1, 140.0, 143.8, 143.3, 143.2, 142.1, 141.9, 141.7, 134.7, 134.4, 134.0, 133.8, 132.6, 132.3, 132.2, 131.9, 131.7, 131.5, 129.5, 128.8, 128.7, 128.0, 127.7, 127.4, 127.3, 127.2, 127.1, 127.0, 127.0, 126.9, 125.3, 125.2 ,124.5, 123.8, 122.7, 121.9, 120.8, 120.1, 119.9, 119.3, 37.0, 36.8;

^{19}F NMR (471 MHz, CD_2Cl_2): δ -56.70 (s, 3F), -134.62 (dd, *J* = 22.8, 13.3 Hz, 2F), -138.64 (td, *J* = 22.3, 13.3 Hz, 2F);

^{31}P NMR (203 MHz, CD_2Cl_2): δ -13.04

HRMS (ESI): m/z calcd for $C_{106}H_{56}N_3O_8F_{14}S_2P_2$ [(M-H)$^-$]: 1890.276614; Found: 1890.278010.

$[\alpha]_D^{25}$ = 218.02 (c = 0.11, CH_2Cl_2)

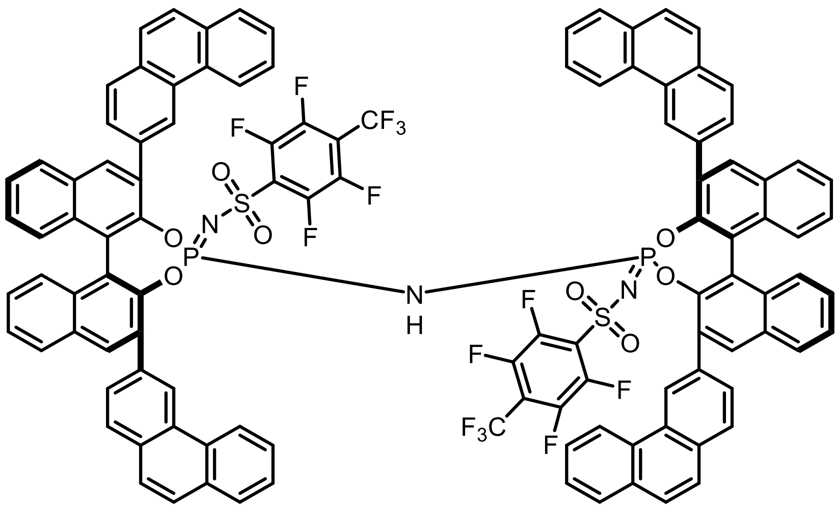

IDPi-3

Yield: 75%

Appearance: white solid;

^{1}H NMR (500 MHz, CD_2Cl_2) δ 8.97 (d, *J* = 1.7 Hz, 2H), 8.77 (d, *J* = 8.2 Hz, 2H), 8.49 (s, 2H), 8.43-8.49 (m, 2H), 8.20 (d, *J* = 8.2 Hz, 2H), 8.11 (s, 2H), 8.08 (d, *J* = 8.2 Hz, 2H), 8.08 (ddd, *J* = 8.2, 5.4, 2.7 Hz, 2H), 7.85 (s, 2H), 7.73-7.78 (m, 6H), 7.51-7.70 (m, 13H), 7.42-7.48 (m, 8H), 7.36 (d, *J* = 9.0 Hz, 2H), 7.12 (d, *J* = 8.8 Hz, 2H), 6.73 (d, *J* = 8.2 Hz, 2H), 6.49 (dd, *J* = 8.3, 1.8 Hz, 4H), 6.05 (d, *J* = 8.4 Hz, 2H), 5.83 (dd, *J* = 8.3, 1.7 Hz, 4H), 5.41 (br, 1H), 5.99 (d, *J* = 7.7 Hz, 2H), 3.50-3.66 (m, 8H);

^{13}C NMR (125 MHz, CD_2Cl_2) δ 144.1, 143.8, 142.5, 134.4, 134.1, 134.0, 132.9, 132.7, 132.6, 132.3, 132.2, 132.1, 131.9, 131.7, 131.4, 130.9, 130.5, 130.3, 130.1, 139.7, 128.9, 128.8, 128.7, 128.5, 128.2, 128.0, 128.0, 127.7, 127.5, 127.4, 127.4, 127.1, 127.1, 127.0, 126.9, 126.8, 126.7, 126.6, 126.5, 124.8, 124.2, 123.8, 122.8, 122.4, 119.1;

^{19}F NMR (471 MHz, CD_2Cl_2): δ -59.96 (t, *J* = 21.7 Hz, 3F), -138.57 (q, *J* = 13.3 Hz, 2F), -138.07 (tq, *J* = 22.3, 9.5 Hz, 2F);

^{31}P NMR (203 MHz, CD_2Cl_2): δ -14.87

HRMS (ESI): m/z calcd for $C_{110}H_{56}N_3O_8F_{14}S_2P_2$ [(M-H)$^-$]: 1938.276614; Found: 1938.279180.

$[\alpha]_D^{25}$ = 138.50 (c = 0.44, CH_2Cl_2)

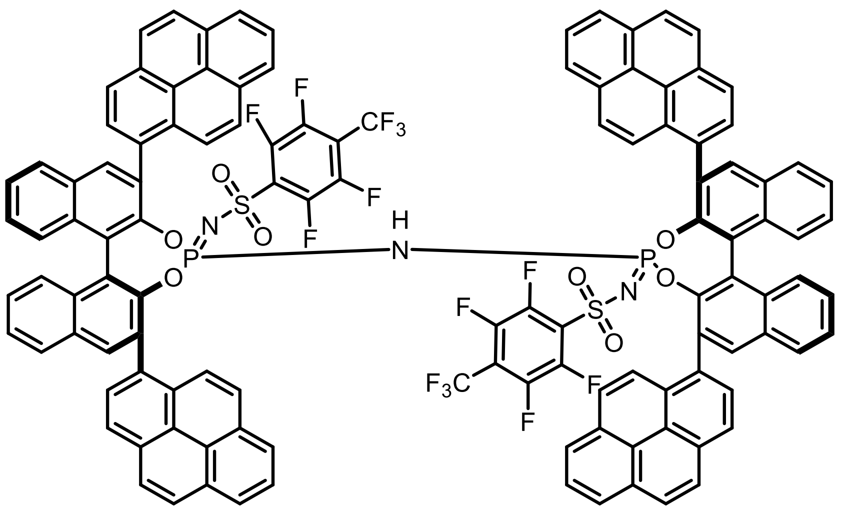

IDPi-4

Yield: 23%

Appearance: light yellow solid;

^{1}H NMR (500 MHz, CD_2Cl_2, mixture of rotamers) δ 6.81-8.32 (m, 46H), 6.81-7.02 (m, 2H), 6.52-6.70 (m, 2H);

^{13}C NMR (125 MHz, CD_2Cl_2) δ 145.3, 144.5, 135.3, 134.4, 134.3, 134.2, 133.5, 133.2, 133.0, 132.5, 132.2, 132.2, 131.8, 131.7, 131.6, 131.4, 131.3, 131.2, 131.1, 130.8, 130.6, 130.6, 129.9, 129.4, 129.4, 129.3, 129.2, 129.0, 128.2, 128.0, 1277.9, 127.9, 127.8, 127.8, 127.7, 127.6, 127.4, 127.3, 127.2, 127.1, 126.9, 126.5, 126.3, 126.1, 125.8, 125.8, 125.7, 125.6, 125.5, 125.0, 124.9, 124.8, 124.6, 124.3, 124.3, 124.2, 123.9, 123.0, 122.0;

^{19}F NMR (471 MHz, CD_2Cl_2, mixture of rotamers, assigned as major rotamer): δ -57.34 (t, *J* = 22.3 Hz, 3F), -136.7 (q, *J* = 12.2 Hz, 2F), -138.68 (tq, *J* = 22.3, 9.5 Hz, 2F);

^{31}P NMR (203 MHz, CD_2Cl_2): δ -10.50

HRMS (ESI): m/z calcd for $C_{118}H_{56}N_3O_8F_{14}S_2P_2$ [$(M-H)^-$]: 2034.279880; Found: 2034.276614.

$[\alpha]_D^{25}$ = 152.38 (c = 0.29, CH_2Cl_2)

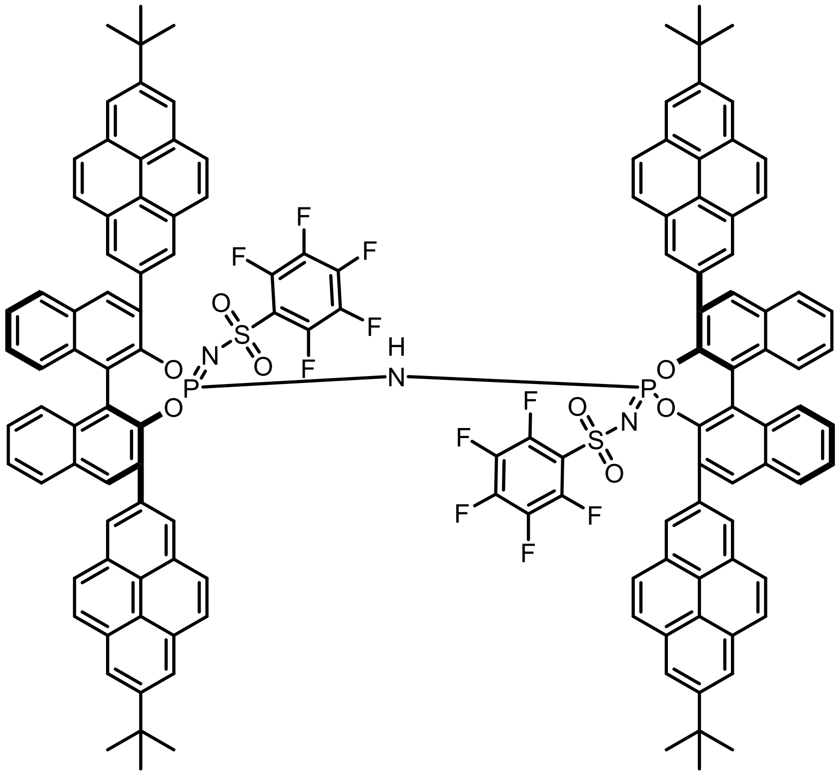

IDPi-6
Yield: 80%
Appearance: light yellow solid;
^{1}H NMR (500 MHz, CD_2Cl_2) δ 8.33 (s, 2H), 8.19 (d, *J* = 7.9 Hz, 2H), 8.11 (d, *J* = 7.6 Hz, 2H), 8.03 (d, *J* = 8.0 Hz, 2H), 7.98 (s, 5H), 7.85-7.92 (m, 11H), 7.76 (d, *J* = 9.0 Hz, 6H), 7.69 (d, *J* = 9.1 Hz, 6H), 7.59 (d, *J* = 9.1 Hz, 6H), 7.73-7.78 (m, 6H), 7.51-7.70 (m, 13H), 7.42-7.48 (m, 8H), 7.36 (d, *J* = 9.0 Hz, 2H), 7.12 (d, *J* = 9.0 Hz, 4H), 7.48 (s, 4H), 7.28 (s, 2H), 7.20 (s, 4H), 7.17 (s, 2H), 6.81 (d, *J* = 9.0 Hz, 2H), 4.21 (br, 1H), 1.51 (s, 18H), 1.21 (s, 18H);
^{13}C NMR (125 MHz, CD_2Cl_2) δ 149.5, 149.4, 145.1, 143.8, 142.6, 137.8, 135.8, 134.6, 133.7, 133.6, 133.2, 132.7, 132.5, 132.3, 132.1, 131.4, 131.2, 131.1, 130.4, 130.3, 129.2, 128.41, 128.1, 127.7, 127.5, 127.3, 127.2, 127.1, 126.9, 126.3, 125.2, 124.4, 123.9, 123.1, 123.0, 122.8, 122.5, 122.1, 122.0, 35.4, 35.2, 31.9, 31.6;
^{19}F NMR (471 MHz, CD_2Cl_2): δ -137.61 (s, 2F), -146.71 (s, 1F), -160.10 (s, 2F);
^{31}P NMR (203 MHz, CD_2Cl_2): δ -16.55
HRMS (ESI): m/z calcd for $C_{132}H_{88}N_3O_8F_{10}S_2P_2$ [(M-H)$^-$]: 2158.533400; Found: 2158.536050.
$[\alpha]_D^{25}$ = 190.03 (c = 0.30, CH_2Cl_2)

10. General procedure for the preparation of enol ethers 2a, 2q-2u

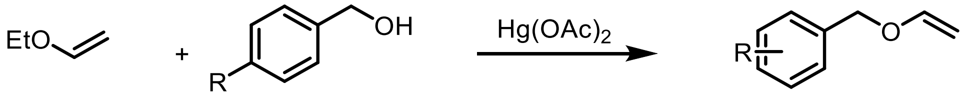

A solution containing benzylic alcohol (10.0 mmol), ethyl vinyl ether (50.0 mmol) and mercury acetate (318 mg, 1.0 mmol) was stirred at reflux for 24 hours. After evaporation of ethyl vinyl ether, the obtained residue was purified by column chromatography to give enol ether as a colorless oil.

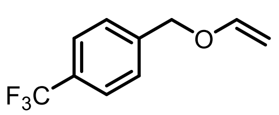

1-(trifluoromethyl)-4-((vinyloxy)methyl)benzene (2a):
Yield: 37%
Appearance: colorless oil.
^{1}H NMR (500 MHz, CD_2Cl_2) δ 7.64 (d, *J* = 8.1 Hz, 2H), 7.50 (d, *J* = 8.0 Hz, 2H), 6.58 (dd, *J* = 14.3, 6.8 Hz, 1H), 4.84 (s, 2H), 4.31 (dd, *J* = 14.3, 2.3 Hz, 1H), 4.12 (dd, *J* = 6.8, 2.3 Hz, 1H);
^{13}C NMR (125 MHz, CD_2Cl_2) δ 151.8, 141.8, 130.1 (q, $^3J_{C\text{-}F}$ = 32.0 Hz, 1C), 127.9, 125.8 (q, $^3J_{C\text{-}F}$ = 7=3.7 Hz, 1C), 124.7 (q, $^1J_{C\text{-}F}$ = 269.8 Hz, 1C), 88.0, 69.5;
^{19}F NMR (471 MHz, CD_2Cl_2): δ -62.86 (s, 3F);
HRMS (EI): m/z calcd for $C_{10}H_9OF_3$ [M^+]: 202.060001; Found: 202.059970.

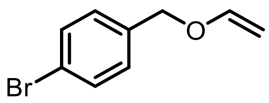

1-methyl-4-((vinyloxy)methyl)benzene (2q)
Yield: 43%
Appearance: colorless oil.
^{1}H NMR (500 MHz, CD_2Cl_2) δ 7.49-7.52 (m, 2H), 7.24-7.26 (m, 2H), 6.55 (dd, *J* = 14.3, 6.8 Hz, 1H), 4.72 (s, 2H), 4.29 (dd, *J* = 14.3, 2.1 Hz, 1H), 4.09 (dd, *J* = 6.9, 2.1 Hz, 1H);
^{13}C NMR (125 MHz, CD_2Cl_2) δ 151.9, 136.7, 131.9, 129.6, 122.0, 87.7, 69.7;
HRMS (EI): m/z calcd for C_9H_9OBr [M^+]: 211.983220; Found: 211.983140.

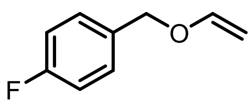

1-fluoro-4-((vinyloxy)methyl)benzene (2r)
Yield: 45%
Appearance: colorless oil.
^{1}H NMR (500 MHz, CD_2Cl_2) δ 7.34 (m, 2H), 7.07 (m, 2H), 6.56 (dd, *J* = 14.3, 6.8 Hz, 1H), 4.73 (s, 2H), 4.30 (dd, *J* = 14.3, 2.111 Hz, 1H), 4.09 (dd, *J* = 6.8, 2.1 Hz, 1H);
^{13}C NMR (125 MHz, CD_2Cl_2) δ 162.9 (d, $^1J_{C\text{-}F}$ = 243.5 Hz, 1C), 152.0, 133.5 (d, $^4J_{C\text{-}F}$ = 3.2 Hz, 2C) 129.8 (d, $^3J_{C\text{-}F}$ = 8.0 Hz, 2C), 115.6 (d, $^4J_{C\text{-}F}$ = 21.5 Hz, 1C), 87.6, 69.8;
^{19}F NMR (471 MHz, CD_2Cl_2): δ -115.26 (s, 1F);
HRMS (EI): m/z calcd for C_9H_9OF [M^+]: 152.063194; Found: 152.063340.

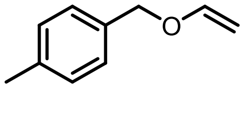

1-methyl-4-((vinyloxy)methyl)benzene (2t)
Yield: 56%
Appearance: colorless oil.

^{1}**H NMR** (500 MHz, CD_2Cl_2) δ 7.24 (d, *J* = 8.0 Hz, 2H), 7.18 (d, *J* = 7.8 Hz, 2H), 6.56 (dd, *J* = 14.3, 6.8 Hz, 1H), 4.71 (s, 2H), 4.29 (dd, *J* = 14.3, 2.0 Hz, 1H), 4.06 (dd, *J* = 6.8, 2.0 Hz, 1H), 2.35 (s, 3H);
^{13}**C NMR** (125 MHz, CD_2Cl_2) δ 152.1, 138.1, 134.4, 129.4, 128.0, 87.3, 70.4, 21.2;
HRMS (EI): m/z calcd for $C_{10}H_{12}O$ [M^+]: 148.088265; Found: 148.088370.

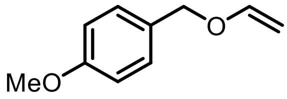

1-methoxy-4-((vinyloxy)methyl)benzene (2u)
Yield: 52%
Appearance: colorless oil.
^{1}**H NMR** (500 MHz, CD_2Cl_2) δ 7.28 (m, 2H), 6.90 (m, 2H), 6.55 (dd, *J* = 14.3, 6.8 Hz, 1H), 4.69 (s, 2H), 4.29 (dd, *J* = 14.3, 2.0 Hz, 1H), 4.06 (dd, *J* = 6.8, 2.0 Hz, 1H), 3.80 (s, 3H);
^{13}**C NMR** (100 MHz, CD_2Cl_2) δ 198.4, 171.4, 143.3, 139.4, 136.0, 136.0, 133.0, 132.2, 130.9, 130.8, 130.1, 128.7, 128.6, 127.4, 127.0, 116.6;
HRMS (EI): m/z calcd for $C_{10}H_{12}O_2$ [M^+]: 164.083180; Found: 164.083330.

11. General procedure for the preparation of α-substituted enol ethers 2b-2o

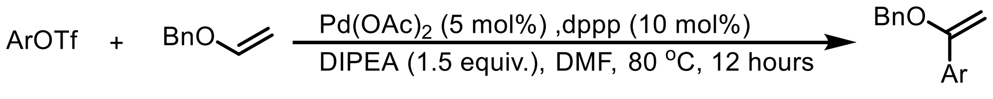

$Pd(OAc)_2$ (0.05 mmol, 5 mol %) and dppp (0.10 mmol, 10 mol %) was suspended in DMF (2.5 mL) in the flame-dried, argon-filled Schlenk flask. Then DIPEA (7.5 mmol), ArOTf (5.5 mmol) and benzyl vinyl ether (5 mmol) were added to the solution at once. The resultant mixture was degassed with Argon for 10 minnutes and then was sealed and heated to 80 °C for 12 hours. Cool the reaction mixture to room temperature and dilute with Et2O (5 mL). Then NaOH (50 mL, 10% aqeous solution) was added dropwie. Separate the organic phase and wash it with NaOH (10 mL of a 10% aqeous solution). Extract the collected water phases with Et2O (10 mL). The combined organic layer was dried over anhydrous Na_2SO_4, filtered, and concentrated under reduced pressure. The residue was purified by flash column chromatography on silica gel (eluting with pentane with 1% v/v TEA) to afford the α-substituted enol ethers.[2]

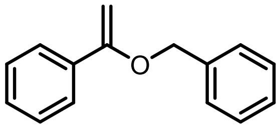

(1-(benzyloxy)vinyl)benzene (2b)
Yield: 82%
Appearance: colorless oil.
^{1}**H NMR** (500 MHz, CD_2Cl_2) δ 7.67-7.69 (m, 2H), 7.47-7.49 (m, 2H), 7.40-7.43 (m, 2H), 7.33-7.38 (m, 4H), 4.98 (s, 2H), 4.77 (d, *J* = 3.0 Hz, 1H), 4.36 (d, *J* = 2.8 Hz, 1H);
^{13}**C NMR** (125 MHz, CD_2Cl_2) δ 160.2, 137.7, 136.8, 128.9, 128.9, 128.5, 128.2, 127.9, 125.8, 83.4, 70.3;
HRMS (EI): m/z calcd for $C_{15}H_{14}O$ [M^+]: 210.103915; Found: 210.103720.

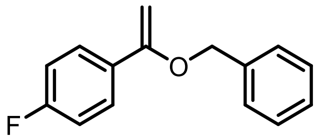

1-(1-(benzyloxy)vinyl)-4-fluorobenzene (2c)
Yield: 46%
Appearance: colorless oil.
^{1}H NMR (500 MHz, CD_2Cl_2) δ 7.63-7.7 (m, 2H), 7.45-7.49 (m, 2H), 7.39-7.43 (m, 2H), 7.31-7.37 (m, 1H), 7.01-7.09 (m, 1H), 4.96 (s, 2H), 4.71 (d, J = 3.2 Hz, 1H), 4.34 (d, J = 3.2 Hz, 1H);
^{13}C NMR (125 MHz, CD_2Cl_2) δ 163.4 (d, $^1J_{C\text{-}F}$ = 245.1 Hz, 1C), 159.3, 137.6, 133.1 (d, $^4J_{C\text{-}F}$ = 3.2 Hz, 1C), 128.9, 128.3, 127.9, 127.7 (d, $^3J_{C\text{-}F}$ = 8.4 Hz, 2C), 115.3 (d, $^2J_{C\text{-}F}$ = 21.6 Hz, 1C), 83.2, 70.3;
^{19}F NMR (471 MHz, CD_2Cl_2): δ -114.3 (s, 1F);
HRMS (EI): m/z calcd for $C_{15}H_{13}OF$ [M$^+$]: 228.094494; Found: 228.094650

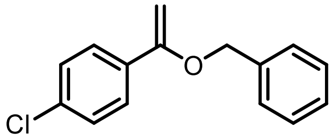

1-(1-(benzyloxy)vinyl)-4-chlorobenzene (2d)
Yield: 57%
Appearance: colorless oil.
^{1}H NMR (500 MHz, CD_2Cl_2) δ 7.61-7.64 (m, 2H), 7.45-7.47 (m, 2H), 7.41 (td, J = 7.6, 1.5 Hz, 2H), 7.32-7.37 (m, 4H), 4.96 (s, 2H), 4.78 (d, J = 3.2 Hz, 1H), 4.38 (d, J = 3.2 Hz, 1H);
^{13}C NMR (125 MHz, CD_2Cl_2) δ 159.1, 137.5, 135.4, 134.6, 128.9, 128.7, 128.3, 128.0, 127.2, 83.8, 70.4;
HRMS (EI): m/z calcd for $C_{15}H_{13}OCl$ [M$^+$]: 244.064943; Found: 244.064610.

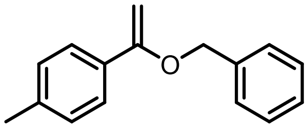

1-(1-(benzyloxy)vinyl)-4-methylbenzene (2e)
Yield: 77%
Appearance: yellow oil.
^{1}H NMR (500 MHz, CD_2Cl_2) δ 7.57-7.60 (m, 2H), 7.48-7.50 (m, 2H), 7.41-7.44 (m, 2H), 7.35-7.38 (m, 1H), 7.38 (d, J = 8.0 Hz, 2H), 4.98 (s, 2H), 4.74 (d, J = 2.8 Hz, 1H), 4.32 (d, J = 2.8 Hz, 1H), 2.37 (s, 3H);
^{13}C NMR (125 MHz, CD_2Cl_2) δ 160.2, 139.0, 137.8, 129.2, 128.9, 128.2, 127.9, 125.6, 82.6, 70.2, 21.3;
HRMS (EI): m/z calcd for $C_{16}H_{16}O$ [M$^+$]: 224.119565; Found: 224.119340.

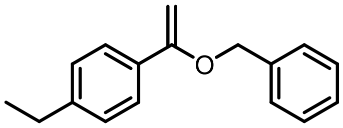

1-(1-(benzyloxy)vinyl)-4-ethylbenzene (2f)
Yield: 83%

Appearance: yellow oil.

^{1}H NMR (500 MHz, CD_2Cl_2) δ 7.57-7.60 (m, 2H), 7.46-7.48 (m, 2H), 7.39-7.42 (m, 2H), 7.32-7.37 (m, 1H), 7.19 (dd, J = 8.4, 2.4 Hz, 2H), 4.96 (s, 2H), 4.72 (t, J = 2.9 Hz, 1H), 4.30 (t, J = 2.7 Hz, 1H), 2.66 (qd, J = 7.6, 2.4 Hz, 2H), 1.23 (td, J = 7.6, 2.4 Hz, 3H);

^{13}C NMR (125 MHz, CD_2Cl_2) δ 160.3, 145.4, 137.8, 134.3, 128.9, 128.2, 128.0, 127.9, 125.7, 82.6, 70.2, 29.0, 15.8;

HRMS (EI): m/z calcd for $C_{17}H_{18}O$ [M$^+$]: 238.135215; Found: 238.135060.

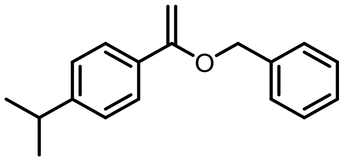

1-(1-(benzyloxy)vinyl)-4-isopropylbenzene (2g)

Yield: 84%

Appearance: colorless oil.

^{1}H NMR (500 MHz, CD_2Cl_2) δ 7.58-7.61 (m, 2H), 7.46-7.48 (m, 2H), 7.7.39-7.43 (m, 2H), 7.31-7.36 (m, 2H), 7.21-7.23 (m, 2H), 4.96 (s, 2H), 4.71-4.73 (m, 1H), 4.29-4.31 (m, 1H), 2.88-2.96 (m, 1H), 1.24-1.26 (m, 6H);

^{13}C NMR (125 MHz, CD_2Cl_2) δ 160.3, 149.9, 137.8, 134.4, 138.9, 128.2, 127.9, 126.6, 125.8, 82.6, 70.2, 34.3, 24.1;

HRMS (EI): m/z calcd for $C_{18}H_{20}O$ [M$^+$]: 252.150865; Found: 252.150830.

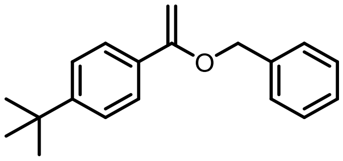

1-(1-(benzyloxy)vinyl)-4-(tert-butyl)benzene (2h)

Yield: 87%

Appearance: white solid;

^{1}H NMR (500 MHz, CD_2Cl_2) δ 7.61-7.63 (m, 2H), 7.48-7.49 (m, 2H), 7.34-7.43 (m, 5H), 4.98 (s, 2H), 4.75 (d, J = 2.8 Hz, 1H), 4.32 (d, J = 2.7 Hz, 1H), 1.35 (s, 9H);

^{13}C NMR (125 MHz, CD_2Cl_2) δ 160.2, 152.1, 137.8, 134.0, 128.9, 128.9, 128.2, 127.9, 125.5, 82.7, 70.2, 34.9, 31.4, ;

HRMS (EI): m/z calcd for $C_{19}H_{22}O$ [M$^+$]: 266.166515; Found: 266.166540.

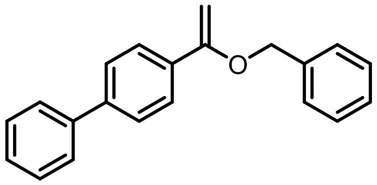

4-(1-(benzyloxy)vinyl)-1,1'-biphenyl (2i)

Yield: 71%

Appearance: white solid;

^{1}H NMR (500 MHz, CD_2Cl_2) δ 7.75-7.78 (m, 2H), 7.60-7.65 (m, 4H), 7.41-7.51 (m, 5H), 7.34-7.38 (m, 2H), 5.00 (s, 2H), 4.83 (d, J = 2.8 Hz, 1H), 4.39 (d, J = 3.0 Hz, 1H);

^{13}C NMR (125 MHz, CD_2Cl_2) δ 159.8, 141.6, 140.9, 137.7, 135.8, 129.2, 128.9, 128.2, 127.9, 127.9, 127.3, 127.2, 126.2, 85.5, 70.3;
HRMS (EI): m/z calcd for $C_{21}H_{18}O$ [M^+]: 286.135215; Found: 286.135430.

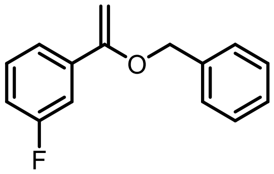

1-(1-(benzyloxy)vinyl)-3-fluorobenzene (2j)
Yield: 36%
Appearance: colorless oil;
^{1}H NMR (500 MHz, CD_2Cl_2) δ 7.45-7.49 (m, 3H), 7.30-7.43 (m, 5H), 7.03 (tdd, J = 8.5, 2.7, 1.1 Hz, 1H), 4.97 (s, 2H), 4.80 (d, J = 3.2 Hz, 1H), 4.40 (d, J = 3.2 Hz, 1H);
^{13}C NMR (125 MHz, CD_2Cl_2) δ 163.2 (d, $^1J_{C\text{-}F}$ = 239.8 Hz, 1C), 158.9 (d, $^4J_{C\text{-}F}$ = 2.6 Hz, 1C), 139.2 (d, $^3J_{C\text{-}F}$ = 7.9 Hz, 1C), 130.1 (d, $^3J_{C\text{-}F}$ = 8.4 Hz, 1C), 115.6 (d, $^2J_{C\text{-}F}$ = 21.0 Hz, 1C), 112.7 (d, $^2J_{C\text{-}F}$ = 23.1 Hz, 1C), 84.4, 70.4;
^{19}F NMR (471 MHz, CD_2Cl_2): δ -114.3 (s, 1F);
HRMS (EI): m/z calcd for $C_{15}H_{13}OF$ [M^+]: 228.094493; Found: 228.094140.

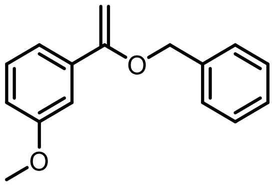

1-(1-(benzyloxy)vinyl)-3-propylbenzene (2k)
Yield: 67%
Appearance: colorless oil.
^{1}H NMR (500 MHz, CD_2Cl_2) δ 7.46 (d, J = 7.6 Hz, 2H), 7.40 (t, J = 7.6 Hz, 2H), 7.32-7.36 (m, 1H), 7.25-7.27 (m, 2H), 7.20-7.22 (m, 1H), 6.84-6.89 (m, 1H), 4.97 (s, 2H), 4.76 (d, J = 3.0 Hz, 1H), 4.35 (d, J = 2.8 Hz, 1H), 3.81 (s, 3H);
^{13}C NMR (125 MHz, CD_2Cl_2) δ 160.0, 138.3, 137.7, 129.5, 128.9, 128.2, 127.8, 118.3, 114.3, 111.6, 83.7, 70.3, 55.6;
HRMS (EI): m/z calcd for $C_{16}H_{16}O_2$ [M^+]: 240.114480; Found: 240.114530.

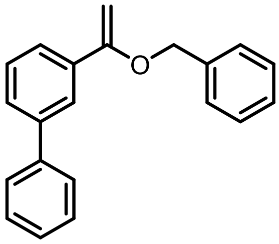

3-(1-(benzyloxy)vinyl)-1,1'-biphenyl (2l)
Yield: 76%
Appearance: white solid.

^{1}H NMR (500 MHz, CD_2Cl_2) δ 7.91 (s, 1H), 7.57-7.68 (m, 4H), 7.33-7.50 (m, 9H), 5.01 (s, 2H), 4.84 (d, *J* = 3.0 Hz, 1H), 4.40 (d, *J* = 3.0 Hz, 1H);
^{13}C NMR (125 MHz, CD_2Cl_2) δ 160.1, 141.5, 141.4, 137.7, 137.4, 129.2, 129.0, 128.9, 128.2, 127.9, 127.8, 127.7, 127.5, 124.8, 124.6, 83.8, 70.3;
HRMS (EI): m/z calcd for $C_{21}H_{18}O$ [M^+]: 286.135215; Found: 286.135280.

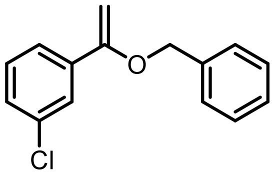

1-(1-(benzyloxy)vinyl)-3-chlorobenzene (2m)
Yield: 42%
Appearance: colorless oil.
^{1}H NMR (500 MHz, CD_2Cl_2) δ 7.66-7.67 (m, 1H), 7.56-7.58 (m, 2H), 7.45-7.47 (m, 2H), 7.40-7.43 (m, 2H), 7.28-7.37 (m, 3H), 6.84-6.89 (m, 1H), 4.97 (s, 2H), 4.79 (d, *J* = 3.2 Hz, 1H), 4.40 (d, *J* = 3.2 Hz, 1H);
^{13}C NMR (125 MHz, CD_2Cl_2) δ 158.8, 138.7, 137.4, 134.5, 129.9, 128.9, 128.8, 128.3, 128.0, 125.9, 124.0;
HRMS (EI): m/z calcd for $C_{15}H_{13}OCl$ [M^+]: 244.064943; Found: 244.065100.

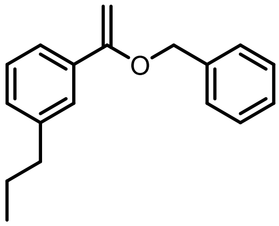

1-(1-(benzyloxy)vinyl)-3-propylbenzene (2n)
Yield: 78%
Appearance: colorless oil.
^{1}H NMR (500 MHz, CD_2Cl_2) δ 7.46-7.49 (m, 4H), 7.39-7.42 (m, 2H), 7.32-7.36 (m, 1H), 7.24-7.27 (m, 1H), 7.15-7.17 (m, 1H), 4.97 (s, 2H), 4.75 (d, *J* = 2.8 Hz, 1H), 4.33 (d, *J* = 2.8 Hz, 1H), 2.61 (t, *J* = 7.7 Hz, 2H), 1.61-1.69 (m, 2H), 0.95 (t, *J* = 7.3 Hz, 3H);
^{13}C NMR (125 MHz, CD_2Cl_2) δ 160.5, 143.1, 137.8, 136.8, 129+.1 ,128.9, 128.4, 128.2, 127.9, 125.9, 123.2, 83.2, 70.2, 38.4, 25.1, 14.0;
HRMS (EI): m/z calcd for $C_{18}H_{20}O$ [M^+]: 252.150865; Found: 252.150710.

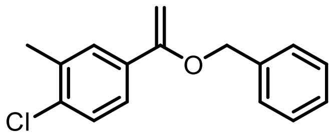

4-(1-(benzyloxy)vinyl)-1-chloro-2-methylbenzene (2o)
Yield: 57%
Appearance: colorless oil.
^{1}H NMR (500 MHz, CD_2Cl_2) δ 7.55 (d, *J* = 2.7 Hz, 1H), 7.39-7.47 (m, 5H), 7.33-7.36 (m, 1H), 7.31 (d, *J* = 8.4 Hz, 1H), 4.95 (s, 2H), 4.74 (d, *J* = 3.0 Hz, 1H), 4.35 (d, *J* = 3.0 Hz, 1H), 2.38 (s, 3H);

^{13}C NMR (125 MHz, CD_2Cl_2) δ 15.9.3, 137.5, 136.2, 135.4, 134.8, 129.1, 128.9, 128.3, 128.2, 128.0, 124.6, 83.7, 70.4, 20.3;
HRMS (EI): m/z calcd for $C_{16}H_{15}OCl$ [M^+]: 258.080593; Found: 258.081030.

12. Procedure for the asymmetric synthesis of 3a-1

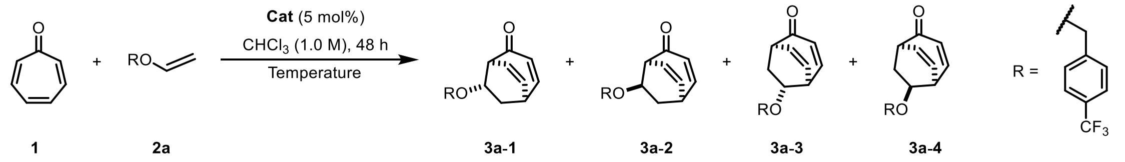

To a solution of tropone (0.10 mmol) and IDPi-**4** (0.0025 mmol) in chloroform (0.1 mL) was added 2a (1.0 mmol) at -20 °C. The resultant mixture was stirred for 7 days and quenched with triethylamine (0.1 mL). After removing the solvent under reduced pressure. The crude residue was subjected to flash column chromatography on silica gel (hexane/ethyl acetate 20: 1 to 10: 1) to afford product **3a.**

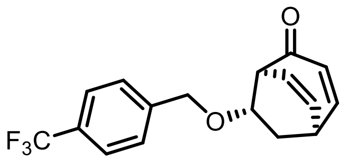

(1S, 5S, 8S)-8-((4-(trifluoromethyl)benzyl)oxy)bicyclo[3.2.2]nona-3,6-dien-2-one (3a-1)
Appearance: white solid.
^{1}H NMR (500 MHz, CD_2Cl_2) δ 7.60 (d, J = 8.4 Hz, 2H), 7.45 (d, J = 8.4 Hz, 2H), 7.08 (dd, J = 11.0, 8.7 Hz, 1H), 6.69 (t, J = 7.8 Hz, 1H), 6.19 (ddt, J = 8.4, 7.1, 1.2Hz, 1H), 5.63 (dd, J = 11.0, 2.2 Hz, 1H), 4.63 (d, J = 12.6 Hz, 2H), 4.55 (d, J = 12.6 Hz, 1H), 4.15 (ddt, J = 8.4, 4.4, 1.3 Hz, 1H), 3.77 (dd, J = 7.2, 1.5 Hz, 1H), 3.26-3.30 (m, 1H), 2.54 (ddd, J = 13.4, 8.4, 2.2 Hz, 1H), 1.74 (dt, J = 13.4, 4.5 Hz, 1H);
^{13}C NMR (125 MHz, CD_2Cl_2) δ 195.2, 143.1, 139.1, 129.8 (q, $^2J_{CF3}$ = 47.3 Hz, 2C), 129.7, 127.9, 125.6 (q, $^2J_{CF3}$ = 4.8 Hz, 2C), 125.0, 124.7 (q, $^2J_{CF3}$ = 269.8 Hz, 1C), 76.3, 70.6, 59.3, 35.7, 35.2;
^{19}F NMR (471 MHz, CD_2Cl_2): δ -62.79 (s, CF_3);
HRMS (EI): m/z calcd for $C_{17}H_{15}F_3O_2$ $[M]^+$: 308.10196; Found: 308.10187.
$[\alpha]_D^{25}$ = 22.59 (c = 0.54, CH_2Cl_2)
HPLC: Daicel Chiralcel IF-3R, MeCN/H_2O = 50/50, 0.5 mL/min, 25 °C, 220 nm, t_R (major) = 29.6 min, t_R (min) = 38.9 min;
e.r. = 99.0: 1.0

13. General procedure for the synthesis of 3b-3o

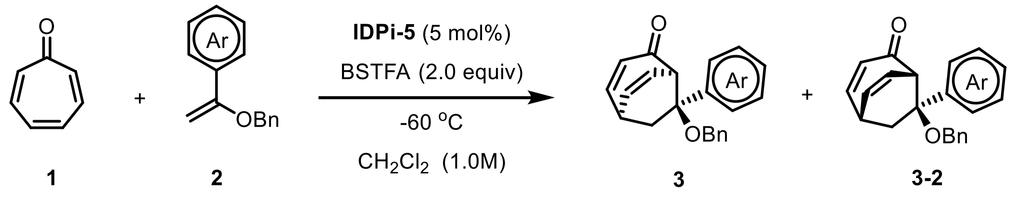

To a solution of BSTFA (0.2 mmol) and IDPi-**5 or** IDPi-**6** (0.005 mmol) in the indicated solvent (0.1 or 0.2 mL) was added tropone and **2** (0.50 mmol) at indicated temperature. The resultant mixture was stirred for indicated time and quenched with triethylamine (0.1 mL). After removal of the solvent under reduced pressure, the crude residue was subjected to flash column chromatography on silica gel (hexane/ethyl acetate 20: 1 to 10: 1) to afford product **3** and **3-2.**

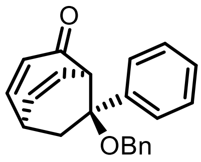

(1R, 5R, 8S)-8-(benzyloxy)-8-phenylbicyclo[3.2.2]nona-3,6-dien-2-one (3b)
Appearance: white solid.
^{1}H NMR (500 MHz, CD_2Cl_2) δ 7.39 (m, 4H), 7.23 (m, 7H), 6.65 (ddd, *J* = 8.2, 7.1, 1.0 Hz, 1H), 6.19 (ddd, *J* = 8.4, 7.5, 1.1 Hz, 1H), 5.86 (ddd, *J* = 10.8, 1.9, 0.8 Hz, 1H), 4.34 (ddd, *J* = 7.6, 1.4, 1.4 Hz, 1H), 4.30 (d, *J* = 10.9 Hz, 1H), 4.03 (d, *J* = 10.9 Hz, 1H), 3.37 (m, 1H), 2.72 (dd, *J* = 14.2, 1.8 Hz, 1H), 2.37 (dd, *J* = 14.1, 5.1 Hz, 1H);
^{13}C NMR (125 MHz, CD_2Cl_2) δ 195.2, 152.6, 145.5, 139.7, 139.0, 130.7, 128.9, 128.5, 128.0, 127.7 (2), 127.0, 126.1, 83.2, 66.1, 63.2, 44.1, 37.4;
HRMS (EI): m/z calcd for $C_{22}H_{20}O_2$ [M]$^+$: 316.145740; Found: 316.145780.
$[\alpha]_D^{25}$ = -83.08 (c = 0.59, CH_2Cl_2)
HPLC: Daicel Chiralcel IH-3R, MeCN/H_2O = 50/50, 0.5 mL/min, 25 °C, 230 nm, t_R (major) = 33.5 min, t_R (min) = 36.7 min;
e.r. = 97.5: 2.5

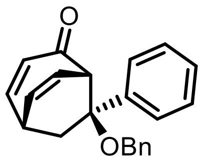

(1S, 5S, 8S)-8-(benzyloxy)-8-phenylbicyclo[3.2.2]nona-3,6-dien-2-one (3b-2)
Appearance: white solid.
^{1}H NMR (500 MHz, CD_2Cl_2) δ 7.52 (m, 2H), 7.37 (m, 2H), 7.27 (m, 6H), 7.13 (dd, *J* = 10.9, 8.8 z, 1H), 6.75 (ddd, *J* = 8.3, 7.0, 1.1 Hz, 1H), 6.25 (ddd, *J* = 8.3, 7.2, 1.1 Hz, 1H), 5.48 (ddd, *J* = 10.9, 2.1, 0.7 Hz, 1H), 4.12 (s, 2H), 3.95 (d, *J* = 7.2 Hz, 1H), 3.49 (m, 1H), 3.02 (dd, *J* = 14.1, 2.0 Hz, 1H), 2.41 (dd, *J* = 14.1, 4.9 Hz, 1H);
^{13}C NMR (125 MHz, CD_2Cl_2) δ 194.4, 152.5, 142.8, 139.3, 138.5, 130.7, 128.7, 128.6, 128.4, 128.1, 127.7, 127.6, 127.2, 83.2, 68.4, 65.6, 37.8, 36.4;
HRMS (EI): m/z calcd for $C_{22}H_{20}O_2$ [M]$^+$: 316.145930; Found: 316.145780.
$[\alpha]_D^{25}$ = 78.89 (c = 0.18, CH_2Cl_2)
HPLC: Daicel Chiralcel IH-3R, MeCN/H_2O = 50/50, 0.5 mL/min, 25 °C, 230 nm, t_R (minor) = 23.5 min, t_R (min) = 25.7 min;
e.r. = 97.0: 3.0

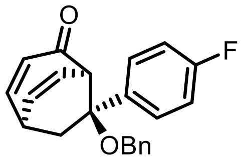

(1R, 5R, 8S)-8-(benzyloxy)-8-(4-fluorophenyl)bicyclo[3.2.2]nona-3,6-dien-2-one (3c)

Appearance: white solid.

^{1}H NMR (500 MHz, CD_2Cl_2) δ 7.39 (m, 2H), 7.23 (m, 6H), 7.07 (m, 2H), 6.65 (ddd, J = 8.3, 7.2, 1.0 Hz, 1H), 6.16 (ddd, J = 10.9, 1.9, 0.8 Hz, 1H), 4.29 (m, 2H), 4.02 (d, J = 10.9 Hz, 2H), 3.38 (m, 1H), 2.71 (dd, J = 14.0, 1.8 Hz, 1H), 2.34 (dd, J = 14.1, 5.1 Hz, 1H);

^{13}C NMR (125 MHz, CD_2Cl_2) δ 195.0, 162.3 (d, $^{1}J_{C\text{-}F}$ = 244.0 Hz, 1C), 152.6, 141.5 (d, $^{4}J_{C\text{-}F}$ = 3.2 Hz, 1C), 139.9, 138.9, 130.6, 128.8 (d, $^{3}J_{C\text{-}F}$ = 7.9 Hz, 2C), 128.6, 128.0, 127.7, 125.9, 115.6 (d, $^{2}J_{C\text{-}F}$ = 21.2 Hz, 2C), 82.8, 66.1, 63.4, 43.9, 37.3;

^{19}F NMR (471 MHz, CD_2Cl_2): δ -116.16 (s, 1F);

HRMS (ESI): m/z calcd for $C_{22}H_{19}FO_2Na$ [(M+Na)$^{+}$]: 357.12613; Found: 357.12639.

$[\alpha]_D^{25}$ = -51.77 (c = 0.17, CH_2Cl_2)

HPLC: Daicel Chiralcel OZ-3R, MeCN/H_2O = 50/50, 0.5 mL/min, 25 °C, 220 nm, t_R (minor) = 27.9 min, t_R (major) = 33.0 min;

e.r. = 96.0: 4.0

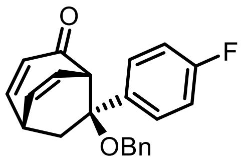

(1S, 5S, 8S)-8-(benzyloxy)-8-(4-fluorophenyl)bicyclo[3.2.2]nona-3,6-dien-2-one (3c-2)

Appearance: colorless viscous oil;

^{1}H NMR (500 MHz, CD_2Cl_2) δ 7.50 (m, 2H), 7.28 (m, 5H), 7.13 (dd, J = 10.9, 8.8 Hz, 1H), 7.05 (m, 2H), 6.74 (m, 1H), 6.24 (ddd, J = 8.2, 7.1, 1.1 Hz, 1H), 5.48 (ddd, J = 10.8, 2.0 Hz, 1H), 4.10 (dd, J = 15.4, 11.6 Hz, 2H), 3.93 (d, J = 7.1 Hz, 1H), 3.49 (q, J = 6.7 Hz, 1H), 2.98 (dd, J = 14.1, 2.1 Hz, 1H), 2.42 (dd, J = 14.1, 4.9 Hz, 1H);

^{13}C NMR (125 MHz, CD_2Cl_2) δ 194.3, 162.6 (d, $^{1}J_{C\text{-}F}$ = 232.3 Hz, 1C), 152.5, 139.1, 138.8 (d, $^{4}J_{C\text{-}F}$ = 3.5 Hz, 1C), 138.5, 130.7, 130.3 (d, $^{3}J_{C\text{-}F}$ = 8.2 Hz, 2C), 128.6, 127.8, 127.7, 127.0, 115.4 (d, $^{2}J_{C\text{-}F}$ = 21.3 Hz, 2C), 82.7, 68.3, 65.6, 38.0, 36.3;

^{19}F NMR (471 MHz, CD_2Cl_2): δ -115.5 (s, 1F);

HRMS (CI): m/z calcd for $C_{22}H_{19}FO_2$ [(M)$^{+}$]: 334.136359; Found: 334.136060.

$[\alpha]_D^{25}$ = 68.80 (c = 0.13, CH_2Cl_2)

HPLC: Daicel Chiralcel OZ-3R, MeCN/H_2O = 50/50, 0.5 mL/min, 25 °C, 220 nm, t_R (major) = 23.7 min, t_R (minor) = 25.6 min;

e.r. = 95.0: 5.0

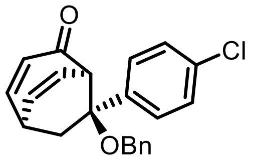

(1R, 5R, 8S)-8-(benzyloxy)-8-(4-chlorophenyl)bicyclo[3.2.2]nona-3,6-dien-2-one (3d)
liAppearance: yellow solid.
^{1}H NMR (500 MHz, CD_2Cl_2) δ 7.36 (s, 4H), 7.24 (m, 6H), 6.66 (ddd, *J* = 8.2, 7.2, 1.0 Hz, 1H), 6.16 (t, *J* = 7.9 Hz, 1H), 5.86 (dd, *J* = 11.0, 1.1 Hz, 1H), 4.28 (m, 2H), 4.03 (d, *J* = 10.9 Hz, 1H), 3.38 (m, 1H), 2.71 (dd, *J* = 14.1, 1.9 Hz, 1H), 2.34 (dd, *J* = 14.1, 5.2 Hz, 1H);
^{13}C NMR (125 MHz, CD_2Cl_2) δ 194.9, 152.6, 144.2, 140.0, 138.7, 133.4, 130.6, 129.0, 128.6, 128.6, 127.9, 127.8, 125.9, 82.8, 66.2, 63.3, 43.8, 37.2;
HRMS (ESI): m/z calcd for $C_{22}H_{19}C_{l1}O_2Na$ [(M+Na)$^+$]: 373.09707; Found: 373.09658.
$[\alpha]_D^{25}$ = -73.85 (c = 0.78, CH_2Cl_2)
HPLC: Daicel Chiralcel OJ-3R, MeCN/H_2O = 50/50, 1.0 mL/min, 25 °C, 254 nm, t_R (minor) = 23.2 min, t_R (major) = 28.2 min;
e.r. = 96.0: 4.0

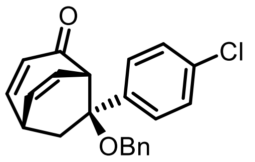

(1S, 5S, 8S)-8-(benzyloxy)-8-(4-chlorophenyl)bicyclo[3.2.2]nona-3,6-dien-2-one (3d-2)
liAppearance: yellow viscous oil;
^{1}H NMR (500 MHz, CD_2Cl_2) δ 7.47 (m, 2H), 7.29 (m, 7H), 7.13 (dd, *J* = 10.9, 8.8 Hz, 1H), 6.75 (ddd, *J* = 8.3, 7.1, 1.1 Hz, 1H), 6.24 (ddd, *J* = 8.3, 7.1, 1.1 Hz, 1H), 5.49 (dd, *J* = 10.9, 2.0, 0.7 Hz, 1H), 4.11 (d, *J* = 18.4, 11.5 Hz, 1H), 3.92 (d, *J* = 7.3 Hz, 1H), 3.49 (m, 1H), 2.96 (dd, *J* = 14.1, 2.1 Hz, 1H), 2.42 (dd, *J* = 14.1, 4.9 Hz, 1H);
^{13}C NMR (125 MHz, CD_2Cl_2) δ 194.2, 152.6, 141.6, 139.0, 138.6, 134.0, 130.7, 129.9, 128.8, 128.7, 127.8, 127.6, 127.0, 82.8, 68.3, 65.7, 37.8, 36.2;
HRMS (EI): m/z calcd for $C_{22}H_{19}O_2C_{l1}$ [M]$^+$: 350.107030; Found: 350.106808.
$[\alpha]_D^{25}$ = 96.25 (c = 0.15, CH_2Cl_2)
HPLC: Daicel Chiralcel OJ-3R, MeCN/H_2O = 50/50, 1.0 mL/min, 25 °C, 254 nm, t_R (minor) = 19.4 min, t_R (major) = 41.8 min;
e.r. = 95.5: 4.5

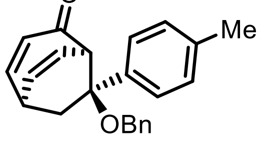

(1R, 5R, 8S)-8-(benzyloxy)-8-(p-tolyl)bicyclo[3.2.2]nona-3,6-dien-2-one (3e)
Appearance: light yellow solid.

^{1}H NMR (500 MHz, CD_2Cl_2) δ 7.25 (m, 11H), 6.63 (ddd, *J* = 8.2, 7.1, 1.0 Hz, 1H), 6.17 (ddd, *J* = 8.4, 7.5, 1.1 Hz, 1H), 5.85 (ddd, *J* = 10.9, 1.9, 0.8 Hz, 1H), 4.34 (d, *J* = 7.7 Hz, 1H), 4.29 (d, *J* = 10.9 Hz, 1H), 4.03 (d, *J* = 10.9 Hz, 1H), 3.36 (m, 1H), 2.71 (dd, *J* = 14.1, 1.8 Hz, 1H), 2.65 (q, *J* = 7.6 Hz, 2H) 2.36 (dd, *J* = 14.1, 5.1 Hz, 1H), 1.24 (t, *J* = 7.6 Hz, 3H);
^{13}C NMR (125 MHz, CD_2Cl_2) δ 195.4, 152.7, 143.9 142.7, 139.6, 139.1, 130.7, 128.5, 128.3, 128.0, 127.6, 127.0, 126.2, 83.1, 66.0, 63.2, 44.0, 37.4, 28.7, 15.7;
HRMS (EI): m/z calcd for $C_{23}H_{22}O_2$ $[(M)^+]$: 330.161430; Found: 330.161800.
$[\alpha]_D^{25}$ = -73.17 (c = 0.80, CH_2Cl_2)
HPLC: Daicel Chiralcel OZ-3R, MeCN/H_2O = 50/50, 0.5 mL/min, 25 °C, 230 nm, t_R (minor) = 35.6 min, t_R (major) = 42.9 min;
e.r. = 98.0: 2.0

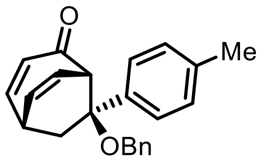

(1S, 5S, 8S)-8-(benzyloxy)-8-(p-tolyl)bicyclo[3.2.2]nona-3,6-dien-2-one (3e-2)
Appearance: yellow viscous oil;
^{1}H NMR (500 MHz, CD_2Cl_2) δ 7.38 (m, 2H), 7.30 (m, 2H), 7.24 (m, 2H), 7.17 (d, *J* = 7.9 Hz, 2H), 7.11 (dd, *J* = 10.9, 8.8 Hz, 1H), 6.73 (ddd, *J* = 8.2, 7.0, 1.1 Hz, 1H), 6.24 (ddd, *J* = 8.2, 7.1, 1.1 Hz, 1H), 5.47 (dd, *J* = 10.9, 2.1 Hz, 1H), 4.10 (s, 2H), 3.93 (d, *J* = 7.2 Hz, 1H), 3.46-3.50 (m, 1H), 3.00 (dd, *J* = 14.0, 2.1 Hz, 1H), 2.38 (dd, *J* =14.1, 4.9 Hz, 1H), 2.33 (s, 3H);
^{13}C NMR (125 MHz, CD_2Cl_2) δ 194.5, 152.5, 139.7, 139.4, 138.5, 138.0, 130.7, 129.3, 128.6, 128.3, 127.6, 127.2, 83.0, 68.4, 65.5, 37.7, 36.4, 21.1;
HRMS (EI): m/z calcd for $C_{23}H_{22}O_2$ $[(M)^+]$: 330.161430; Found: 330.161700.
$[\alpha]_D^{25}$ = 85.19 (c = 0.27, CH_2Cl_2)
HPLC: Daicel Chiralcel OZ-3R, MeCN/H_2O = 50/50, 0.5 mL/min, 25 °C, 230 nm, t_R (major) = 30.8 min, t_R (min) = 33.7 min;
e.r. = 97.5: 2.5

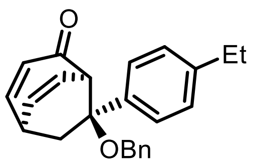

(1R,5R,8S)-8-(benzyloxy)-8-(4-ethylphenyl)bicyclo[3.2.2]nona-3,6-dien-2-one (3f)
Appearance: white solid.
^{1}H NMR (500 MHz, CD_2Cl_2) δ 7.25 (m, 11H), 6.63 (ddd, *J* = 8.2, 7.1, 1.0 Hz, 1H), 6.17 (ddd, *J* = 8.4, 7.5, 1.1 Hz, 1H), 5.85 (ddd, *J* = 10.9, 1.9, 0.8 Hz, 1H), 4.34 (d, *J* = 7.7 Hz, 1H), 4.29 (d, *J* = 10.9 Hz, 1H), 4.03 (d, *J* = 10.9 Hz, 1H), 3.36 (m, 1H), 2.71 (dd, *J* = 14.1, 1.8 Hz, 1H), 2.65 (q, *J* = 7.6 Hz, 2H) 2.36 (dd, *J* = 14.1, 5.1 Hz, 1H), 1.24 (t, *J* = 7.6 Hz, 3H);
^{13}C NMR (125 MHz, CD_2Cl_2) δ 195.4, 152.7, 143.9 142.7, 139.6, 139.1, 130.7, 128.5, 128.3, 128.0, 127.6, 127.0, 126.2, 83.1, 66.0, 63.2, 44.0, 37.4, 28.7, 15.7;

HRMS (EI): m/z calcd for $C_{24}H_{24}O_2$ $[(M)^+]$: 344.177080; Found: 344.177230.
$[\alpha]_D^{25}$ = -64.52 (c = 0.93, CH_2Cl_2)
HPLC: Daicel Chiralcel OJ-3R, MeCN/H_2O = 50/50, 1.0 mL/min, 25 °C, 230 nm, t_R (minor) = 23.8 min, t_R (major) = 26.5 min;
e.r. = 98.0: 2.0

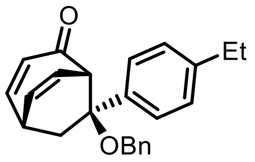

(1S, 5S, 8S)-8-(benzyloxy)-8-(4-ethylphenyl)bicyclo[3.2.2]nona-3,6-dien-2-one (3f-2)
Appearance: white solid.
^{1}H NMR (500 MHz, CD_2Cl_2) δ 7.40 (m, 2H), 7.26 (m, 2H), 7.12 (dd, *J* = 10.9, 8.8 Hz, 1H), 6.73 (ddd, *J* = 8.2, 7.1, 1.1 Hz, 1H), 6.25 (ddd, *J* = 8.3, 7.2, 1.1 Hz, 1H), 5.47 (ddd, *J* = 10.9, 2.1, 0.8 Hz, 1H), 4.11 (d, *J* = 14.7, 11.7 Hz, 2H), 3.94 (d, *J* = 7.1 Hz, 1H), 3.48 (m, 1H), 3.00 (dd, *J* = 14.1, 2.0 Hz, 1H), 2.65 (q, *J* = 7.6 Hz, 2H) 2.39 (dd, *J* = 14.0, 4.9 Hz, 1H), 1.23 (t, *J* = 7.6 Hz, 3H);
^{13}C NMR (125 MHz, CD_2Cl_2) δ 194.5, 152.5, 144.2, 140.0, 139.4, 138.4, 130.7, 128.6, 128.4, 128.1, 127.6, 127.2, 83.0, 68.4, 65.5, 37.9, 36.4, 28.7, 15.4;
HRMS (EI): m/z calcd for $C_{24}H_{24}O_2$ $[(M)^+]$: 344.177080; Found: 344.177160.
$[\alpha]_D^{25}$ = 83.39 (c = 0.30, CH_2Cl_2)
HPLC: Daicel Chiralcel OJ-3R, MeCN/H_2O = 50/50, 1.0 mL/min, 25 °C, 230 nm, t_R (minor) = 18.5 min, t_R (major) = 21.8 min;
e.r. = 94.0: 6.0

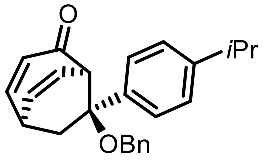

(1R, 5R, 8S)-8-(benzyloxy)-8-(4-isopropylphenyl)bicyclo[3.2.2]nona-3,6-dien-2-one (3g)
Appearance: white solid.
^{1}H NMR (500 MHz, CD_2Cl_2) δ 7.25 (m, 11H), 6.63 (ddd, *J* = 8.2, 7.1, 1.0 Hz, 1H), 6.18 (ddd, *J* = 8.5, 7.5, 1.1 Hz, 1H), 5.85 (ddd, *J* = 10.9, 2.0, 0.8 Hz, 1H), 4.34 (d, *J* = 7.6 Hz, 1H), 4.29 (d, *J* = 10.9 Hz, 1H), 4.03 (d, *J* = 10.9 Hz, 1H), 3.36 (m, 1H), 2.91 (hept, *J* = 6.9 Hz, 1H), 2.71 (dd, *J* = 14.0, 1.8 Hz, 1H), 2.37 (dd, *J* = 14.1, 5.1 Hz, 1H), 1.25 (d, *J* = 6.9 Hz, 6H);
^{13}C NMR (125 MHz, CD_2Cl_2) δ 195.4, 152.7, 148.5, 142.8, 139.6, 139.2, 130.7, 128.5, 128.0, 127.6, 127.0, 126.9, 126.2, 83.0, 66.0, 63.2, 44.1, 37.4, 34.1, 24.1;
HRMS (CI): m/z calcd for $C_{25}H_{26}O_2$ $[M]^+$: 358.192730; Found: 358.192750.
$[\alpha]_D^{25}$ = -73.72 (c = 0.86, CH_2Cl_2)
HPLC: Daicel Chiralcel OJ-3R, MeCN/H_2O = 50/50, 1.0 mL/min, 25 °C, 254 nm, t_R (minor) = 22.9 min, t_R (major) = 32.0 min;
e.r. = 97.0: 3.0

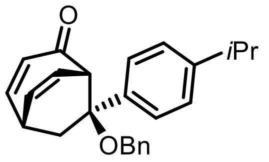

(1S, 5S, 8S)-8-(benzyloxy)-8-(4-isopropylphenyl)bicyclo[3.2.2]nona-3,6-dien-2-one (3g-2)
Appearance: white viscous oil.
^{1}H NMR (500 MHz, CD_2Cl_2) δ 7.42 (m, 2H), 7.30 (m, 2H), 7.23 (m, 5H), 7.12 (ddd, *J* = 8.2, 7.1, 1.1 Hz, 1H), 6.73 (ddd, *J* = 8.3, 7.2, 1.1 Hz, 1H), 6.25 (ddd, *J* = 8.3, 7.2, 1.1 Hz, 1H), 5.49 (ddd, *J* = 10.9, 2.0, 0.7 Hz, 1H), 4.11 (dd, *J* = 17.7, 11.6 Hz, 1H), 3.96 (d, *J* = 7.3 Hz, 1H), 3.48 (m, 1H), 3.00 (dd, *J* = 14.0, 2.0 Hz, 1H), 2.90 (hept, *J* = 6.9 Hz, 1H), 2.40 (dd, *J* = 14.1, 5.0 Hz, 1H), 2.37 (dd, *J* = 14.1, 5.1 Hz, 1H), 1.25 (d, *J* = 6.9 Hz, 6H);
^{13}C NMR (125 MHz, CD_2Cl_2) δ 194.5, 152.6, 148.7, 140.1, 139.4, 138.4, 130.6, 128.6, 128.3, 127.6, 127.3, 126.7, 83.0, 68.2, 65.5, 38.0, 36.4, 34.0, 24.0;
HRMS (ESI): m/z calcd for $C_{25}H_{26}O_2Na$ [$(M+Na)^+$]: 381.18250; Found: 381.18264.
$[\alpha]_D^{25}$ = 72.90 (c = 0.31, CH_2Cl_2)
HPLC: Daicel Chiralcel OJ-3R, MeCN/H_2O = 50/50, 1.0 mL/min, 25 °C, 254 nm, t_R (major) = 19.0 min, t_R (minor) = 20.8 min;
e.r. = 95.0: 5.0

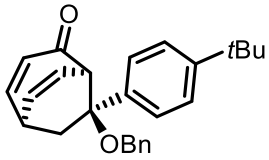

(1R, 5R, 8S)-8-(benzyloxy)-8-(4-(tert-butyl)phenyl)bicyclo[3.2.2]nona-3,6-dien-2-one (3h)
Appearance: white solid.
^{1}H NMR (500 MHz, CD_2Cl_2) δ 7.39 (m, 2H), 7.33 (m, 2H), 7.23 (m, 6H), 6.63 (ddd, *J* = 8.2, 7.1, 1.1 Hz, 1H), 6.18 (ddd, *J* = 8.4, 7.5, 1.1 Hz, 1H), 5.85 (dd, *J* = 10.6, 1.3 Hz, 1H), 4.34 (d, *J* = 7.4 Hz, 1H), 4.29 (d, *J* = 10.9 Hz, 1H), 4.03 (d, *J* = 10.9 Hz, 1H), 3.36 (m, 1H), 2.71 (dd, *J* = 14.1, 1.8 Hz, 1H), 2.36 (dd, *J* = 14.1, 5.1 Hz, 1H), 1.32 (s, 9H);
^{13}C NMR (125 MHz, CD_2Cl_2) δ 195.4, 152.7, 150.7, 142.4, 139.6, 139.2, 130.7, 128.5, 128.0, 127.6, 126.7, 126.2, 125.8, 83.0, 66.0, 63.2, 44.0, 37.4, 34.8, 31.5, ;
HRMS (CI): m/z calcd for $C_{26}H_{28}O_2$ $[M]^+$: 372.208380; Found: 372.208520.
$[\alpha]_D^{25}$ = -66.42 (c = 0.83, CH_2Cl_2)
HPLC: Daicel Chiralcel OZ-3R, MeCN/H_2O = 50/50, 1.0 mL/min, 25 °C, 230 nm, t_R (minor) = 34.8 min, t_R (major) = 44.6 min;
e.r. = 97.5: 2.5

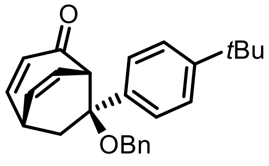

(1S, 5S, 8S)-8-(benzyloxy)-8-(4-(tert-butyl)phenyl)bicyclo[3.2.2]nona-3,6-dien-2-one (3h-2)

Appearance: light yellow solid.

^{1}H NMR (500 MHz, CD_2Cl_2) δ 7.42 (m, 2H), 7.38 (m, 2H), 7.28 (m, 5H), 7.13 (dd, *J* = 10.9, 0.8 Hz, 1H), 6.73 (ddd, *J* = 8.2, 7.1, 1.1 Hz, 1H), 6.25 (ddd, *J* = 8.3, 7.2, 1.1 Hz, 1H), 5.49 (ddd, *J* = 11.0, 2.1, 0.8 Hz, 1H), 4.10 (m, 2H), 3.97 (d, *J* = 7.2 Hz, 1H), 3.48 (m, 1H), 2.99 (dd, *J* = 14.1, 2.0 Hz, 1H), 2.40 (dd, *J* = 14.1, 5.1 Hz, 1H), 1.31 (s, 9H);

^{13}C NMR (125 MHz, CD_2Cl_2) δ 195.5, 160.4, 152.6, 147.2, 139.7, 139.1, 130.7, 129.8, 128.6, 128.0, 127.7, 126.1, 119.2, 112.9, 112.9, 83.2, 66.2, 6;

HRMS (CI): m/z calcd for $C_{26}H_{28}O_2$ $[M]^+$: 372.208380; Found: 372.208550.

$[\alpha]_D^{25}$ = 61.33 (c = 0.23, CH_2Cl_2)

HPLC: Daicel Chiralcel OZ-3R, MeCN/H_2O = 50/50, 1.0 mL/min, 25 °C, 230 nm, t_R (major) = 34.8 min, t_R (minor) = 44.6 min;

e.r. = 95.0: 5.0

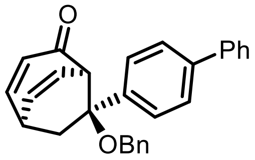

(1R, 5R, 8S)-8-([1,1'-biphenyl]-4-yl)-8-(benzyloxy)bicyclo[3.2.2]nona-3,6-dien-2-one (3i)

Appearance: white solid.

^{1}H NMR (500 MHz, CD_2Cl_2) δ 7.62 (m, 4H), 7.46 (m, 4H), 7.28 (m, 7H), 6.67 (ddd, *J* = 8.2, 7.1, 1.0 Hz, 1H), 6.22 (m, 1H), 5.87 (ddd, *J* = 10.9, 2.0, 0.8 Hz, 1H), 4.38 (d, *J* = 7.6 Hz, 1H), 4.35 (d, *J* = 10.9 Hz, 1H), 4.10 (d, *J* = 10.9 Hz, 1H), 3.40 (m, 1H), 2.76 (dd, *J* = 14.1, 1.8 Hz, 1H), 2.42 (dd, *J* = 14.1, 5.1 Hz, 1H);

^{13}C NMR (125 MHz, CD_2Cl_2) δ 195.2, 152.7, 144.6, 140.8, 140.5, 139.8, 139.0, 130.7, 129.2, 128.6, 128.0, 127.8, 127.7, 127.6, 127.5, 127.3, 126.1, 83.1, 66.2, 63.3, 44.0, 37.4;

HRMS (ESI): m/z calcd for $C_{28}H_{24}O_2Na$ $[(M+Na)^+]$: 415.16685; Found: 415.16735.

$[\alpha]_D^{25}$ = -57.50 (c = 0.24, CH_2Cl_2)

HPLC: Daicel Chiralcel OZ-3R, MeCN/H_2O = 50/50, 1.0 mL/min, 25 °C, 220 nm, t_R (minor) = 44.0 min, t_R (min) = 54.6 min;

e.r. = 96.0: 4.0

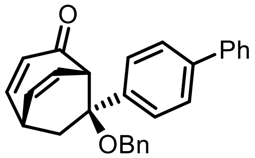

(1S, 5S, 8S)-8-([1,1'-biphenyl]-4-yl)-8-(benzyloxy)bicyclo[3.2.2]nona-3,6-dien-2-one (3i-2)

Appearance: light yellow solid.

^{1}H NMR (500 MHz, CD_2Cl_2) δ 7.61 (m, 6H), 7.45 (t, *J* = 7.6 Hz, 4H), 7.30 (m, 6H), 7.16 (dd, *J* = 10.9, 8.8 Hz, 1H) 6.76 (t, *J* = 7.7 Hz, 1H), 6.28 (t, *J* = 7.7 Hz, 1H), 5.52 (dd, *J* = 11.0, 2.0 Hz, 1H), 4.16 (d, *J* = 2.2 Hz, 2H), 4.01 (d, *J* = 6.5 Hz, 1H), 3.52 (q, *J* = 7.3 Hz, 1H), 3.06 (dd, *J* = 14.2, 2.1 Hz, 1H), 2.45 (dd, *J* = 14.2, 5.0 Hz, 1H);

^{13}C NMR (125 MHz, CD_2Cl_2) δ 194.0, 152.2, 141.5, 140.3, 140.2, 138.9, 138.1, 130.3, 128.8, 128.5, 128.2, 127.4, 127.3 (2), 126.9, 126.8, 126.7, 82.6, 67.8, 65.3, 37.5, 35.9;

HRMS (ESI): m/z calcd for $C_{28}H_{24}O_2Na$ $[(M+Na)^+]$: 415.16685; Found: 415.16730.

$[\alpha]_D^{25}$ = 97.33 (c = 0.15, CH_2Cl_2)
HPLC: Daicel Chiralcel OJ-3R, $MeCN/H_2O$ = 50/50, 0.5 mL/min, 25 °C, 220 nm, t_R (minor) = 49.2 min, t_R (major) = 58.3 min;
e.r. = 95.0: 5.0

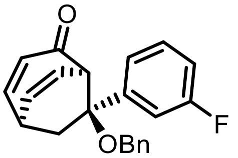

(1R, 5R, 8S)-8-(benzyloxy)-8-(3-fluorophenyl)bicyclo[3.2.2]nona-3,6-dien-2-one (3j)
Appearance: light yellow solid.
^{1}H NMR (500 MHz, CD_2Cl_2) δ 7.12-7.38 (m, 9H), 6.99 (tdd, *J* = 8.3, 2.6, 0.9 Hz, 1H), 6.67 (t, *J* = 7.5 Hz, 1H), 6.18 (t, *J* = 7.9 Hz, 1H), 5.86 (dd, *J* = 11.1, 1.8 Hz, 1H), 4.28-4.33 (m, 2H), 4.06 (d, *J* = 10.9 Hz, 2H), 3.36-3.40 (m, 1H), 2.71 (dd, *J* = 14.2, 1.8 Hz, 1H), 2.33 (dd, *J* = 14.2, 5.1 Hz, 1H);
^{13}C NMR (125 MHz, CD_2Cl_2) δ 194.8, 163.5 (d, $^1J_{C-F}$ = 243.5 Hz, 1C), 152.5, 148.5 (d, $^3J_{C-F}$ = 6.3 Hz, 1C), 140.0, 138.7, 130.7, 130.5 (d, $^3J_{C-F}$ = 8.2 Hz, 1C), 128.6, 128.0, 127.7, 125.9, 122.6 (d, $^4J_{C-F}$ = 2.8 Hz, 1C), 114.6 (d, $^2J_{C-F}$ = 20.8 Hz, 1C), 114.1 (d, $^2J_{C-F}$ = 22.5 Hz, 1C), 82.9, 66.3, 63.2, 43.9, 37.3;
^{19}F NMR (471 MHz, CD_2Cl_2): δ -113.24 (s, 1F);
HRMS (ESI): m/z calcd for $C_{22}H_{19}FO_2$ [(M-H)$^-$]: 334.136358; Found: 334.136540.
$[\alpha]_D^{25}$ = -76.23 (c = 0.53, CH_2Cl_2)
HPLC: Daicel Chiralcel OZ-3R, $MeCN/H_2O$ = 50/50, 1.0 mL/min, 25 °C, 230 nm, t_R (minor) = 14.9 min, t_R (major) = 17.4 min;
e.r. = 96.5: 3.5

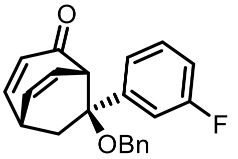

(1S, 5S, 8S)-8-(benzyloxy)-8-(3-fluorophenyl)bicyclo[3.2.2]nona-3,6-dien-2-one (3j-2)
Appearance: yellow viscous oil.
^{1}H NMR (500 MHz, CD_2Cl_2) δ7.21-7.37 (m, 8H), 7.14 (dd, *J* = 10.9, 8.8 Hz, 1H), 7.00 (tdd, *J* = 8.1, 2.6, 1.2 Hz, 1H), 6.75 (ddd, *J* = 8.3, 7.0, 1.1 Hz, 1H), 6.25 (ddd, *J* = 8.4, 7.2, 1.1 Hz, 1H), 5.51 (dd, *J* = 11.0, 1.9 Hz, 1H), 4.14 (s, 2H), 3.91 (d, *J* = 7.3 Hz, 1H), 3.47-3.52 (m, 1H), 2.94 (dd, *J* = 14.2, 2.0 Hz, 1H), 2.43 (dd, *J* = 14.2, 4.9 Hz, 1H);
^{13}C NMR (125 MHz, CD_2Cl_2) δ 194.1, 163.3 (d, $^1J_{C-F}$ = 243.5 Hz, 1C), 152.6, 145.9, 139.0, 138.5, 130.7, 130.2 (d, $^3J_{C-F}$ = 8.0 Hz, 1C), 128.7, 127.8, 127.6, 127.0, 124.1 (d, $^4J_{C-F}$ = 2.7 Hz, 1C), 115.5 (d, $^2J_{C-F}$ = 22.4 Hz, 1C), 115.0 (d, $^2J_{C-F}$ = 21.1 Hz, 1C), 83.0, 68.4, 65.8, 37.9, 36.2, ;
^{19}F NMR (471 MHz, CD_2Cl_2): δ -113.3 (s, 1F);
HRMS (ESI): m/z calcd for $C_{22}H_{19}FO_2$ [(M-H)$^-$]: 334.136358; Found: 334.136530.
$[\alpha]_D^{25}$ = 87.27 (c = 0.22, CH_2Cl_2)
HPLC: Daicel Chiralcel OZ-3R, $MeCN/H_2O$ = 50/50, 1.0 mL/min, 25 °C, 230 nm, t_R (major) = 11.7 min, t_R (minor) = 13.7 min;
e.r. = 95.0: 5.0

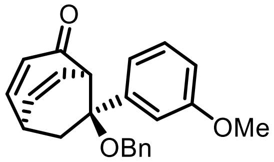

(1R, 5R, 8S)-8-(benzyloxy)-8-(3-methoxyphenyl)bicyclo[3.2.2]nona-3,6-dien-2-one (3k)

Appearance: colorless viscous oil.

^{1}H NMR (500 MHz, CD_2Cl_2) δ 7.15-7.30 (m, 6H), 6.95-6.98 (m, 2H), 6.81 (ddd, J = 8.2, 2.5, 0.9 Hz, 1H), 6.64 (ddd, J = 8.3, 7.2, 1.0 Hz, 1H), 6.19 (ddd, J = 8.4, 7.5, 1.1 Hz, 1H), 5.85 (ddd, J = 10.9, 1.9, 0.8 Hz, 1H), 4.30-4.33 (m, 2H), 4.07 (d, J = 10.9 Hz, 1H), 3.77 (s, 3H), 3.34-3.39 (m, 1H), 2.70 (dd, J = 14.1, 1.8 Hz, 1H), 2.34 (dd, J = 14.1, 5.1 Hz, 1H);

^{13}C NMR (125 MHz, CD_2Cl_2) δ 195.4, 152.7, 150.7, 142.4, 139.6, 139.2, 130.7, 128.5, 128.0, 127.6, 126.7, 126.2, 125.8, 83.0, 66.0, 63.2, 44.0, 37.4, 34.8, 31.5;

HRMS (EI): m/z calcd for $C_{22}H_{23}O_2$ [(M)$^+$]: 346.156345; Found: 346.156440.

$[\alpha]_D^{25}$ = -55.17 (c = 0.44, CH_2Cl_2)

HPLC: Daicel Chiralcel OZ-3R, MeCN/H_2O = 50/50, 1.0 mL/min, 25 °C, 230 nm, t_R (minor) = 14.7 min, t_R (minor) = 17.4 min;

e.r. = 96.5: 3.5

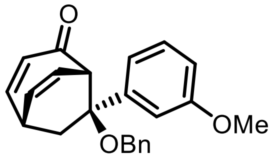

(1S, 5S, 8S)-8-(benzyloxy)-8-(3-methoxyphenyl)bicyclo[3.2.2]nona-3,6-dien-2-one (3k-2)

Appearance: colorless viscous oil.

^{1}H NMR (500 MHz, CD_2Cl_2) δ 7.23-7.32 (m, 6H), 7.04-7.15 (m, 3H), 6.82 (dd, J = 8.2, 2.6 Hz, 1H), 6.74 (t, J = 7.7 Hz, 1H), 6.24 (ddd, J = 8.3, 7.1, 1.1 Hz, 1H), 5.51 (dd, J = 10.8, 2.1 Hz, 1H), 4.15 (dd J = 14.6 11.7 Hz, 2H), 3.93 (d, J = 7.2 Hz, 1H), 3.77 (s, 3H), 3.46-3.50 (m, 1H), 2.97 (dd, J = 14.1, 2.1 Hz, 1H), 2.40 (dd, J = 14.2, 5.0 Hz, 1H);

^{13}C NMR (125 MHz, CD_2Cl_2) δ 195.2, 160.4, 152.6, 147.2, 139.7, 139.1, 130.7, 129.8, 128.6, 128.0, 127.7, 126.1, 119.2, 112.9, 112.9, 83.2, 66.2, 63.1, 55.6, 44.1, 37.4;

HRMS (EI): m/z calcd for $C_{22}H_{23}O_2$ [(M)$^+$]: 346.156345; Found: 346.156730.

$[\alpha]_D^{25}$ = 60.47 (c = 0.13, CH_2Cl_2)

HPLC: Daicel Chiralcel OZ-3R, MeCN/H_2O = 50/50, 1.0 mL/min, 25 °C, 254 nm, t_R (major) = 12.0 min, t_R (minor) = 14.0 min;

e.r. = 95.5: 4.5

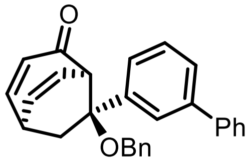

(1R,5R,8S)-8-([1,1'-biphenyl]-3-yl)-8-(benzyloxy)bicyclo[3.2.2]nona-3,6-dien-2-one (3l)
Appearance: colorless viscous oil.
^{1}H NMR (500 MHz, CD_2Cl_2) δ 7.64 (t, J = 1.9 Hz, 1H), 7.52-7.59 (m, 3H), 7.34-7.47 (m, 5H), 7.18-7.31 (m, 6H), 6.68 (ddd, J = 8.2, 7.1, 1.0 Hz, 1H), 6.23 (ddd, J = 8.5, 7.5, 1.1 Hz, 1H), 5.88 (ddd, J = 10.9, 2.0, 0.8 Hz, 1H), 4.37-4.40 (m, 2H), 4.14 (d, J = 11.0 Hz, 1H), 3.37-3.42 (m, 1H), 2.77 (dd, J = 14.0, 1.8 Hz, 1H), 2.44 (dd, J = 14.1, 5.1 Hz, 1H);
^{13}C NMR (125 MHz, CD_2Cl_2) δ 195.2, 152.7, 146.2, 141.8, 141.4, 139.8, 139.1, 130.7, 129.4, 129.2, 128.6, 127.9, 127.9, 127.7, 127.5, 126.5, 126.1, 126.0, 125.8, 83.4, 66.3, 63.3, 44.0, 37.4;
HRMS (ESI): m/z calcd for $C_{28}H_{24}O_2Na$ [$(M+Na)^+$]: 415.166849; Found: 415.167020.
$[\alpha]_D^{25}$ = -41.15 (c = 1.04, CH_2Cl_2)
HPLC: Daicel Chiralcel OJ-3R, MeCN/H_2O = 50/50, 1.0 mL/min, 25 °C, 230 nm, t_R (minor) = 35.3 min, t_R (minor) = 55.2 min;
e.r. = 95.0: 5.0

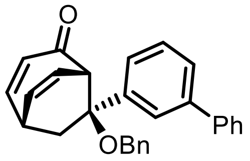

(1S, 5S, 8S)-8-([1,1'-biphenyl]-3-yl)-8-(benzyloxy)bicyclo[3.2.2]nona-3,6-dien-2-one (3l-2)
Appearance: colorless viscous oil.
^{1}H NMR (500 MHz, CD_2Cl_2) δ 7.73 (t, J = 1.9 Hz, 1H), 7.50-7.57 (m, 4H), 7.43-7.46 (ddd, J = 8.0, 7.0, 1.7 Hz, 13H), 7.23-7.37 (m, 6H), 7.17 (dd, J = 10.9, 8.8 Hz, 1H), 6.77 (ddd, J = 8.3, 7.0, 1.1 Hz, 1H), 6.28 (ddd, J = 8.3, 7.1, 1.1 Hz, 1H), 5.88 (dd, J = 10.9, 2.0 Hz, 1H), 4.19 (dd, J = 13.8, 11.7 Hz, 2H), 4.03 (d, J = 7.2 Hz, 1H), 3.52 (q, J = 7.0 Hz, 1H), 3.09 (dd, J = 14.1, 2.1 Hz, 1H), 2.47 (dd, J = 14.1, 5.0 Hz, 1H);
^{13}C NMR (125 MHz, CD_2Cl_2) δ 194.4, 152.6, 143.5, 141.4, 141.4, 139.3, 138.6, 130.7, 129.2, 129.2, 128.6, 127.8, 127.7, 127.6, 127.5, 127.4, 127.3, 127.1, 126.9, 83.3, 68.2, 65.8, 38.2, 36.4;
HRMS (ESI): m/z calcd for $C_{28}H_{24}O_2Na$ [$(M+Na)^+$]: 415.166849; Found: 415.167020.
$[\alpha]_D^{25}$ = 79.17 (c = 0.24, CH_2Cl_2)
HPLC: Daicel Chiralcel OJ-3R, MeCN/H_2O = 50/50, 1.0 mL/min, 25 °C, 230 nm, t_R (major) = 30.4 min, t_R (minor) = 33.3 min;
e.r. = 92.0: 8.0

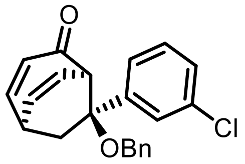

(1R, 5R, 8S)-8-(benzyloxy)-8-(3-chlorophenyl)bicyclo[3.2.2]nona-3,6-dien-2-one (3m)
Appearance: light yellow solid.
^{1}H NMR (500 MHz, CD_2Cl_2) δ 7.50 (m, 1H), 7.26 (m, 9H), 7.15 (dd, J = 10.9, 8.8 Hz, 1H), 6.67 (ddd, J = 8.3, 7.1, 1.0 Hz, 1H), 6.17 (ddd, J = 8.4, 7.4, 1.1 Hz, 1H), 5.86 (ddd, J = 10.9, 1.9, 0.8 Hz, 1H), 4.29 (m, 2H), 4.05 (d, J = 10.9 Hz, 2H), 3.38 (m, 1H), 2.70 (dd, J = 14.2, 1.8 Hz, 1H), 2.33 (dd, J = 14.2, 5.1 Hz, 1H);

^{13}C NMR (125 MHz, CD_2Cl_2) δ 194.7, 152.5, 147.8, 140.0, 138.6, 134.8, 130.6, 130.3, 128.6, 127.9, 127.8, 127.8, 127.2, 125.8, 125.3, 82.9, 66.2, 63.2, 43.7, 37.2;

HRMS (CI): m/z calcd for $C_{22}H_{19}O_2C_{l1}$ [M]$^+$: 350.106880; Found: 350.106808.

$[\alpha]_D^{25}$ = -61.77 (c = 0.40, CH_2Cl_2)

HPLC: Daicel Chiralcel OJ-3R, MeCN/H_2O = 50/50, 1.0 mL/min, 25 °C, 254 nm, t_R (minor) = 20.4 min, t_R (major) = 23.0 min;

e.r. = 93.0: 7.0

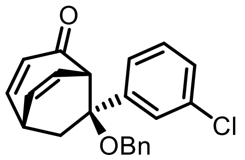

(1S, 5S, 8S)-8-(benzyloxy)-8-(3-chlorophenyl)bicyclo[3.2.2]nona-3,6-dien-2-one (3m-2)

Appearance: white solid.

^{1}H NMR (500 MHz, CD_2Cl_2) δ 7.50 (t, *J* = 2.0 Hz, 1H), 7.43 (dt, *J* = 7.7, 1.5 Hz, 1H), 7.29 (m, 7H), 7.15 (dd, *J* = 10.9, 8.8 Hz, 1H), 6.75 (ddd, *J* = 8.2, 7.0, 1.1 Hz, 1H), 6.25 (ddd, *J* = 8.2, 7.1, 1.1 Hz, 1H), 5.52 (ddd, *J* = 10.9, 2.1, 0.7 Hz, 1H), 4.13 (dd, *J* = 13.8, 11.6 Hz, 2H), 3.92 (d, *J* = 7.2Hz, 1H), 3.50 (m, 1H), 2.95 (dd, *J* = 14.2, 2.0 Hz, 1H), 2.42 (dd, *J* = 14.1, 4.9 Hz, 1H);

^{13}C NMR (125 MHz, CD_2Cl_2) δ 194.1, 152.6, 145.3, 138.9, 138.6, 134.7, 130.7, 130.1, 128.7, 128.6, 128.3, 127.8, 127.6, 126.9, 126.7, 82.9, 68.3, 65.9, 37.8, 36.2;

HRMS (CI): m/z calcd for $C_{22}H_{19}O_2C_{l1}$ [M]$^+$: 350.106820; Found: 350.106808.

$[\alpha]_D^{25}$ = 49.23 (c = 0.20, CH_2Cl_2)

HPLC: Daicel Chiralcel OJ-3R, MeCN/H_2O = 50/50, 1.0 mL/min, 25 °C, 254 nm, t_R (minor) = 15.9 min, t_R (major) = 27.5 min;

e.r. = 93.0: 7.0

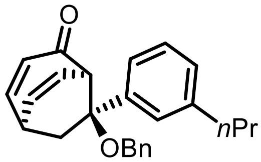

(1R, 5R, 8S)-8-(benzyloxy)-8-(3-propylphenyl)bicyclo[3.2.2]nona-3,6-dien-2-one (3n)

Appearance: yellow viscous oil.

^{1}H NMR (500 MHz, CD_2Cl_2) δ 7.16-7.30 (m, 9H), 7.10 (dt, *J* = 7.4, 1.5 Hz, 1H), 6.63 (ddd, *J* = 8.3, 7.2, 1.1 Hz, 1H), 6.18 (ddd, *J* = 8.4, 7.5, 1.1 Hz, 1H), 5.86 (ddd, *J* = 10.9, 1.9, 0.8 Hz, 1H), 4.35 (d, *J* = 7.7, 2H), 4.29 (d, *J* = 10.8 Hz, 1H), 4.02 (d, *J* = 10.8 Hz, 1H), 3.34-3.39 (m, 1H), 2.71 (dd, *J* = 14.1, 1.8 Hz, 1H), 2.60 (t, *J* = 7.7 Hz, 2H), 2.37 (dd, *J* = 14.1, 5.1 Hz, 1H), 1.63 (hept, *J* = 7.3 Hz, 2h), 0.93 (t, *J* = 7.3 Hz, 3H);

^{13}C NMR (125 MHz, CD_2Cl_2) δ 195.4, 152.7, 145.3, 143.5, 1.9.6, 139.1, 130.7, 128.6, 128.5, 128.1, 127.9, 127.6, 127.3, 126.1, 124.2, 83.2, 66.1, 63.1, 44.1, 38.5, 37.4, 25.1, 14.0;

HRMS (EI): m/z calcd for $C_{25}H_{26}O_2$ [(M)$^+$]: 358.192730; Found: 358.193180.

$[\alpha]_D^{25}$ = -59.41 (c = 0.52, CH_2Cl_2)

HPLC: Daicel Chiralcel OJ-3R, MeCN/H_2O = 50/50, 1.0 mL/min, 25 °C, 254 nm, t_R (major) = 26.2 min, t_R (minor) = 30.8 min;

e.r. = 95.5: 4.5

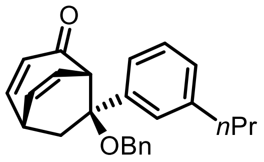

(1S, 5S, 8S)-8-(benzyloxy)-8-(3-propylphenyl)bicyclo[3.2.2]nona-3,6-dien-2-one (3n-2)

Appearance: colorless viscous oil.

^{1}H NMR (500 MHz, CD_2Cl_2) δ 7.23-7.32 (m, 8H), 7.09-7.13 (m, 2H), 6.74 (ddd, *J* = 8.3, 7.5, 1.2 Hz, 1H), 6.24 (ddd, *J* = 8.2, 7.1, 1.1 Hz, 1H), 5.47 (ddd, *J* = 10.9, 2.1, 0.7 Hz, 1H), 4.12 (dd, *J* = 14.8, 11.6 Hz, 2H), 3.93 (d, *J* = 7.2 Hz, 1H), 3.46-3.51 (m, 1H), 3.02 (dd, *J* = 14.1, 2.0 Hz, 1H), 2.59 (dd, *J* = 8.5, 6.7 Hz, 2H), 2.40 (dd, *J* = 14.1, 4.9 Hz, 1H), 1.61 (hept, *J* = 7.3 Hz, 2h), 0.91 (t, *J* = 7.3 Hz, 3H);

^{13}C NMR (125 MHz, CD_2Cl_2) δ 194.4, 152.3, 143.1, 142.6, 139.4, 138.5, 130.7, 128.9, 128.7, 128.6, 128.4, 128.3, 127.7, 127.2, 125.7, 83.2, 68.4, 65.6, 38.4, 37.9, 36.4, 25.0, 13.9;

HRMS (EI): m/z calcd for $C_{25}H_{26}O_2$ [$(M)^+$]: 358.192730; Found: 358.193040.

$[\alpha]_D^{25}$ = 72.11 (c = 0.18, CH_2Cl_2)

HPLC: Daicel Chiralcel OJ-3R, MeCN/H_2O = 50/50, 1.0 mL/min, 25 °C, 254 nm, t_R (minor) = 19.9 min, t_R (major) = 33.5 min;

e.r. = 94.0: 6.0

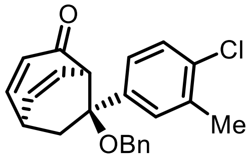

(1R, 5R, 8S)-8-(benzyloxy)-8-(4-chloro-3-methylphenyl)bicyclo[3.2.2]nona-3,6-dien-2-one (3o)

Appearance: white solid.

^{1}H NMR (500 MHz, CD_2Cl_2) δ 7.16-7.34 (m, 8H), 6.65 (ddd, *J* = 8.2, 7.1, 1.0 Hz, 1H), 6.16 (ddd, *J* = 8.5, 7.5, 1.1 Hz, 1H), 5.85 (dd, *J* = 10.1, 1.6 Hz, 1H), 4.26-4.31 (m, 2H), 4.03 (d, *J* = 10.9 Hz, 1H), 3.35-3.40 (m, 1H), 2.69 (dd, *J* = 14.2, 1.8 Hz, 1H), 2.38 (s, 3H), 2.33 (dd, *J* =14.1, 5.1 Hz, 1H);

^{13}C NMR (125 MHz, CD_2Cl_2) δ 195.0, 152.6, 144.2, 139.9, 138.9, 1366.5, 130.6, 129.8, 129.3, 128.6, 128.0, 127.7, 125.9, 125.9, 82.8, 66.2, 63.3, 43.7, 37.3, 20.4;

HRMS (CI): m/z calcd for $C_{23}H_{21}O_2Cl_1$ [M^+]: 364.122458; Found: 364.122640.

$[\alpha]_D^{25}$ = -59.00 (c = 0.60, CH_2Cl_2)

HPLC: Daicel Chiralcel OJ-3R, MeCN/H_2O = 50/50, 1.0 mL/min, 25 °C, 230 nm, t_R (minor) = 30.3 min, t_R (major) = 34.1 min;

e.r. = 95.0: 5.0

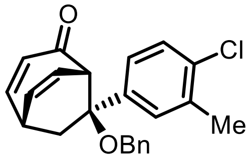

(1S, 5S, 8R)-8-(benzyloxy)-8-(4-chloro-3-methylphenyl)bicyclo[3.2.2]nona-3,6-dien-2-one (3o-2)
Appearance: white solid.
^{1}H NMR (500 MHz, CD_2Cl_2) δ 7.38 (d, J = 2.6 Hz, 1H), 7.22-7.32 (m, 7H), 7.13 (dd, J = 10.9, 8.8 Hz, 1H), 6.74 (ddd, J = 8.3, 7.1, 1.0 Hz, 1H), 6.24 (ddd, J = 8.2, 7.2, 1.1 Hz, 1H), 5.49 (dd, J = 10.7, 2.0 Hz, 1H), 4.10 (d, J = 1.5 Hz, 2H), 3.92 (d, J = 7.2 Hz, 1H), 3.46-3.50 (m, 1H), 2.96 (dd, J = 14.1, 2.1 Hz, 1H), 2.37-2.41 (m, 4H);
^{13}C NMR (125 MHz, CD_2Cl_2) δ 194.3, 152.5, 141.6, 139.1, 138.5, 136.3, 134.1, 131.1, 130.7, 129.1, 128.6, 127.7, 127.7, 127.3, 127.0, 82.8, 68.2, 65.7, 37.8, 36.3, 20.5;
HRMS (CI): m/z calcd for $C_{23}H_{21}O_2Cl_1$ [M$^+$]: 364.122458; Found: 364.122820.
$[\alpha]_D^{25}$ = 82.22 (c = 0.18, CH_2Cl_2)
HPLC: Daicel Chiralcel OJ-3R, MeCN/H_2O = 50/50, 1.0 mL/min, 25 °C, 230 nm, t_R (minor) = 21.1 min, t_R (major) = 38.6 min;
e.r. = 6.0: 94.0

13. Procedure for the synthesis of 3p:

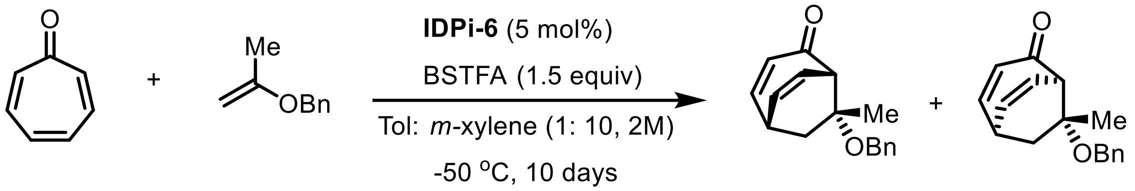

1 **2p** **3p** **3p-2**

To a solution of BSTFA (0.2 mmol) and IDPi-**6** (0.005 mmol) in tol/m-xylene (1: 10, v/v, 0.1 mL) was added tropone (0.1 mmol) and **2p** (0.20 mmol) at indicated temperature. The resultant mixture was stirred for indicated time and quenched with triethylamine (0.1 mL). After removal of the solvent under reduced pressure, the crude residue was subjected to flash column chromatography on silica gel (hexane/ethyl acetate 20: 1 to 10: 1) to afford product **3-1** and **3-2.**

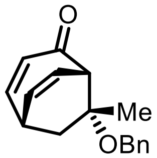

(1S, 5S, 8R)-8-(benzyloxy)-8-methylbicyclo[3.2.2]nona-3,6-dien-2-one (3p)
Appearance: white solid.
^{1}H NMR (500 MHz, CD_2Cl_2) δ 7.21-7.31 (m, 5H), 7.09 (dd, J = 11.0, 8.8 Hz, 1H), 6.58 (ddd, J = 8.2, 7.0, 1.0 Hz, 1H), 6.11 (t, J = 8.0 Hz, 1H), 5.74 (dd, J = 11.0, 2.8 Hz, 1H), 4.49 (s, 2H), 3.78 (dt, J = 7.7, 1.5 Hz, 1H), 3.26-3.30 (m, 1H), 2.35 (dd, J = 13.7, 2.1 Hz, 1H), 1.81 (dd, J = 13.7, 5.0 Hz, 1H), 1.44 (s, 3H);
^{13}C NMR (125 MHz, CD_2Cl_2) δ 195.8, 152.2, 139.5, 139.0, 130.5, 128.5, 127.8, 127.6, 126.6, 79.5, 65.4, 64.8, 42.1, 37.3, 27.7;
HRMS (EI): m/z calcd for $C_{17}H_{18}O_2$ [M]$^+$: 254.130130; Found: 254.130390.
$[\alpha]_D^{25}$ = 11.82 (c = 0.40, CH_2Cl_2)
HPLC: Daicel Chiralcel IG-3R, MeCN/H_2O = 50/50, 0.5 mL/min, 25 °C, 254 nm, t_R (major) = 19.1 min, t_R (min) = 26.4 min;
e.r. = 95.0: 5.0 e.r.

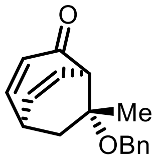

(1S, 5S, 8R)-8-(benzyloxy)-8-methylbicyclo[3.2.2]nona-3,6-dien-2-one (3p-2)

Appearance: white solid.

^{1}H NMR (500 MHz, CD_2Cl_2) δ 7.23-7.33 (m, 5H), 7.12 (dd, J = 10.9, 8.8 Hz, 1H), 6.62 (t, J = 7.8 Hz, 1H), 6.14 (ddd, J = 8.4, 7.1, 1.0 Hz, 1H), 5.67 (dd, J = 10.9, 2.2 Hz, 1H), 4.38 (dd, J = 14.8, 11.2 Hz, 2H), 3.74 (d, J = 6.2 Hz, 1H), 3.30 (q, J = 7.2 Hz, 1H), 2.07-2.16 (m, 2H), 1.45 (s, 3H);

^{13}C NMR (125 MHz, CD_2Cl_2) δ 195.8, 154.1, 139.6, 137.2, 129.4, 128.6, 127.8, 127.6, 127.1, 78.6, 64.9, 64.2, 41.9, 36.0, 27.2;

HRMS (EI): m/z calcd for $C_{17}H_{18}O_2$ $[M]^+$: 254.130130; Found: 254.130380.

$[\alpha]_D^{25}$ = -16.00 (c = 0.10, CH_2Cl_2)

HPLC: Daicel Chiralcel IG-3R, MeCN/H_2O = 50/50, 0.5 mL/min, 25 °C, 254 nm, t_R (major) = 28.8 min, t_R (min) = 31.8 min;

e.r. = 93.0: 7.0 e.r.

14. X-ray Crystal Structure Analysis

Single crystal structure analysis of **14909**

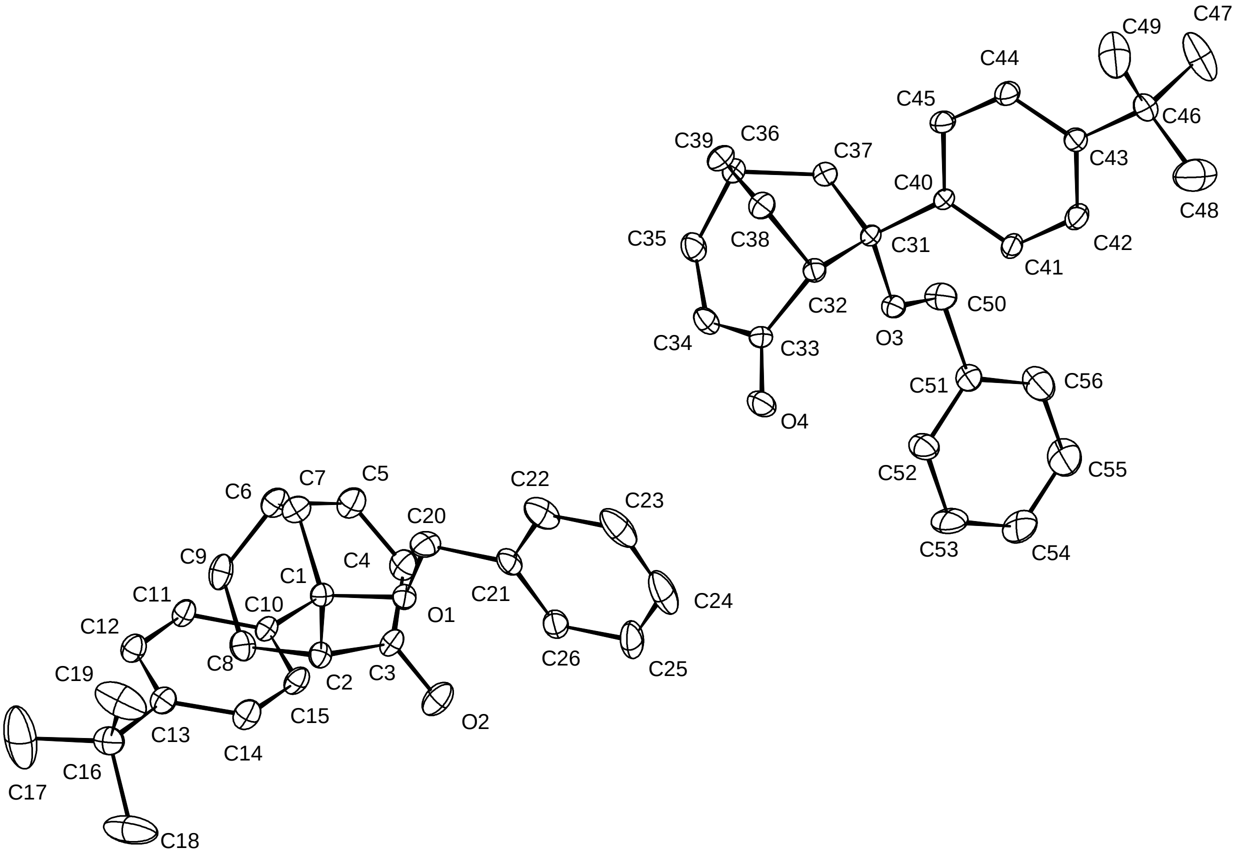

Figure S8. The asymmetric unit of **14909**. H atoms have been removed for clarity.

X-ray Crystal Structure Analysis of **14909**:

$C_{26}H_{28}O_2$, M_r = 372.511 g mol^{-1}, colourless prism, crystal size 0.28 x 0.118 x 0.05 mm^3, monoclinic, space group $P2_1$ [4], a = 11.8321(6) Å, b = 6.5546(3) Å, c = 26.7099(13)Å, β= 99.063(2)°, V = 372.511 Å^3,T = 100(2) K, Z = 4, D_{calc} = 1.210 g·cm^3, λ = 1.54178 Å, $\mu(Cu\text{-}K_\alpha)$ = 0.580 mm^{-1}, Gaussian correction ($T_{\min}$ = 0.87933, $T_{\max}$ = 0.97315), Bruker-AXS Kappa Mach3 with APEX-II detector and FR591 rotating anode X-ray source, 1.67 < θ < 36.32°, 74810 measured reflections, 7702 independent reflections, 7196 reflections with $I > 2\sigma(I)$, R_{int} = 0.0358. The structure was solved by *SHELXT* and refined by full-matrix least-squares (*SHELXL*). The final structure refinement was performed by olex2.refine 1.5 (L-M) together with NoSpherA2 (atomic form factors) against F^2 to R_1 = 0.0370 [$I > 2\sigma(I)$], wR_2 = 0.0867 [all data] with 673 parameters, 1 restraints and an absolute structure parameter Flack x = 0.04(4).

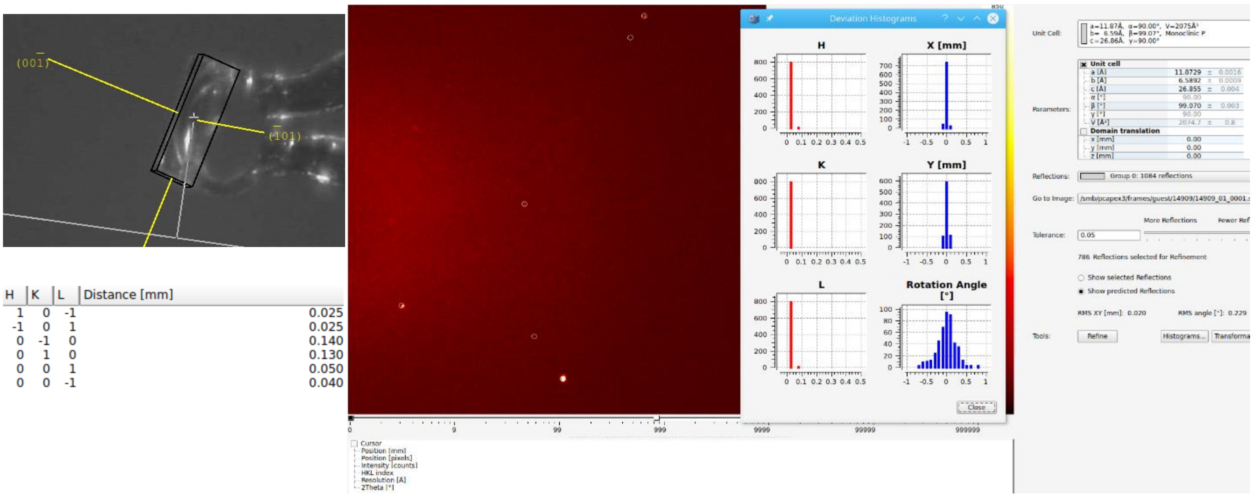

Figure S9. Crystal faces and unit cell determination/refinement of 14909.

INTENSITY STATISTICS FOR DATASET

Resolution	#Data	#Theory	%Complete	Redundancy	Mean I	Mean I/s	Rmerge	Rsigma
Inf - 3.28	116	116	100.0	9.71	162.01	91.62	0.0282	0.0100
3.28 - 2.21	269	269	100.0	9.25	51.86	77.34	0.0290	0.0110
2.21 - 1.77	389	389	100.0	10.66	41.94	72.04	0.0277	0.0110
1.77 - 1.55	385	385	100.0	9.94	18.06	55.84	0.0315	0.0139
1.55 - 1.41	381	381	100.0	8.90	13.60	44.35	0.0368	0.0172
1.41 - 1.30	403	403	100.0	7.64	12.20	37.31	0.0392	0.0209
1.30 - 1.22	426	426	100.0	11.56	15.11	53.83	0.0368	0.0171
1.22 - 1.16	393	393	100.0	15.05	14.54	66.99	0.0360	0.0138
1.16 - 1.11	364	364	100.0	14.54	12.27	60.92	0.0358	0.0143
1.11 - 1.07	372	372	100.0	13.90	11.90	59.02	0.0374	0.0148
1.07 - 1.03	440	440	100.0	13.40	8.98	52.18	0.0401	0.0169
1.03 - 1.00	370	376	98.4	12.19	7.73	48.36	0.0485	0.0186
1.00 - 0.97	364	386	94.3	10.81	6.84	43.50	0.0504	0.0192
0.97 - 0.94	425	454	93.6	10.34	5.17	39.49	0.0578	0.0222
0.94 - 0.92	326	343	95.0	10.20	4.75	36.86	0.0590	0.0232
0.92 - 0.90	363	383	94.8	9.89	3.64	32.06	0.0664	0.0270
0.90 - 0.88	392	417	94.0	9.48	3.74	32.85	0.0660	0.0264
0.88 - 0.86	436	467	93.4	5.63	2.97	19.42	0.0949	0.0682
0.86 - 0.84	421	458	91.9	2.09	1.86	5.12	0.1433	0.1800
0.84 - 0.82	493	543	90.8	1.92	2.02	5.70	0.1215	0.1546
0.82 - 0.81	182	290	62.8	0.98	1.52	3.78	0.1360	0.2033
0.91 - 0.81	2118	2378	89.1	4.56	2.57	15.45	0.0810	0.0910
Inf - 0.81	7710	8055	95.7	9.29	13.79	42.85	0.0358	0.0178

Complete .cif-data of the compound are available under the CCDC number **CCDC-2423660**.

The final structure refinement was carried out with using aspherical scattering factors with NoSpherA2.[3] DFT-calculated with ORCA using a B3LYP functional and def2-TZVPP basis set, whereby the H atom positions were refined using anisotropic atomic displacement parameters.

NoSpherA2 implementation of HAR makes use of tailor-made aspherical atomic form factors calculated on-the-fly from a Hirshfeld-partitioned electron density (ED) - not from spherical-atom form factors. The ED is calculated from a gaussian basis set single determinant SCF wave function - either Hartree-Fock or DFT using selected functional - for a fragment of the crystal. This fragment can be embedded in an electrostatic crystal field by employing cluster charges or modelled using implicit solvation models, depending on the software used. The following options were used:

SOFTWARE:	ORCA 5.0
PARTITIONING:	NoSpherA2
INT ACCURACY:	Normal
METHOD:	B3LYP
BASIS SET:	def2-TZVPP
CHARGE:	0
MULTIPLICITY:	1
DATE:	2025-02-12_13-18-53

Table S1. Crystal data and structure refinement of 14909.

Identification code	14909	
Empirical formula	$C_{26} H_{28} O_2$	
Color	colourless	
Formula weight	372.511 g·mol^{-1}	
Temperature	100(2) K	
Wavelength	1.54178 Å	
Crystal system	Monoclinic	
Space group	$P2_1$, (no. 4)	
Unit cell dimensions	a = 11.8321(6) Å	α= 90°.
	b = 6.5546(3) Å	β= 99.063(2)°.
	c = 26.7099(13) Å	γ = 90°.
Volume	2045.62(17) Å^3	
Z	4	
Density (calculated)	1.210 Mg·m^{-3}	
Absorption coefficient	0.580 mm^{-1}	
F(000)	802.416 e	
Crystal size	0.28 x 0.118 x 0.05 mm^3	
θ range for data collection	1.67 to 72.12°.	
Index ranges	-14 ≤ h ≤ 14, -8≤ k ≤ 8, -32 ≤ l ≤ 30	
Reflections collected	74810	
Independent reflections	7702 [R_{int} = 0.0358]	
Reflections with I>2σ(I)	7196	
Completeness to θ = 67.6786°	99.56 %	
Absorption correction	Gaussian	
Max. and min. transmission	0.97315 and 0.87933	
Refinement method	Full-matrix least-squares on F^2	
Data / restraints / parameters	7702 / 1 / 673	
Goodness-of-fit on F^2	1.0211	
Final R indices [I>2σ(I)]	R_1 = 0.0370	wR^2 = 0.0780
R indices (all data)	R_1 = 0.0424	wR^2 = 0.0867
Absolute structure parameter	0.04(4)	
Largest diff. peak and hole	0.2457 and -0.2241 e·Å^{-3}	

Table S2. Bond lengths [Å] and angles [°] of 14909.

O(1)-C(1)	1.434(2)	O(1)-C(20)	1.419(3)
O(2)-C(3)	1.225(2)	C(1)-C(2)	1.561(3)
C(1)-C(7)	1.547(3)	C(1)-C(10)	1.531(3)
C(2)-C(3)	1.533(3)	C(2)-C(8)	1.507(3)
C(2)-H(2)	1.14(2)	C(3)-C(4)	1.471(3)
C(4)-C(5)	1.350(3)	C(4)-H(4)	1.09(2)
C(5)-C(6)	1.503(3)	C(5)-H(5)	1.14(3)
C(6)-C(7)	1.545(3)	C(6)-C(9)	1.511(3)
C(6)-H(6)	1.09(2)	C(7)-H(7a)	1.07(2)
C(7)-H(7b)	1.13(2)	C(8)-C(9)	1.331(3)
C(8)-H(8)	1.10(2)	C(9)-H(9)	1.04(3)
C(10)-C(11)	1.397(3)	C(10)-C(15)	1.390(3)
C(11)-C(12)	1.395(3)	C(11)-H(11)	1.09(2)
C(12)-C(13)	1.394(3)	C(12)-H(12)	1.09(2)
C(13)-C(14)	1.406(3)	C(13)-C(16)	1.529(3)
C(14)-C(15)	1.389(3)	C(14)-H(14)	1.08(2)
C(15)-H(15)	1.14(2)	C(16)-C(17)	1.530(4)
C(16)-C(18)	1.526(3)	C(16)-C(19)	1.508(3)
C(17)-H(17a)	1.13(4)	C(17)-H(17b)	1.07(4)
C(17)-H(17c)	1.15(4)	C(18)-H(18a)	1.10(4)
C(18)-H(18b)	1.23(4)	C(18)-H(18c)	1.06(4)
C(19)-H(19a)	1.11(4)	C(19)-H(19b)	1.11(4)
C(19)-H(19c)	1.12(3)	C(20)-C(21)	1.502(3)
C(20)-H(20a)	1.10(3)	C(20)-H(20b)	1.15(2)
C(21)-C(22)	1.401(3)	C(21)-C(26)	1.390(3)
C(22)-C(23)	1.389(3)	C(22)-H(22)	1.13(3)
C(23)-C(24)	1.398(4)	C(23)-H(23)	1.13(3)
C(24)-C(25)	1.393(4)	C(24)-H(24)	1.12(3)
C(25)-C(26)	1.388(3)	C(25)-H(25)	1.07(3)
C(26)-H(26)	1.12(2)	O(3)-C(31)	1.436(2)
O(3)-C(50)	1.421(2)	O(4)-C(33)	1.228(2)
C(31)-C(32)	1.567(3)	C(31)-C(37)	1.546(3)
C(31)-C(40)	1.534(3)	C(32)-C(33)	1.524(3)
C(32)-C(38)	1.508(3)	C(32)-H(32)	1.10(2)
C(33)-C(34)	1.471(3)	C(34)-C(35)	1.346(3)
C(34)-H(34)	1.08(2)	C(35)-C(36)	1.507(3)

C(35)-H(35)	1.09(2)	C(36)-C(37)	1.541(3)
C(36)-C(39)	1.509(3)	C(36)-H(36)	1.11(2)
C(37)-H(37a)	1.09(2)	C(37)-H(37b)	1.08(2)
C(38)-C(39)	1.339(3)	C(38)-H(38)	1.10(2)
C(39)-H(39)	1.10(3)	C(40)-C(41)	1.387(3)
C(40)-C(45)	1.393(3)	C(41)-C(42)	1.405(3)
C(41)-H(41)	1.14(2)	C(42)-C(43)	1.398(3)
C(42)-H(42)	1.12(3)	C(43)-C(44)	1.394(3)
C(43)-C(46)	1.528(3)	C(44)-C(45)	1.401(3)
C(44)-H(44)	1.08(2)	C(45)-H(45)	1.10(2)
C(46)-C(47)	1.518(3)	C(46)-C(48)	1.524(3)
C(46)-C(49)	1.520(3)	C(47)-H(47a)	1.10(3)
C(47)-H(47b)	1.08(4)	C(47)-H(47c)	1.08(3)
C(48)-H(48a)	1.10(4)	C(48)-H(48b)	1.05(4)
C(48)-H(48c)	1.12(4)	C(49)-H(49a)	1.16(4)
C(49)-H(49b)	1.01(3)	C(49)-H(49c)	1.12(3)
C(50)-C(51)	1.506(3)	C(50)-H(50a)	1.10(2)
C(50)-H(50b)	1.17(3)	C(51)-C(52)	1.390(3)
C(51)-C(56)	1.386(3)	C(52)-C(53)	1.395(3)
C(52)-H(52)	1.11(3)	C(53)-C(54)	1.389(3)
C(53)-H(53)	1.10(3)	C(54)-C(55)	1.397(4)
C(54)-H(54)	1.08(3)	C(55)-C(56)	1.392(3)
C(55)-H(55)	1.15(3)	C(56)-H(56)	1.05(3)

C(20)-O(1)-C(1)	116.28(15)	C(2)-C(1)-O(1)	103.38(15)
C(7)-C(1)-O(1)	111.70(16)	C(7)-C(1)-C(2)	111.06(16)
C(10)-C(1)-O(1)	110.63(16)	C(10)-C(1)-C(2)	107.38(16)
C(10)-C(1)-C(7)	112.26(17)	C(3)-C(2)-C(1)	112.06(16)
C(8)-C(2)-C(1)	110.08(16)	C(8)-C(2)-C(3)	109.32(16)
H(2)-C(2)-C(1)	108.4(11)	H(2)-C(2)-C(3)	106.0(11)
H(2)-C(2)-C(8)	111.0(11)	C(2)-C(3)-O(2)	119.37(19)
C(4)-C(3)-O(2)	120.16(19)	C(4)-C(3)-C(2)	120.43(17)
C(5)-C(4)-C(3)	124.25(19)	H(4)-C(4)-C(3)	115.4(13)
H(4)-C(4)-C(5)	120.2(13)	C(6)-C(5)-C(4)	124.5(2)
H(5)-C(5)-C(4)	118.4(13)	H(5)-C(5)-C(6)	117.1(13)
C(7)-C(6)-C(5)	109.65(18)	C(9)-C(6)-C(5)	111.03(19)
C(9)-C(6)-C(7)	109.36(17)	H(6)-C(6)-C(5)	107.4(12)
H(6)-C(6)-C(7)	108.0(13)	H(6)-C(6)-C(9)	111.4(13)

C(6)-C(7)-C(1)	114.19(18)	H(7a)-C(7)-C(1)	109.7(12)
H(7a)-C(7)-C(6)	109.8(12)	H(7b)-C(7)-C(1)	108.3(13)
H(7b)-C(7)-C(6)	108.3(12)	H(7b)-C(7)-H(7a)	106.2(17)
C(9)-C(8)-C(2)	117.8(2)	H(8)-C(8)-C(2)	117.9(13)
H(8)-C(8)-C(9)	124.2(13)	C(8)-C(9)-C(6)	117.9(2)
H(9)-C(9)-C(6)	121.5(13)	H(9)-C(9)-C(8)	120.6(13)
C(11)-C(10)-C(1)	122.09(18)	C(15)-C(10)-C(1)	120.53(17)
C(15)-C(10)-C(11)	117.29(18)	C(12)-C(11)-C(10)	121.31(19)
H(11)-C(11)-C(10)	117.9(12)	H(11)-C(11)-C(12)	120.8(12)
C(13)-C(12)-C(11)	121.7(2)	H(12)-C(12)-C(11)	118.3(12)
H(12)-C(12)-C(13)	119.9(12)	C(14)-C(13)-C(12)	116.52(19)
C(16)-C(13)-C(12)	122.54(19)	C(16)-C(13)-C(14)	120.95(19)
C(15)-C(14)-C(13)	121.7(2)	H(14)-C(14)-C(13)	119.6(12)
H(14)-C(14)-C(15)	118.7(12)	C(14)-C(15)-C(10)	121.41(19)
H(15)-C(15)-C(10)	119.3(12)	H(15)-C(15)-C(14)	119.2(12)
C(17)-C(16)-C(13)	111.6(2)	C(18)-C(16)-C(13)	109.43(19)
C(18)-C(16)-C(17)	106.3(3)	C(19)-C(16)-C(13)	109.44(19)
C(19)-C(16)-C(17)	110.4(3)	C(19)-C(16)-C(18)	109.5(2)
H(17a)-C(17)-C(16)	94(2)	H(17b)-C(17)-C(16)	120(2)
H(17b)-C(17)-H(17a)	117(3)	H(17c)-C(17)-C(16)	109(2)
H(17c)-C(17)-H(17a)	97(3)	H(17c)-C(17)-H(17b)	117(3)
H(18a)-C(18)-C(16)	110(2)	H(18b)-C(18)-C(16)	112.5(19)
H(18b)-C(18)-H(18a)	107(3)	H(18c)-C(18)-C(16)	109(2)
H(18c)-C(18)-H(18a)	106(3)	H(18c)-C(18)-H(18b)	112(3)
H(19a)-C(19)-C(16)	107.7(18)	H(19b)-C(19)-C(16)	109.4(19)
H(19b)-C(19)-H(19a)	116(3)	H(19c)-C(19)-C(16)	112.0(17)
H(19c)-C(19)-H(19a)	103(3)	H(19c)-C(19)-H(19b)	109(3)
C(21)-C(20)-O(1)	110.22(17)	H(20a)-C(20)-O(1)	111.1(12)
H(20a)-C(20)-C(21)	109.5(13)	H(20b)-C(20)-O(1)	111.4(12)
H(20b)-C(20)-C(21)	108.5(13)	H(20b)-C(20)-H(20a)	106.0(18)
C(22)-C(21)-C(20)	118.3(2)	C(26)-C(21)-C(20)	122.76(19)
C(26)-C(21)-C(22)	118.9(2)	C(23)-C(22)-C(21)	120.9(2)
H(22)-C(22)-C(21)	117.4(14)	H(22)-C(22)-C(23)	121.7(14)
C(24)-C(23)-C(22)	119.7(2)	H(23)-C(23)-C(22)	120.8(16)
H(23)-C(23)-C(24)	119.4(16)	C(25)-C(24)-C(23)	119.4(2)
H(24)-C(24)-C(23)	119.5(15)	H(24)-C(24)-C(25)	121.1(15)
C(26)-C(25)-C(24)	120.6(3)	H(25)-C(25)-C(24)	117.0(14)
H(25)-C(25)-C(26)	122.4(14)	C(25)-C(26)-C(21)	120.4(2)

H(26)-C(26)-C(21)	118.4(13)	H(26)-C(26)-C(25)	121.1(13)
C(50)-O(3)-C(31)	117.74(15)	C(32)-C(31)-O(3)	102.38(15)
C(37)-C(31)-O(3)	112.46(16)	C(37)-C(31)-C(32)	111.01(16)
C(40)-C(31)-O(3)	110.39(15)	C(40)-C(31)-C(32)	106.65(16)
C(40)-C(31)-C(37)	113.26(16)	C(33)-C(32)-C(31)	111.18(16)
C(38)-C(32)-C(31)	111.33(16)	C(38)-C(32)-C(33)	108.71(16)
H(32)-C(32)-C(31)	108.3(11)	H(32)-C(32)-C(33)	104.5(11)
H(32)-C(32)-C(38)	112.7(11)	C(32)-C(33)-O(4)	119.79(19)
C(34)-C(33)-O(4)	120.15(19)	C(34)-C(33)-C(32)	120.03(17)
C(35)-C(34)-C(33)	124.1(2)	H(34)-C(34)-C(33)	114.2(13)
H(34)-C(34)-C(35)	121.3(13)	C(36)-C(35)-C(34)	125.0(2)
H(35)-C(35)-C(34)	118.8(13)	H(35)-C(35)-C(36)	116.3(13)
C(37)-C(36)-C(35)	110.47(17)	C(39)-C(36)-C(35)	111.12(17)
C(39)-C(36)-C(37)	108.65(18)	H(36)-C(36)-C(35)	109.5(12)
H(36)-C(36)-C(37)	106.5(12)	H(36)-C(36)-C(39)	110.5(12)
C(36)-C(37)-C(31)	113.80(17)	H(37a)-C(37)-C(31)	109.1(12)
H(37a)-C(37)-C(36)	108.6(12)	H(37b)-C(37)-C(31)	108.6(13)
H(37b)-C(37)-C(36)	110.2(12)	H(37b)-C(37)-H(37a)	106.2(16)
C(39)-C(38)-C(32)	117.76(18)	H(38)-C(38)-C(32)	119.2(13)
H(38)-C(38)-C(39)	122.7(13)	C(38)-C(39)-C(36)	117.50(19)
H(39)-C(39)-C(36)	120.2(13)	H(39)-C(39)-C(38)	122.3(13)
C(41)-C(40)-C(31)	120.18(18)	C(45)-C(40)-C(31)	121.94(17)
C(45)-C(40)-C(41)	117.60(19)	C(42)-C(41)-C(40)	121.3(2)
H(41)-C(41)-C(40)	119.8(12)	H(41)-C(41)-C(42)	118.9(12)
C(43)-C(42)-C(41)	121.66(19)	H(42)-C(42)-C(41)	116.6(13)
H(42)-C(42)-C(43)	121.7(13)	C(44)-C(43)-C(42)	116.30(19)
C(46)-C(43)-C(42)	122.21(18)	C(46)-C(43)-C(44)	121.49(19)
C(45)-C(44)-C(43)	122.3(2)	H(44)-C(44)-C(43)	120.2(13)
H(44)-C(44)-C(45)	117.5(13)	C(44)-C(45)-C(40)	120.81(19)
H(45)-C(45)-C(40)	118.5(12)	H(45)-C(45)-C(44)	120.6(12)
C(47)-C(46)-C(43)	109.04(18)	C(48)-C(46)-C(43)	111.81(19)
C(48)-C(46)-C(47)	109.1(2)	C(49)-C(46)-C(43)	110.73(18)
C(49)-C(46)-C(47)	108.7(2)	C(49)-C(46)-C(48)	107.4(2)
H(47a)-C(47)-C(46)	109.6(17)	H(47b)-C(47)-C(46)	109.9(18)
H(47b)-C(47)-H(47a)	108(3)	H(47c)-C(47)-C(46)	111.1(17)
H(47c)-C(47)-H(47a)	113(2)	H(47c)-C(47)-H(47b)	105(3)
H(48a)-C(48)-C(46)	116(2)	H(48b)-C(48)-C(46)	109(2)
H(48b)-C(48)-H(48a)	105(3)	H(48c)-C(48)-C(46)	108.5(19)

H(48c)-C(48)-H(48a)	113(3)	H(48c)-C(48)-H(48b)	105(3)
H(49a)-C(49)-C(46)	113.6(17)	H(49b)-C(49)-C(46)	111.9(18)
H(49b)-C(49)-H(49a)	105(2)	H(49c)-C(49)-C(46)	110.4(16)
H(49c)-C(49)-H(49a)	106(2)	H(49c)-C(49)-H(49b)	110(2)
C(51)-C(50)-O(3)	109.05(17)	H(50a)-C(50)-O(3)	112.5(12)
H(50a)-C(50)-C(51)	107.6(13)	H(50b)-C(50)-O(3)	108.1(12)
H(50b)-C(50)-C(51)	109.0(12)	H(50b)-C(50)-H(50a)	110.5(18)
C(52)-C(51)-C(50)	121.05(19)	C(56)-C(51)-C(50)	119.6(2)
C(56)-C(51)-C(52)	119.3(2)	C(53)-C(52)-C(51)	120.2(2)
H(52)-C(52)-C(51)	119.4(14)	H(52)-C(52)-C(53)	120.4(13)
C(54)-C(53)-C(52)	120.3(2)	H(53)-C(53)-C(52)	120.7(14)
H(53)-C(53)-C(54)	119.0(14)	C(55)-C(54)-C(53)	119.6(2)
H(54)-C(54)-C(53)	118.7(14)	H(54)-C(54)-C(55)	121.6(14)
C(56)-C(55)-C(54)	119.6(2)	H(55)-C(55)-C(54)	121.1(16)
H(55)-C(55)-C(56)	119.2(16)	C(55)-C(56)-C(51)	121.0(2)
H(56)-C(56)-C(51)	118.5(15)	H(56)-C(56)-C(55)	120.5(15)

Single crystal structure analysis of **15851**

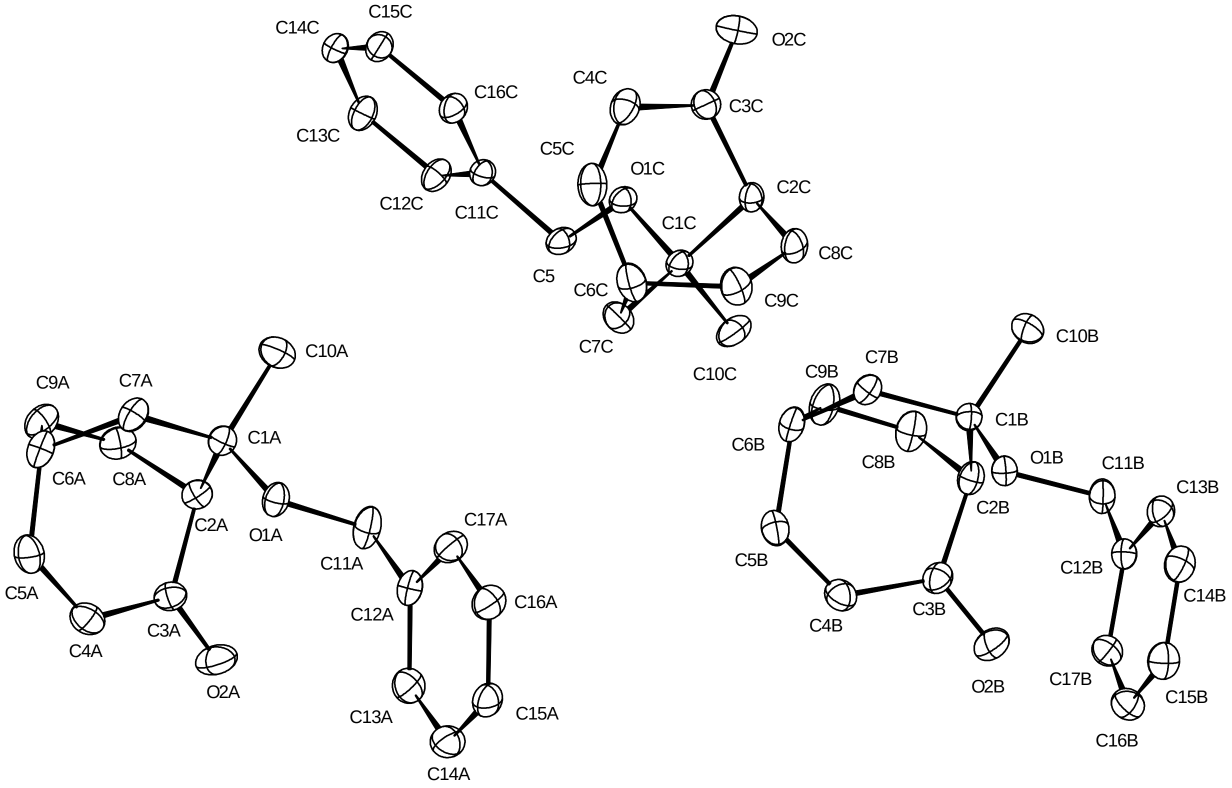

Figure S9. The asymmetric unit of **15851**. H atoms have been removed for clarity.

X-ray Crystal Structure Analysis of **15851**:

C_{17} H_{18} O_2, M_r = 254.331 g mol^{-1}, colourless prism, crystal size 0.409 x 0.316 x 0.190 mm^3, monoclinic, space group $P2_1$ [4], a = 12.2689(14) Å, b = 11.416(1) Å, c = 14.4948(15) Å, β= 92.442(3)°, V = 2028.3(4) Å^3,T = 100(2) K, Z = 6, D_{calc} = 1.249 g·cm^3, λ = 0.71073 Å, μ(Mo-K_α) = 0.580 mm^{-1}, Gaussian correction ($T_{\min}$ = 0.9668, $T_{\max}$ = 0.9943), Bruker-AXS D8 Venture with Photon III detector and IµS Diamond microfocus Mo-anode X-ray source, 2.13 < θ < 33.79°, 405123 measured reflections, 16209 independent reflections, 15786 reflections with $I > 2\sigma(I)$, R_{int} = 0.0541. The structure was solved by *SHELXT* and refined by full-matrix least-squares (*SHELXL*). The final structure refinement was performed by olex2.refine 1.5 (L-M) together with NoSpherA2 (atomic form factors) against F^2 to R_1 = 0.0171 [$I > 2\sigma(I)$], wR_2 = 0.0355 [all data] with 1000 parameters, 1 restraints and an absolute structure parameter Flack x = 0.08(7).

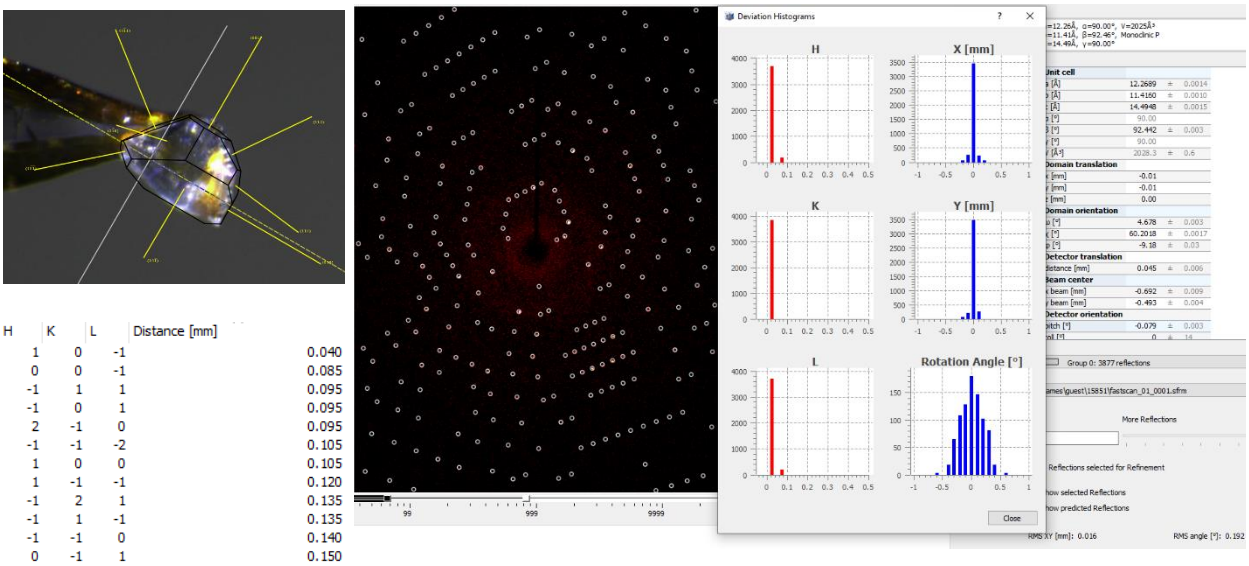

H	K	L	Distance [mm]
1	0	-1	0.040
0	0	-1	0.085
-1	1	1	0.095
-1	0	1	0.095
2	-1	0	0.095
-1	-1	-2	0.105
1	0	0	0.105
1	-1	-1	0.120
-1	2	1	0.135
-1	1	-1	0.135
-1	-1	0	0.140
0	-1	1	0.150
1	-1	1	0.165
0	1	0	0.170
1	1	0	0.170
0	0	1	0.170
1	-2	-1	0.175
1	1	2	0.195

Figure S10. Crystal faces and unit cell determination/refinement of 15851.

INTENSITY STATISTICS FOR DATASET

Resolution	#Data	#Theory	%Complete	Redundancy	Mean I	Mean I/s	Rmerge	Rsigma
Inf - 2.58	246	255	96.5	15.20	83.92	91.81	0.0525	0.0310
2.58 - 1.73	565	568	99.5	25.52	31.25	126.65	0.0271	0.0156
1.73 - 1.37	843	843	100.0	30.32	12.58	119.08	0.0296	0.0072
1.37 - 1.20	796	796	100.0	29.69	11.49	110.91	0.0318	0.0074
1.20 - 1.09	827	827	100.0	29.21	10.03	102.70	0.0354	0.0079
1.09 - 1.01	855	855	100.0	27.68	7.16	83.41	0.0434	0.0094
1.01 - 0.95	823	823	100.0	27.71	4.59	69.88	0.0557	0.0117
0.95 - 0.90	869	869	100.0	27.31	3.28	56.45	0.0686	0.0142
0.90 - 0.86	865	865	100.0	26.94	2.61	47.23	0.0793	0.0167
0.86 - 0.83	740	740	100.0	26.48	2.36	42.70	0.0867	0.0185
0.83 - 0.80	835	835	100.0	25.91	2.24	39.99	0.0940	0.0202
0.80 - 0.78	694	694	100.0	25.62	2.34	40.14	0.0927	0.0203
0.78 - 0.75	1105	1105	100.0	24.97	2.32	37.51	0.0970	0.0213
0.75 - 0.73	845	845	100.0	23.62	2.09	32.91	0.1051	0.0243
0.73 - 0.71	939	939	100.0	22.60	1.97	30.65	0.1125	0.0269
0.71 - 0.70	520	520	100.0	22.68	1.58	26.06	0.1321	0.0313
0.70 - 0.68	1123	1123	100.0	22.30	1.59	25.24	0.1343	0.0321

0.68 - 0.67	616	616	100.0	21.97	1.39	22.75	0.1458	0.0358
0.67 - 0.66	643	643	100.0	20.89	1.32	21.22	0.1475	0.0386
0.66 - 0.65	714	714	100.0	20.62	1.16	19.38	0.1598	0.0427
0.65 - 0.64	763	799	95.5	17.19	1.01	16.06	0.1731	0.0583
0.74 - 0.64	5748	5784	99.4	21.31	1.50	23.96	0.1344	0.0347
Inf - 0.64	16226	16274	99.7	24.90	6.08	53.80	0.0538	0.0180

Complete .cif-data of the compound are available under the CCDC number **CCDC-XXXXXXX**.

The final structure refinement was carried out with using aspherical scattering factors with NoSpherA2.[1] DFT-calculated with ORCA using a B3LYP functional and def2-TZVPP basis set, whereby the H atom positions were refined using anisotropic atomic displacement parameters.

NoSpherA2 implementation of HAR makes use of tailor-made aspherical atomic form factors calculated on-the-fly from a Hirshfeld-partitioned electron density (ED) - not from spherical-atom form factors. The ED is calculated from a gaussian basis set single determinant SCF wave function - either Hartree-Fock or DFT using selected functional - for a fragment of the crystal. This fragment can be embedded in an electrostatic crystal field by employing cluster charges or modelled using implicit solvation models, depending on the software used. The following options were used:

SOFTWARE:	ORCA 5.0
PARTITIONING:	NoSpherA2
INT ACCURACY:	Normal
METHOD:	B3LYP
BASIS SET:	def2-TZVPP
CHARGE:	0
MULTIPLICITY:	1
DATE:	2025-02-12_14-25-31

Table S3. Crystal data and structure refinement of 15851.

Identification code	15851	
Empirical formula	$C_{17} H_{18} O_2$	
Color	colourless	
Formula weight	254.331 g·mol^{-1}	
Temperature	100(2) K	
Wavelength	0.71073 Å	
Crystal system	Monoclinic	
Space group	$P2_1$, (no. 4)	
Unit cell dimensions	a = 12.2689(14) Å	α= 90°.
	b = 11.416(1) Å	β= 92.442(3)°.
	c = 14.4948(15) Å	γ = 90°.
Volume	2028.3(4) Å^3	
Z	6	
Density (calculated)	1.249 Mg·m^{-3}	
Absorption coefficient	0.080 mm^{-1}	
F(000)	816.490 e	
Crystal size	0.409 x 0.316 x 0.190 mm^3	
θ range for data collection	2.13 to 33.79°.	
Index ranges	$-19 \le h \le 19$, $-17\le k \le 17$, $-22 \le l \le 22$	
Reflections collected	405123	
Independent reflections	16209 [R_{int} = 0.0541]	
Reflections with $I>2\sigma(I)$	15786	
Completeness to θ = 25.2417°	99.92 %	
Absorption correction	Numerical	
Max. and min. transmission	0.9943 and 0.9668	
Refinement method	Full-matrix least-squares on F^2	
Data / restraints / parameters	16209 / 1 / 1000	
Goodness-of-fit on F^2	1.2969	
Final R indices [$I>2\sigma(I)$]	R_1 = 0.0171	wR^2 = 0.0351
R indices (all data)	R_1 = 0.0181	wR^2 = 0.0355
Absolute structure parameter	0.08(7)	
Largest diff. peak and hole	0.1795 and -0.1981 e·Å^{-3}	

Table S4. Bond lengths [Å] and angles [°] of 15851.

O(1A)-C(1A)	1.4414(4)	O(1A)-C(11A)	1.4282(5)
O(2A)-C(3A)	1.2240(5)	C(1A)-C(2A)	1.5637(5)
C(1A)-C(7A)	1.5438(5)	C(1A)-C(10A)	1.5324(5)
C(2A)-H(2A)	1.069(6)	C(2A)-C(3A)	1.5197(5)
C(2A)-C(8A)	1.5135(5)	C(3A)-C(4A)	1.4718(5)
C(4A)-H(4A)	1.065(7)	C(4A)-C(5A)	1.3462(6)
C(5A)-H(5A)	1.065(6)	C(5A)-C(6A)	1.5059(6)
C(6A)-H(6A)	1.078(6)	C(6A)-C(7A)	1.5493(5)
C(6A)-C(9A)	1.5133(5)	C(7A)-H(7Aa)	1.080(6)
C(7A)-H(7Ab)	1.091(7)	C(8A)-H(8A)	1.085(6)
C(8A)-C(9A)	1.3315(6)	C(9A)-H(9A)	1.072(6)
C(10A)-H(10a)	1.083(7)	C(10A)-H(10b)	1.079(7)
C(10A)-H(10c)	1.083(7)	C(11A)-H(11a)	1.094(7)
C(11A)-H(11b)	1.095(8)	C(11A)-C(12A)	1.5035(5)
C(12A)-C(13A)	1.3941(6)	C(12A)-C(17A)	1.3968(5)
C(13A)-H(13A)	1.056(7)	C(13A)-C(14A)	1.3957(6)
C(14A)-H(14A)	1.096(7)	C(14A)-C(15A)	1.3893(6)
C(15A)-H(15A)	1.065(6)	C(15A)-C(16A)	1.3930(6)
C(16A)-H(16A)	1.090(8)	C(16A)-C(17A)	1.3916(6)
C(17A)-H(17A)	1.066(7)	O(1B)-C(1B)	1.4402(4)
O(1B)-C(11B)	1.4356(4)	O(2B)-C(3B)	1.2229(5)
C(1B)-C(2B)	1.5645(5)	C(1B)-C(7B)	1.5457(5)
C(1B)-C(10B)	1.5295(5)	C(2B)-H(2B)	1.076(6)
C(2B)-C(3B)	1.5260(5)	C(2B)-C(8B)	1.5111(5)
C(3B)-C(4B)	1.4727(5)	C(4B)-H(4B)	1.065(7)
C(4B)-C(5B)	1.3406(6)	C(5B)-H(5B)	1.085(6)
C(5B)-C(6B)	1.5087(6)	C(6B)-H(6B)	1.068(6)
C(6B)-C(7B)	1.5511(5)	C(6B)-C(9B)	1.5156(6)
C(7B)-H(7Ba)	1.108(6)	C(7B)-H(7Bb)	1.085(6)
C(8B)-H(8B)	1.086(6)	C(8B)-C(9B)	1.3332(5)
C(9B)-H(9B)	1.067(7)	C(10B)-H(10d)	1.064(6)
C(10B)-H(10e)	1.085(7)	C(10B)-H(10f)	1.079(7)
C(11B)-H(11c)	1.072(7)	C(11B)-H(11d)	1.094(7)
C(11B)-C(12B)	1.5018(5)	C(12B)-C(13B)	1.3959(5)
C(12B)-C(17B)	1.3967(5)	C(13B)-H(13B)	1.064(7)

C(13B)-C(14B)	1.3943(6)	C(14B)-H(14B)	1.048(7)
C(14B)-C(15B)	1.3936(6)	C(15B)-H(15B)	1.085(7)
C(15B)-C(16B)	1.3929(6)	C(16B)-H(16B)	1.072(7)
C(16B)-C(17B)	1.3936(5)	C(17B)-H(17B)	1.088(7)
O(1C)-C(1C)	1.4398(4)	O(1C)-C(5)	1.4180(4)
O(2C)-C(3C)	1.2195(5)	C(1C)-C(2C)	1.5569(5)
C(1C)-C(7C)	1.5470(5)	C(1C)-C(10C)	1.5283(5)
C(2C)-H(2C)	1.077(5)	C(2C)-C(3C)	1.5267(5)
C(2C)-C(8C)	1.5106(5)	C(3C)-C(4C)	1.4758(5)
C(4C)-H(4C)	1.091(7)	C(4C)-C(5C)	1.3433(6)
C(5)-H(5c)	1.082(6)	C(5)-H(5d)	1.096(6)
C(5)-C(11C)	1.5088(5)	C(5C)-H(5Ca)	1.061(6)
C(5C)-C(6C)	1.5111(7)	C(6C)-H(6C)	1.074(6)
C(6C)-C(7C)	1.5497(6)	C(6C)-C(9C)	1.5151(5)
C(7C)-H(7Ca)	1.118(6)	C(7C)-H(7Cb)	1.092(7)
C(8C)-H(8C)	1.082(6)	C(8C)-C(9C)	1.3321(5)
C(9C)-H(9C)	1.081(6)	C(10C)-H(10g)	1.084(6)
C(10C)-H(10h)	1.062(7)	C(10C)-H(10i)	1.067(7)
C(11C)-C(12C)	1.3955(5)	C(11C)-C(16C)	1.3949(5)
C(12C)-H(12C)	1.059(7)	C(12C)-C(13C)	1.3928(5)
C(13C)-H(13C)	1.073(6)	C(13C)-C(14C)	1.3931(6)
C(14C)-H(14C)	1.079(6)	C(14C)-C(15C)	1.3928(5)
C(15C)-H(15C)	1.090(7)	C(15C)-C(16C)	1.3933(5)
C(16C)-H(16C)	1.088(6)		

C(11A)-O(1A)-C(1A)	116.75(3)	C(2A)-C(1A)-O(1A)	111.04(3)
C(7A)-C(1A)-O(1A)	104.36(3)	C(7A)-C(1A)-C(2A)	111.47(3)
C(10A)-C(1A)-O(1A)	111.92(3)	C(10A)-C(1A)-C(2A)	107.18(3)
C(10A)-C(1A)-C(7A)	110.95(3)	H(2A)-C(2A)-C(1A)	108.6(4)
C(3A)-C(2A)-C(1A)	112.45(3)	C(3A)-C(2A)-H(2A)	105.9(4)
C(8A)-C(2A)-C(1A)	108.85(3)	C(8A)-C(2A)-H(2A)	110.3(3)
C(8A)-C(2A)-C(3A)	110.65(3)	C(2A)-C(3A)-O(2A)	119.93(3)
C(4A)-C(3A)-O(2A)	119.97(4)	C(4A)-C(3A)-C(2A)	120.07(3)
H(4A)-C(4A)-C(3A)	114.7(4)	C(5A)-C(4A)-C(3A)	124.40(4)
C(5A)-C(4A)-H(4A)	120.8(4)	H(5A)-C(5A)-C(4A)	117.8(4)
C(6A)-C(5A)-C(4A)	124.94(3)	C(6A)-C(5A)-H(5A)	117.2(4)
H(6A)-C(6A)-C(5A)	107.8(4)	C(7A)-C(6A)-C(5A)	111.64(3)
C(7A)-C(6A)-H(6A)	108.5(4)	C(9A)-C(6A)-C(5A)	110.42(3)

C(9A)-C(6A)-H(6A)	110.1(3)	C(9A)-C(6A)-C(7A)	108.27(3)
C(6A)-C(7A)-C(1A)	113.80(3)	H(7Aa)-C(7A)-C(1A)	107.6(3)
H(7Aa)-C(7A)-C(6A)	111.1(4)	H(7Ab)-C(7A)-C(1A)	109.4(3)
H(7Ab)-C(7A)-C(6A)	108.0(3)	H(7Ab)-C(7A)-H(7Aa)	106.6(5)
H(8A)-C(8A)-C(2A)	120.1(4)	C(9A)-C(8A)-C(2A)	118.20(3)
C(9A)-C(8A)-H(8A)	121.7(4)	C(8A)-C(9A)-C(6A)	117.35(3)
H(9A)-C(9A)-C(6A)	119.2(4)	H(9A)-C(9A)-C(8A)	123.4(4)
H(10a)-C(10A)-C(1A)	109.8(4)	H(10b)-C(10A)-C(1A)	111.8(4)
H(10b)-C(10A)-H(10a)	109.2(6)	H(10c)-C(10A)-C(1A)	112.2(4)
H(10c)-C(10A)-H(10a)	107.6(5)	H(10c)-C(10A)-H(10b)	106.1(6)
H(11a)-C(11A)-O(1A)	109.8(3)	H(11b)-C(11A)-O(1A)	110.4(4)
H(11b)-C(11A)-H(11a)	111.0(5)	C(12A)-C(11A)-O(1A)	107.83(3)
C(12A)-C(11A)-H(11a)	109.1(4)	C(12A)-C(11A)-H(11b)	108.6(4)
C(13A)-C(12A)-C(11A)	120.77(4)	C(17A)-C(12A)-C(11A)	120.38(4)
C(17A)-C(12A)-C(13A)	118.84(3)	H(13A)-C(13A)-C(12A)	119.7(4)
C(14A)-C(13A)-C(12A)	120.65(4)	C(14A)-C(13A)-H(13A)	119.7(4)
H(14A)-C(14A)-C(13A)	120.8(4)	C(15A)-C(14A)-C(13A)	119.94(4)
C(15A)-C(14A)-H(14A)	119.3(4)	H(15A)-C(15A)-C(14A)	119.4(4)
C(16A)-C(15A)-C(14A)	119.93(4)	C(16A)-C(15A)-H(15A)	120.7(4)
H(16A)-C(16A)-C(15A)	121.6(4)	C(17A)-C(16A)-C(15A)	119.88(4)
C(17A)-C(16A)-H(16A)	118.5(4)	C(16A)-C(17A)-C(12A)	120.75(4)
H(17A)-C(17A)-C(12A)	119.3(4)	H(17A)-C(17A)-C(16A)	120.0(4)
C(11B)-O(1B)-C(1B)	115.72(2)	C(2B)-C(1B)-O(1B)	109.73(3)
C(7B)-C(1B)-O(1B)	105.34(3)	C(7B)-C(1B)-C(2B)	111.71(3)
C(10B)-C(1B)-O(1B)	111.50(3)	C(10B)-C(1B)-C(2B)	108.73(3)
C(10B)-C(1B)-C(7B)	109.83(3)	H(2B)-C(2B)-C(1B)	108.3(3)
C(3B)-C(2B)-C(1B)	110.41(3)	C(3B)-C(2B)-H(2B)	106.0(3)
C(8B)-C(2B)-C(1B)	108.94(3)	C(8B)-C(2B)-H(2B)	111.0(3)
C(8B)-C(2B)-C(3B)	112.11(3)	C(2B)-C(3B)-O(2B)	120.11(3)
C(4B)-C(3B)-O(2B)	119.63(4)	C(4B)-C(3B)-C(2B)	120.26(3)
H(4B)-C(4B)-C(3B)	115.5(4)	C(5B)-C(4B)-C(3B)	124.51(4)
C(5B)-C(4B)-H(4B)	119.9(4)	H(5B)-C(5B)-C(4B)	117.1(4)
C(6B)-C(5B)-C(4B)	124.93(3)	C(6B)-C(5B)-H(5B)	118.0(4)
H(6B)-C(6B)-C(5B)	107.9(4)	C(7B)-C(6B)-C(5B)	109.64(3)
C(7B)-C(6B)-H(6B)	109.7(4)	C(9B)-C(6B)-C(5B)	110.83(3)
C(9B)-C(6B)-H(6B)	109.6(3)	C(9B)-C(6B)-C(7B)	109.14(3)
C(6B)-C(7B)-C(1B)	113.70(3)	H(7Ba)-C(7B)-C(1B)	109.7(3)
H(7Ba)-C(7B)-C(6B)	109.0(4)	H(7Bb)-C(7B)-C(1B)	107.3(3)

H(7Bb)-C(7B)-C(6B)	110.1(3)	H(7Bb)-C(7B)-H(7Ba)	106.9(6)
H(8B)-C(8B)-C(2B)	117.6(3)	C(9B)-C(8B)-C(2B)	117.90(3)
C(9B)-C(8B)-H(8B)	124.4(3)	C(8B)-C(9B)-C(6B)	117.86(3)
H(9B)-C(9B)-C(6B)	120.4(4)	H(9B)-C(9B)-C(8B)	121.8(4)
H(10d)-C(10B)-C(1B)	112.7(4)	H(10e)-C(10B)-C(1B)	110.1(4)
H(10e)-C(10B)-H(10d)	106.2(5)	H(10f)-C(10B)-C(1B)	110.2(3)
H(10f)-C(10B)-H(10d)	108.3(5)	H(10f)-C(10B)-H(10e)	109.2(6)
H(11c)-C(11B)-O(1B)	110.6(4)	H(11d)-C(11B)-O(1B)	109.8(3)
H(11d)-C(11B)-H(11c)	109.2(5)	C(12B)-C(11B)-O(1B)	107.38(3)
C(12B)-C(11B)-H(11c)	110.1(3)	C(12B)-C(11B)-H(11d)	109.8(3)
C(13B)-C(12B)-C(11B)	120.38(3)	C(17B)-C(12B)-C(11B)	120.56(3)
C(17B)-C(12B)-C(13B)	119.05(3)	H(13B)-C(13B)-C(12B)	119.2(4)
C(14B)-C(13B)-C(12B)	120.71(4)	C(14B)-C(13B)-H(13B)	120.1(3)
H(14B)-C(14B)-C(13B)	120.9(4)	C(15B)-C(14B)-C(13B)	119.71(3)
C(15B)-C(14B)-H(14B)	119.3(4)	H(15B)-C(15B)-C(14B)	120.1(4)
C(16B)-C(15B)-C(14B)	120.05(4)	C(16B)-C(15B)-H(15B)	119.8(4)
H(16B)-C(16B)-C(15B)	120.7(4)	C(17B)-C(16B)-C(15B)	119.94(4)
C(17B)-C(16B)-H(16B)	119.3(4)	C(16B)-C(17B)-C(12B)	120.51(3)
H(17B)-C(17B)-C(12B)	117.5(4)	H(17B)-C(17B)-C(16B)	122.0(4)
C(5)-O(1C)-C(1C)	116.65(3)	C(2C)-C(1C)-O(1C)	103.43(3)
C(7C)-C(1C)-O(1C)	110.92(3)	C(7C)-C(1C)-C(2C)	111.97(3)
C(10C)-C(1C)-O(1C)	110.98(3)	C(10C)-C(1C)-C(2C)	108.20(3)
C(10C)-C(1C)-C(7C)	111.08(3)	H(2C)-C(2C)-C(1C)	108.1(3)
C(3C)-C(2C)-C(1C)	111.32(3)	C(3C)-C(2C)-H(2C)	105.8(3)
C(8C)-C(2C)-C(1C)	110.22(3)	C(8C)-C(2C)-H(2C)	110.8(3)
C(8C)-C(2C)-C(3C)	110.51(3)	C(2C)-C(3C)-O(2C)	120.56(3)
C(4C)-C(3C)-O(2C)	120.60(4)	C(4C)-C(3C)-C(2C)	118.83(3)
H(4C)-C(4C)-C(3C)	113.2(4)	C(5C)-C(4C)-C(3C)	124.75(4)
C(5C)-C(4C)-H(4C)	122.0(4)	H(5c)-C(5)-O(1C)	110.5(4)
H(5d)-C(5)-O(1C)	110.7(3)	H(5d)-C(5)-H(5c)	107.3(5)
C(11C)-C(5)-O(1C)	108.92(3)	C(11C)-C(5)-H(5c)	109.9(3)
C(11C)-C(5)-H(5d)	109.6(3)	H(5Ca)-C(5C)-C(4C)	118.6(4)
C(6C)-C(5C)-C(4C)	125.48(3)	C(6C)-C(5C)-H(5Ca)	115.9(4)
H(6C)-C(6C)-C(5C)	107.5(4)	C(7C)-C(6C)-C(5C)	110.45(3)
C(7C)-C(6C)-H(6C)	108.5(4)	C(9C)-C(6C)-C(5C)	112.23(3)
C(9C)-C(6C)-H(6C)	111.2(4)	C(9C)-C(6C)-C(7C)	106.97(3)
C(6C)-C(7C)-C(1C)	113.00(3)	H(7Ca)-C(7C)-C(1C)	109.1(3)
H(7Ca)-C(7C)-C(6C)	108.6(4)	H(7Cb)-C(7C)-C(1C)	110.1(4)

H(7Cb)-C(7C)-C(6C)	107.3(4)	H(7Cb)-C(7C)-H(7Ca)	108.6(5)
H(8C)-C(8C)-C(2C)	118.7(3)	C(9C)-C(8C)-C(2C)	117.84(3)
C(9C)-C(8C)-H(8C)	123.5(3)	C(8C)-C(9C)-C(6C)	117.79(3)
H(9C)-C(9C)-C(6C)	119.4(4)	H(9C)-C(9C)-C(8C)	122.8(4)
H(10g)-C(10C)-C(1C)	109.5(4)	H(10h)-C(10C)-C(1C)	111.6(3)
H(10h)-C(10C)-H(10g)	107.8(6)	H(10i)-C(10C)-C(1C)	110.4(4)
H(10i)-C(10C)-H(10g)	110.3(6)	H(10i)-C(10C)-H(10h)	107.2(6)
C(12C)-C(11C)-C(5)	120.26(3)	C(16C)-C(11C)-C(5)	120.56(3)
C(16C)-C(11C)-C(12C)	119.15(3)	H(12C)-C(12C)-C(11C)	118.8(3)
C(13C)-C(12C)-C(11C)	120.80(3)	C(13C)-C(12C)-H(12C)	120.4(3)
H(13C)-C(13C)-C(12C)	121.2(4)	C(14C)-C(13C)-C(12C)	119.66(3)
C(14C)-C(13C)-H(13C)	119.2(4)	H(14C)-C(14C)-C(13C)	121.2(3)
C(15C)-C(14C)-C(13C)	119.89(3)	C(15C)-C(14C)-H(14C)	118.9(4)
H(15C)-C(15C)-C(14C)	119.2(3)	C(16C)-C(15C)-C(14C)	120.20(3)
C(16C)-C(15C)-H(15C)	120.6(3)	C(15C)-C(16C)-C(11C)	120.25(3)
H(16C)-C(16C)-C(11C)	118.5(3)	H(16C)-C(16C)-C(15C)	121.3(3)

Single crystal structure analysis of **15873**

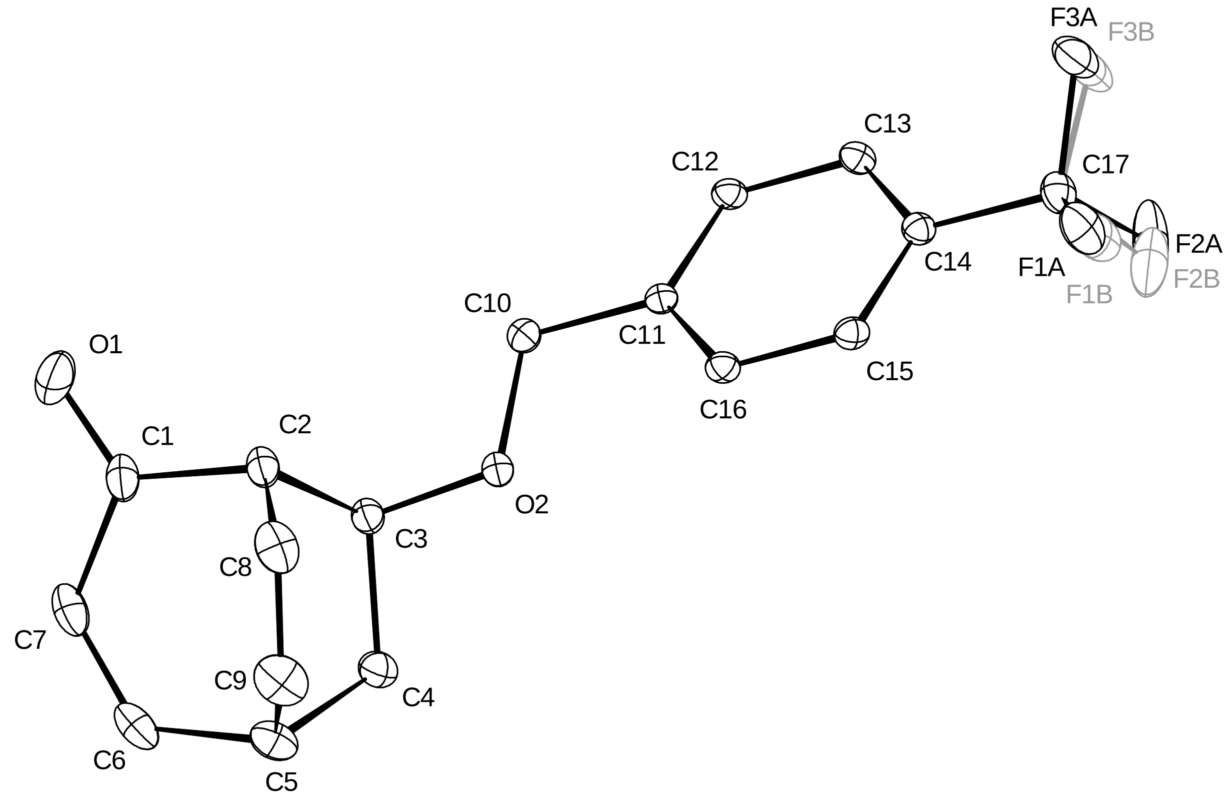

Figure S11. The asymmetric unit of **15873**. H atoms have been removed for clarity and disordered parts are shown in grey.

X-ray Crystal Structure Analysis of **15873**:

C_{17} H_{15} F_3 O_2, M_r = 308.303 g mol^{-1}, colourless block, crystal size 0.482 x 0.332 x 0.16 mm^3, monoclinic, space group $P2_1$ [4], a = 6.1329(3) Å, b = 6.8890(3) Å, c = 17.0270(8) Å, β= 99.836(2)°, V = 708.81(6) Å^3,T = 100(2) K, Z = 2, D_{calc} = 1.445g·cm^3, λ = 0.71073 Å, $\mu(Mo\text{-}K_\alpha)$ = 0.580 mm^{-1}, Gaussian correction ($T_{\min}$ = 0.95361, $T_{\max}$ = 0.98474), Bruker-AXS Kappa Mach3 with APEX-II detector and IμS microfocus Mo-anode X-ray source, 1.21 < θ< 36.32°, 42806 measured reflections, 6849 independent reflections, 6676 reflections with $I > 2\sigma(I)$, R_{int} = 0.0207. The structure was solved by *SHELXT* and refined by full-matrix least-squares (*SHELXL*). The final structure refinement was performed by olex2.refine 1.5 (L-M) together with NoSpherA2 (atomic form factors) against F^2 to R_1 = 0.0148 [$I > 2\sigma(I)$], wR_2 = 0.0342 [all data] with 362 parameters, 1 restraints and an absolute structure parameter Flack x = -0.01(7).

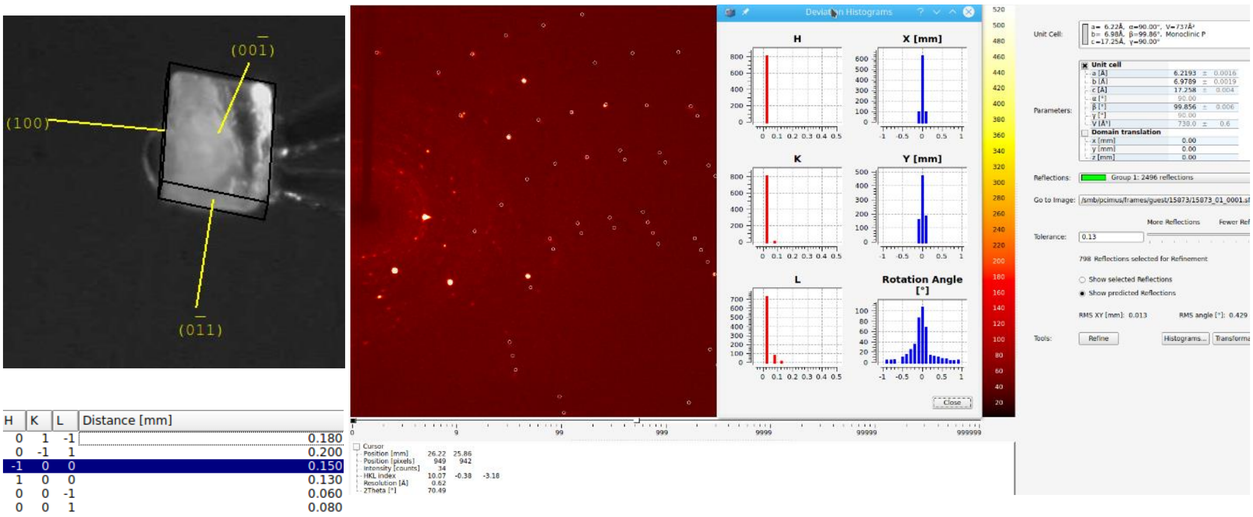

Figure S12. Crystal faces and unit cell determination/refinement of 15873.

INTENSITY STATISTICS FOR DATASET

Resolution	#Data	#Theory	%Complete	Redundancy	Mean I	Mean I/s	Rmerge	Rsigma
Inf - 2.40	107	107	100.0	10.63	135.53	143.86	0.0191	0.0069
2.40 - 1.63	252	252	100.0	11.79	47.02	136.64	0.0158	0.0065
1.63 - 1.27	369	369	100.0	11.70	21.90	118.00	0.0172	0.0074
1.27 - 1.12	345	345	100.0	11.17	17.97	105.71	0.0186	0.0079
1.12 - 1.01	354	354	100.0	8.95	15.41	89.10	0.0204	0.0092
1.01 - 0.94	367	367	100.0	7.21	8.01	68.98	0.0230	0.0121
0.94 - 0.88	370	370	100.0	6.34	6.34	59.51	0.0261	0.0139
0.88 - 0.84	336	336	100.0	5.80	4.44	48.14	0.0276	0.0167
0.84 - 0.80	417	417	100.0	5.63	3.99	43.18	0.0321	0.0191
0.80 - 0.77	320	320	100.0	5.27	5.04	45.72	0.0278	0.0180
0.77 - 0.74	434	434	100.0	5.15	3.50	36.88	0.0358	0.0220
0.74 - 0.72	331	331	100.0	4.78	3.61	33.92	0.0356	0.0234
0.72 - 0.70	316	317	99.7	4.73	3.52	34.44	0.0392	0.0247
0.70 - 0.68	402	403	99.8	4.55	3.07	30.23	0.0412	0.0272
0.68 - 0.66	446	447	99.8	4.28	3.02	27.32	0.0407	0.0287
0.66 - 0.65	258	262	98.5	4.19	2.46	24.61	0.0472	0.0335
0.65 - 0.63	508	517	98.3	4.13	2.20	22.95	0.0523	0.0367
0.63 - 0.62	280	284	98.6	3.75	2.25	20.67	0.0515	0.0393
0.62 - 0.61	298	303	98.3	3.89	1.86	19.55	0.0633	0.0443
0.61 - 0.60	351	362	97.0	3.64	1.64	16.66	0.0751	0.0498
0.60 - 0.59	261	343	76.1	1.69	1.83	14.74	0.0816	0.0602

```
--------------------------------------------------------------------------
---
0.69 - 0.59   2596  2713    95.7    3.74     2.27   21.95  0.0519  0.0379
Inf - 0.59   7122  7240    98.4    5.92     9.34   50.45  0.0207  0.0123
--------------------------------------------------------------------------
---
```

Complete .cif-data of the compound are available under the CCDC number **CCDC-2423659**.
A SHEL card (SHEL 999 0.6) was applied to the data set to exclude poorly determined intensities at high diffraction angles.

The final structure refinement was carried out with using aspherical scattering factors with NoSpherA2.[3] DFT-calculated with ORCA using a B3LYP functional and def2-TZVPP basis set, whereby the H atom positions were refined using anisotropic atomic displacement parameters.
NoSpherA2 implementation of HAR makes use of tailor-made aspherical atomic form factors calculated on-the-fly from a Hirshfeld-partitioned electron density (ED) - not from spherical-atom form factors. The ED is calculated from a gaussian basis set single determinant SCF wave function - either Hartree-Fock or DFT using selected functional - for a fragment of the crystal. This fragment can be embedded in an electrostatic crystal field by employing cluster charges or modelled using implicit solvation models, depending on the software used. The following options were used:

SOFTWARE:	ORCA 5.0
PARTITIONING:	NoSpherA2
INT ACCURACY:	High
METHOD:	B3LYP
BASIS SET:	def2-TZVPP
CHARGE:	0
MULTIPLICITY:	1
DATE:	2024-11-26_09-00-16

Table S5. Crystal data and structure refinement of 15873.

Identification code	15873	
Empirical formula	$C_{17} H_{15} F_3 O_2$	
Color	colourless	
Formula weight	308.303 g·mol^{-1}	
Temperature	100(2) K	
Wavelength	0.71073 Å	
Crystal system	Monoclinic	
Space group	$P2_1$, (no. 4)	
Unit cell dimensions	a = 6.1329(3) Å	α= 90°.
	b = 6.8890(3) Å	β= 99.836(2)°.
	c = 17.0270(8) Å	γ = 90°.
Volume	708.81(6) Å^3	
Z	2	
Density (calculated)	1.445 Mg·m^{-3}	
Absorption coefficient	0.120 mm^{-1}	
F(000)	320.271 e	
Crystal size	0.482 x 0.332 x 0.16 mm^3	
θ range for data collection	1.21 to 36.32°.	
Index ranges	$-10 \le h \le 10, -11\le k \le 11, -28 \le l \le 28$	
Reflections collected	42806	
Independent reflections	6849 [R_{int} = 0.0207]	
Reflections with $I>2\sigma(I)$	6676	
Completeness to θ = 25.2417°	100.00 %	
Absorption correction	Gaussian	
Max. and min. transmission	0.98474 and 0.95361	
Refinement method	Full-matrix least-squares on F^2	
Data / restraints / parameters	6849 / 1 / 362	
Goodness-of-fit on F^2	1.2600	
Final R indices [$I>2\sigma(I)$]	R_1 = 0.0148	wR^2 = 0.0340
R indices (all data)	R_1 = 0.0157	wR^2 = 0.0342
Absolute structure parameter	-0.01(7)	
Largest diff. peak and hole	0.1297 and -0.0753 e·Å^{-3}	

Table S6. Bond lengths [Å] and angles [°] of 15873.

F(1A)-C(17)	1.349(2)	F(2A)-C(17)	1.335(3)
F(3A)-C(17)	1.367(3)	O(1)-C(1)	1.2230(5)
O(2)-C(3)	1.4291(4)	O(2)-C(10)	1.4120(4)
C(1)-C(2)	1.5247(4)	C(1)-C(7)	1.4714(5)
C(2)-H(2)	1.083(6)	C(2)-C(3)	1.5590(4)
C(2)-C(8)	1.5110(5)	C(3)-H(3)	1.087(5)
C(3)-C(4)	1.5375(4)	C(4)-H(4b)	1.075(6)
C(4)-H(4a)	1.086(7)	C(4)-C(5)	1.5473(5)
C(5)-H(5)	1.085(7)	C(5)-C(6)	1.5088(5)
C(5)-C(9)	1.5125(5)	C(6)-H(6)	1.095(6)
C(6)-C(7)	1.3461(6)	C(7)-H(7)	1.079(6)
C(8)-H(8)	1.069(7)	C(8)-C(9)	1.3350(6)
C(9)-H(9)	1.089(7)	C(10)-H(10a)	1.115(5)
C(10)-H(10b)	1.095(5)	C(10)-C(11)	1.5047(4)
C(11)-C(12)	1.3976(4)	C(11)-C(16)	1.3967(4)
C(12)-H(12)	1.087(5)	C(12)-C(13)	1.3908(4)
C(13)-H(13)	1.082(5)	C(13)-C(14)	1.3963(4)
C(14)-C(15)	1.3953(4)	C(14)-C(17)	1.4987(4)
C(15)-H(15)	1.089(5)	C(15)-C(16)	1.3940(4)
C(16)-H(16)	1.062(5)	C(17)-F(3B)	1.280(5)
C(17)-F(2B)	1.347(4)	C(17)-F(1B)	1.302(5)
C(10)-O(2)-C(3)	114.61(2)	C(2)-C(1)-O(1)	120.43(3)
C(7)-C(1)-O(1)	120.18(3)	C(7)-C(1)-C(2)	119.36(3)
H(2)-C(2)-C(1)	105.8(3)	C(3)-C(2)-C(1)	109.91(2)
C(3)-C(2)-H(2)	108.4(3)	C(8)-C(2)-C(1)	111.82(3)
C(8)-C(2)-H(2)	111.3(3)	C(8)-C(2)-C(3)	109.50(3)
C(2)-C(3)-O(2)	109.63(2)	H(3)-C(3)-O(2)	108.1(3)
H(3)-C(3)-C(2)	109.0(3)	C(4)-C(3)-O(2)	106.13(2)
C(4)-C(3)-C(2)	112.78(2)	C(4)-C(3)-H(3)	111.1(3)
H(4b)-C(4)-C(3)	111.4(3)	H(4a)-C(4)-C(3)	106.5(3)
H(4a)-C(4)-H(4b)	108.3(5)	C(5)-C(4)-C(3)	112.96(3)
C(5)-C(4)-H(4b)	108.7(3)	C(5)-C(4)-H(4a)	108.8(4)
H(5)-C(5)-C(4)	109.5(3)	C(6)-C(5)-C(4)	109.72(3)
C(6)-C(5)-H(5)	108.2(3)	C(9)-C(5)-C(4)	108.34(3)

C(9)-C(5)-H(5)	109.4(3)	C(9)-C(5)-C(6)	111.72(3)
H(6)-C(6)-C(5)	117.9(4)	C(7)-C(6)-C(5)	125.26(3)
C(7)-C(6)-H(6)	116.8(4)	C(6)-C(7)-C(1)	124.79(3)
H(7)-C(7)-C(1)	115.6(4)	H(7)-C(7)-C(6)	119.6(4)
H(8)-C(8)-C(2)	120.0(4)	C(9)-C(8)-C(2)	117.94(3)
C(9)-C(8)-H(8)	122.1(4)	C(8)-C(9)-C(5)	117.74(3)
H(9)-C(9)-C(5)	120.5(4)	H(9)-C(9)-C(8)	121.8(4)
H(10a)-C(10)-O(2)	110.7(3)	H(10b)-C(10)-O(2)	110.7(3)
H(10b)-C(10)-H(10a)	106.4(5)	C(11)-C(10)-O(2)	108.89(2)
C(11)-C(10)-H(10a)	110.0(3)	C(11)-C(10)-H(10b)	110.2(3)
C(12)-C(11)-C(10)	119.19(2)	C(16)-C(11)-C(10)	121.50(2)
C(16)-C(11)-C(12)	119.30(3)	H(12)-C(12)-C(11)	119.9(3)
C(13)-C(12)-C(11)	120.87(2)	C(13)-C(12)-H(12)	119.2(3)
H(13)-C(13)-C(12)	119.6(3)	C(14)-C(13)-C(12)	119.32(2)
C(14)-C(13)-H(13)	121.1(3)	C(15)-C(14)-C(13)	120.42(3)
C(17)-C(14)-C(13)	119.20(2)	C(17)-C(14)-C(15)	120.36(2)
H(15)-C(15)-C(14)	120.6(3)	C(16)-C(15)-C(14)	119.79(2)
C(16)-C(15)-H(15)	119.6(3)	C(15)-C(16)-C(11)	120.29(2)
H(16)-C(16)-C(11)	119.3(3)	H(16)-C(16)-C(15)	120.4(3)
F(2A)-C(17)-F(1A)	111.94(19)	F(3A)-C(17)-F(1A)	103.74(19)
F(3A)-C(17)-F(2A)	106.1(2)	C(14)-C(17)-F(1A)	110.96(15)
C(14)-C(17)-F(2A)	112.81(18)	C(14)-C(17)-F(3A)	110.78(17)
F(3B)-C(17)-F(1A)	108.9(3)	F(3B)-C(17)-F(2A)	96.4(3)
F(3B)-C(17)-F(3A)	9.8(3)	F(3B)-C(17)-C(14)	115.0(3)
F(2B)-C(17)-F(1A)	104.0(3)	F(2B)-C(17)-F(2A)	10.4(4)
F(2B)-C(17)-F(3A)	115.5(3)	F(2B)-C(17)-C(14)	111.4(3)
F(2B)-C(17)-F(3B)	105.8(4)	F(1B)-C(17)-F(1A)	8.6(2) F(1B)-
C(17)-F(2A)	103.4(2)	F(1B)-C(17)-F(3A)	107.5(2)
F(1B)-C(17)-C(14)	115.6(3)	F(1B)-C(17)-F(3B)	111.4(4)
F(1B)-C(17)-F(2B)	95.5(3)		

Several attempts were made to grow single crystals of diffraction-quality from various solvents. All were unsuccessful and resulted in a polycrystalline powder. We therefore investigated the potential for melt crystallisation from the pure liquid. Despite the limited amount of sample available, further DSC measurements were not possible to investigate the phase behaviour at higher temperatures.

To obtain information on melting point and possible crystallisation, a heating stage was added to the optical polarisation microscope. The major advantage of this method is that less than 1 mg of sample is required. The microscope was a Nikkon SMZ1500 with polarisation filters and a high flux LED (Alu-Star LZ ML, 10W, 3.1V, 1500mA, 260lm, 5000K) light source. The microscope is also equipped with a CMOS camera (Bresser MikroCam II Full HD HSP) to obtain high resolution images of small crystallites. For thermal treatment of the sample, a Reichert Thermogeräte micro hot stage was placed on the optical axis of the Nikkon microscope. The hot stage was electrically connected to the control transformer and a liquid thermometer (20 - 230°C) was used to estimate the temperature.

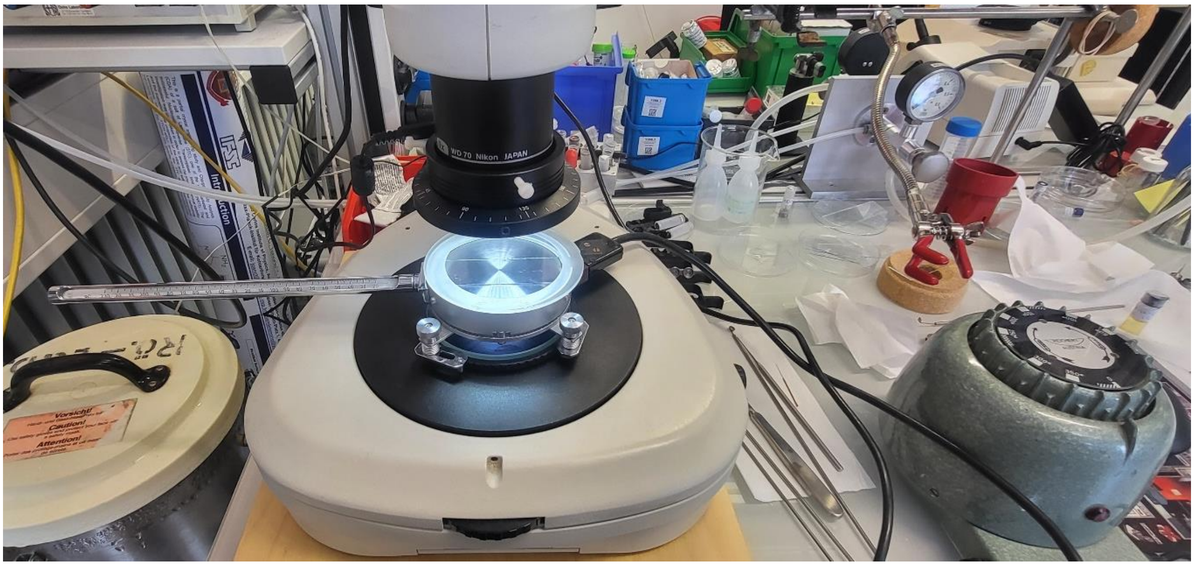

Figure S13: Experimental set-up for determining the melting and solidification temperature as well as for crystallisation from the melt.

A microspatula tip of the compound was first placed on a glass microscope slide. This was transferred to the hot stage and covered with a glass plate on top of the oven. Several images were taken at different temperatures to determine the melting and crystallisation points of the compound.

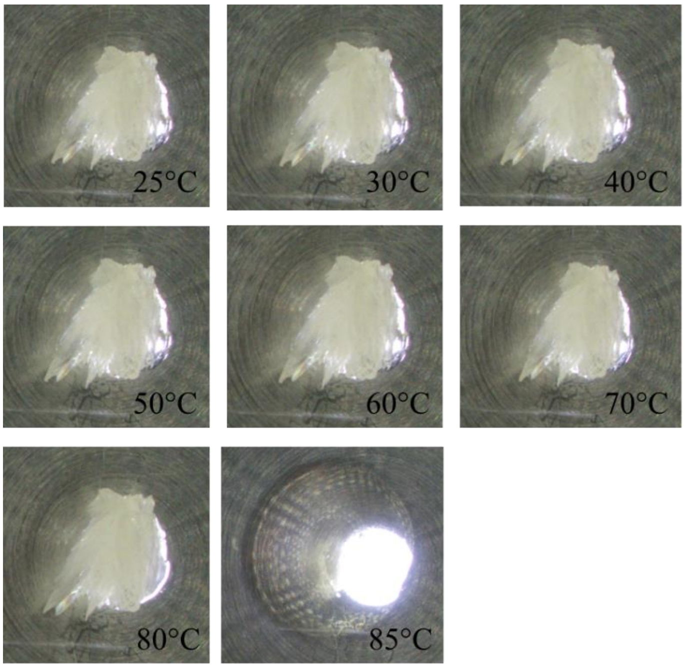

Figure S14: Microscopic images of the compound at different temperatures during the first melting cycle.

When the compound was heated slowly, it was found that slight changes in crystal shape occur around 80°C. At 83°C the solid spontaneously transforms into a liquid phase. The melting point was therefore qualitatively estimated to be 83°C. To grow crystals, the melt was then allowed to cool gently. At 72°C, small crystallites were obtained in the outer regions of the melt. These grow over time by Osswald ripening. With these initial crystalline seeds, the hot stage was reheated to 80°C to re-melt most of the solid fraction. In a second cycle, the melt was allowed to cool gently. The remaining crystals grew to a suitable size and could later be used for the diffraction experiment.

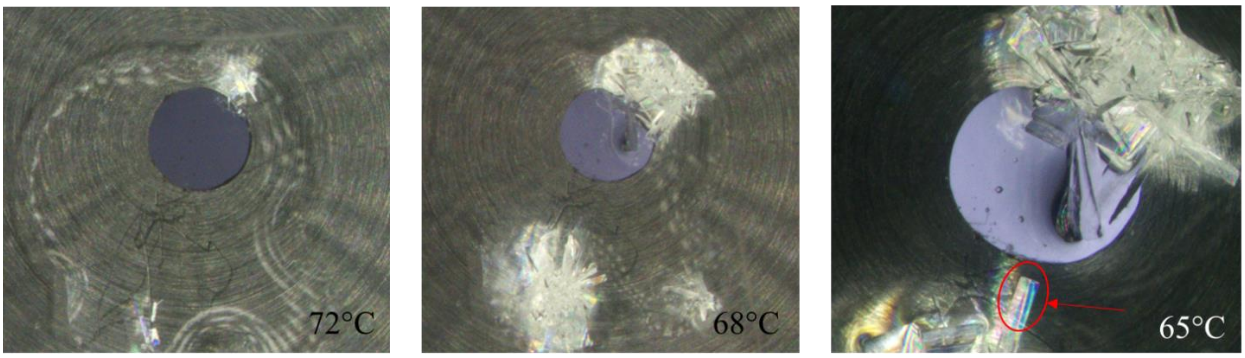

Figure S15: Images of recrystallisation from the melt. The crystal selected for diffraction is marked with a red circle.

Single crystal structure analysis of **15904**

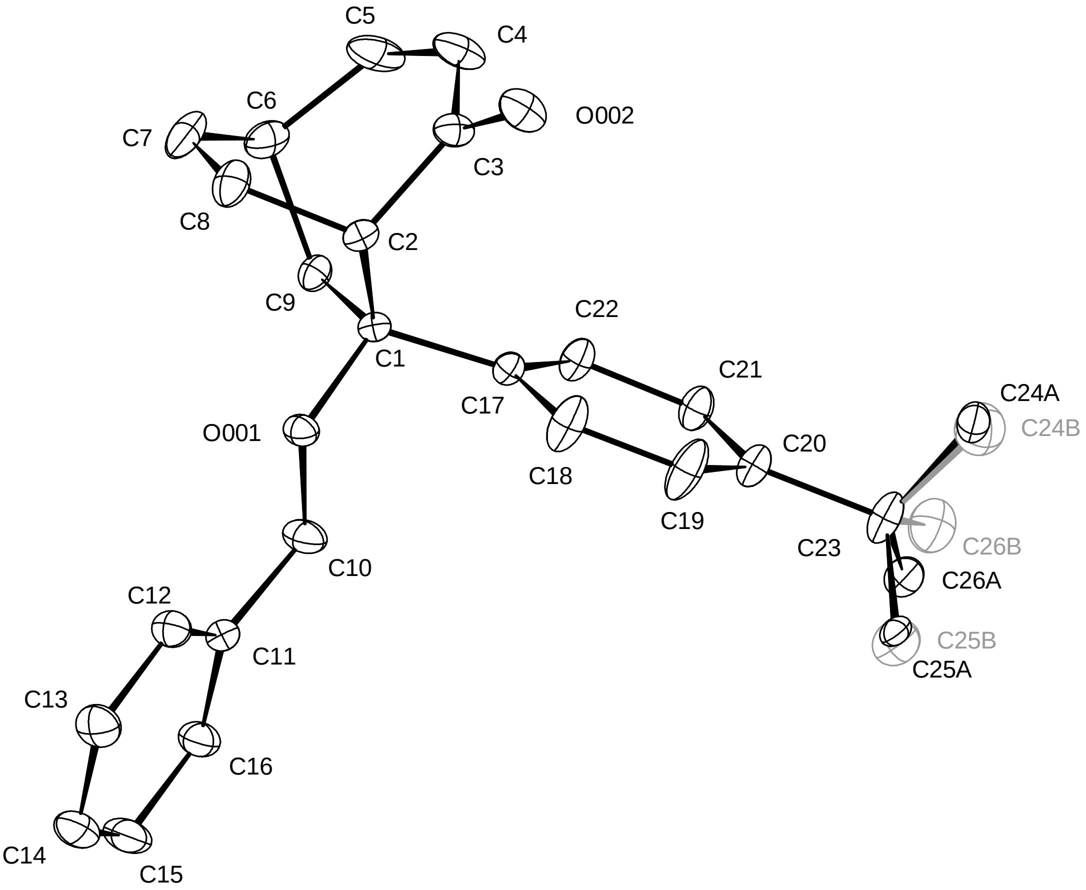

Figure S16. The asymmetric unit of **15904**. H atoms have been removed for clarity and disordered parts are shown in grey.

X-ray Crystal Structure Analysis of 15904:

C_{26} H_{28} O_2, M_r = 372.511 g mol^{-1}, colourless prism, crystal size 0.134 x 0.096 x 0.06 mm^3, orthorhombic, space group $P2_12_12_1$ [19], a = 6.1903(2) Å, b = 11.0711(4) Å, c = 30.2942(12) Å, V = 2076.17(13) Å^3,T = 100(2) K, Z = 4, D_{calc} = 1.192 g·cm^3, λ = 1.54178 Å, $\mu(Cu\text{-}K_\alpha)$ = 0.571 mm^{-1}, Gaussian correction ($T_{\min}$ = 0.94746, $T_{\max}$ = 0.97317),), Bruker-AXS Kappa Mach3 with APEX-II detector and FR591 rotating anode X-ray source, 2.92 < θ < 72.14°, 75733 measured reflections, 4070 independent reflections, 3831 reflections with $I > 2\sigma(I)$, R_{int} = 0.0413. The structure was solved by *SHELXT* and refined by full-matrix least-squares (*SHELXL*). The final structure refinement was performed by olex2.refine 1.5 (L-M) together with NoSpherA2 (atomic form factors) against F^2 to R_1 = 0.0277 [$I > 2\sigma(I)$], wR_2 = 0.0662 [all data] with 344 parameters, 38 restraints and an absolute structure parameter Flack x = 0.14(4).

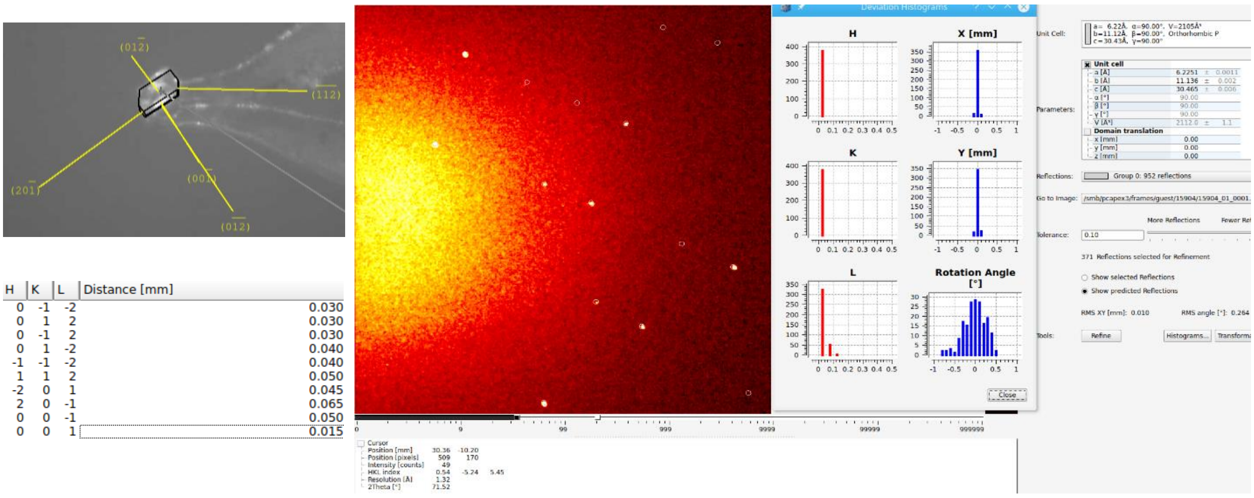

Figure S17. Crystal faces and unit cell determination/refinement of **15904**.

INTENSITY STATISTICS FOR DATASET

Resolution	#Data	#Theory	%Complete	Redundancy	Mean I	Mean I/s	Rmerge	Rsigma
Inf - 3.37	63	63	100.0	17.60	181.56	115.61	0.0281	0.0073
3.37 - 2.23	143	143	100.0	17.91	71.18	110.32	0.0260	0.0075
2.23 - 1.77	205	205	100.0	21.09	45.40	101.38	0.0287	0.0080
1.77 - 1.53	208	208	100.0	19.78	23.22	75.95	0.0347	0.0104
1.53 - 1.39	208	208	100.0	17.52	15.48	55.71	0.0436	0.0139
1.39 - 1.29	203	203	100.0	14.91	12.97	43.94	0.0535	0.0180
1.29 - 1.21	212	212	100.0	26.86	21.44	78.52	0.0398	0.0102
1.21 - 1.15	209	209	100.0	30.14	17.76	81.33	0.0421	0.0097
1.15 - 1.10	205	205	100.0	28.54	13.28	68.05	0.0471	0.0115
1.10 - 1.06	186	186	100.0	26.95	12.94	66.37	0.0499	0.0123
1.06 - 1.02	221	221	100.0	26.62	11.36	59.93	0.0550	0.0133
1.02 - 0.99	201	201	100.0	23.34	8.78	51.09	0.0676	0.0162
0.99 - 0.96	234	234	100.0	21.57	7.73	45.86	0.0644	0.0182
0.96 - 0.93	225	225	100.0	20.92	5.59	37.63	0.0818	0.0222
0.93 - 0.91	171	171	100.0	20.39	4.72	34.23	0.0910	0.0240
0.91 - 0.89	211	211	100.0	19.12	3.59	29.31	0.0967	0.0291
0.89 - 0.87	217	217	100.0	17.29	4.61	31.92	0.0925	0.0253
0.87 - 0.85	246	246	100.0	4.57	3.96	9.89	0.1302	0.0990
0.85 - 0.84	125	125	100.0	3.55	4.33	9.03	0.1047	0.0959
0.84 - 0.82	290	291	99.7	3.31	3.79	8.74	0.1103	0.1039
0.82 - 0.81	116	156	74.4	1.58	3.10	5.94	0.1318	0.1656

```
------------------------------------------------------------------------------
---
0.91 - 0.81   1205  1246   96.7    8.48     3.93  16.51  0.1004  0.0781
Inf - 0.81   4099  4140   99.0   18.36    16.56  50.88  0.0412  0.0152
------------------------------------------------------------------------------
---
```

Complete .cif-data of the compound are available under the CCDC number **CCDC-2423661**.

The distances between C23 and C24A and C23 and C25B have been treated with the DFIX instruction. To treat the atomic displacement parameters of C25A, C25B, C24A, C24B, C26A and C26B, the ISOR command was applied.

The final structure refinement was carried out with using aspherical scattering factors with NoSpherA2.[1] DFT-calculated with ORCA using a B3LYP functional and def2-TZVPP basis set, whereby the H atom positions were refined using anisotropic atomic displacement parameters.

NoSpherA2 implementation of HAR makes use of tailor-made aspherical atomic form factors calculated on-the-fly from a Hirshfeld-partitioned electron density (ED) - not from spherical-atom form factors. The ED is calculated from a gaussian basis set single determinant SCF wave function - either Hartree-Fock or DFT using selected functional - for a fragment of the crystal. This fragment can be embedded in an electrostatic crystal field by employing cluster charges or modelled using implicit solvation models, depending on the software used. The following options were used:

SOFTWARE:	ORCA 5.0
PARTITIONING:	NoSpherA2
INT ACCURACY:	Normal
METHOD:	B3LYP
BASIS SET:	def2-TZVPP
CHARGE:	0
MULTIPLICITY:	1
DATE:	2025-02-12_13-55-28

Table S7. Crystal data and structure refinement of **15904**.

Identification code	15904	
Empirical formula	$C_{26} H_{28} O_2$	
Color	colourless	
Formula weight	372.511 g·mol^{-1}	
Temperature	100(2) K	
Wavelength	1.54178 Å	
Crystal system	Orthorhombic	
Space group	$P2_12_12_1$, (no. 19)	
Unit cell dimensions	a = 6.1903(2) Å	α= 90°.
	b = 11.0711(4) Å	β= 90°.
	c = 30.2942(12) Å	γ = 90°.
Volume	2076.17(13) Å^3	
Z	4	
Density (calculated)	1.192 Mg·m^{-3}	
Absorption coefficient	0.571 mm^{-1}	
F(000)	802.416 e	
Crystal size	0.134 x 0.096 x 0.06 mm^3	
θ range for data collection	2.92 to 72.14°.	
Index ranges	-7 ≤ h ≤ 7, -13≤ k ≤ 13, -36 ≤ l ≤ 37	
Reflections collected	75733	
Independent reflections	4070 [R_{int} = 0.0413]	
Reflections with I>2σ(I)	3831	
Completeness to θ = 67.6786°	100.00 %	
Absorption correction	Gaussian	
Max. and min. transmission	0.97317 and 0.94746	
Refinement method	Full-matrix least-squares on F^2	
Data / restraints / parameters	4070 / 38 / 344	
Goodness-of-fit on F^2	1.0584	
Final R indices [I>2σ(I)]	R_1 = 0.0277	wR^2 = 0.0639
R indices (all data)	R_1 = 0.0309	wR^2 = 0.0662
Absolute structure parameter	0.14(4)	
Largest diff. peak and hole	0.2319 and -0.2227 e·Å^{-3}	

Table S8. Bond lengths [Å] and angles [°] of **15904**.

O(001)-C(1)	1.4499(13)	O(001)-C(10)	1.4136(14)
O(002)-C(3)	1.2247(15)	C(1)-C(2)	1.5622(16)
C(1)-C(9)	1.5493(16)	C(1)-C(17)	1.5312(15)
C(2)-H(2)	1.087(14)	C(2)-C(3)	1.5204(17)
C(2)-C(8)	1.5100(16)	C(3)-C(4)	1.4729(17)
C(4)-H(4)	1.096(15)	C(4)-C(5)	1.3435(19)
C(5)-H(5)	1.047(16)	C(5)-C(6)	1.5055(18)
C(6)-H(6)	1.041(14)	C(6)-C(7)	1.513(2)
C(6)-C(9)	1.5490(17)	C(7)-H(7)	1.076(15)
C(7)-C(8)	1.331(2)	C(8)-H(8)	1.096(16)
C(9)-H(9a)	1.059(14)	C(9)-H(9b)	1.072(14)
C(10)-H(10a)	1.128(15)	C(10)-H(10b)	1.104(14)
C(10)-C(11)	1.5036(17)	C(11)-C(12)	1.3968(18)
C(11)-C(16)	1.3929(18)	C(12)-H(12)	1.079(14)
C(12)-C(13)	1.3980(18)	C(13)-H(13)	1.037(16)
C(13)-C(14)	1.388(2)	C(14)-H(14)	1.087(16)
C(14)-C(15)	1.393(2)	C(15)-H(15)	1.083(16)
C(15)-C(16)	1.3886(18)	C(16)-H(16)	1.070(15)
C(17)-C(18)	1.3962(17)	C(17)-C(22)	1.3918(17)
C(18)-H(18)	1.055(16)	C(18)-C(19)	1.3877(18)
C(19)-H(19)	1.087(16)	C(19)-C(20)	1.3997(18)
C(20)-C(21)	1.3891(17)	C(20)-C(23)	1.5321(16)
C(21)-H(21)	1.084(14)	C(21)-C(22)	1.3980(17)
C(22)-H(22)	1.123(14)	C(23)-C(24A)	1.5627(17)
C(23)-C(25A)	1.517(3)	C(23)-C(26A)	1.543(3)
C(23)-C(24B)	1.424(6)	C(23)-C(25B)	1.5526(19)
C(23)-C(26B)	1.579(4)	C(24A)-H(24a)	0.9800
C(24A)-H(24b)	0.9800	C(24A)-H(24c)	0.9800
C(25A)-H(25a)	0.9800	C(25A)-H(25b)	0.9800
C(25A)-H(25c)	0.9800	C(26A)-H(26a)	0.9800
C(26A)-H(26b)	0.9800	C(26A)-H(26c)	0.9800
C(24B)-H(24d)	0.9800	C(24B)-H(24e)	0.9800
C(24B)-H(24f)	0.9800	C(25B)-H(25d)	0.9800
C(25B)-H(25e)	0.9800	C(25B)-H(25f)	0.9800
C(26B)-H(26d)	0.9800	C(26B)-H(26e)	0.9800
C(26B)-H(26f)	0.9800		

C(10)-O(001)-C(1)	115.30(9)	C(2)-C(1)-O(001)	101.01(9)
C(9)-C(1)-O(001)	109.35(9)	C(9)-C(1)-C(2)	110.99(9)
C(17)-C(1)-O(001)	108.82(9)	C(17)-C(1)-C(2)	110.44(9)
C(17)-C(1)-C(9)	115.26(9)	H(2)-C(2)-C(1)	107.0(7)
C(3)-C(2)-C(1)	112.73(10)	C(3)-C(2)-H(2)	106.1(7)
C(8)-C(2)-C(1)	110.09(10)	C(8)-C(2)-H(2)	111.3(7)
C(8)-C(2)-C(3)	109.56(10)	C(2)-C(3)-O(002)	120.55(11)
C(4)-C(3)-O(002)	120.12(11)	C(4)-C(3)-C(2)	119.33(11)
H(4)-C(4)-C(3)	115.5(8)	C(5)-C(4)-C(3)	124.51(12)
C(5)-C(4)-H(4)	120.0(8)	H(5)-C(5)-C(4)	118.6(9)
C(6)-C(5)-C(4)	124.71(12)	C(6)-C(5)-H(5)	116.7(8)
H(6)-C(6)-C(5)	107.8(8)	C(7)-C(6)-C(5)	113.50(12)
C(7)-C(6)-H(6)	111.5(8)	C(9)-C(6)-C(5)	108.54(11)
C(9)-C(6)-H(6)	107.0(8)	C(9)-C(6)-C(7)	108.30(11)
H(7)-C(7)-C(6)	120.4(9)	C(8)-C(7)-C(6)	117.28(11)
C(8)-C(7)-H(7)	122.2(9)	C(7)-C(8)-C(2)	117.98(12)
H(8)-C(8)-C(2)	117.6(8)	H(8)-C(8)-C(7)	124.4(8)
C(6)-C(9)-C(1)	113.65(10)	H(9a)-C(9)-C(1)	110.8(7)
H(9a)-C(9)-C(6)	109.4(7)	H(9b)-C(9)-C(1)	108.4(7)
H(9b)-C(9)-C(6)	107.4(7)	H(9b)-C(9)-H(9a)	107.0(10)
H(10a)-C(10)-O(001)	111.3(7)	H(10b)-C(10)-O(001)	108.3(7)
H(10b)-C(10)-H(10a)	109.6(10)	C(11)-C(10)-O(001)	110.19(10)
C(11)-C(10)-H(10a)	108.7(7)	C(11)-C(10)-H(10b)	108.7(7)
C(12)-C(11)-C(10)	123.03(11)	C(16)-C(11)-C(10)	117.95(11)
C(16)-C(11)-C(12)	119.01(11)	H(12)-C(12)-C(11)	122.0(8)
C(13)-C(12)-C(11)	119.90(12)	C(13)-C(12)-H(12)	118.0(8)
H(13)-C(13)-C(12)	119.7(8)	C(14)-C(13)-C(12)	120.65(13)
C(14)-C(13)-H(13)	119.6(8)	H(14)-C(14)-C(13)	120.4(8)
C(15)-C(14)-C(13)	119.48(12)	C(15)-C(14)-H(14)	120.1(8)
H(15)-C(15)-C(14)	120.8(8)	C(16)-C(15)-C(14)	119.95(13)
C(16)-C(15)-H(15)	119.2(8)	C(15)-C(16)-C(11)	121.02(13)
H(16)-C(16)-C(11)	118.1(8)	H(16)-C(16)-C(15)	120.8(8)
C(18)-C(17)-C(1)	118.89(10)	C(22)-C(17)-C(1)	124.30(10)
C(22)-C(17)-C(18)	116.77(10)	H(18)-C(18)-C(17)	118.8(8)
C(19)-C(18)-C(17)	121.72(12)	C(19)-C(18)-H(18)	119.5(8)
H(19)-C(19)-C(18)	117.2(8)	C(20)-C(19)-C(18)	121.86(12)
C(20)-C(19)-H(19)	121.0(8)	C(21)-C(20)-C(19)	116.10(11)

C(23)-C(20)-C(19)	121.28(11)	C(23)-C(20)-C(21)	122.58(11)
H(21)-C(21)-C(20)	119.9(7)	C(22)-C(21)-C(20)	122.32(12)
C(22)-C(21)-H(21)	117.7(7)	C(21)-C(22)-C(17)	121.15(11)
H(22)-C(22)-C(17)	122.2(7)	H(22)-C(22)-C(21)	116.7(7)
C(24A)-C(23)-C(20)	107.41(13)	C(25A)-C(23)-C(20)	111.91(18)
C(25A)-C(23)-C(24A)	110.3(2)	C(26A)-C(23)-C(20)	112.20(13)
C(26A)-C(23)-C(24A)	104.50(18)	C(26A)-C(23)-C(25A)	110.2(2)
C(24B)-C(23)-C(20)	114.2(3)	C(24B)-C(23)-C(24A)	16.2(3)
C(24B)-C(23)-C(25A)	117.7(3)	C(24B)-C(23)-C(26A)	88.3(3)
C(25B)-C(23)-C(20)	108.8(2)	C(25B)-C(23)-C(24A)	102.5(2)
C(25B)-C(23)-C(25A)	10.6(3)	C(25B)-C(23)-C(26A)	120.1(2)
C(25B)-C(23)-C(24B)	112.1(3)	C(26B)-C(23)-C(20)	108.56(17)
C(26B)-C(23)-C(24A)	126.0(2)	C(26B)-C(23)-C(25A)	91.8(2)
C(26B)-C(23)-C(26A)	23.40(14)	C(26B)-C(23)-C(24B)	110.1(3)
C(26B)-C(23)-C(25B)	102.4(3)	H(24a)-C(24A)-C(23)	109.5 H(24b)-
C(24A)-C(23)	109.5	H(24b)-C(24A)-H(24a)	109.5 H(24c)-
C(24A)-C(23)	109.5	H(24c)-C(24A)-H(24a)	109.5 H(24c)-
C(24A)-H(24b)	109.5	H(25a)-C(25A)-C(23)	109.5 H(25b)-
C(25A)-C(23)	109.5	H(25b)-C(25A)-H(25a)	109.5 H(25c)-
C(25A)-C(23)	109.5	H(25c)-C(25A)-H(25a)	109.5 H(25c)-
C(25A)-H(25b)	109.5	H(26a)-C(26A)-C(23)	109.5 H(26b)-
C(26A)-C(23)	109.5	H(26b)-C(26A)-H(26a)	109.5 H(26c)-
C(26A)-C(23)	109.5	H(26c)-C(26A)-H(26a)	109.5 H(26c)-
C(26A)-H(26b)	109.5	H(24d)-C(24B)-C(23)	109.5 H(24e)-
C(24B)-C(23)	109.5	H(24e)-C(24B)-H(24d)	109.5 H(24f)-
C(24B)-C(23)	109.5	H(24f)-C(24B)-H(24d)	109.5 H(24f)-
C(24B)-H(24e)	109.5	H(25d)-C(25B)-C(23)	109.5 H(25e)-
C(25B)-C(23)	109.5	H(25e)-C(25B)-H(25d)	109.5 H(25f)-
C(25B)-C(23)	109.5	H(25f)-C(25B)-H(25d)	109.5 H(25f)-
C(25B)-H(25e)	109.5	H(26d)-C(26B)-C(23)	109.5 H(26e)-
C(26B)-C(23)	109.5	H(26e)-C(26B)-H(26d)	109.5 H(26f)-
C(26B)-C(23)	109.5	H(26f)-C(26B)-H(26d)	109.5 H(26f)-
C(26B)-H(26e)	109.5		

15. NMR spectra

Diol-1

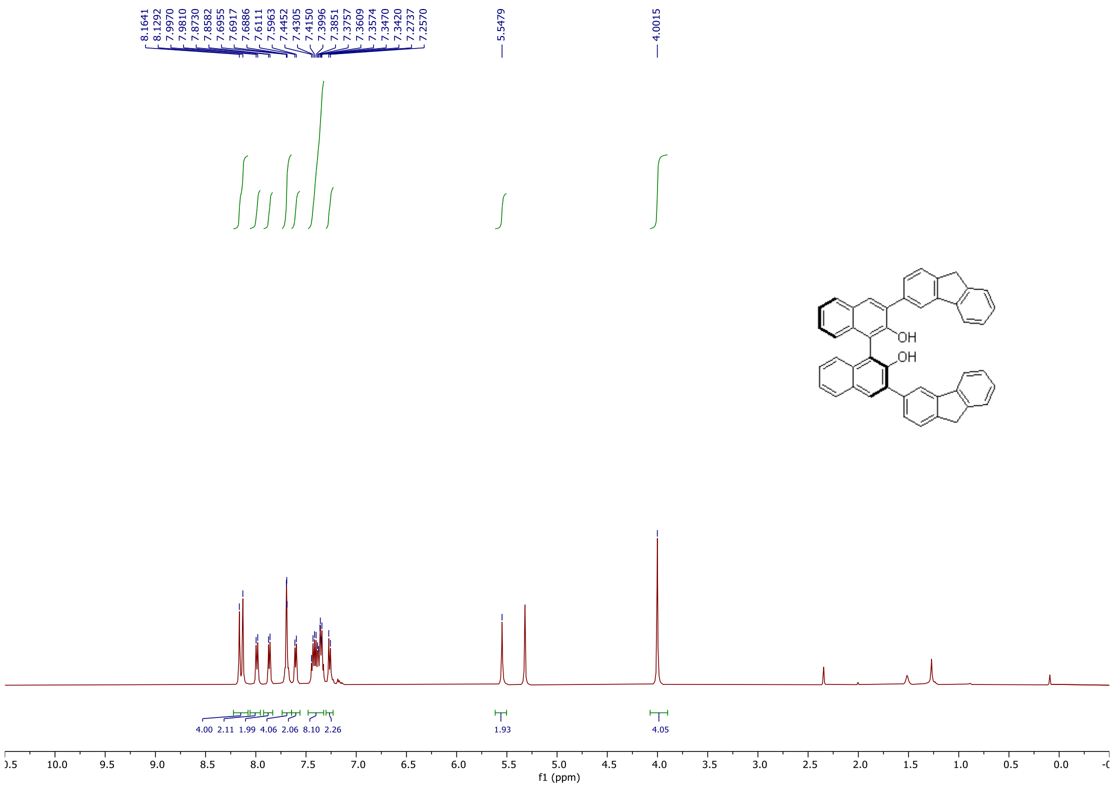

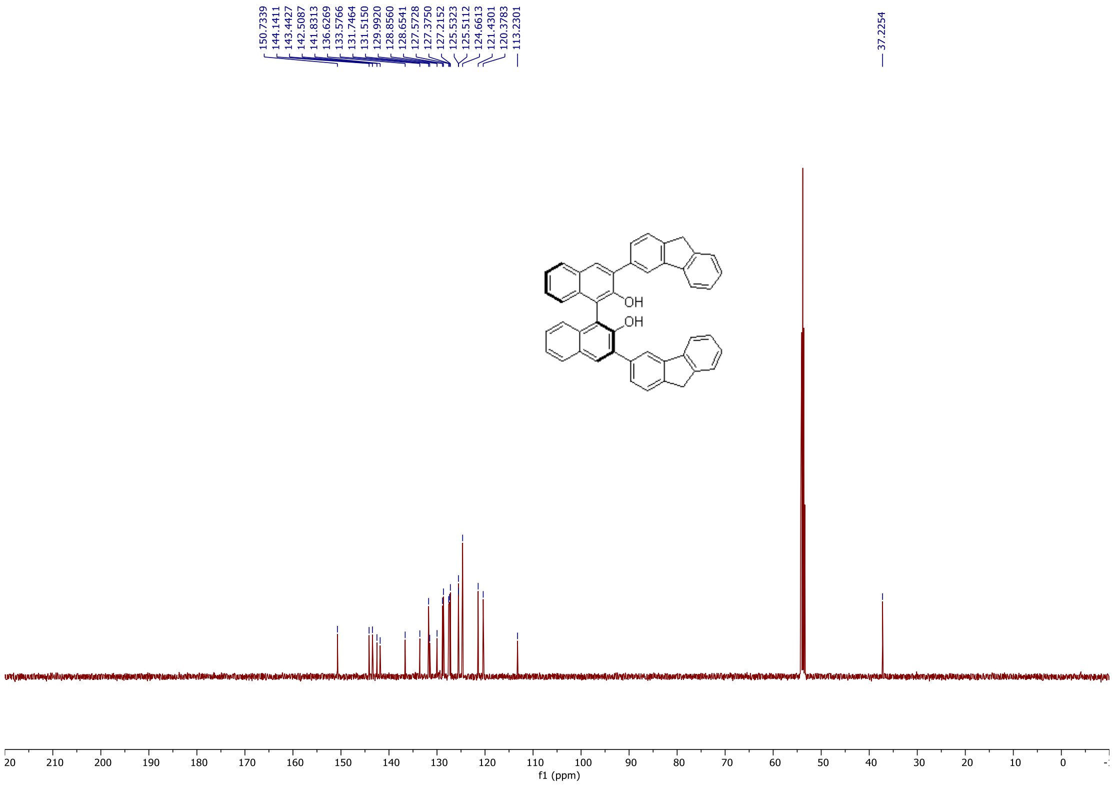

Diol-2

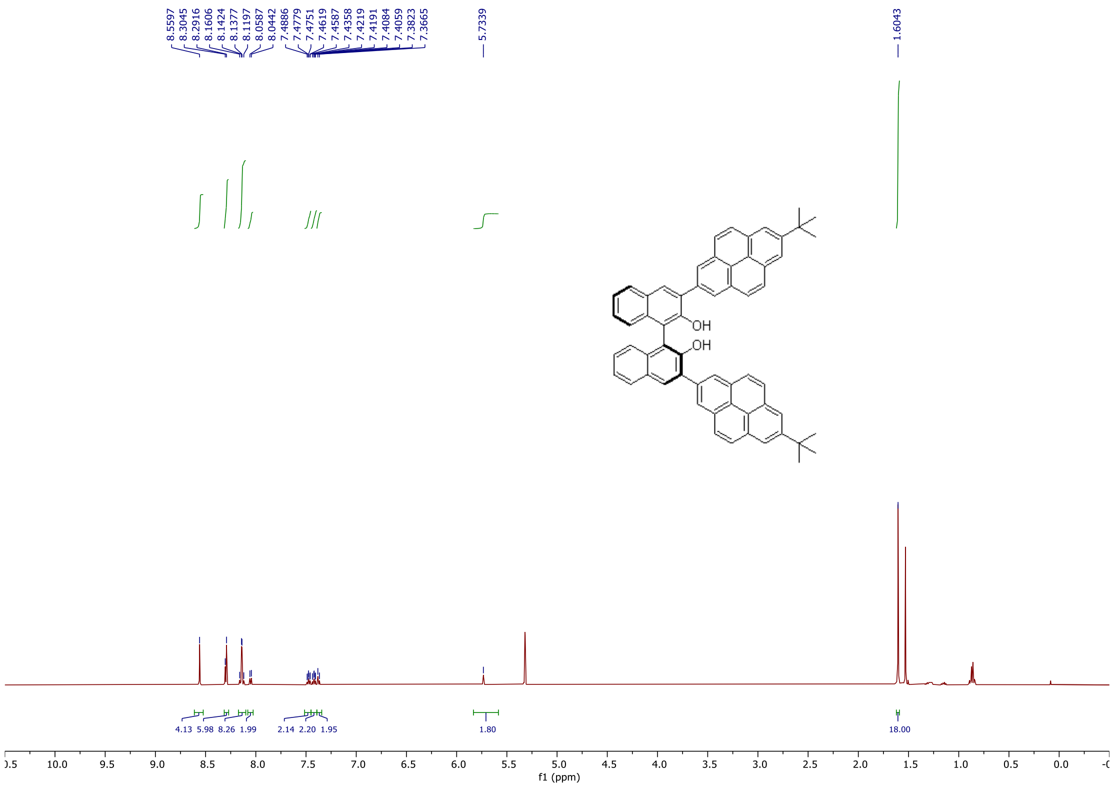

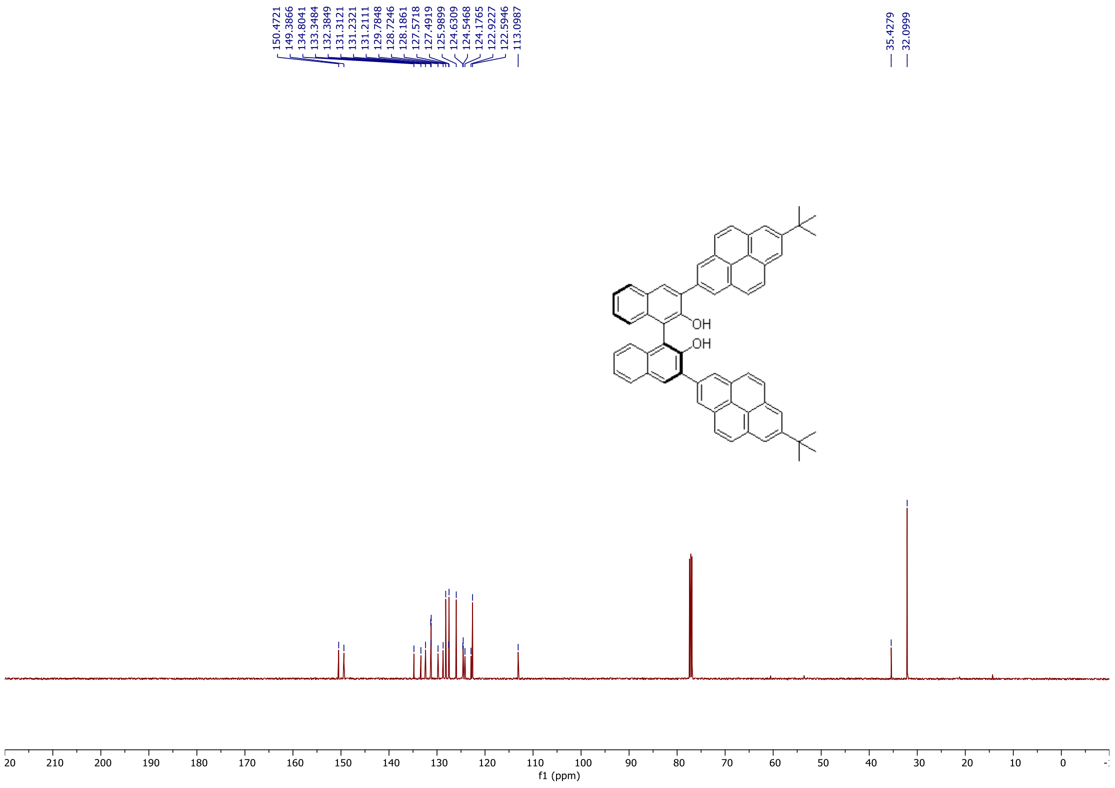

IDPi-1

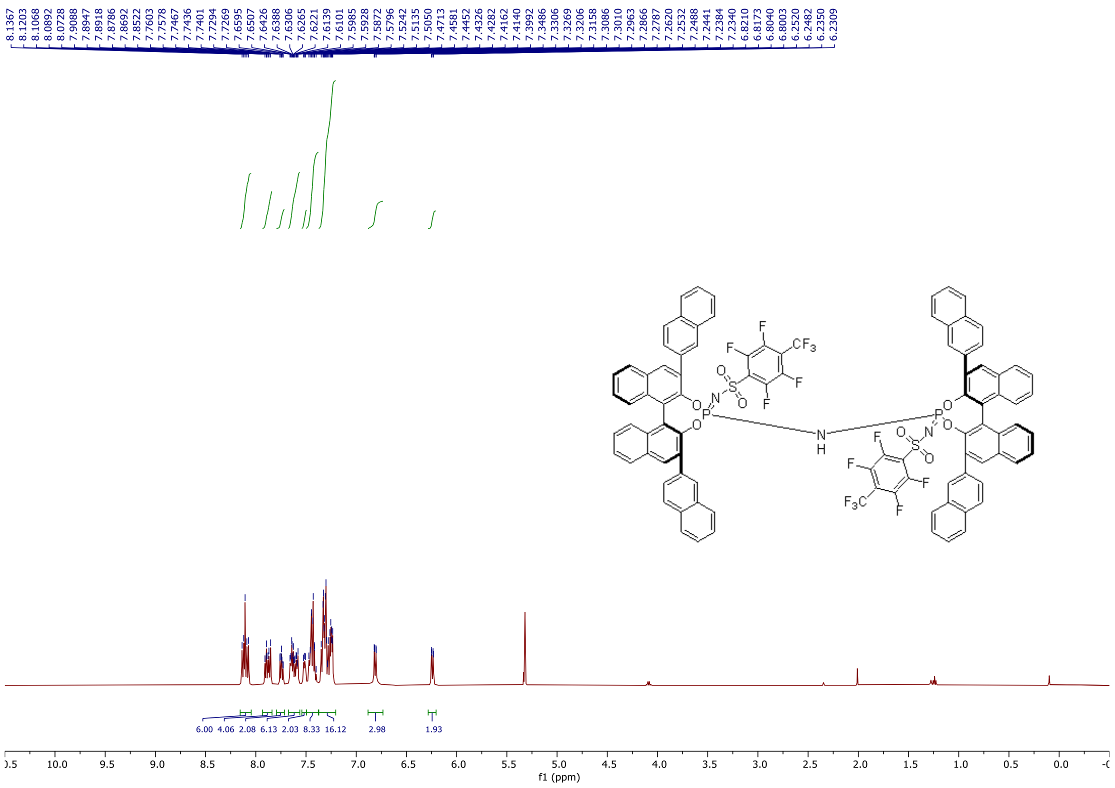
8.1367 8.1203 8.1068 8.0892 8.0728 7.9088 7.8947 7.8918 7.8786 7.8692 7.8522 7.7603 7.7578 7.7467 7.7436 7.7401 7.7294 7.7269 7.6595 7.6507 7.6426 7.6388 7.6306 7.6265 7.6221 7.6139 7.6101 7.5985 7.5928 7.5872 7.5796 7.5242 7.5135 7.5050 7.4713 7.4581 7.4452 7.4326 7.4282 7.4162 7.4140 7.3992 7.3486 7.3306 7.3269 7.3206 7.3158 7.3086 7.3010 7.2963 7.2866 7.2787 7.2620 7.2532 7.2488 7.2441 7.2384 7.2340 6.8210 6.8173 6.8040 6.8003 6.2520 6.2482 6.2350 6.2309
6.00 4.06 2.08 6.13 2.03 8.33 16.12 2.98 1.93
f1 (ppm)

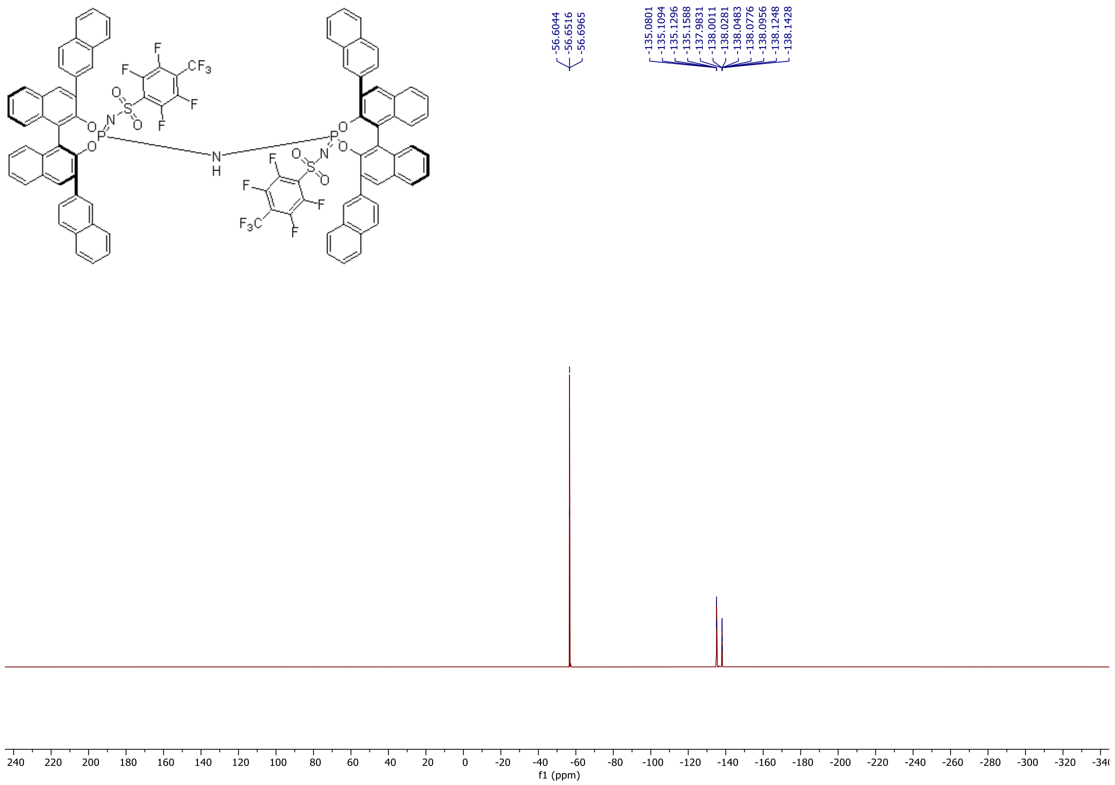
-56.6044 -56.6516 -56.6965
-135.0801 -135.1094 -135.1296 -135.1588 -137.9831 -138.0011 -138.0281 -138.0483 -138.0776 -138.0956 -138.1248 -138.1428
f1 (ppm)

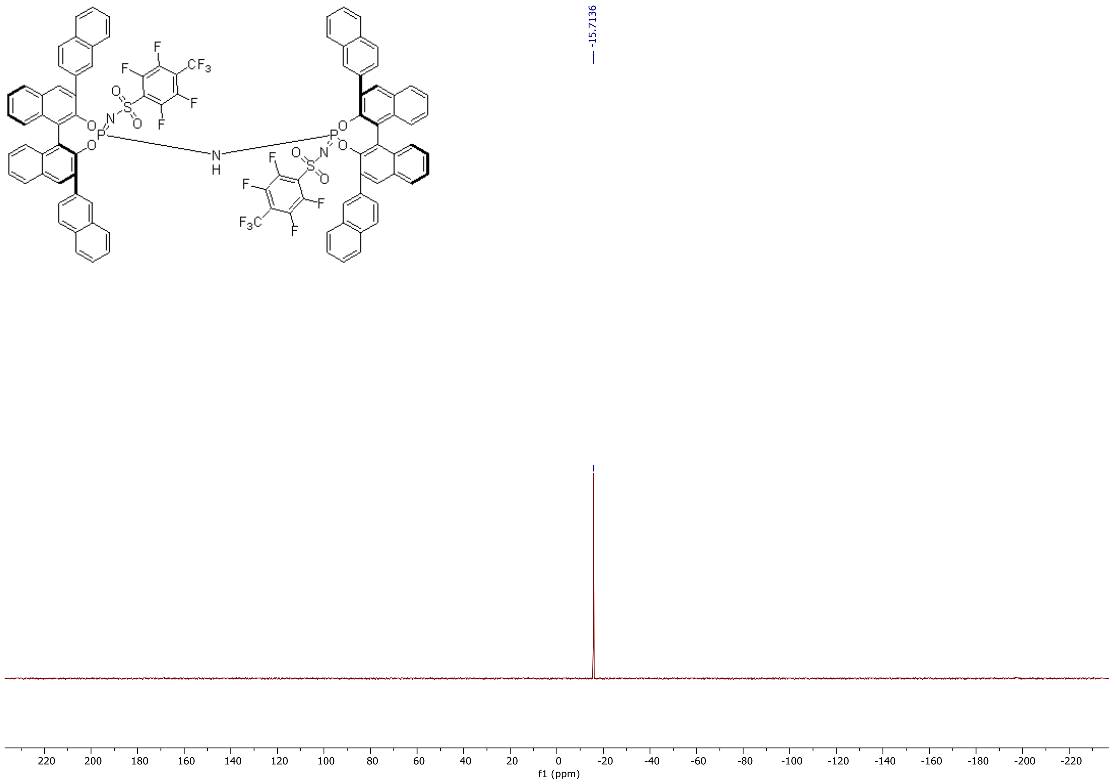

-15.7136
f1 (ppm)

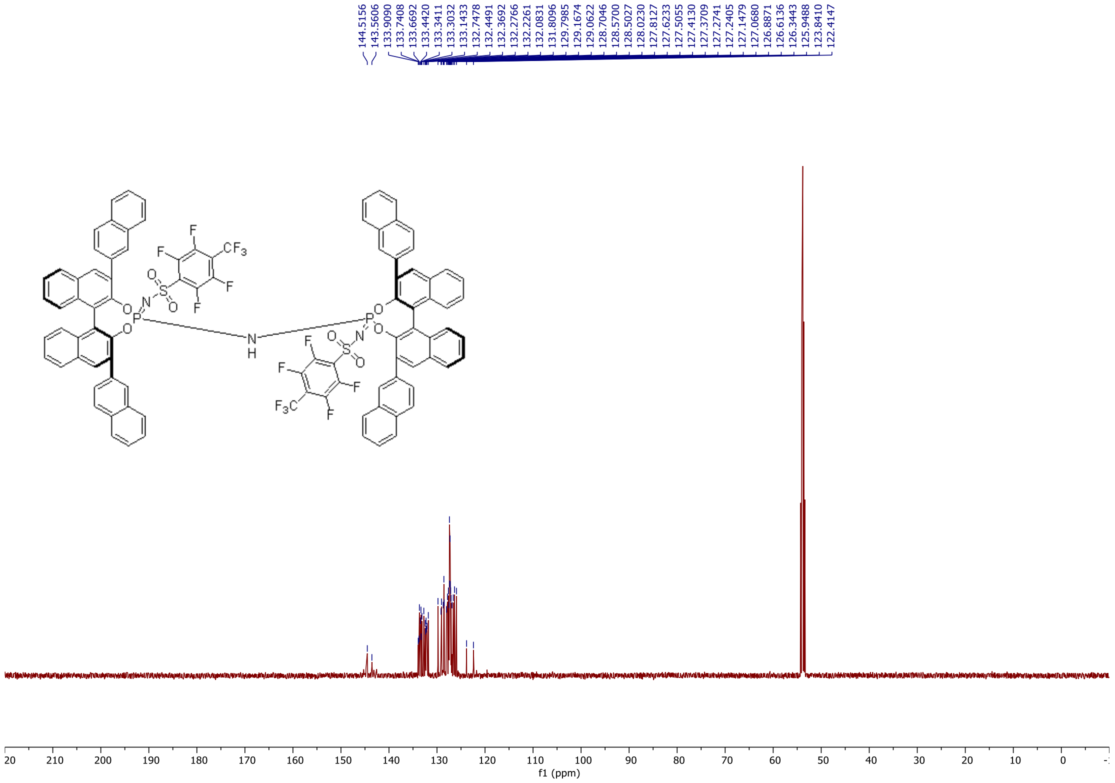

144.5156
143.5606
133.9090
133.7408
133.6692
133.4420
133.3411
133.3032
133.1433
132.7478
132.4491
132.3692
132.2766
132.2261
132.0831
131.8096
129.7985
129.1674
129.0622
128.7046
128.5700
128.5027
128.0230
127.8127
127.6233
127.5055
127.4130
127.3709
127.2741
127.2405
127.1479
127.0680
126.8871
126.6136
126.3443
125.9488
123.8410
122.4147
f1 (ppm)

IDPi-2

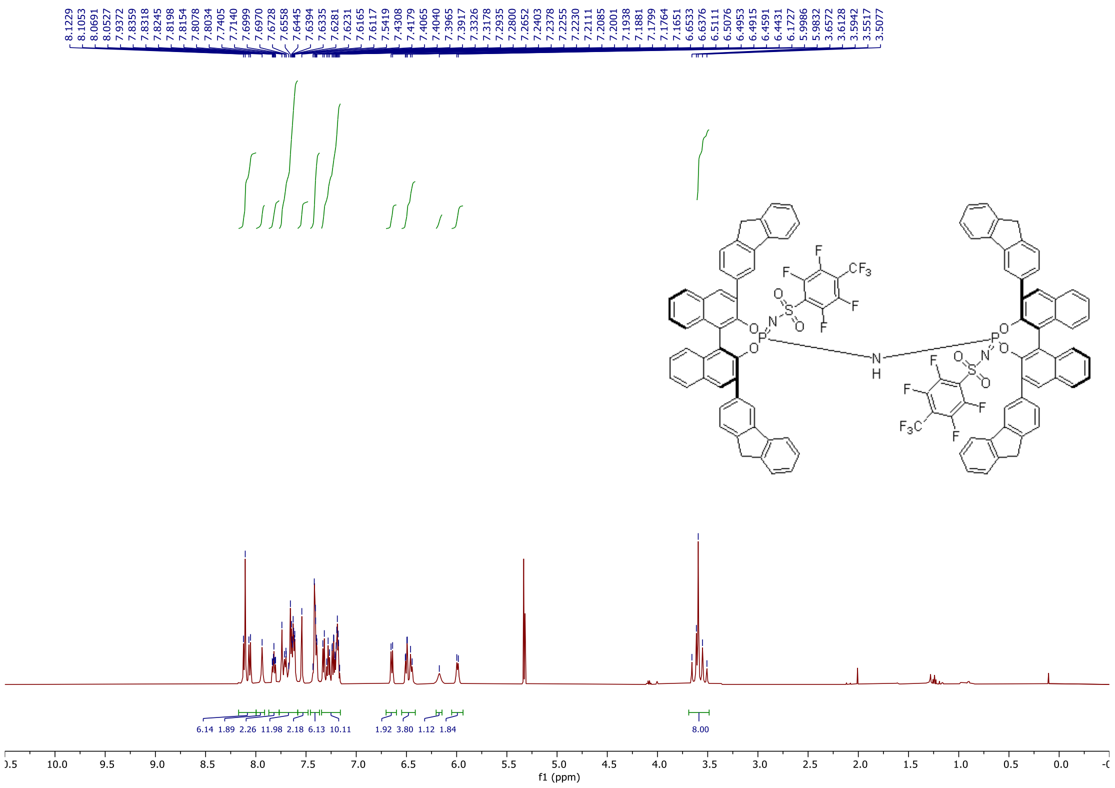
8.1229 8.1053 8.0691 8.0527 7.9372 7.8359 7.8318 7.8245 7.8198 7.8154 7.8078 7.8034 7.7405 7.7140 7.6999 7.6970 7.6728 7.6558 7.6445 7.6394 7.6335 7.6281 7.6231 7.6165 7.6117 7.5419 7.4308 7.4179 7.4065 7.4040 7.3965 7.3917 7.3326 7.3178 7.2935 7.2800 7.2652 7.2403 7.2378 7.2255 7.2230 7.2111 7.2085 7.2001 7.1938 7.1881 7.1799 7.1764 7.1651 6.6533 6.6376 6.5111 6.5076 6.4953 6.4915 6.4591 6.4431 6.1727 5.9986 5.9832 3.6572 3.6128 3.5942 3.5517 3.5077
6.14 1.89 2.26 11.98 2.18 6.13 10.11 1.92 3.80 1.12 1.84 8.00
f1 (ppm)

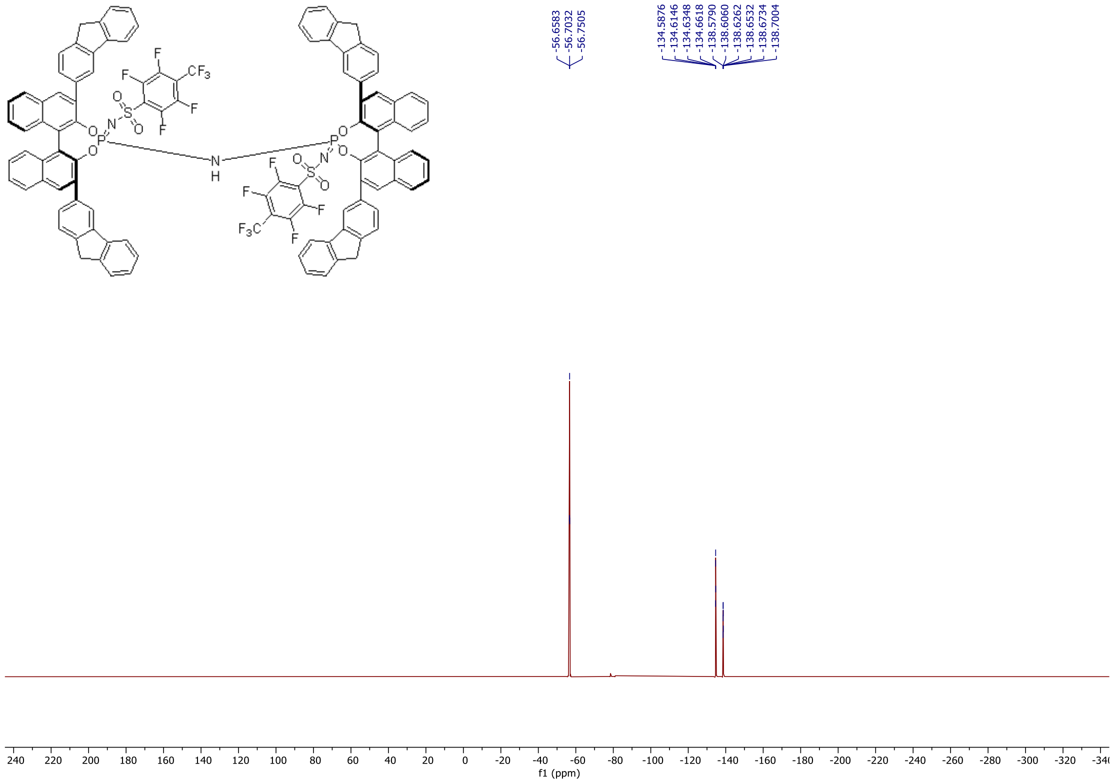
-56.6583 -56.7032 -56.7505
-134.5876 -134.6146 -134.6348 -134.6618 -138.5790 -138.6060 -138.6262 -138.6532 -138.6734 -138.7004
f1 (ppm)

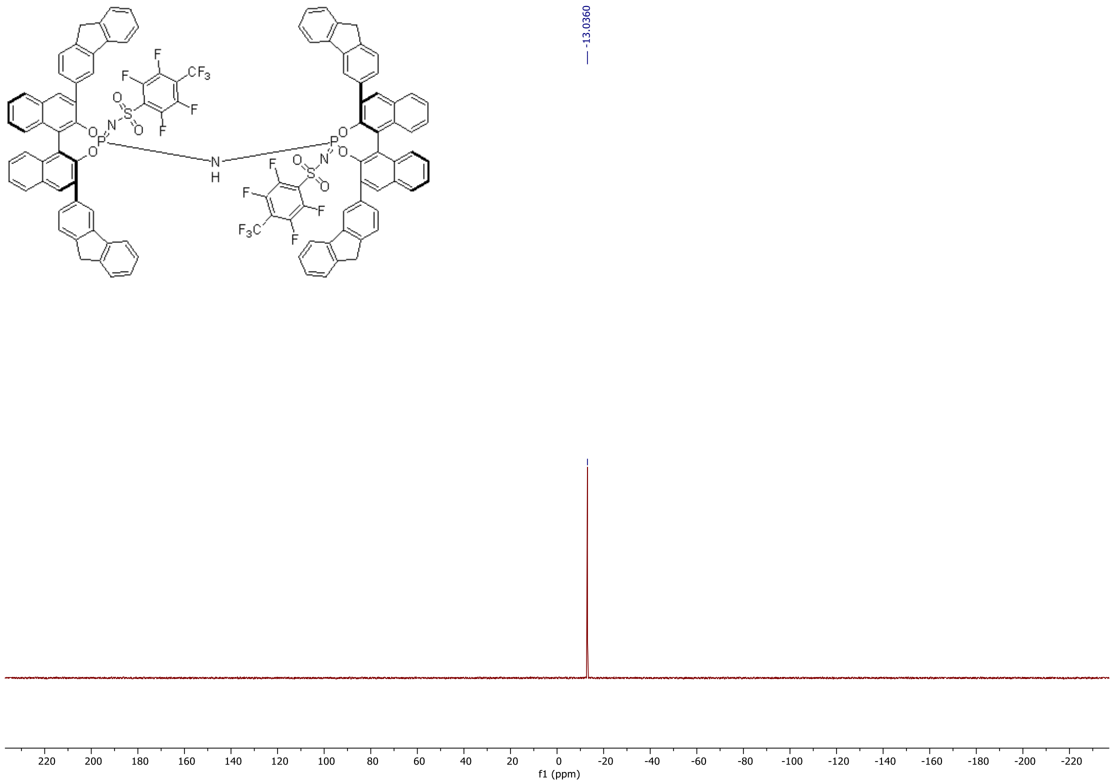

-13.0360
f1 (ppm)

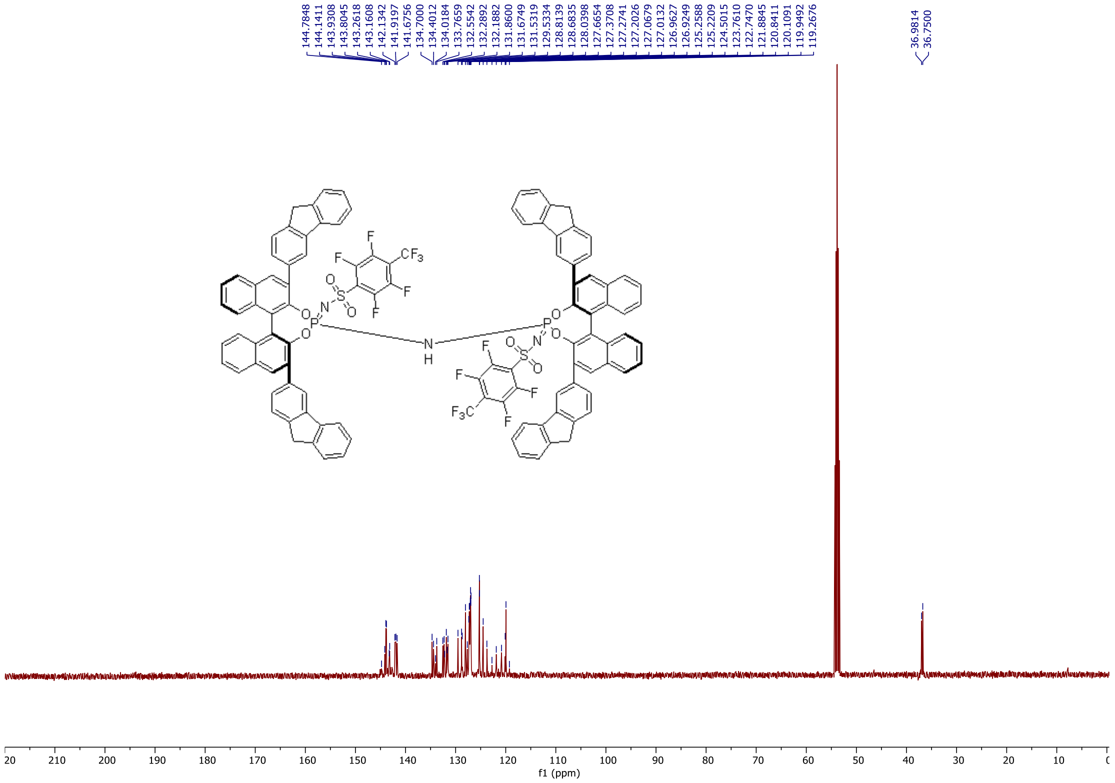

144.7848
144.1411
143.9308
143.8045
143.2618
143.1608
142.1342
141.9197
141.6756
134.7000
134.4012
134.0184
133.7659
132.5542
132.2892
132.1882
131.8600
131.6749
131.5319
129.5334
128.8139
128.6835
128.0398
127.6654
127.3708
127.2741
127.2026
127.0679
127.0132
126.9627
126.9249
125.2588
125.2209
124.5015
123.7610
122.7470
121.8845
120.8411
120.1091
119.9492
119.2676
36.9814
36.7500
f1 (ppm)

IDPi-3

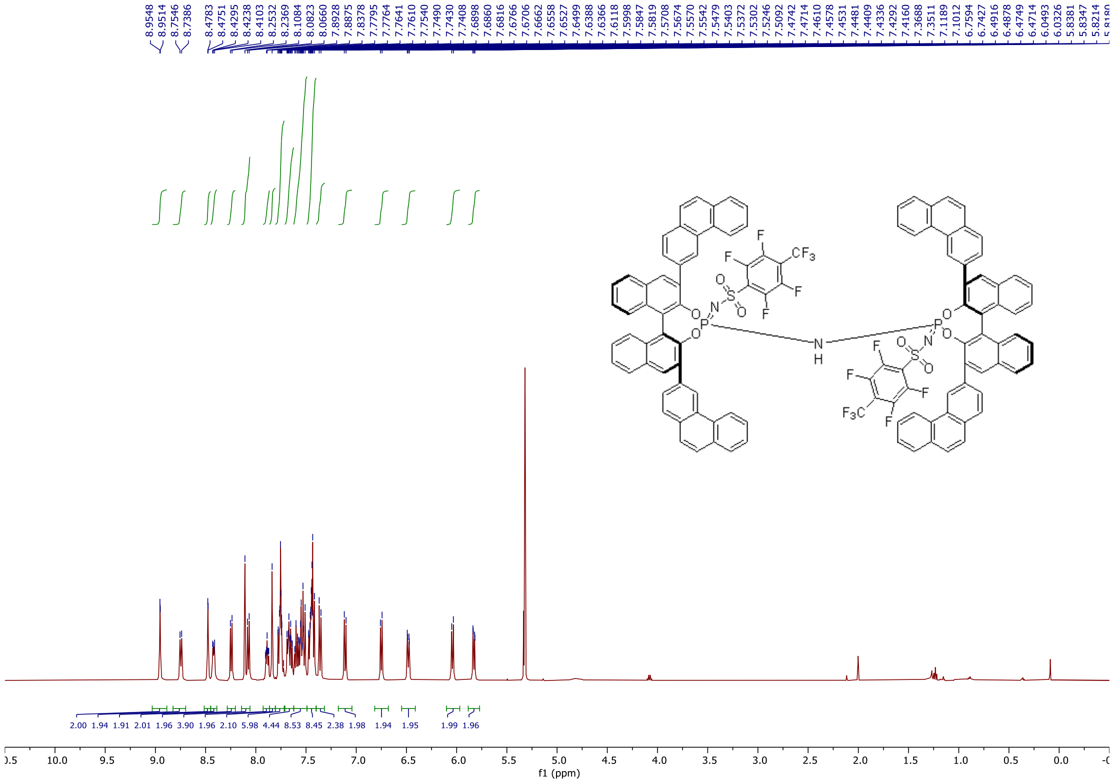
8.9548
8.9514
8.7546
8.7386
8.4783
8.4751
8.4295
8.4238
8.4103
8.2532
8.2369
8.1084
8.0823
8.0660
7.8928
7.8875
7.8378
7.7795
7.7764
7.7641
7.7610
7.7540
7.7490
7.7430
7.7408
7.6898
7.6860
7.6816
7.6766
7.6706
7.6662
7.6558
7.6527
7.6499
7.6388
7.6366
7.6118
7.5998
7.5847
7.5819
7.5708
7.5674
7.5570
7.5542
7.5479
7.5403
7.5372
7.5302
7.5246
7.5092
7.4742
7.4714
7.4610
7.4578
7.4531
7.4481
7.4409
7.4336
7.4292
7.4160
7.3688
7.3511
7.1189
7.1012
6.7594
6.7427
6.4916
6.4878
6.4749
6.4714
6.0493
6.0326
5.8381
5.8347
5.8214
2.00 1.94 1.91 2.01 1.96 3.90 1.96 2.10 5.98 4.44 8.53 8.45 2.38 1.98 1.94 1.95 1.99 1.96
f1 (ppm)

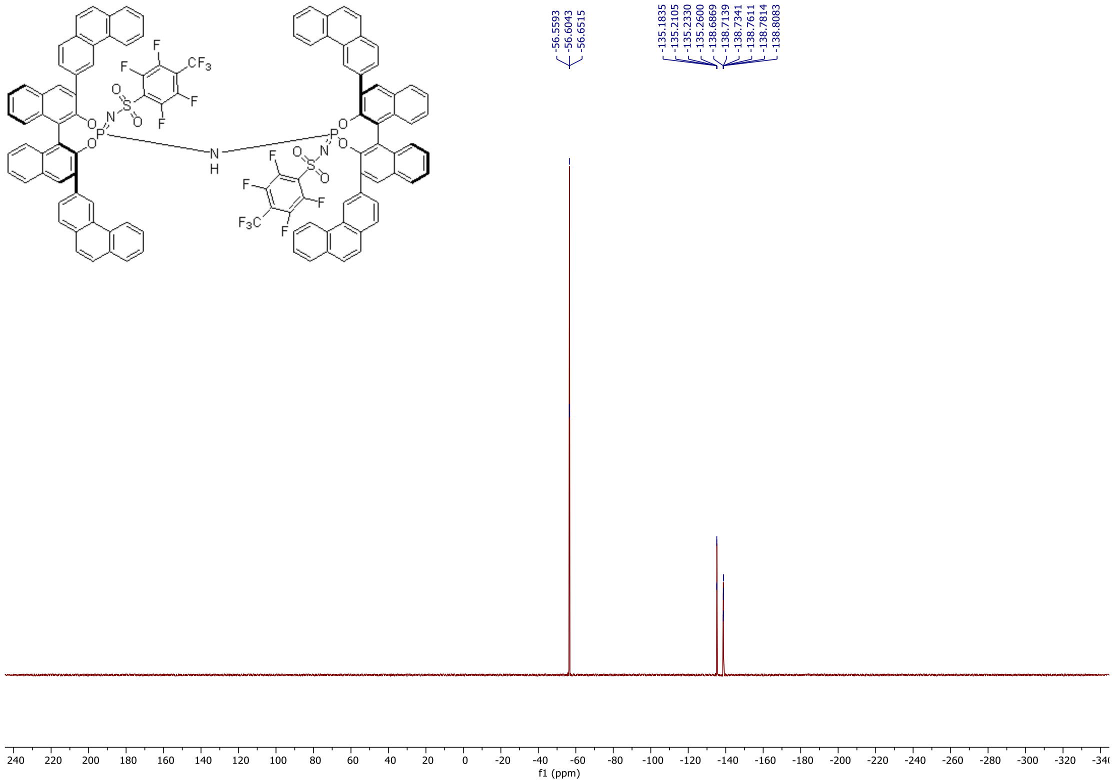
-56.5593
-56.6043
-56.6515
-135.1835
-135.2105
-135.2330
-135.2600
-138.6869
-138.7139
-138.7341
-138.7611
-138.7814
-138.8083
f1 (ppm)

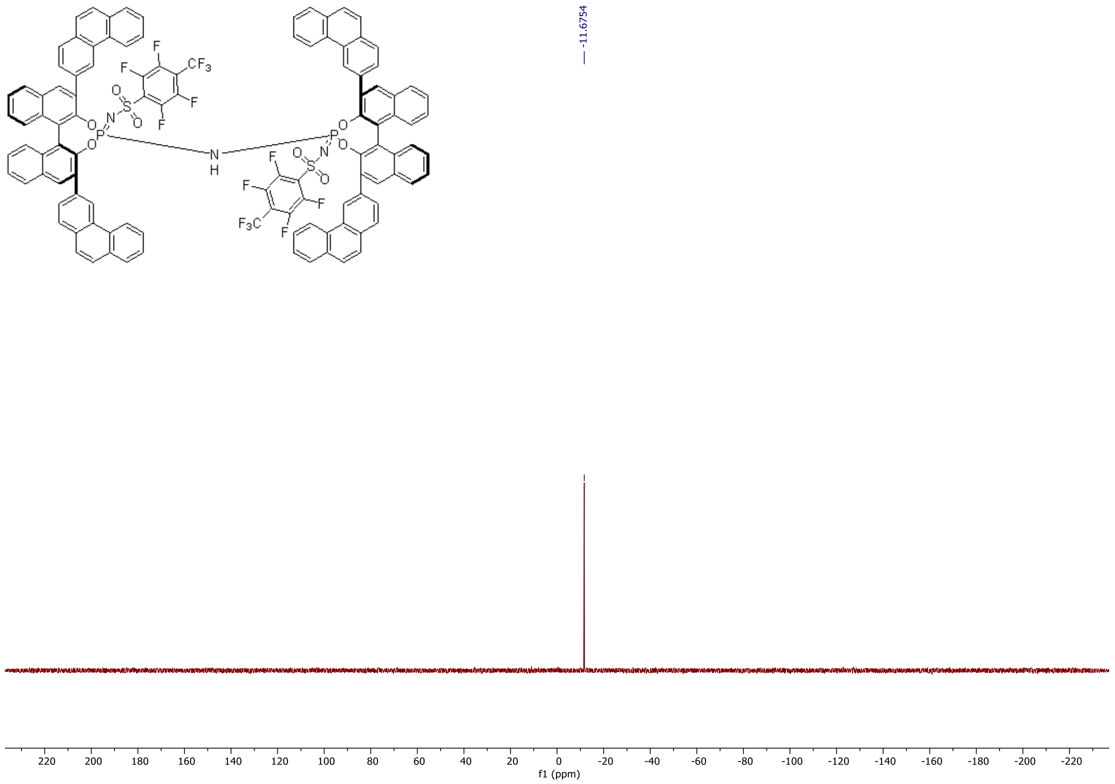
-11.6754
220 200 180 160 140 120 100 80 60 40 20 0 -20 -40 -60 -80 -100 -120 -140 -160 -180 -200 -220
f1 (ppm)

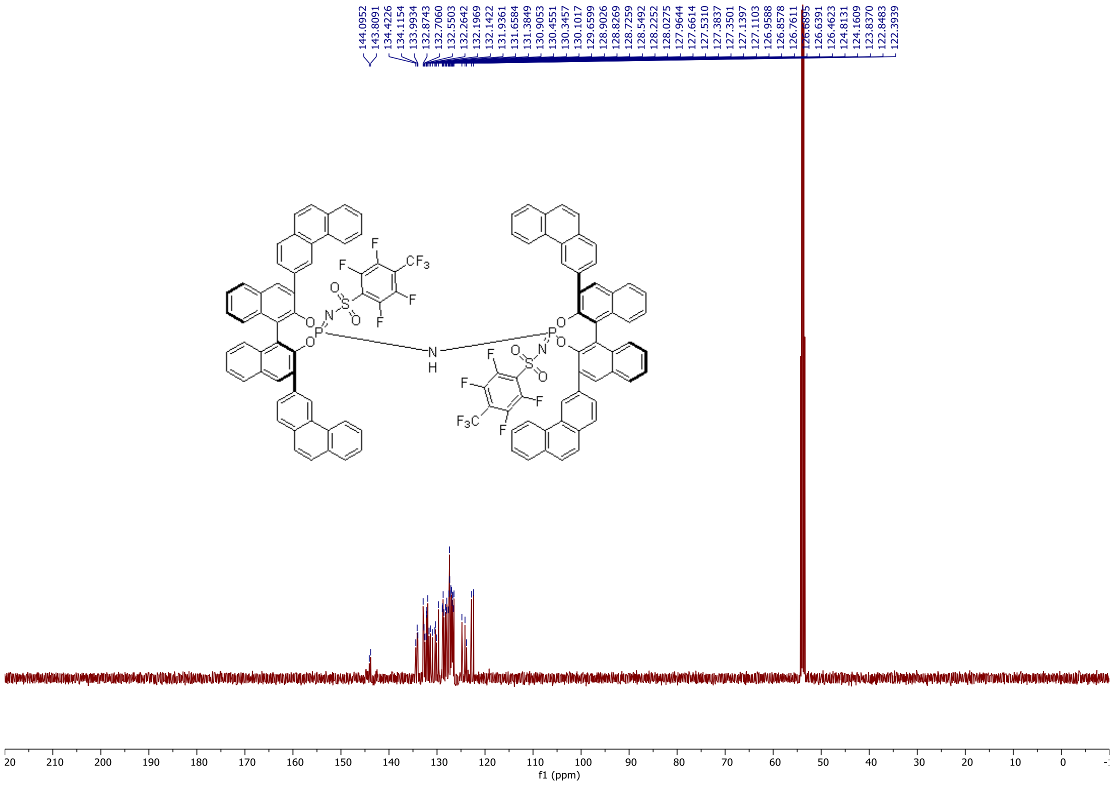
144.0952
143.8091
134.4226
134.1154
133.9934
132.8743
132.7060
132.5503
132.2642
132.1969
132.1422
131.9361
131.6584
131.3849
130.9053
130.4551
130.3457
130.1017
129.6599
128.9026
128.8269
128.7259
128.5492
128.2252
128.0275
127.9644
127.6614
127.5310
127.3837
127.3501
127.1397
127.1103
126.9588
126.8578
126.7611
126.6895
126.6391
126.4623
124.8131
124.1609
123.8370
122.8483
122.3939
220 210 200 190 180 170 160 150 140 130 120 110 100 90 80 70 60 50 40 30 20 10 0
f1 (ppm)

IDPi-4

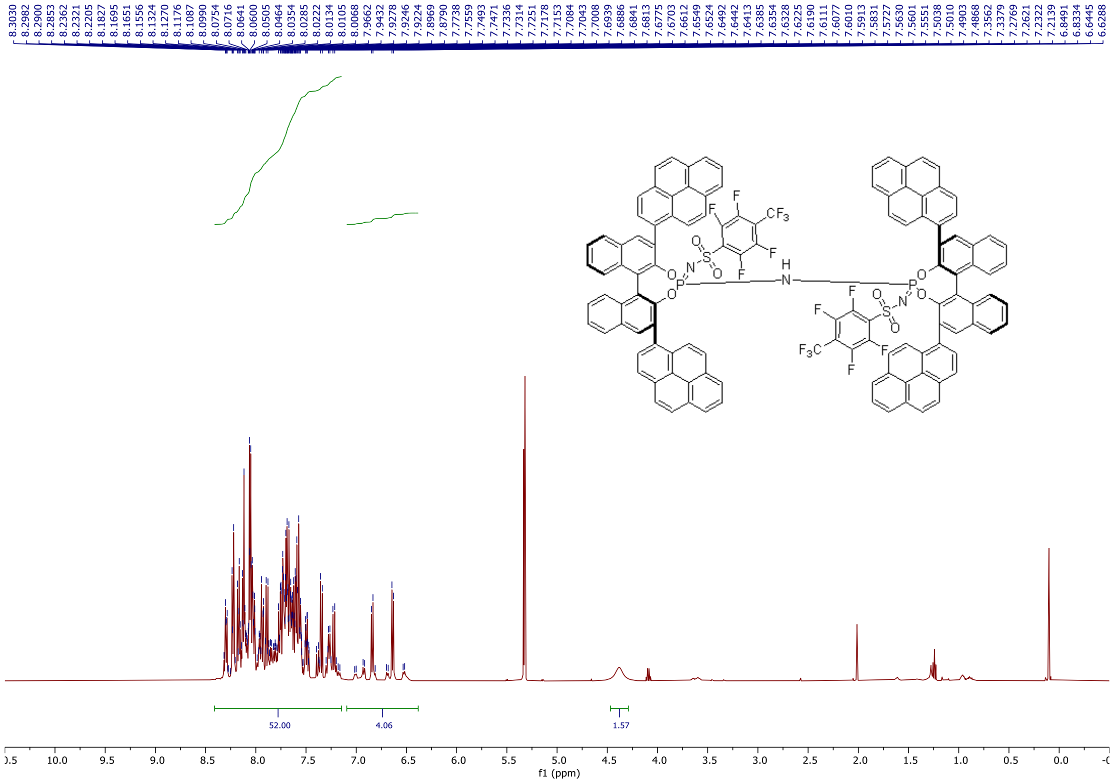

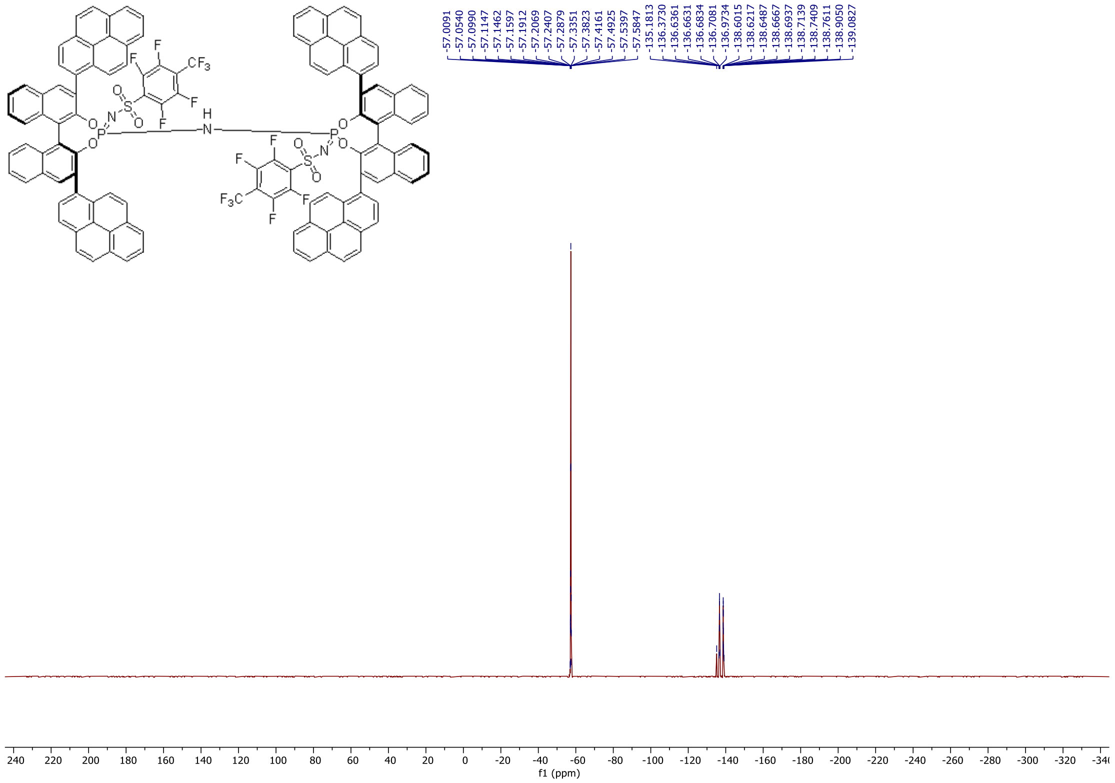

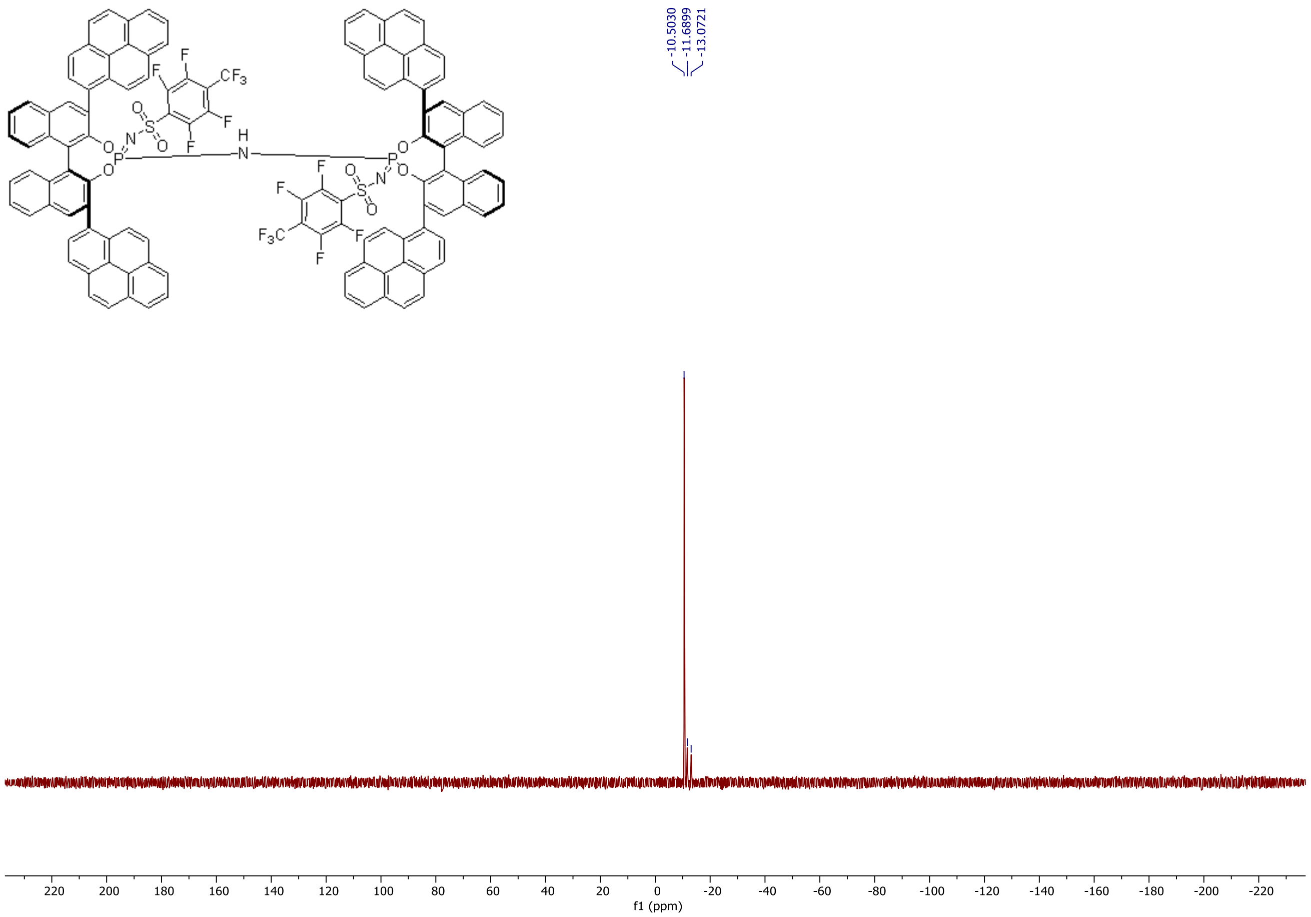
-10.5030
-11.6899
-13.0721
f1 (ppm)

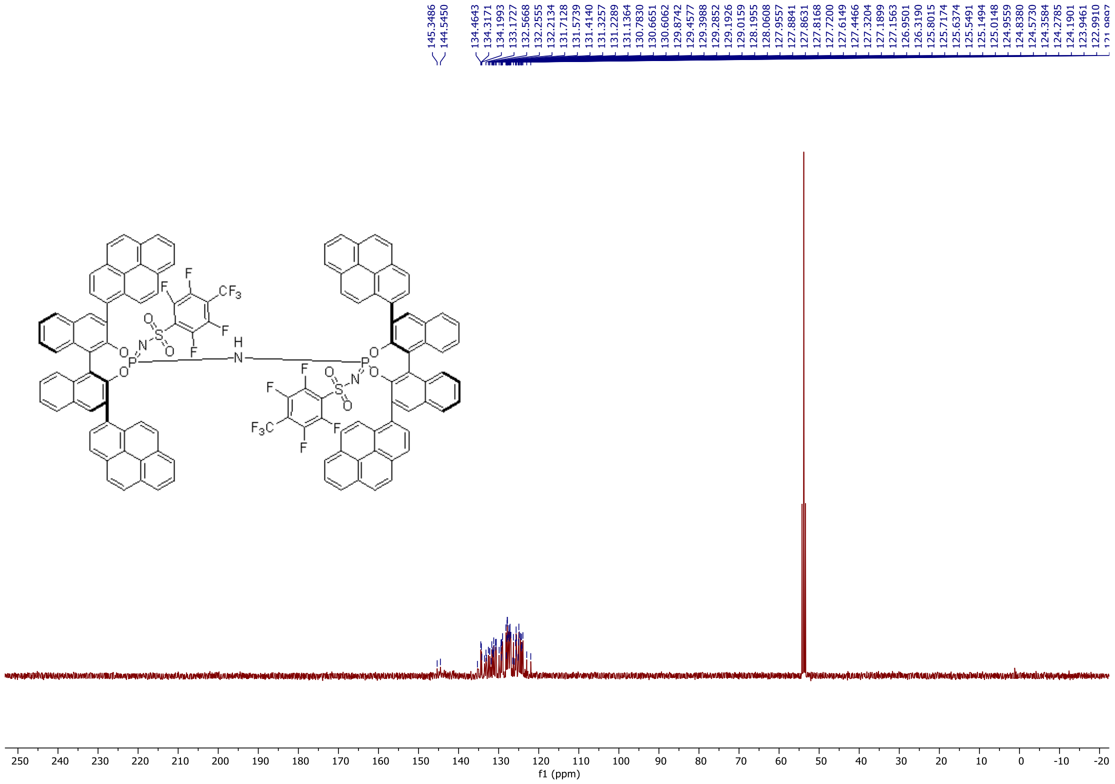
145.3486
144.5450
134.4643
134.3171
134.1993
133.1727
132.5668
132.2555
132.2134
131.7128
131.5739
131.4140
131.3257
131.2289
131.1364
130.7830
130.6651
130.6062
129.8742
129.4577
129.3988
129.2852
129.1926
129.0159
128.1955
128.0608
127.9557
127.8841
127.8631
127.8168
127.7200
127.6149
127.4466
127.3204
127.1899
127.1563
126.9501
126.3190
125.8015
125.7174
125.6374
125.5491
125.1494
125.0148
124.9559
124.8380
124.5730
124.3584
124.2785
124.1901
123.9461
122.9910
f1 (ppm)

IDPi-6

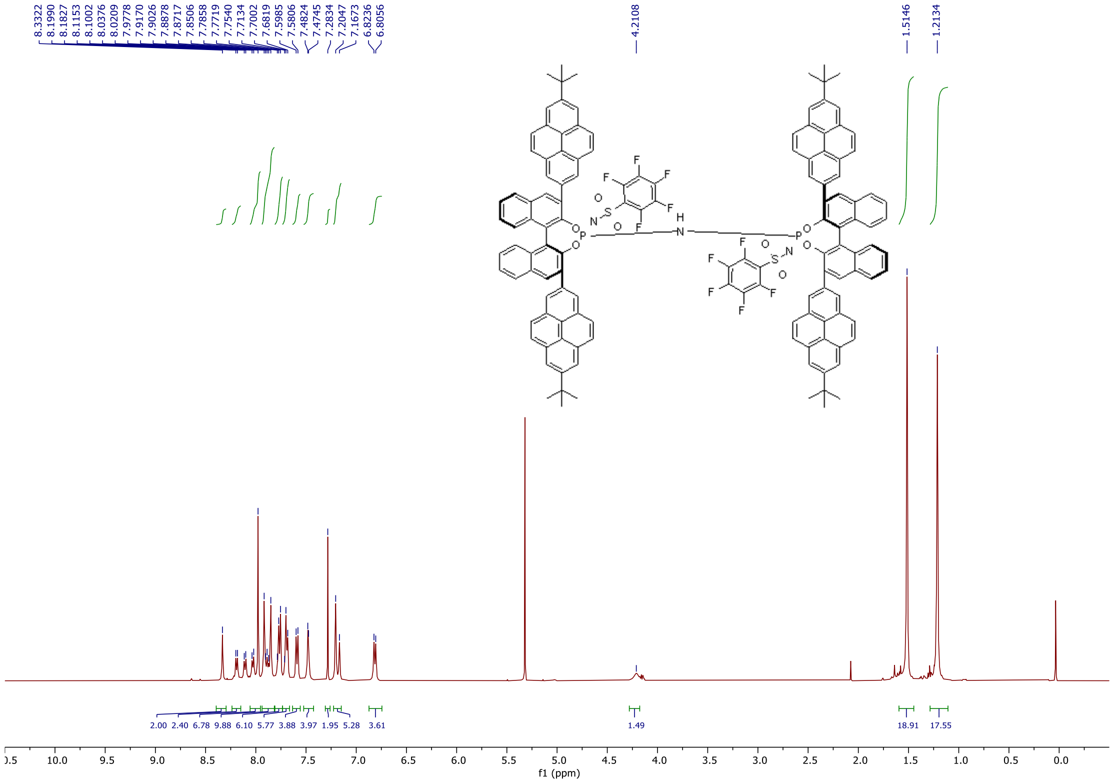
8.3322
8.1990
8.1827
8.1153
8.1002
8.0376
8.0209
7.9778
7.9170
7.9026
7.8878
7.8717
7.8506
7.7858
7.7719
7.7540
7.7134
7.7002
7.6819
7.5985
7.5806
7.4824
7.4745
7.2834
7.2047
7.1673
6.8236
6.8056
4.2108
1.5146
1.2134
2.00 2.40 6.78 9.88 6.10 5.77 3.88 3.97 1.95 5.28 3.61
1.49
18.91 17.55
f1 (ppm)

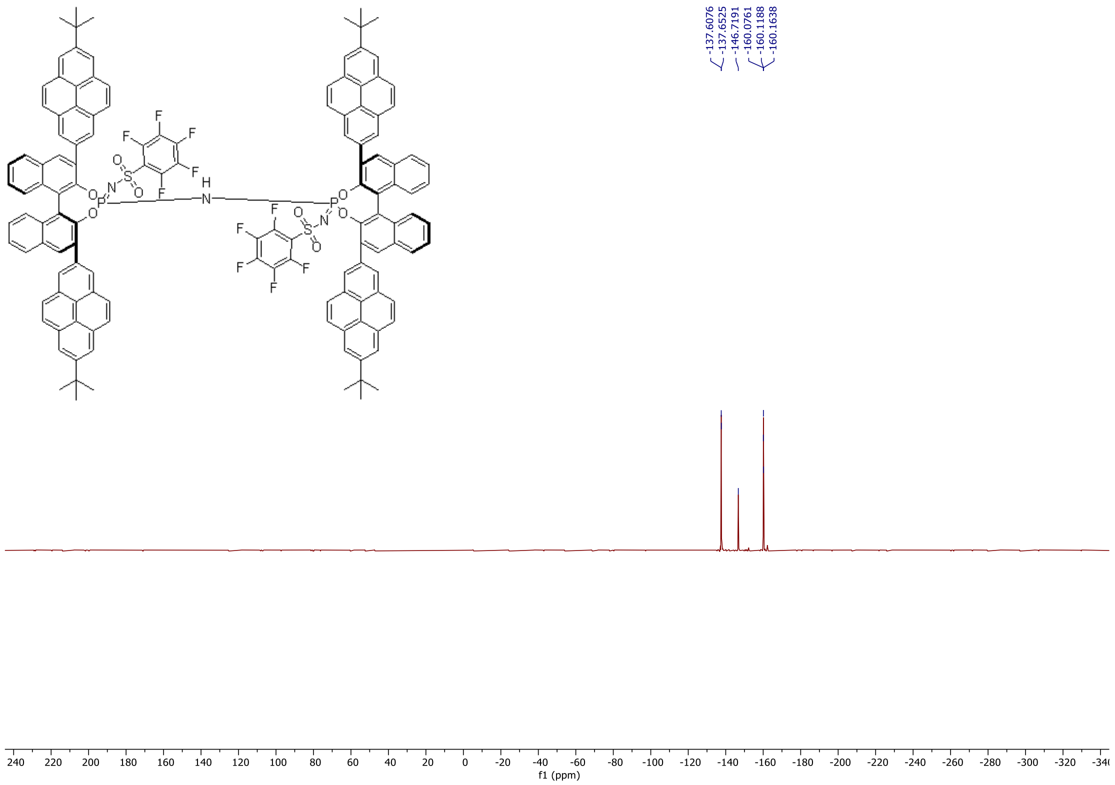
-137.6076
-137.6525
-146.7191
-160.0761
-160.1188
-160.1638
f1 (ppm)

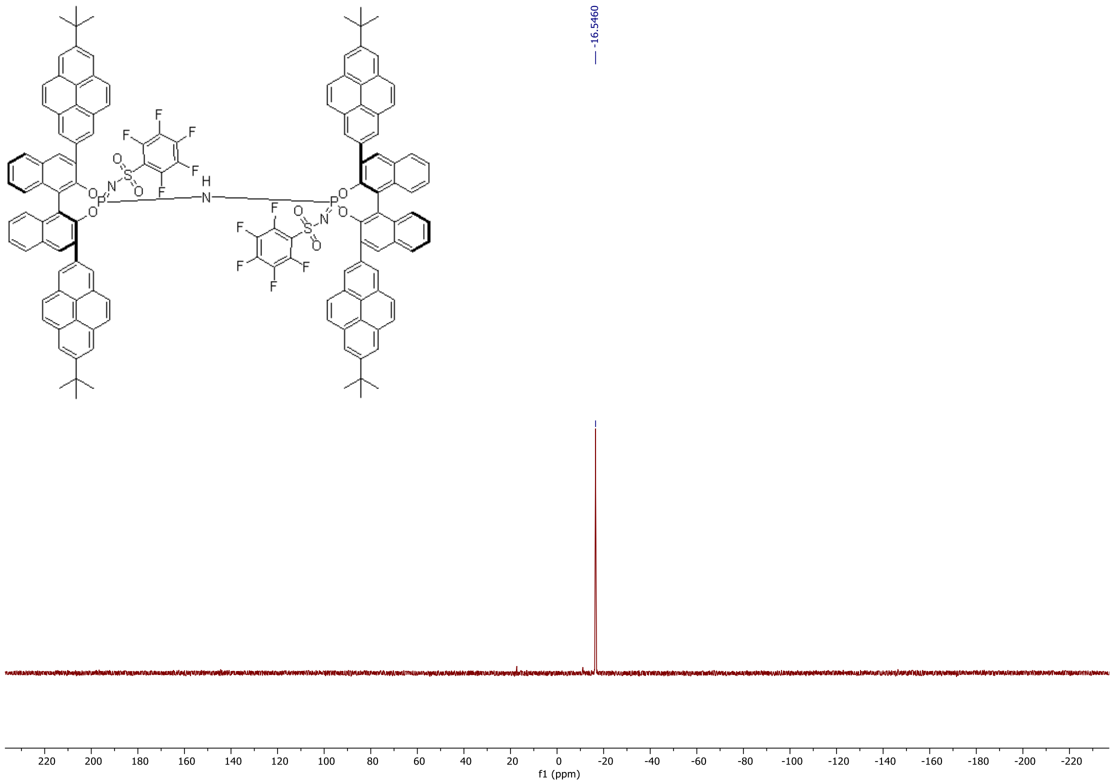
-16.5460
f1 (ppm)

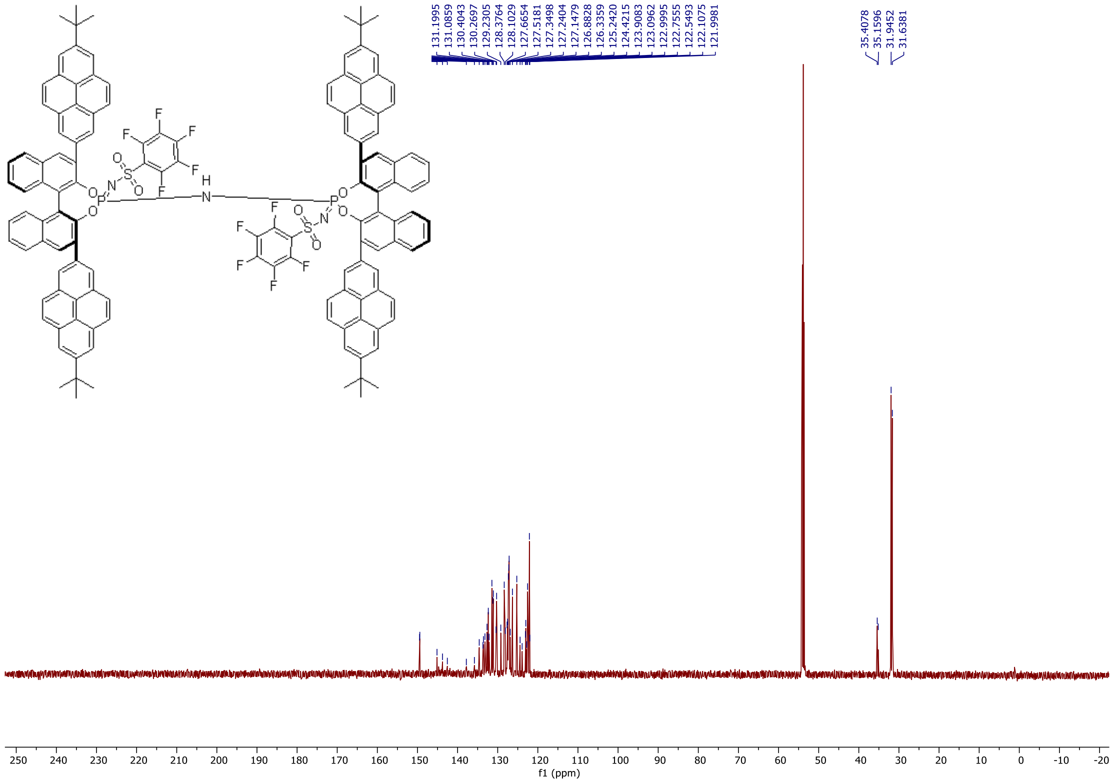
131.1995
131.0859
130.4043
130.2697
129.2305
128.3764
128.1029
127.6654
127.5181
127.3498
127.2404
127.1479
126.8828
126.3359
125.2420
124.4215
123.9083
123.0962
122.9995
122.7555
122.5493
122.1075
121.9981
35.4078
35.1596
31.9452
31.6381
f1 (ppm)

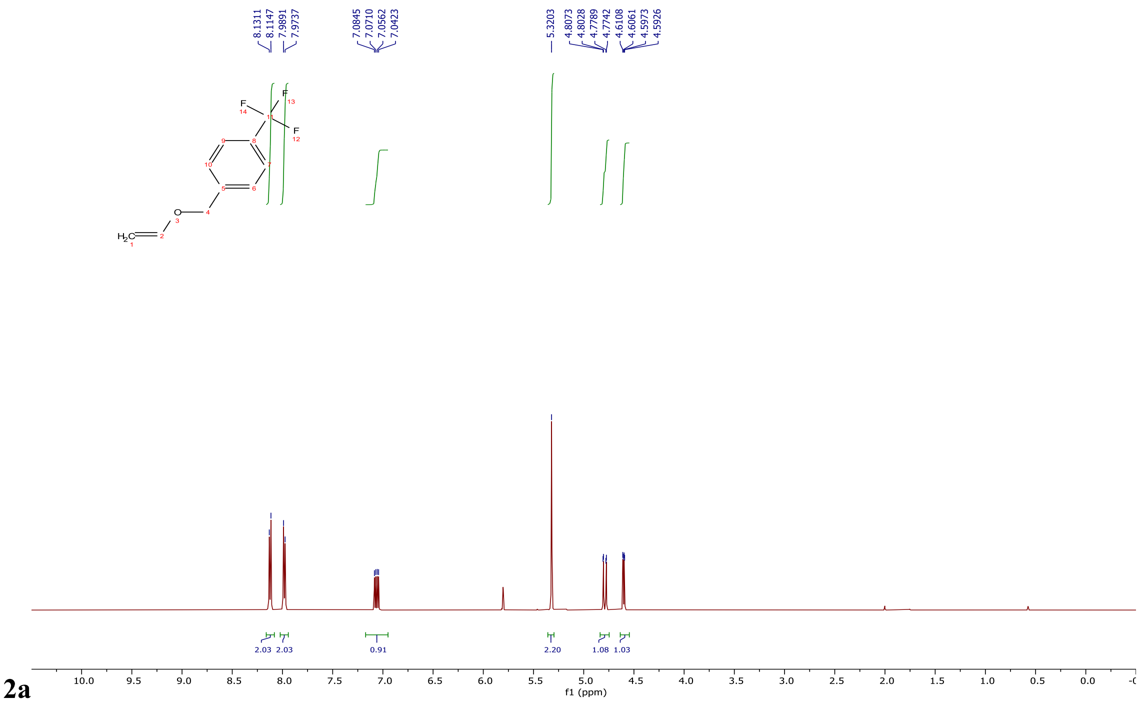

2a

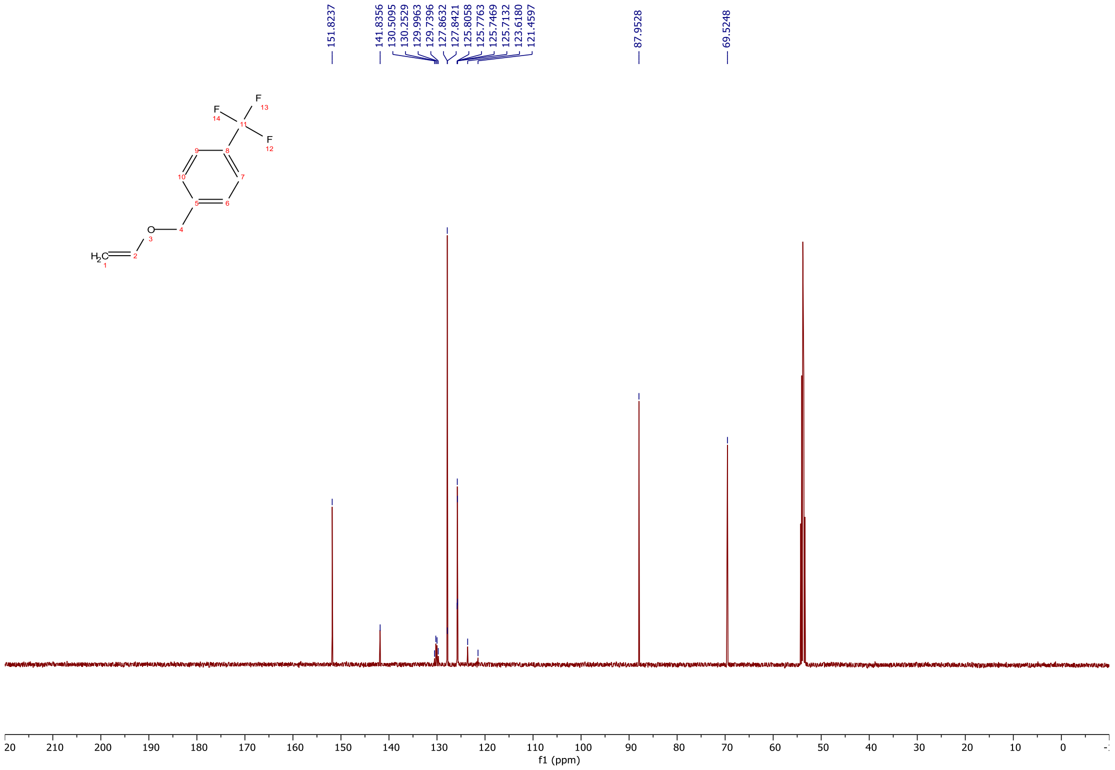

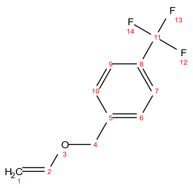

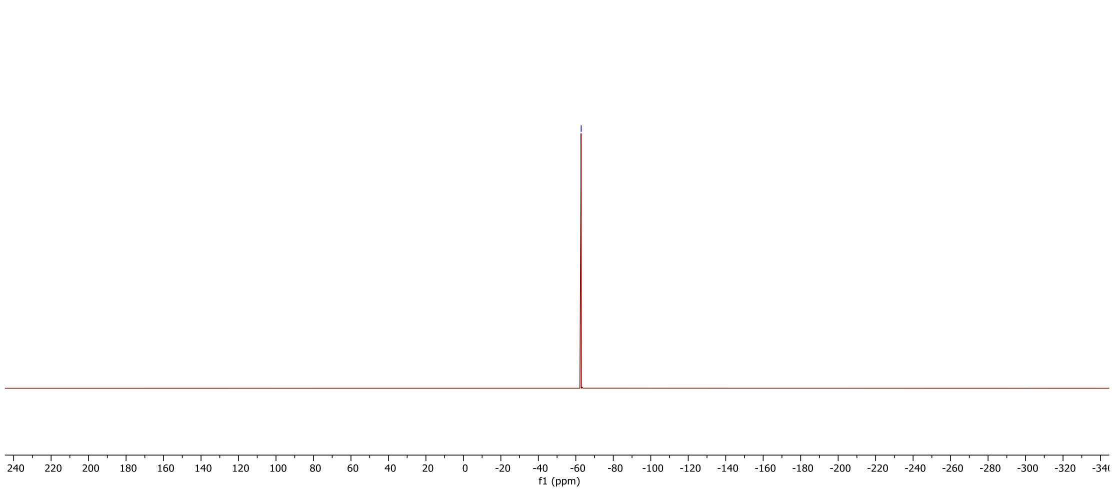
-62.8601
240 220 200 180 160 140 120 100 80 60 40 20 0 -20 -40 -60 -80 -100 -120 -140 -160 -180 -200 -220 -240 -260 -280 -300 -320
f1 (ppm)

2q

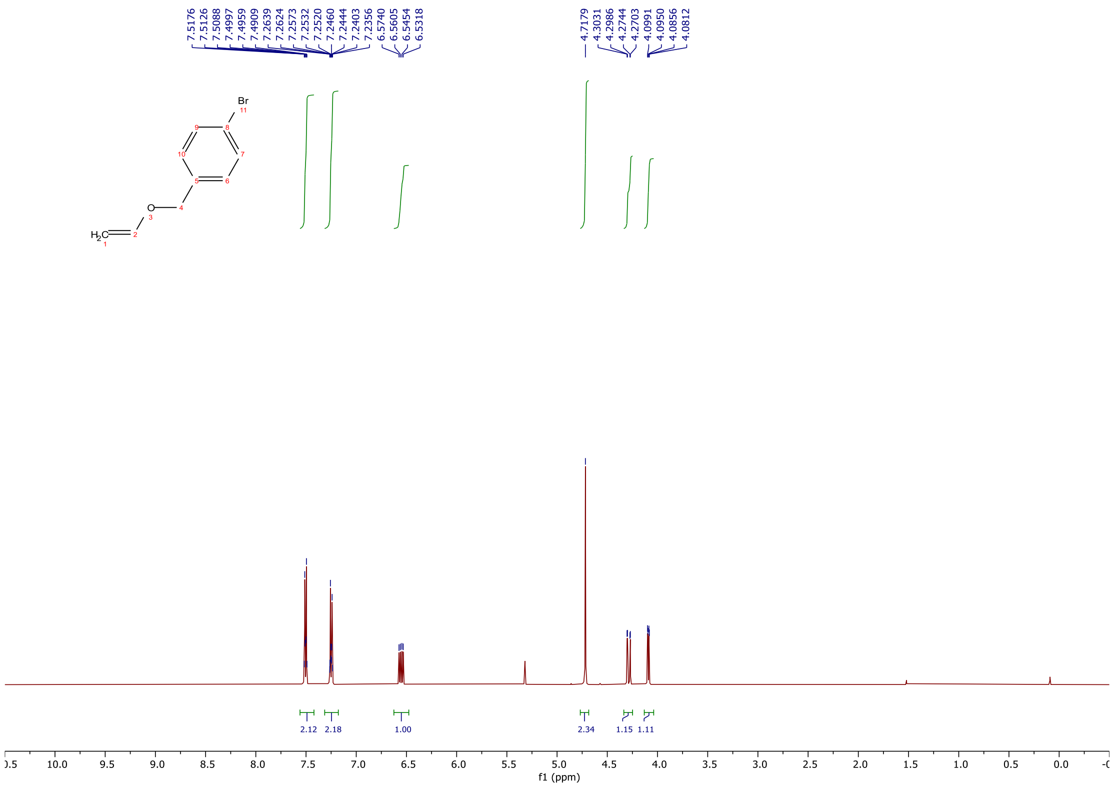
7.5176
7.5126
7.5088
7.4997
7.4959
7.4909
7.2639
7.2624
7.2573
7.2532
7.2520
7.2460
7.2444
7.2403
7.2356
6.5740
6.5605
6.5454
6.5318
4.7179
4.3031
4.2986
4.2744
4.2703
4.0991
4.0950
4.0856
4.0812
Br
2.12
2.18
1.00
2.34
1.15
1.11
f1 (ppm)

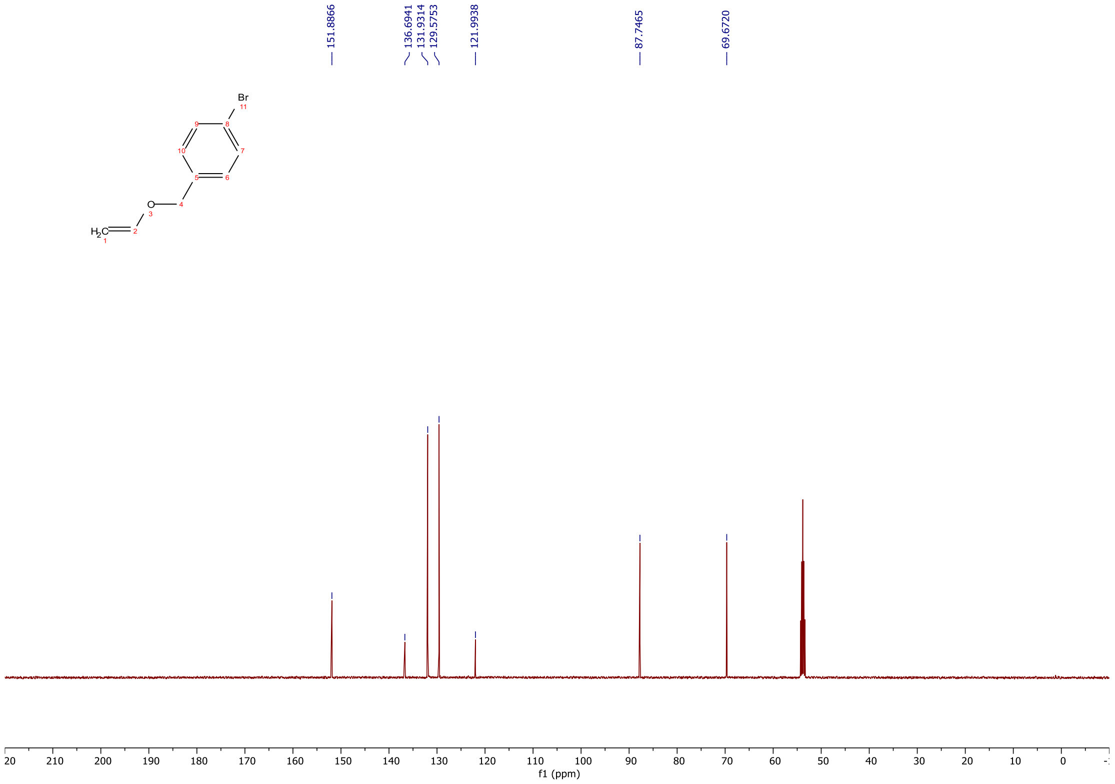
151.8866
136.6941
131.9314
129.5753
121.9938
87.7465
69.6720
Br
f1 (ppm)

2r

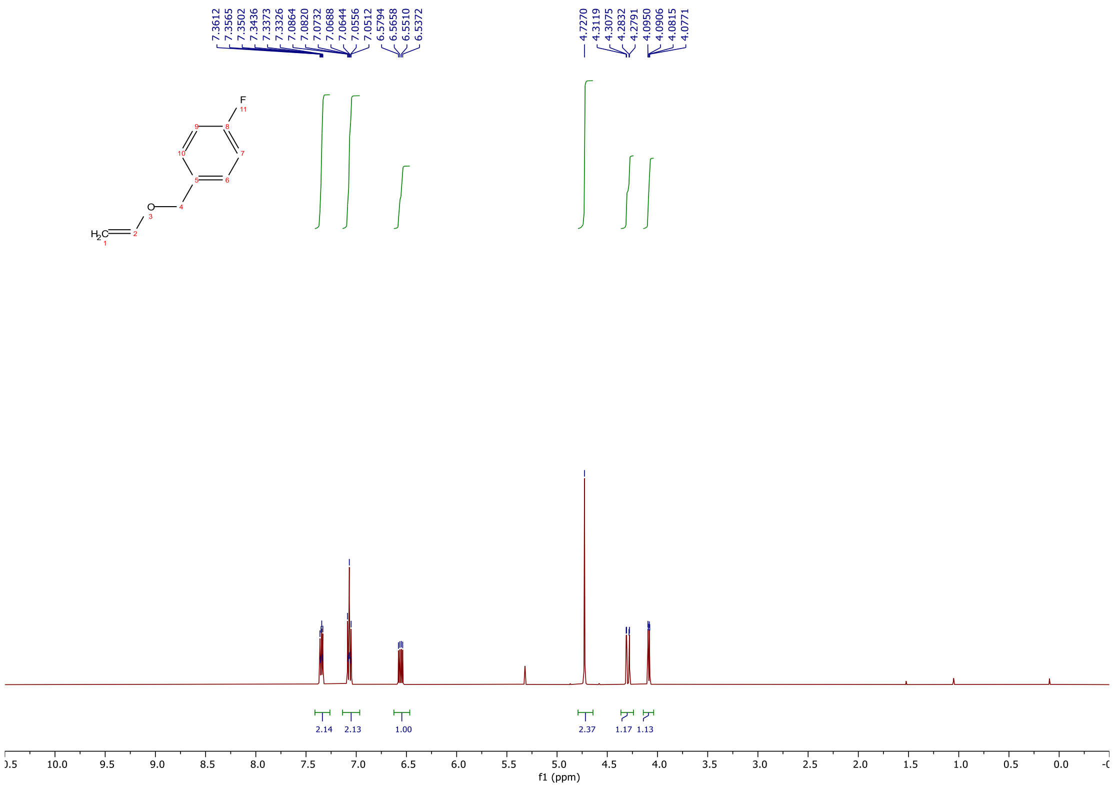
7.3612
7.3565
7.3502
7.3436
7.3373
7.3326
7.0864
7.0820
7.0732
7.0688
7.0644
7.0556
7.0512
6.5794
6.5658
6.5510
6.5372
4.7270
4.3119
4.3075
4.2832
4.2791
4.0950
4.0906
4.0815
4.0771
F
O
H_2C
2.14
2.13
1.00
2.37
1.17
1.13
f1 (ppm)

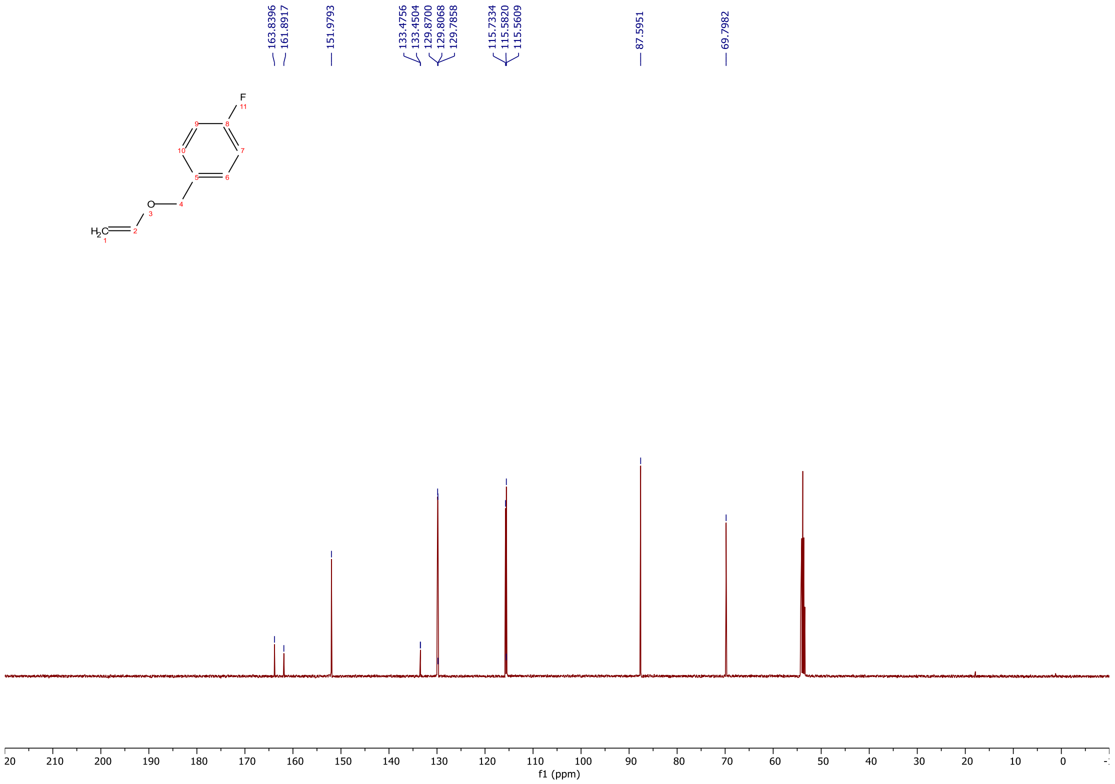
163.8396
161.8917
151.9793
133.4756
133.4504
129.8700
129.8068
129.7858
115.7334
115.5820
115.5609
87.5951
69.7982
F
H_2C
O
f1 (ppm)

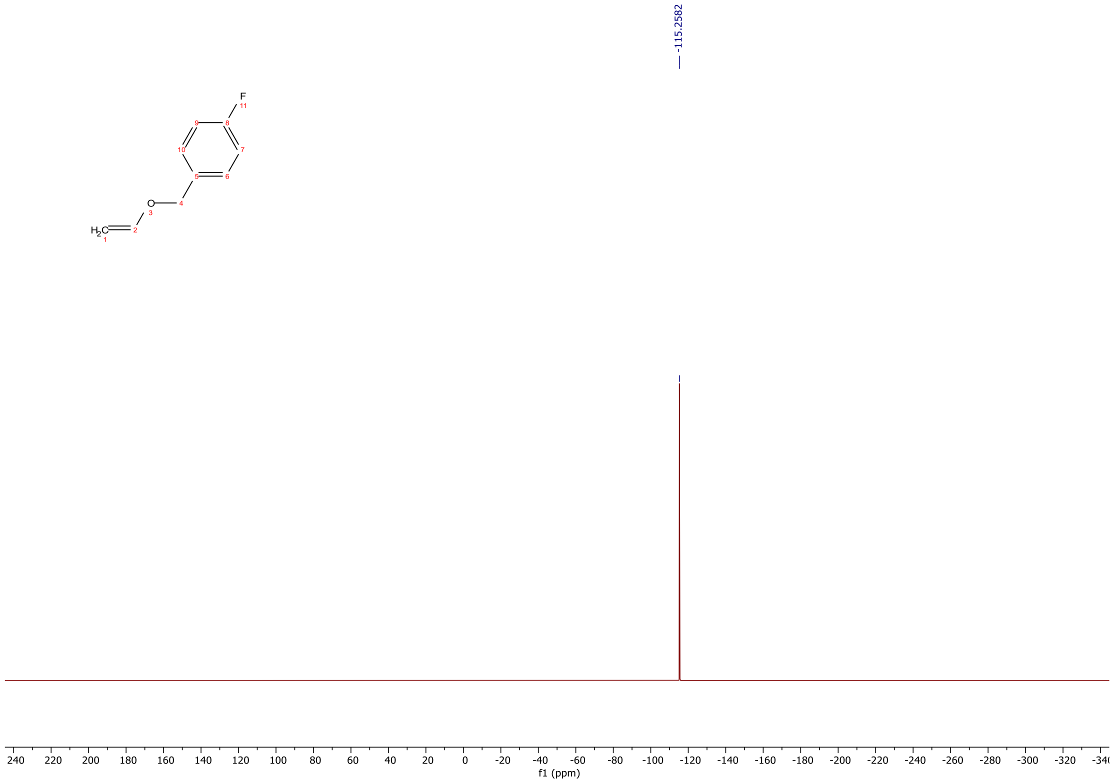
-115.2582
F
H_2C
O
f1 (ppm)

2t

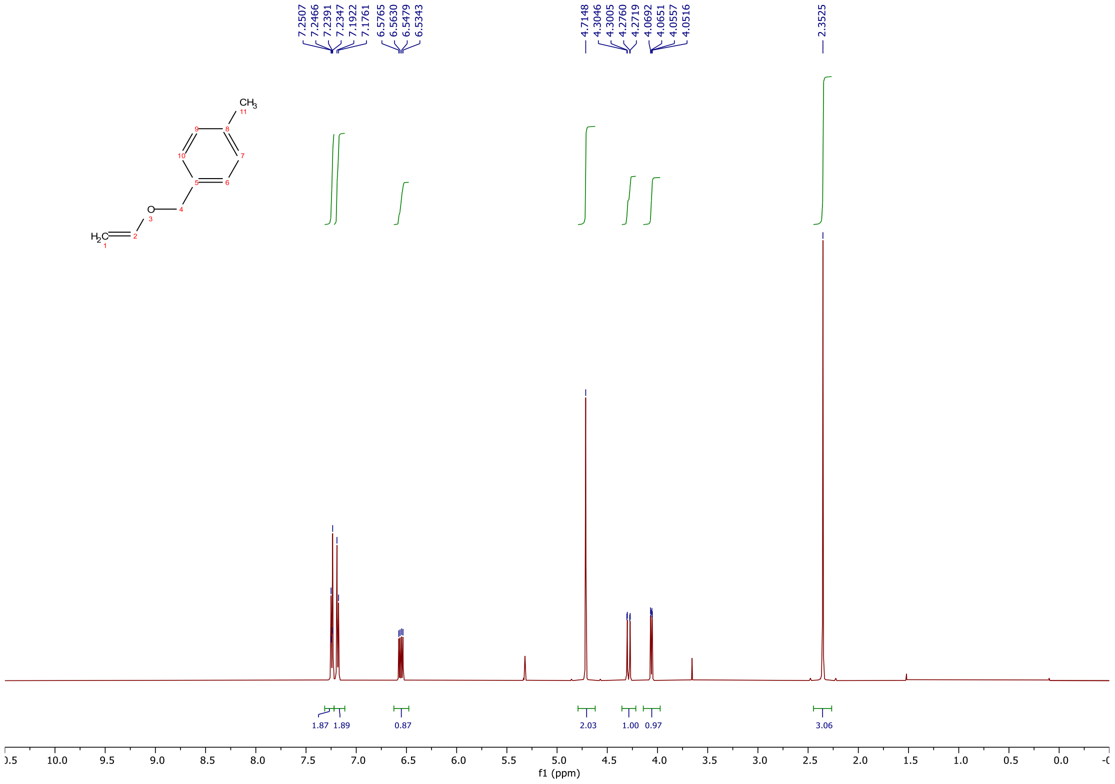
7.2507
7.2466
7.2391
7.2347
7.1922
7.1761
6.5765
6.5630
6.5479
6.5343
4.7148
4.3046
4.3005
4.2760
4.2719
4.0692
4.0651
4.0557
4.0516
2.3525
CH_3
H_2C
O
1.87
1.89
0.87
2.03
1.00
0.97
3.06
f1 (ppm)

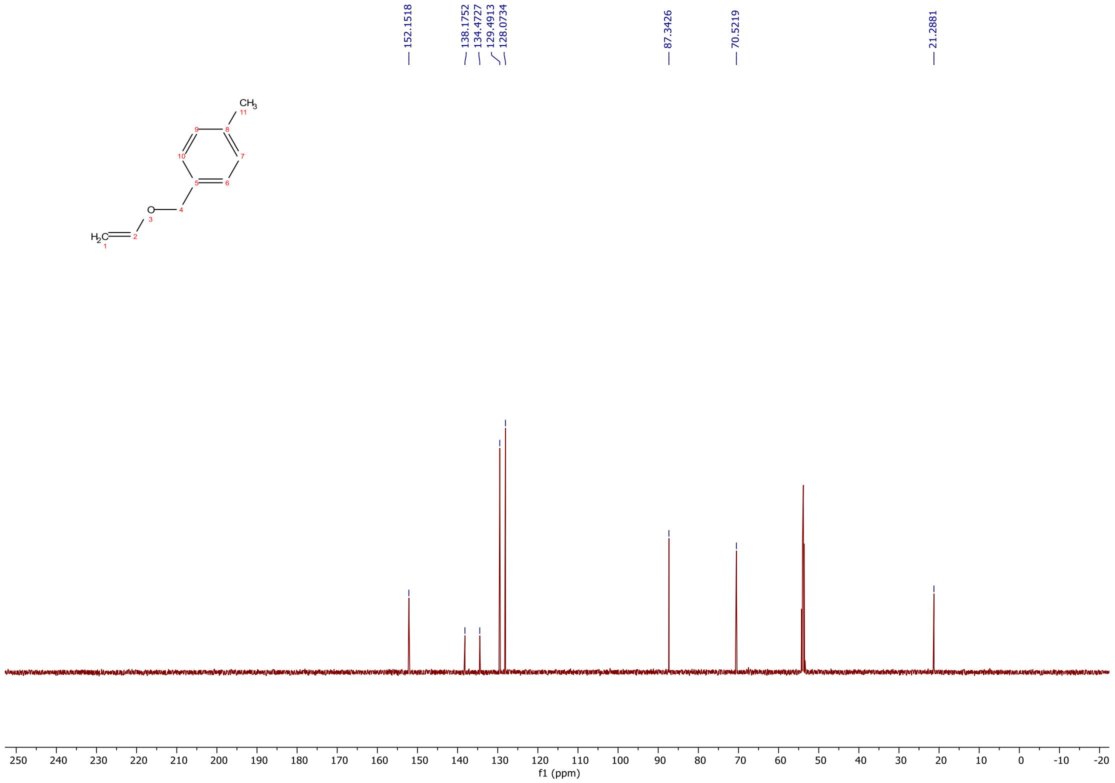
152.1518
138.1752
134.4727
129.4913
128.0734
87.3426
70.5219
21.2881
CH_3
H_2C
O
f1 (ppm)

2u

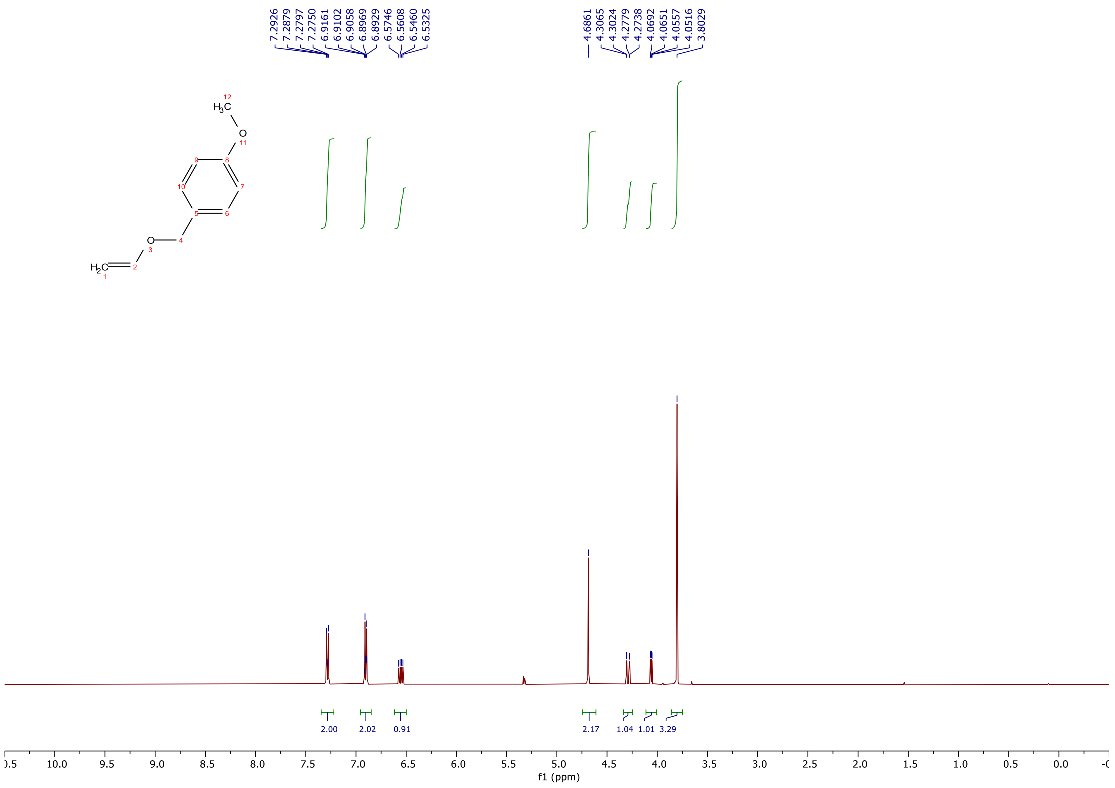

7.2926
7.2879
7.2797
7.2750
6.9161
6.9102
6.9058
6.8969
6.8929
6.5746
6.5608
6.5460
6.5325
4.6861
4.3065
4.3024
4.2779
4.2738
4.0692
4.0651
4.0557
4.0516
3.8029
2.00
2.02
0.91
2.17
1.04
1.01
3.29
f1 (ppm)

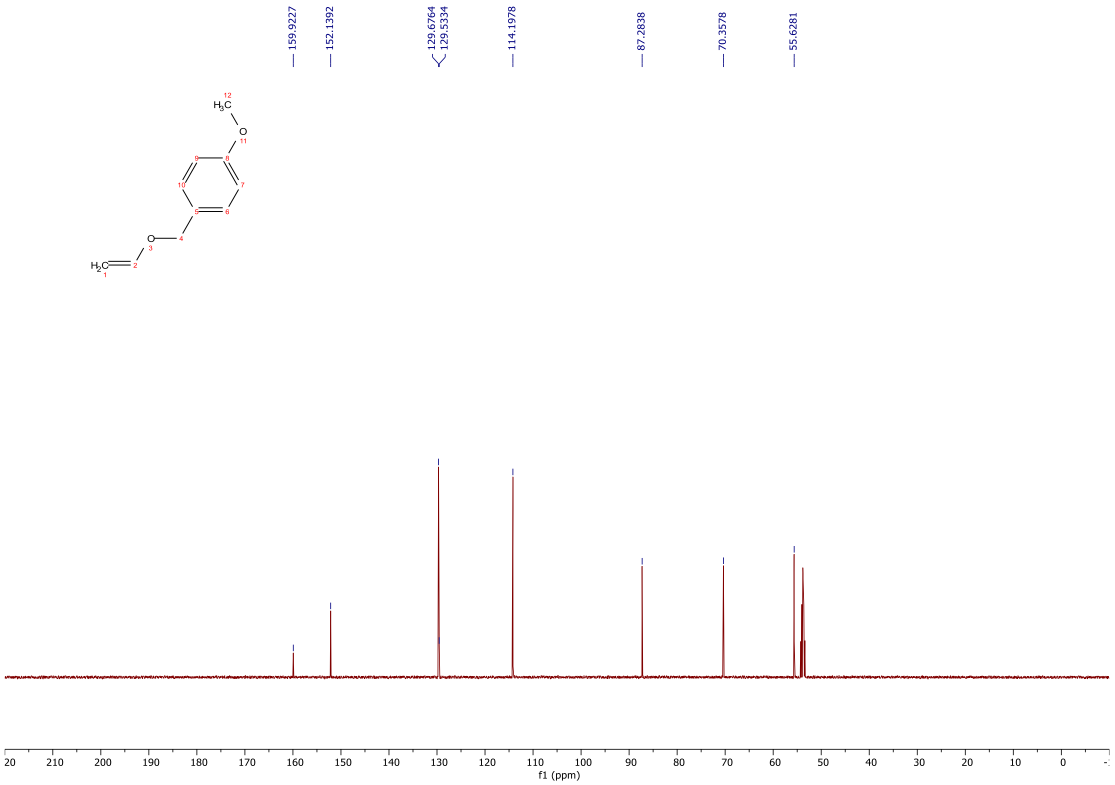

159.9227
152.1392
129.6764
129.5334
114.1978
87.2838
70.3578
55.6281
f1 (ppm)

3a-1

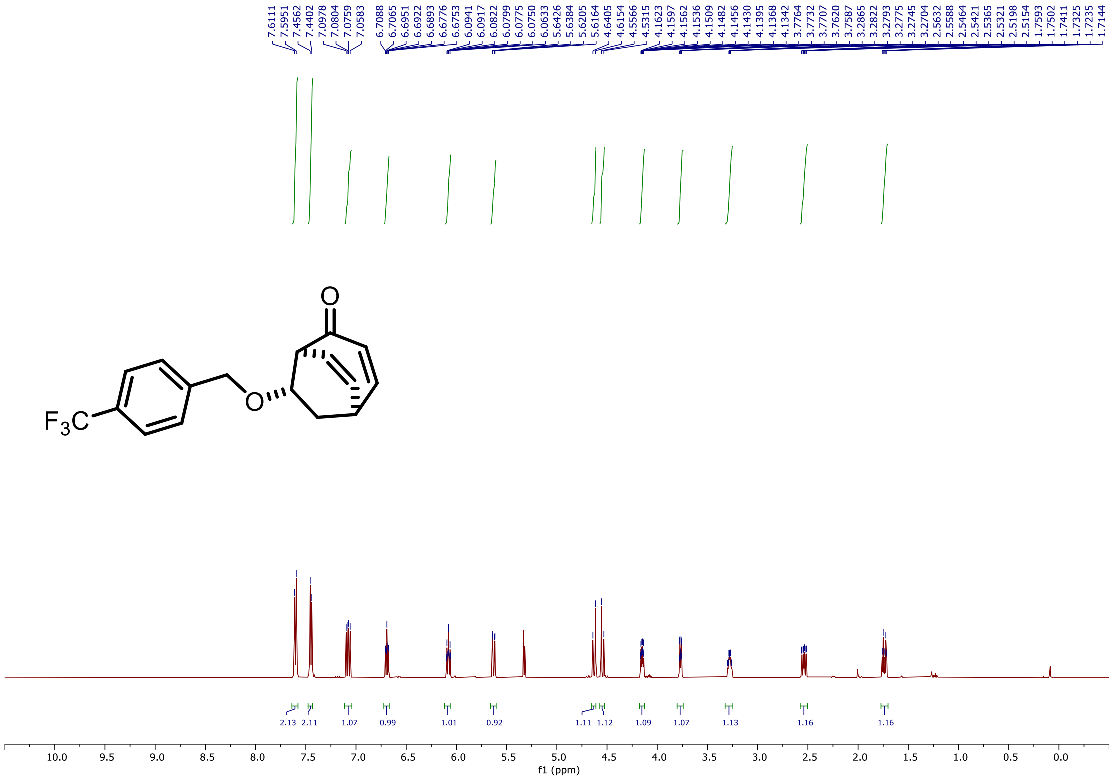

7.6111
7.5951
7.4562
7.4402
7.0978
7.0804
7.0759
7.0583
6.7088
6.7065
6.6951
6.6922
6.6893
6.6776
6.6753
6.0941
6.0917
6.0822
6.0799
6.0775
6.0750
6.0633
5.6426
5.6384
5.6205
5.6164
4.6405
4.6154
4.5566
4.5315
4.1623
4.1597
4.1562
4.1536
4.1509
4.1482
4.1456
4.1430
4.1395
4.1368
4.1342
3.7764
3.7732
3.7707
3.7620
3.7587
3.2865
3.2822
3.2793
3.2775
3.2745
3.2704
2.5632
2.5588
2.5464
2.5421
2.5365
2.5321
2.5198
2.5154
1.7593
1.7502
1.7411
1.7325
1.7235
1.7144
2.13
2.11
1.07
0.99
1.01
0.92
1.11
1.12
1.09
1.07
1.13
1.16
1.16
O
F3C
O
f1 (ppm)

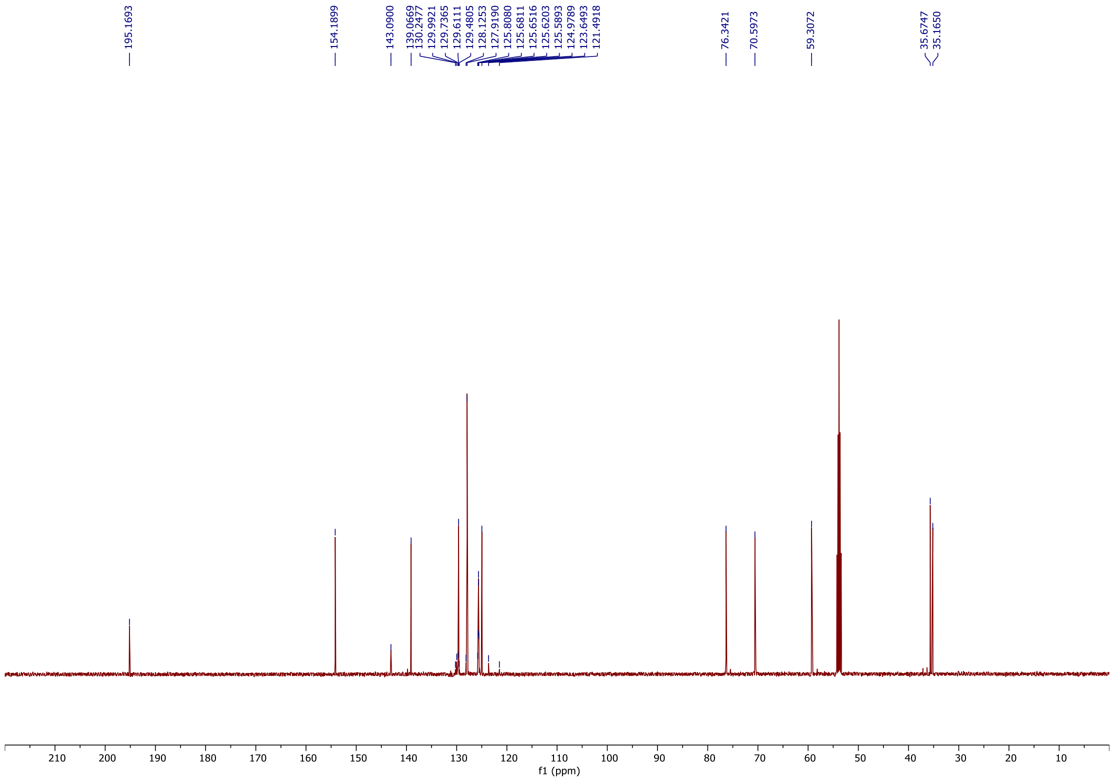

195.1693
154.1899
143.0900
139.0669
130.2477
129.9921
129.7365
129.6111
129.4805
128.1253
127.9190
125.8080
125.6811
125.6516
125.6203
125.5893
124.9789
123.6493
121.4918
76.3421
70.5973
59.3072
35.6747
35.1650
f1 (ppm)

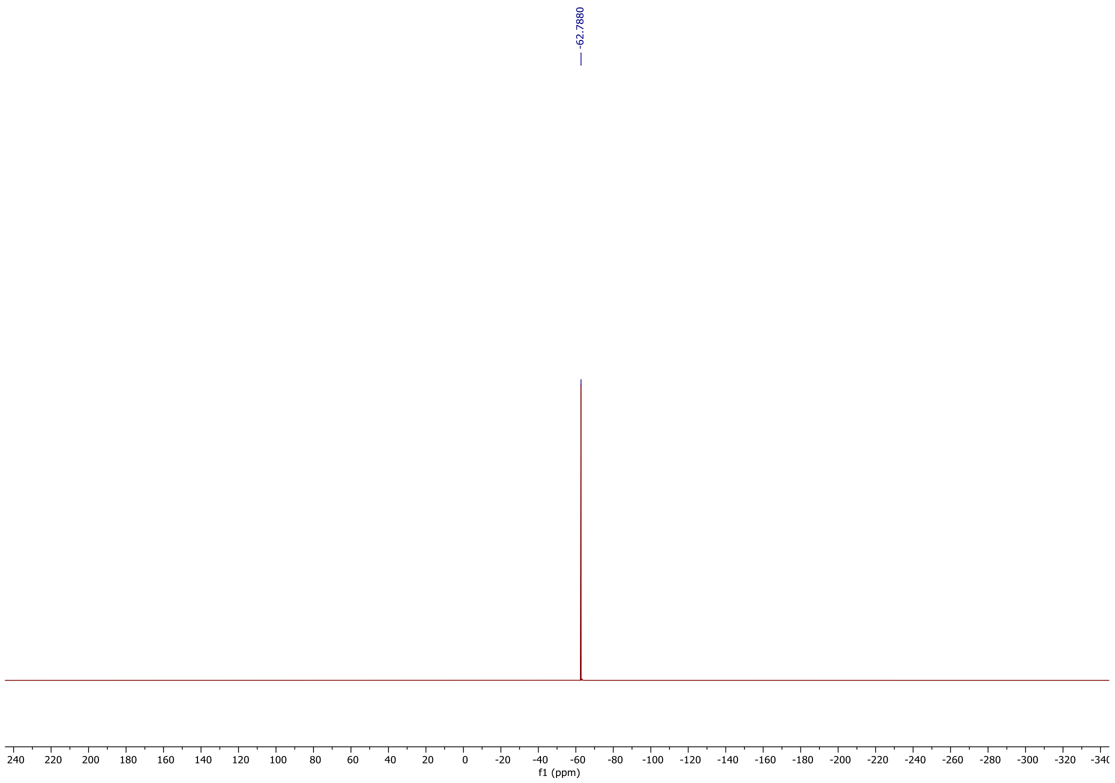
-62.7880
240 220 200 180 160 140 120 100 80 60 40 20 0 -20 -40 -60 -80 -100 -120 -140 -160 -180 -200 -220 -240 -260 -280 -300 -320 -340
f1 (ppm)

2b

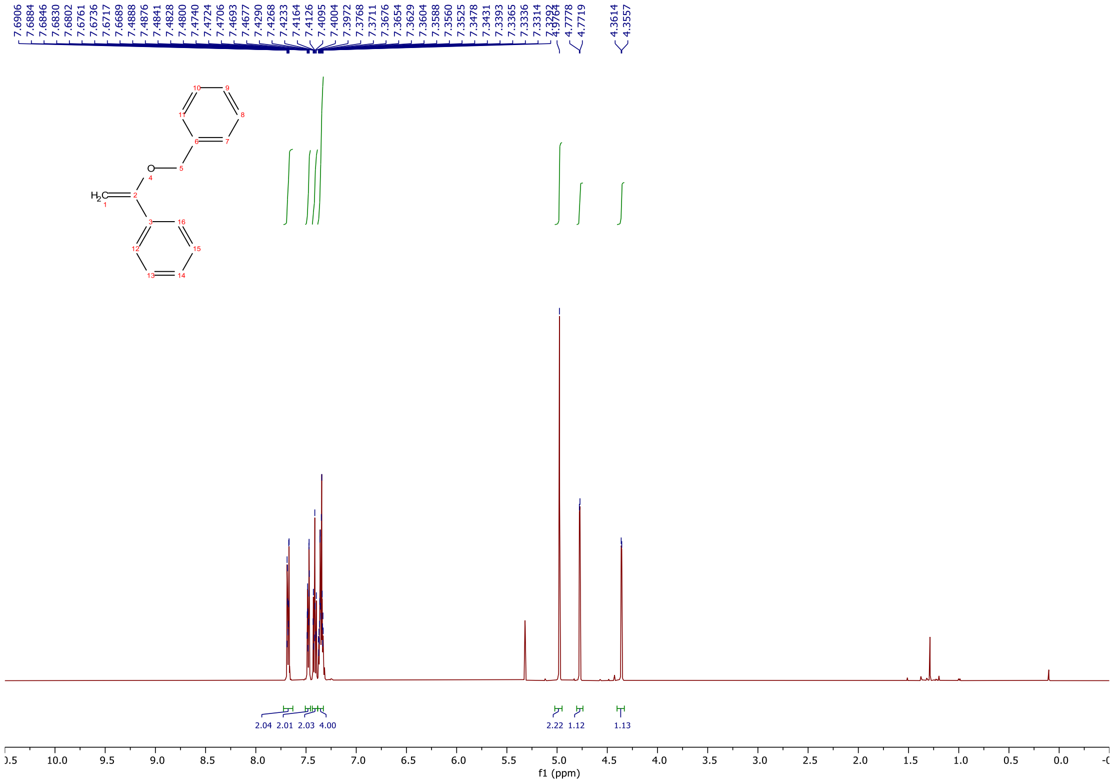

7.6906
7.6884
7.6846
7.6830
7.6802
7.6761
7.6736
7.6717
7.6689
7.4888
7.4876
7.4841
7.4828
7.4800
7.4740
7.4724
7.4706
7.4693
7.4677
7.4290
7.4268
7.4233
7.4164
7.4126
7.4095
7.4004
7.3972
7.3768
7.3711
7.3676
7.3654
7.3629
7.3604
7.3588
7.3560
7.3525
7.3478
7.3431
7.3393
7.3365
7.3336
7.3314
7.3292
4.9764
4.7778
4.7719
4.3614
4.3557
2.04 2.01 2.03 4.00
2.22 1.12
1.13
f1 (ppm)

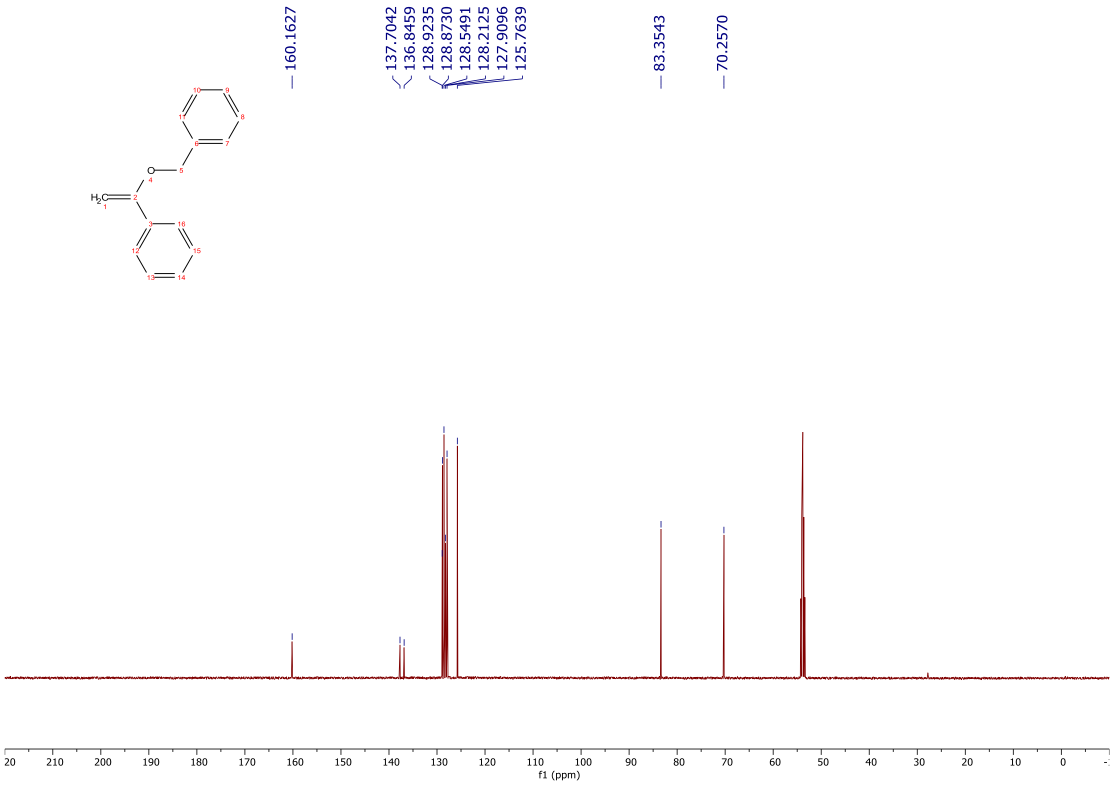

160.1627
137.7042
136.8459
128.9235
128.8730
128.5491
128.2125
127.9096
125.7639
83.3543
70.2570
f1 (ppm)

2c

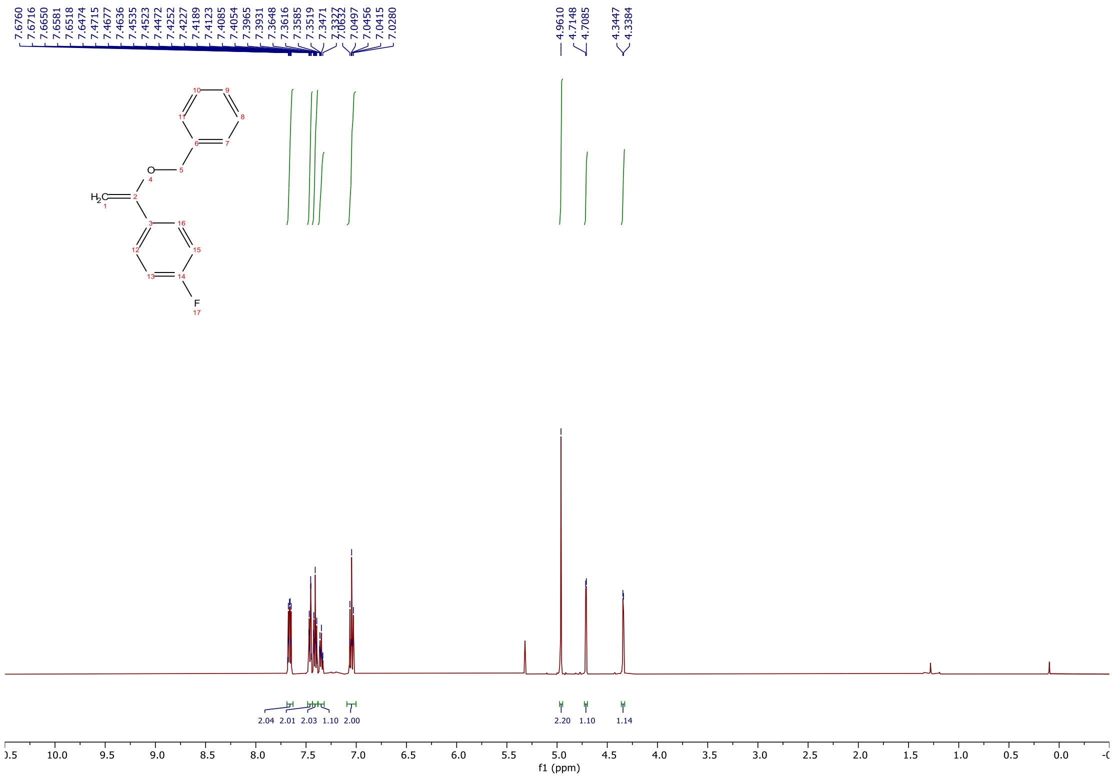
7.6760 7.6716 7.6650 7.6581 7.6518 7.6474 7.4715 7.4677 7.4636 7.4535 7.4523 7.4472 7.4252 7.4227 7.4189 7.4123 7.4085 7.4054 7.3965 7.3931 7.3648 7.3616 7.3585 7.3519 7.3471
7.0497 7.0456 7.0415 7.0280
4.9610 4.7148 4.7085
4.3447 4.3384
H2C
O
F
2.04 2.01 2.03 1.10 2.00
2.20 1.10 1.14
f1 (ppm)

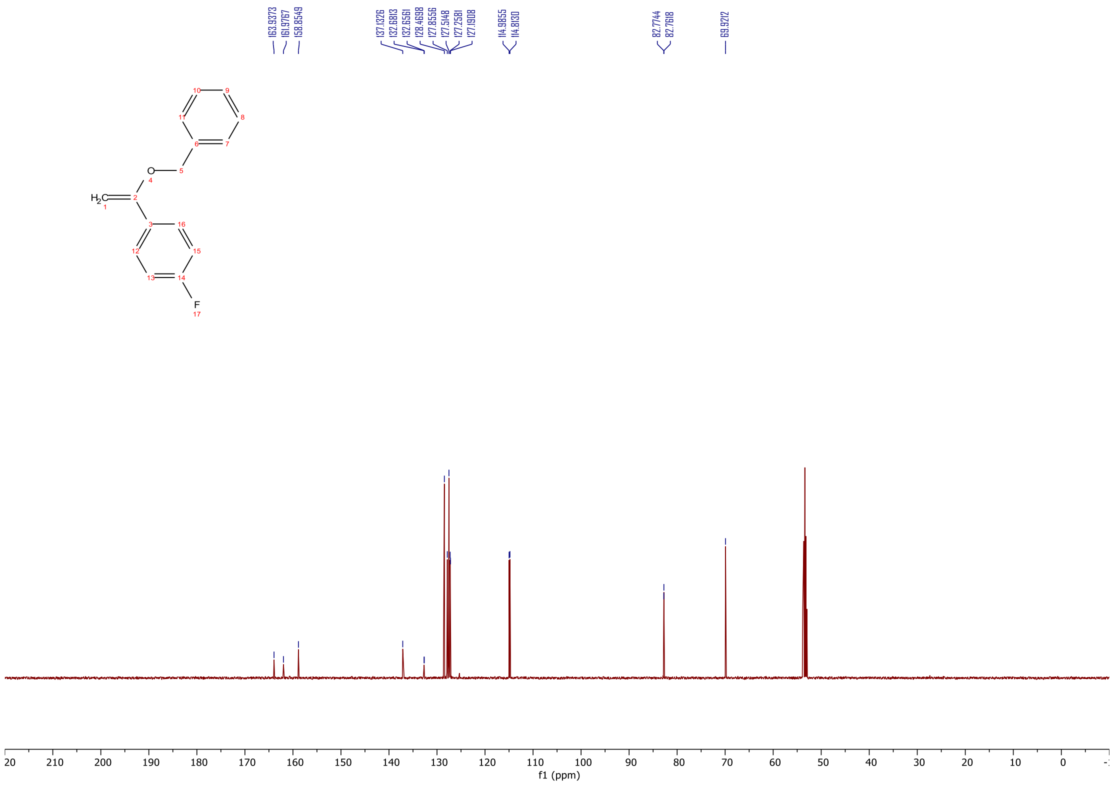
163.9373 161.9767 158.8549
137.1326 132.6813 132.6561 128.4698 127.8556 127.5148 127.2581 127.1908
114.9855 114.8130
82.7744 82.7618
69.9212
H2C
O
F
f1 (ppm)

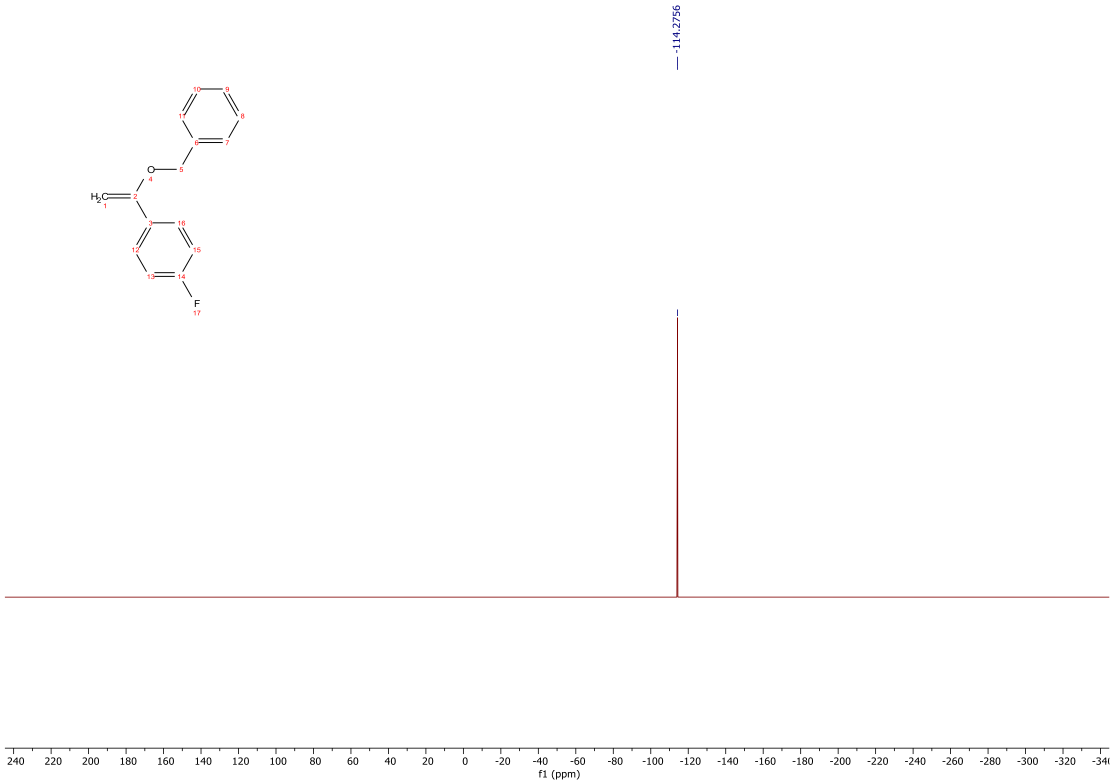
-114.2756
f1 (ppm)

2d

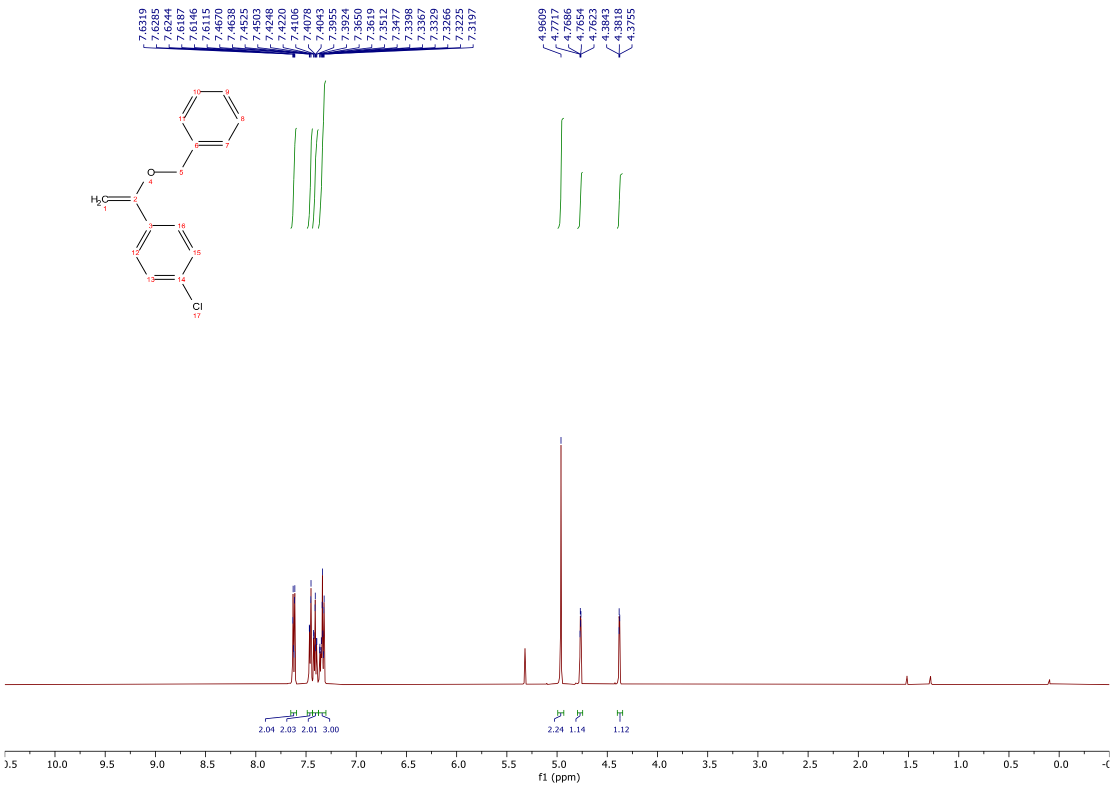

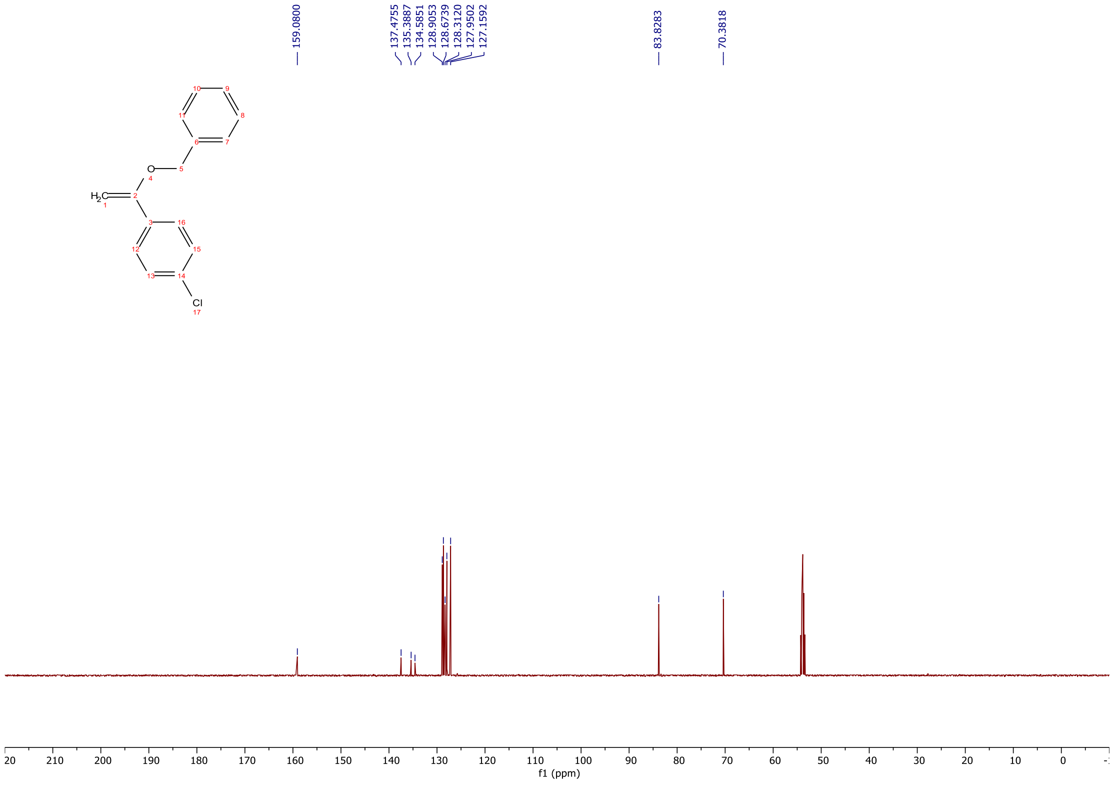

2e

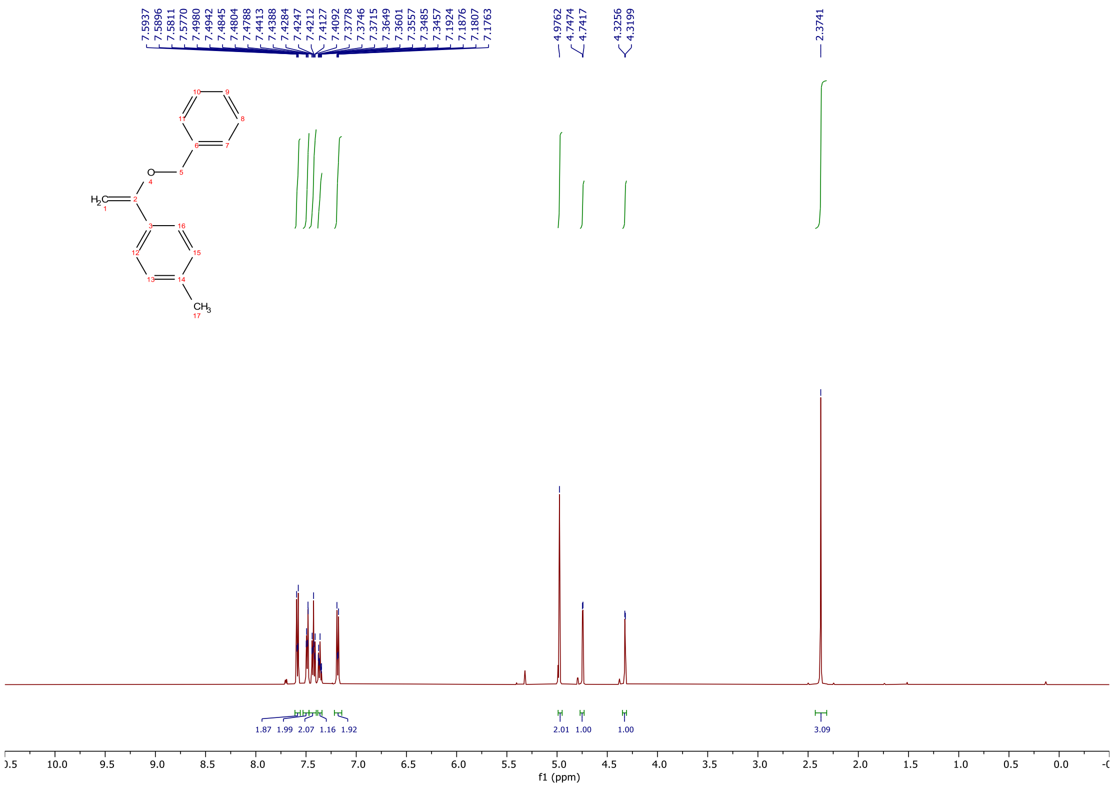

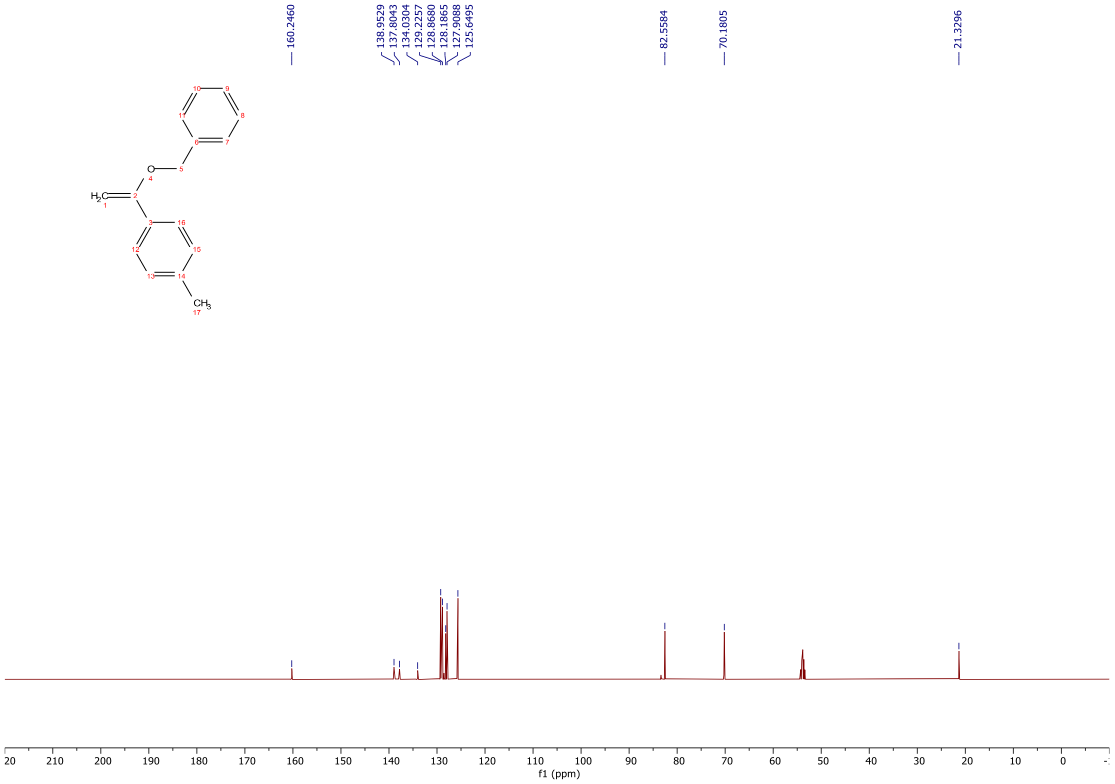

2f

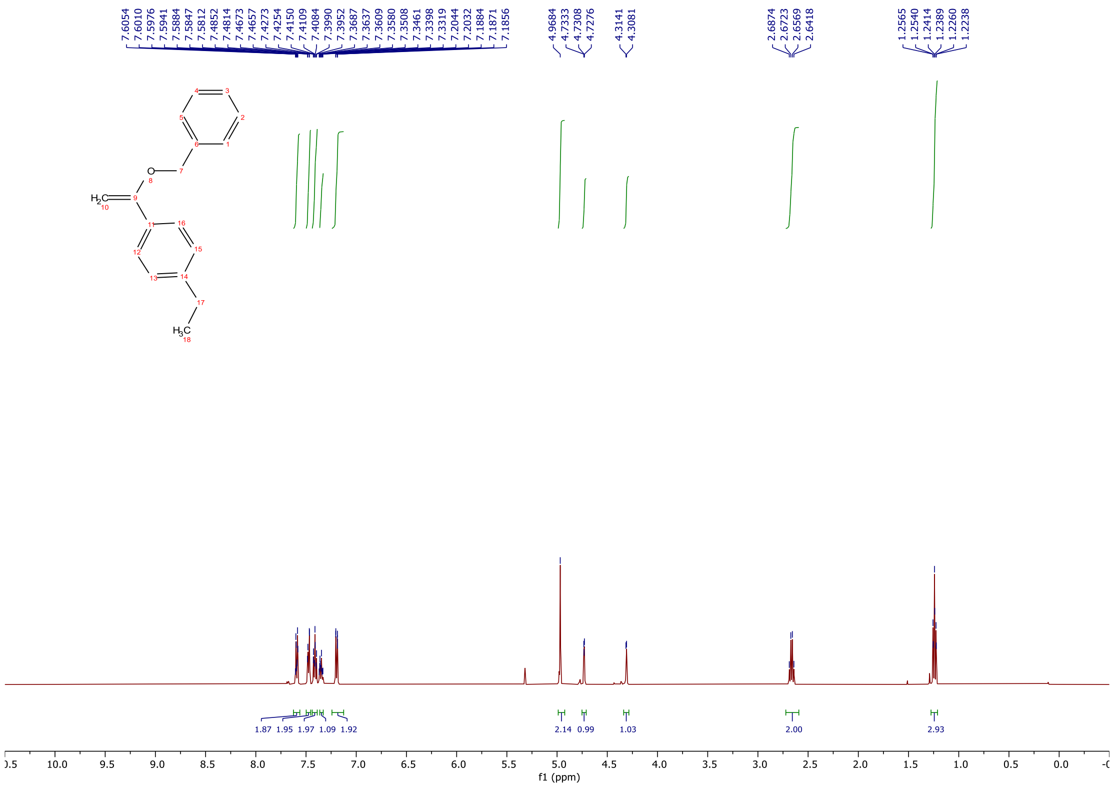
7.6054
7.6010
7.5976
7.5941
7.5884
7.5847
7.5812
7.4852
7.4814
7.4673
7.4657
7.4273
7.4254
7.4150
7.4109
7.4084
7.3990
7.3952
7.3687
7.3637
7.3609
7.3580
7.3508
7.3461
7.3398
7.3319
7.2044
7.2032
7.1884
7.1871
7.1856
4.9684
4.7333
4.7308
4.7276
4.3141
4.3081
2.6874
2.6723
2.6569
2.6418
1.2565
1.2540
1.2414
1.2389
1.2260
1.2238
1.87
1.95
1.97
1.09
1.92
2.14
0.99
1.03
2.00
2.93
f1 (ppm)

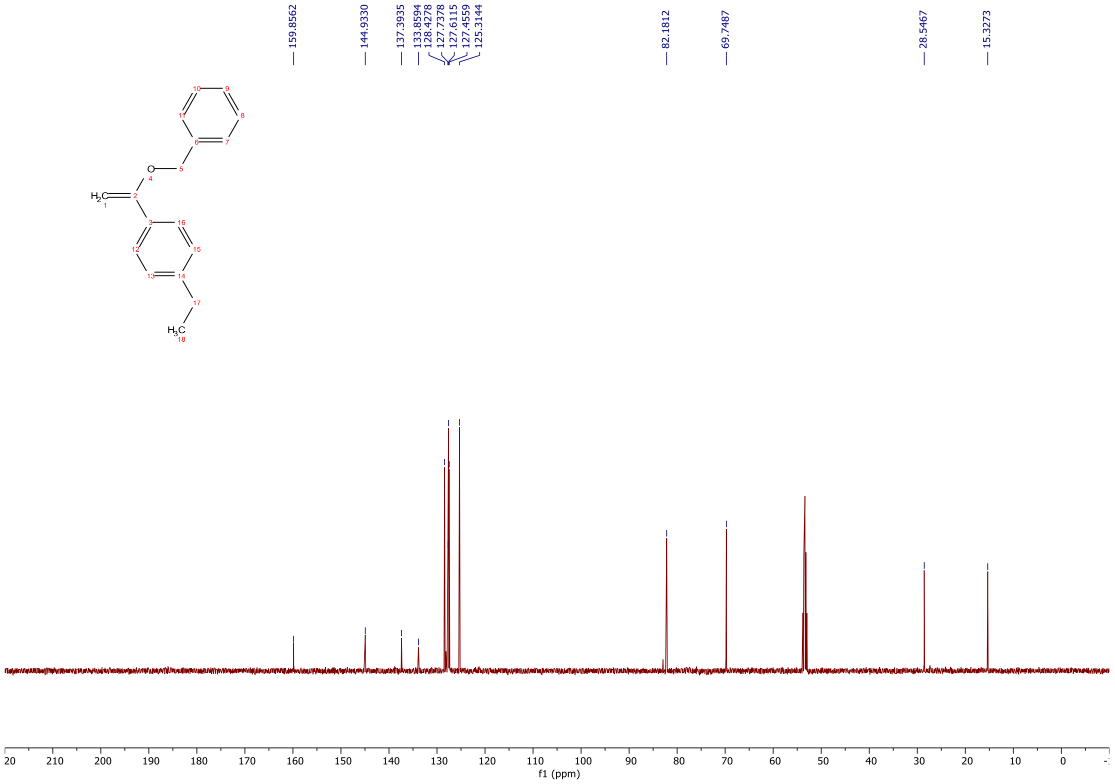
159.8562
144.9330
137.3935
133.8594
128.4278
127.7378
127.6115
127.4559
125.3144
82.1812
69.7487
28.5467
15.3273
f1 (ppm)

2g

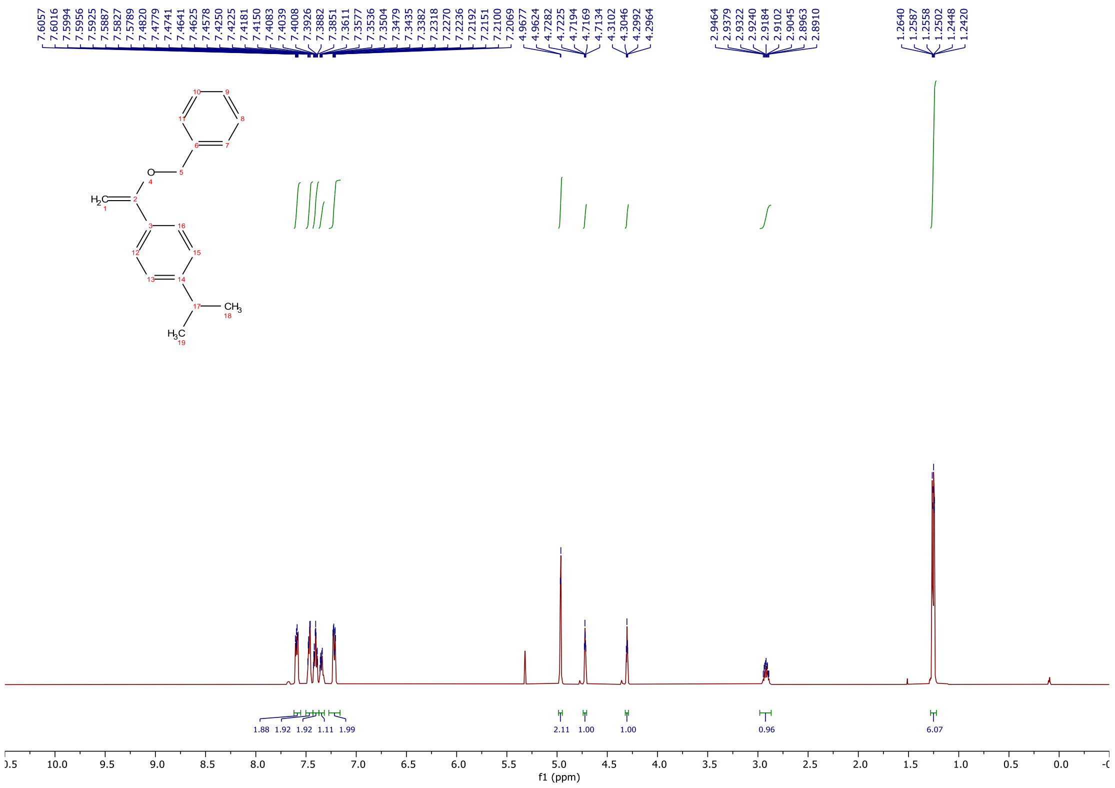
7.6057
7.6016
7.5994
7.5956
7.5925
7.5887
7.5827
7.5789
7.4820
7.4779
7.4741
7.4641
7.4625
7.4578
7.4250
7.4225
7.4181
7.4150
7.4083
7.4039
7.4008
7.3926
7.3882
7.3851
7.3611
7.3577
7.3536
7.3504
7.3479
7.3435
7.3382
7.2318
7.2270
7.2236
7.2192
7.2151
7.2100
7.2069
4.9677
4.9624
4.7282
4.7225
4.7194
4.7169
4.7134
4.3102
4.3046
4.2992
4.2964
2.9464
2.9379
2.9322
2.9240
2.9184
2.9102
2.9045
2.8963
2.8910
1.2640
1.2587
1.2558
1.2502
1.2448
1.2420
1.88 1.92 1.92 1.11 1.99
2.11 1.00 1.00 0.96 6.07
f1 (ppm)

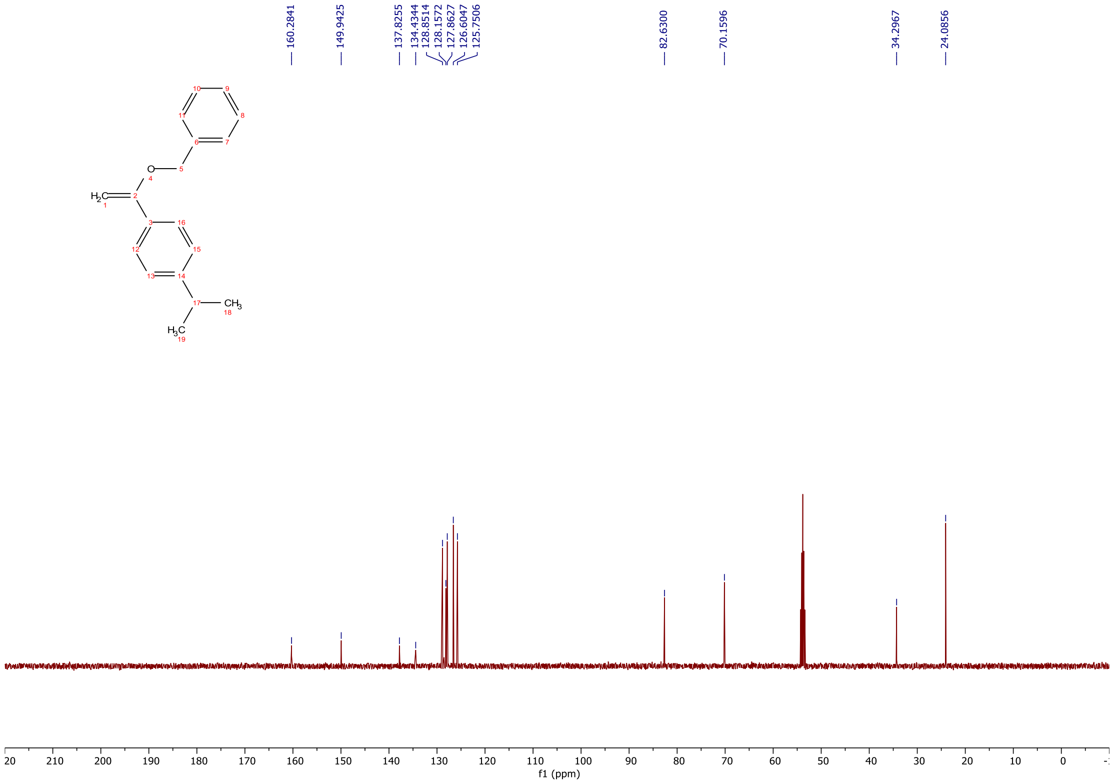
160.2841
149.9425
137.8255
134.4344
128.8514
128.1572
127.8627
126.6047
125.7506
82.6300
70.1596
34.2967
24.0856
f1 (ppm)

2h

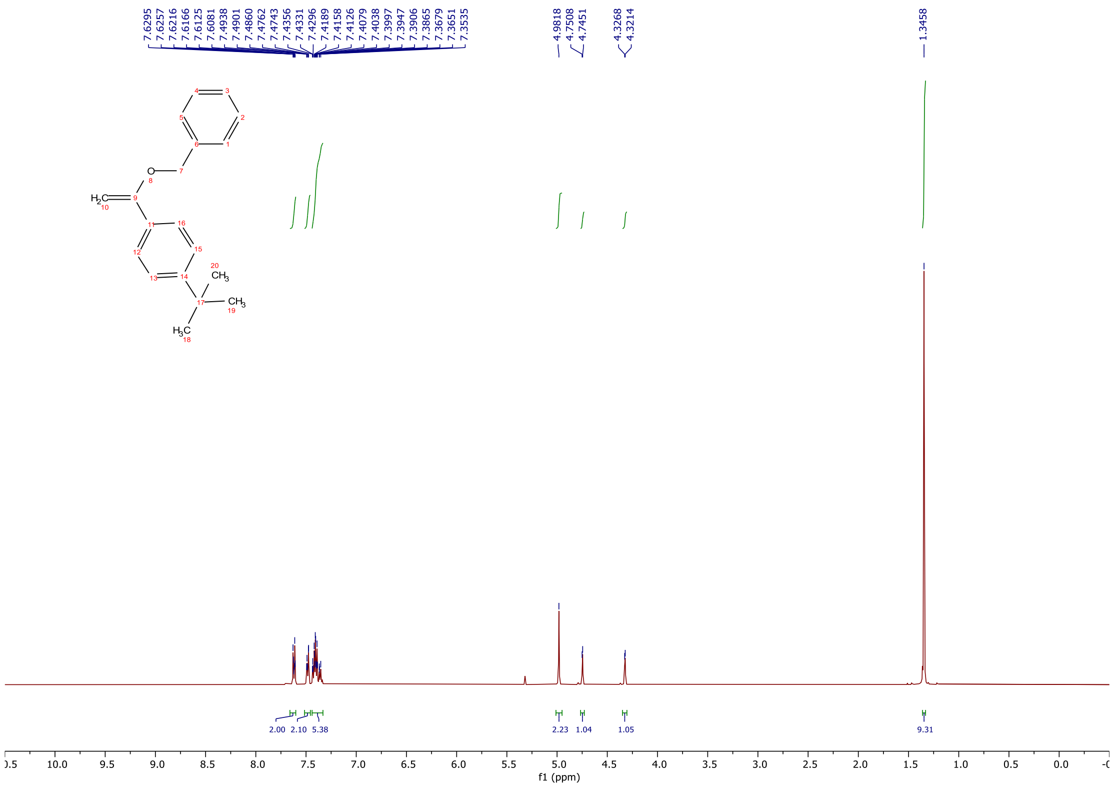
7.6295
7.6257
7.6216
7.6166
7.6125
7.6081
7.4938
7.4901
7.4860
7.4762
7.4743
7.4356
7.4331
7.4296
7.4189
7.4158
7.4126
7.4079
7.4038
7.3997
7.3947
7.3906
7.3865
7.3679
7.3651
7.3535
4.9818
4.7508
4.7451
4.3268
4.3214
1.3458
2.00
2.10
5.38
2.23
1.04
1.05
9.31
f1 (ppm)

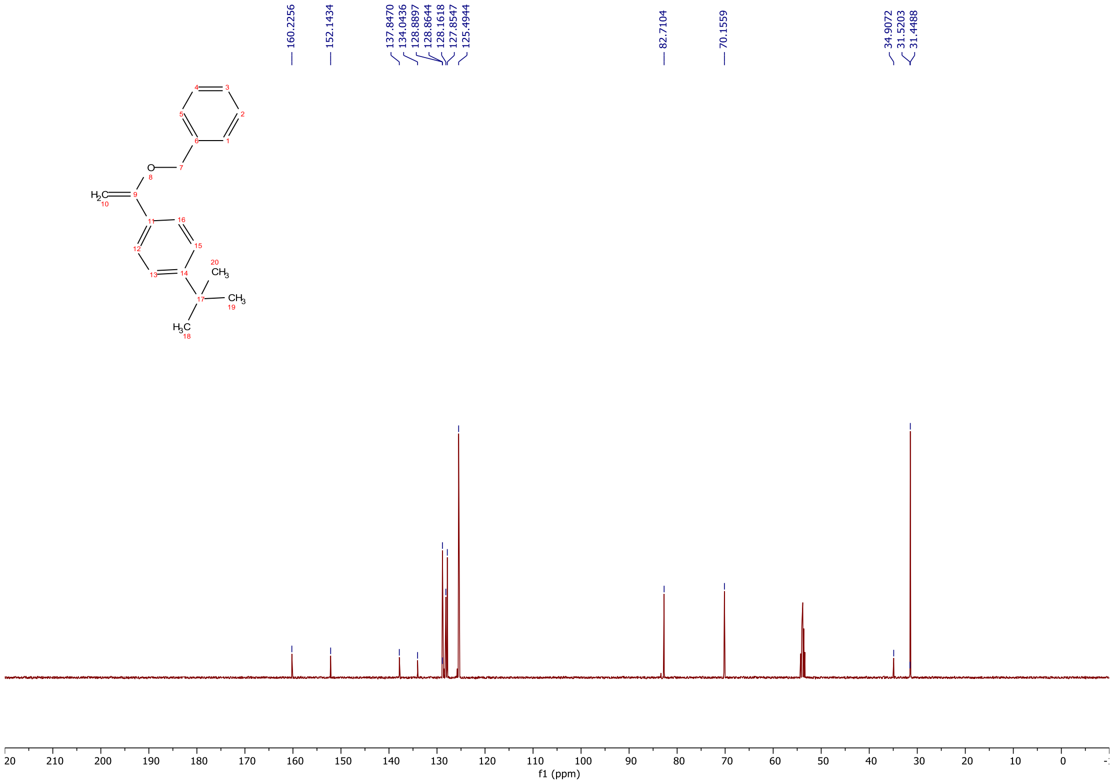
160.2256
152.1434
137.8470
134.0436
128.8897
128.8644
128.1618
127.8547
125.4944
82.7104
70.1559
34.9072
31.5203
31.4488
f1 (ppm)

2i

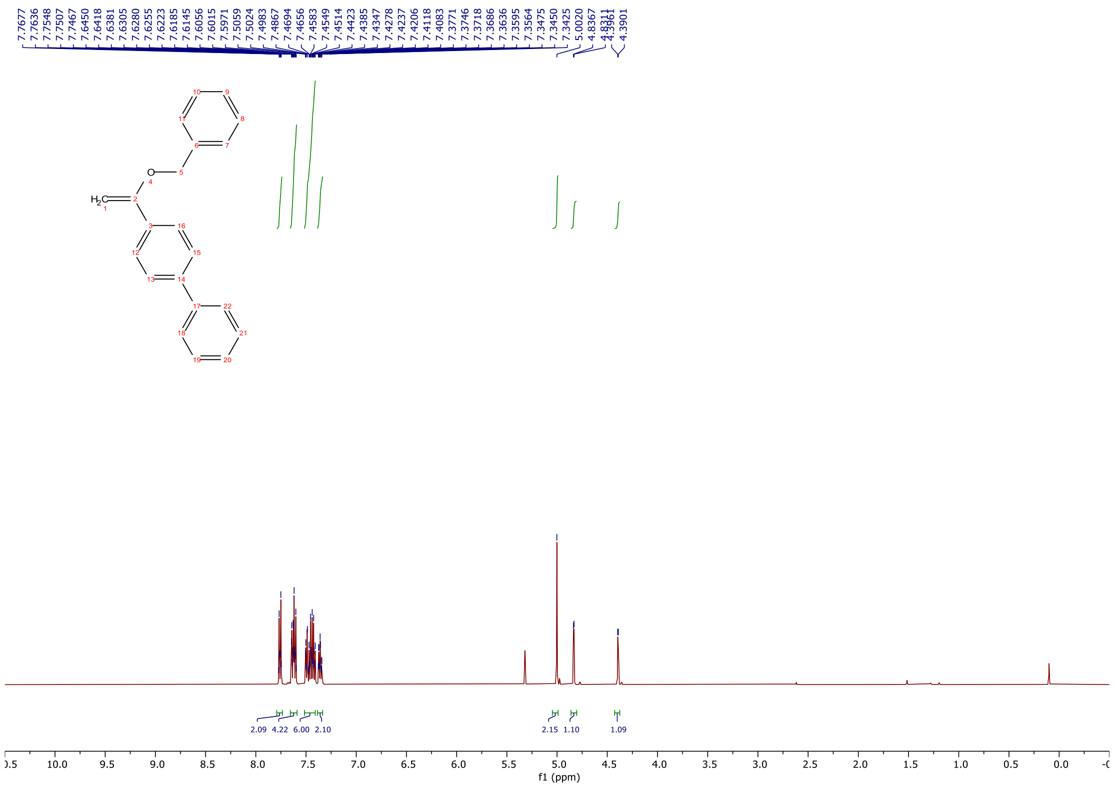

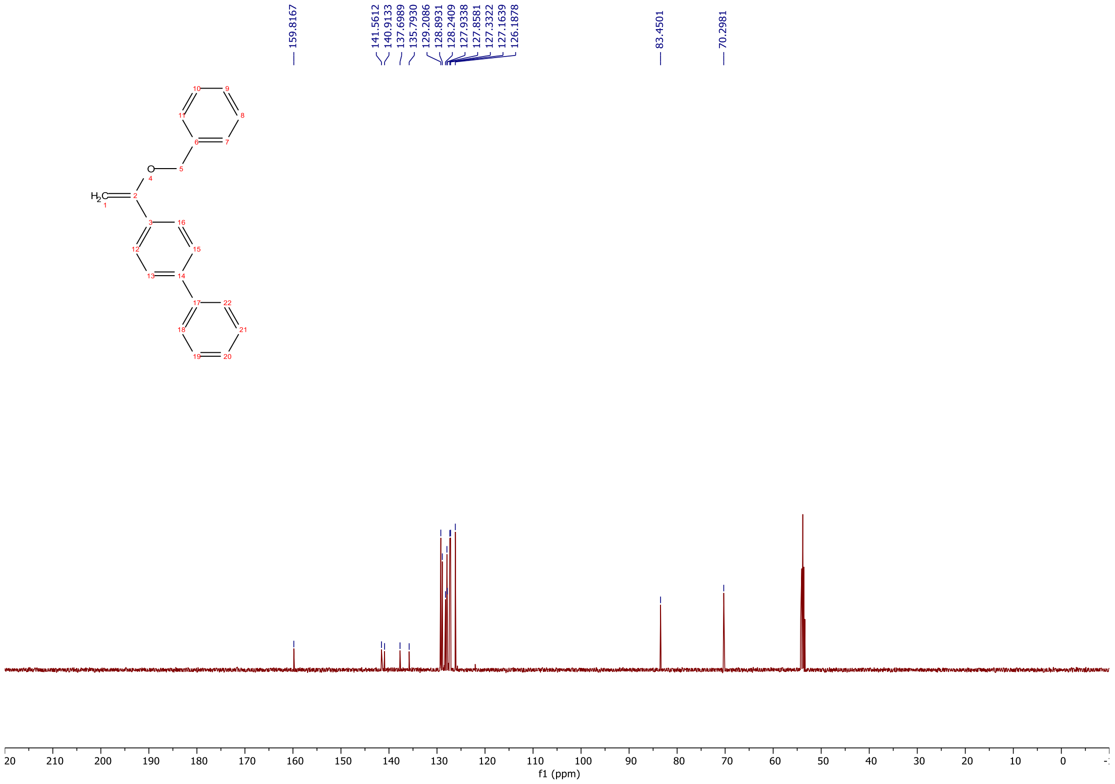

2j

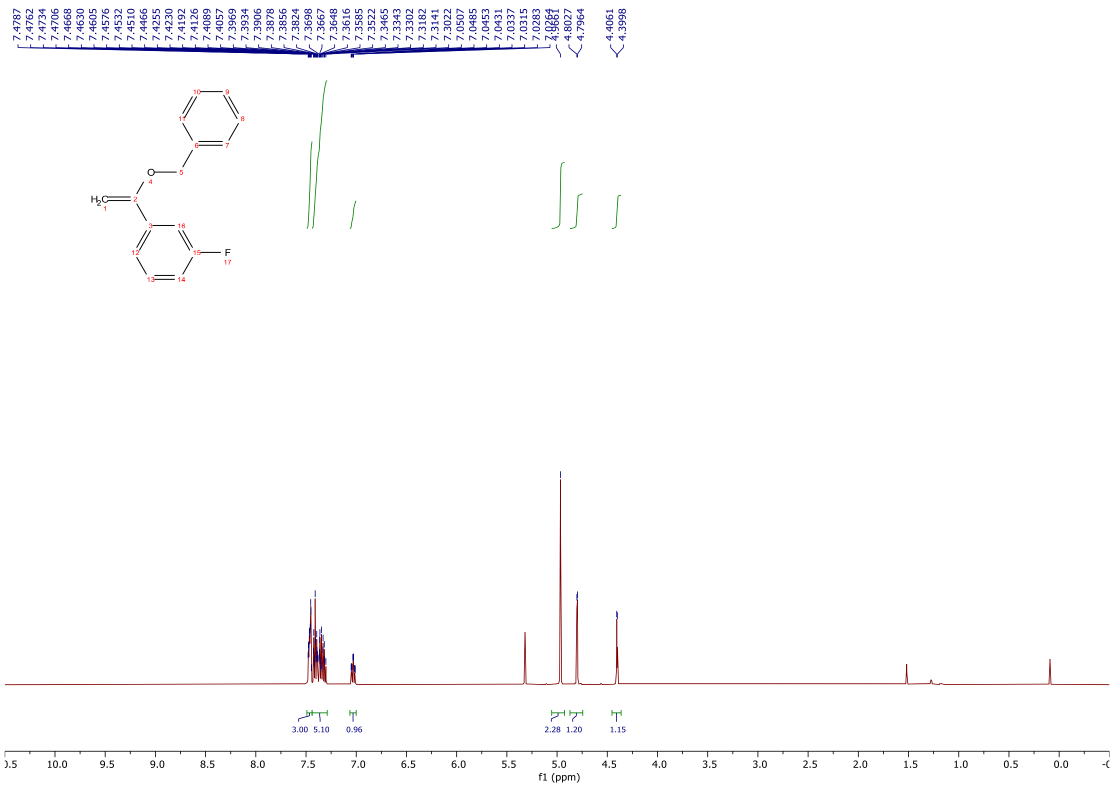

7.4787
7.4762
7.4734
7.4706
7.4668
7.4630
7.4605
7.4576
7.4532
7.4510
7.4466
7.4255
7.4230
7.4192
7.4126
7.4089
7.4057
7.3969
7.3934
7.3906
7.3878
7.3856
7.3824
7.3698
7.3667
7.3648
7.3616
7.3585
7.3522
7.3465
7.3343
7.3302
7.3182
7.3141
7.3022
7.0507
7.0485
7.0453
7.0431
7.0337
7.0315
7.0283
7.0264
4.9661
4.8027
4.7964
4.4061
4.3998
3.00
5.10
0.96
2.28
1.20
1.15
f1 (ppm)

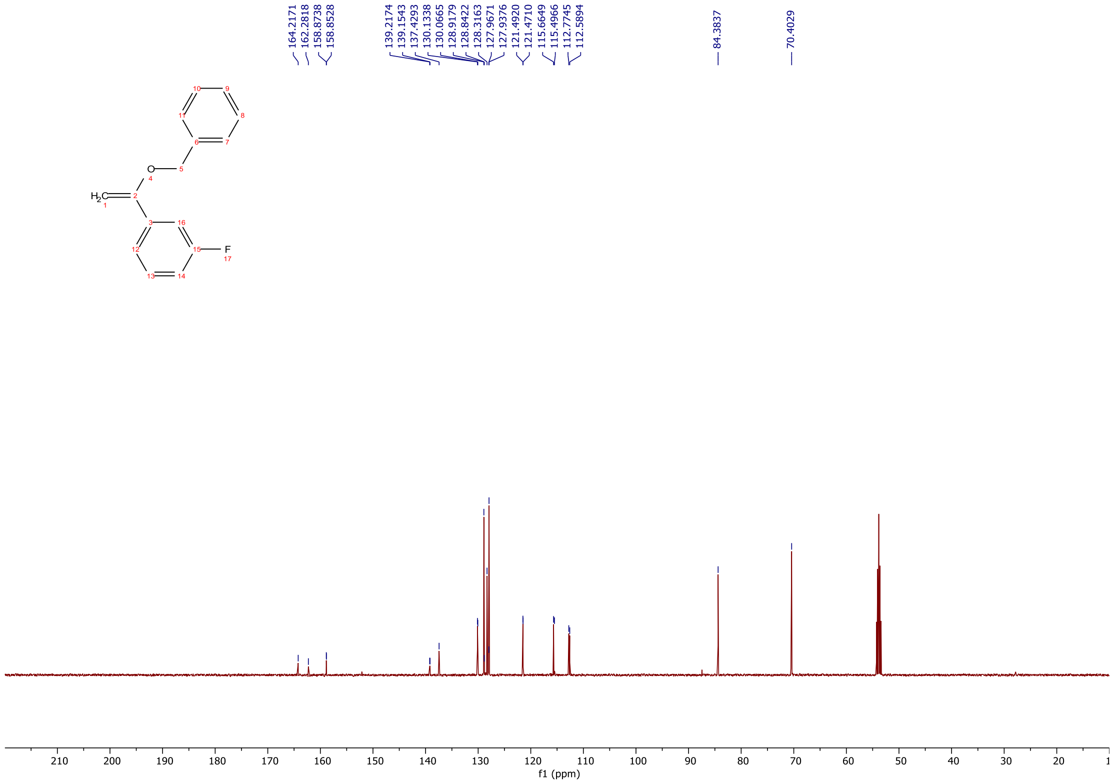

164.2171
162.2818
158.8738
158.8528
139.2174
139.1543
137.4293
130.1338
130.0665
128.9179
128.8422
128.3163
127.9671
127.9376
121.4920
121.4710
115.6649
115.4966
112.7745
112.5894
84.3837
70.4029
f1 (ppm)

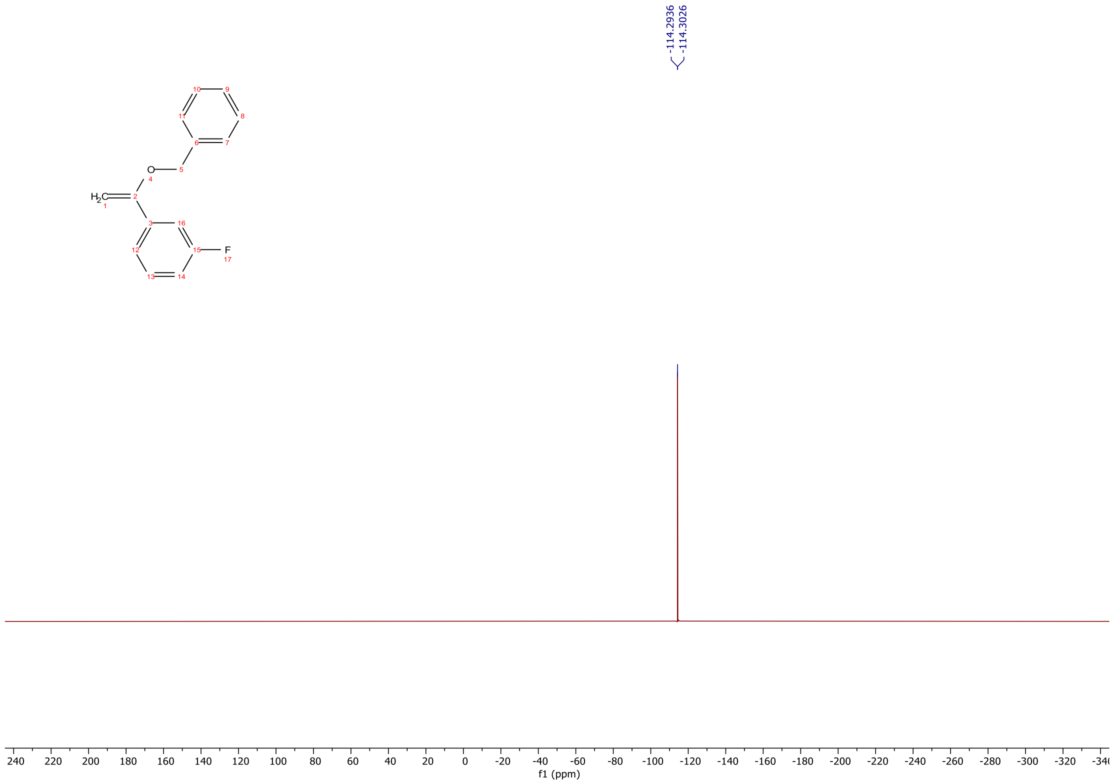

-114.2936
-114.3026
240
220
200
180
160
140
120
100
80
60
40
20
0
-20
-40
-60
-80
-100
-120
-140
-160
-180
-200
-220
-240
-260
-280
-300
-320
f1 (ppm)

2k

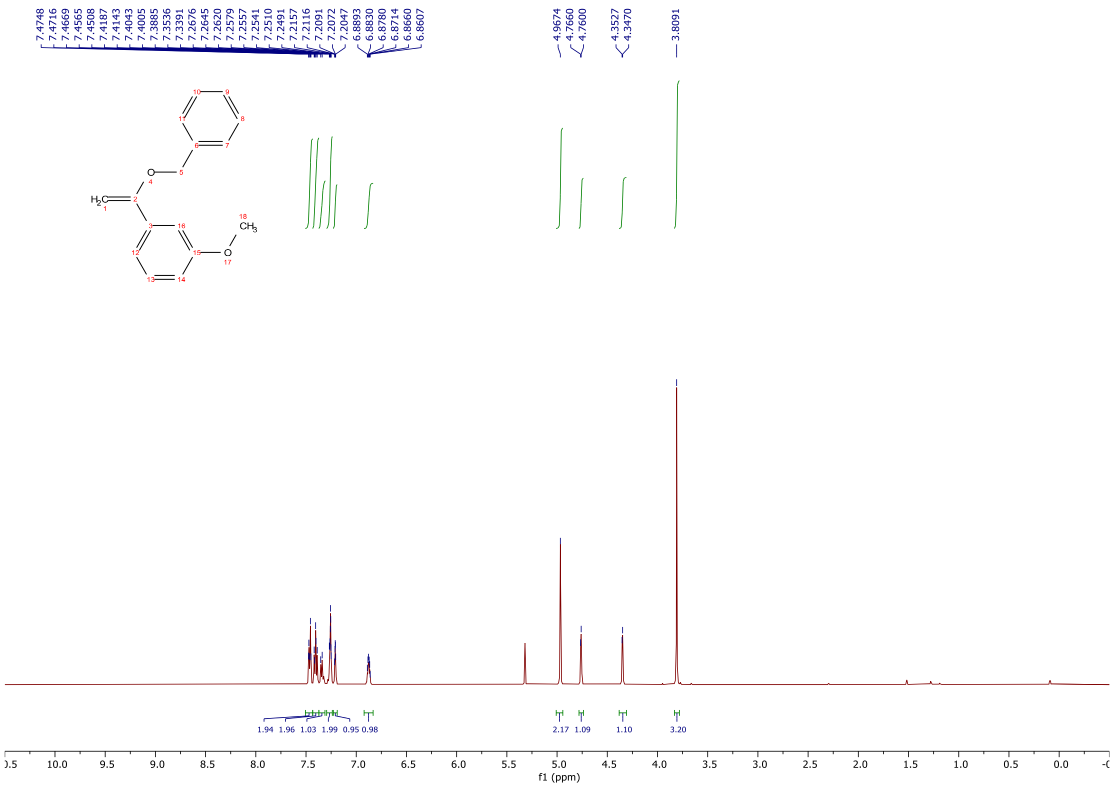

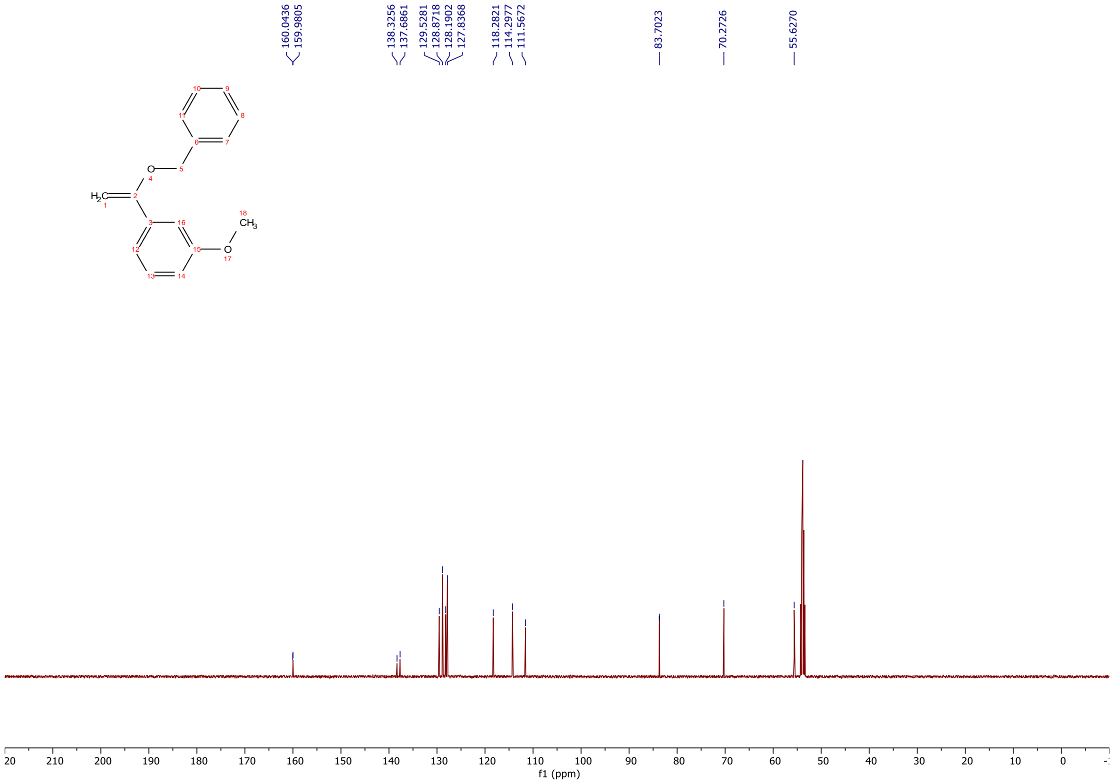

2l

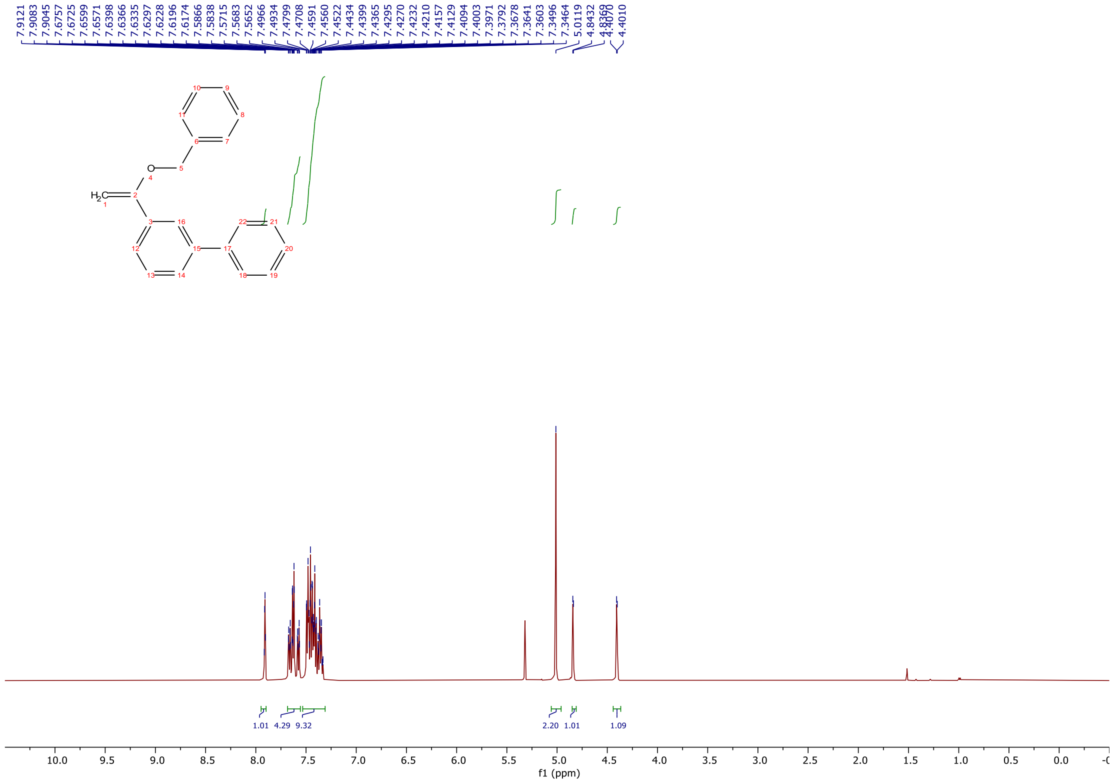
7.9121
7.9083
7.9045
7.6757
7.6725
7.6599
7.6571
7.6398
7.6366
7.6335
7.6297
7.6228
7.6196
7.6174
7.5866
7.5838
7.5715
7.5683
7.5652
7.4966
7.4934
7.4799
7.4708
7.4591
7.4560
7.4522
7.4434
7.4399
7.4365
7.4295
7.4270
7.4232
7.4210
7.4157
7.4129
7.4094
7.4003
7.3971
7.3792
7.3678
7.3641
7.3603
7.3496
7.3464
5.0119
4.8432
4.8369
4.4070
4.4010
H2C
O
1.01
4.29
9.32
2.20
1.01
1.09
10.0 9.5 9.0 8.5 8.0 7.5 7.0 6.5 6.0 5.5 5.0 4.5 4.0 3.5 3.0 2.5 2.0 1.5 1.0 0.5 0.0
f1 (ppm)

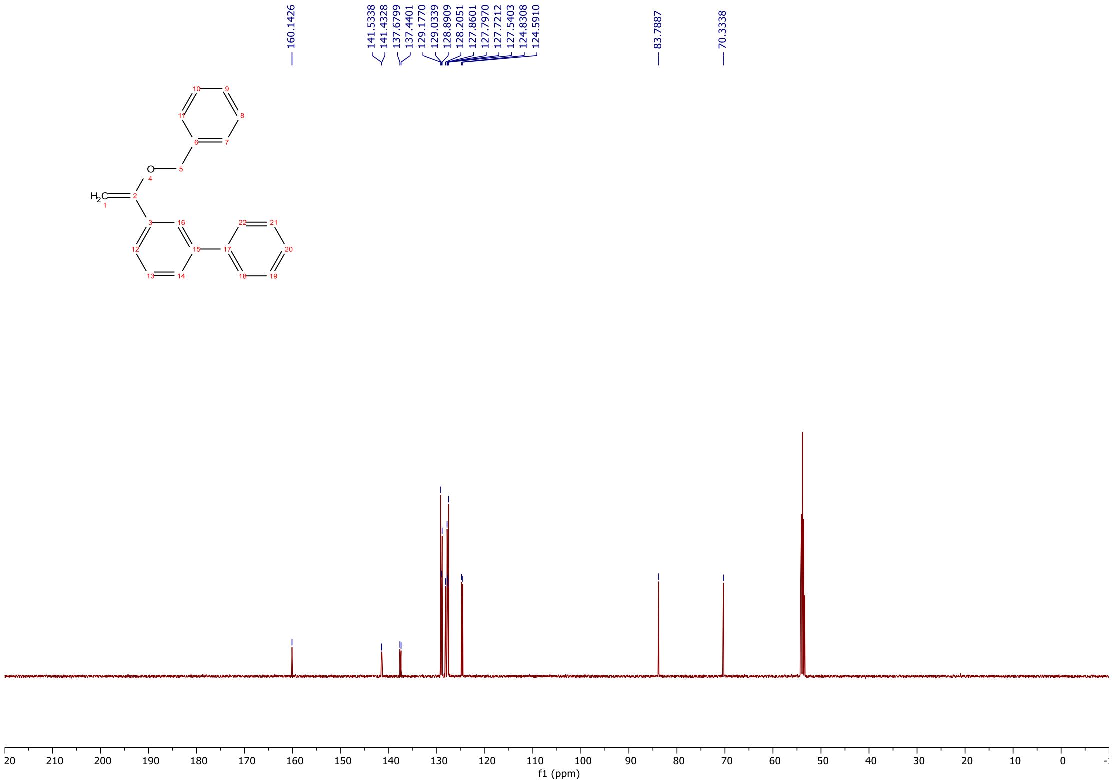
160.1426
141.5338
141.4328
137.6799
137.4401
129.1770
129.0339
128.8909
128.2051
127.8601
127.7970
127.7212
127.5403
124.8308
124.5910
83.7887
70.3338
H2C
O
210 200 190 180 170 160 150 140 130 120 110 100 90 80 70 60 50 40 30 20 10 0
f1 (ppm)

2m

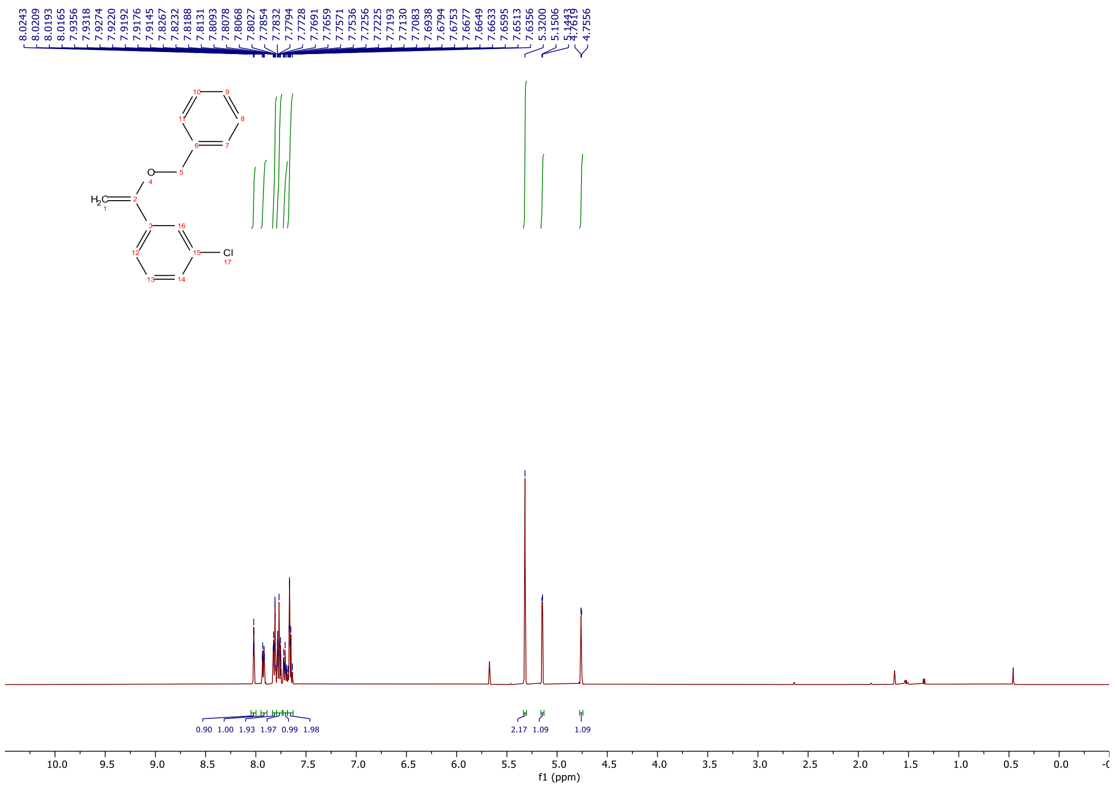
8.0243
8.0209
8.0193
8.0165
7.9356
7.9318
7.9274
7.9220
7.9192
7.9176
7.9145
7.8267
7.8232
7.8188
7.8131
7.8093
7.8078
7.8068
7.8027
7.7854
7.7832
7.7794
7.7728
7.7691
7.7659
7.7571
7.7536
7.7256
7.7225
7.7193
7.7130
7.7083
7.6938
7.6794
7.6753
7.6677
7.6649
7.6633
7.6595
7.6513
7.6356
5.3200
5.1506
5.1443
4.7619
4.7556
O
H2C
Cl
0.90
1.00
1.93
1.97
0.99
1.98
2.17
1.09
1.09
f1 (ppm)

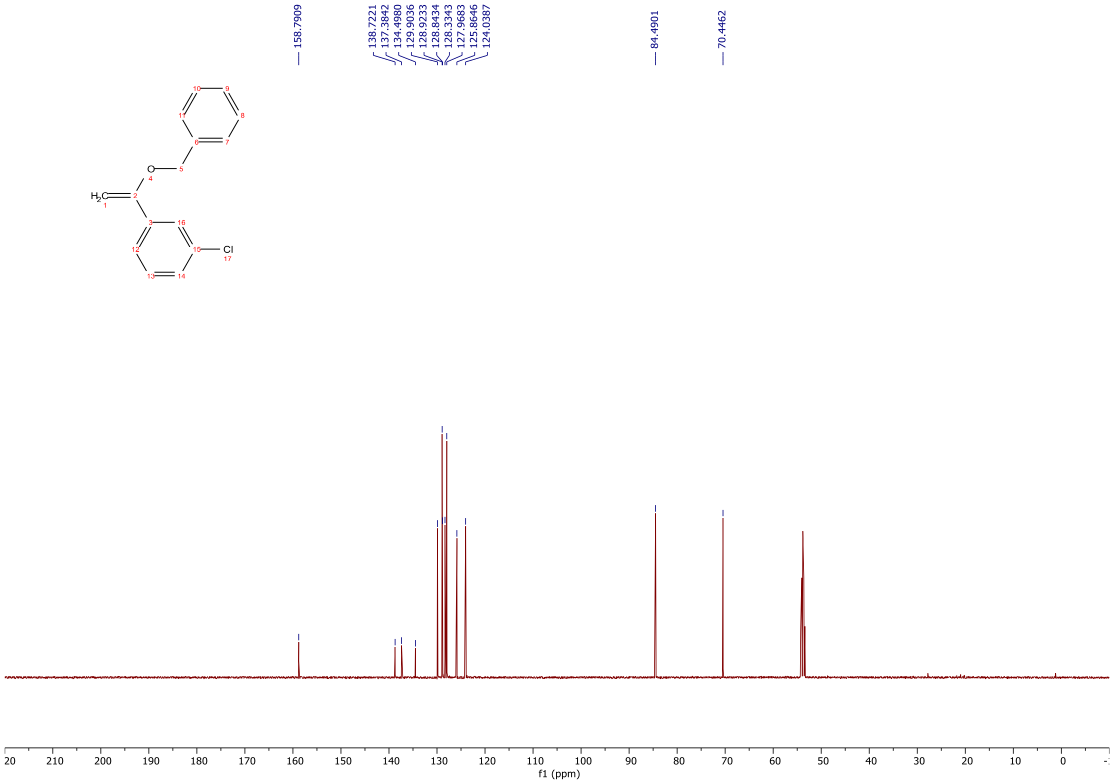
158.7909
138.7221
137.3842
134.4980
129.9036
128.9233
128.8434
128.3343
127.9683
125.8646
124.0387
84.4901
70.4462
O
H2C
Cl
f1 (ppm)

2n

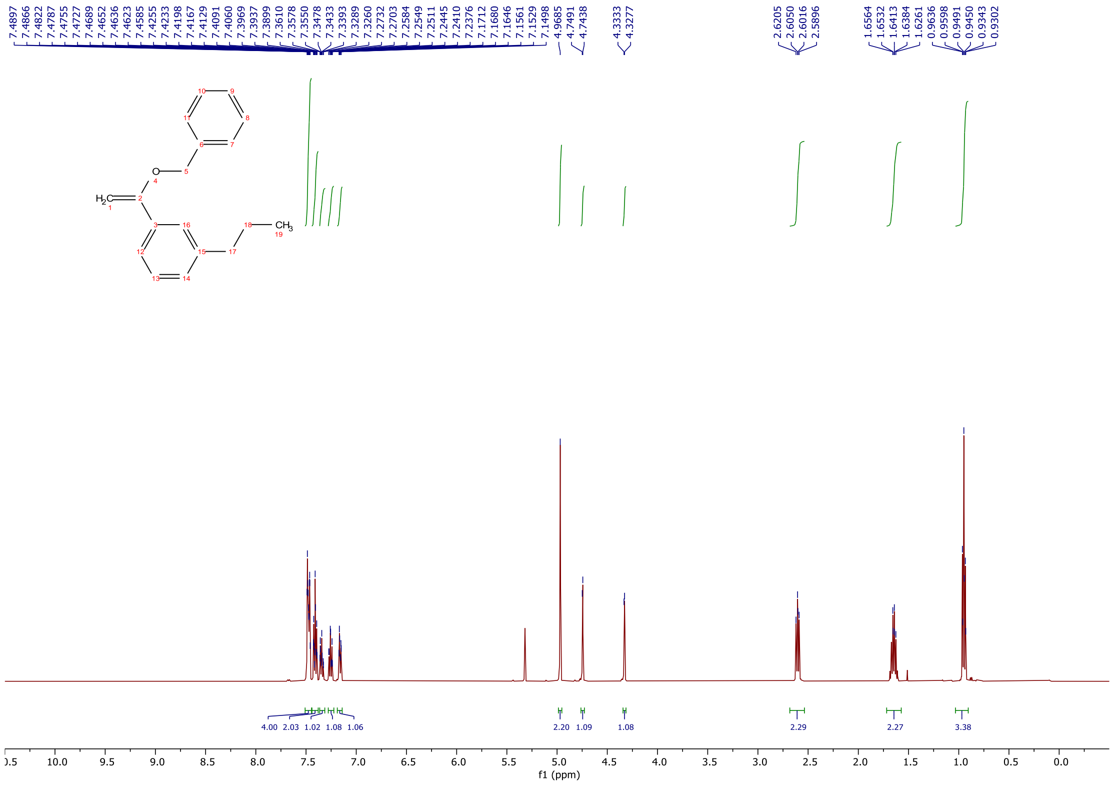
7.4897 7.4866 7.4822 7.4787 7.4755 7.4727 7.4689 7.4652 7.4636 7.4623 7.4585 7.4255 7.4233 7.4198 7.4167 7.4129 7.4091 7.4060 7.3969 7.3937 7.3899 7.3610 7.3578 7.3550 7.3478 7.3433 7.3393 7.3289 7.3260 7.2732 7.2703 7.2584 7.2549 7.2511 7.2445 7.2410 7.2376 7.1712 7.1680 7.1646 7.1561 7.1529 7.1498 4.9685 4.7491 4.7438 4.3333 4.3277
2.6205 2.6050 2.6016 2.5896
1.6564 1.6532 1.6413 1.6384 1.6261 0.9636 0.9598 0.9491 0.9450 0.9343 0.9302
4.00 2.03 1.02 1.08 1.06
2.20 1.09 1.08
2.29 2.27 3.38
f1 (ppm)

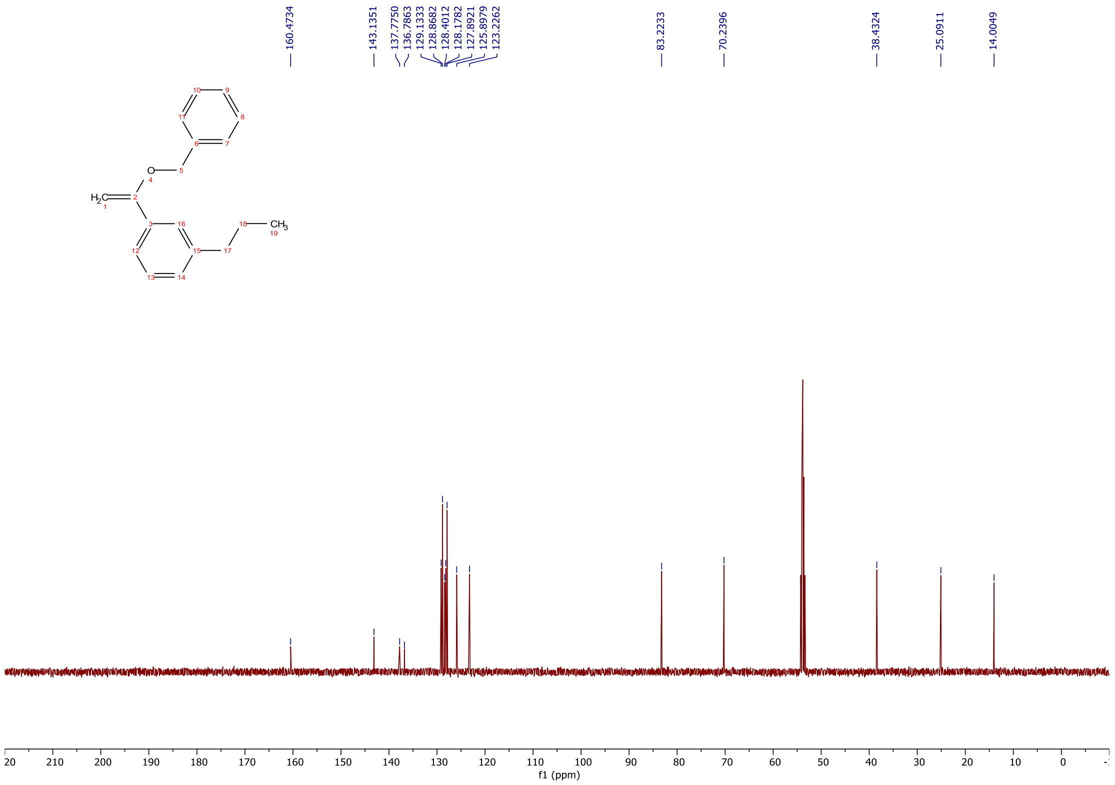
160.4734
143.1351 137.7750 136.7863 129.1333 128.8682 128.4012 128.1782 127.8921 125.8979 123.2262
83.2233
70.2396
38.4324
25.0911
14.0049
f1 (ppm)

2o

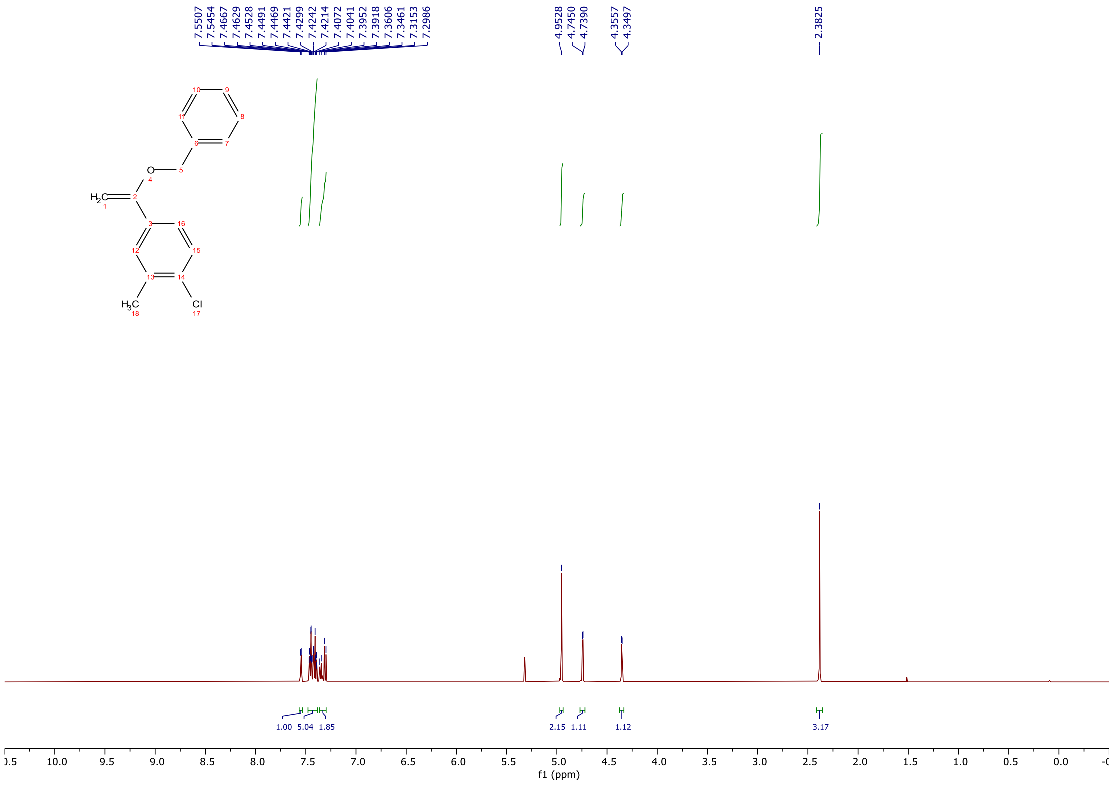
7.5507
7.5454
7.4667
7.4629
7.4528
7.4491
7.4469
7.4421
7.4299
7.4242
7.4214
7.4072
7.4041
7.3952
7.3918
7.3606
7.3461
7.3153
7.2986
4.9528
4.7450
4.7390
4.3557
4.3497
2.3825
H2C
O
H3C
Cl
1.00
5.04
1.85
2.15
1.11
1.12
3.17
f1 (ppm)

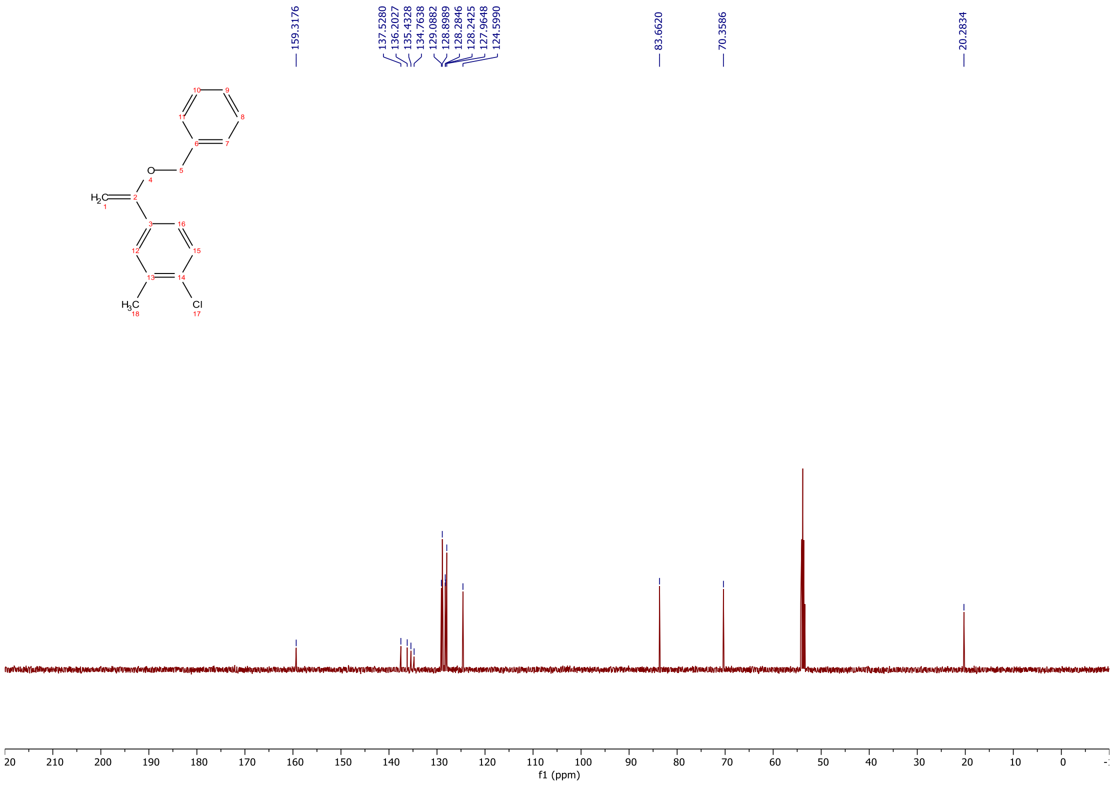
159.3176
137.5280
136.2027
135.4328
134.7638
129.0882
128.8989
128.2846
128.2425
127.9648
124.5990
83.6620
70.3586
20.2834
H2C
O
H3C
Cl
f1 (ppm)

3b

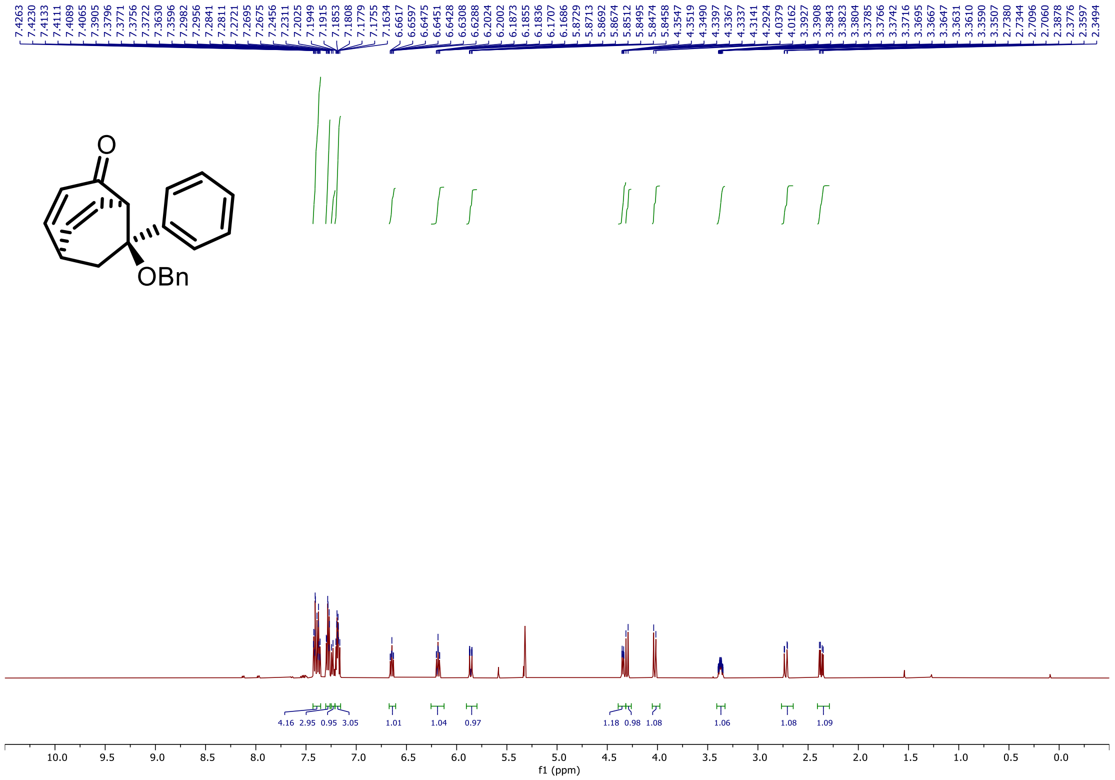

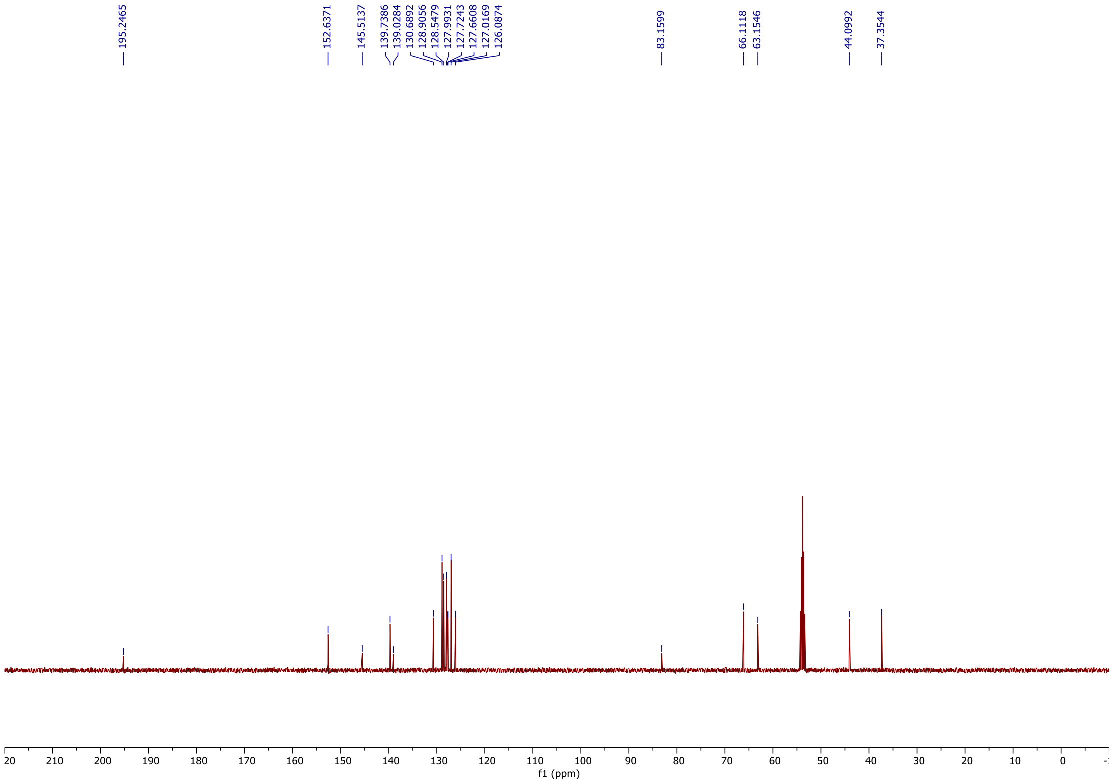

3b-2

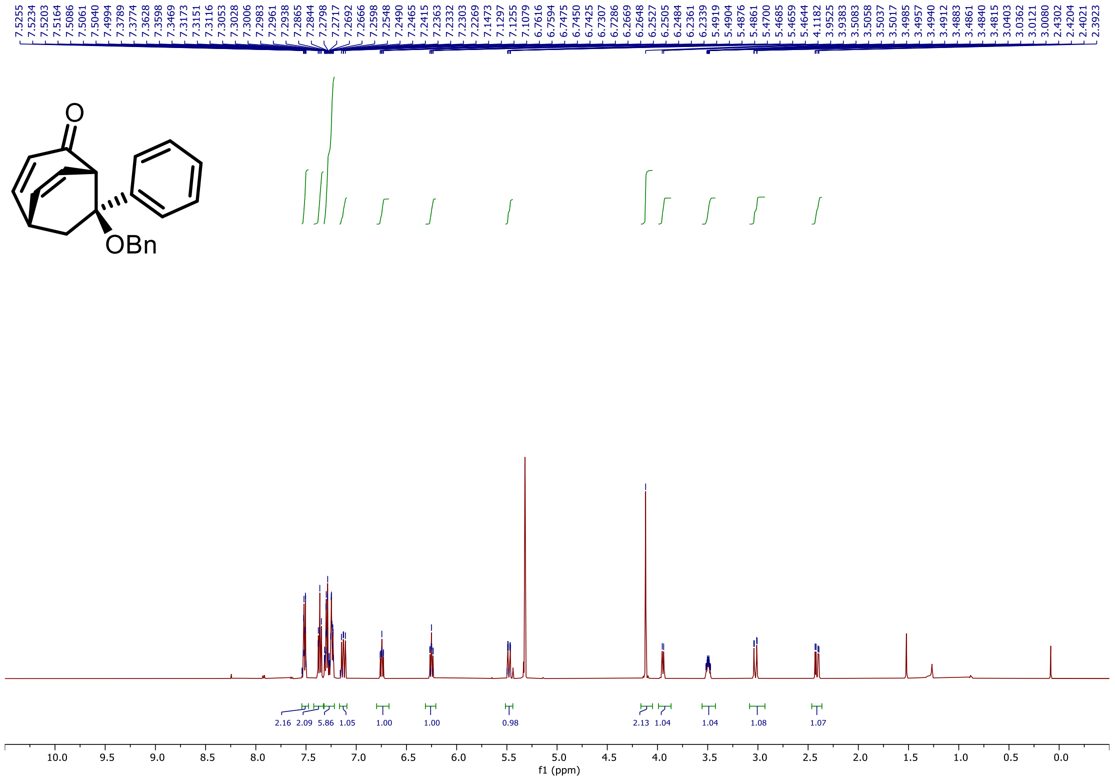
O
OBn
7.5255 7.5234 7.5203 7.5164 7.5086 7.5061 7.5040 7.4994 7.3789 7.3774 7.3628 7.3598 7.3469 7.3173 7.3151 7.3116 7.3053 7.3028 7.3006 7.2983 7.2961 7.2938 7.2865 7.2844 7.2798 7.2717 7.2692 7.2666 7.2598 7.2548 7.2490 7.2465 7.2415 7.2363 7.2332 7.2303 7.2269 7.1473 7.1297 7.1255 7.1079 6.7616 6.7594 6.7475 6.7450 6.7425 6.7307 6.7286 6.2669 6.2648 6.2527 6.2505 6.2484 6.2361 6.2339 5.4919 5.4904 5.4876 5.4861 5.4700 5.4685 5.4659 5.4644 4.1182 3.9525 3.9383 3.5083 3.5058 3.5037 3.5017 3.4985 3.4957 3.4940 3.4912 3.4883 3.4861 3.4840 3.4815 3.0403 3.0362 3.0121 3.0080 2.4302 2.4204 2.4021 2.3923
2.16 2.09 5.86 1.05 1.00 1.00 0.98 2.13 1.04 1.04 1.08 1.07
10.0 9.5 9.0 8.5 8.0 7.5 7.0 6.5 6.0 5.5 5.0 4.5 4.0 3.5 3.0 2.5 2.0 1.5 1.0 0.5 0.0
f1 (ppm)

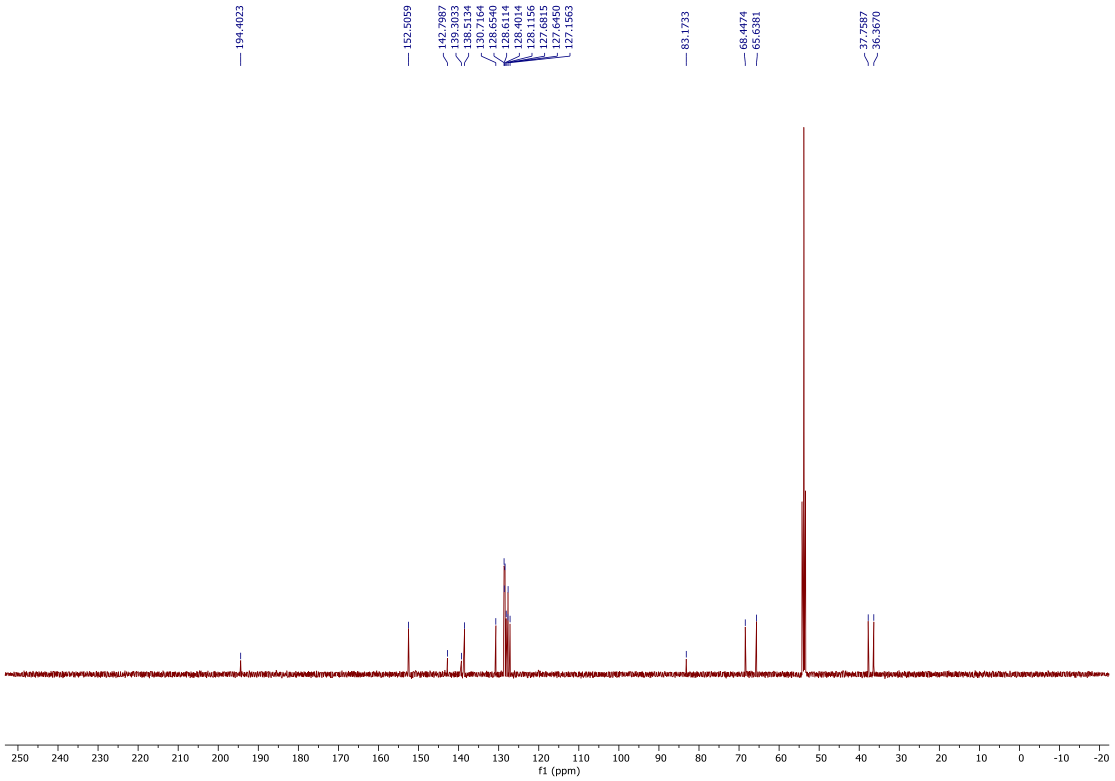
194.4023
152.5059 142.7987 139.3033 138.5134 130.7164 128.6540 128.6114 128.4014 128.1156 127.6815 127.6450 127.1563
83.1733
68.4474 65.6381
37.7587 36.3670
250 240 230 220 210 200 190 180 170 160 150 140 130 120 110 100 90 80 70 60 50 40 30 20 10 0 -10 -20
f1 (ppm)

3c

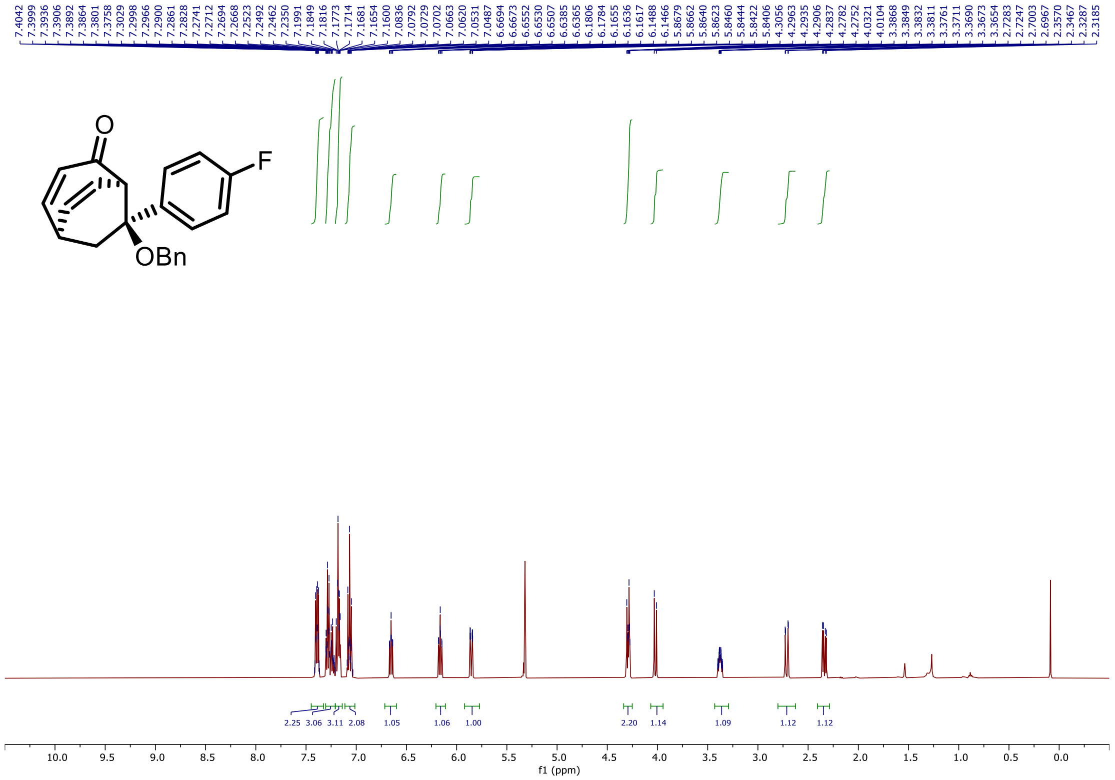

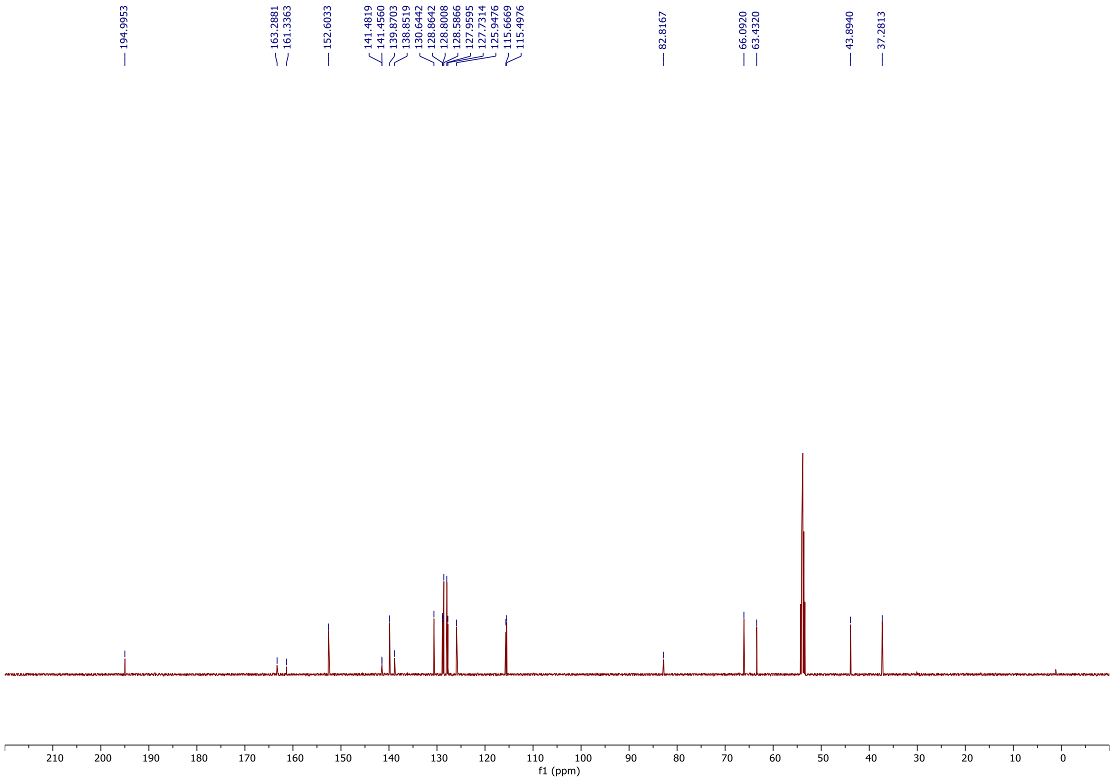

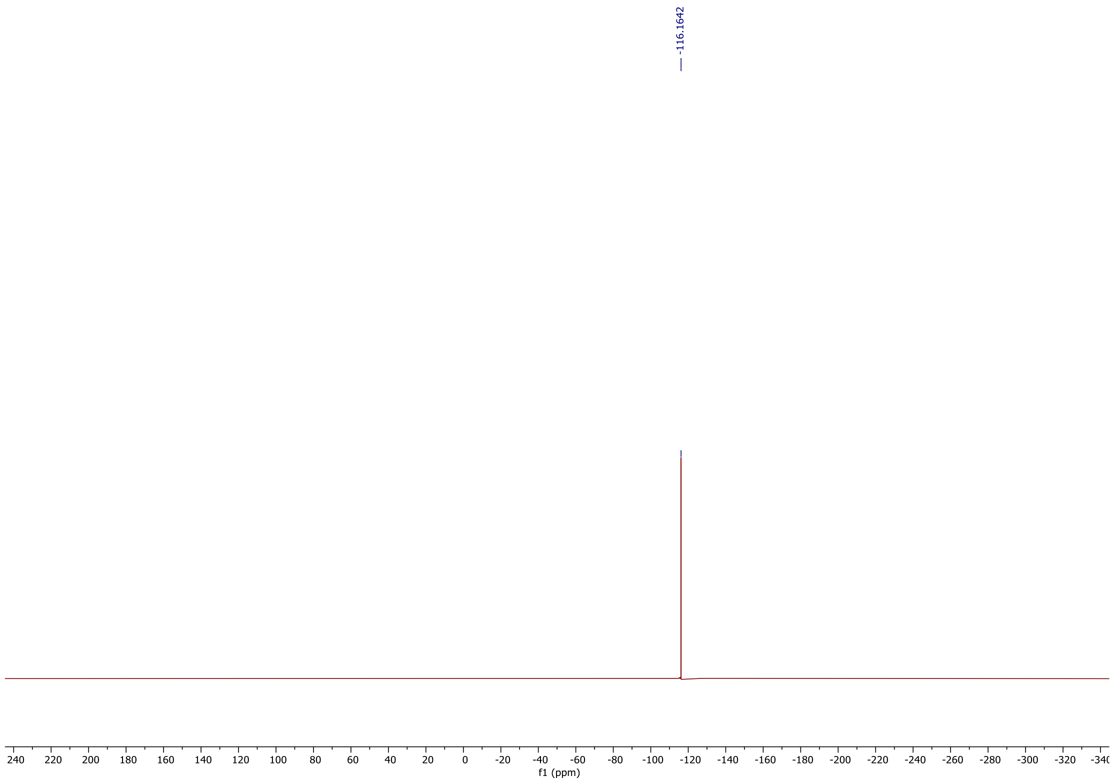
-116.1642
240 220 200 180 160 140 120 100 80 60 40 20 0 -20 -40 -60 -80 -100 -120 -140 -160 -180 -200 -220 -240 -260 -280 -300 -320 -340
f1 (ppm)

3c-2

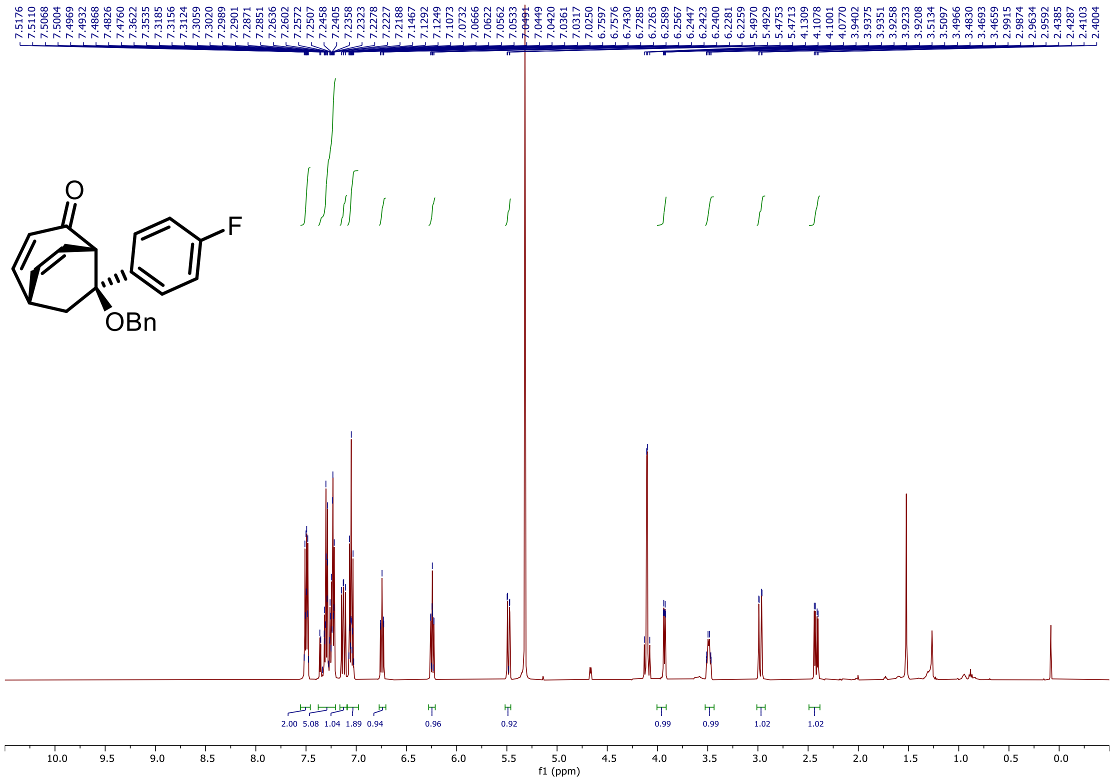
O
F
OBn
7.5176
7.5110
7.5068
7.5004
7.4969
7.4932
7.4868
7.4826
7.4760
7.3622
7.3535
7.3185
7.3156
7.3124
7.3059
7.3020
7.2989
7.2901
7.2871
7.2851
7.2636
7.2602
7.2572
7.2507
7.2458
7.2405
7.2358
7.2323
7.2278
7.2227
7.2188
7.1467
7.1292
7.1249
7.1073
7.0732
7.0666
7.0622
7.0562
7.0533
7.0491
7.0449
7.0420
7.0361
7.0317
7.0250
6.7597
6.7576
6.7430
6.7285
6.7263
6.2589
6.2567
6.2447
6.2423
6.2400
6.2281
6.2259
5.4970
5.4929
5.4753
5.4713
4.1309
4.1078
4.1001
4.0770
3.9402
3.9375
3.9351
3.9258
3.9233
3.9208
3.5134
3.5097
3.4966
3.4830
3.4693
3.4659
2.9915
2.9874
2.9634
2.9592
2.4385
2.4287
2.4103
2.4004
2.00
5.08
1.04
1.89
0.94
0.96
0.92
0.99
0.99
1.02
1.02
10.0
9.5
9.0
8.5
8.0
7.5
7.0
6.5
6.0
5.5
5.0
4.5
4.0
3.5
3.0
2.5
2.0
1.5
1.0
0.5
0.0
f1 (ppm)

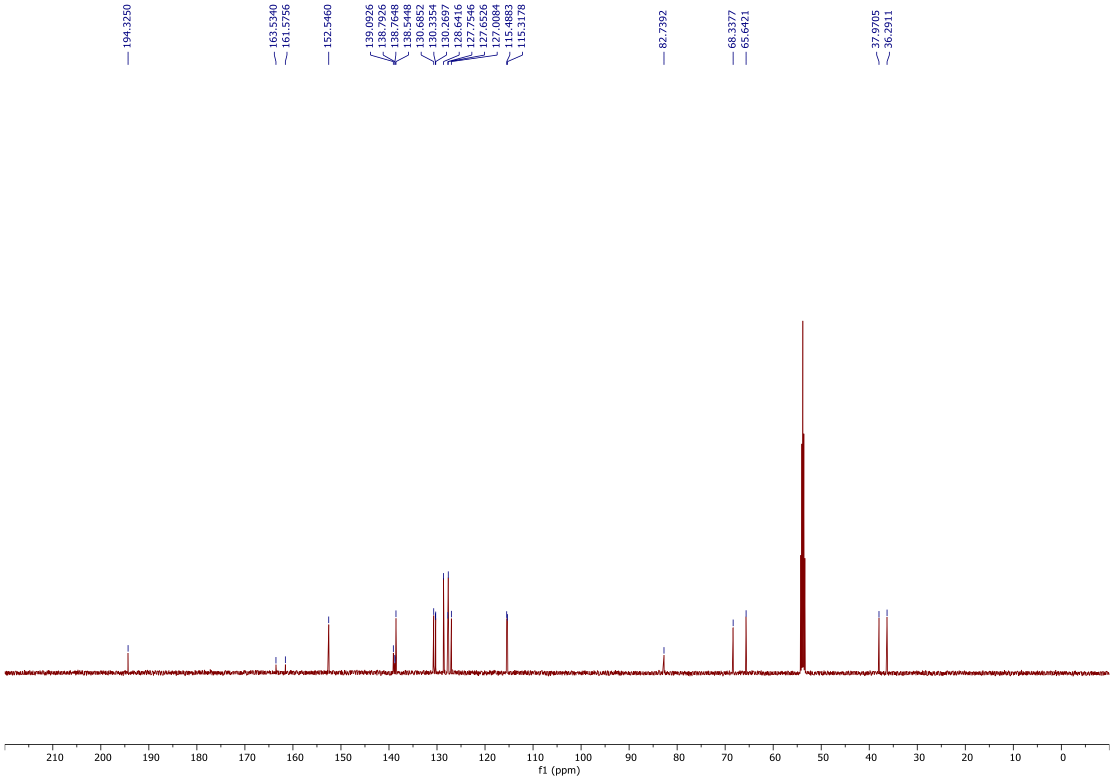
194.3250
163.5340
161.5756
152.5460
139.0926
138.7926
138.7648
138.5448
130.6852
130.3354
130.2697
128.6416
127.7546
127.6526
127.0084
115.4883
115.3178
82.7392
68.3377
65.6421
37.9705
36.2911
210
200
190
180
170
160
150
140
130
120
110
100
90
80
70
60
50
40
30
20
10
0
f1 (ppm)

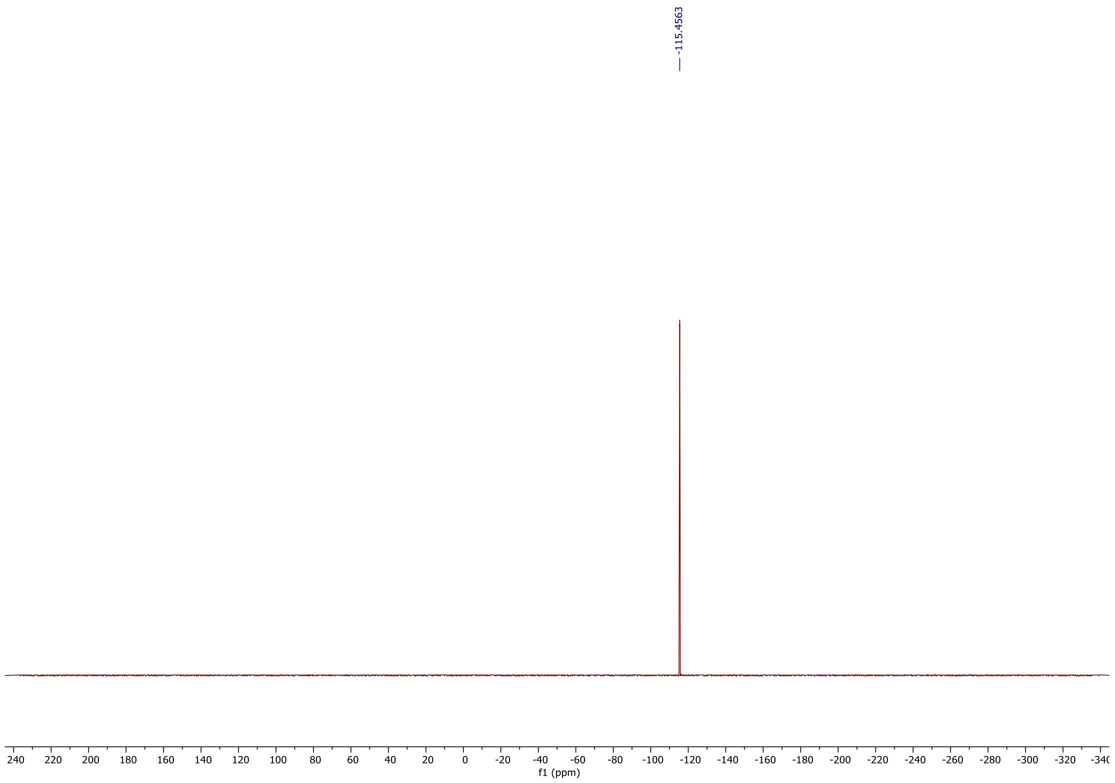
-115.4563
240 220 200 180 160 140 120 100 80 60 40 20 0 -20 -40 -60 -80 -100 -120 -140 -160 -180 -200 -220 -240 -260 -280 -300 -320 -340
f1 (ppm)

3d

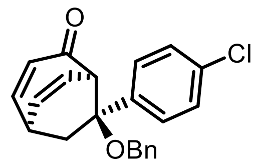
O
Cl
OBn

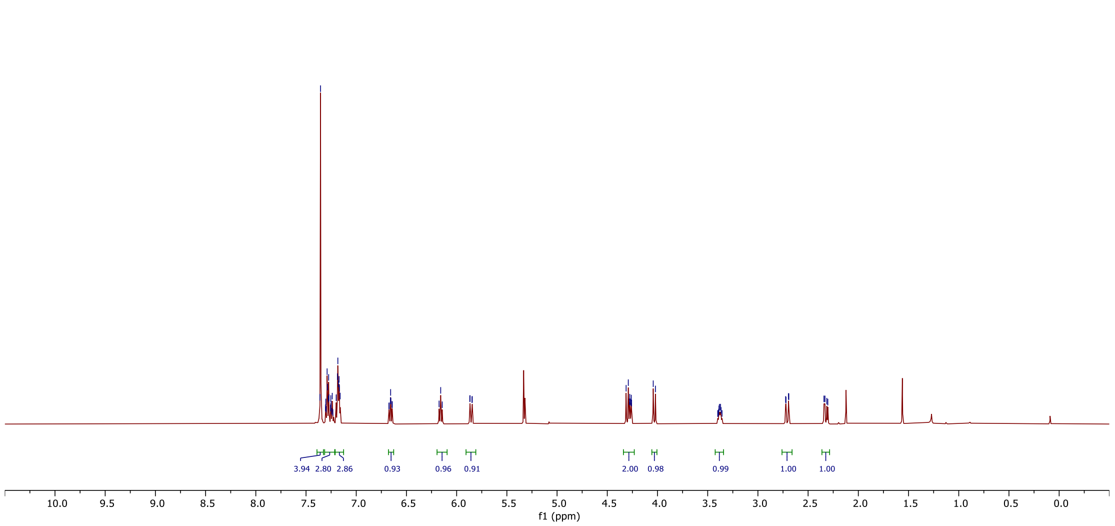
7.3611
7.3555
7.3064
7.3033
7.3001
7.2899
7.2866
7.2778
7.2747
7.2721
7.2564
7.2532
7.2501
7.2438
7.2389
7.2331
7.1994
7.1872
7.1832
7.1778
7.1706
7.1679
7.1603
6.6740
6.6719
6.6596
6.6574
6.6552
6.6429
6.6410
6.1748
6.1590
6.1433
5.8688
5.8668
5.8470
5.8447
4.3124
4.2907
4.2788
4.2760
4.2731
4.2638
4.2609
4.2580
4.0423
4.0205
3.4000
3.3970
3.3908
3.3876
3.3833
3.3724
3.3670
3.3570
2.7240
2.7201
2.6958
2.6921
2.3424
2.3321
2.3141
2.3039
3.94
2.80
2.86
0.93
0.96
0.91
2.00
0.98
0.99
1.00
1.00
10.0
9.5
9.0
8.5
8.0
7.5
7.0
6.5
6.0
5.5
5.0
4.5
4.0
3.5
3.0
2.5
2.0
1.5
1.0
0.5
0.0
f1 (ppm)

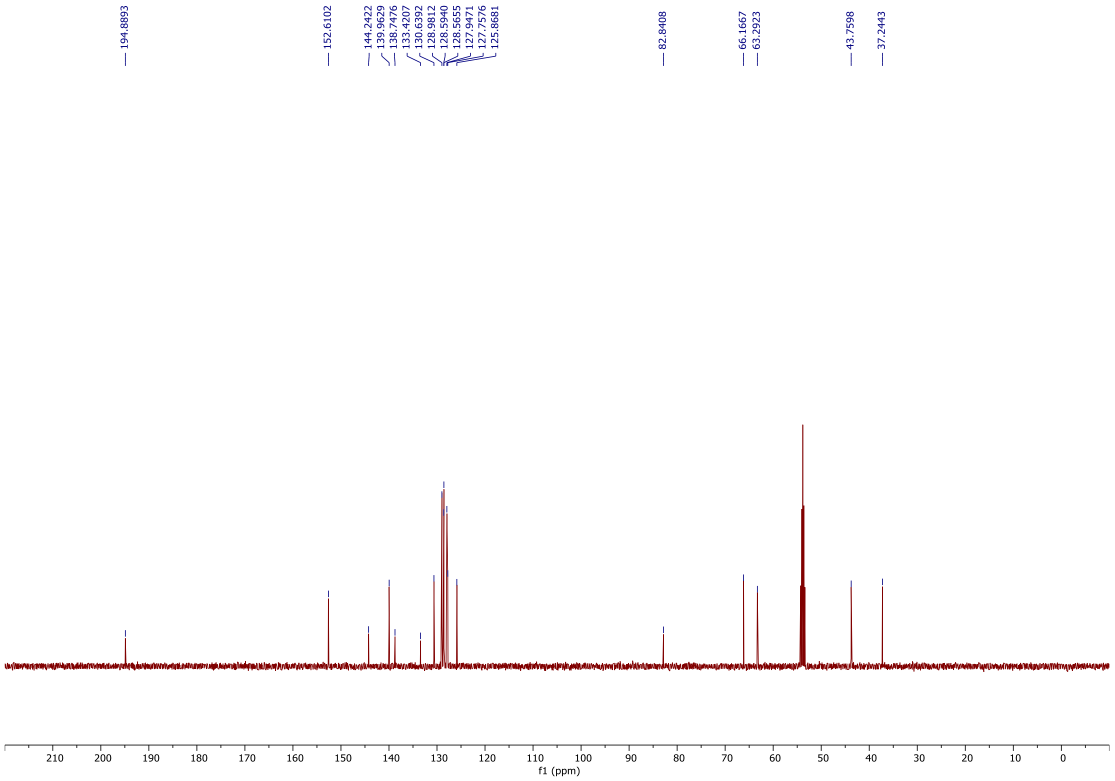
194.8893
152.6102
144.2422
139.9629
138.7476
133.4207
130.6392
128.9812
128.5940
128.5655
127.9471
127.7576
125.8681
82.8408
66.1667
63.2923
43.7598
37.2443
210
200
190
180
170
160
150
140
130
120
110
100
90
80
70
60
50
40
30
20
10
0
f1 (ppm)

3d-2

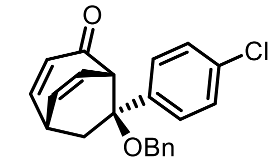
O
Cl
OBn

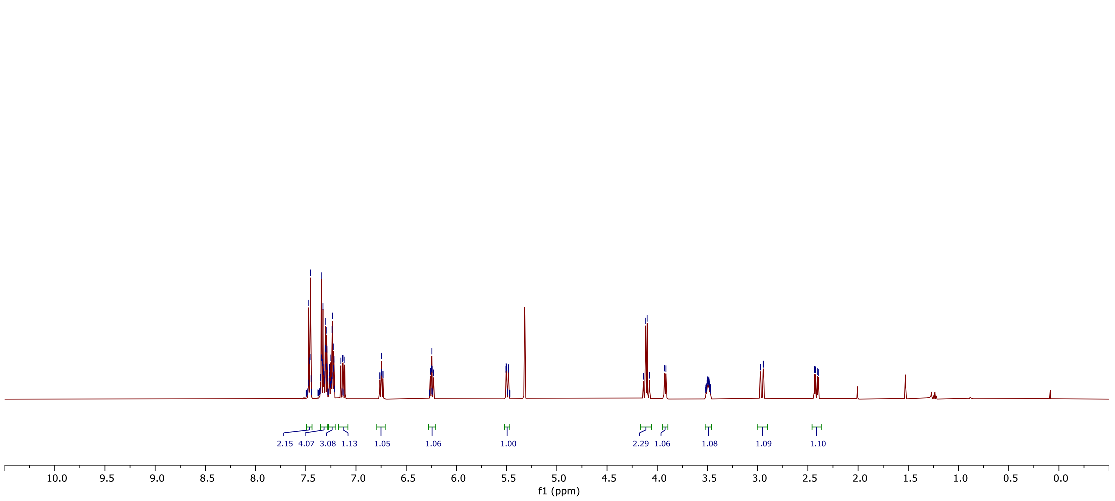
7.4758 7.4701 7.4660 7.4570 7.4528 7.4472 7.3514 7.3460 7.3418 7.3327 7.3287 7.3228 7.3196 7.3165 7.3096 7.3060 7.3030 7.2939 7.2911 7.2891 7.2866 7.2677 7.2646 7.2616 7.2552 7.2502 7.2447 7.2384 7.2350 7.2304 7.2248 7.2216 7.2190 7.1504 7.1330 7.1287 7.1111 6.7630 6.7609 6.7489 6.7465 6.7441 6.7322 6.7300 6.2608 6.2586 6.2465 6.2442 6.2420 6.2300 6.2278 5.5066 5.5051 5.5026 5.5011 5.4849 5.4834 5.4808 5.4794 4.1376 4.1146 4.1008 4.0777 3.9279 3.9134 3.5135 3.5061 3.5035 3.5014 3.4991 3.4960 3.4936 3.4918 3.4890 3.4856 3.4834 3.4817 3.4794 2.9738 2.9696 2.9455 2.9414 2.4344 2.4247 2.4062 2.3964
2.15 4.07 3.08 1.13 1.05 1.06 1.00 2.29 1.06 1.08 1.09 1.10
10.0 9.5 9.0 8.5 8.0 7.5 7.0 6.5 6.0 5.5 5.0 4.5 4.0 3.5 3.0 2.5 2.0 1.5 1.0 0.5 0.0
f1 (ppm)

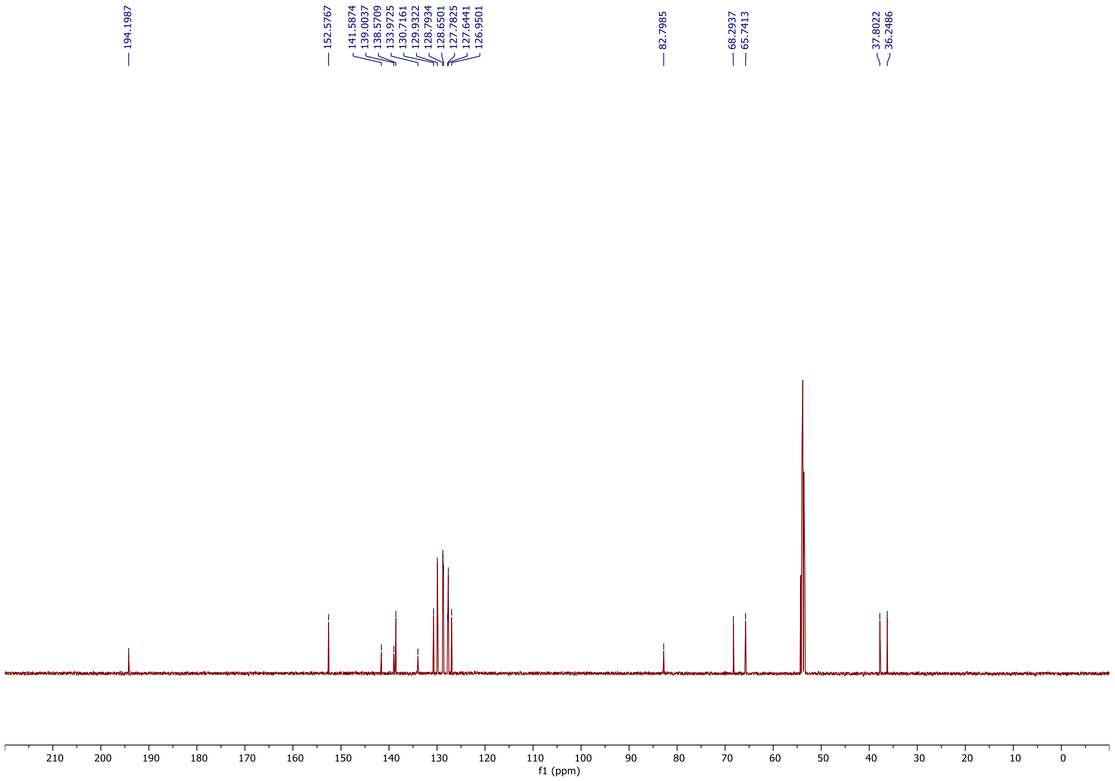
194.1987
152.5767 141.5874 139.0037 138.5709 133.9725 130.7161 129.9322 128.7934 128.6501 127.7825 127.6441 126.9501
82.7985
68.2937 65.7413
37.8022 36.2486
210 200 190 180 170 160 150 140 130 120 110 100 90 80 70 60 50 40 30 20 10 0
f1 (ppm)

3e

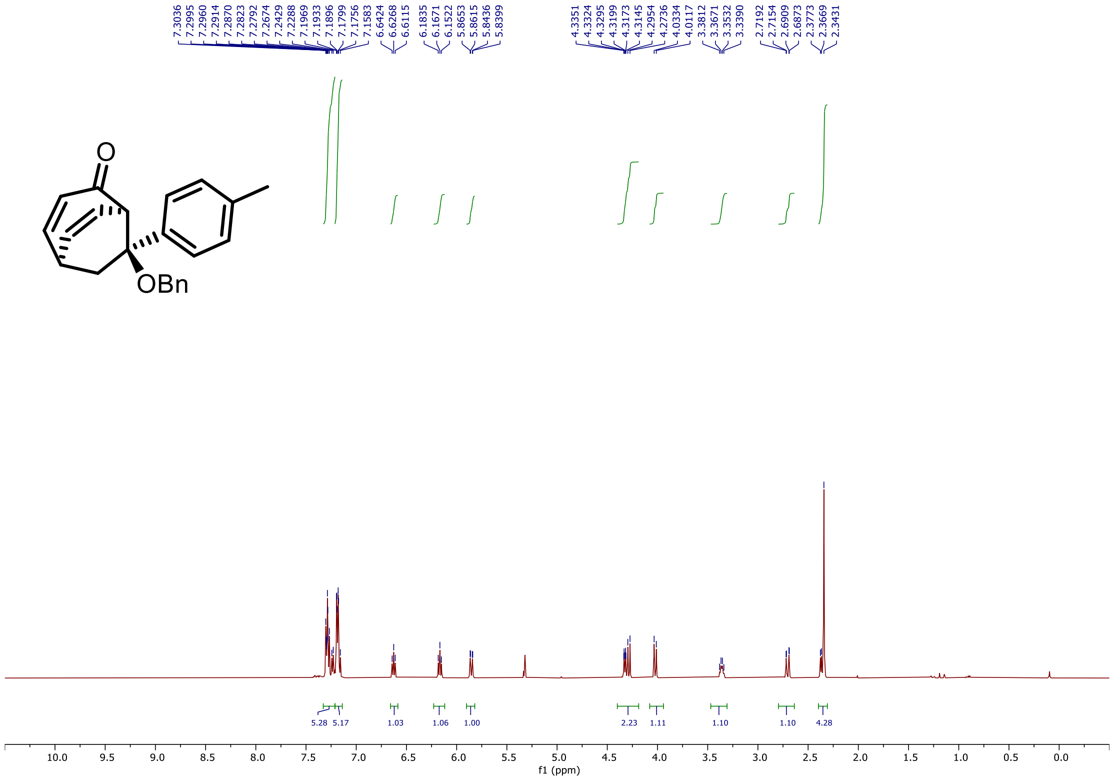

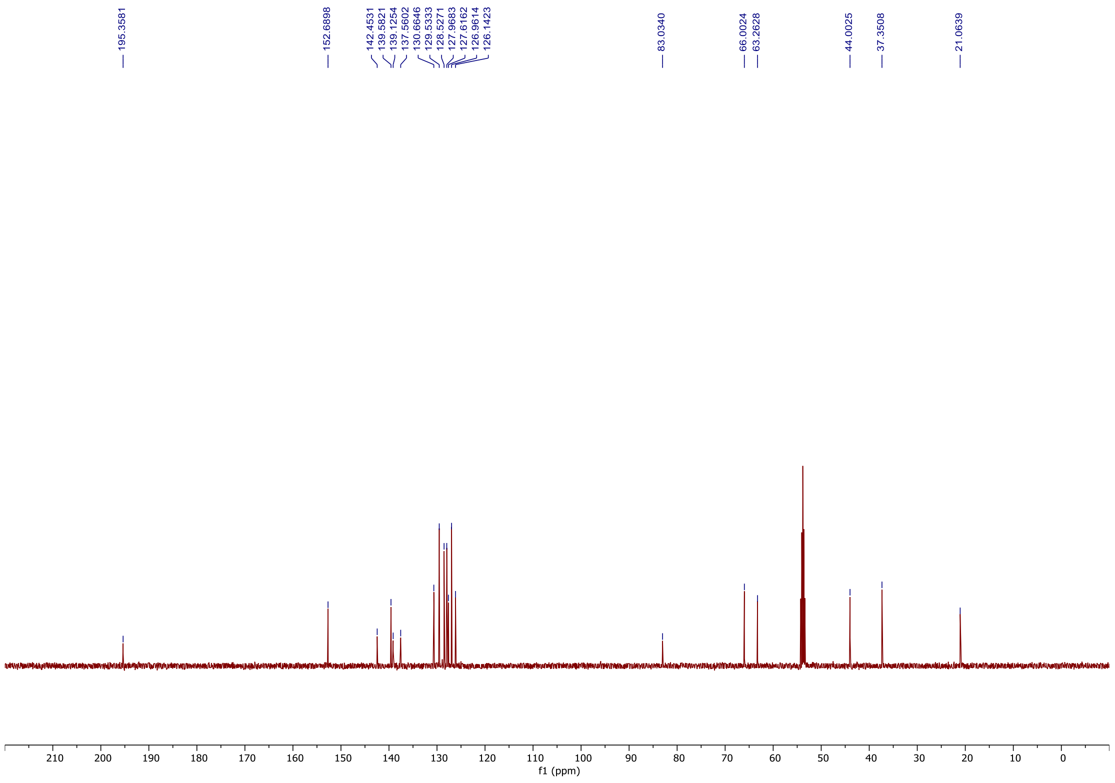

3e-2

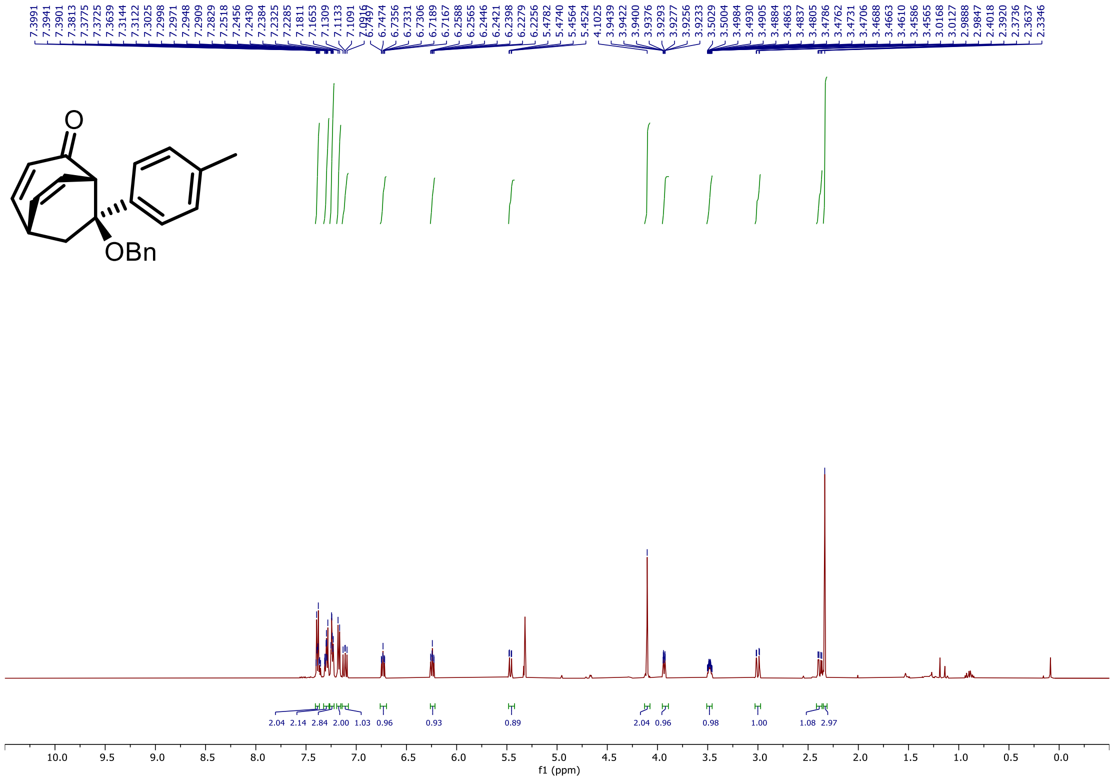

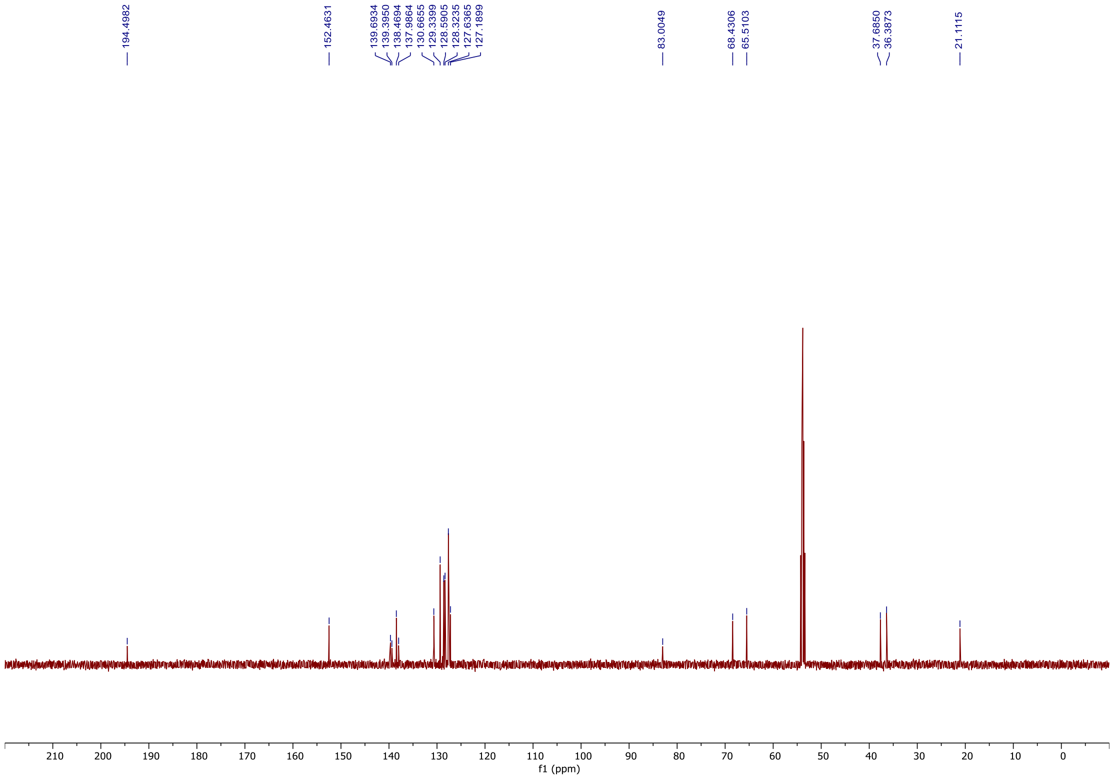

3f

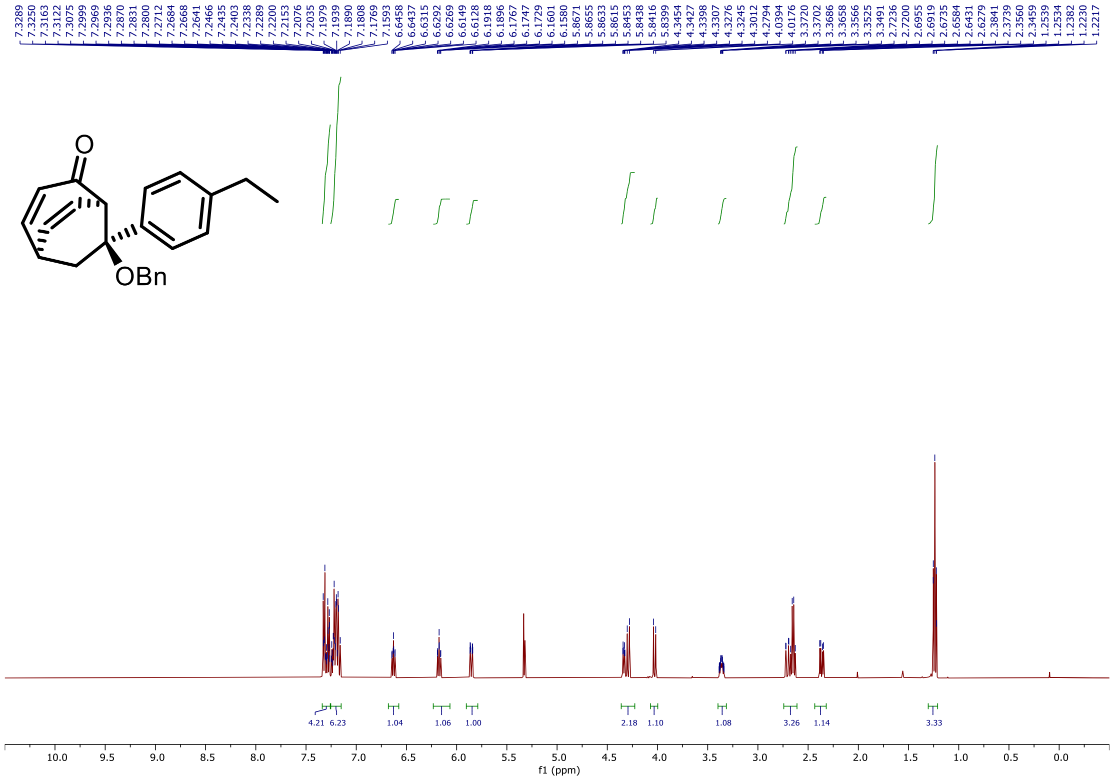
7.3289
7.3250
7.3163
7.3122
7.3075
7.2999
7.2969
7.2936
7.2870
7.2831
7.2800
7.2712
7.2684
7.2668
7.2641
7.2466
7.2435
7.2403
7.2338
7.2289
7.2200
7.2153
7.2076
7.2035
7.1979
7.1939
7.1890
7.1808
7.1769
7.1593
6.6458
6.6437
6.6315
6.6292
6.6269
6.6149
6.6128
6.1918
6.1896
6.1767
6.1747
6.1729
6.1601
6.1580
5.8671
5.8655
5.8633
5.8615
5.8453
5.8438
5.8416
5.8399
4.3454
4.3427
4.3398
4.3307
4.3276
4.3245
4.3012
4.2794
4.0394
4.0176
3.3720
3.3702
3.3686
3.3658
3.3566
3.3525
3.3491
2.7236
2.7200
2.6955
2.6919
2.6735
2.6584
2.6431
2.6279
2.3841
2.3739
2.3560
2.3459
1.2539
1.2534
1.2382
1.2230
1.2217
O
OBn
4.21
6.23
1.04
1.06
1.00
2.18
1.10
1.08
3.26
1.14
3.33
10.0
9.5
9.0
8.5
8.0
7.5
7.0
6.5
6.0
5.5
5.0
4.5
4.0
3.5
3.0
2.5
2.0
1.5
1.0
0.5
0.0
f1 (ppm)

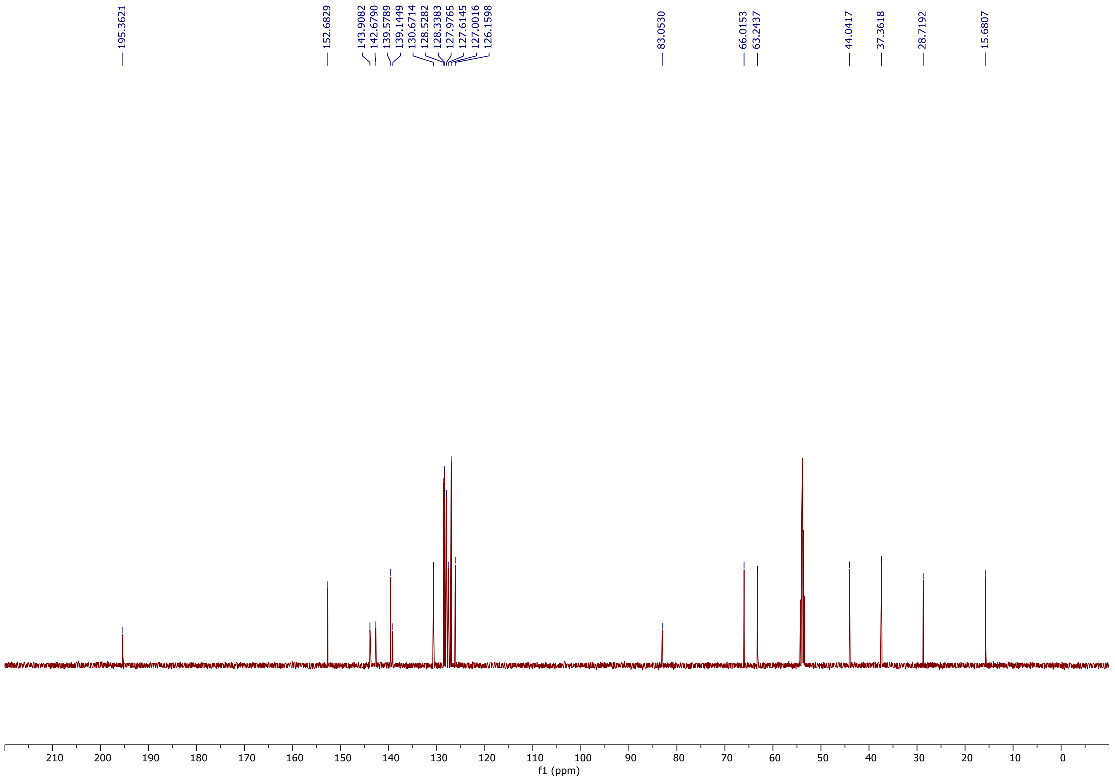
195.3621
152.6829
143.9082
142.6790
139.5799
139.1449
130.6714
128.5282
128.3383
127.9765
127.6145
127.0016
126.1598
83.0530
66.0153
63.2437
44.0417
37.3618
28.7192
15.6807
210
200
190
180
170
160
150
140
130
120
110
100
90
80
70
60
50
40
30
20
10
0
f1 (ppm)

3f-2

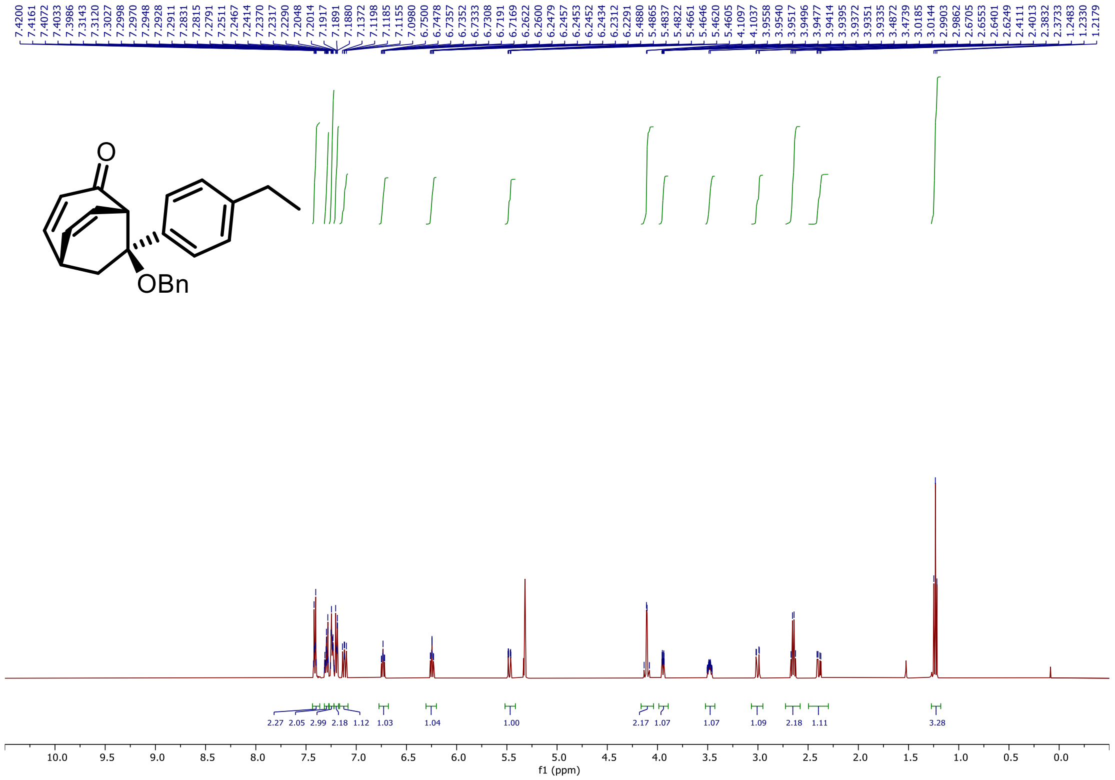
O
OBn
7.4200 7.4161 7.4072 7.4033 7.3986 7.3143 7.3120 7.3027 7.2998 7.2970 7.2948 7.2928 7.2911 7.2831 7.2815 7.2791 7.2511 7.2467 7.2414 7.2370 7.2317 7.2290 7.2048 7.2014 7.1917 7.1891 7.1880 7.1372 7.1198 7.1185 7.1155 7.0980 6.7500 6.7478 6.7357 6.7352 6.7333 6.7308 6.7191 6.7169 6.2622 6.2600 6.2479 6.2457 6.2453 6.2452 6.2434 6.2312 6.2291 5.4880 5.4865 5.4837 5.4822 5.4661 5.4646 5.4620 5.4605 4.1097 4.1037 3.9558 3.9540 3.9517 3.9496 3.9477 3.9414 3.9395 3.9372 3.9351 3.9335 3.4872 3.4739 3.0185 3.0144 2.9903 2.9862 2.6705 2.6553 2.6401 2.6249 2.4111 2.4013 2.3832 2.3733 1.2483 1.2330 1.2179
2.27 2.05 2.99 2.18 1.12 1.03 1.04 1.00 2.17 1.07 1.07 1.09 2.18 1.11 3.28
10.0 9.5 9.0 8.5 8.0 7.5 7.0 6.5 6.0 5.5 5.0 4.5 4.0 3.5 3.0 2.5 2.0 1.5 1.0 0.5 0.0
f1 (ppm)

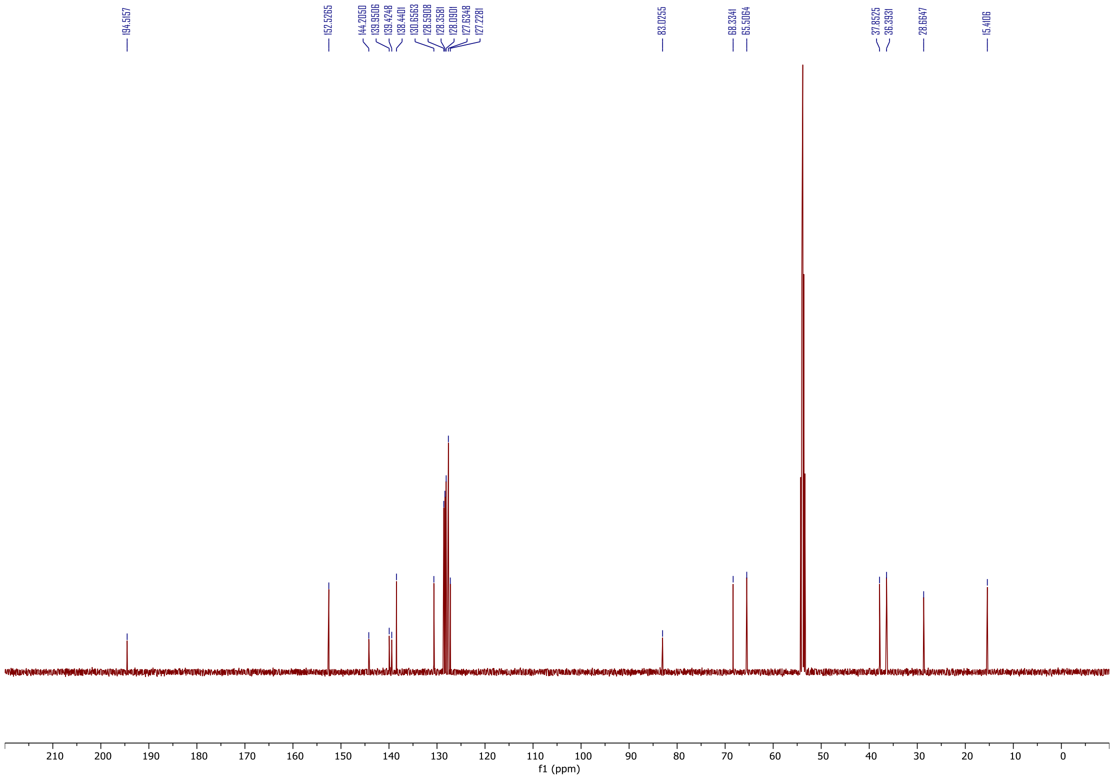
194.5157
152.5265
144.2050 139.9506 139.4248 138.4401 130.6563 128.5808 128.3581 128.0801 127.6348 127.2281
83.0255
68.3341 65.5064
37.8525 36.3931
28.6547
15.4106
210 200 190 180 170 160 150 140 130 120 110 100 90 80 70 60 50 40 30 20 10 0
f1 (ppm)

3g

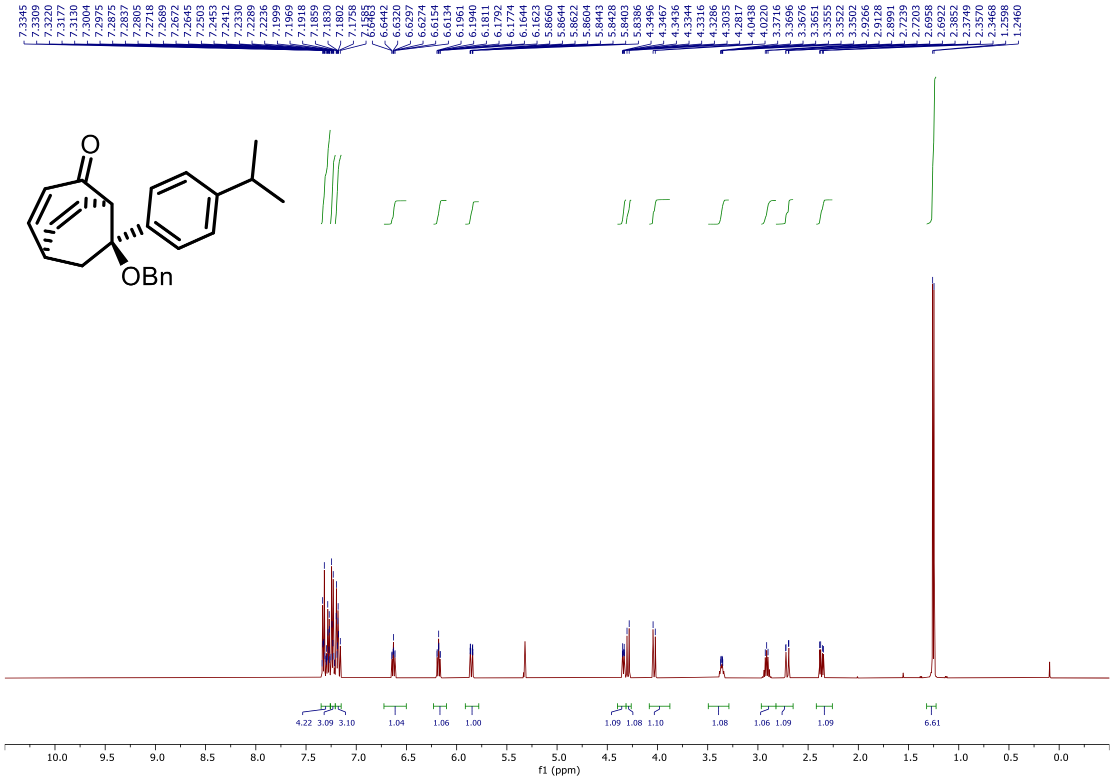

7.3345
7.3309
7.3220
7.3177
7.3130
7.3004
7.2975
7.2875
7.2837
7.2805
7.2718
7.2689
7.2672
7.2645
7.2503
7.2453
7.2412
7.2330
7.2289
7.2236
7.1999
7.1969
7.1918
7.1859
7.1830
7.1802
7.1758
7.1585
6.6463
6.6442
6.6320
6.6297
6.6274
6.6154
6.6134
6.1961
6.1940
6.1811
6.1792
6.1774
6.1644
6.1623
5.8660
5.8644
5.8622
5.8604
5.8443
5.8428
5.8403
5.8386
4.3496
4.3467
4.3436
4.3344
4.3316
4.3286
4.3035
4.2817
4.0438
4.0220
3.3716
3.3696
3.3676
3.3651
3.3555
3.3522
3.3502
2.9266
2.9128
2.8991
2.7239
2.7203
2.6958
2.6922
2.3852
2.3749
2.3570
2.3468
1.2598
1.2460
4.22 3.09 3.10
1.04
1.06
1.00
1.09 1.08 1.10
1.08
1.06 1.09
1.09
6.61
10.0 9.5 9.0 8.5 8.0 7.5 7.0 6.5 6.0 5.5 5.0 4.5 4.0 3.5 3.0 2.5 2.0 1.5 1.0 0.5 0.0
f1 (ppm)

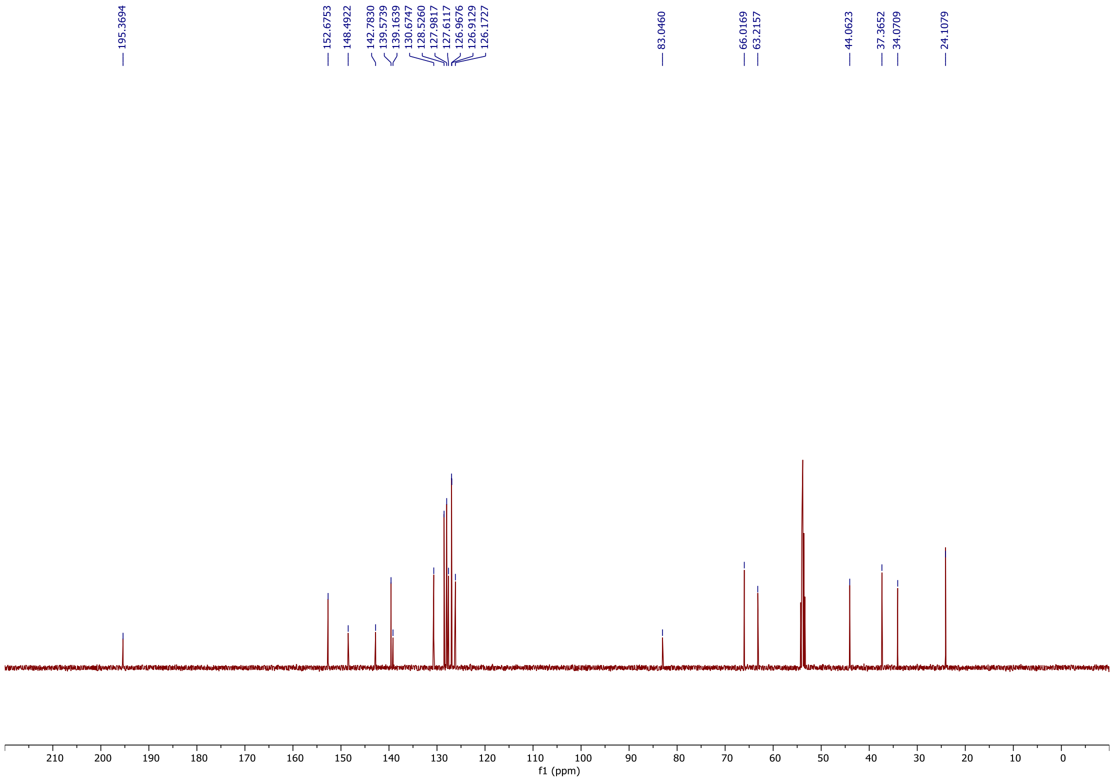

195.3694
152.6753
148.4922
142.7830
139.5739
139.1639
130.6747
128.5260
127.9817
127.6117
126.9676
126.9129
126.1727
83.0460
66.0169
63.2157
44.0623
37.3652
34.0709
24.1079
210 200 190 180 170 160 150 140 130 120 110 100 90 80 70 60 50 40 30 20 10 0
f1 (ppm)

3g-2

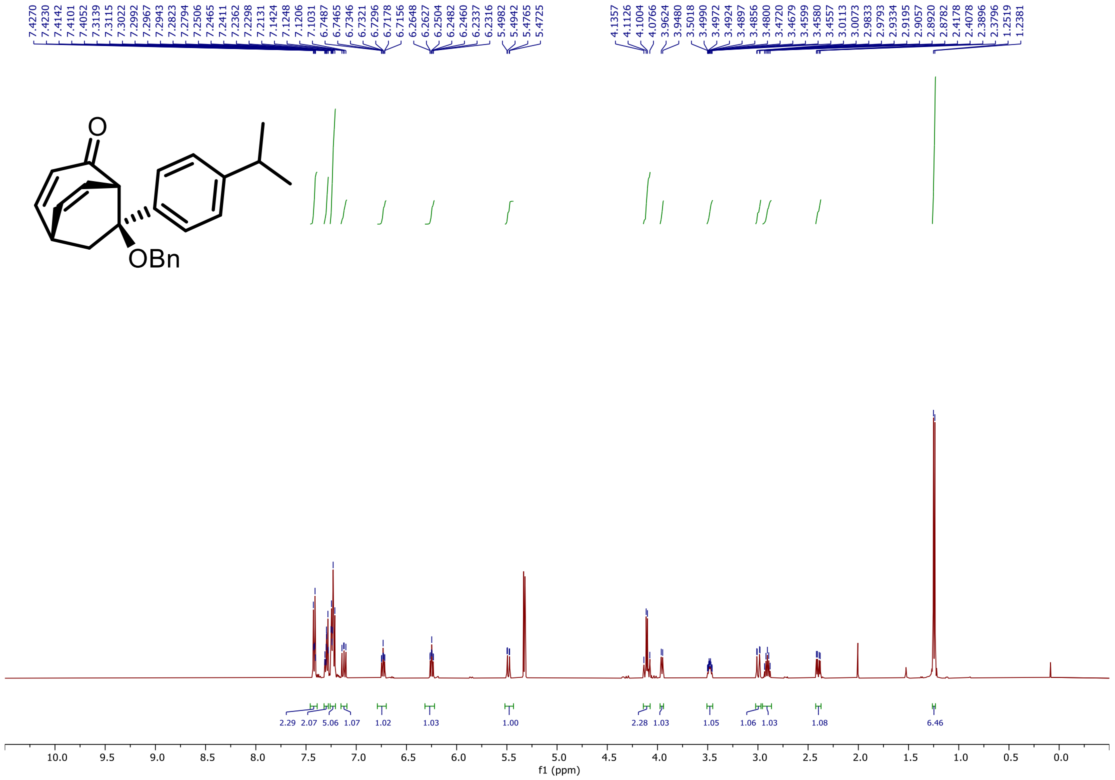

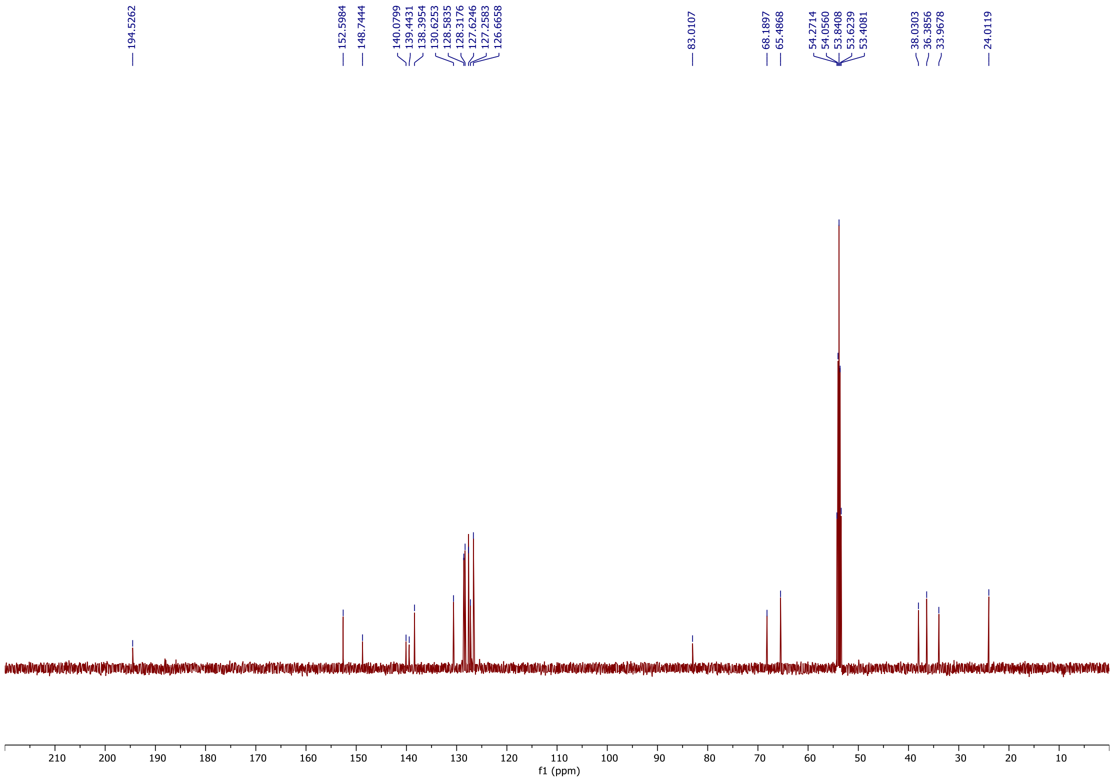

3h

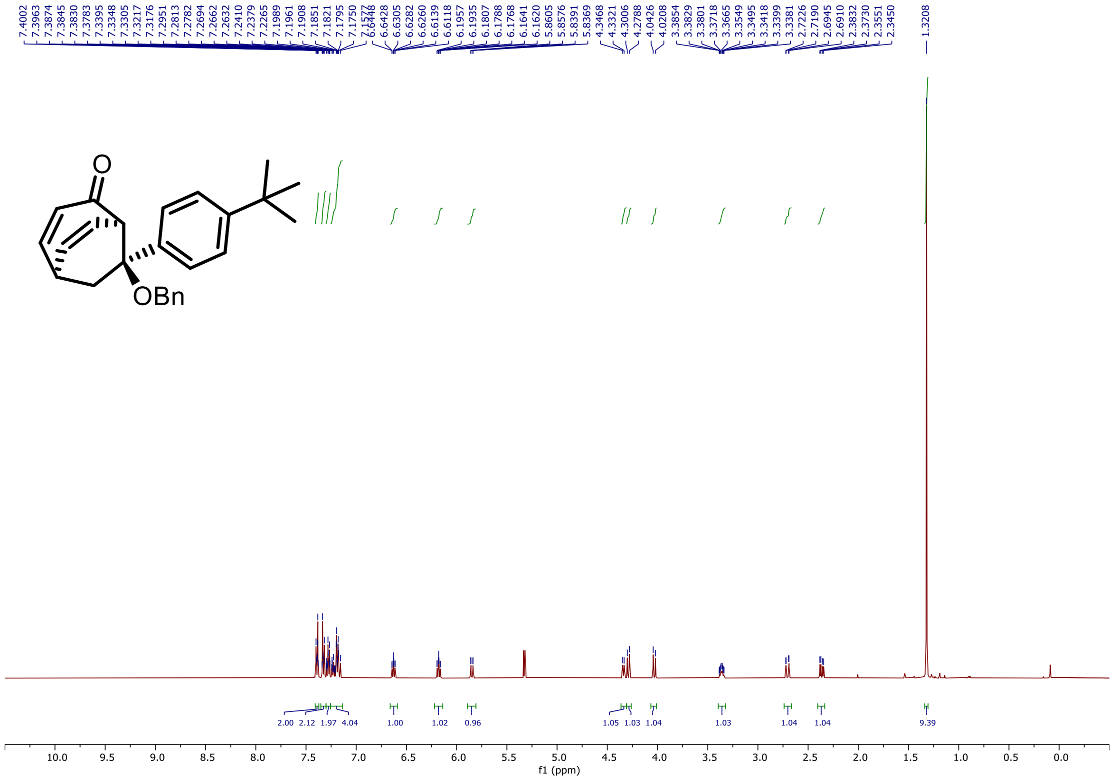
7.4002 7.3963 7.3874 7.3845 7.3830 7.3783 7.3395 7.3348 7.3305 7.3217 7.3176 7.2951 7.2813 7.2782 7.2694 7.2662 7.2632 7.2410 7.2379 7.2265 7.1989 7.1961 7.1908 7.1851 7.1821 7.1795 7.1750 7.1577 6.6448 6.6428 6.6305 6.6282 6.6260 6.6139 6.6118 6.1957 6.1935 6.1807 6.1788 6.1768 6.1641 6.1620 5.8605 5.8576 5.8391 5.8369 4.3468 4.3321 4.3006 4.2788 4.0426 4.0208 3.3854 3.3829 3.3801 3.3718 3.3665 3.3549 3.3495 3.3418 3.3399 3.3381 2.7226 2.7190 2.6945 2.6910 2.3832 2.3730 2.3551 2.3450 1.3208
O
OBn
2.00 2.12 1.97 4.04 1.00 1.02 0.96 1.05 1.03 1.04 1.03 1.04 1.04 9.39
10.0 9.5 9.0 8.5 8.0 7.5 7.0 6.5 6.0 5.5 5.0 4.5 4.0 3.5 3.0 2.5 2.0 1.5 1.0 0.5 0.0
f1 (ppm)

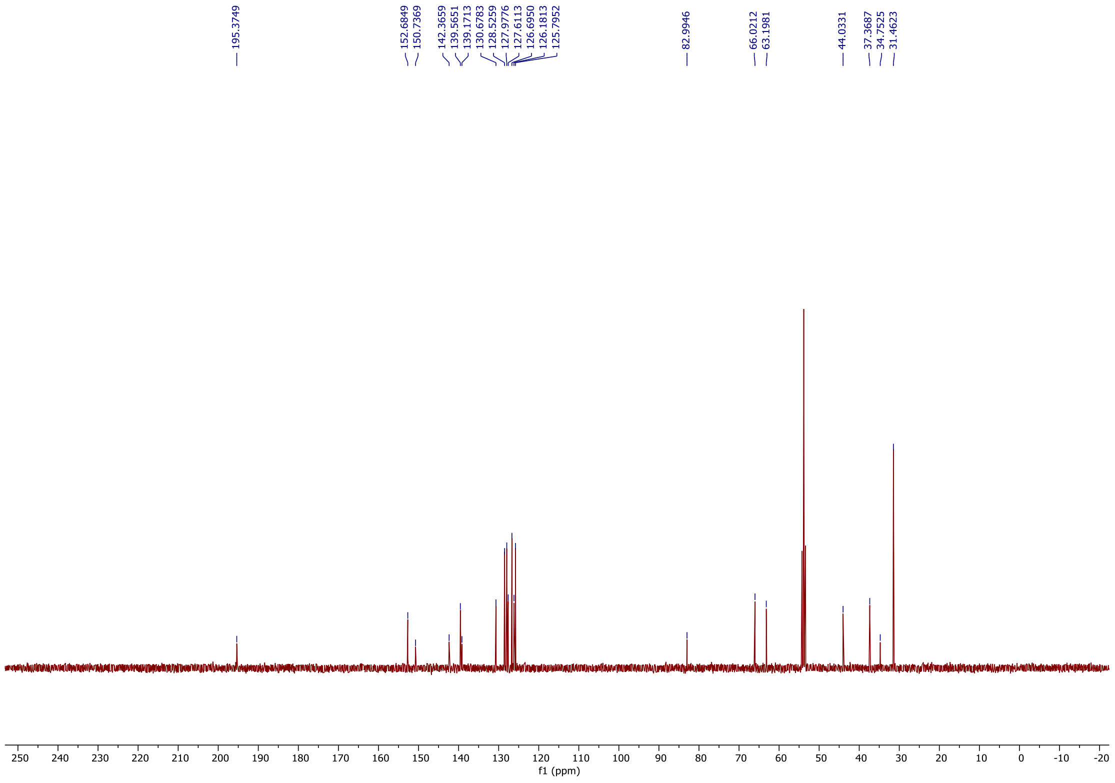
195.3749
152.6849 150.7369 142.3659 139.5651 139.1713 130.6783 128.5259 127.9776 127.6113 126.6950 126.1813 125.7952
82.9946
66.0212 63.1981
44.0331 37.3687 34.7525 31.4623
250 240 230 220 210 200 190 180 170 160 150 140 130 120 110 100 90 80 70 60 50 40 30 20 10 0 -10 -20
f1 (ppm)

3h-2

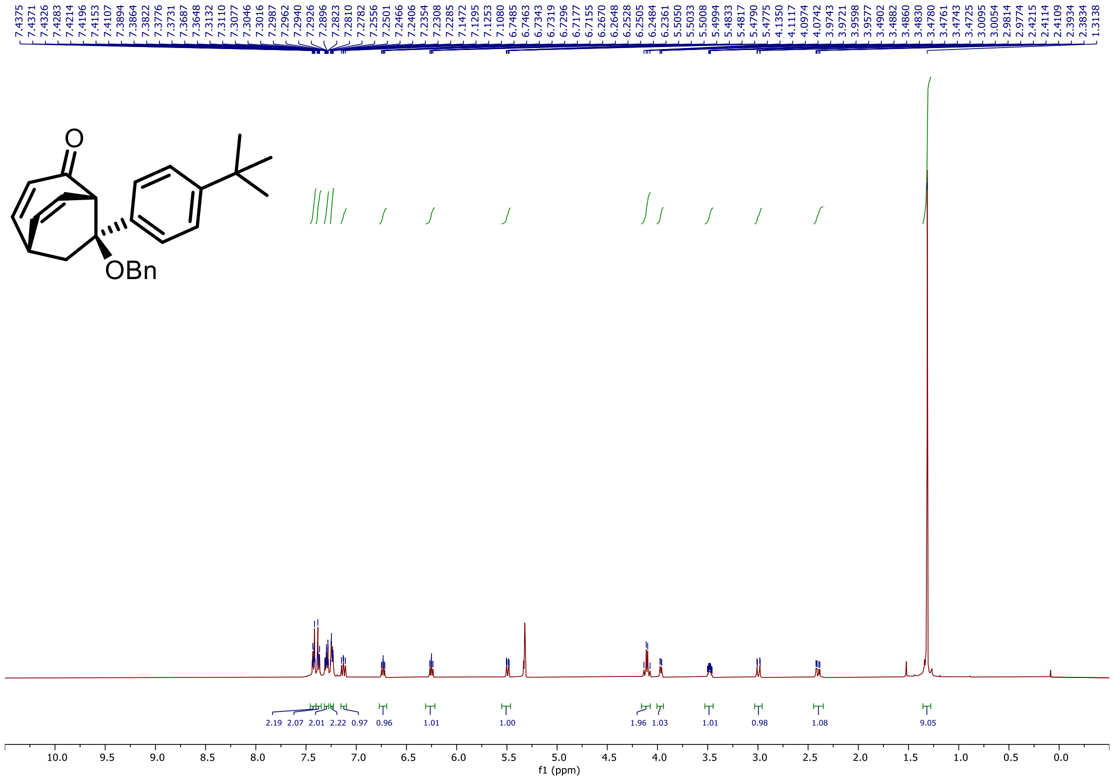
O
OBn
7.4375
7.4371
7.4326
7.4283
7.4214
7.4196
7.4153
7.4107
7.3894
7.3864
7.3822
7.3776
7.3731
7.3687
7.3648
7.3132
7.3110
7.3077
7.3046
7.3016
7.2987
7.2962
7.2940
7.2926
7.2896
7.2823
7.2810
7.2782
7.2556
7.2501
7.2466
7.2406
7.2354
7.2308
7.2285
7.1472
7.1295
7.1253
7.1080
6.7485
6.7463
6.7343
6.7319
6.7296
6.7177
6.7155
6.2670
6.2648
6.2528
6.2505
6.2484
6.2361
5.5050
5.5033
5.5008
5.4994
5.4833
5.4817
5.4790
5.4775
4.1350
4.1117
4.0974
4.0742
3.9743
3.9721
3.9598
3.9577
3.4902
3.4882
3.4860
3.4830
3.4780
3.4761
3.4743
3.4725
3.0095
3.0054
2.9814
2.9774
2.4215
2.4114
2.4109
2.3934
2.3834
1.3138
2.19
2.07
2.01
2.22
0.97
0.96
1.01
1.00
1.96
1.03
1.01
0.98
1.08
9.05
10.0
9.5
9.0
8.5
8.0
7.5
7.0
6.5
6.0
5.5
5.0
4.5
4.0
3.5
3.0
2.5
2.0
1.5
1.0
0.5
0.0
f1 (ppm)

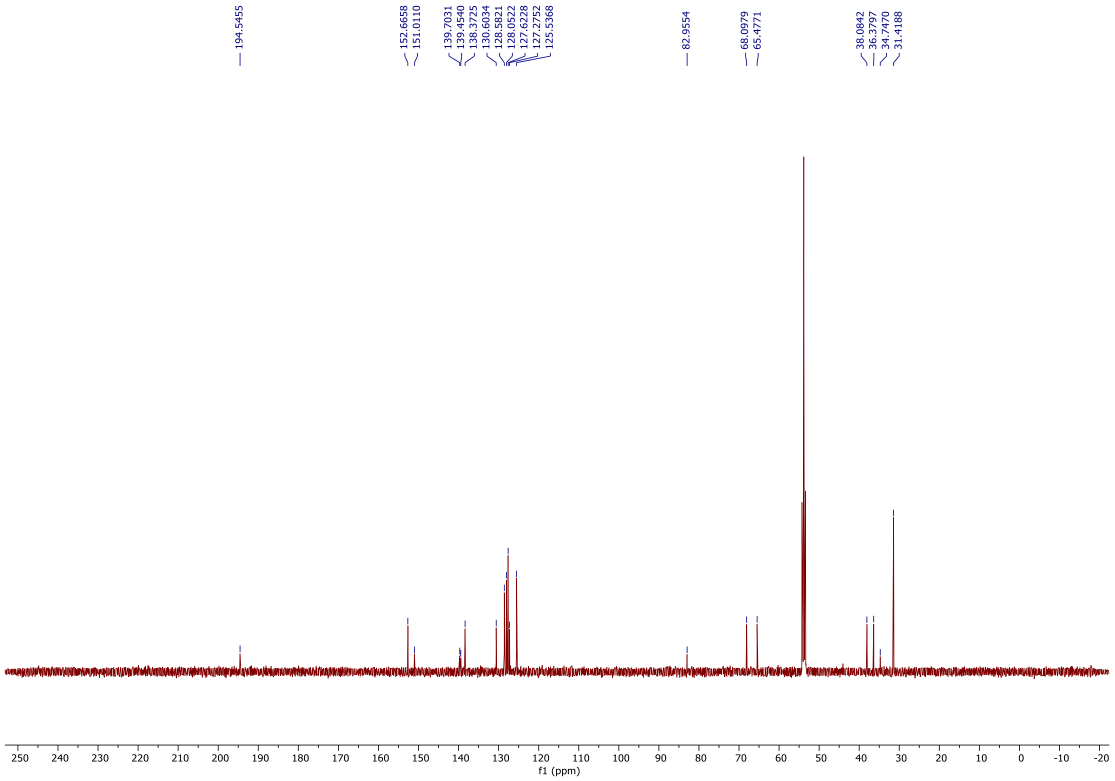
194.5455
152.6658
151.0110
139.7031
139.4540
138.3725
130.6034
128.5821
128.0522
127.6228
127.2752
125.5368
82.9554
68.0979
65.4771
38.0842
36.3797
34.7470
31.4188
250
240
230
220
210
200
190
180
170
160
150
140
130
120
110
100
90
80
70
60
50
40
30
20
10
0
-10
-20
f1 (ppm)

3i

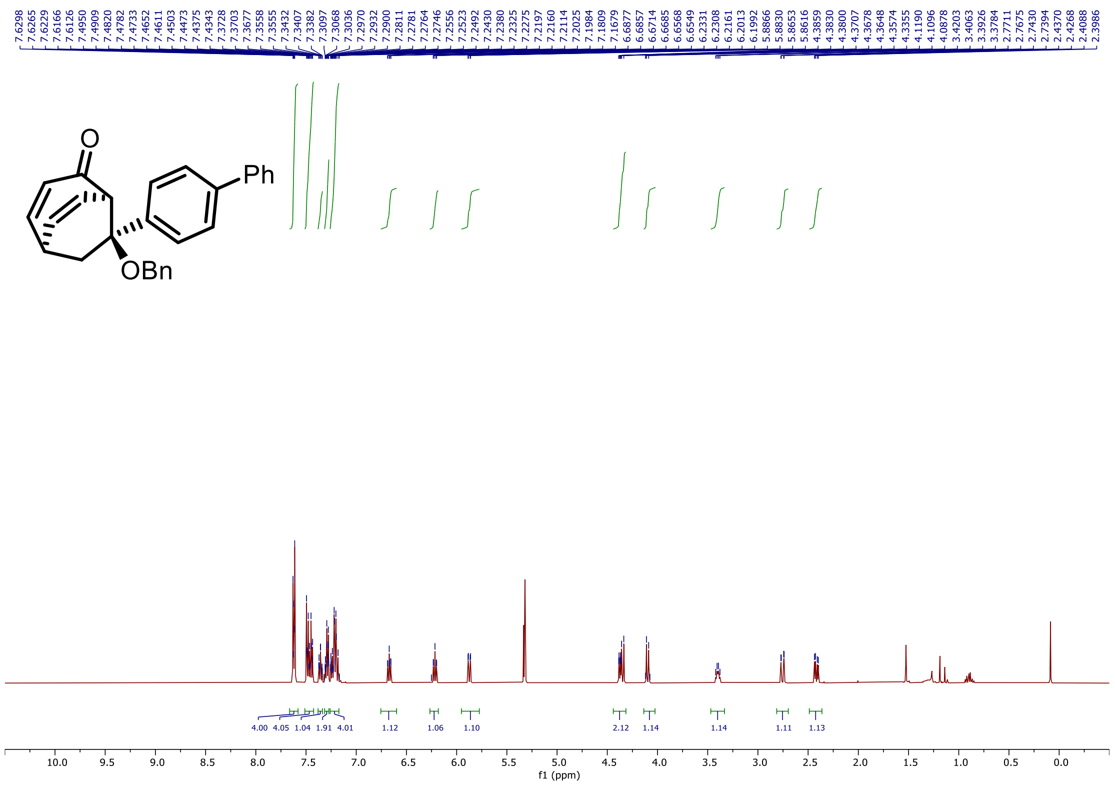

Ph
O
OBn
7.6298
7.6265
7.6229
7.6166
7.6126
7.4950
7.4909
7.4820
7.4782
7.4733
7.4652
7.4611
7.4503
7.4473
7.4375
7.4343
7.3728
7.3703
7.3677
7.3558
7.3555
7.3432
7.3407
7.3382
7.3097
7.3068
7.3036
7.2970
7.2932
7.2900
7.2811
7.2781
7.2764
7.2746
7.2556
7.2523
7.2492
7.2430
7.2380
7.2325
7.2275
7.2197
7.2160
7.2114
7.2025
7.1984
7.1809
7.1679
6.6877
6.6857
6.6714
6.6685
6.6568
6.6549
6.2331
6.2308
6.2161
6.2013
6.1992
5.8866
5.8830
5.8653
5.8616
4.3859
4.3830
4.3800
4.3707
4.3678
4.3648
4.3574
4.3355
4.1190
4.1096
4.0878
3.4203
3.4063
3.3926
3.3784
2.7711
2.7675
2.7430
2.7394
2.4370
2.4268
2.4088
2.3986
4.00
4.05
1.04
1.91
4.01
1.12
1.06
1.10
2.12
1.14
1.14
1.11
1.13
f1 (ppm)

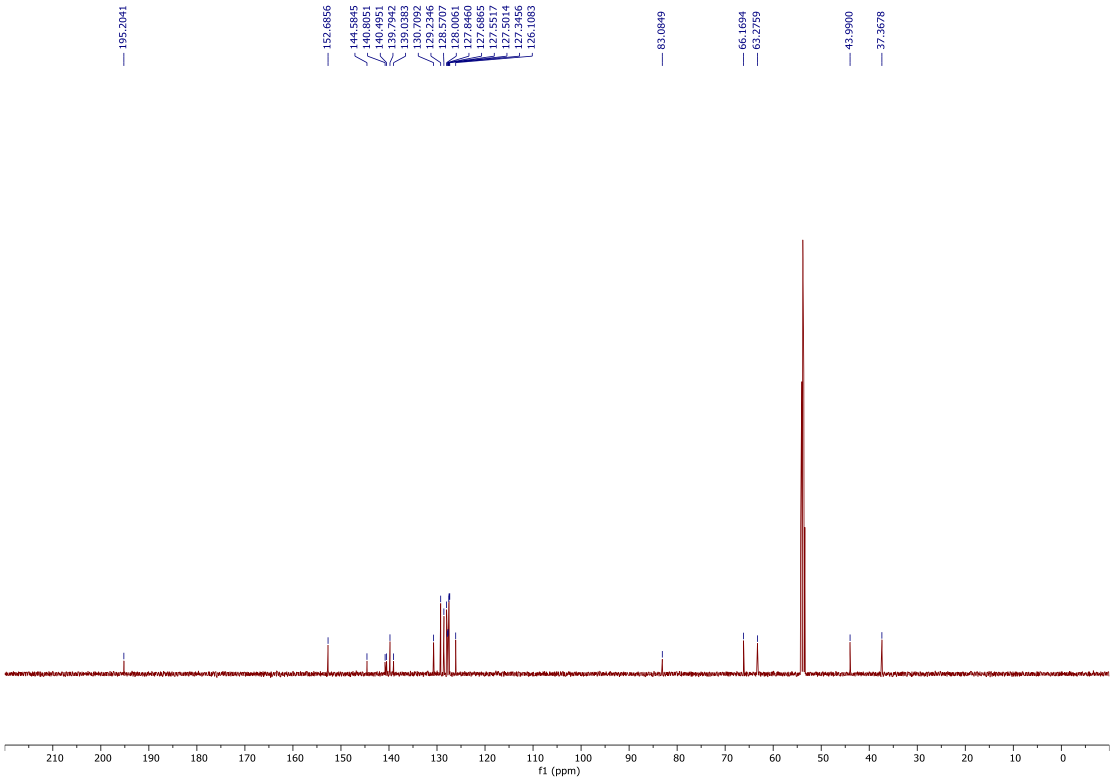

195.2041
152.6856
144.5845
140.8051
140.4951
139.7942
139.0383
130.7092
129.2346
128.5707
128.0061
127.8460
127.6865
127.5517
127.5014
127.3456
126.1083
83.0849
66.1694
63.2759
43.9900
37.3678
f1 (ppm)

3i-2

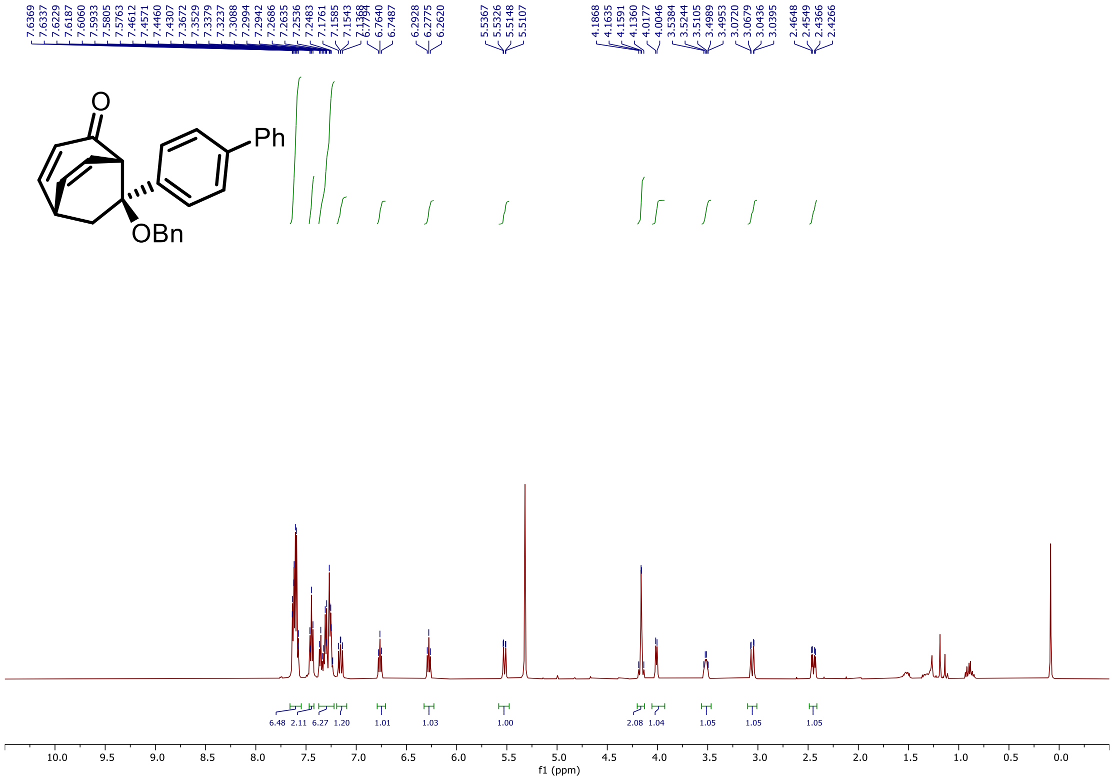

O
Ph
OBn
7.6369 7.6337 7.6229 7.6187 7.6060 7.5933 7.5805 7.5763 7.4612 7.4571 7.4460 7.4307 7.3672 7.3529 7.3379 7.3237 7.3088 7.2994 7.2942 7.2686 7.2635 7.2536 7.2483 7.1761 7.1585 7.1543
6.7640 6.7487
6.2928 6.2775 6.2620
5.5367 5.5326 5.5148 5.5107
4.1868 4.1635 4.1591 4.1360 4.0177 4.0046
3.5384 3.5244 3.5105 3.4989 3.4953
3.0720 3.0679 3.0436 3.0395
2.4648 2.4549 2.4366 2.4266
6.48 2.11 6.27 1.20 1.01 1.03 1.00 2.08 1.04 1.05 1.05 1.05
f1 (ppm)

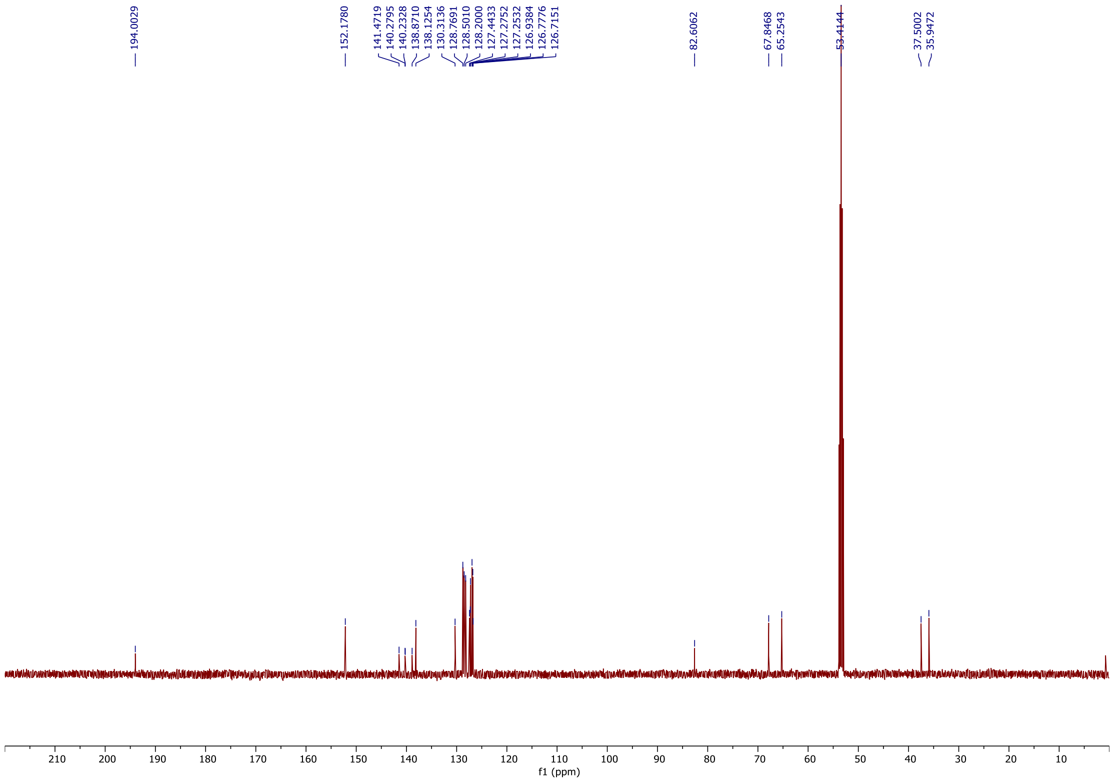

194.0029
152.1780
141.4719 140.2795 140.2328 138.8710 138.1254 130.3136 128.7691 128.5010 128.2000 127.4433 127.2752 127.2532 126.9384 126.7776 126.7151
82.6062
67.8468 65.2543
53.4144
37.5002 35.9472
f1 (ppm)

3j

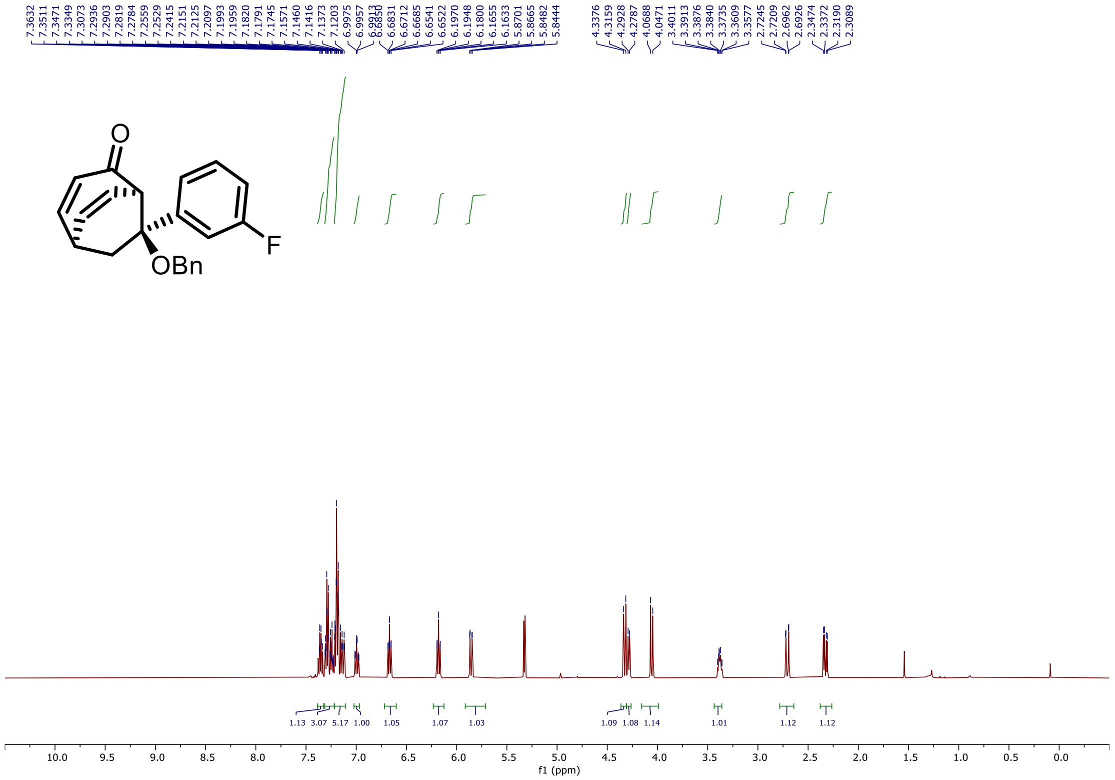
O
F
OBn
7.3632
7.3511
7.3471
7.3349
7.3073
7.2936
7.2903
7.2819
7.2784
7.2559
7.2529
7.2415
7.2151
7.2125
7.2097
7.1993
7.1959
7.1820
7.1791
7.1745
7.1571
7.1460
7.1416
7.1373
7.1203
6.9975
6.9957
6.9913
6.6850
6.6831
6.6712
6.6685
6.6541
6.6522
6.1970
6.1948
6.1800
6.1655
6.1633
5.8701
5.8665
5.8482
5.8444
4.3376
4.3159
4.2928
4.2787
4.0688
4.0471
3.4011
3.3913
3.3876
3.3840
3.3735
3.3609
3.3577
2.7245
2.7209
2.6962
2.6926
2.3474
2.3372
2.3190
2.3089
1.13 3.07 5.17 1.00 1.05 1.07 1.03 1.09 1.08 1.14 1.01 1.12 1.12
10.0 9.5 9.0 8.5 8.0 7.5 7.0 6.5 6.0 5.5 5.0 4.5 4.0 3.5 3.0 2.5 2.0 1.5 1.0 0.5 0.0
f1 (ppm)

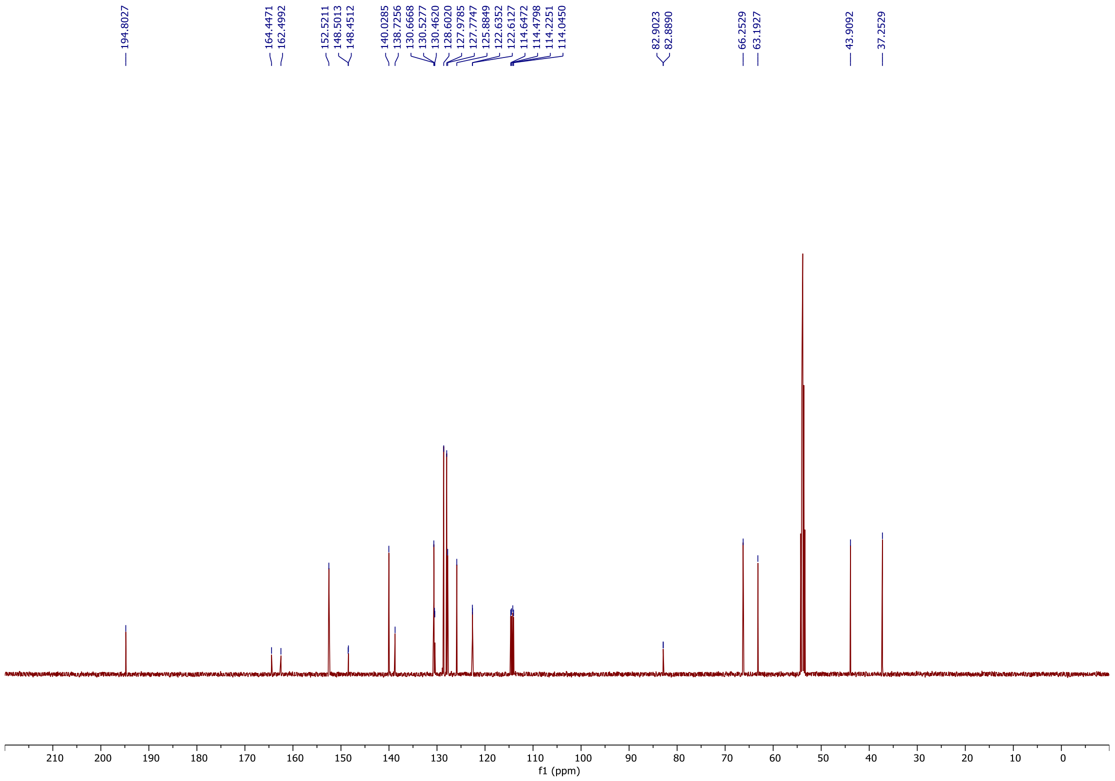
194.8027
164.4471
162.4992
152.5211
148.5013
148.4512
140.0285
138.7256
130.6668
130.5277
130.4620
128.6020
127.9785
127.7747
125.8849
122.6352
122.6127
114.6472
114.4798
114.2251
114.0450
82.9023
82.8890
66.2529
63.1927
43.9092
37.2529
210 200 190 180 170 160 150 140 130 120 110 100 90 80 70 60 50 40 30 20 10 0
f1 (ppm)

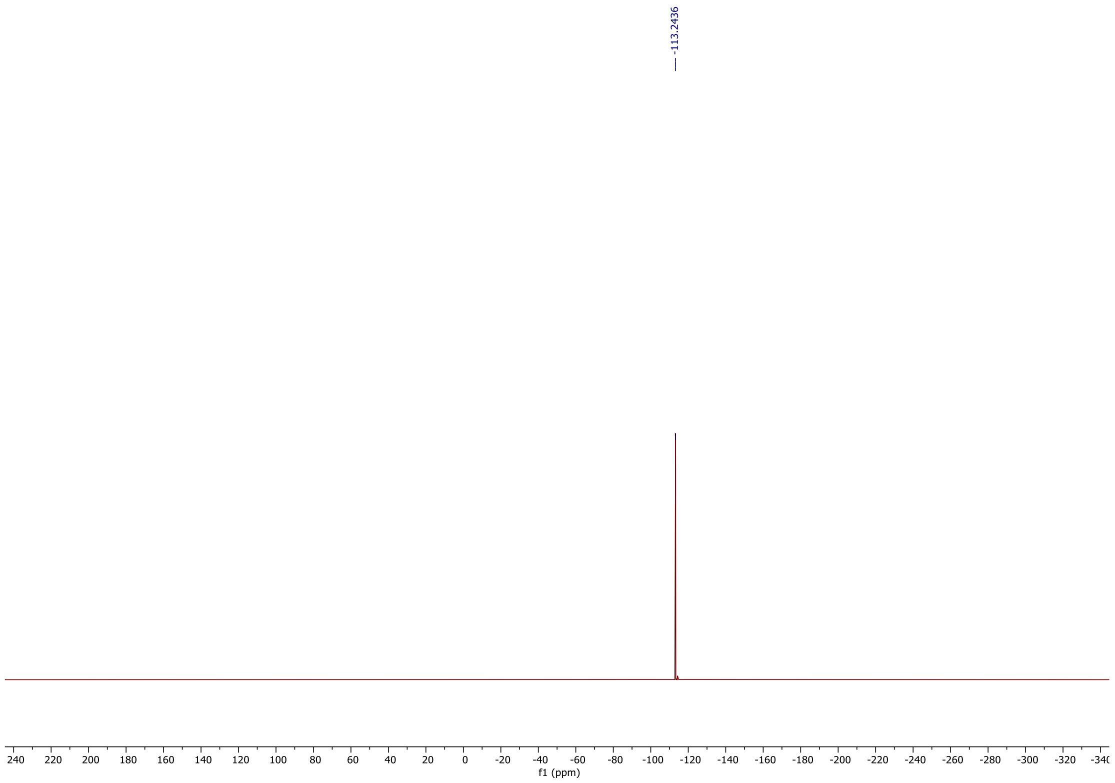
-113.2436
240
220
200
180
160
140
120
100
80
60
40
20
0
-20
-40
-60
-80
-100
-120
-140
-160
-180
-200
-220
-240
-260
-280
-300
-320
-340
f1 (ppm)

3j-2

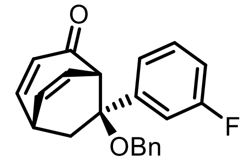
O
F
OBn

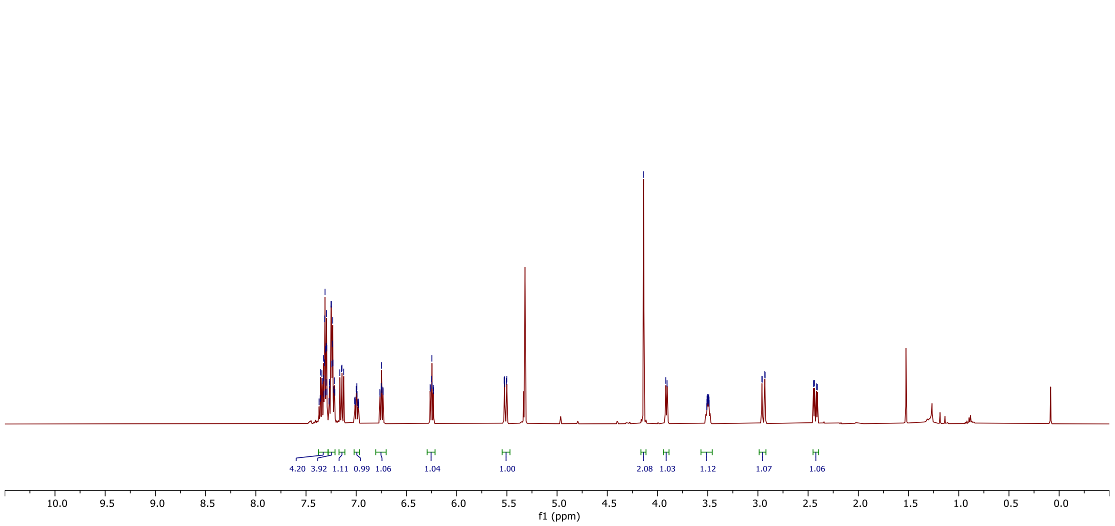
7.3699
7.3580
7.3541
7.3423
7.3381
7.3267
7.3239
7.3210
7.3170
7.3141
7.3107
7.3077
7.3034
7.3013
7.2984
7.2956
7.2937
7.2911
7.2704
7.2672
7.2642
7.2509
7.2471
7.2435
7.2385
7.2345
7.2317
7.2222
7.2179
7.2136
7.1640
7.1465
7.1423
7.1248
7.0161
7.0137
7.0108
7.0086
6.9996
6.9972
6.9944
6.9919
6.9836
6.9809
6.9783
6.9758
6.7649
6.7626
6.7507
6.7482
6.7457
6.7340
6.7318
6.2637
6.2614
6.2493
6.2471
6.2447
6.2327
6.2305
5.5264
5.5229
5.5213
5.5048
5.5008
4.1380
3.9176
3.9047
3.5074
3.5054
3.5033
3.5005
3.4975
3.4955
3.4930
3.4900
3.4875
3.4856
2.9604
2.9562
2.9320
2.9280
2.4465
2.4367
2.4184
2.4083
4.20
3.92
1.11
0.99
1.06
1.04
1.00
2.08
1.03
1.12
1.07
1.06
10.0 9.5 9.0 8.5 8.0 7.5 7.0 6.5 6.0 5.5 5.0 4.5 4.0 3.5 3.0 2.5 2.0 1.5 1.0 0.5 0.0
f1 (ppm)

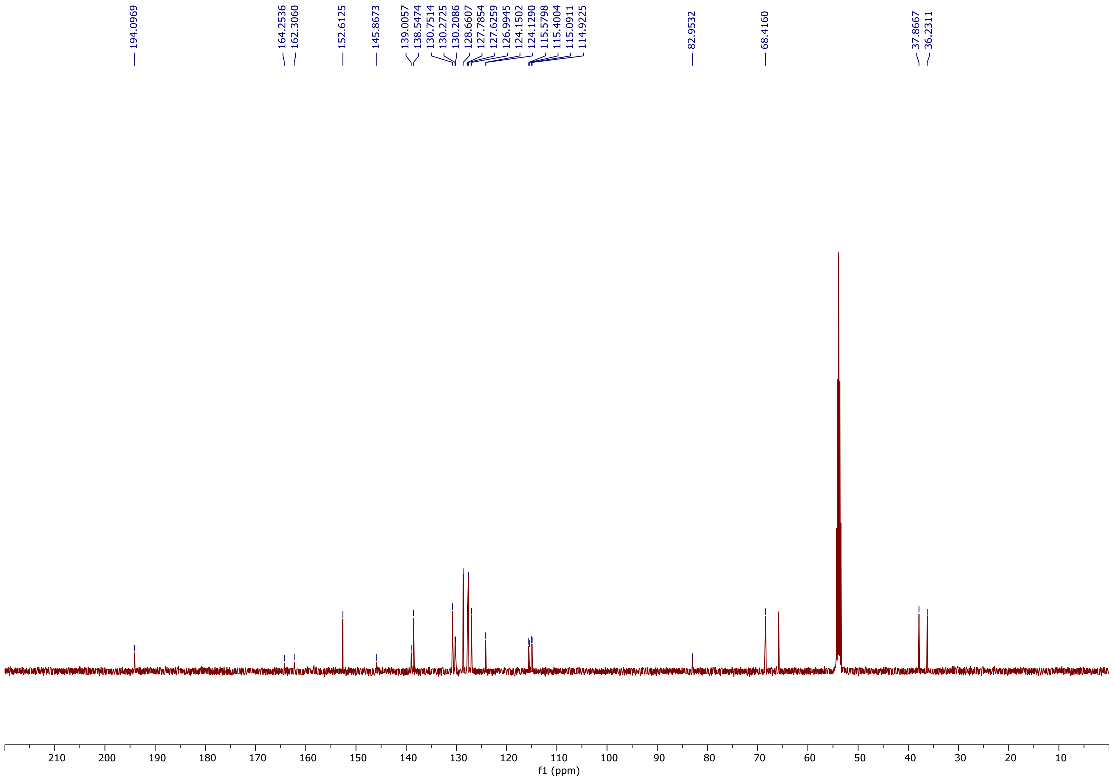
194.0969
164.2536
162.3060
152.6125
145.8673
139.0057
138.5474
130.7514
130.2725
130.2086
128.6607
127.7854
127.6259
126.9945
124.1502
124.1290
115.5798
115.4004
115.0911
114.9225
82.9532
68.4160
37.8667
36.2311
210 200 190 180 170 160 150 140 130 120 110 100 90 80 70 60 50 40 30 20 10
f1 (ppm)

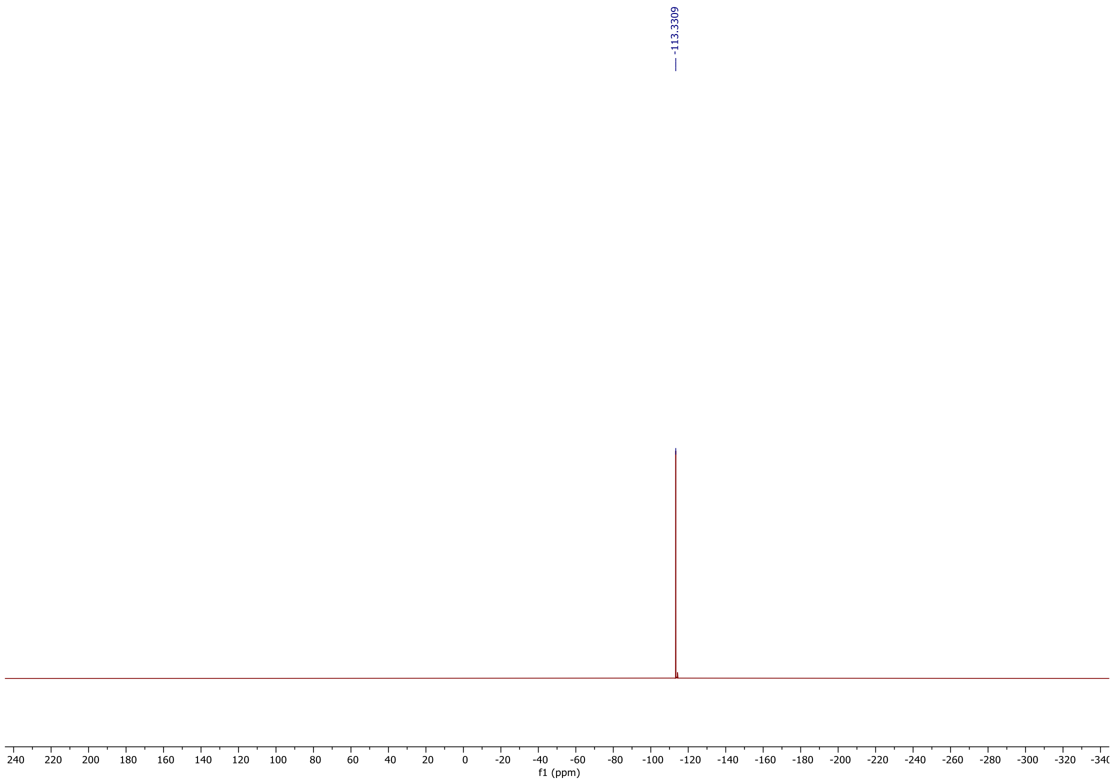
-113.3309
240 220 200 180 160 140 120 100 80 60 40 20 0 -20 -40 -60 -80 -100 -120 -140 -160 -180 -200 -220 -240 -260 -280 -300 -320 -340
f1 (ppm)

3k

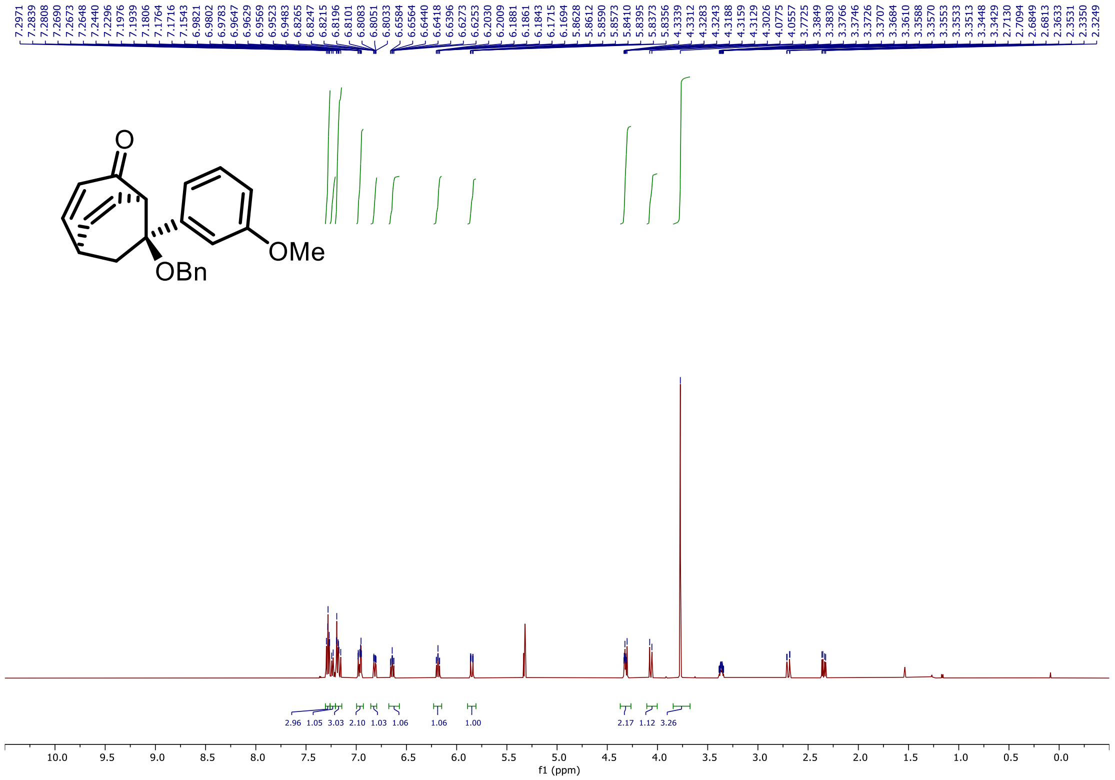
O
OBn
OMe
7.2971
7.2839
7.2808
7.2690
7.2673
7.2648
7.2440
7.2296
7.1976
7.1939
7.1806
7.1764
7.1716
7.1543
6.9821
6.9802
6.9783
6.9647
6.9629
6.9569
6.9523
6.9483
6.8265
6.8247
6.8215
6.8196
6.8101
6.8083
6.8051
6.8033
6.6584
6.6564
6.6440
6.6418
6.6396
6.6273
6.6253
6.2030
6.2009
6.1881
6.1861
6.1843
6.1715
6.1694
5.8628
5.8612
5.8590
5.8573
5.8410
5.8395
5.8373
5.8356
4.3339
4.3312
4.3283
4.3243
4.3188
4.3159
4.3129
4.3026
4.0775
4.0557
3.7725
3.3849
3.3830
3.3766
3.3746
3.3726
3.3707
3.3684
3.3610
3.3588
3.3570
3.3553
3.3533
3.3513
3.3448
3.3429
2.7130
2.7094
2.6849
2.6813
2.3633
2.3531
2.3350
2.3249
2.96 1.05 3.03 2.10 1.03 1.06 1.06 1.00 2.17 1.12 3.26
10.0 9.5 9.0 8.5 8.0 7.5 7.0 6.5 6.0 5.5 5.0 4.5 4.0 3.5 3.0 2.5 2.0 1.5 1.0 0.5 0.0
f1 (ppm)

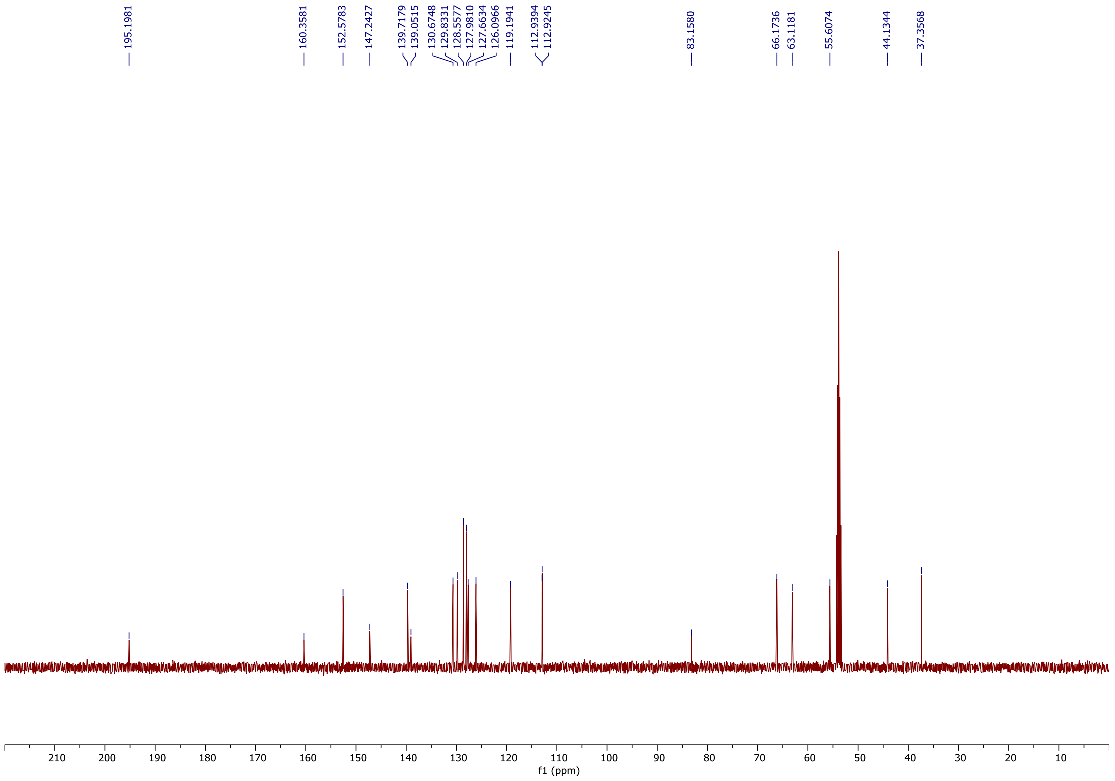
195.1981
160.3581
152.5783
147.2427
139.7179
139.0515
130.6748
129.8331
128.5577
127.9810
127.6634
126.0966
119.1941
112.9394
112.9245
83.1580
66.1736
63.1181
55.6074
44.1344
37.3568
210 200 190 180 170 160 150 140 130 120 110 100 90 80 70 60 50 40 30 20 10
f1 (ppm)

3k-2

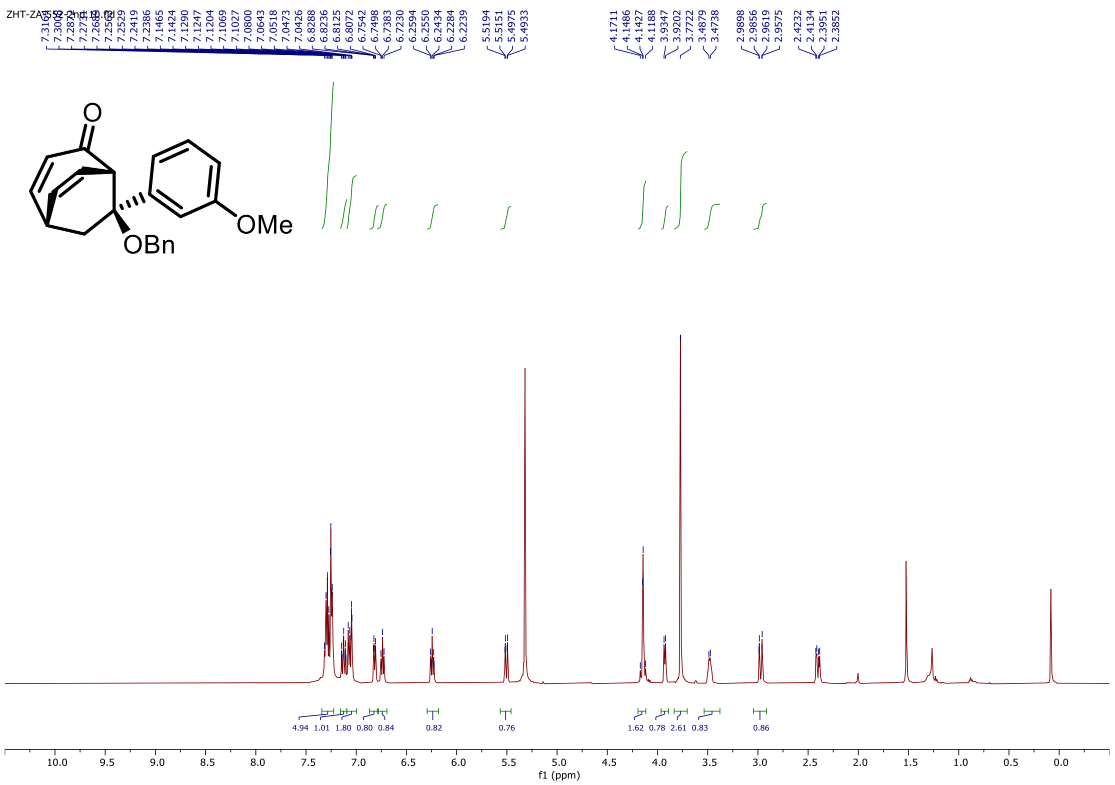

7.2529
7.2419
7.2386
7.1465
7.1424
7.1290
7.1247
7.1204
7.1069
7.1027
7.0800
7.0643
7.0518
7.0473
7.0426
6.8288
6.8236
6.8125
6.8072
6.7998
6.7542
6.7383
6.7230
6.2594
6.2550
6.2434
6.2284
6.2239
5.5194
5.5151
5.4975
5.4933
4.1711
4.1486
4.1427
4.1188
3.9347
3.9202
3.7722
3.4879
3.4738
2.9898
2.9856
2.9619
2.9575
2.4232
2.4134
2.3951
2.3852
O
OMe
OBn
4.94
1.01
1.80
0.80
0.84
0.82
0.76
1.62
0.78
2.61
0.83
0.86
f1 (ppm)

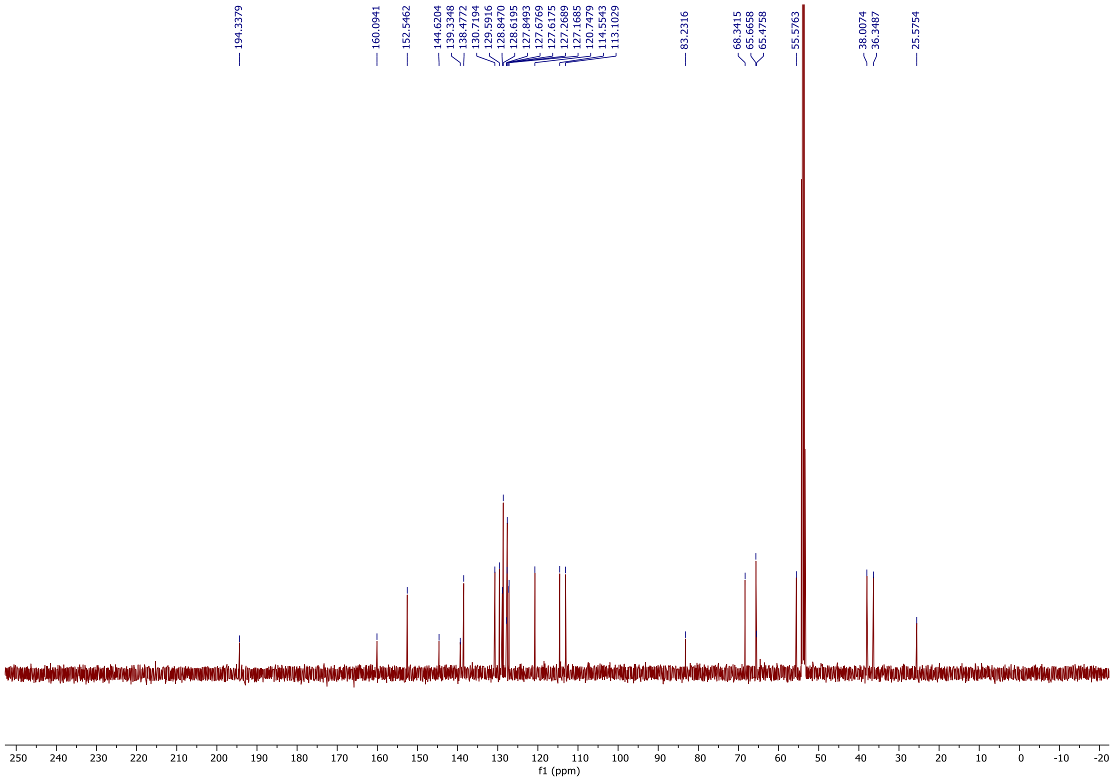

194.3379
160.0941
152.5462
144.6204
139.3348
138.4772
130.7194
129.5916
128.8470
128.6195
127.8493
127.6769
127.6175
127.2689
121.1685
120.7479
114.5543
113.1029
83.2316
68.3415
65.6658
65.4758
55.5763
38.0074
36.3487
25.5754
f1 (ppm)

3l

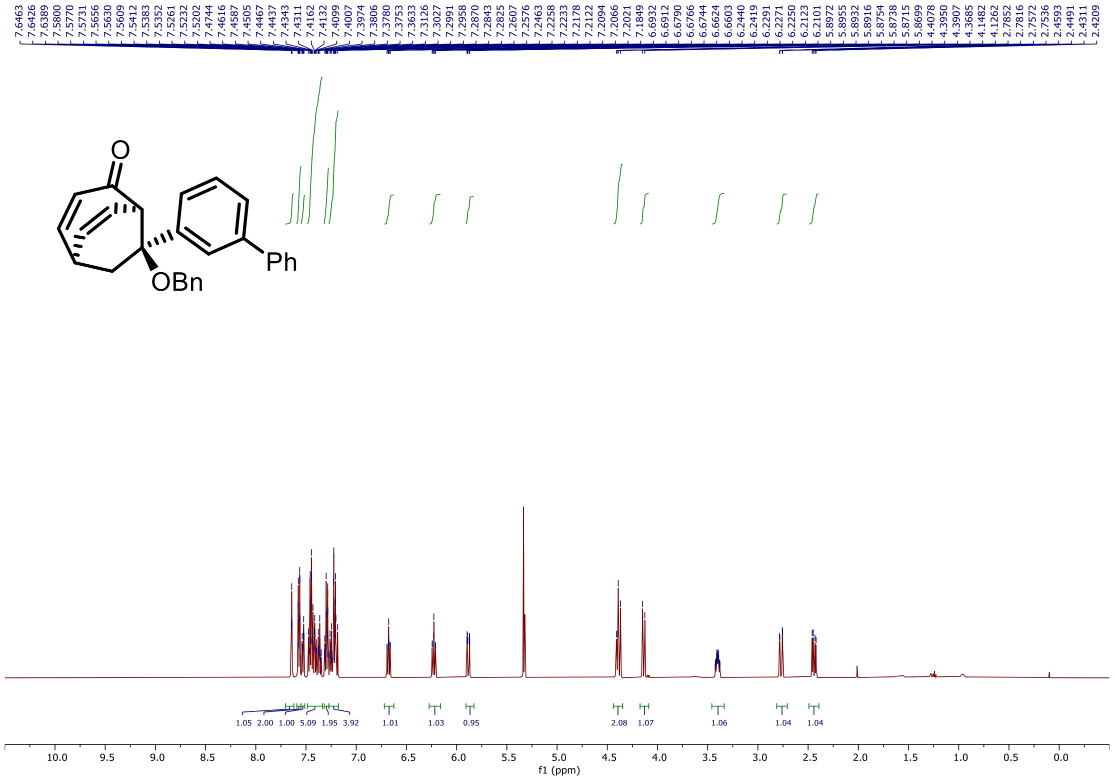

7.6463
7.6426
7.6389
7.5800
7.5770
7.5731
7.5656
7.5630
7.5609
7.5412
7.5383
7.5352
7.5261
7.5232
7.5202
7.4744
7.4616
7.4587
7.4505
7.4467
7.4437
7.4343
7.4311
7.4162
7.4132
7.4099
7.4007
7.3974
7.3806
7.3780
7.3753
7.3633
7.3126
7.3027
7.2991
7.2958
7.2870
7.2843
7.2825
7.2607
7.2576
7.2463
7.2258
7.2233
7.2178
7.2122
7.2094
7.2066
7.2021
7.1849
6.6932
6.6912
6.6790
6.6766
6.6744
6.6624
6.6603
6.2440
6.2419
6.2291
6.2271
6.2250
6.2123
6.2101
5.8972
5.8955
5.8932
5.8916
5.8754
5.8738
5.8715
5.8699
4.4078
4.3950
4.3907
4.3685
4.1482
4.1262
2.7852
2.7816
2.7572
2.7536
2.4593
2.4491
2.4311
2.4209
O
Ph
OBn
1.05 2.00 1.00 5.09 1.95 3.92
1.01
1.03
0.95
2.08
1.07
1.06
1.04
1.04
10.0 9.5 9.0 8.5 8.0 7.5 7.0 6.5 6.0 5.5 5.0 4.5 4.0 3.5 3.0 2.5 2.0 1.5 1.0 0.5 0.0
f1 (ppm)

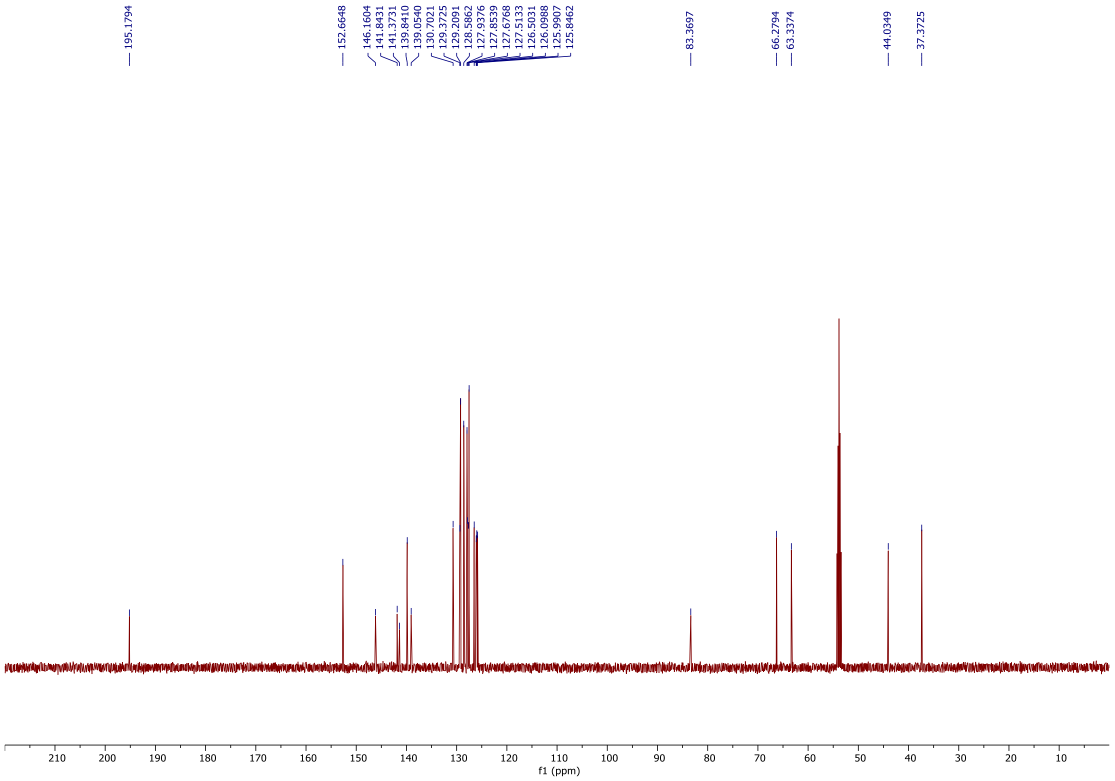

195.1794
152.6648
146.1604
141.8431
141.3731
139.8410
139.0540
130.7021
129.3725
129.2091
128.5862
127.9376
127.8539
127.6768
127.5133
126.5031
126.0988
125.9907
125.8462
83.3697
66.2794
63.3374
44.0349
37.3725
210 200 190 180 170 160 150 140 130 120 110 100 90 80 70 60 50 40 30 20 10
f1 (ppm)

3l-2

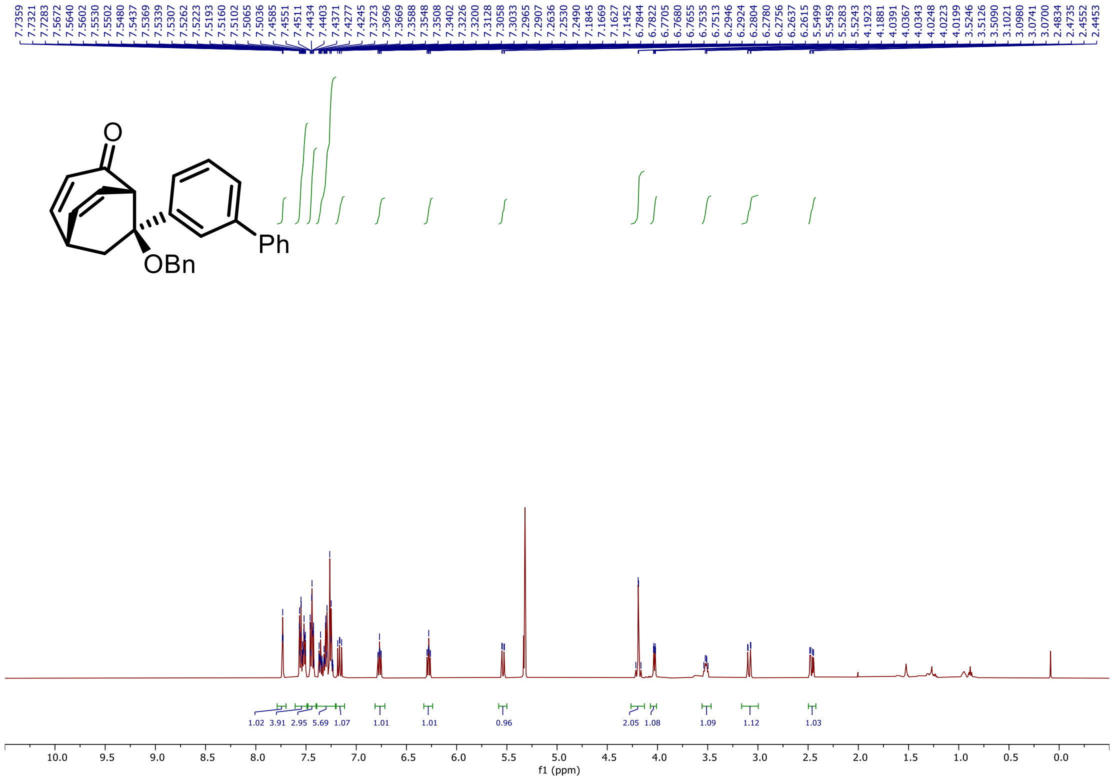

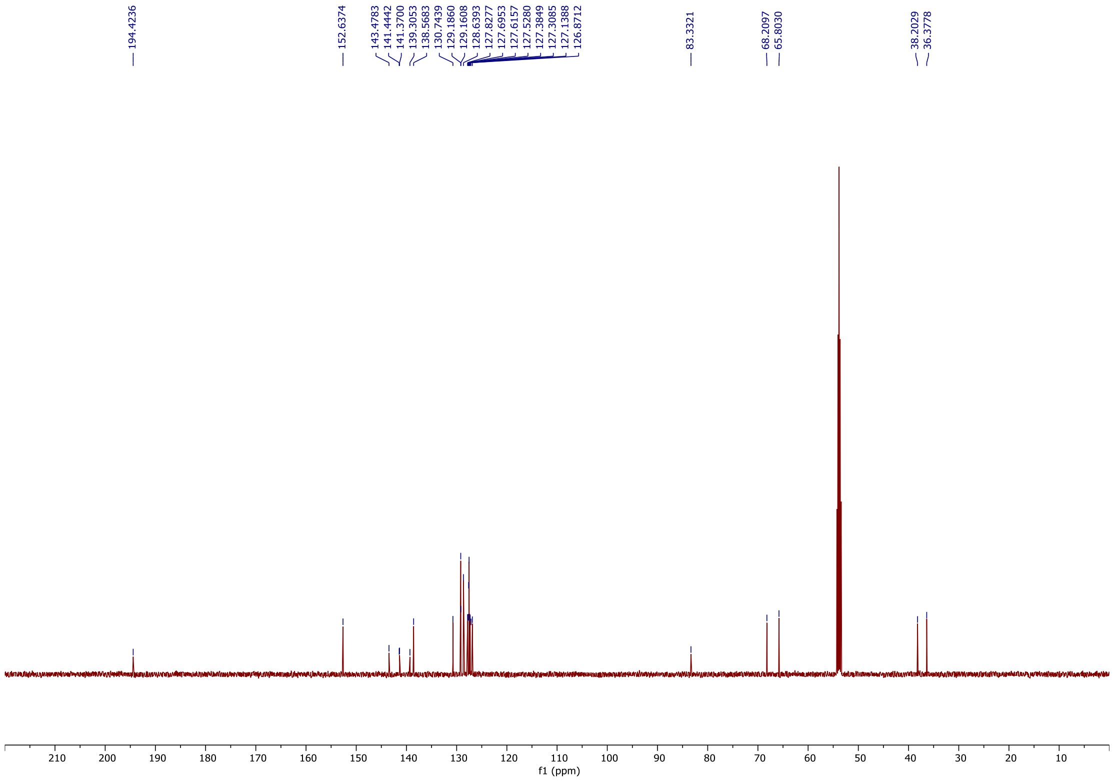

3m

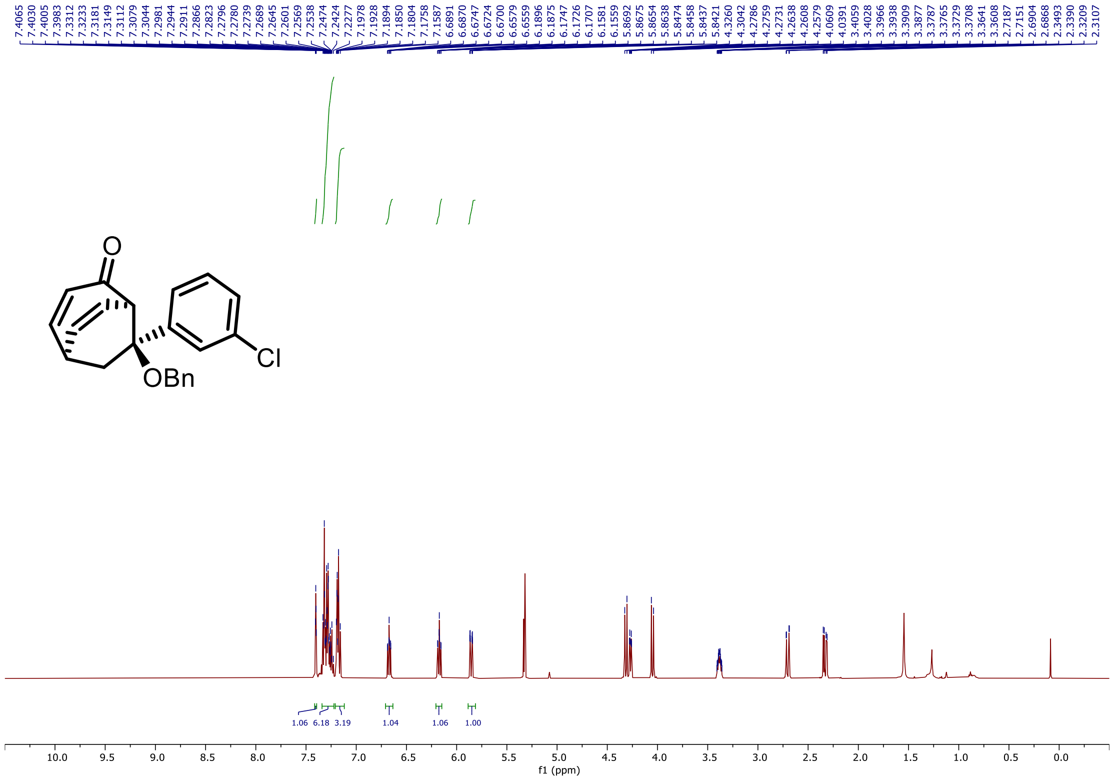

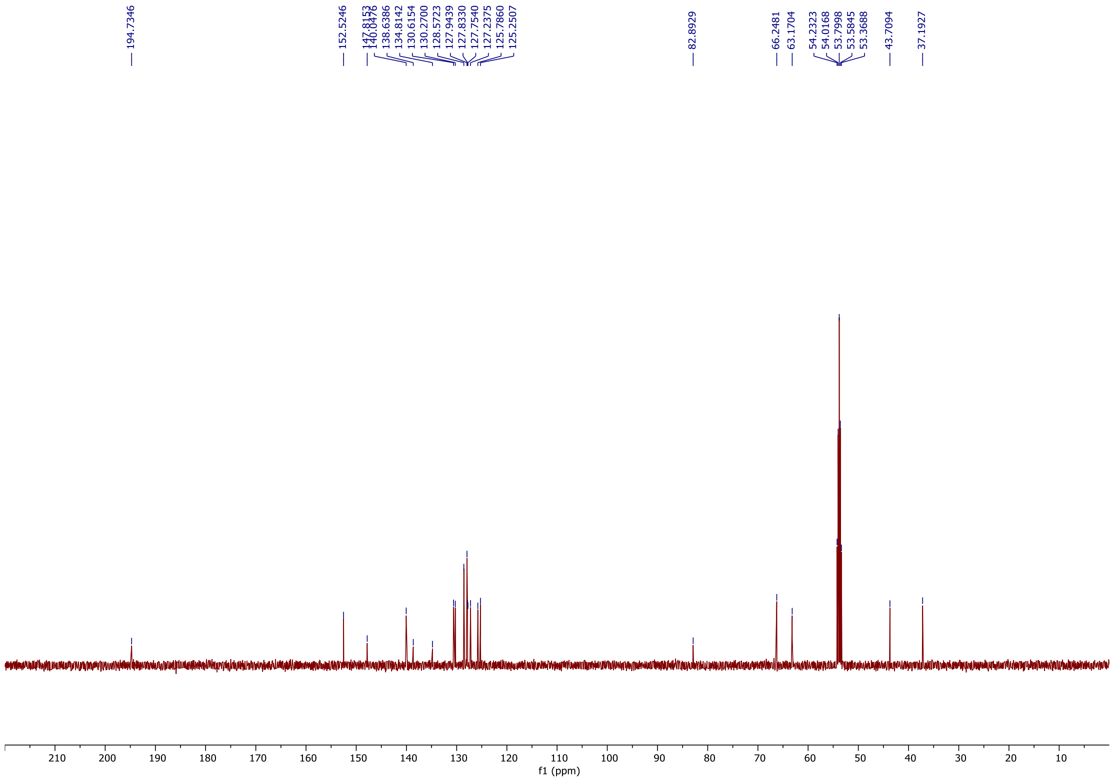

3m-2

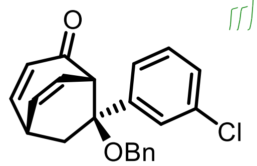
O
Cl
OBn

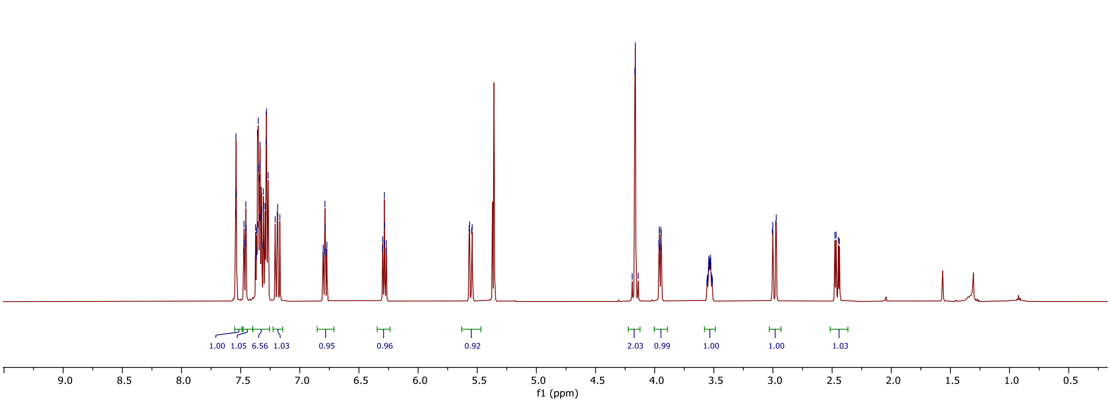
7.5436
7.5398
7.5358
7.4747
7.4716
7.4684
7.4595
7.4562
7.4530
7.3736
7.3675
7.3645
7.3617
7.3578
7.3547
7.3509
7.3479
7.3424
7.3390
7.3362
7.3343
7.3279
7.3244
7.3214
7.3118
7.3081
7.3053
7.2933
7.2853
7.2818
7.2681
7.2079
7.1904
7.1863
7.1685
6.8041
6.8018
6.7900
6.7876
6.7852
6.7732
6.7710
6.3015
6.2992
6.2873
6.2850
6.2826
6.2706
6.2683
5.5686
5.5646
5.5469
5.5428
4.1903
4.1674
4.1630
4.1398
3.9638
3.9614
3.9592
3.9575
3.9494
3.9471
3.9449
3.9433
3.5557
3.5488
3.5464
3.5444
3.5422
3.5385
3.5346
3.5318
3.5288
3.5266
3.5247
3.5217
3.5139
3.0050
3.0009
2.9766
2.9725
2.4762
2.4663
2.4478
2.4380
1.00
1.05
6.56
1.03
0.95
0.96
0.92
2.03
0.99
1.00
1.00
1.03
9.0
8.5
8.0
7.5
7.0
6.5
6.0
5.5
5.0
4.5
4.0
3.5
3.0
2.5
2.0
1.5
1.0
0.5
f1 (ppm)

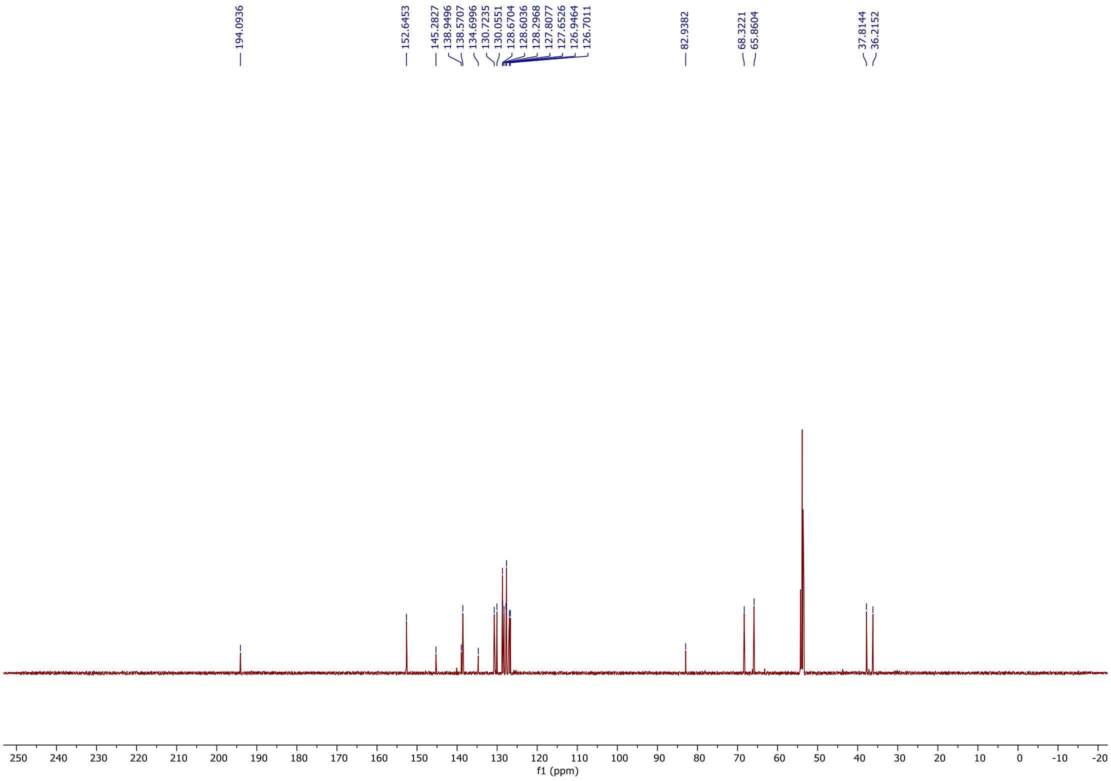
194.0936
152.6453
145.2827
138.9496
138.5707
134.6996
130.7235
130.0551
128.6704
128.6036
128.2968
127.8077
127.6526
126.9464
126.7011
82.9382
68.3221
65.8604
37.8144
36.2152
250
240
230
220
210
200
190
180
170
160
150
140
130
120
110
100
90
80
70
60
50
40
30
20
10
0
-10
-20
f1 (ppm)

3n

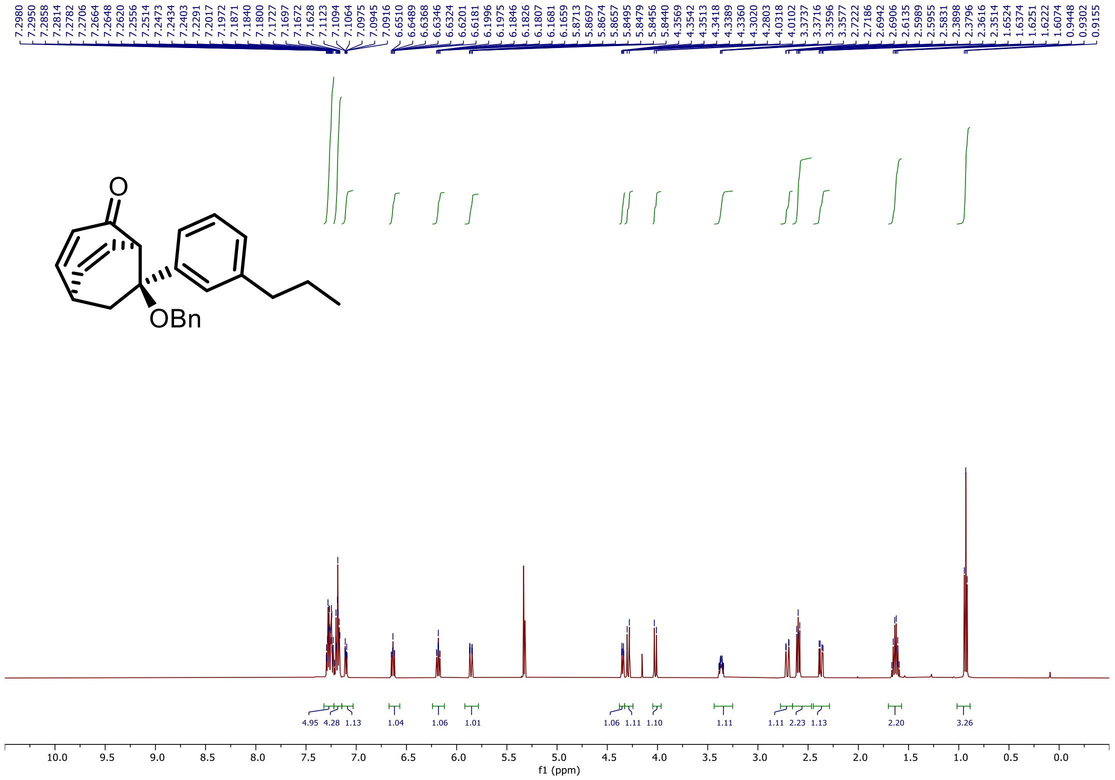

7.2980 7.2950 7.2858 7.2814 7.2782 7.2706 7.2664 7.2648 7.2620 7.2556 7.2514 7.2473 7.2434 7.2403 7.2291 7.2017 7.1972 7.1871 7.1840 7.1800 7.1727 7.1697 7.1672 7.1628 7.1123 7.1094 7.1064 7.0975 7.0945 7.0916 6.6510 6.6489 6.6368 6.6346 6.6324 6.6201 6.6181 6.1996 6.1975 6.1846 6.1826 6.1807 6.1681 6.1659 5.8713 5.8697 5.8674 5.8657 5.8495 5.8479 5.8456 5.8440 4.3569 4.3542 4.3513 4.3418 4.3389 4.3360 4.3020 4.2803 4.0318 4.0102 3.3737 3.3716 3.3596 3.3577 2.7222 2.7186 2.6942 2.6906 2.6135 2.5989 2.5955 2.5831 2.3898 2.3796 2.3616 2.3514 1.6524 1.6374 1.6251 1.6222 1.6074 0.9448 0.9302 0.9155
O
OBn
4.95 4.28 1.13 1.04 1.06 1.01 1.06 1.11 1.10 1.11 1.11 2.23 1.13 2.20 3.26
10.0 9.5 9.0 8.5 8.0 7.5 7.0 6.5 6.0 5.5 5.0 4.5 4.0 3.5 3.0 2.5 2.0 1.5 1.0 0.5 0.0
f1 (ppm)

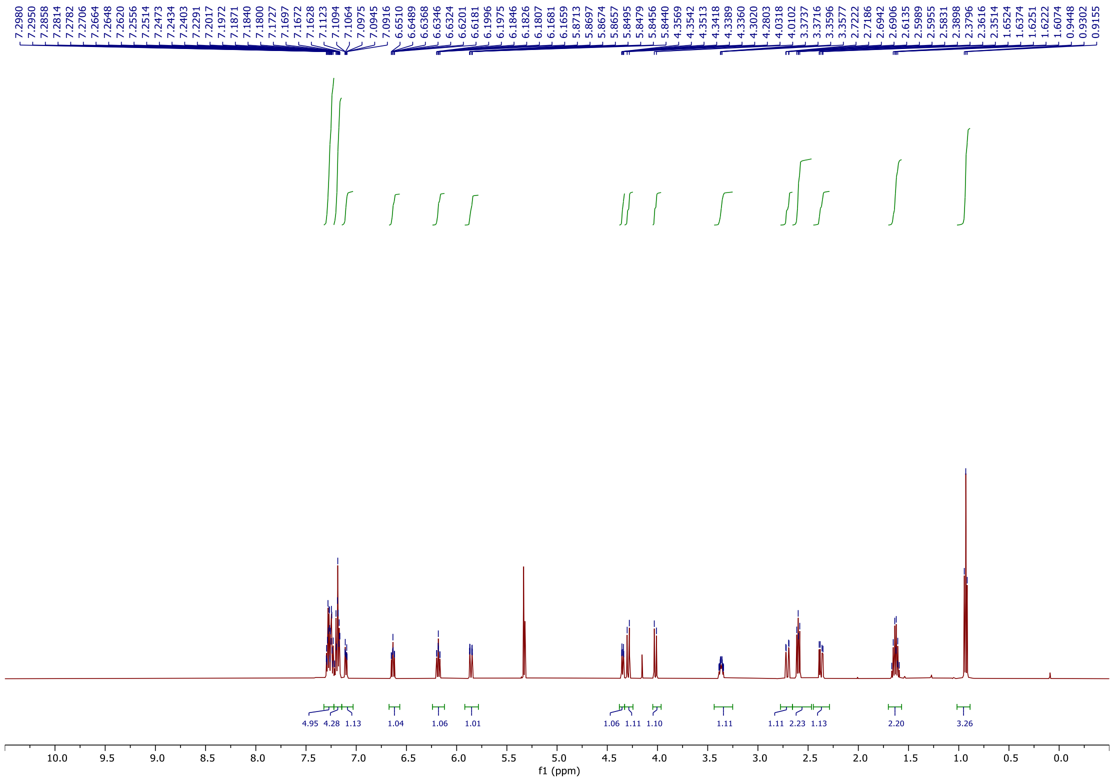

7.2980 7.2950 7.2858 7.2814 7.2782 7.2706 7.2664 7.2648 7.2620 7.2556 7.2514 7.2473 7.2434 7.2403 7.2291 7.2017 7.1972 7.1871 7.1840 7.1800 7.1727 7.1697 7.1672 7.1628 7.1123 7.1094 7.1064 7.0975 7.0945 7.0916 6.6510 6.6489 6.6368 6.6346 6.6324 6.6201 6.6181 6.1996 6.1975 6.1846 6.1826 6.1807 6.1681 6.1659 5.8713 5.8697 5.8674 5.8657 5.8495 5.8479 5.8456 5.8440 4.3569 4.3542 4.3513 4.3418 4.3389 4.3360 4.3020 4.2803 4.0318 4.0102 3.3737 3.3716 3.3596 3.3577 2.7222 2.7186 2.6942 2.6906 2.6135 2.5989 2.5955 2.5831 2.3898 2.3796 2.3616 2.3514 1.6524 1.6374 1.6251 1.6222 1.6074 0.9448 0.9302 0.9155
4.95 4.28 1.13 1.04 1.06 1.01 1.06 1.11 1.10 1.11 1.11 2.23 1.13 2.20 3.26
10.0 9.5 9.0 8.5 8.0 7.5 7.0 6.5 6.0 5.5 5.0 4.5 4.0 3.5 3.0 2.5 2.0 1.5 1.0 0.5 0.0
f1 (ppm)

3n-2

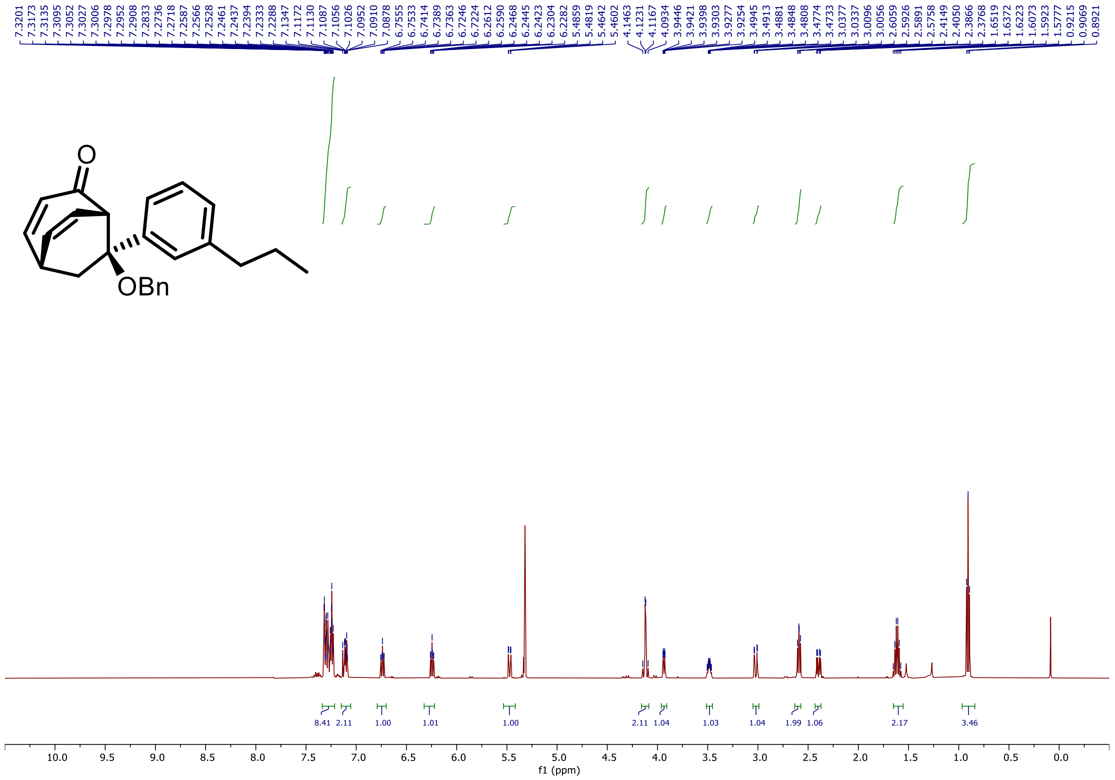
O
OBn
7.3201 7.3173 7.3135 7.3095 7.3052 7.3027 7.3006 7.2978 7.2952 7.2908 7.2833 7.2736 7.2718 7.2587 7.2566 7.2528 7.2461 7.2437 7.2394 7.2333 7.2288 7.1347 7.1172 7.1130 7.1087 7.1056 7.1026 7.0952 7.0910 7.0878 6.7555 6.7533 6.7414 6.7389 6.7363 6.7246 6.7224 6.2612 6.2590 6.2468 6.2445 6.2423 6.2304 6.2282 5.4859 5.4819 5.4642 5.4602 4.1463 4.1231 4.1167 4.0934 3.9446 3.9421 3.9398 3.9303 3.9277 3.9254 3.4945 3.4913 3.4881 3.4848 3.4808 3.4774 3.4733 3.0377 3.0337 3.0096 3.0056 2.6059 2.5926 2.5891 2.5758 2.4149 2.4050 2.3866 2.3768 1.6519 1.6372 1.6223 1.6073 1.5923 1.5777 0.9215 0.9069 0.8921
8.41 2.11 1.00 1.01 1.00 2.11 1.04 1.03 1.04 1.99 1.06 2.17 3.46
10.0 9.5 9.0 8.5 8.0 7.5 7.0 6.5 6.0 5.5 5.0 4.5 4.0 3.5 3.0 2.5 2.0 1.5 1.0 0.5 0.0
f1 (ppm)

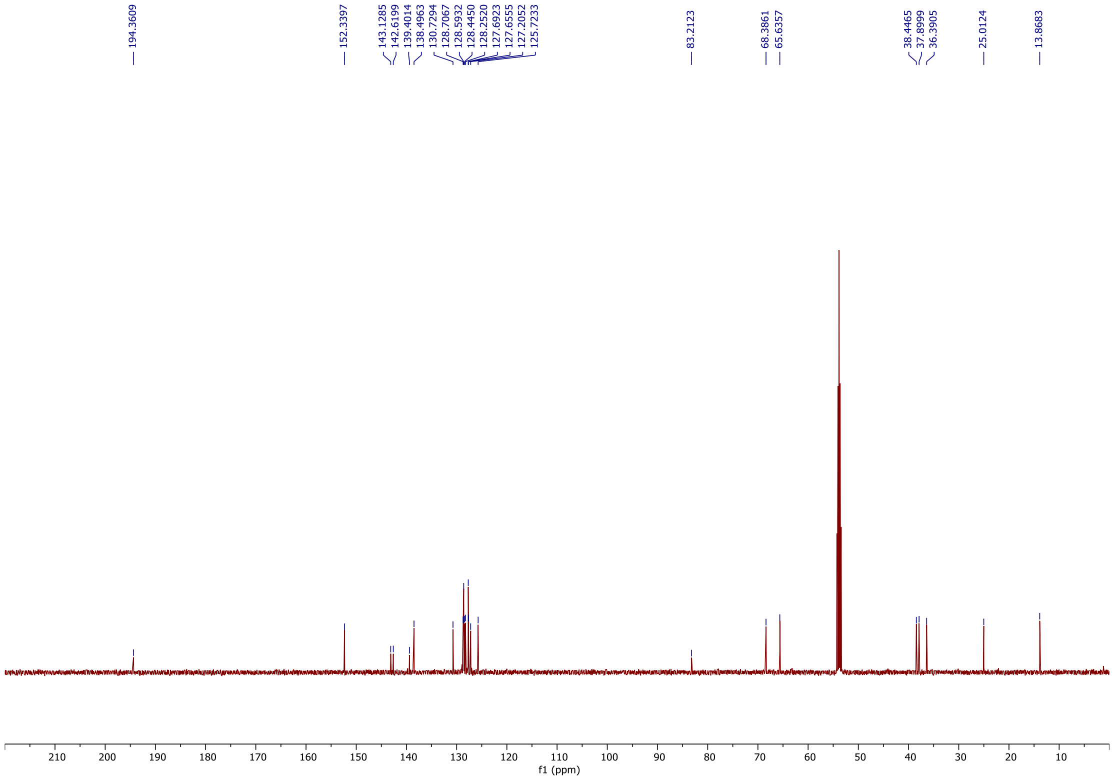
194.3609
152.3397
143.1285 142.6199 139.4014 138.4963 130.7294 128.7067 128.5932 128.4450 128.2520 127.6923 127.6555 127.2052 125.7233
83.2123
68.3861 65.6357
38.4465 37.8999 36.3905
25.0124
13.8683
210 200 190 180 170 160 150 140 130 120 110 100 90 80 70 60 50 40 30 20 10
f1 (ppm)

3o

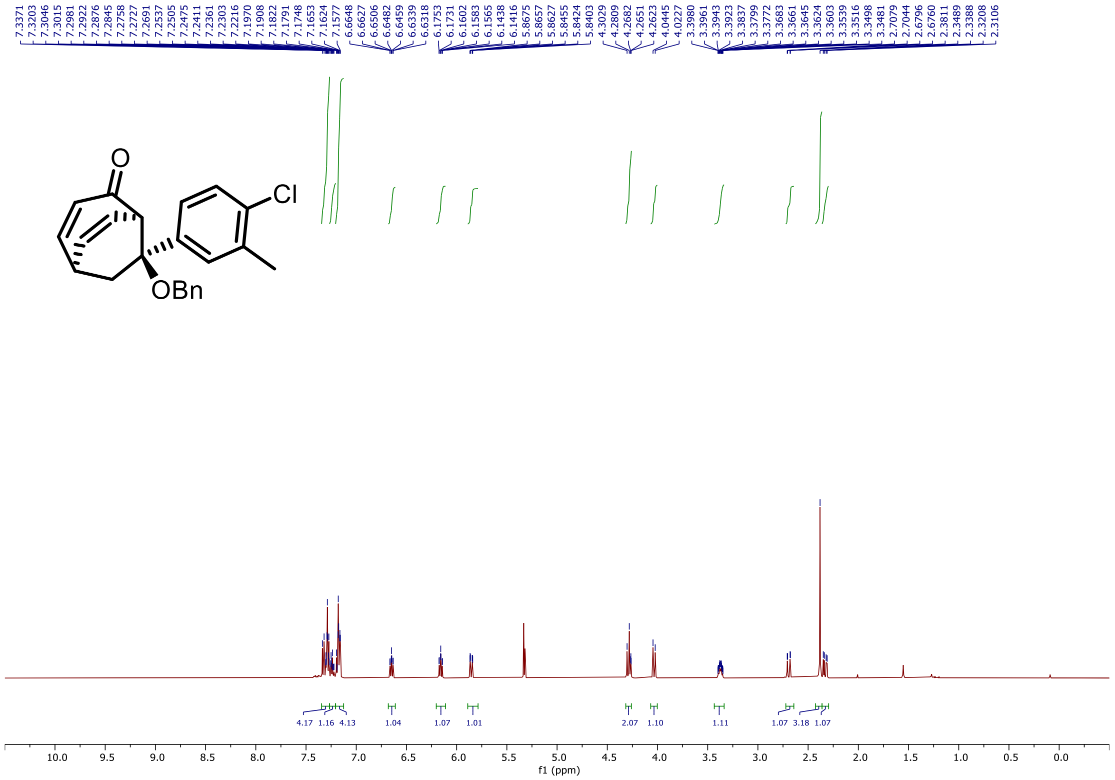
O
Cl
OBn
7.3371 7.3203 7.3046 7.3015 7.2981 7.2922 7.2876 7.2845 7.2758 7.2727 7.2691 7.2537 7.2505 7.2475 7.2411 7.2361 7.2303 7.2216 7.1970 7.1908 7.1822 7.1791 7.1748 7.1653 7.1624 7.1577 6.6648 6.6627 6.6506 6.6482 6.6459 6.6339 6.6318 6.1753 6.1731 6.1602 6.1583 6.1565 6.1438 6.1416 5.8675 5.8657 5.8627 5.8455 5.8424 5.8403 4.3029 4.2809 4.2682 4.2651 4.2623 4.0445 4.0227 3.3980 3.3961 3.3943 3.3923 3.3837 3.3799 3.3772 3.3683 3.3661 3.3645 3.3624 3.3603 3.3539 3.3516 3.3498 3.3481 2.7079 2.7044 2.6796 2.6760 2.3811 2.3489 2.3388 2.3208 2.3106
4.17 1.16 4.13
1.04
1.07
1.01
2.07
1.10
1.11
1.07 3.18 1.07
10.0 9.5 9.0 8.5 8.0 7.5 7.0 6.5 6.0 5.5 5.0 4.5 4.0 3.5 3.0 2.5 2.0 1.5 1.0 0.5 0.0
f1 (ppm)

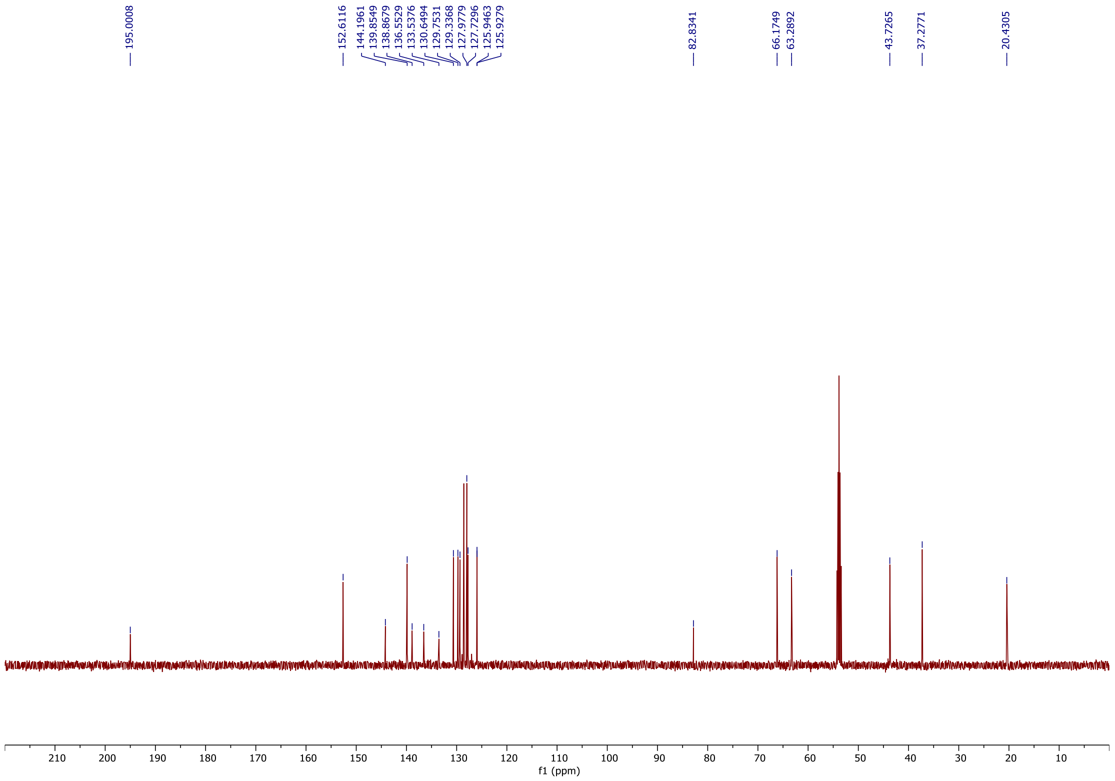
195.0008
152.6116 144.1961 139.8549 138.8679 136.5529 133.5376 130.6494 129.7531 129.3368 127.9779 127.7296 125.9463 125.9279
82.8341
66.1749 63.2892
43.7265
37.2771
20.4305
210 200 190 180 170 160 150 140 130 120 110 100 90 80 70 60 50 40 30 20 10
f1 (ppm)

3o-2

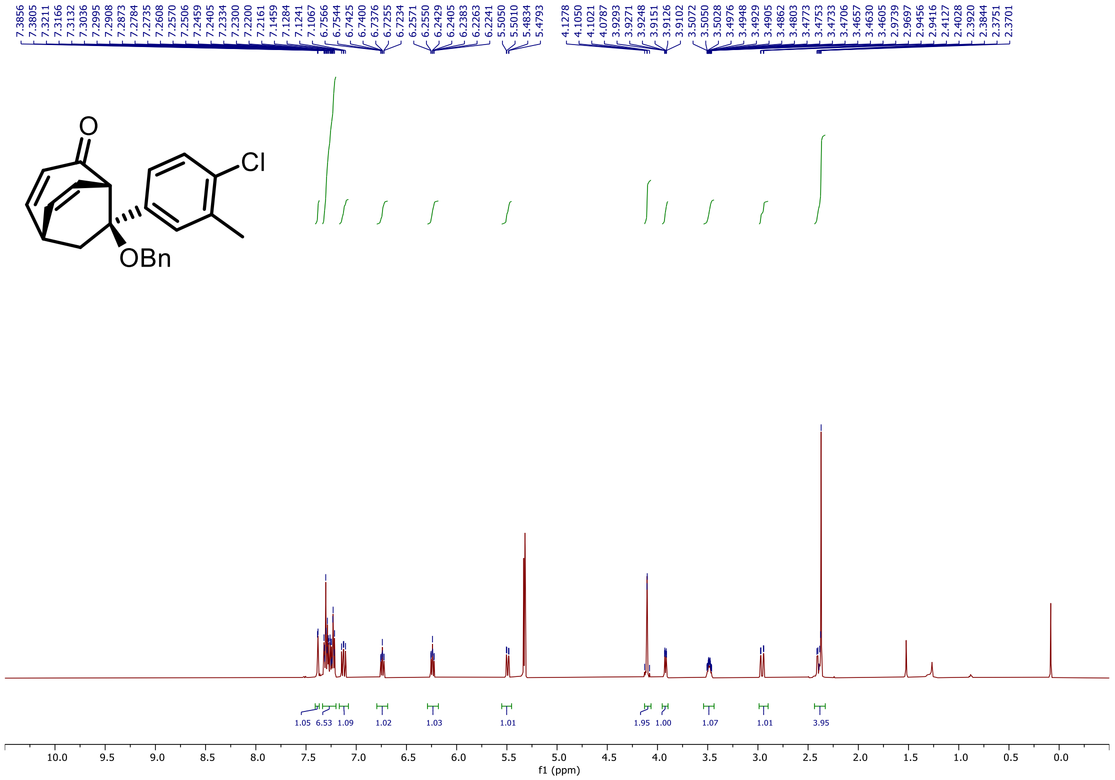
O
Cl
OBn
7.3856 7.3805 7.3211 7.3166 7.3132 7.3036 7.2995 7.2908 7.2873 7.2784 7.2735 7.2608 7.2570 7.2506 7.2459 7.2405 7.2334 7.2300 7.2200 7.2161 7.1459 7.1284 7.1241 7.1067 6.7566 6.7544 6.7425 6.7400 6.7376 6.7255 6.7234 6.2571 6.2550 6.2429 6.2405 6.2383 6.2263 6.2241 5.5050 5.5010 5.4834 5.4793 4.1278 4.1050 4.1021 4.0787 3.9293 3.9271 3.9248 3.9151 3.9126 3.9102 3.5072 3.5050 3.5028 3.4976 3.4948 3.4929 3.4905 3.4862 3.4803 3.4773 3.4753 3.4733 3.4706 3.4657 3.4630 3.4605 2.9739 2.9697 2.9456 2.9416 2.4127 2.4028 2.3920 2.3844 2.3751 2.3701
1.05 6.53 1.09 1.02 1.03 1.01 1.95 1.00 1.07 1.01 3.95
10.0 9.5 9.0 8.5 8.0 7.5 7.0 6.5 6.0 5.5 5.0 4.5 4.0 3.5 3.0 2.5 2.0 1.5 1.0 0.5 0.0
f1 (ppm)

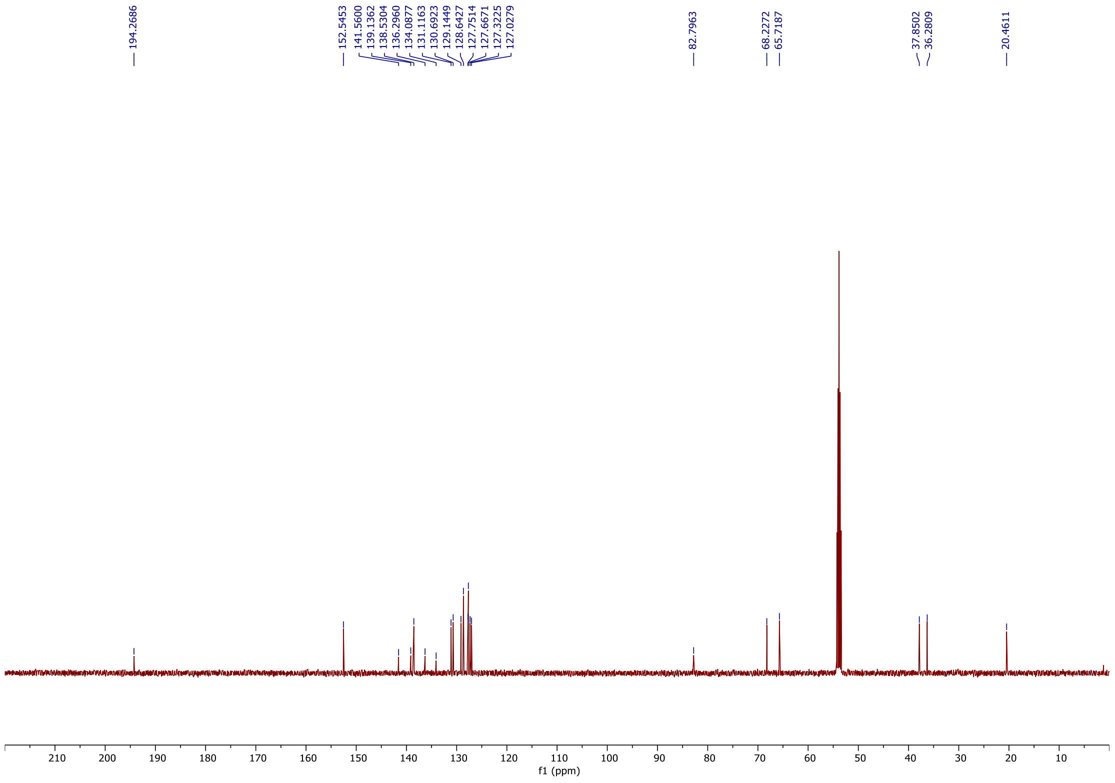
194.2686
152.5453 141.5600 139.1362 138.5304 136.2960 134.0877 131.1163 130.6923 129.1449 128.6427 127.7514 127.6671 127.3225 127.0279
82.7963
68.2272 65.7187
37.8502 36.2809
20.4611
210 200 190 180 170 160 150 140 130 120 110 100 90 80 70 60 50 40 30 20 10
f1 (ppm)

3p

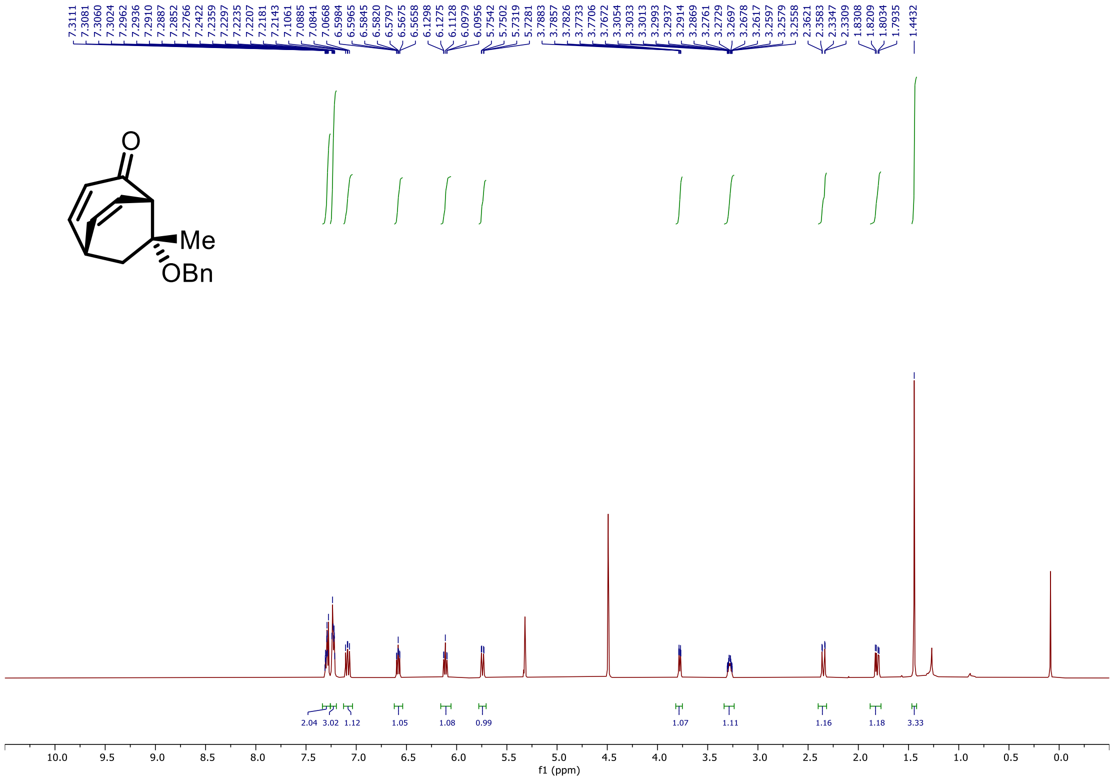
O
Me
OBn
7.3111
7.3081
7.3060
7.3024
7.2962
7.2936
7.2910
7.2887
7.2852
7.2766
7.2422
7.2359
7.2297
7.2235
7.2207
7.2181
7.2143
7.1061
7.0885
7.0841
7.0668
6.5984
6.5965
6.5845
6.5820
6.5797
6.5675
6.5558
6.1298
6.1275
6.1128
6.0979
6.0956
5.7542
5.7502
5.7319
5.7281
3.7883
3.7857
3.7826
3.7733
3.7706
3.7672
3.3054
3.3033
3.3013
3.2993
3.2937
3.2914
3.2869
3.2761
3.2729
3.2697
3.2678
3.2617
3.2597
3.2579
3.2558
2.3621
2.3583
2.3347
2.3309
1.8308
1.8209
1.8034
1.7935
1.4432
2.04
3.02
1.12
1.05
1.08
0.99
1.07
1.11
1.16
1.18
3.33
10.0
9.5
9.0
8.5
8.0
7.5
7.0
6.5
6.0
5.5
5.0
4.5
4.0
3.5
3.0
2.5
2.0
1.5
1.0
0.5
0.0
f1 (ppm)

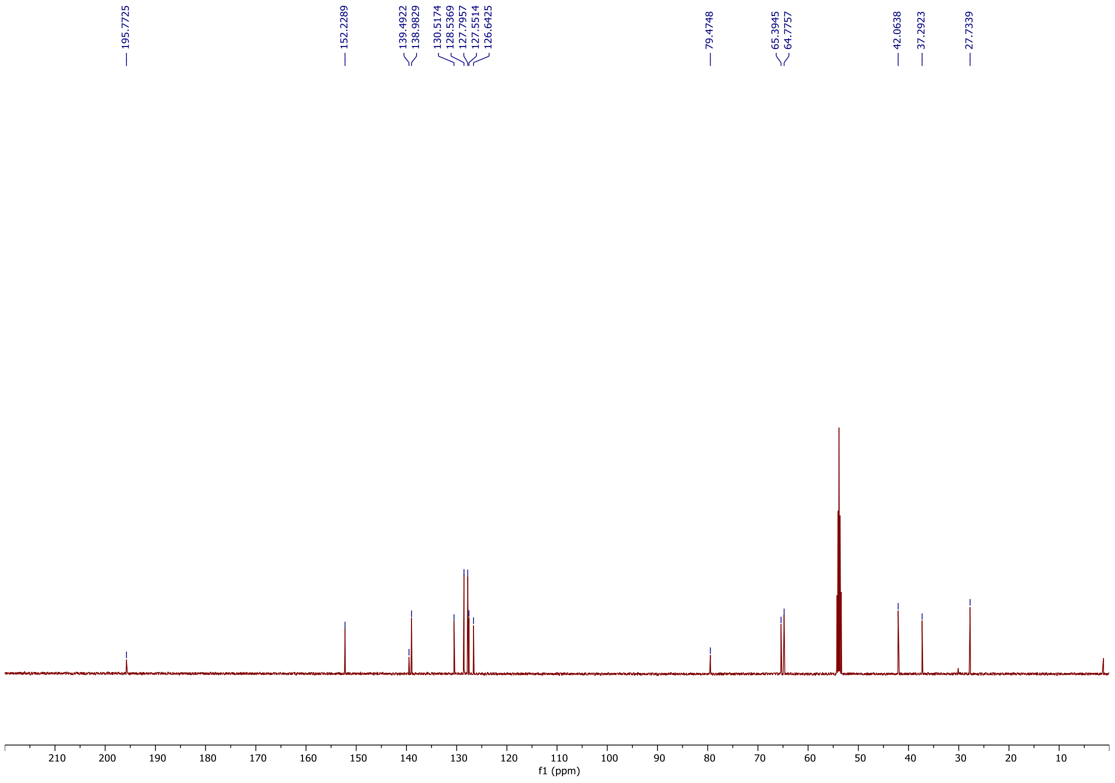
195.7725
152.2289
139.4922
138.9829
130.5174
128.5369
127.7957
127.5514
126.6425
79.4748
65.3945
64.7757
42.0638
37.2923
27.7339
210
200
190
180
170
160
150
140
130
120
110
100
90
80
70
60
50
40
30
20
10
f1 (ppm)

3p-2

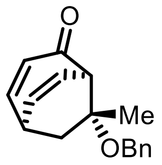
O
Me
OBn

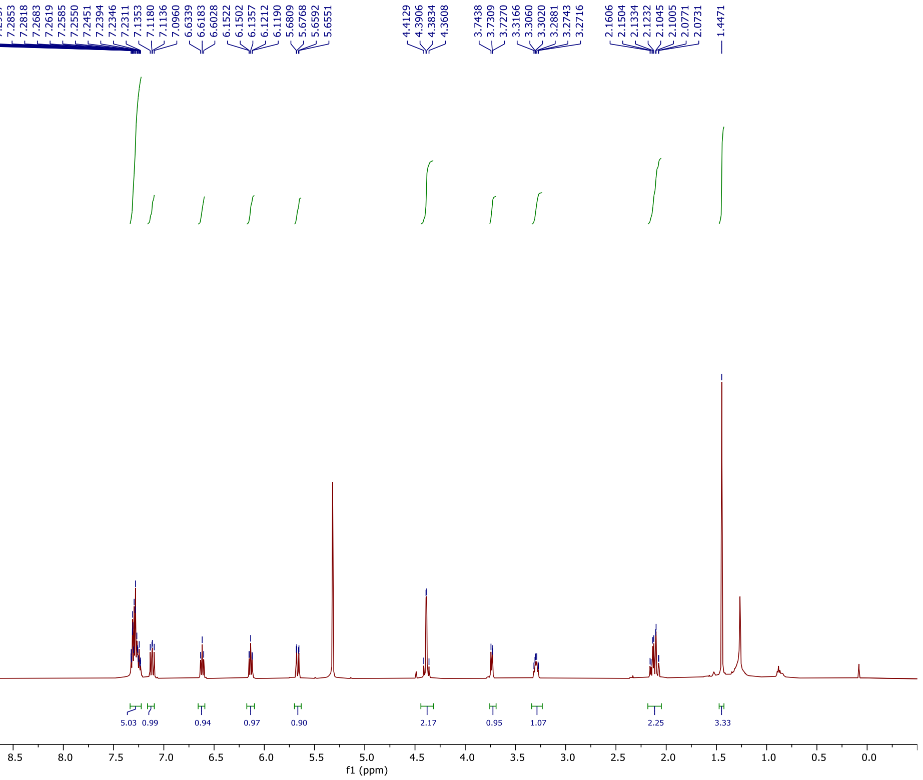
7.3270
7.3246
7.3214
7.3124
7.3101
7.3078
7.3009
7.2957
7.2853
7.2818
7.2683
7.2619
7.2585
7.2550
7.2451
7.2394
7.2346
7.2311
7.1353
7.1180
7.1136
7.0960
6.6339
6.6183
6.6028
6.1522
6.1502
6.1357
6.1212
6.1190
5.6809
5.6768
5.6592
5.6551
4.4129
4.3906
4.3834
4.3608
3.7438
3.7309
3.7270
3.3166
3.3060
3.3020
3.2881
3.2743
3.2716
2.1606
2.1504
2.1334
2.1232
2.1045
2.1005
2.0771
2.0731
1.4471
5.03
0.99
0.94
0.97
0.90
2.17
0.95
1.07
2.25
3.33
10.0
9.5
9.0
8.5
8.0
7.5
7.0
6.5
6.0
5.5
5.0
4.5
4.0
3.5
3.0
2.5
2.0
1.5
1.0
0.5
0.0
f1 (ppm)

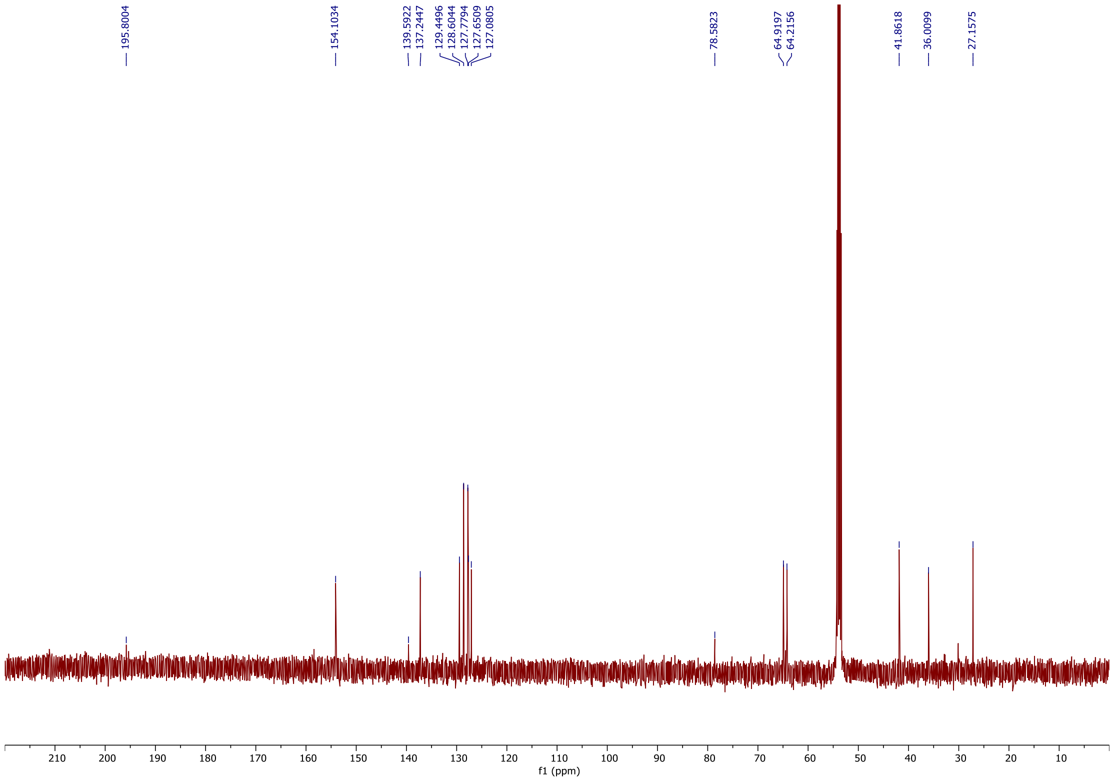
195.8004
154.1034
139.5922
137.2447
129.4496
128.6044
127.7794
127.6509
127.0805
78.5823
64.9197
64.2156
41.8618
36.0099
27.1575
210
200
190
180
170
160
150
140
130
120
110
100
90
80
70
60
50
40
30
20
10
f1 (ppm)

16. HPLC traces

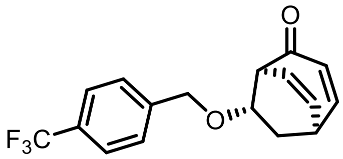

3a

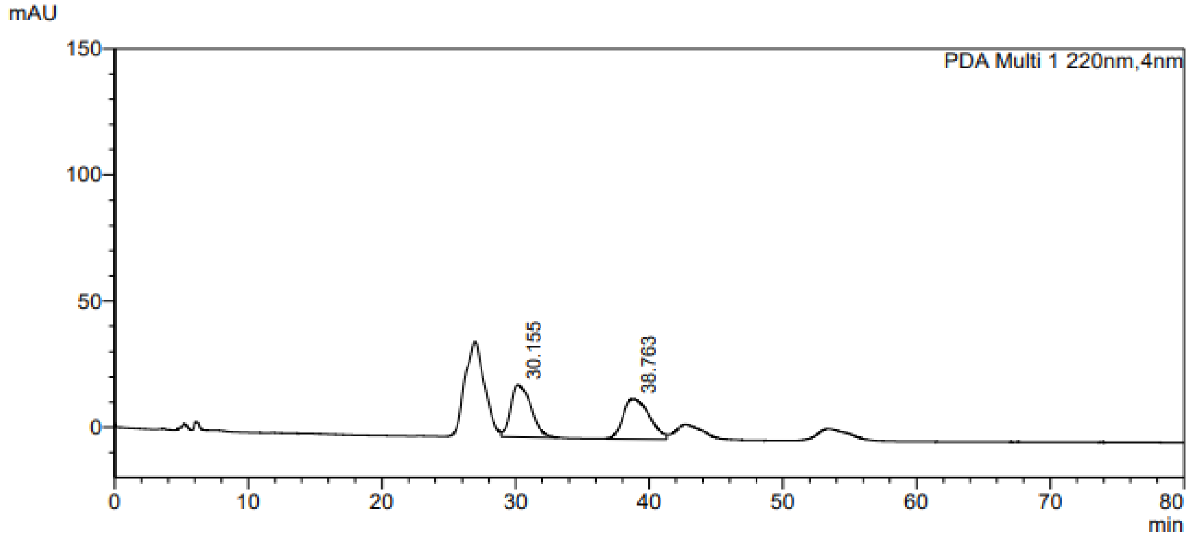

<Peak Table>

PDA Ch1 220nm

Peak#	Ret. Time	Area	Area%
1	30.155	2179803	50.023
2	38.763	2177807	49.977
Total		4357610	100.000

<Chromatogram>

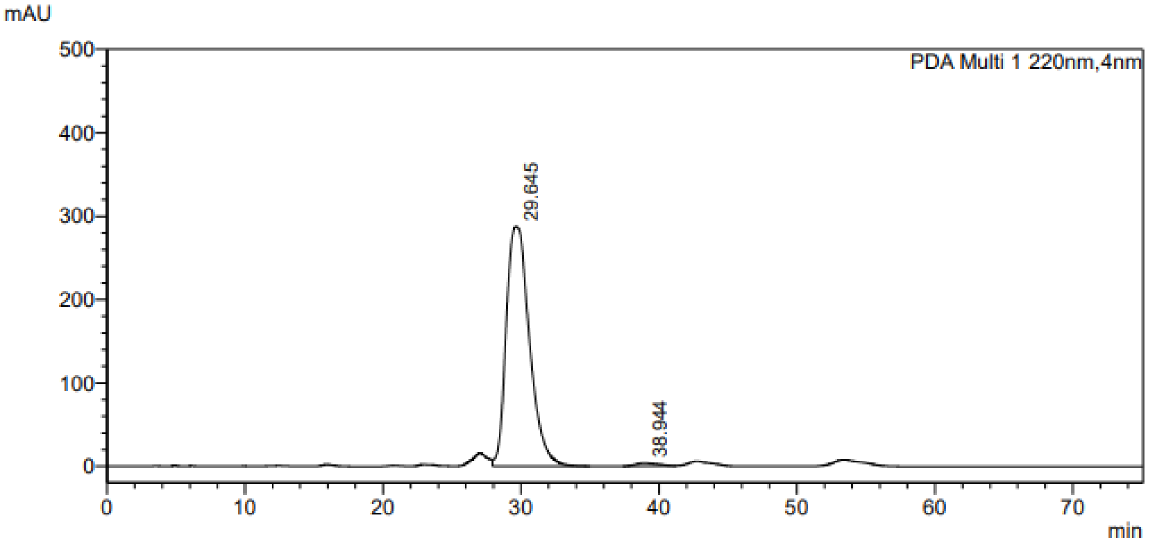

<Peak Table>

PDA Ch1 220nm

Peak#	Ret. Time	Area	Area%
1	29.645	33766864	98.863
2	38.944	388446	1.137
Total		34155310	100.000

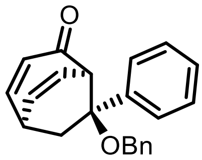

3b

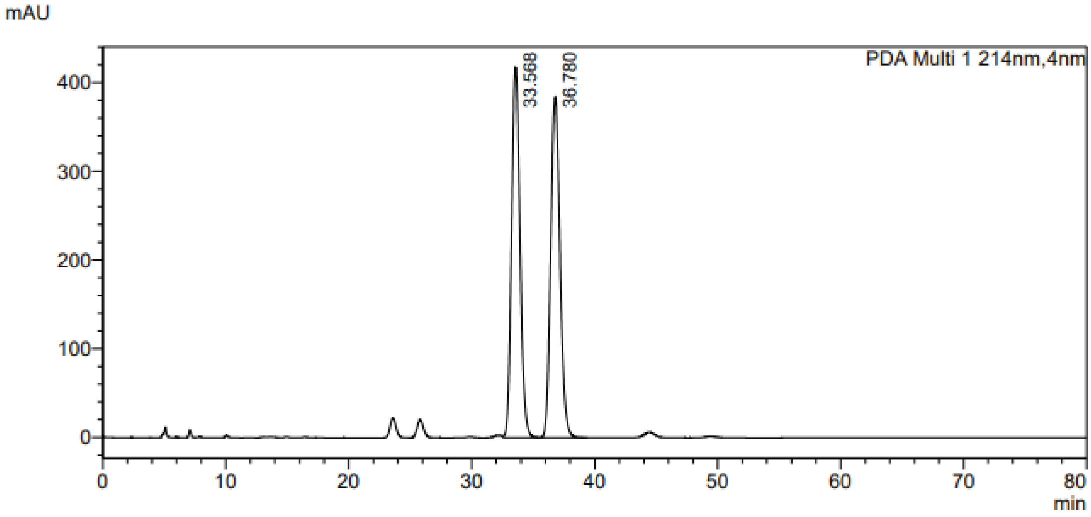

<Peak Table>

PDA Ch1 214nm

Peak#	Ret. Time	Area	Area%
1	33.568	19734056	49.654
2	36.780	20008696	50.346
Total		39742752	100.000

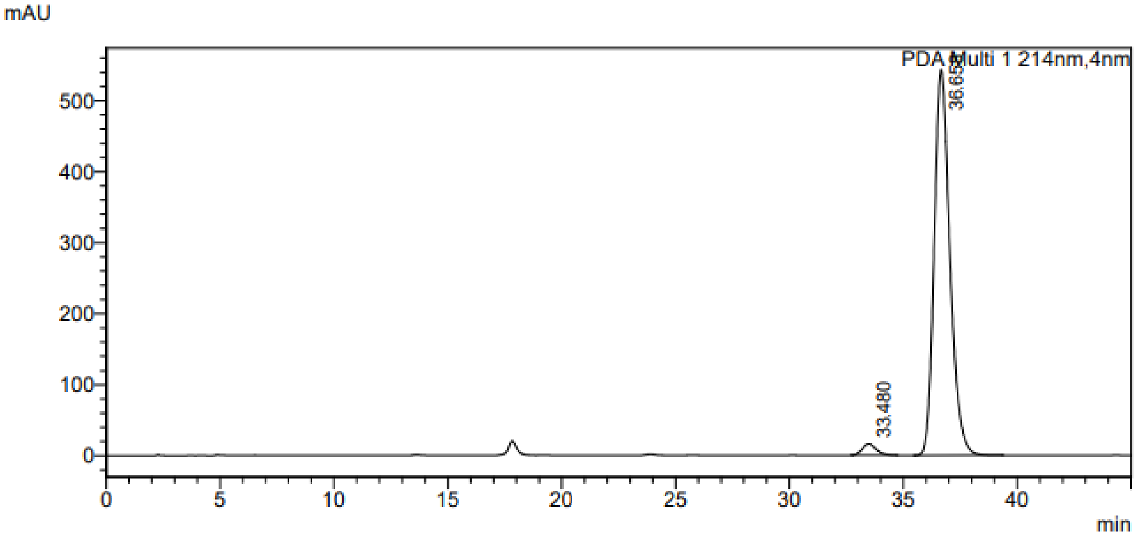

<Peak Table>

PDA Ch1 214nm

Peak#	Ret. Time	Area	Area%
1	33.480	686112	2.549
2	36.658	26228596	97.451
Total		26914707	100.000

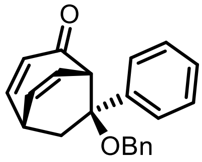

3b-2

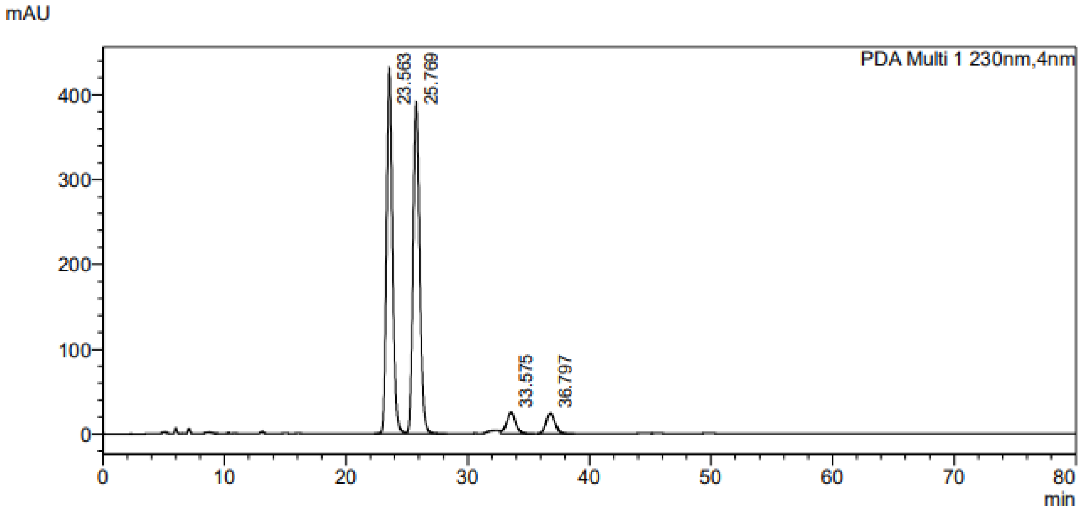

<Peak Table>

PDA Ch1 230nm

Peak#	Ret. Time	Area	Area%
1	23.563	14839817	46.017
2	25.769	14810562	45.927
3	33.575	1332150	4.131
4	36.797	1265789	3.925
Total		32248319	100.000

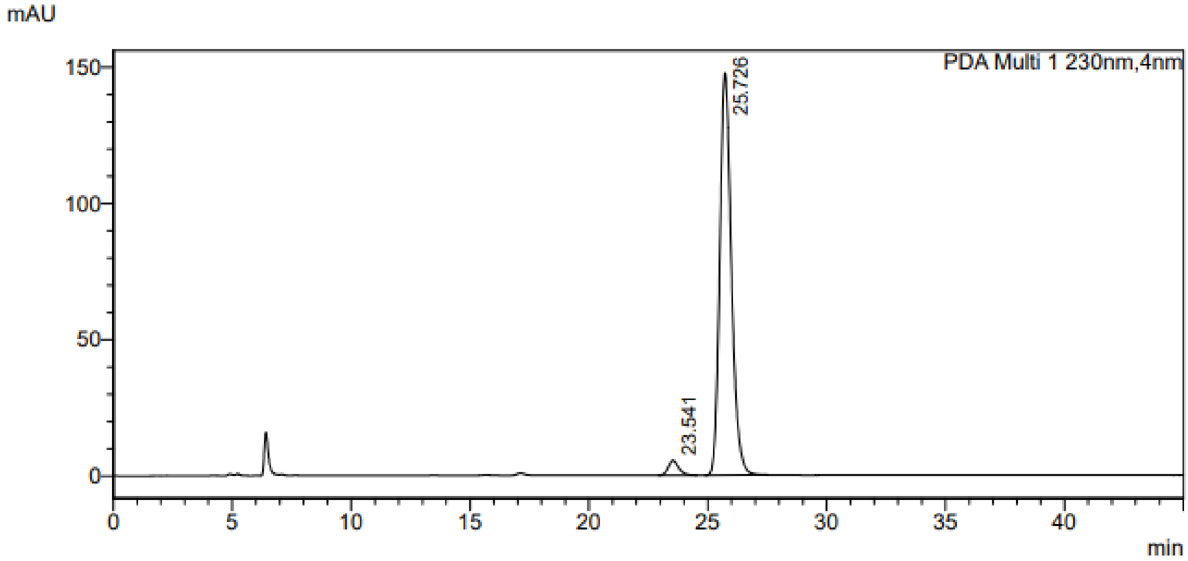

<Peak Table>

PDA Ch1 230nm

Peak#	Ret. Time	Area	Area%
1	23.541	163776	3.157
2	25.726	5024088	96.843
Total		5187863	100.000

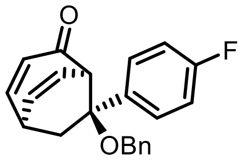

3c

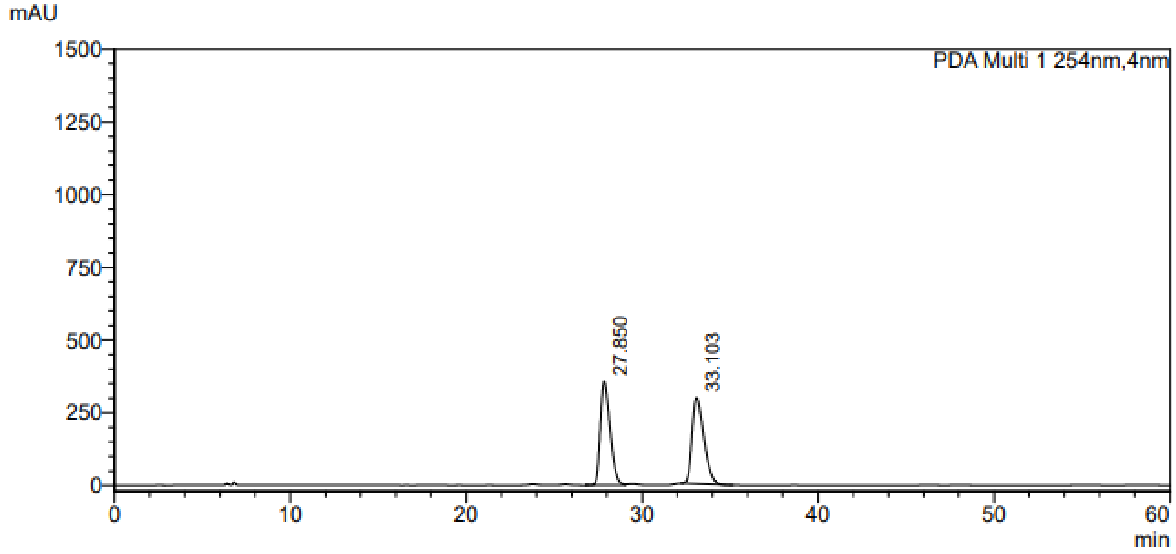

<Peak Table>

PDA Ch1 254nm

Peak#	Ret. Time	Area	Area%
1	27.850	13998877	49.909
2	33.103	14049653	50.091
Total		28048529	100.000

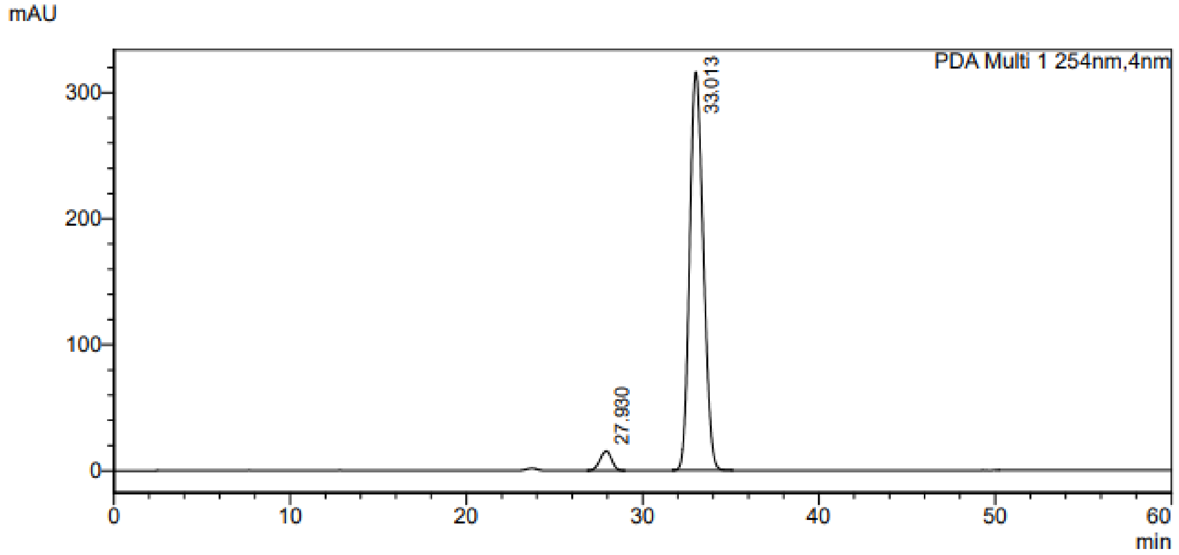

<Peak Table>

PDA Ch1 254nm

Peak#	Ret. Time	Area	Area%
1	27.930	700486	3.965
2	33.013	16966082	96.035
Total		17666568	100.000

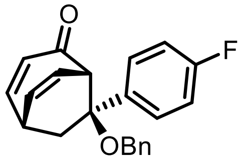

3c-2

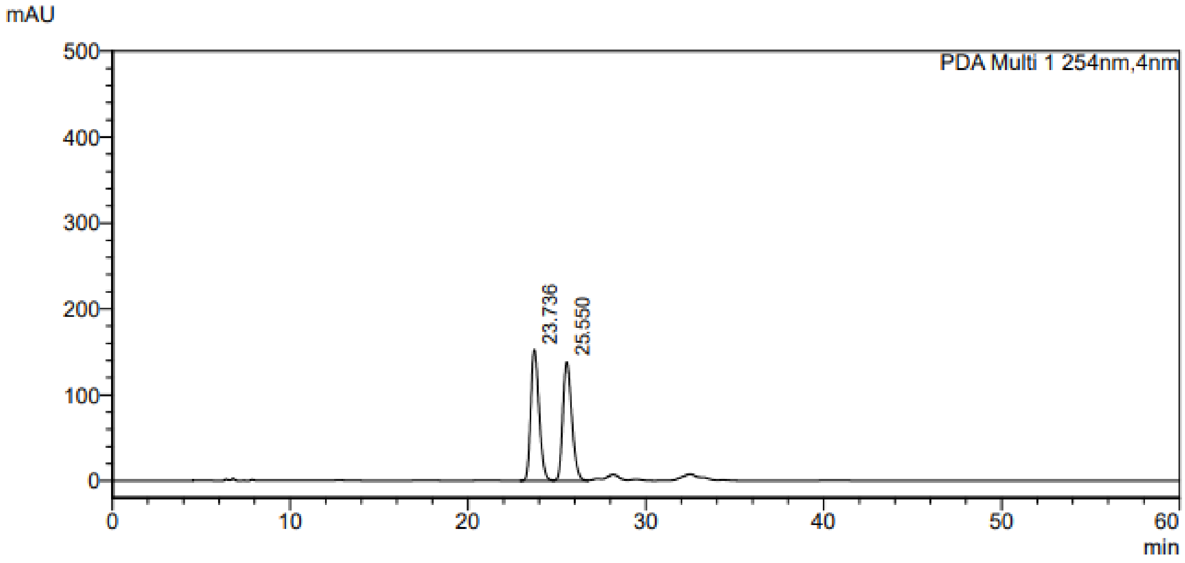

<Peak Table>

PDA Ch1 254nm

Peak#	Ret. Time	Area	Area%
1	23.736	4979313	50.287
2	25.550	4922414	49.713
Total		9901727	100.000

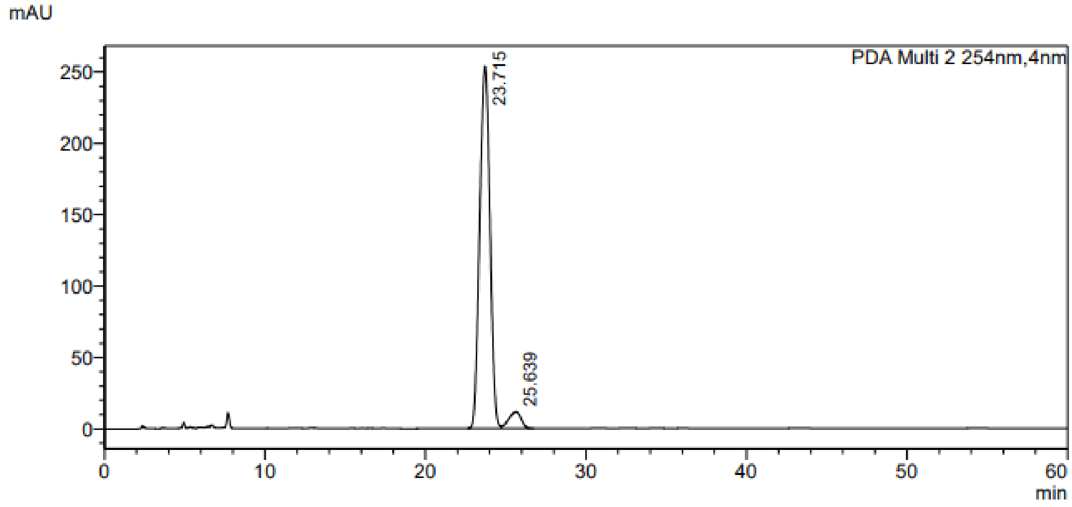

<Peak Table>

PDA Ch2 254nm

Peak#	Ret. Time	Area	Area%
1	23.715	11380800	94.859
2	25.639	616779	5.141
Total		11997580	100.000

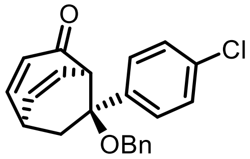

3d

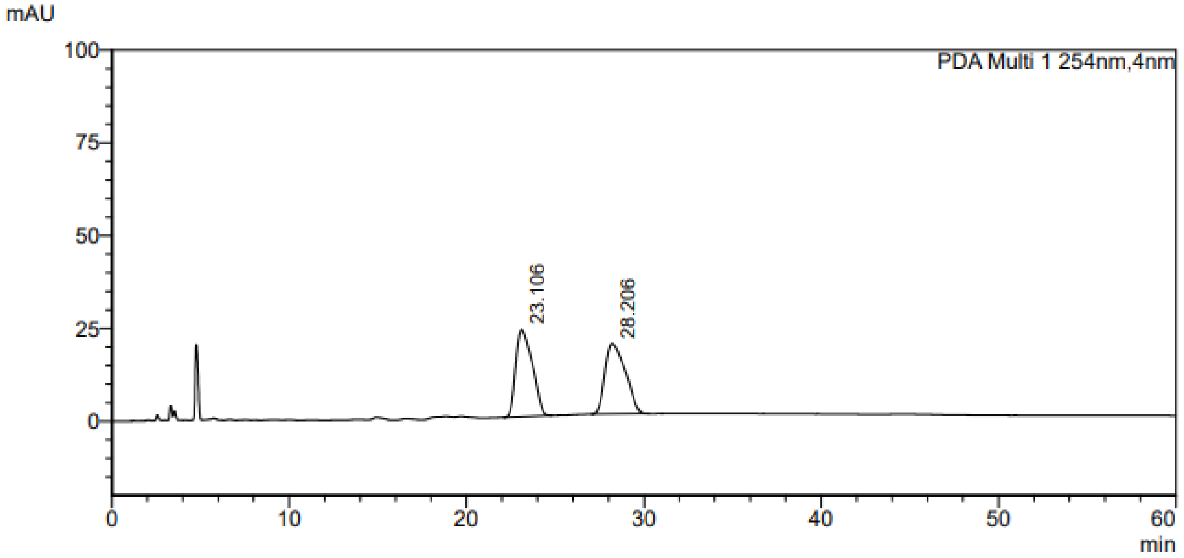

<Peak Table>

PDA Ch1 254nm

Peak#	Ret. Time	Area	Area%
1	23.106	1526202	50.194
2	28.206	1514424	49.806
Total		3040625	100.000

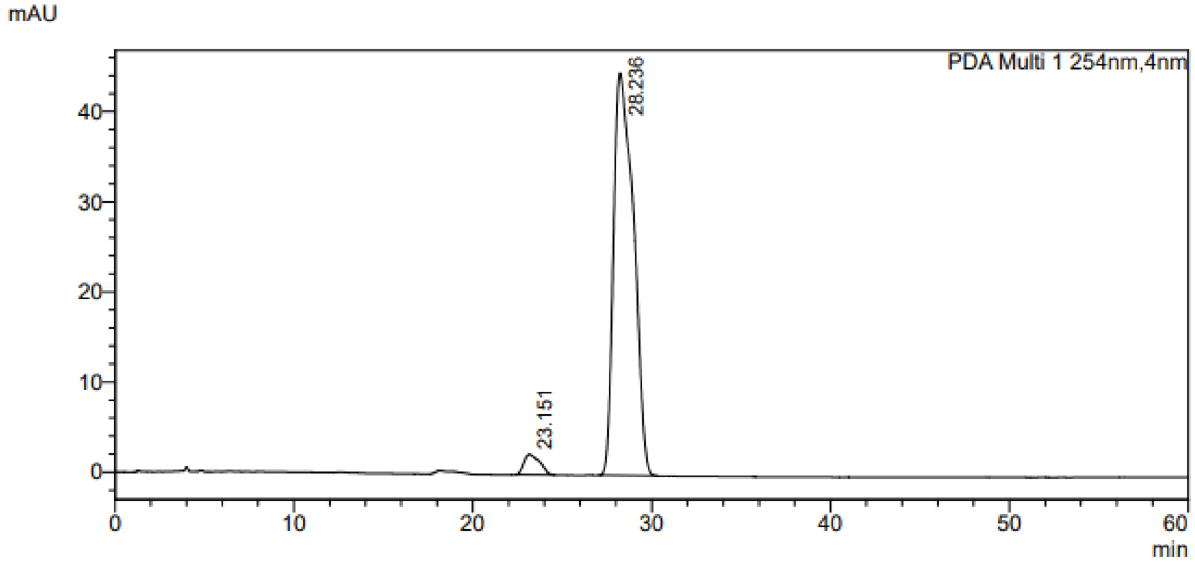

<Peak Table>

PDA Ch1 254nm

Peak#	Ret. Time	Area	Area%
1	23.151	138297	3.844
2	28.236	3459663	96.156
Total		3597960	100.000

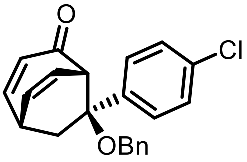

3d-2

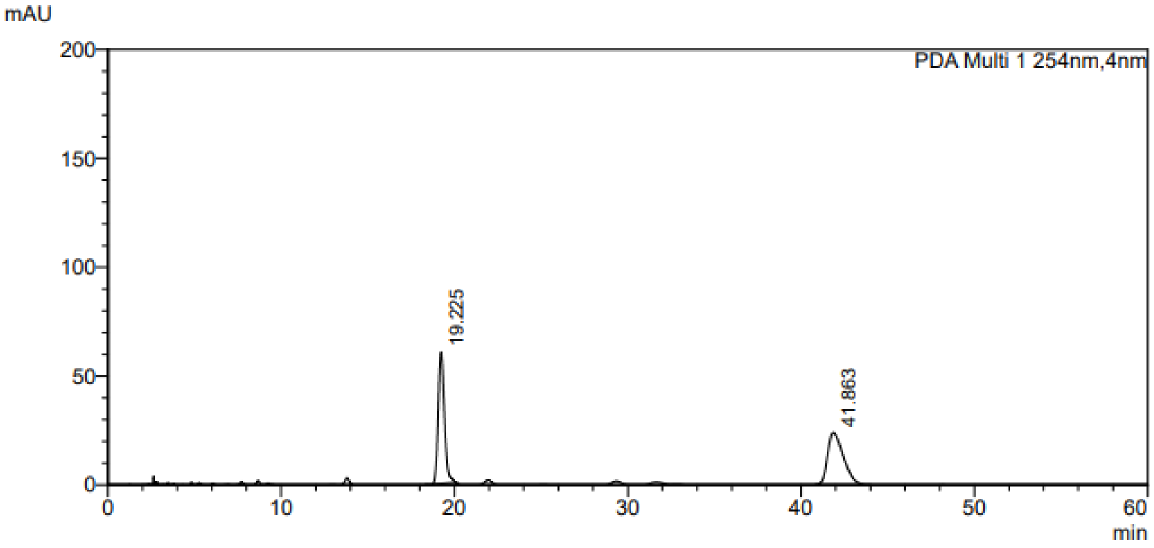

<Peak Table>

PDA Ch1 254nm

Peak#	Ret. Time	Area	Area%
1	19.225	1507758	49.733
2	41.863	1523949	50.267
Total		3031707	100.000

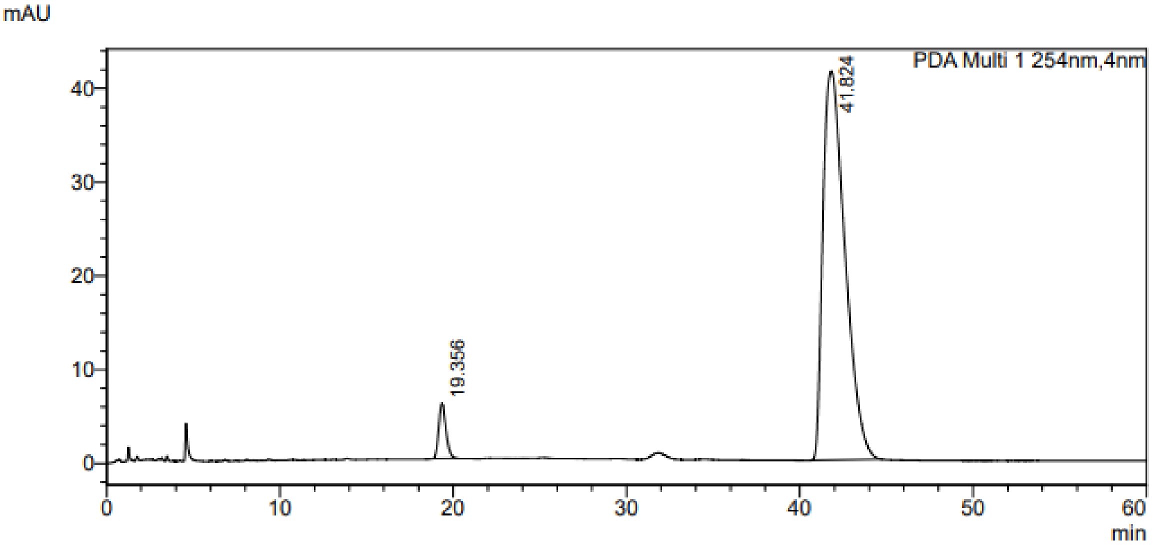

<Peak Table>

PDA Ch1 254nm

Peak#	Ret. Time	Area	Area%
1	19.356	169456	4.407
2	41.824	3675308	95.593
Total		3844764	100.000

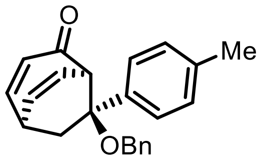

3e

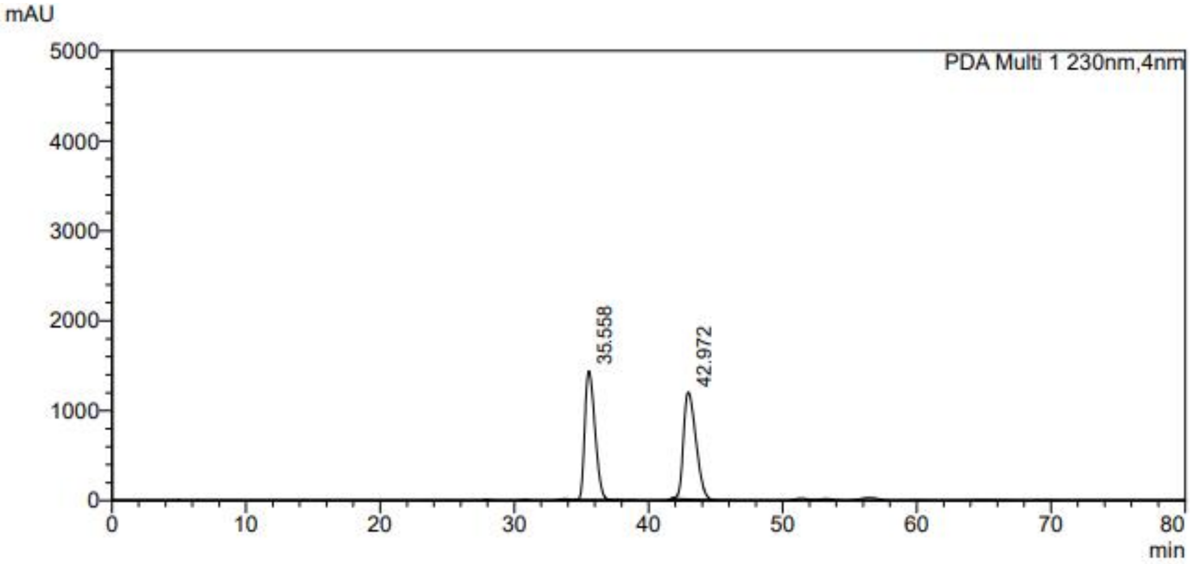

<Peak Table>

PDA Ch1 230nm

Peak#	Ret. Time	Area	Area%
1	35.558	75789559	49.587
2	42.972	77051416	50.413
Total		152840975	100.000

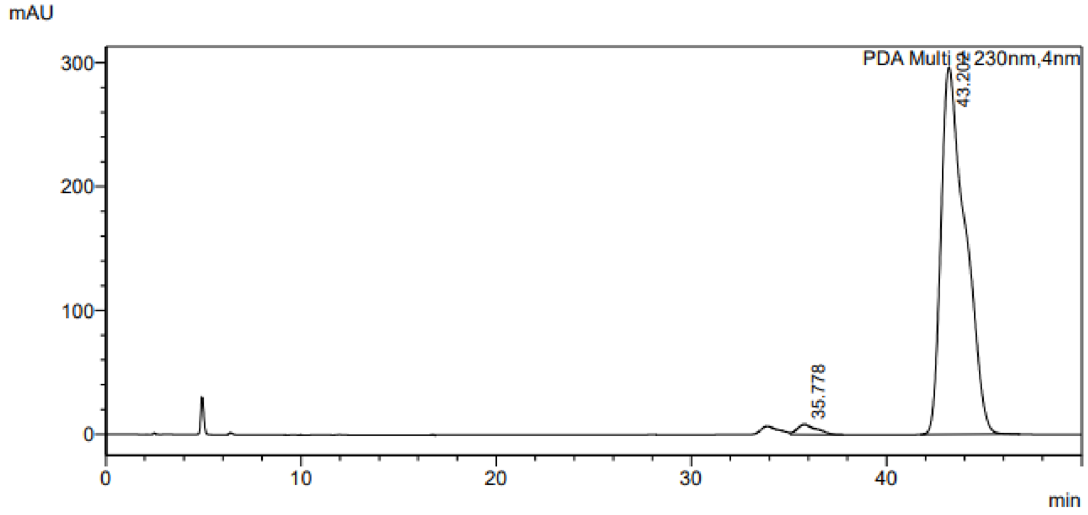

<Peak Table>

PDA Ch1 230nm

Peak#	Ret. Time	Area	Area%
1	35.778	580484	2.208
2	43.202	25706833	97.792
Total		26287317	100.000

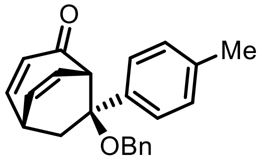

3e-2

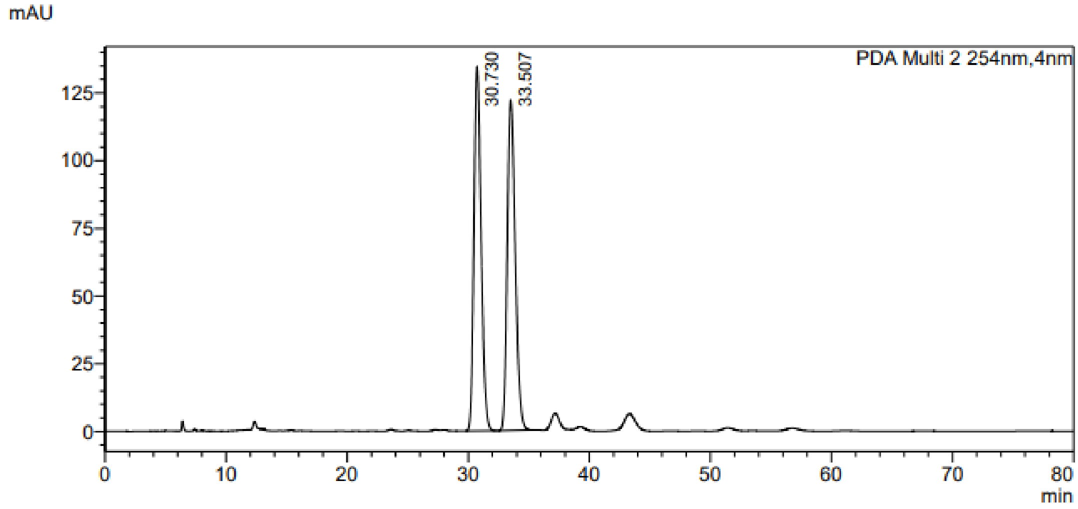

<Peak Table>

PDA Ch2 254nm

Peak#	Ret. Time	Area	Area%
1	30.730	5578540	49.857
2	33.507	5610477	50.143
Total		11189017	100.000

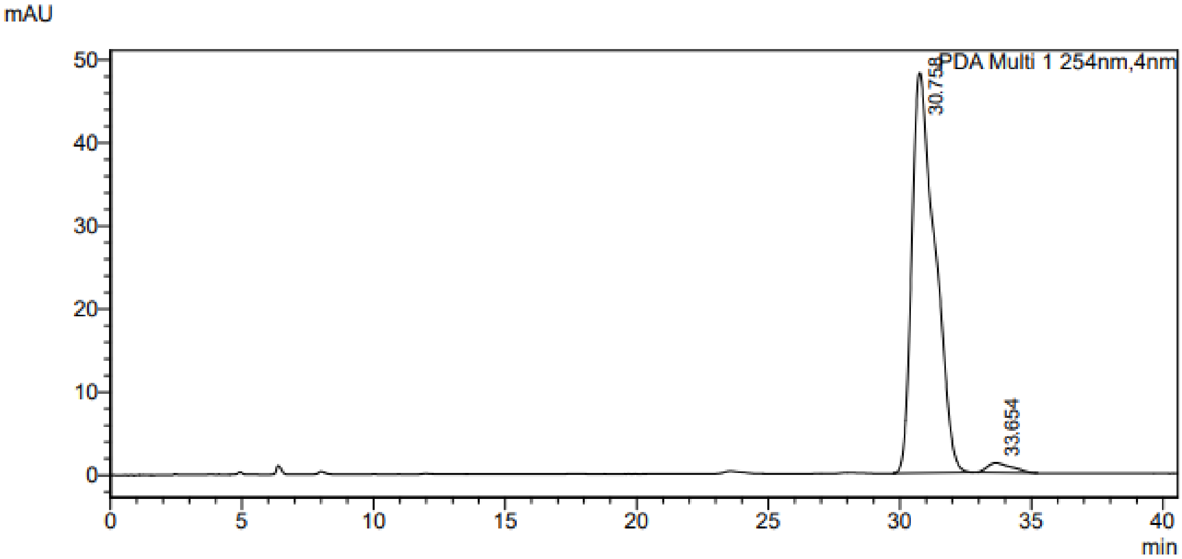

<Peak Table>

PDA Ch1 254nm

Peak#	Ret. Time	Area	Area%
1	30.758	2945466	97.556
2	33.654	73788	2.444
Total		3019254	100.000

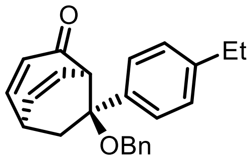

3f

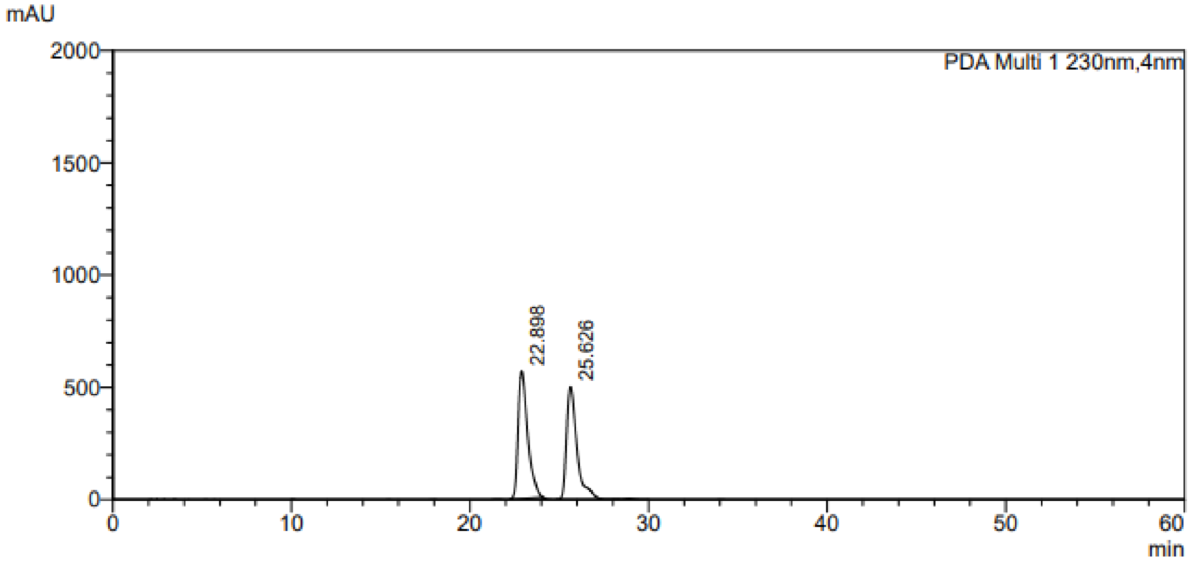

<Peak Table>

PDA Ch1 230nm

Peak#	Ret. Time	Area	Area%
1	22.898	21375708	50.494
2	25.626	20957661	49.506
Total		42333368	100.000

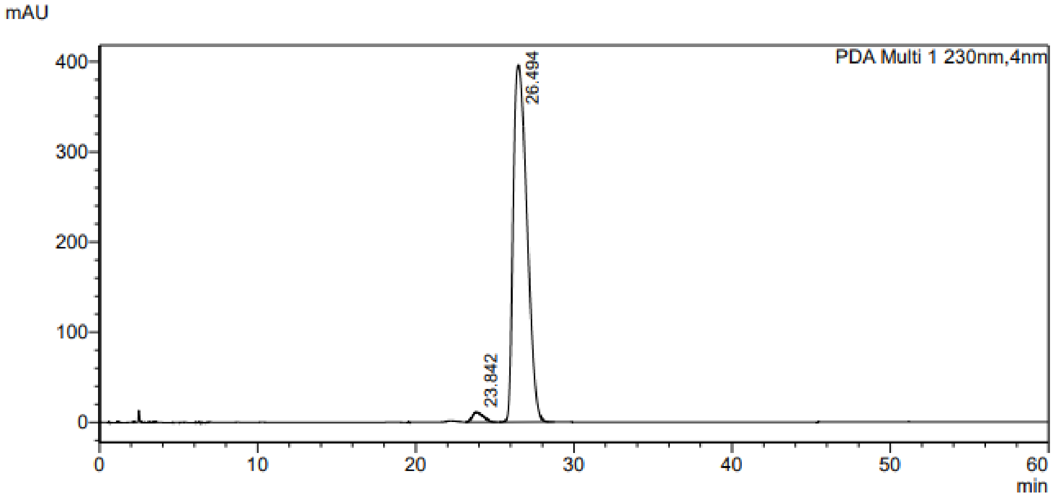

<Peak Table>

PDA Ch1 230nm

Peak#	Ret. Time	Area	Area%
1	23.842	527449	2.162
2	26.494	23867145	97.838
Total		24394594	100.000

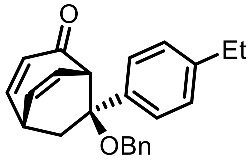

3f-2

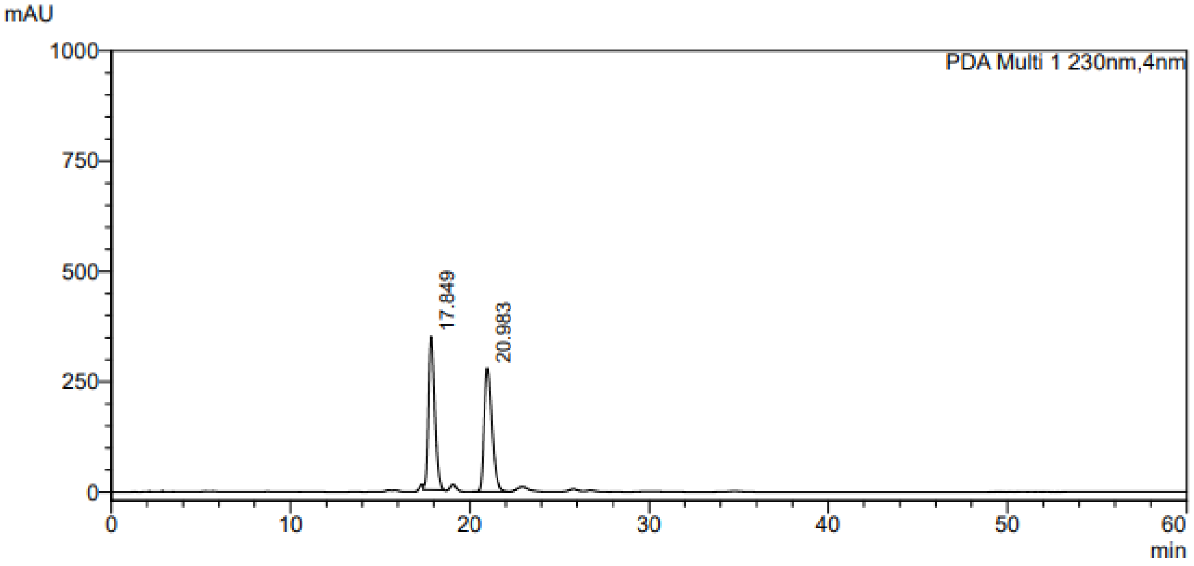

<Peak Table>

PDA Ch1 230nm

Peak#	Ret. Time	Area	Area%
1	17.849	8653243	49.955
2	20.983	8668931	50.045
Total		17322174	100.000

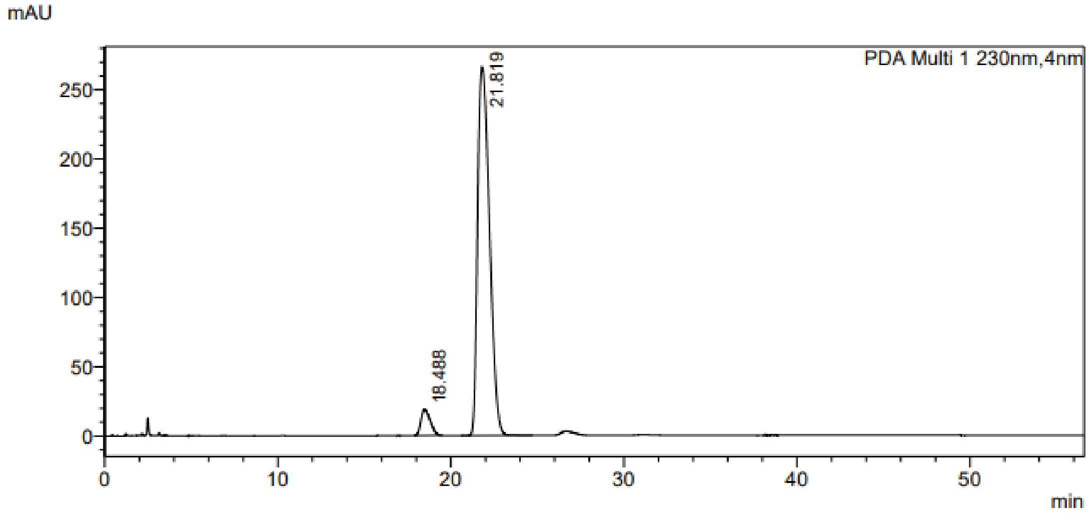

<Peak Table>

PDA Ch1 230nm

Peak#	Ret. Time	Area	Area%
1	18.488	700032	5.202
2	21.819	12755718	94.798
Total		13455749	100.000

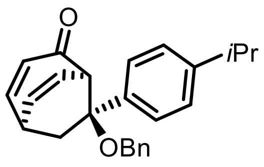

3g

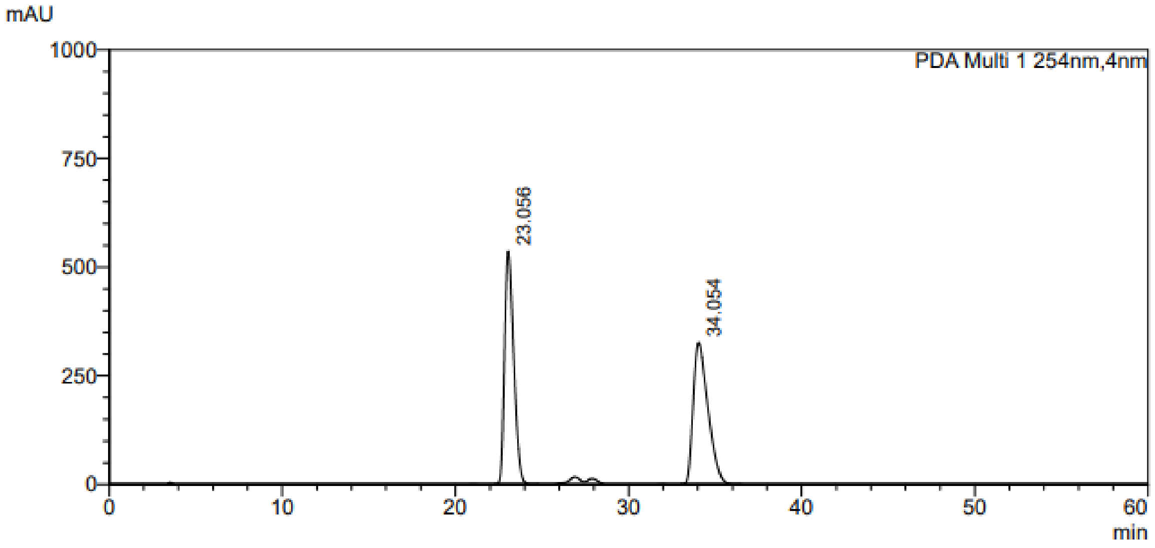

<Peak Table>

PDA Ch1 254nm

Peak#	Ret. Time	Area	Area%
1	23.056	19741705	50.305
2	34.054	19501971	49.695
Total		39243676	100.000

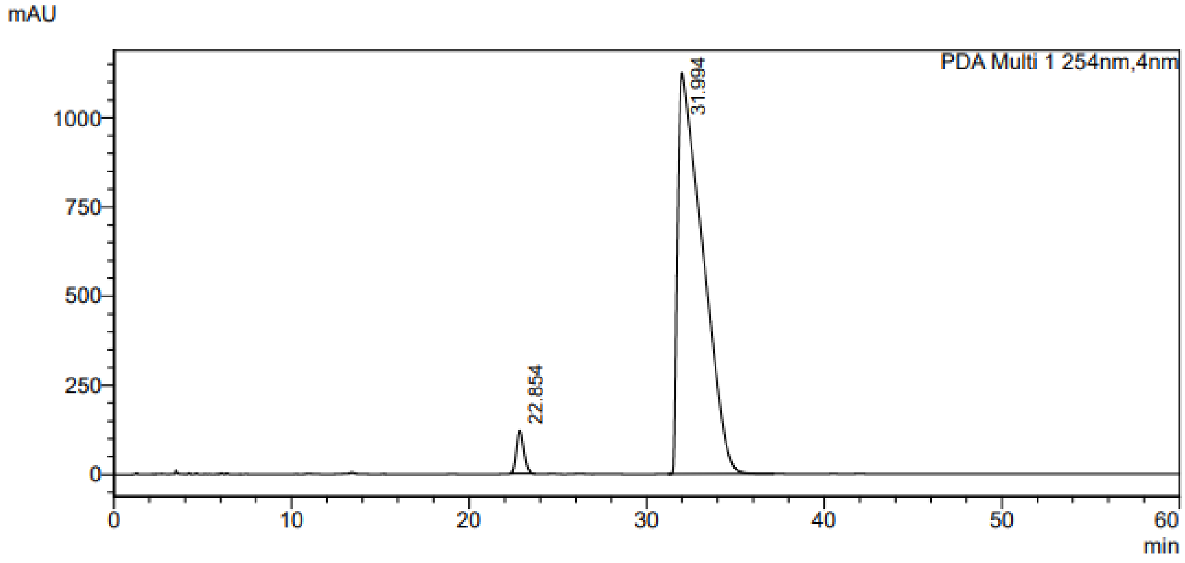

<Peak Table>

PDA Ch1 254nm

Peak#	Ret. Time	Area	Area%
1	22.854	3525510	3.110
2	31.994	109843487	96.890
Total		113368997	100.000

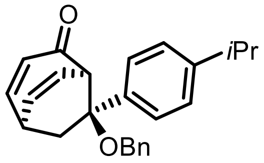

3g-2

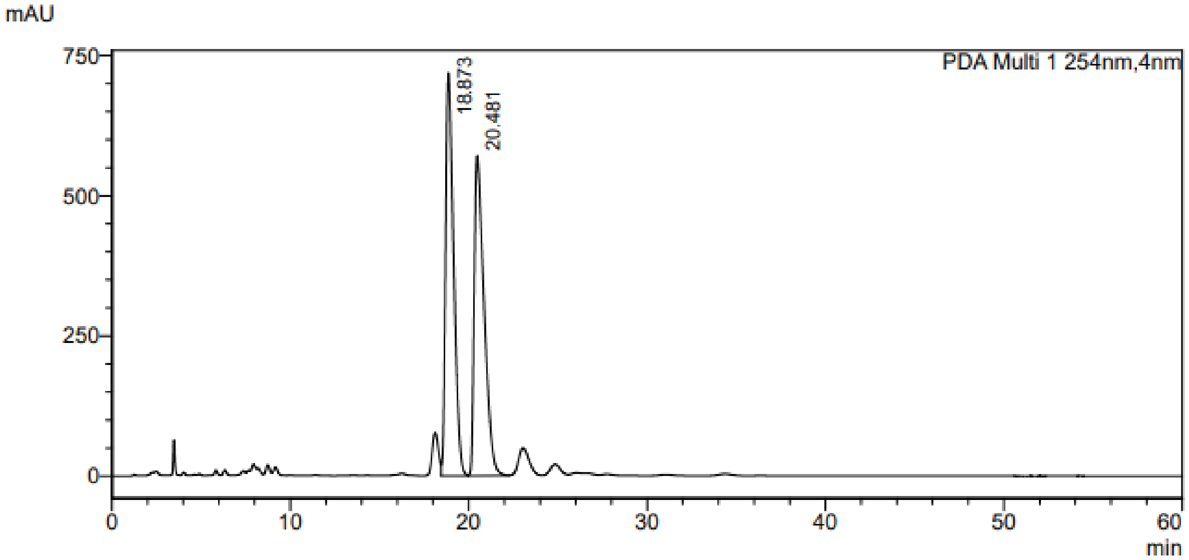

<Peak Table>

PDA Ch1 254nm

Peak#	Ret. Time	Area	Area%
1	18.873	22461479	50.766
2	20.481	21783785	49.234
Total		44245264	100.000

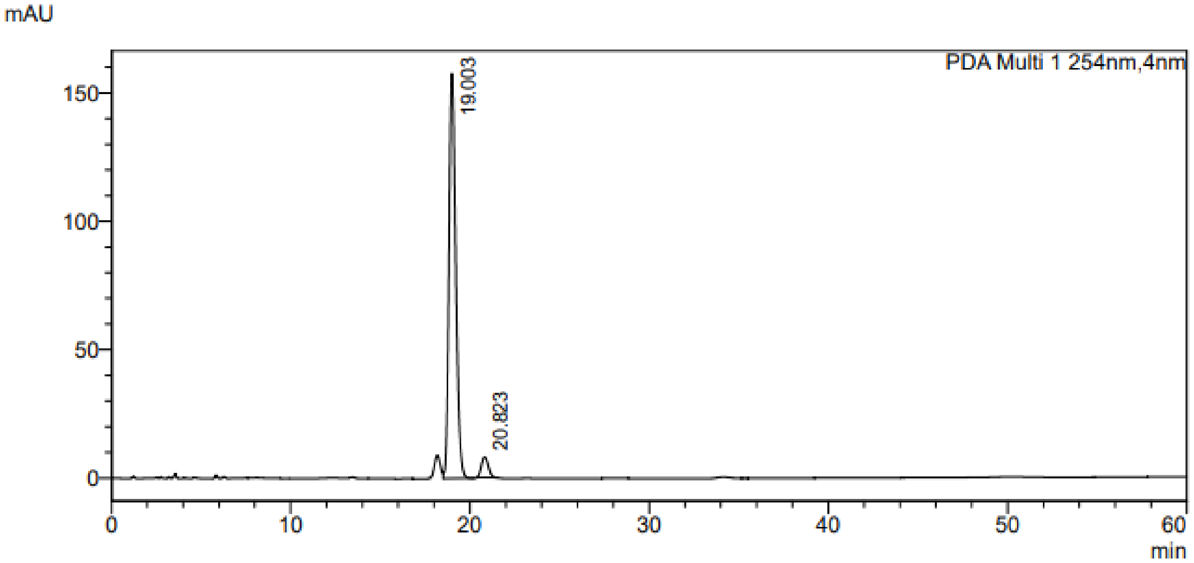

<Peak Table>

PDA Ch1 254nm

Peak#	Ret. Time	Area	Area%
1	19.003	4154933	95.122
2	20.823	213075	4.878
Total		4368008	100.000

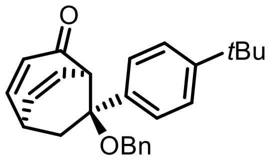

3h

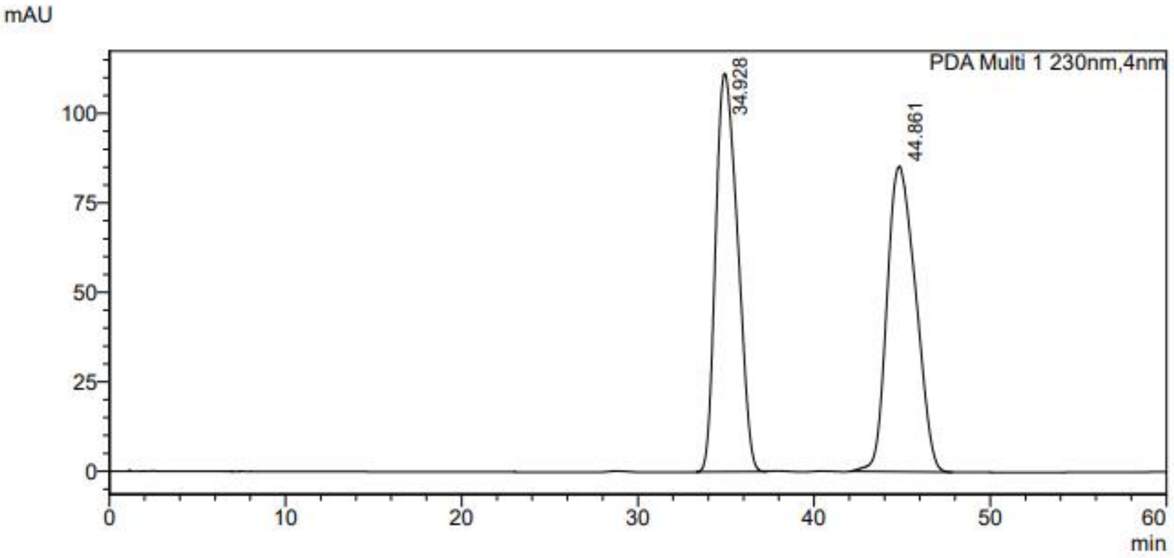

<Peak Table>

PDA Ch1 230nm

Peak#	Ret. Time	Area	Area%
1	34.928	9628209	49.939
2	44.861	9651579	50.061
Total		19279788	100.000

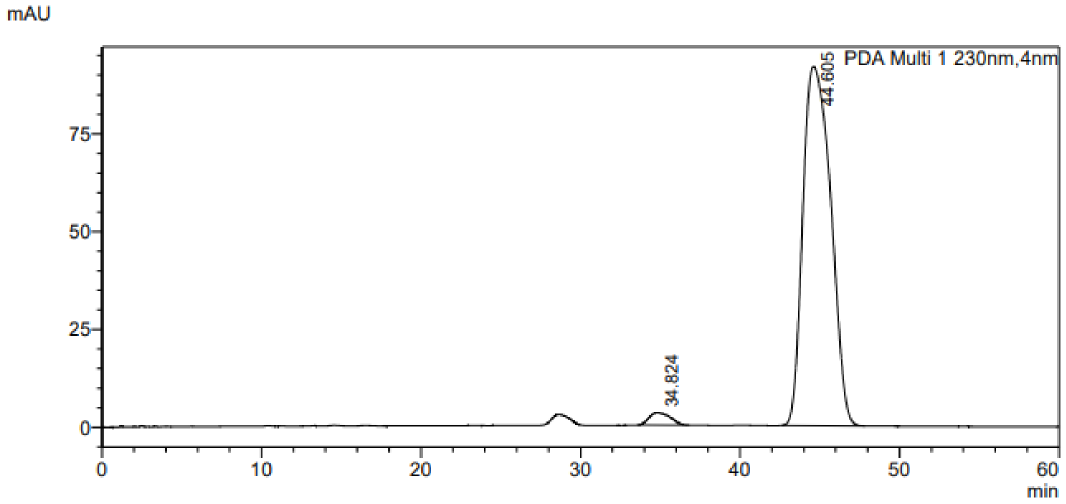

<Peak Table>

PDA Ch1 230nm

Peak#	Ret. Time	Area	Area%
1	34.824	295279	2.571
2	44.605	11187550	97.429
Total		11482829	100.000

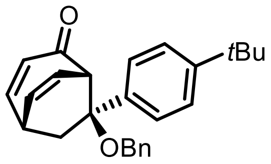

3h-2

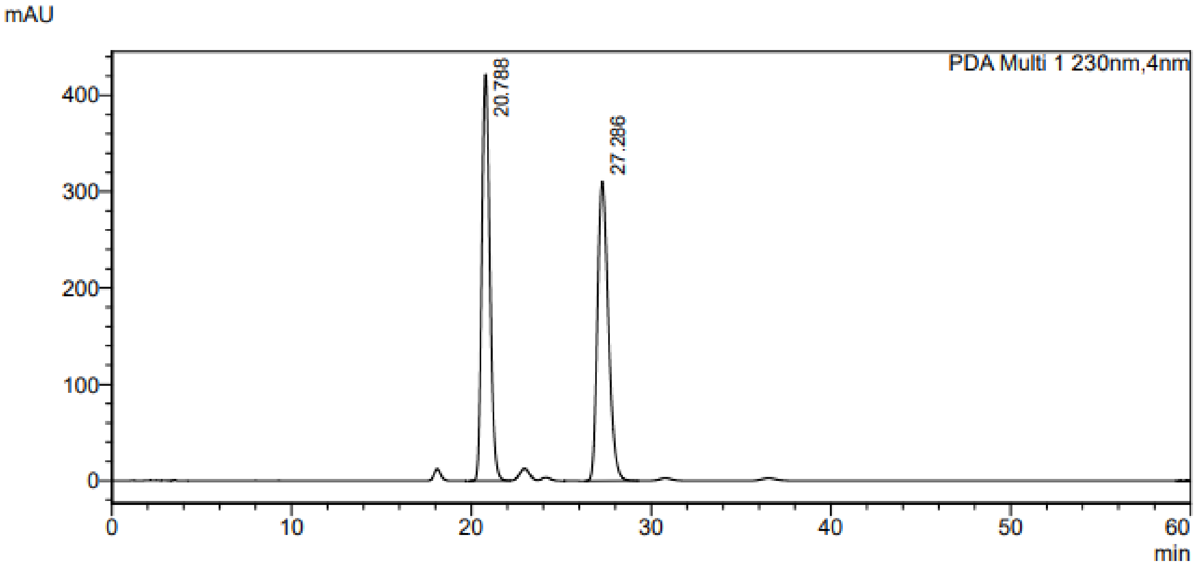

<Peak Table>

PDA Ch1 230nm

Peak#	Ret. Time	Area	Area%
1	20.788	13098431	50.010
2	27.286	13093245	49.990
Total		26191677	100.000

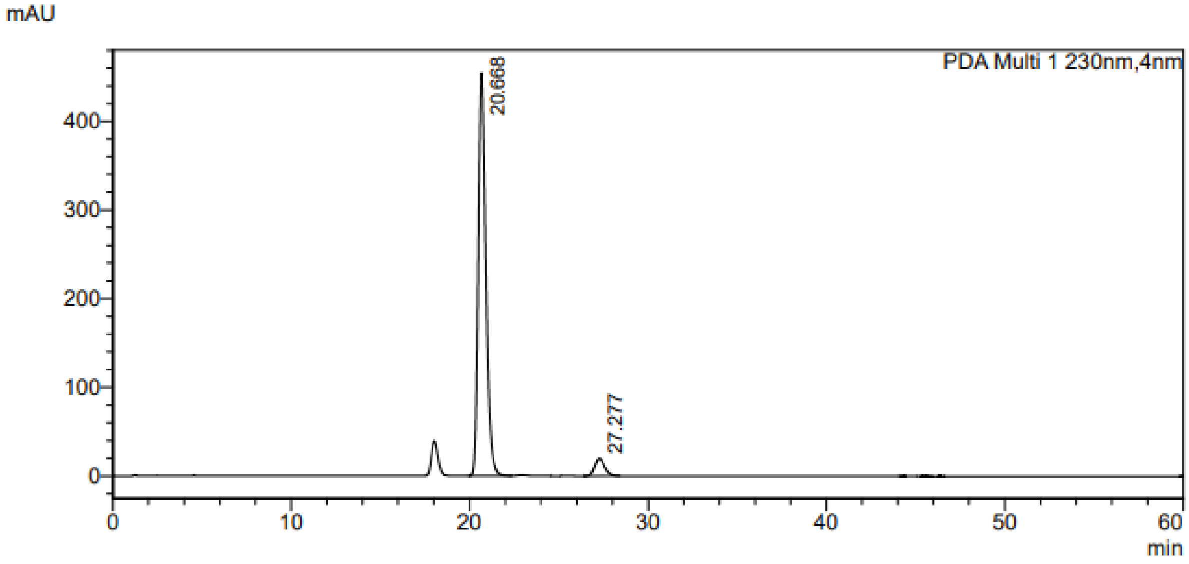

<Peak Table>

PDA Ch1 230nm

Peak#	Ret. Time	Area	Area%
1	20.668	13610183	94.804
2	27.277	746007	5.196
Total		14356190	100.000

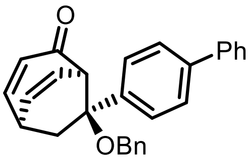

3i

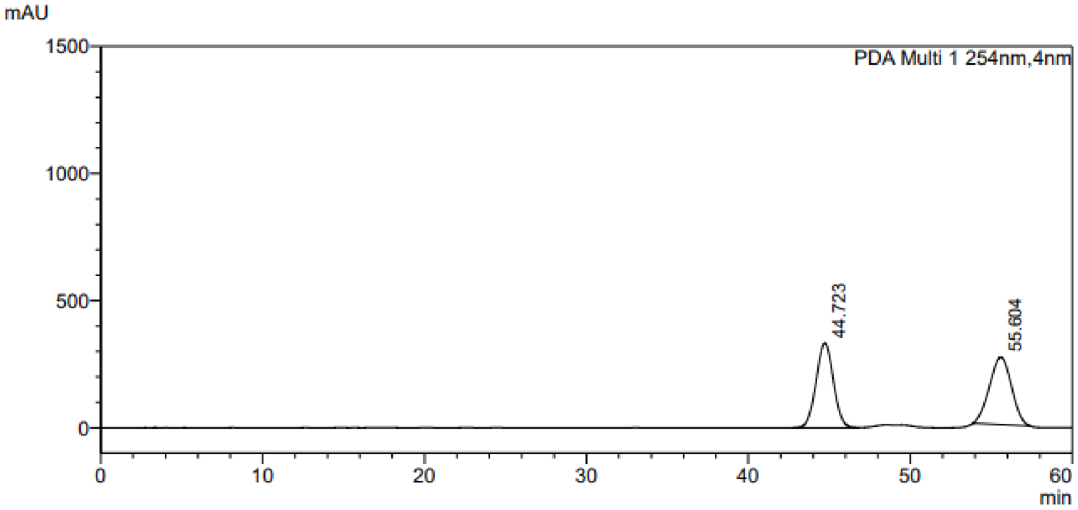

<Peak Table>

PDA Ch1 254nm

Peak#	Ret. Time	Area	Area%
1	44.723	24843375	49.815
2	55.604	25028106	50.185
Total		49871480	100.000

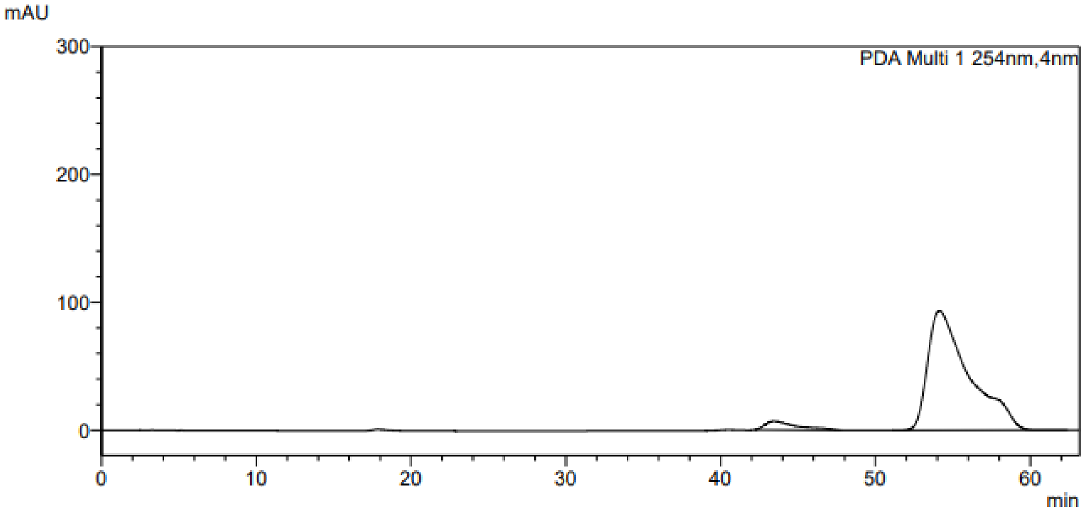

<Peak Table>

PDA Ch1 254nm

Peak#	Ret. Time	Area	Area%
1	43.439	948527	5.168
2	54.144	17405498	94.832
Total		18354025	100.000

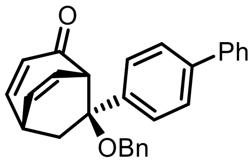

3i-2

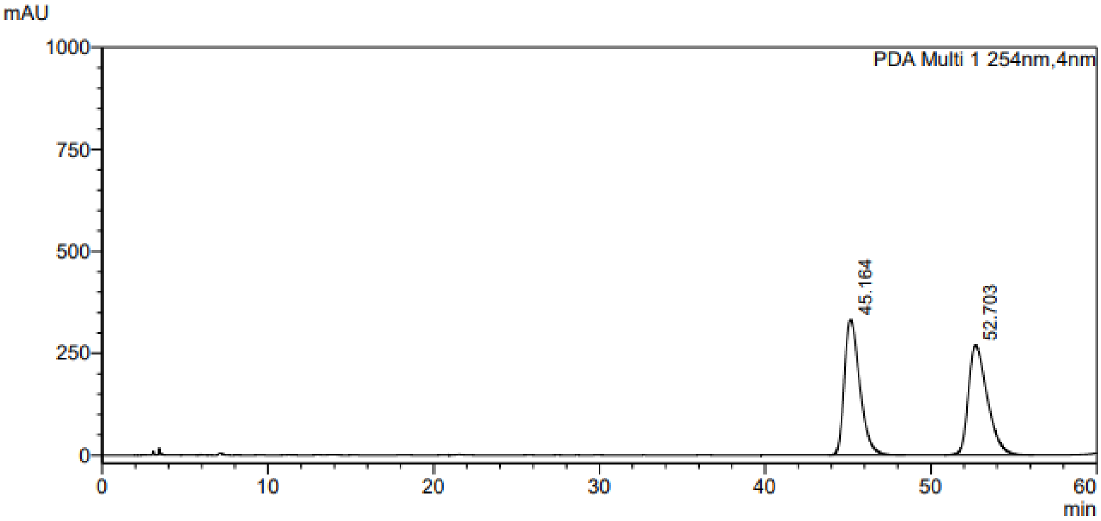

<Peak Table>

PDA Ch1 254nm

Peak#	Ret. Time	Area	Area%
1	45.164	21513527	50.081
2	52.703	21443586	49.919
Total		42957114	100.000

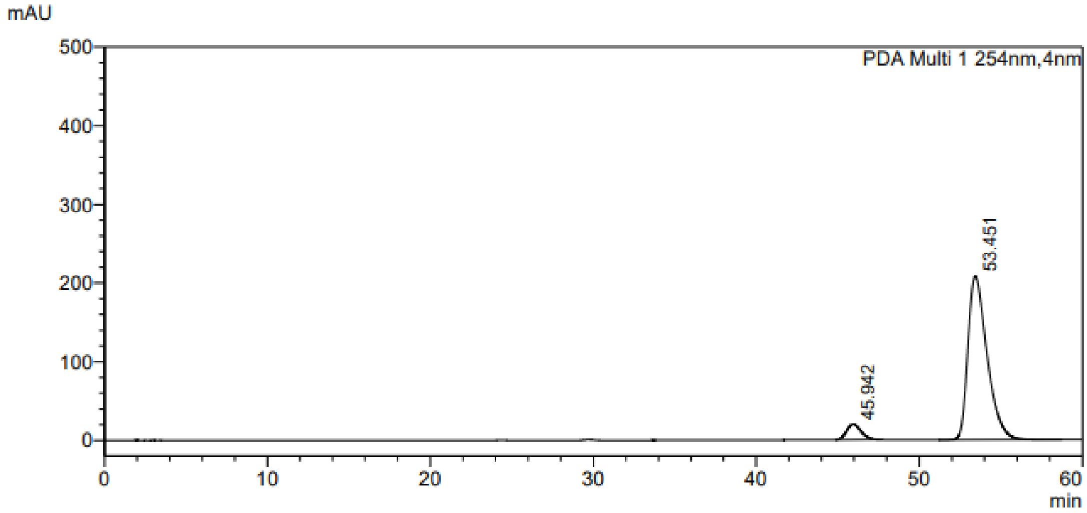

<Peak Table>

PDA Ch1 254nm

Peak#	Ret. Time	Area	Area%
1	45.942	1212285	6.659
2	53.451	16992436	93.341
Total		18204721	100.000

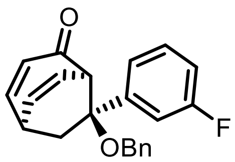

3j

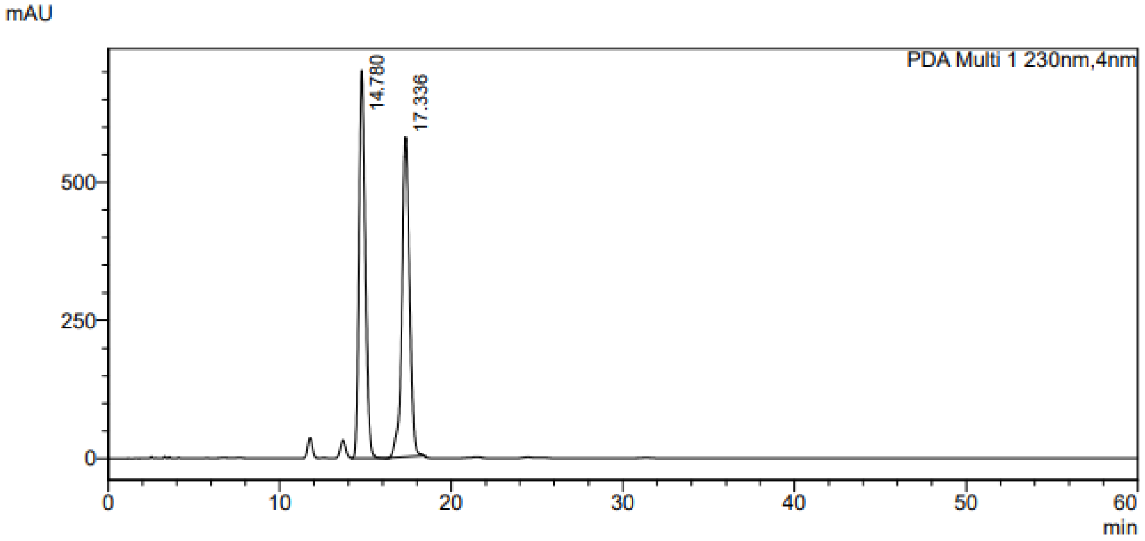

<Peak Table>

PDA Ch1 230nm

Peak#	Ret. Time	Area	Area%
1	14.780	17880203	49.703
2	17.336	18093743	50.297
Total		35973946	100.000

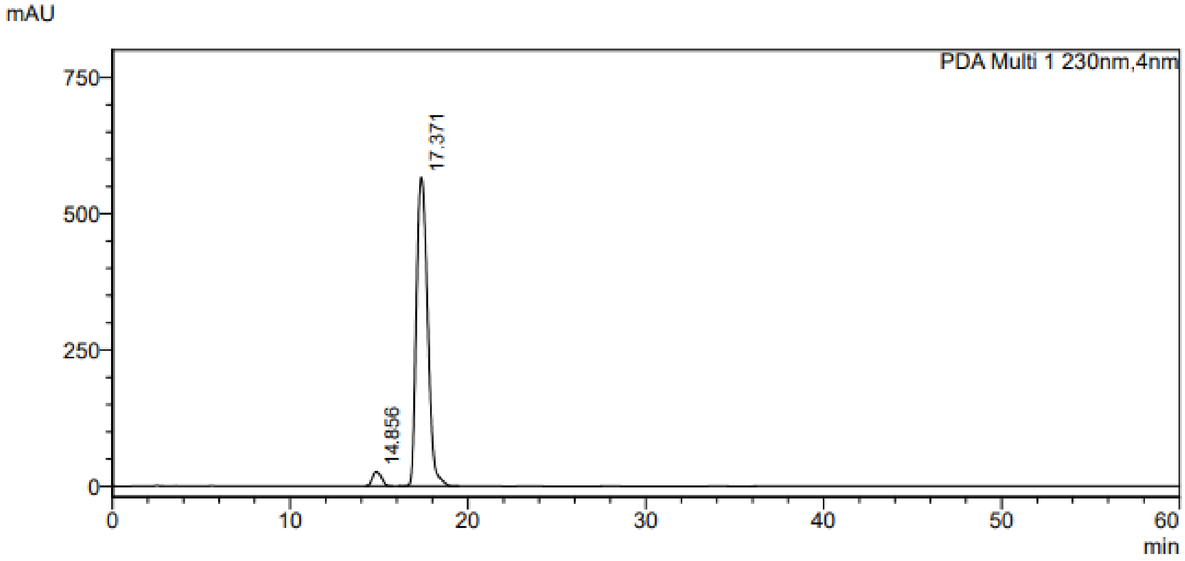

<Peak Table>

PDA Ch1 230nm

Peak#	Ret. Time	Area	Area%
1	14.856	947203	3.669
2	17.371	24869166	96.331
Total		25816369	100.000

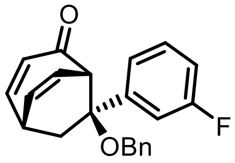

3j-2

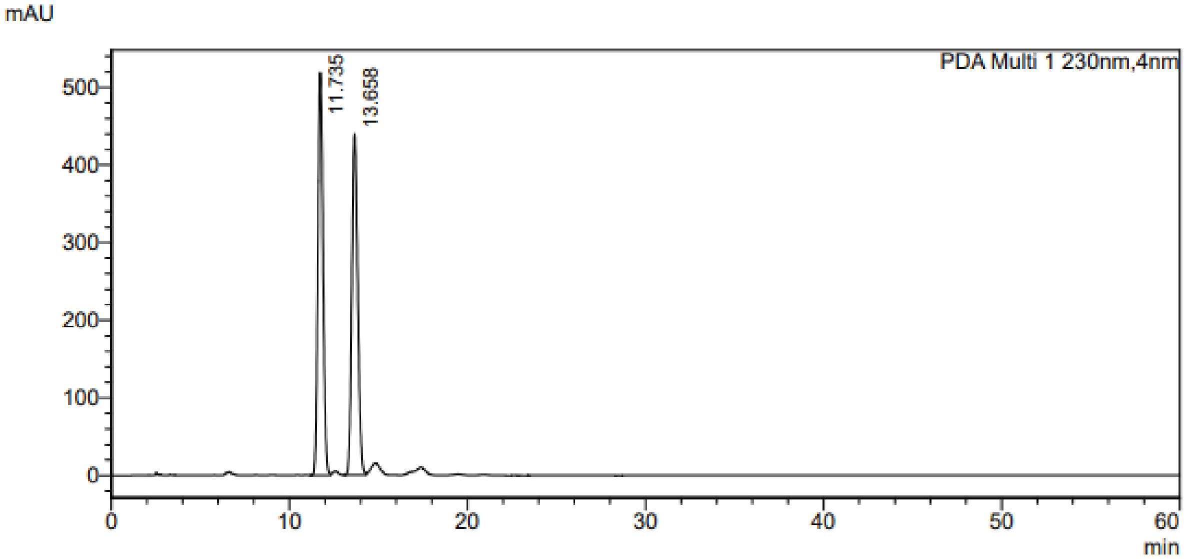

<Peak Table>

PDA Ch1 230nm

Peak#	Ret. Time	Area	Area%
1	11.735	10132487	49.918
2	13.658	10165877	50.082
Total		20298363	100.000

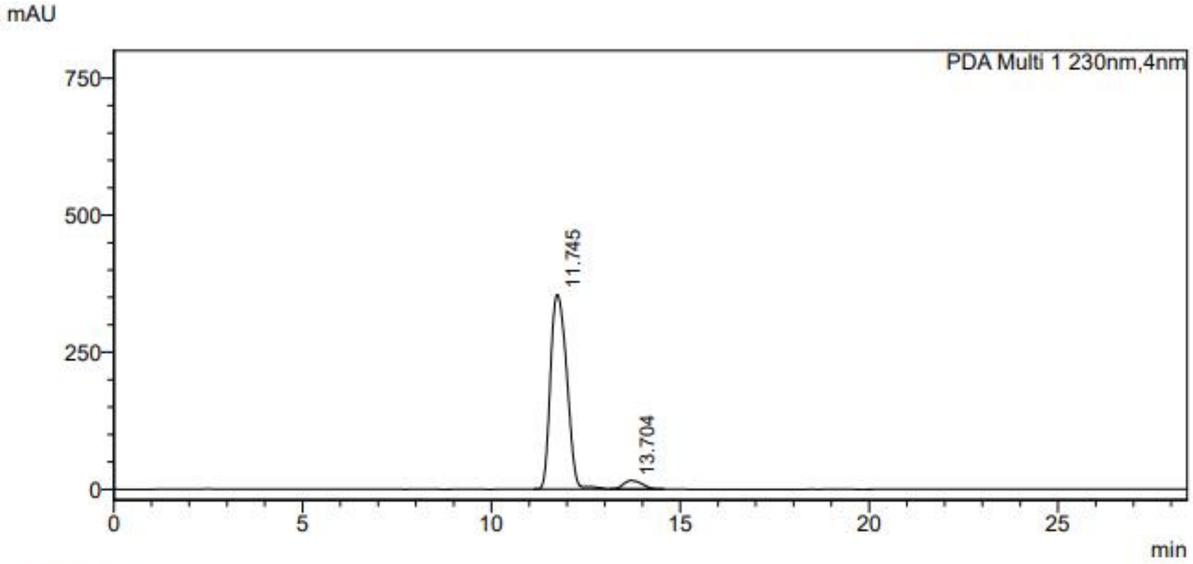

<Peak Table>

PDA Ch1 230nm

Peak#	Ret. Time	Area	Area%
1	11.745	10719068	95.261
2	13.704	533304	4.739
Total		11252372	100.000

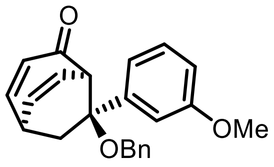

3k

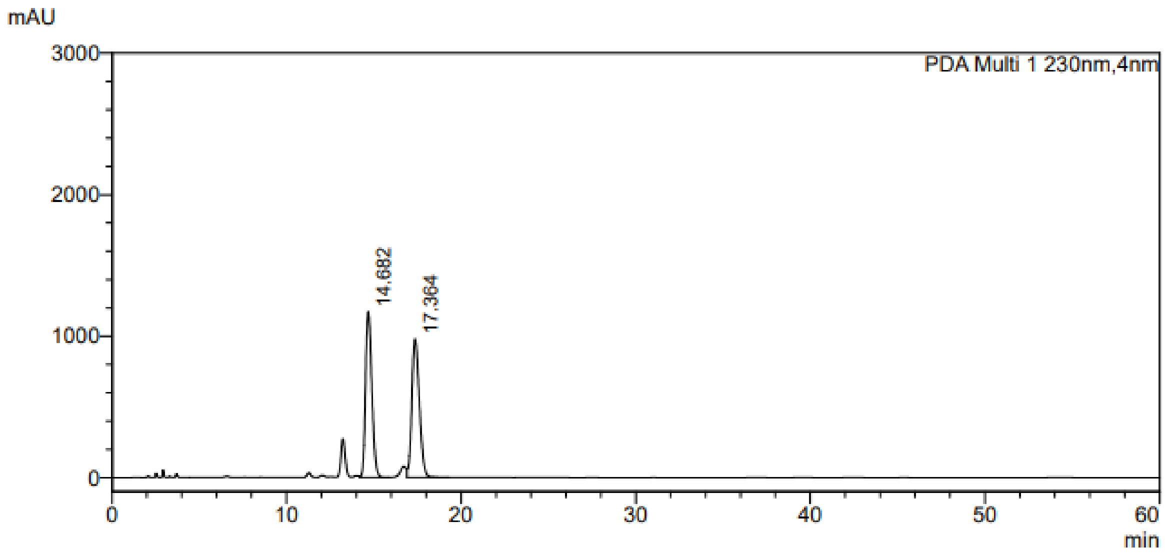

<Peak Table>

PDA Ch1 230nm

Peak#	Ret. Time	Area	Area%
1	14.682	29487668	49.871
2	17.364	29640010	50.129
Total		59127678	100.000

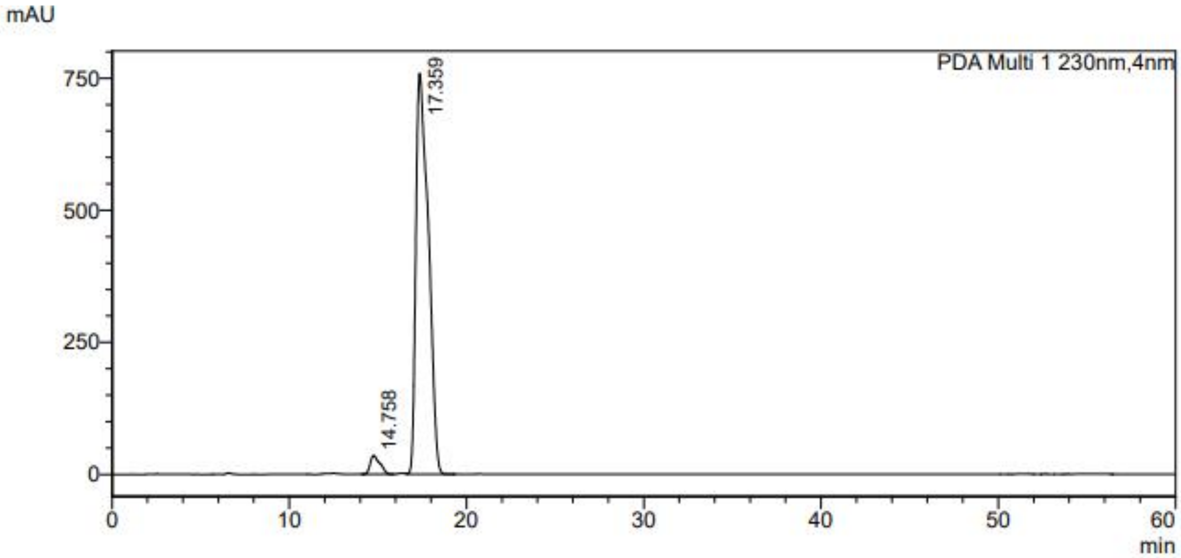

<Peak Table>

PDA Ch1 230nm

Peak#	Ret. Time	Area	Area%
1	14.758	1361526	3.537
2	17.359	37128482	96.463
Total		38490008	100.000

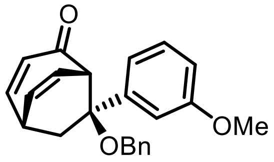

3k-2

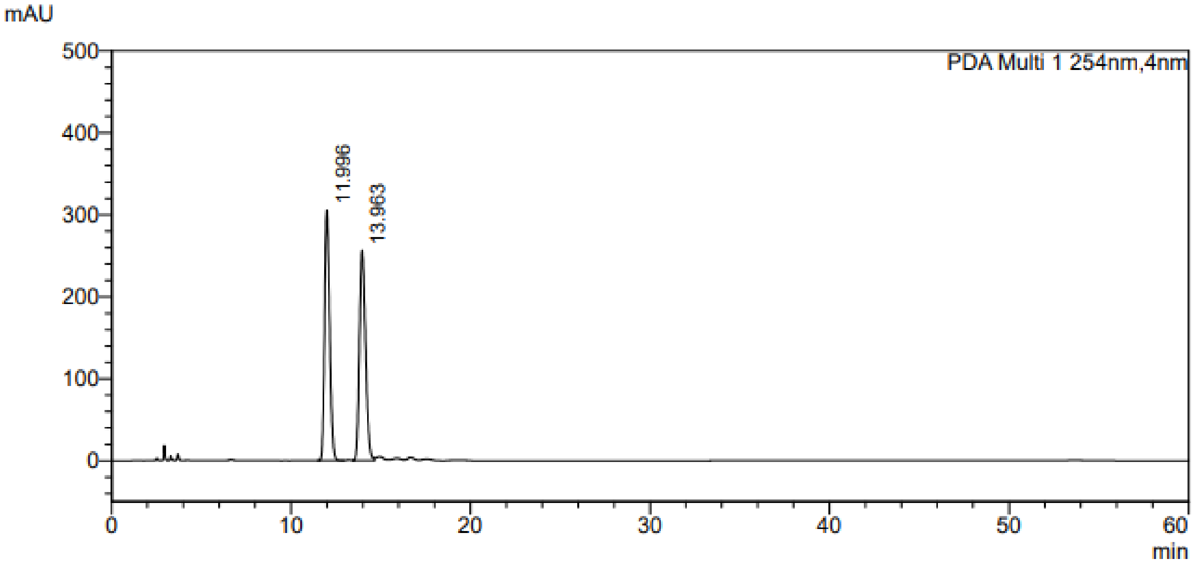

<Peak Table>

PDA Ch1 254nm

Peak#	Ret. Time	Area	Area%
1	11.996	6088342	49.780
2	13.963	6142036	50.220
Total		12230377	100.000

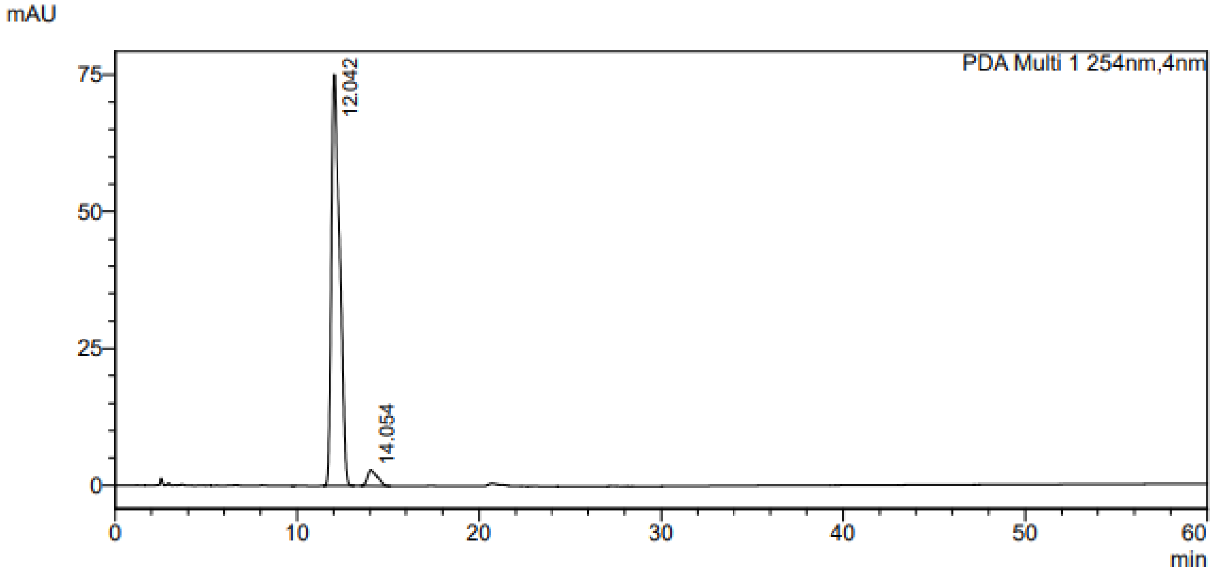

<Peak Table>

PDA Ch1 254nm

Peak#	Ret. Time	Area	Area%
1	12.042	2411869	95.701
2	14.054	108341	4.299
Total		2520210	100.000

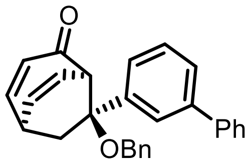

3l

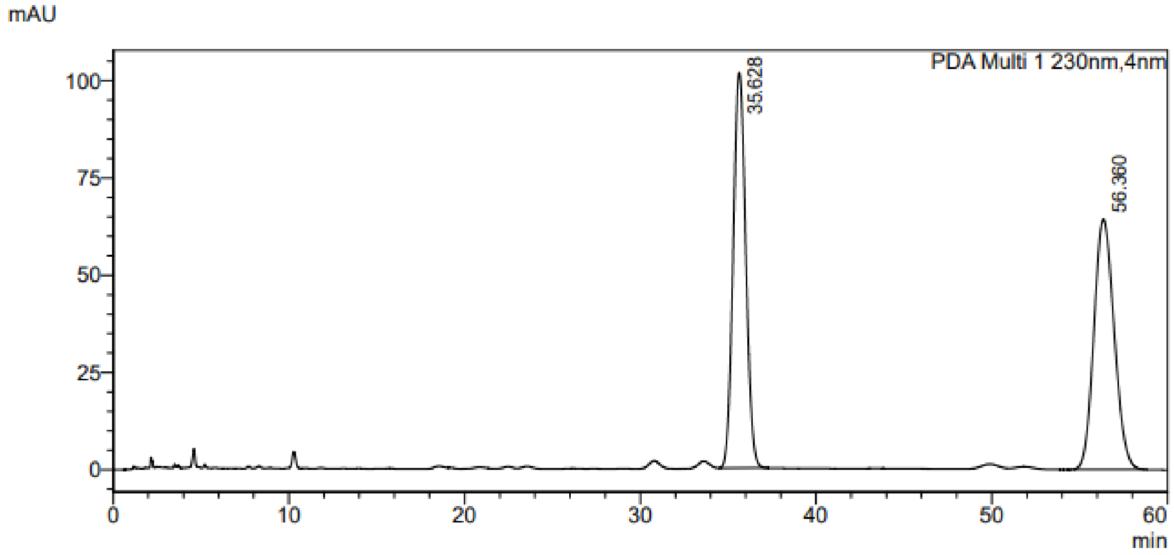

<Peak Table>

PDA Ch1 230nm

Peak#	Ret. Time	Area	Area%
1	35.628	5060318	49.577
2	56.360	5146730	50.423
Total		10207048	100.000

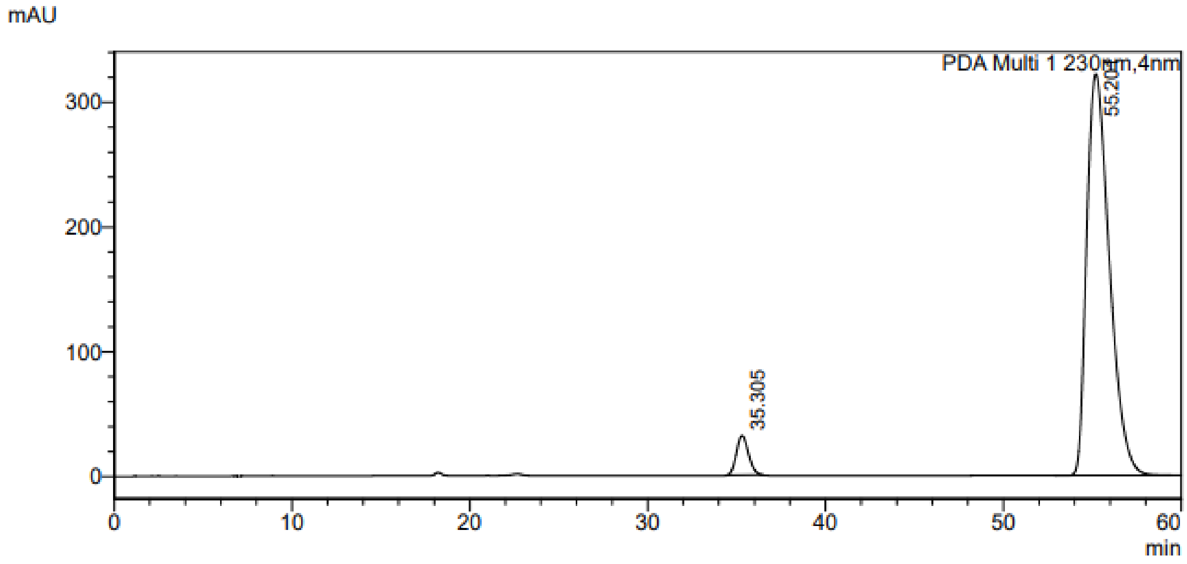

<Peak Table>

PDA Ch1 230nm

Peak#	Ret. Time	Area	Area%
1	35.305	1476678	4.919
2	55.201	28541723	95.081
Total		30018401	100.000

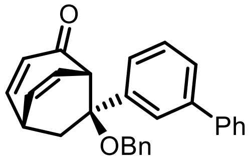

3l-2

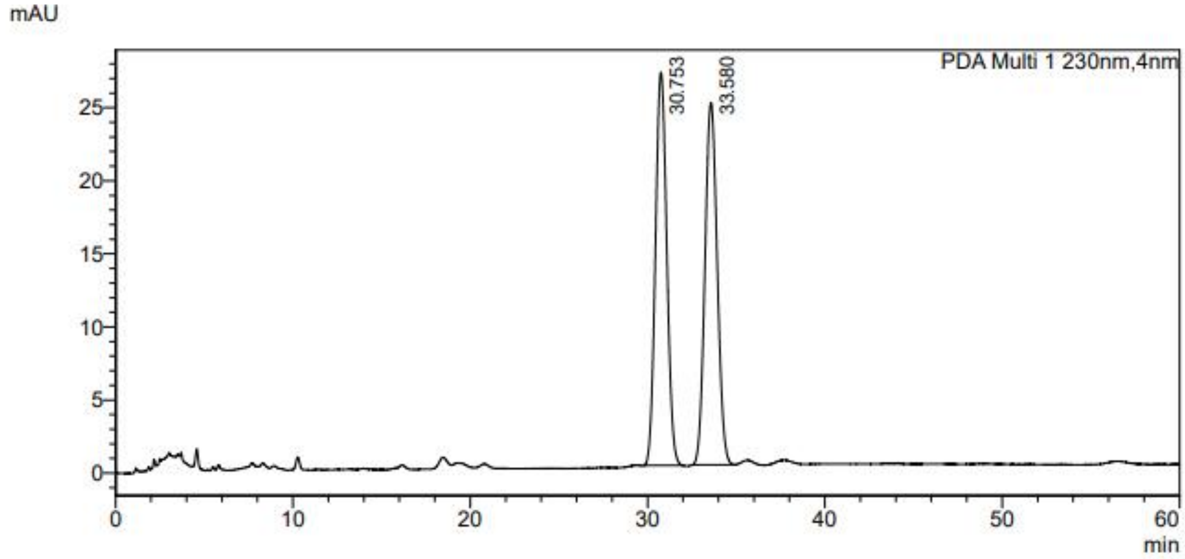

<Peak Table>

PDA Ch1 230nm

Peak#	Ret. Time	Area	Area%
1	30.753	1192633	50.068
2	33.580	1189377	49.932
Total		2382010	100.000

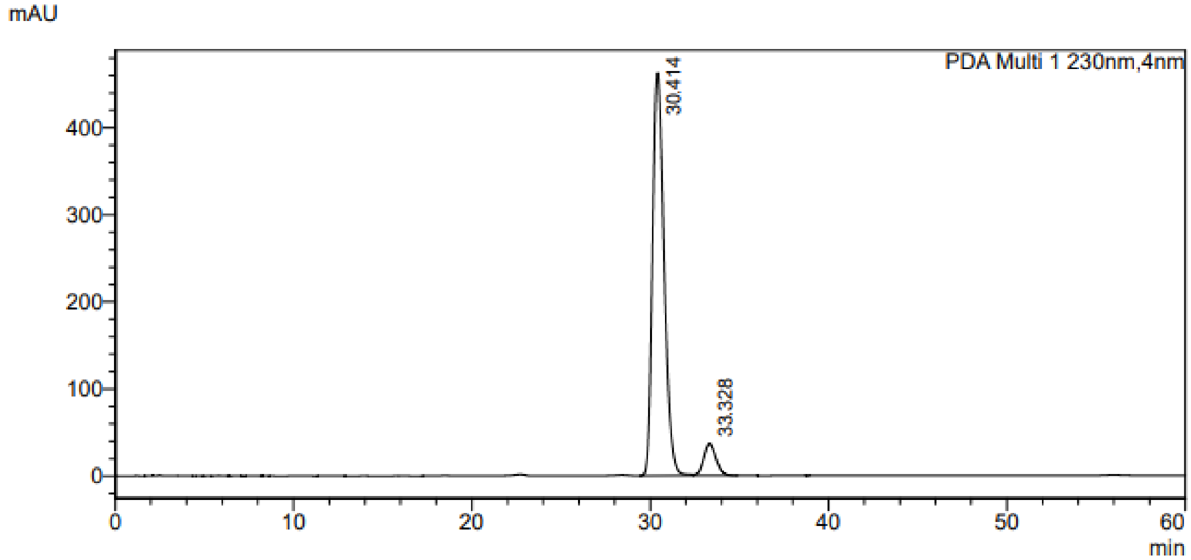

<Peak Table>

PDA Ch1 230nm

Peak#	Ret. Time	Area	Area%
1	30.414	20850440	92.133
2	33.328	1780292	7.867
Total		22630732	100.000

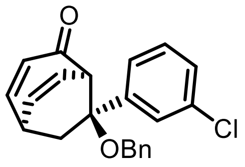

3m

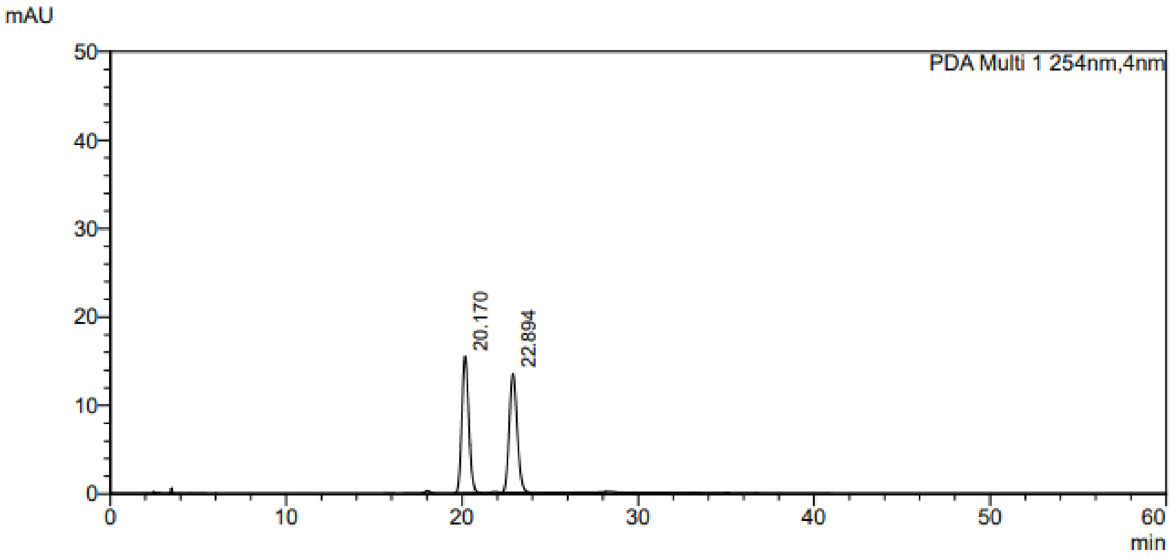

<Peak Table>

PDA Ch1 254nm

Peak#	Ret. Time	Area	Area%
1	20.170	424982	50.296
2	22.894	419985	49.704
Total		844967	100.000

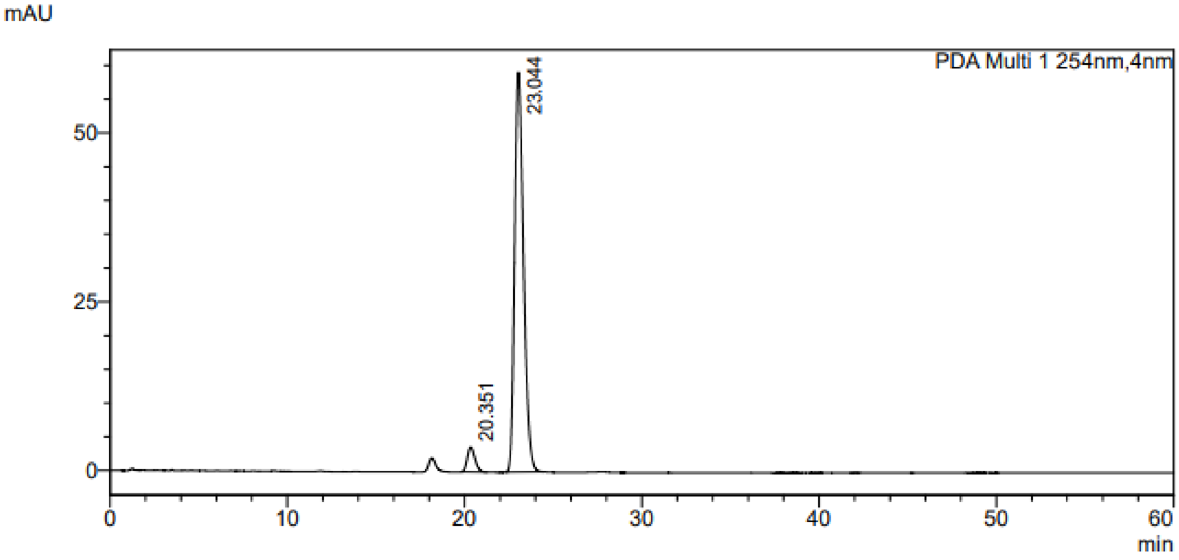

<Peak Table>

PDA Ch1 254nm

Peak#	Ret. Time	Area	Area%
1	20.351	110011	5.006
2	23.044	2087429	94.994
Total		2197440	100.000

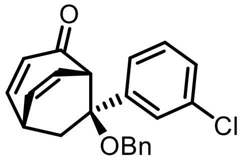

3m-2

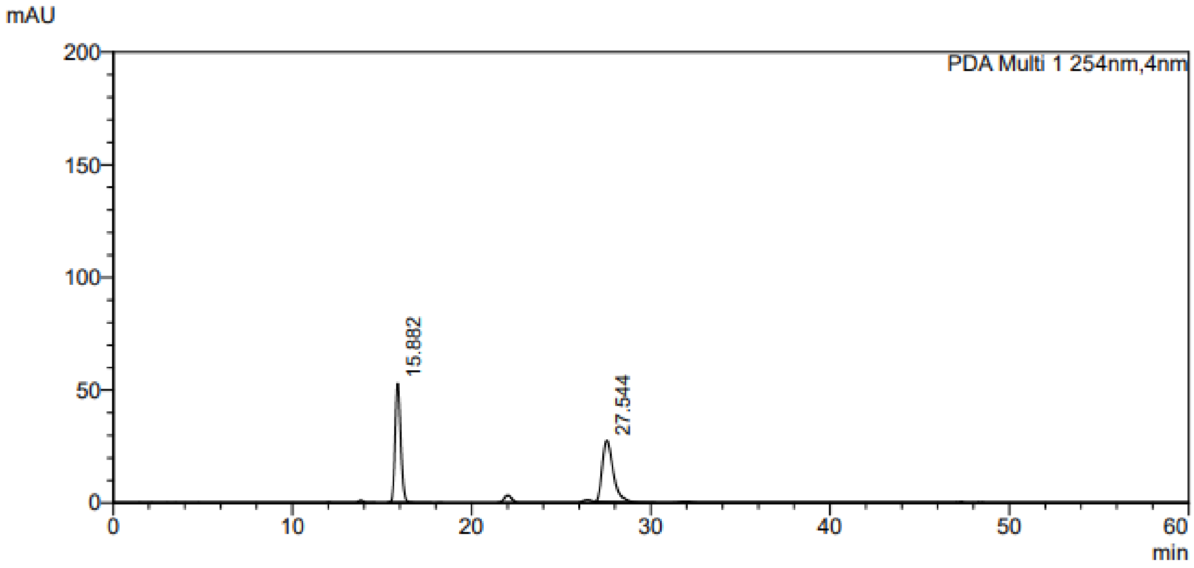

<Peak Table>

PDA Ch1 254nm

Peak#	Ret. Time	Area	Area%
1	15.882	1166222	50.263
2	27.544	1154036	49.737
Total		2320258	100.000

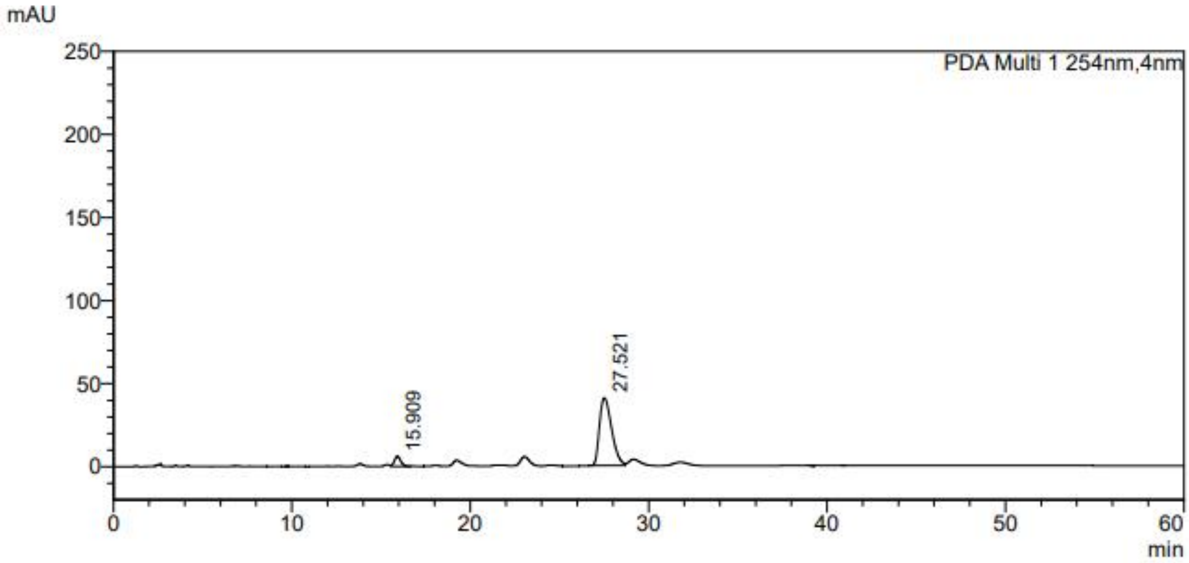

<Peak Table>

PDA Ch1 254nm

Peak#	Ret. Time	Area	Area%
1	15.909	147086	7.048
2	27.521	1939906	92.952
Total		2086991	100.000

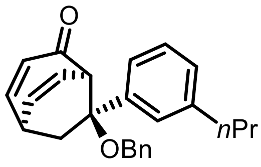

3n

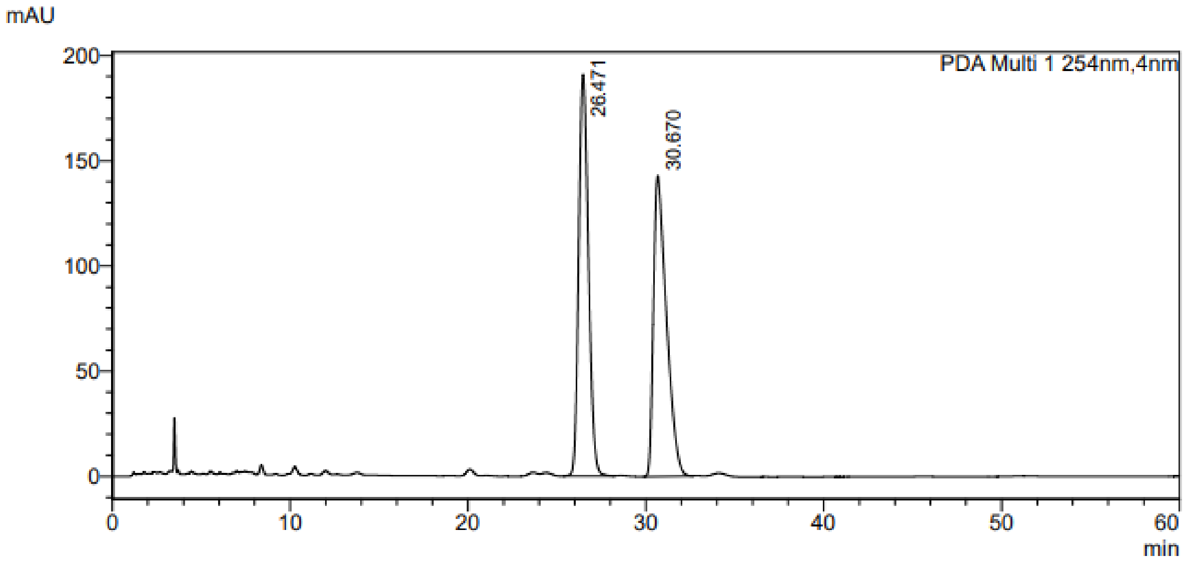

<Peak Table>

PDA Ch1 254nm

Peak#	Ret. Time	Area	Area%
1	26.471	7192703	49.790
2	30.670	7253259	50.210
Total		14445962	100.000

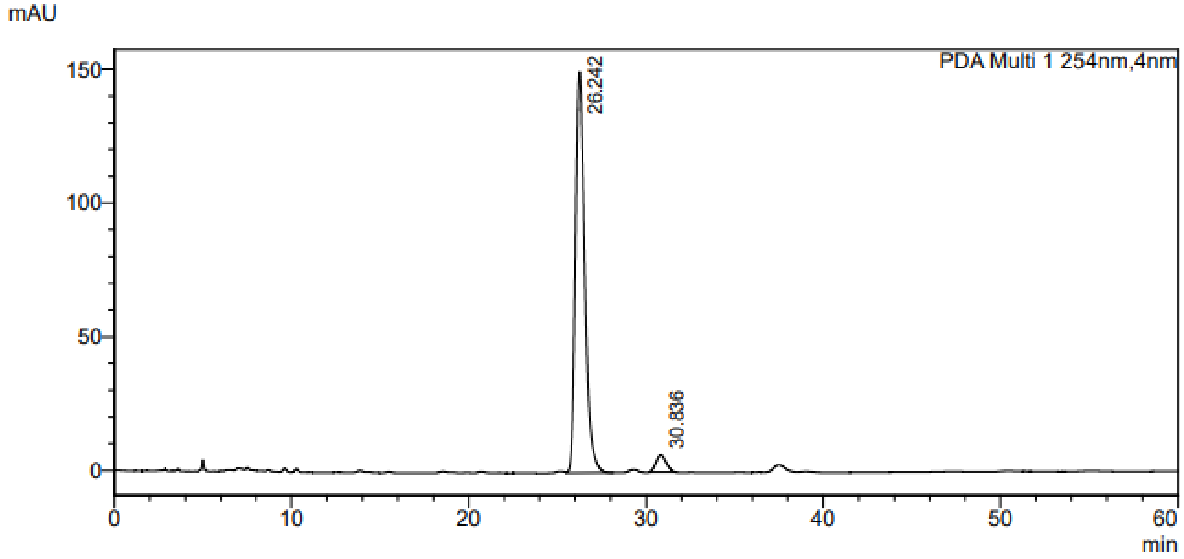

<Peak Table>

PDA Ch1 254nm

Peak#	Ret. Time	Area	Area%
1	26.242	5493022	95.565
2	30.836	254895	4.435
Total		5747917	100.000

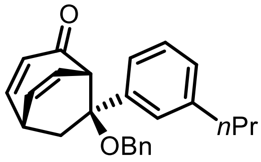

3n-2

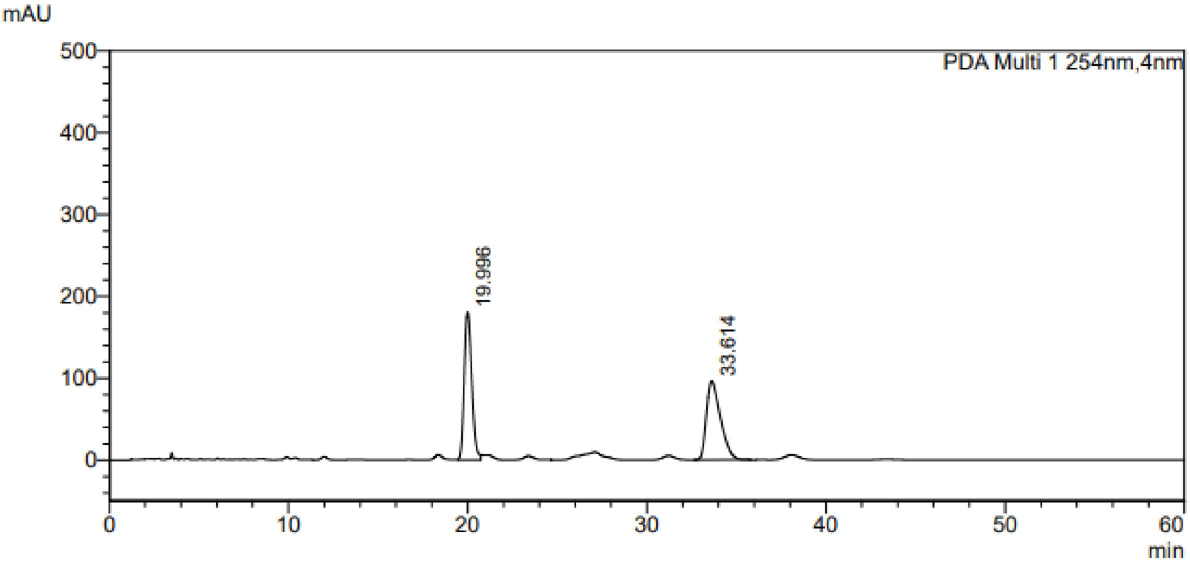

<Peak Table>

PDA Ch1 254nm

Peak#	Ret. Time	Area	Area%
1	19.996	5240565	49.958
2	33.614	5249379	50.042
Total		10489944	100.000

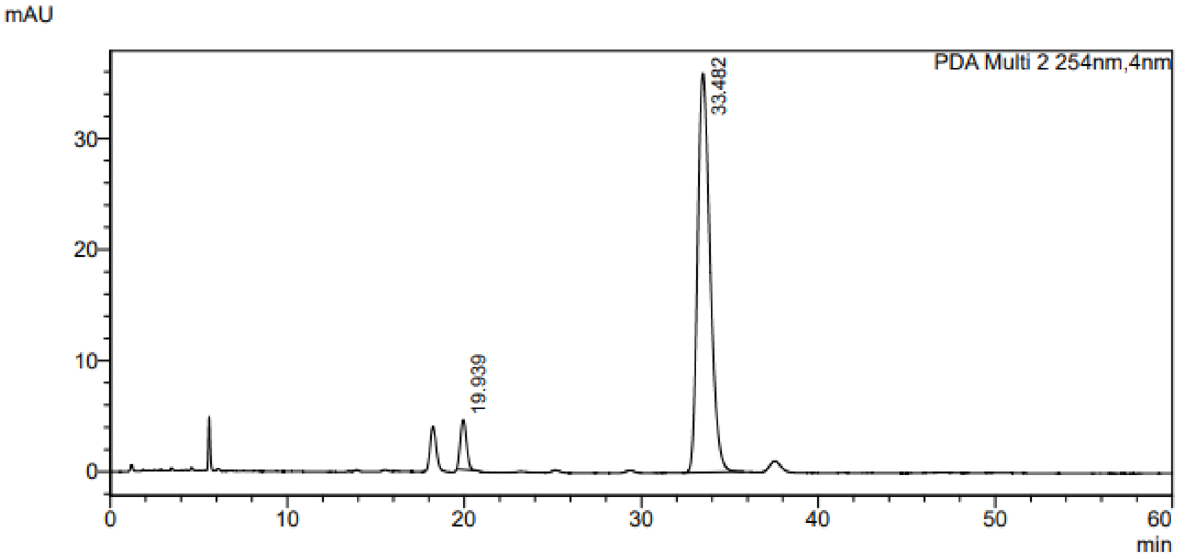

<Peak Table>

PDA Ch2 254nm

Peak#	Ret. Time	Area	Area%
1	19.939	108561	5.807
2	33.482	1760900	94.193
Total		1869461	100.000

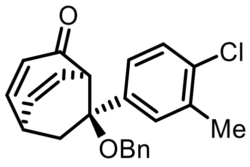

3o

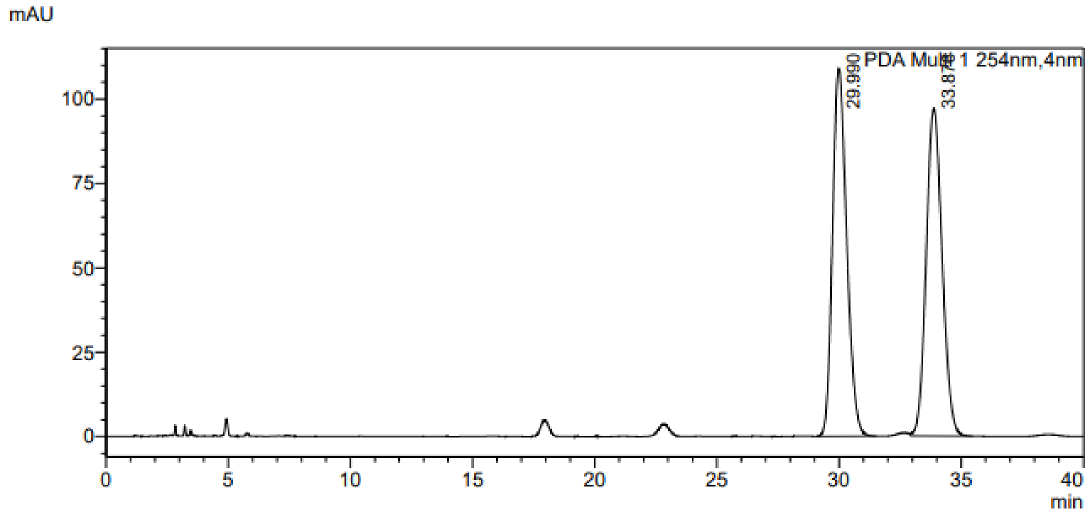

<Peak Table>

PDA Ch1 254nm

Peak#	Ret. Time	Area	Area%
1	29.990	4391912	49.910
2	33.876	4407737	50.090
Total		8799650	100.000

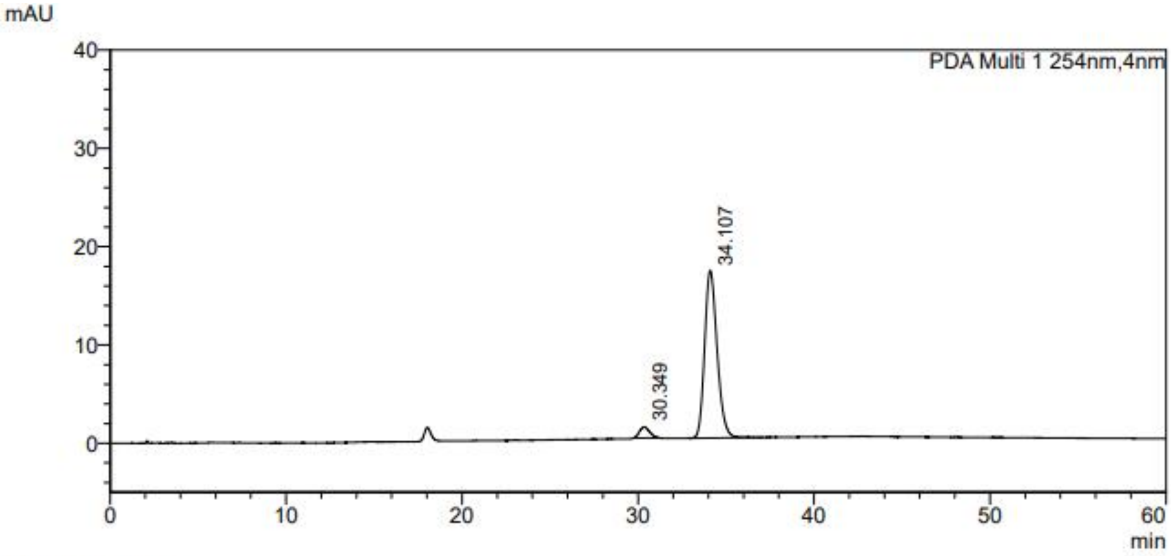

<Peak Table>

PDA Ch1 254nm

Peak#	Ret. Time	Area	Area%
1	30.349	44514	5.050
2	34.107	836946	94.950
Total		881460	100.000

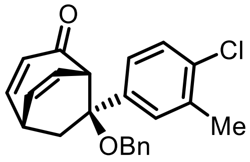

3o-2

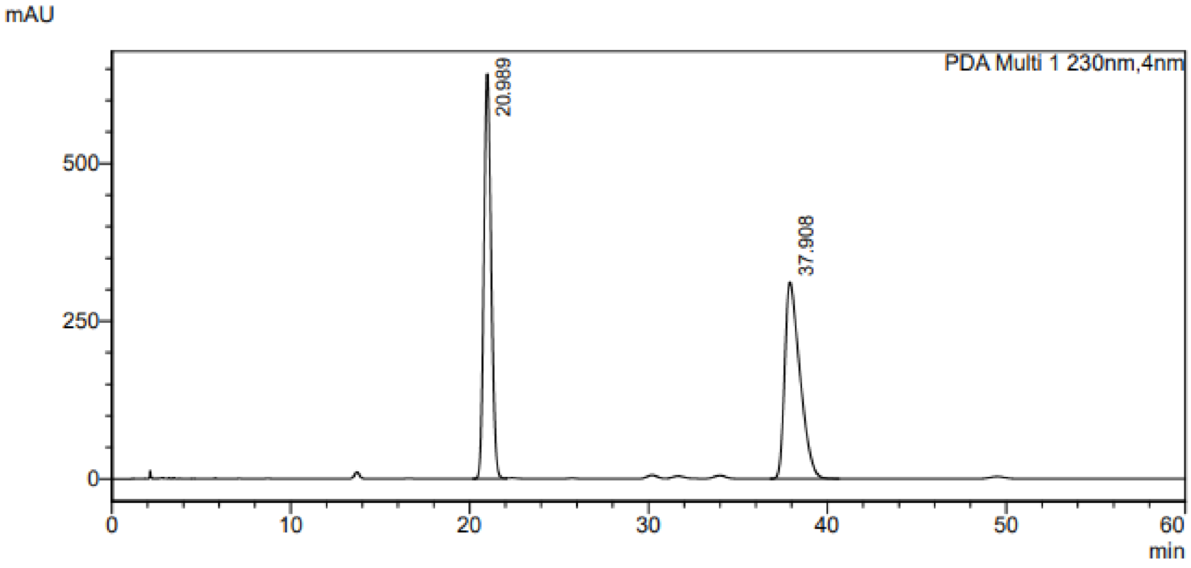

<Peak Table>

PDA Ch1 230nm

Peak#	Ret. Time	Area	Area%
1	20.989	18302203	49.844
2	37.908	18416569	50.156
Total		36718772	100.000

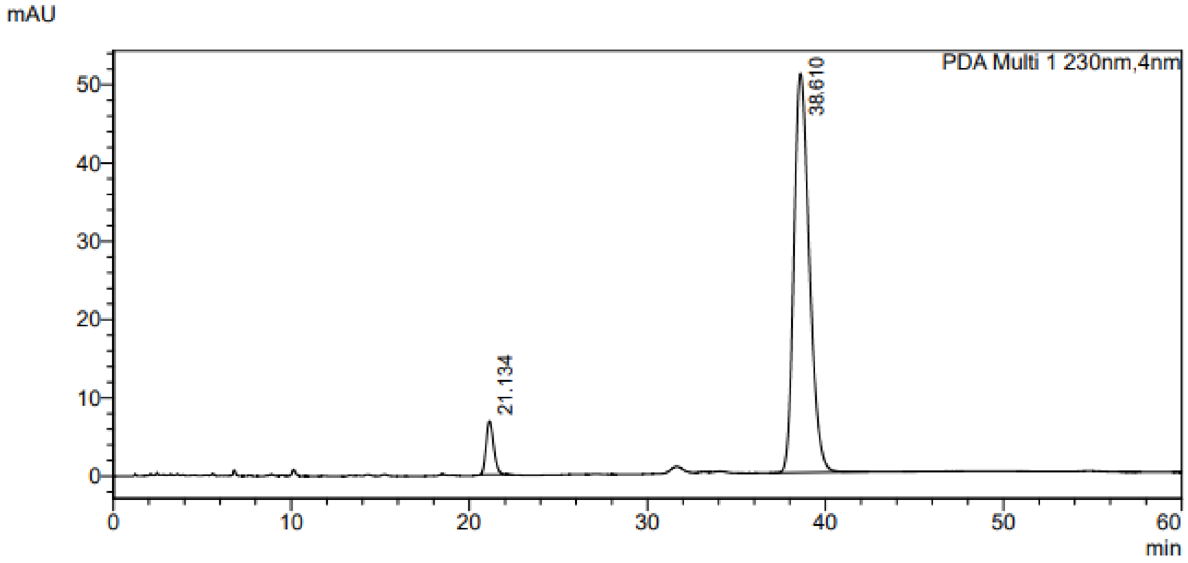

<Peak Table>

PDA Ch1 230nm

Peak#	Ret. Time	Area	Area%
1	21.134	204093	6.218
2	38.610	3078109	93.782
Total		3282202	100.000

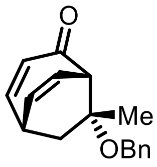

3p-1

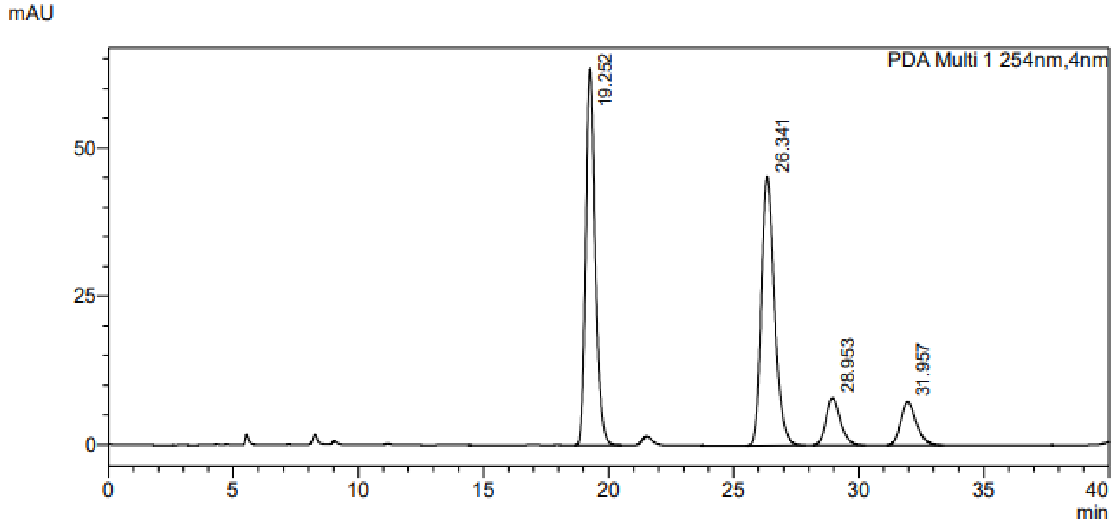

<Peak Table>

PDA Ch1 254nm

Peak#	Ret. Time	Area	Area%
1	19.252	1667360	42.243
2	26.341	1651190	41.833
3	28.953	313350	7.939
4	31.957	315189	7.985
Total		3947090	100.000

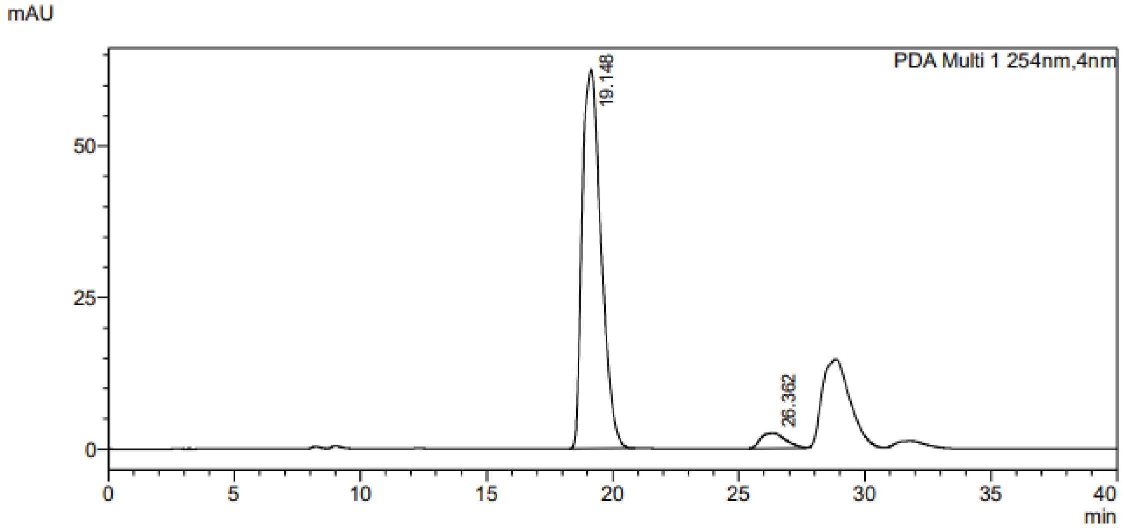

<Peak Table>

PDA Ch1 254nm

Peak#	Ret. Time	Area	Area%
1	19.148	3252716	94.904
2	26.362	174648	5.096
Total		3427364	100.000

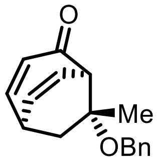

3p-2

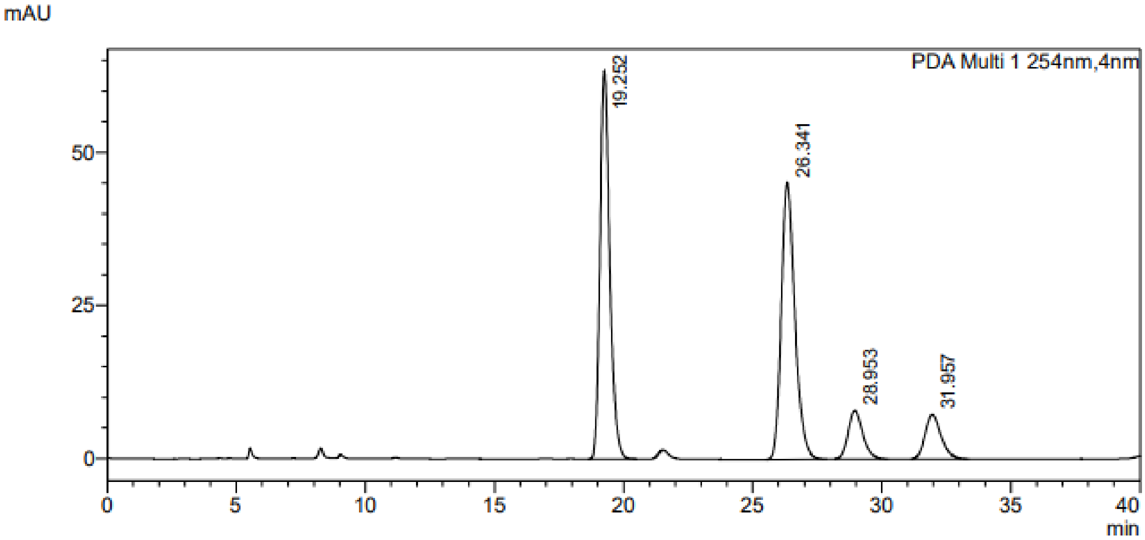

<Peak Table>

PDA Ch1 254nm

Peak#	Ret. Time	Area	Area%
1	19.252	1667360	42.243
2	26.341	1651190	41.833
3	28.953	313350	7.939
4	31.957	315189	7.985
Total		3947090	100.000

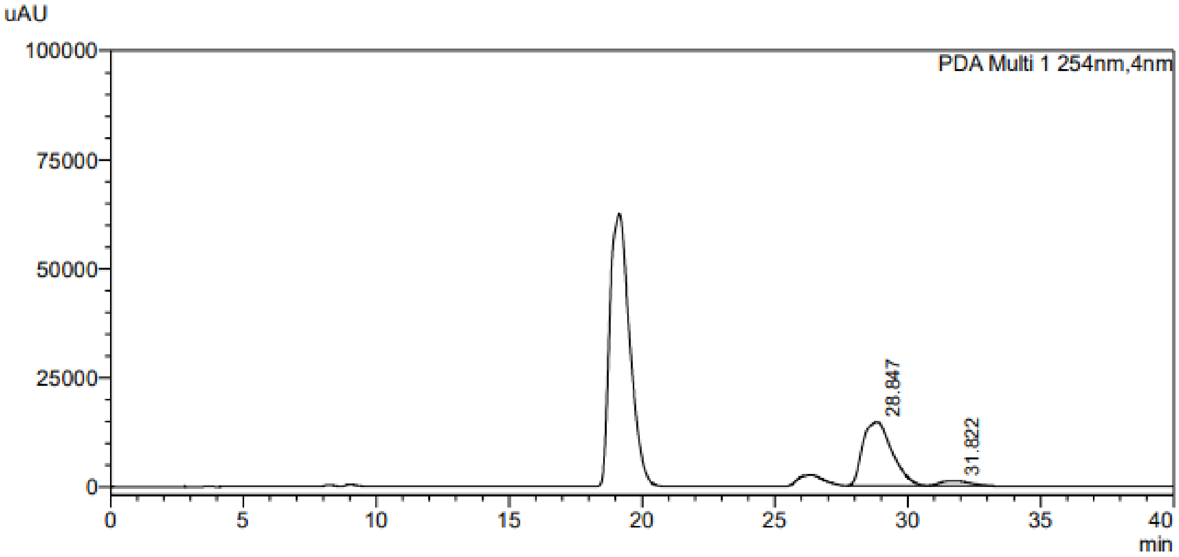

<Peak Table>

PDA Ch1 254nm

Peak#	Ret. Time	Area	Area%
1	28.847	1120298	92.904
2	31.822	85573	7.096
Total		1205871	100.000

17. Computational details

All calculations were performed with a development version of ORCA.[4]

Pre-optimization of free proton catalyzed transition states. To perform a conformational search for the cycloaddition transition states of the catalyzed reactions between **1** and **2a**, we need reasonable guesses of the transition state conformer ensembles of the free proton catalyzed reaction between **1**-H^+ and **2a**. To this end, we first optimized an arbitrarily chosen conformer for each of the transition states of the **1**-H^+ + **2a** reaction that correspond to all possible diastereoselectivities and regioselectivities (**3a-1**, **3a-2**, **3a-3** and **3a-4**; the corresponding proton catalyzed transition states are hereafter termed **TS-3a-1**-H^+ to **TS-3a-4**-H^+, respectively), at the PBE[5]-D3[6]/def2-SVP[7] level of theory. The RI approximation[8] was used to speed up the calculations, with def2/J[9] as the auxiliary basis set. Then, the lengths of the two forming C-C bonds, as well as the C=C double bond dihedral angle of the enol ether, were fixed, and the remaining internal coordinates of the transition states were sampled by the GOAT-ENTROPY keyword of ORCA, at the GFN2-xTB[10] level of theory. The GOAT algorithm generates conformers of a molecule by repeatedly perturbing the internal coordinates of the molecule, and the default convergence criteria were set such that GOAT recovers more conformers than existing tools like CREST[11] (though at a higher cost). As the energetically optimal conformer of the GOAT calculation was in all cases not the conformer that we started with, we used the optimal conformer as the initial guess structure of the DFT transition state optimization, and optimized the structures again. This procedure was repeated until the optimal conformer did not change. Finally, the conformers of **TS-3a-1**-H^+ to **TS-3a-4**-H^+ were mirror inverted to give the transition states leading to the enantiomers of **3a-1** to **3a-4**.

Refitting of the GFN2-xTB method for IDPi systems.
As mentioned in the manuscript, GFN2-xTB is qualitatively wrong for the catalyzed transition states studied herein, such that many important conformers are missed. Unfortunately, we could not afford to use more accurate methods for conformational search. Therefore, we refitted GFN2-xTB against PBE-D3/def2-SVP energies and gradients, so that we can use the refitted GFN2-xTB method in subsequent conformational search. Detailed procedure is given below.

Step 1: Generating conformers of IDPi-4$^-$. Representative conformers of **IDPi-4**$^-$ were generated by the GOAT-ENTROPY keyword, at the GFN2-xTB level of theory.

Step 2: Generating conformers of the catalyzed transition states. The transition state conformers of the proton-catalyzed reaction, including all 8 possible regioisomers, diastereomers and enantiomers, were docked into the catalyst anion conformers generated in Step 1. Of all possible combinations of **IDPi-4**$^-$ conformers and proton-catalyzed transition state conformers, we randomly selected 1/10 for docking at the GFN-FF[12] level, using the DOCKER module of ORCA. Contrary to the usual convention where the catalyst is viewed as the host and the substrate is viewed as the guest, here we treated the proton-catalyzed transition state as the host, and the catalyst anion as guest, as only by this way does the program allow us to constrain the aforementioned three important internal coordinates of the proton-catalyzed transition state (the lengths of the two forming C-C bonds, and the C=C double bond dihedral angle of the enol ether) while docking. Furthermore, the following keywords were added to the %docker block of the input file:

docklevel screening

noopt true
evoptlevel sponly

This resulted in a crude but very fast docking, where only one docked structure was generated for each selected combination of the host and guest conformers, and the structures of the host and guest were not allowed to relax during docking.

Step 3: Diversifying the structures. The aforementioned docking procedure only generates structures that are close to the potential energy local minima at the GFN-FF level of theory. To obtain a more diverse set of structures, we performed a 5 ps GFN2-xTB molecular dynamics (MD) run from each structure generated by docking, using a large time step (1 fs). Harmonic restraints were added to the two forming C-C bonds, with spring constants 10000 kJ/mol/Å^2 and 1000 kJ/mol/Å^2 for the shorter and longer C-C bond, respectively; this ensures that the transition state does not collapse into the reactant or product while sampling the potential energy surface around the transition state. An elastic, spherical cell with initial radius 20 Å, external pressure 1 bar, t_{avg} = 100 fs, and $c_{response}$ = 0.001 was added to prevent the tropylium and/or the enol ether from dissociating from the IDPi cavity. The temperature of the simulation was controlled by a Nosé-Hoover chain (NHC) thermostat, with temperature 253 K (consistent with the experimental temperature) and time constant 100 fs.

Step 4: Generating reference energy and gradient data. PBE-D3/def2-SVP energy and gradient were calculated for the last frame of each MD trajectory.

Step 5: Fitting the GFN2-xTB parameters against DFT data. The following cost function was minimized using the simplex algorithm:

$$\text{cost} = \sum_{i}^{N_{\text{struct}}} \left(E_i^{\text{xTB}} - \bar{E}^{\text{xTB}} - E_i^{\text{DFT}} + \bar{E}^{\text{DFT}}\right)^2 + 0.1 \sum_{i}^{N_{\text{struct}}} \sum_{j}^{3N_{\text{atom}}} \left(g_{ij}^{\text{xTB}} - g_{ij}^{\text{DFT}}\right)^2 + 0.1 \sum_{k}^{N_{\text{param}}} \left(\frac{P_k^{\text{new}}}{P_k^{\text{orig}}} - 1\right)^2 \quad (1)$$

where:

- N_{struct} is the number of structures in the training set.
- E_i^{xTB} is the GFN2-xTB energy of the i-th structure (unit: Hartree).
- $\bar{E}^{\text{xTB}}$ is the average GFN2-xTB energy of all structures (unit: Hartree).
- E_i^{DFT} is the PBE-D3/def2-SVP energy of the i-th structure (unit: Hartree).
- $\bar{E}^{\text{DFT}}$ is the average PBE-D3/def2-SVP energy of all structures (unit: Hartree).
- $N_{\text{atom}} = 241$ is the number of atoms of the catalyzed transition state.
- g_{ij}^{xTB} is the j-th component of the GFN2-xTB gradient of the i-th structure (unit: Hartree/Bohr).
- g_{ij}^{DFT} is the j-th component of the PBE-D3/def2-SVP gradient of the i-th structure (unit: Hartree/Bohr).
- $N_{\text{param}} = 13$ is the total number of semiempirical parameters to be refitted.
- P_k^{orig} is the original value of the k-th semiempirical parameter, as given by the unmodified GFN2-xTB method.
- P_k^{new} is the new value of the k-th semiempirical parameter, as given by the fitting process.

The three terms on the right hand side of Eq. (1) thus accounts for (a) the difference of the GFN2-xTB and DFT energies on the training structures, (b) the difference of the GFN2-xTB and DFT gradients on the training structures, and (c) the difference of the modified GFN2-xTB parameters with respect to their original values. Minimizing the sum of the three terms therefore makes GFN2-xTB reproduce the DFT energies and gradients as closely as possible,

while ensuring that the re-fitted parameters do not acquire unphysical values (i.e. values that are very different from the original GFN2-xTB method).

The parameters that need to be refitted are chosen by the following procedure. Firstly, visual inspection of the GFN2-xTB and DFT gradients suggest that the largest gradient errors occur along the directions of C-S, S-O and P-O bonds. We thus chose the off-diagonal H_0 matrix element scale factors of the three element pairs C-S, S-O and P-O as the first three parameters to be refitted. Secondly, some one-center parameters of phosphorus and sulfur were also included in the refitting to correct the remaining deficiencies of GFN2-xTB for the current system. Empirical tests suggest that only the following parameters of P and S (5 parameters per element) differ substantially from their original values: (1) the 3p and 3d atomic orbital energy levels (H^l); (2) the 3d orbital exponents (ζ^l); and (3) the scaling factor of the Hubbard parameter for the 3p and 3d shells (κ_A^l). Therefore, in the final fit we only allowed these 10 one-center parameters, plus the aforementioned 3 two-center parameters, to change, while holding all other GFN2-xTB parameters fixed at their original values.

As the original (unmodified) GFN2-xTB method exhibits qualitative deficiencies even for the conformational sampling of **IDPi-4**$^-$, we had to perform the re-fitting process twice, so that in the second round of re-fitting we used the GFN2-xTB parameters from the first round to generate the conformers of **IDPi-4**$^-$. This ensures that the structures in the training set are representative of the actual conformer ensemble. The number of training structures (N_{struct}) generated in the first and second rounds are 678 and 895, respectively.

The refitted GFN2-xTB parameters in the first and second rounds are shown in Table S9, together with their original values. Note that some of the parameters (notably the H^l (3d) and κ_A^l (3p) parameters of both phosphorus and sulfur) changed considerably from Round 1 to Round 2, while some others (e.g. ζ^l (3d)) were essentially unchanged by the second round of fitting. This suggests that while the first round of fitting already revealed the need for revising some of the parameters, the second round of fitting is necessary for getting the correct values of these parameters.

Table S9. Original and refitted GFN2-xTB parameters. Unless otherwise noted, all calculations with "the refitted GFN2-xTB method" refer to calculations using the "Round 2" parameters.

Element	Parameter name	Original value	Round 1	Round 2
C-S	H_0 scale factor	1.0	1.0372371316242417	1.0613856503407593
S-O	H_0 scale factor	1.0	1.0002352194965995	1.0211273075748355
P-O	H_0 scale factor	1.0	0.9542074802544871	0.9850265624097557
P	H^l (3p)	-9.842286	-10.838989018119115	-10.227469409058207
P	H^l (3d)	-0.444893	-0.4299392522148764	-0.5550378543981546
P	ζ^l (3d)	1.167533	0.9945054931099073	0.9754691913976866
P	κ_A^l (3p)	-1.558060	-1.6618421087776136	-1.7226602294358062
P	κ_A^l (3d)	-3.500000	-3.339972830738091	-2.9996088330542685
S	H^l (3p)	-11.377694	-18.02007843328787	-17.489097958114137
S	H^l (3d)	-0.420282	-0.4883931656438429	-0.7885011956621273
S	ζ^l (3d)	1.702555	1.0763806231714113	1.0548000979170993
S	κ_A^l (3p)	-1.085866	-1.033586846093704	-0.32011107729590016
S	κ_A^l (3d)	-2.500000	-2.938111001691505	-2.413302338124578

We believe that this set of parameters is probably suitable for studying other IDPi catalysts as well (provided that the substrates do not contain phosphorus and sulfur), although benchmark tests are recommended before using these parameters in other molecular systems, even systems that are very similar to those studied herein.

The current parameters can be used in ORCA via the following procedure:

- copy the file "param_gfn2-xtb.txt" under the share/xtb folder of the xtb program package to the folder where the ORCA input file is situated, and rename it to "param.txt";
- add the following lines in "param.txt" after the first occurrence of "$end":

```
$pairpar
6 16 1.0613856503407593
8 16 1.0211273075748355
8 15 0.9850265624097557
$end
```

- replace the lines $Z=15 … $end by

```
$Z=15 Mon Apr 23 19:31:07 CEST 2018
ao=3s3p3d
lev=    -17.518756      -10.227469409058207      -0.5550378543981546
exp=        1.816945        1.903247        0.9754691913976866
GAM=        0.297739
GAM3=        0.711291
KCNS=        0.547610
KCNP=      -0.489930
KCND=        2.429507
DPOL=        2.110225
QPOL=        0.028679
REPA=        1.143343
REPB=      19.683502
POLYS=    -19.831771
POLYP=      -5.515577
POLYD=      26.397535
LPARP=      -1.7226602294358062
LPARD=      -2.9996088330542685
$end
```

- replace the lines $Z=16 … $end by

```
$Z=16 Tue Apr 24 11:44:34 CEST 2018
ao=3s3p3d
lev=    -20.029654    -17.489097958114137      -0.7885011956621273
exp=        1.981333        2.025643        1.0548000979170993
GAM=        0.339971
```

GAM3= -0.501722
KCNS= -0.256951
KCNP= -0.098465
KCND= 2.007690
DPOL= -0.151117
QPOL= 0.442859
REPA= 1.214553
REPB= 14.995090
POLYS= -25.855520
POLYP= -8.048064
POLYD= 25.993857
LPARP= -0.32011107729590016
LPARD= -2.413302338124578
$end

- add the following line to the ORCA input file that runs GFN2-xTB:

  %xtb xtbinputstring "--vparam param.txt" end

The improvements of the refitted GFN2-xTB method compared to the original method can already be seen from the conformer ensemble of **IDPi-4**⁻. The original GFN2-xTB method predicts that the lowest conformer has two similar substrate pockets, each being sandwiched between a 2,3,5,6-tetrafluoro-4-trifluoromethylphenyl group and a naphthalene moiety (Figure S18). By contrast, the refitted GFN2-xTB method from the first round of fitting already gives a lowest energy conformer that qualitatively reproduce the DFT conformer, with a clearly identifiable "fluorous pocket" and a "non-fluorous pocket". This behavior remains unchanged when using the parameters from the second round of fitting, which also suggests that two rounds of fitting is enough, and it is probably not necessary to carry out a third round of fitting.

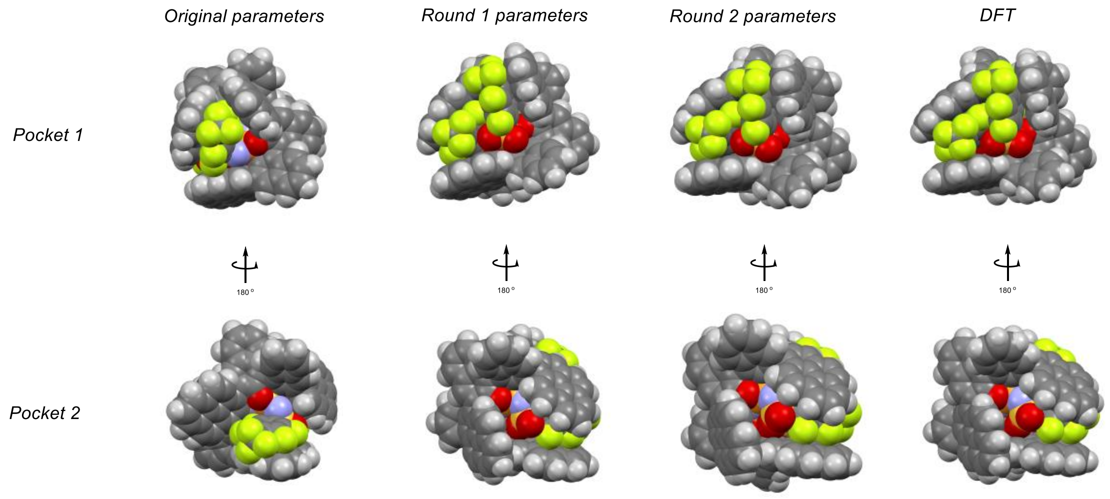

Figure S18. The lowest energy conformer of **IDPi-4**⁻ generated by GFN2-xTB using the original parameters, "Round 1" parameters, and "Round 2" parameters. The lowest energy conformer at the PBE-D3/def2-SVP level is shown for comparison.

One can obtain deeper insight from the energy distribution of the conformers (Figure S19). The original GFN2-xTB method generates a dense manifold of low energy conformers, with 6 conformers within 2 kcal/mol of the lowest conformer, and no clear energy gap between the lowest and second-lowest energy conformers. However, both the “Round 1” and “Round 2” parameters give a single lowest energy conformer that is at least 2 kcal/mol lower than the second lowest energy conformer. The latter observation is more consistent with a highly stereoselective and regioselective catalyst, since different conformers of a catalyst tend to prefer different products, so that a catalyst with many low energy conformers is less likely to display high stereoselectivity and regioselectivity than a catalyst with a well-defined lowest energy conformer.

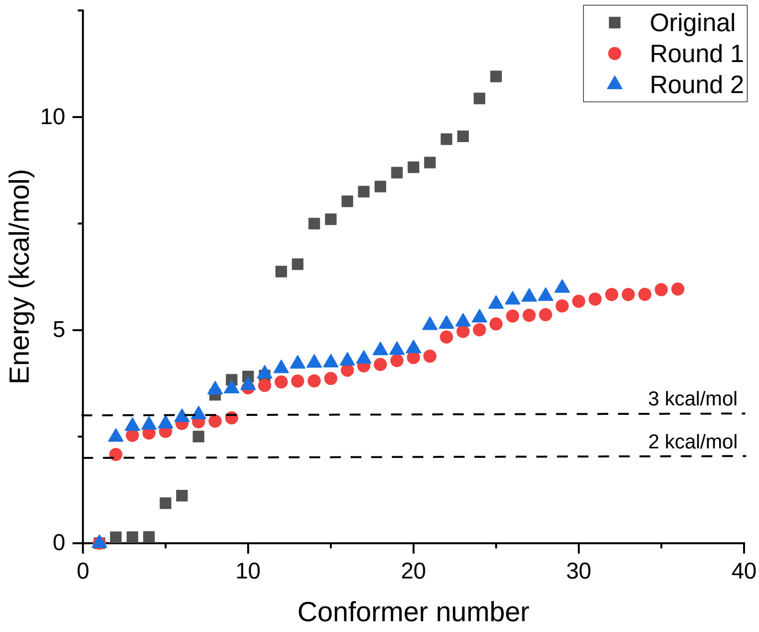

Figure S19. Energies of conformers generated by GFN2-xTB using the original parameters, “Round 1” parameters, and “Round 2” parameters.

For completeness, in addition to the lowest energy conformer obtained using GFN2-xTB with the original parameters (Figure S18), we also give the structures of the other five conformers below 2 kcal/mol (Figure S20). None of these conformers resemble the DFT conformer shown in Figure S18, because in all these conformers, there is only one 2,3,5,6-tetrafluoro-4-trifluoromethylphenyl group in each substrate pocket, while the DFT conformer has one pocket with two such groups and another pocket with no such group. The main difference between these conformers is the relative orientations of pyrene rings with respect to the rest of the molecule.

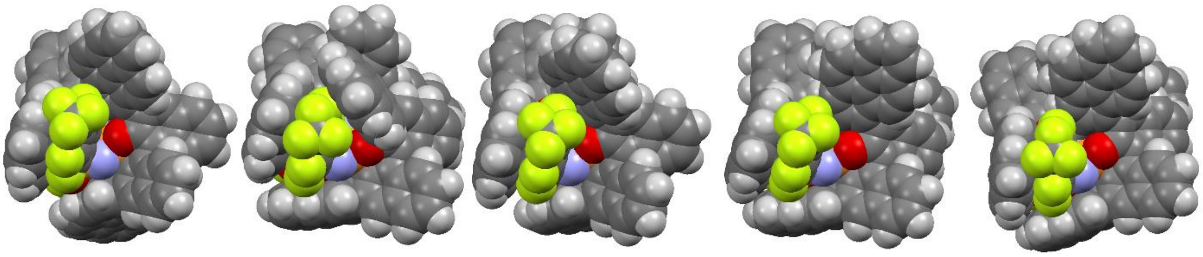

Figure S20. The 2nd to 6th lowest energy conformers of **IDPi-4**$^-$ (ordered from left to right) generated by GFN2-xTB using the original parameters.

Finally, we note that a similar refitting approach has been used by some of us (Z.W. and F.N.)[13] in the molecular dynamics simulation of a rhodium complex (where only two H_0 scale factors have been refitted), with the resulting product distribution agreeing qualitatively with experiment.

Gibbs free energy calculations.

As the correct description of the conformer ensemble is vital for the calculation of $\Delta\Delta G^‡$, we have employed a stepwise filtration process where a large number of reactant and transition state conformers are generated at a low level of theory (GFN2-xTB, refitted), and then progressively refined using successively higher levels of theory. The filtration process is a modification of the procedure used in our previous works.[14-16]

Step 1: Conformer generation. Representative conformers of **IDPi-4** and **IDPi-4**$^-$ were generated by the GOAT-ENTROPY keyword, at the GFN2-xTB (refitted) level of theory.

Although the conformers of the catalyst-substrate complexes as well as the catalyzed transition states can in principle be generated by GOAT as well, in practice this proved to be prohibitively expensive. Thus, the conformers of the complex of **IDPi-4** and **1** (**RC-3**) were generated by docking **IDPi-4** and **1**; the conformers of the same complex but with a proton transferred from **IDPi-4** to **1** (**RC-1**, **RC-2**) were generated by docking **IDPi-4**$^-$ and **1**-H^+; the conformers of the catalyzed transition states (**TS-3a-1**, **TS-3a-1-RRR**, **TS-3a-4**, **TS-3a-4-SRR**) were generated by docking **IDPi-4**$^-$ with **TS-3a-1**-H^+, **TS-3a-4**-H^+ or their enantiomers (where the two forming C-C bonds and the enol ether C=C bond are constrained).

To save computational effort without missing any important conformer, all docking calculations that involved the most stable conformer of **IDPi-4**$^-$ were performed with the keyword "fixhost true" in the "%docker" block of the input file (so that the catalyst's structure is not allowed to relax, but the substrate(s)' structures can relax), and we kept all of the resulting docked structures (about 20 per calculation); while for all other conformers of **IDPi-4**$^-$, we also added the keywords "noopt true" and "evoptlevel sponly" (so that the substrate(s)' structures are also not allowed to relax), and kept only the lowest energy docked structure.

The conformers were subsequently pruned to remove duplicate and high-energy conformers. Conformers whose energies are more than 20 kcal/mol higher than the lowest energy conformer were removed. To accelerate the removal of duplicate conformers, a three-step filtering process was used: (1) we check whether any two of the conformers' energies differ by less than 0.1 kcal/mol; (2) if yes, we compute the rotational constants of the two conformers and check if they differ by less than 0.01 %; (3) if yes, we compute the root mean square deviation (RMSD) of the two conformer structures (where the permutation of atom indices was considered during aligning of the two structures) and check if they differ by less than 0.125 Angstrom. If all three tests pass, one of the conformers is declared duplicate and removed from the conformer ensemble. Steps (1) and (2) helped to reduce the number of

calls of the RMSD program (http://github.com/charnley/rmsd), as calculating the RMSD of two structures is time-consuming if atom permutations are considered.

Step 2: Medium-level single point energy calculation. The single point energies of the surviving conformers were calculated at the PBE-D3/def2-SVP level of theory. Only those conformers whose energies are within 10 kcal/mol of the lowest Gibbs free energy conformer were kept and subject to the next step.

Step 3. Medium-level geometry optimization. The surviving conformers were optimized at the PBE-D3/def2-SVP level of theory with a loose convergence threshold (keyword "looseopt"), using a GFN2-xTB (refitted) Hessian as the initial Hessian. Structures whose energies are more than 10 kcal/mol higher than the lowest energy conformer were removed; the remaining structures were subject to the next step.

Step 4. Medium-level frequency calculation and removal of imaginary frequencies. The surviving conformers were optimized to full convergence, and frequency analyses of the conformers were done at the PBE-D3/def2-SVP level of theory. At this stage, the number of remaining conformers is sufficiently low, so that manual guidance of the convergence becomes feasible. For example, transition state conformers that converged to the reactant or product, or have the wrong number of imaginary frequencies, or have a wrong imaginary vibrational mode, were manually tweaked and re-optimized until they have a single imaginary frequency corresponding to the desired cycloaddition reaction. Structures whose energies are more than 10 kcal/mol higher than the lowest energy conformer were removed; the remaining structures were subject to the next step.

Step 5. High-level single point energy calculation. The single point energies of the finally remaining conformers were calculated at the ωB97M-V[17]/def2-QZVP[7] level of theory. The RIJCOSX approximation[18-21] was used to speed up the calculations, again with def2/J as the auxiliary basis set. Solvation effects were described using the SMD[22] solvation model, with chloroform as solvent. The final Gibbs free energies of all conformers were calculated by combining the single point energies with the Gibbs free energy corrections (at 253 K) obtained in the last step. Finally, the Gibbs free energy contribution due to conformational entropy was added:[23]

$$G_{\text{conf}} = RT \sum_i p_i \ln p_i \tag{2}$$

where the thermal population of the i-th conformer, p_i, is given by the Gibbs free energies of the conformers G_j:

$$p_i = \frac{e^{-G_i/RT}}{\sum_j e^{-G_j/RT}} \tag{3}$$

For simplicity, the degrees of degeneracy of all conformers were assumed to be equal, thus they cancel out and drop from the numerator and denominator of Eq. (3). Note that Grimme et al.[20] used energies of the conformers in Eq. (3) instead of their Gibbs free energies, which we believe is less justified than using Gibbs free energies, since the entropies of different conformers do contribute to the relative weights of conformers.

The number of structures at the beginning of each step is listed in Table S10. For the reactant complex and all transition states, the number of the final surviving conformers is only 0.1%-3% of the size of the initial conformer ensemble, which illustrates the importance of employing a stepwise filtering process. It is interesting to note that the reactant complex and transition states have fewer surviving conformers than the catalyst **IDPi-4** or its anion **IDPi-4$^-$**, suggesting that substrate binding may have reduced the conformational freedom of the catalyst.

Table S10. Number of conformers at the beginning of every step in the stepwise filtration process

Species	**IDPi-4**	**IDPi-4$^-$**	**RC-1**	**TS-3a-1**	**TS-3a-1-RRR**	**TS-3a-4**	**TS-3a-4-SRR**
Step 1	N.A. [a]	N.A. [a]	319 [b]	2383 [b]	2383 [b]	946 [b]	946 [b]
Step 2	117	29	109	296	280	194	217
Step 3	32	24	22	26	28	28	23
Step 4	21	24	9	19	6	19	13
Step 5	20	24	9	12	3	12	13

[a] For these species, the first step is the generation of the conformer ensemble from a single initial guess structure. Thus there is no conformer ensemble at the beginning of Step 1.

[b] The number of structures generated by the docking process (before removing high-energy conformers and duplicates) is shown.

To illustrate again the benefits of refitting the GFN2-xTB method, in Table S11 we compare the activation Gibbs free energies of the **RC-1** + **2a** reaction calculated with conformers from the original GFN2-xTB method, as well as from the Round 2 parameters. The original GFN2-xTB parameters predict low enantio- and regioselectivities, both of which are opposite to experimental observation. The low selectivity is consistent with the lack of a unique lowest energy conformer (Figure S19). By contrast, the Round 2 parameters predict excellent selectivity (> 99.9 %) for **3a-1**.

Table S11. Relative activation Gibbs free energies $\Delta\Delta G^{\ddagger}$ (kcal/mol) and relative Boltzmann weights w of different pathways of the reaction **RC-1** + **2a**, where the conformational search and docking steps were performed using the original and refitted GFN2-xTB methods, respectively

Product	Original parameters		Round 2 parameters	
	$\Delta\Delta G^{\ddagger}$	w	$\Delta\Delta G^{\ddagger}$	w
**3a-1**	0.0	1.0	0.0	1.0
**3a-1-RRR**	-0.5	2.7	+4.7	9.6×10^{-5}
**3a-4**	-0.2	1.8	+3.8	5.3×10^{-4}
**3a-4-SRR**	N.A. [a]	N.A. [a]	+5.0	4.7×10^{-5}

[a] Not calculated

HF-LD calculations. Dispersion energies of **TS-3a-1**, **TS-3a-1-RRR**, **TS-3a-4** and **TS-3a-4-SRR** were calculated at the HF-LD[24]/def2-QZVP[7] level of theory. The RIJCOSX approximation was used, and the def2/J and def2-QZVP/C[25] auxiliary basis sets were used as Coulomb and correlation auxiliary basis sets, respectively. The catalyst anion, tropylium, and enol ether were defined as three different molecular fragments, allowing us to calculate the tropylium-catalyst and enol ether-catalyst dispersion energies separately.

The computed dispersion energies (which are the sum of the "strong pairs" and "weak pairs" contributions given in the output files) are summarized in Table S12. It can be seen that although **TS-3a-1-RRR** has the most favorable dispersion interaction between the tropylium and the catalyst anion, this is compensated by an unfavorable enol ether-catalyst interaction, which is corroborated by the relatively large distance between the 4-trifluoromethylbenzyl group and the catalyst cavity as shown in Figure 4C. Overall, the total dispersion energy of (enol ether + tropylium) and the catalyst anion correlates well with the computed activation Gibbs free energies (which predicts that **TS-3a-1** has the lowest Gibbs free energy), as well as the experimentally observed selectivity.

Table S12. Tropylium-catalyst and enol ether-catalyst dispersion energies (kcal/mol) of **TS-3a-1**, **TS-3a-1-RRR**, **TS-3a-4** and **TS-3a-4-SRR**

	**TS-3a-1**	**TS-3a-1-RRR**	**TS-3a-4**	**TS-3a-4-SRR**
Tropylium-catalyst	-27.3	-30.1	-26.5	-25.3
Enol ether-catalyst	-29.8	-17.3	-27.7	-25.9
Total	-57.1	-47.5	-54.3	-51.2

Close contact distances in the transition states. To understand why **TS-3a-1** has the strongest dispersion interaction between the catalyst and the substrates, we list important close contact distances of the transition states in Figure S21. The 4-trifluorobenzyl groups of both **TS-3a-1** and **TS-3a-4** exhibit C-H…π close contacts with the naphthalene and pyrene groups of **IDPi-4**$^-$. However, the close contact distances in the former (2.824, 2.744 Å) are respectively shorter than the latter (2.844, 2.839 Å). Furthermore, two C-H…O interactions between the 4-trifluorobenzyl group and an S=O group can be seen in **TS-3a-1**, while only one such interaction is present in **TS-3a-4**. Although **TS-3a-4** possesses a shorter (2.604 Å) C-H…π interaction between one of the alkene C-H bonds of the enol ether substrate and another pyrene group of **IDPi-4**$^-$ (shown on the left) than **TS-3a-1** (2.930 Å), the C-H…C angle in the former (129.8°; not shown on the figure for the sake of clarity) deviates much more from linearity than the corresponding angle in the latter (154.8°), suggesting that this C-H…π does not necessarily stabilize **TS-3a-4** preferentially compared to **TS-3a-1**. Overall, we conclude that closer C-H…π and C-H…O contacts are possible reasons for the more favorable enol ether-catalyst interactions in **TS-3a-1** compared to **TS-3a-4**. While these interactions can stabilize **TS-3a-1** by themselves, they also bring the 4-trifluorobenzyl group closer to the catalyst anion, so that other kinds of non-covalent interactions (in particular π-π interactions) may also become more favorable in **TS-3a-1** than in **TS-3a-4**.

In sharp contrast with **TS-3a-1** and **TS-3a-4**, much fewer close contacts can be seen between the enol ether and the catalyst anion in **TS-3a-1-RRR** and **TS-3a-4-SRR**, consistent with their unfavorable enol ether-catalyst dispersion interactions as shown in Table S4.

IRC analyses of the lowest free energy conformers of all transition states. The structures of the transition states (Figure 5C and S20) show a notable asynchronous character, with the terminal carbon (=CH_2) of the enol ether moiety being much closer to the tropone that the internal carbon (-CH=) is. To see if the reactions are actually stepwise, we performed IRC analyses of the transition states (Figure S22), and optimized the product sides of the IRC trajectories till convergence. In all four cases, the product sides of the IRC trajectories ended at structures that are very close to the TS (i.e. with one essentially formed C-C bond and one essentially unformed C-C bond; Figure S23). However, optimizing the geometries of the product sides of the IRC trajectories, or performing relaxed scans of the unformed C-C bond, invariably leads to the barrierless collapse to a converged structure that has two fully formed C-C bonds (Figure S24). This confirms that all four reactions are asynchronous concerted at the PBE-D3/def2-SVP level, but they are very close to being stepwise, in that the potential energy surface is very flat near the region where the first C-C bond is formed but the second is not.

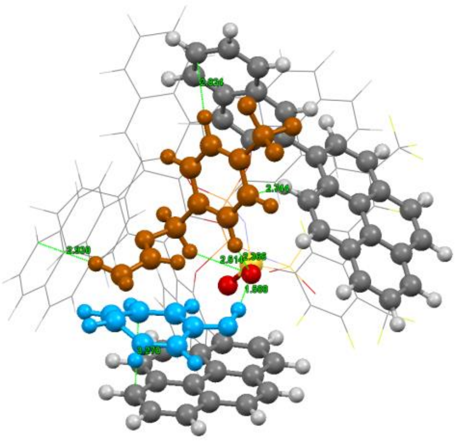

TS-3a-1

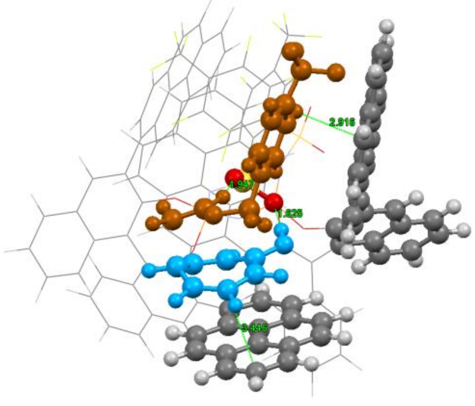

TS-3a-1-RRR

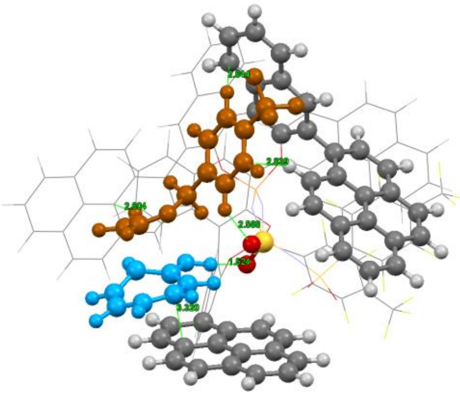

TS-3a-4

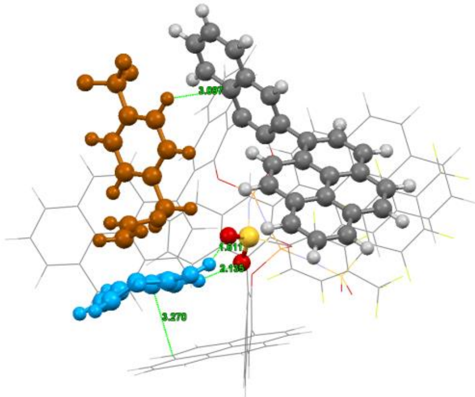

TS-3a-4-SRR

Figure S21. Close contact distances (Å) between the substrates and the catalyst pocket in **TS-3a-1**, **TS-3a-1-RRR**, **TS-3a-4** and **TS-3a-4-SRR**

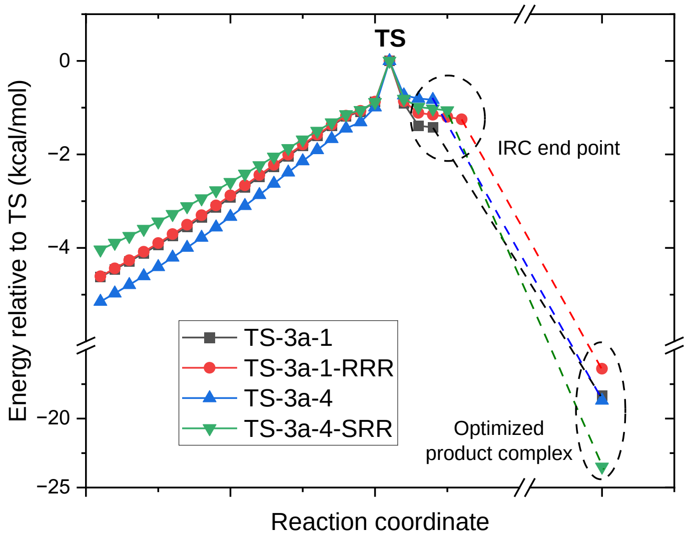

Figure S22. IRC plots of **TS-3a-1**, **TS-3a-1-RRR**, **TS-3a-4** and **TS-3a-4-SRR** at the PBE-D3/def2-SVP level. The energies of the optimized product complexes were shown for comparison.

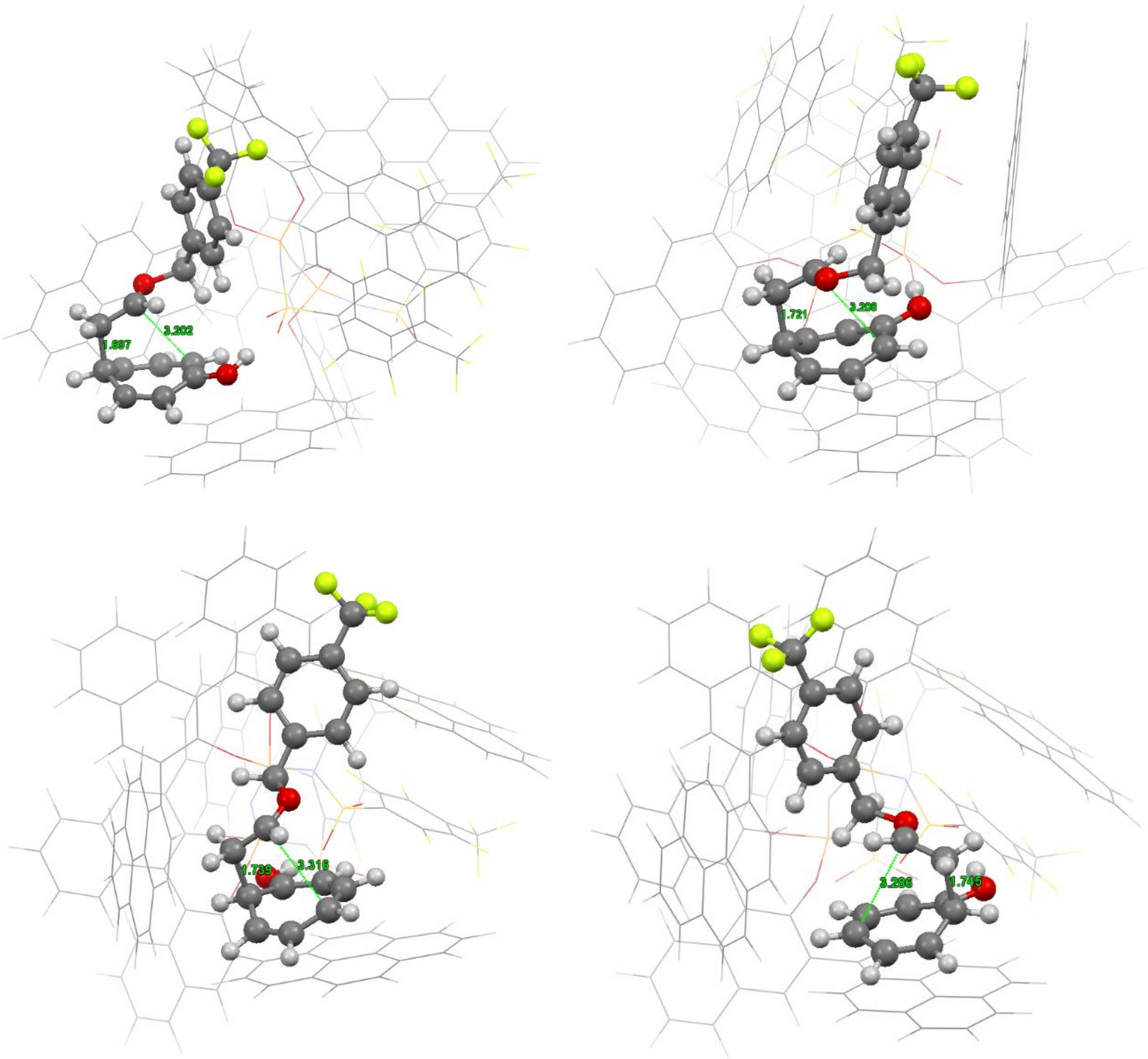

Figure S23. IRC end point structures of **TS-3a-1**, **TS-3a-1-RRR**, **TS-3a-4** and **TS-3a-4-SRR**, highlighting the bond lengths of the formed/forming C-C bonds (Å). These structures satisfy the default IRC convergence criteria (root mean square gradient 5e-4, maximum gradient 2e-3), but not the default geometry convergence criteria (root mean square gradient 1e-4, maximum gradient 3e-4), of ORCA. Therefore, they are not local minima on the potential energy surface, but the potential energy surface is very flat around these geometries.

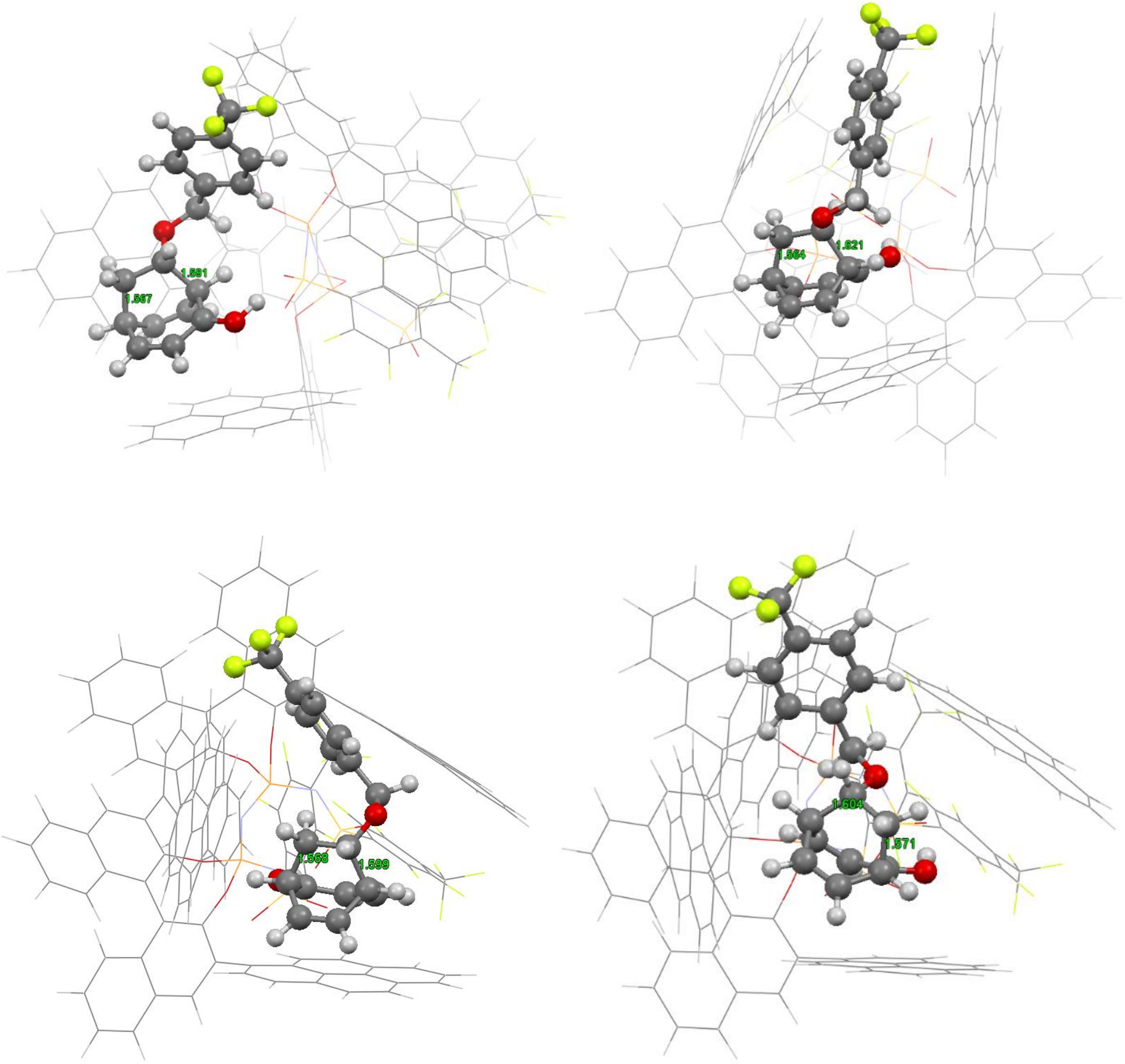

Figure S24. Optimized product complex structures of **TS-3a-1** (upper left), **TS-3a-1-RRR** (upper right), **TS-3a-4** (lower left) and **TS-3a-4-SRR** (lower right), highlighting the bond lengths of the formed C-C bonds (Å).

Simulation of circular dichroism (CD) spectra. As the experimental CD spectra of **3a-1** exhibits rapid oscillations near 200-220 nm, even a moderate absorption wavelength error of ~20 nm may lead to the erroneous assignment of its absolute stereochemistry. Thus, not only is the choice of an accurate functional necessary, but the vibrational averaging effects must be considered as well. Therefore, a molecular dynamics (MD) simulation was first carried out at the PBE-D3/def2-SVP level of theory in vacuum. A time step of 0.5 fs was chosen to ensure proper description of the vibration of C-H bonds. The Nosé-Hoover chain (NHC) thermostat was used to keep the system at 298 K, with a time constant of 10 fs. A 2 ps equilibration phase simulation was conducted, followed by 10 ps of production phase simulation. 100 snapshots were sampled at regular intervals from the production phase trajectory. The CD spectra of the sampled structures were then computed at the TD-ωB97X[26]/ma-def2-TZVP[27] level of theory (with auxiliary basis sets generated by AutoAux[28]). The solvation effects of the experimental solvent (2-methylpentane) was described with the SMD solvation model. Finally, the CD spectra of the snapshots (with a Gaussian broadening

of 2000 cm-1 for each snapshot) were averaged. The calculated CD spectrum of the (S,S,S) enantiomer of **3a-1** agrees well with the experimental spectrum (Figure S25, left), supporting the assignment of the experimental absolute stereochemistry as (S,S,S).

The necessity of doing MD averaging is illustrated by the CD spectra of some of the MD snapshots (Figure S25, right). The spectra of these MD snapshots differ a lot among each other and also with the averaged spectrum, especially below 230 nm, suggesting that calculating the CD spectrum at a single geometry would not suffice to give a reliable prediction of the actual CD spectrum.

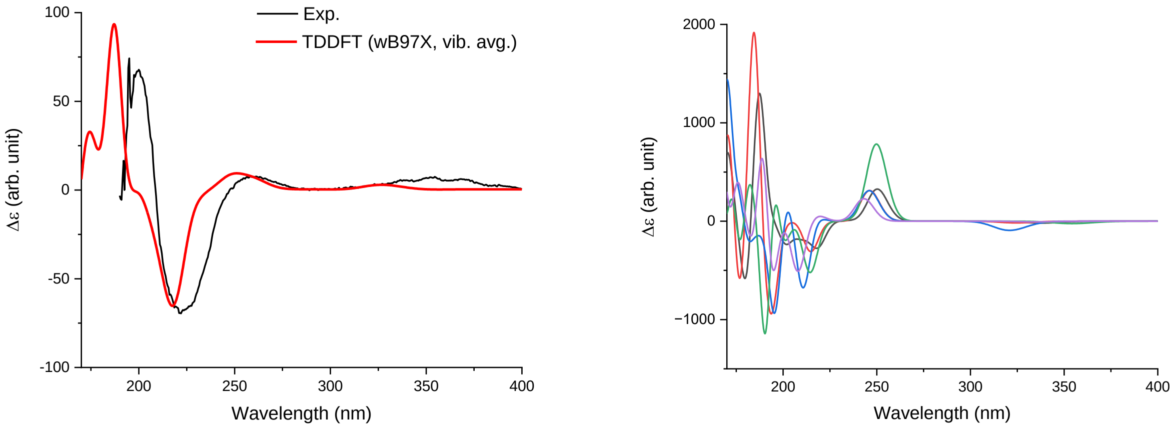

Figure S21. Left: Experimental and calculated CD spectra (ωB97X/ma-def2-TZVP, vibrationally averaged) of **3a-1**. Right: The CD spectra of five randomly selected MD snapshots.

List of DFT-optimized coordinates.

Unless otherwise noted, all structures are the most stable conformers of the respective species, as ranked by their ωB97M-V/def2-QZVP/SMD(CHCl3)//PBE-D3/def2-SVP free energies.

1 (tropone)

C	-2.94919404604667	-4.08638287291544	-0.17701535894245
C	-1.57223102866213	-4.08692656055528	-0.17454234193202
C	-0.68812404598271	-3.01050140123609	0.17327965694214
C	-3.83369288474083	-3.00925483592024	0.16762525634158
C	-0.97435559178306	-1.73221356253478	0.58387022931785
C	-3.54792104372658	-1.73119220857569	0.57923602341282
C	-2.26126543173386	-1.03974197162629	0.80507776650669
H	-3.44132273724437	-5.02485406613954	-0.47997232383902
H	-1.07975901187638	-5.02578755455941	-0.47572923973706
H	0.38490269623582	-3.25533744039161	0.09652332061228
H	-4.90663079005010	-3.25323727421149	0.08700745864604
H	-0.12322130117248	-1.06474400933388	0.80007287977187
H	-4.39929464504756	-1.06304067768236	0.79236953020317
O	-2.26147752816906	0.14055315568211	1.18287105269613

1-H$^+$ (tropylium)

C	-2.95742215926947	-4.08042626267207	-0.17508919817886
C	-1.56484907123096	-4.09028866482167	-0.17563932454681
C	-0.68585848718319	-3.03243678981723	0.16618007786788
C	-3.82365384974015	-3.01060792938025	0.16731267908729
C	-0.97338971552190	-1.73692620752002	0.58188962841923
C	-3.51909063218688	-1.72097905361685	0.58226821151002
C	-2.24102655288227	-1.11884935567509	0.77802230405288
H	-3.45303521240936	-5.01709693874134	-0.47721816237475
H	-1.08106585924118	-5.03291214804537	-0.47783935386895
H	0.38824516107210	-3.26913541982572	0.09233747282648
H	-4.90062858887852	-3.23359385459780	0.09382296788666
H	-0.09883745719332	-1.09514006222653	0.78992498455338
H	-4.36293971682053	-1.04531181592372	0.79795545239207
O	-2.31093313105131	0.14302910373725	1.18337264276505
H	-1.42553774746304	0.54348312912642	1.31384319760843

2a (enol ether)

C	-4.85709715477517	0.40495174518812	0.25456651147958
H	-4.96704908177669	1.42164728363250	-0.14637801992908
H	-5.72642356376250	-0.26304822194931	0.19418827596250
C	-3.72100591137443	-0.06183297962361	0.81150848449539
H	-3.64070379593869	-1.08793147559889	1.20546612298515
O	-2.54300353059500	0.58902256786978	0.98369984180824
C	-2.47034815768298	1.92774110597562	0.53035544963997
H	-3.24653744179585	2.54701972789036	1.04203075535695
H	-2.70140810313251	1.97478133437930	-0.56146907343427
C	-1.09758442898160	2.49642181545067	0.79356387451089
C	-0.08896082446368	1.73801308863239	1.41140312773318
C	-0.81690899754687	3.82210834274403	0.40526792013378
C	1.17762292896902	2.29399819137201	1.64027866184752
H	-0.30681097418563	0.70355460518761	1.70867715139329
C	0.44483397519831	4.37991021531077	0.63068172151525
H	-1.59699976905729	4.42585594487847	-0.08548113730843
C	1.44897174143775	3.61512138158824	1.25297021699883
H	1.96616159209953	1.69522537834081	2.11785194746005
H	0.65962723835863	5.41148400964760	0.31626143345632
C	2.79243993278995	4.24032989653450	1.53797915646176
F	2.76067726294687	4.99809718587536	2.66453467230208
F	3.76016030815805	3.30931701146369	1.71303729922837
F	3.18695969933220	5.05682848529790	0.52926695295235

TS-3a-1-H$^+$

C 2.64498389976296 1.70861193162689 -2.0472519652258

H 2.76419828268863 0.81080662312553 -2.6758746475244
H 3.58876584415589 2.20709886176079 -1.77566312345901
C 1.74407727126116 1.54985701796044 -0.9808140457819
H 1.74984992324215 2.21262010250623 -0.09441549138645
O 0.80619608110816 0.63657247812792 -1.06314030701434
C -0.07459364977005 0.41361235552661 0.10002122420199
H -0.54597336572787 -0.55966249243243 -0.12351218899154
H 0.57359013015943 0.30516410241204 0.99221602964106
C -1.08822761026597 1.50865890020123 0.26833141042642
C -0.81554556352745 2.61232011173441 1.10179597235324
C -2.30768266800298 1.45983207261529 -0.43573858953791
C -1.72044570059814 3.67658618921222 1.19289647071204
H 0.10683070043062 2.63776419276534 1.7030091568162
C -3.22220616207702 2.51524581427708 -0.3423940569796
H -2.54269173116148 0.58778477189668 -1.06495357087134
C -2.9142662185123 3.63341153935525 0.45314777859547
H -1.50211398286096 4.53923834398072 1.83871581097334
H -4.17468735855238 2.4796630483693 -0.89061860118188
C 2.0183593831623 2.92110679518873 -3.37684438037506
C 0.71249897230178 2.50325493589346 -3.85217289473698
C -0.5203306811634 2.93389976864614 -3.39788402681329
C 2.2443365165778 4.28396862846748 -2.88045995125914
C -0.83771949880332 3.9326489399947 -2.44337658800473
C 1.39429476914998 5.10793665240361 -2.19968464800566
C 0.0033404777983 4.89592006127033 -1.87259119931607
H 2.81900020910732 2.62086445760918 -4.07407289951699
H 0.70969518420848 1.71567157904587 -4.62268020411618
H -1.39549122784592 2.43168867080373 -3.8419107307607
H 3.2628158271097 4.67400345448197 -3.04609749651998
H -1.90262613314177 4.02574703319163 -2.17956208648541
H 1.78353584110266 6.07986183362332 -1.85598738863889
O -0.47650857106936 5.81624004743582 -1.02674604536773
C -3.80488779609278 4.85178881613054 0.41744544218692
F -3.79885782821222 5.53826874069233 1.5658148692728
F -5.0655780328802 4.57414896213839 0.07298955839043
F -3.31206805505355 5.71993523760239 -0.56250873982406
H -1.44965832917364 5.72111433727378 -0.9054182626197

TS-3a-2-H$^+$
C -0.74388359803205 3.90328260463704 -1.5184839955234
H -0.58279946012493 4.53786285193993 -0.63569518473833
H -1.52185988284836 3.12960867110133 -1.41922929090326
C 0.39007047044213 3.53037956964033 -2.24751321398977
H 1.36764174763861 4.04303344513858 -2.15829909886068

O 0.26065411088827 2.59998153605294 -3.16228650788376
C 1.39339946413336 2.19636116895652 -3.98990251270355
H 2.12522710625797 3.02864942096197 -4.04313251569044
H 1.86409713584663 1.33821821630333 -3.46350170333054
C 0.88604778155603 1.80812508855436 -5.35204098812824
C 1.57362570962964 2.21967997021614 -6.50761098024703
C -0.27198407904808 1.01436793576125 -5.48020952205173
C 1.10831095816449 1.85317906651109 -7.7775904796608
H 2.47831685167276 2.83897389261168 -6.41978274620678
C -0.74635922349655 0.65933627670871 -6.74743483167519
H -0.81022939592539 0.67697589824652 -4.58214041254779
C -0.05915234451492 1.08356224562168 -7.89898452277469
H 1.65035544572535 2.16832347859159 -8.68030166682504
H -1.64755140243875 0.03778693844034 -6.84889672118622
C -1.82059794136539 5.15978714588386 -2.67513063793215
C -1.35232234823951 6.49343545321464 -2.35952961208312
C -0.22169770587345 7.09911433485892 -2.86803359746158
C -1.76911344155022 4.65321510489717 -4.04221988889636
C 0.66988946923624 6.5975248590547 -3.85201523207249
C -0.76980301634002 4.82876720839808 -4.96453230772925
C 0.43975263053562 5.59342118258916 -4.80156079731213
H -2.76733566606232 4.91020293815672 -2.16952696996489
H -1.90620522487745 7.03200634155213 -1.57384287655769
H 0.05587714250927 8.07322109322859 -2.43398533901798
H -2.60903967964306 4.00924453636323 -4.34977847309332
H 1.6138790070022 7.15889529950774 -3.97356801438886
H -0.85673252921478 4.31298878395742 -5.93372828963006
O 1.35793990238576 5.33539077934525 -5.75505195278045
C -0.61524637563568 0.75572555582376 -9.27076913879193
F 0.35643259345897 0.72555266459271 -10.20642644096176
F -1.51882237978895 1.69259814433 -9.64991290764229
F -1.24399468132259 -0.43853847090532 -9.27514112773523
H 2.10415892925913 5.96508984915517 -5.68845038102031
TS-3a-3-H^{+}
C -0.5199885883403 5.32887671509675 -1.21625856562924
H -0.78156734533989 5.74848940895655 -0.22498127087831
C -0.45665230796174 6.09737536240396 -2.38159656446688
H -0.9820959137899 7.06328511569076 -2.37521814377205
O -0.15594908962231 4.0704757380582 -1.27004069512004
C -0.18331606313372 3.23335158304465 -0.06911095668837
H -0.55991909055317 3.82917447297129 0.78694329919717
H -0.91465266385831 2.43246997732282 -0.30110405621297
C 1.19467068305376 2.68894250377562 0.1842908371524
C 1.86077449510794 1.96610217998498 -0.82664012861376

C 1.84525216205402 2.92804614183116 1.40803510487062
C 3.17841527757743 1.53902274832819 -0.63592366155895
H 1.35002440551896 1.7528914398827 -1.77785530918595
C 3.16279569199095 2.49166267142486 1.60758795034045
H 1.32146003252575 3.46591007882962 2.2136347365017
C 3.83759653311511 1.82147792410632 0.5750687638367
H 3.70372783952593 0.98155681245336 -1.42475735886669
H 3.67495501507659 2.67834932513586 2.56226053840881
C 2.58051645725218 6.05064019377809 0.21683311005869
C 2.85610146635657 5.38527494159293 -1.00710842548252
C 2.38947249344508 5.71756498351956 -2.26712545383591
C 2.07453465418244 7.34013661857422 0.38974921358627
C 1.44490994644394 6.75441556978719 -2.60602014156294
C 1.68643730271144 8.2868053126019 -0.58634651361959
C 1.44361404442876 8.05519462422824 -1.93701366741232
H 2.9088977486278 5.53162179589524 1.12987587443971
H 3.49672100703296 4.49124871096814 -0.93459305303014
H 2.69985663327097 5.06871996787116 -3.10152959274476
H 2.05398672594919 7.71055013388616 1.42792044064324
H 1.31122773110263 6.89267577310338 -3.69135687346242
H 1.51447568363856 9.31613875767486 -0.22692670908797
O 1.0563172665489 9.03869001087924 -2.7640210819295
C 5.30626623897901 1.47907512029947 0.72374268639519
F 6.06232839802806 2.47819889075235 0.19676325610619
F 5.62203375682913 0.34403830050993 0.06950546136482
F 5.66111746226907 1.33893254372447 2.01629813668649
H -0.50569399985514 5.53306678726496 -3.32625531560583
H 1.0807729898113 9.90605144379071 -2.31023695082133

TS-3a-4-H^{+}
C 2.08876253750428 1.84757277996532 -3.48180919032574
H 1.09017491396482 1.54850080527888 -3.85426974470556
C 2.3761115119101 2.10312196486468 -2.13681514067454
H 3.44064647303719 2.0367005174296 -1.85888715492799
O 3.02506078222043 2.11538472955144 -4.36304512926849
C 2.78936999769111 1.96772250505361 -5.79229744076704
H 3.21535464589735 0.98167959151562 -6.07575847764872
H 1.69770068348452 1.95820579942022 -5.99161642380428
C 3.47873919170096 3.11060448268866 -6.48875817540507
C 2.81294951601523 3.85307267759993 -7.47962667144842
C 4.78962045752355 3.473405389503 -6.11326758334608
C 3.42830214103429 4.9705852294342 -8.063071771181
H 1.79868587086752 3.5645144114573 -7.79720872207279
C 5.4024339189473 4.59302215170098 -6.68701376266704

H 5.327081193524 2.88321341457147 -5.35597144379689
C 4.7136196440972 5.35454490202413 -7.65033858041963
H 2.90743765067572 5.55304761992827 -8.8361988736823
H 6.42716036324503 4.87463572102328 -6.40258046750212
C -0.19561122423258 4.51717502584167 -3.77780282323427
C -0.23558785694929 4.48139667084847 -2.35754018390336
C 0.8377610257241 4.35341569593431 -1.49881990186696
C 0.90954533176095 4.81193737873181 -4.57847818347949
C 2.21306907152652 4.07457315857461 -1.85084116903677
C 2.25307896949236 5.04225579627896 -4.20487142829954
C 2.86174072221396 4.72222884564627 -2.99166146595061
H -1.16458415541263 4.44186858765525 -4.29664227187283
H -1.23460172483741 4.54620313393711 -1.89650181081734
H 0.61864754332492 4.34220315553436 -0.41874608513623
H 0.70138899593539 4.95726554760174 -5.65133100557906
H 2.8930714665241 4.06993775181629 -0.98293144268935
H 2.91074419127386 5.45754408637419 -4.98673284539582
O 4.17540994814855 4.90725537324144 -2.79976065224131
C 5.34287507093275 6.62562286408579 -8.18698371627081
F 5.11434413579642 7.65097585825373 -7.32667617028183
F 4.82906118251861 6.9721958421225 -9.38263659359184
F 6.67878019989928 6.49679957974218 -8.31703460674814
H 1.65972064044512 1.72473998402517 -1.39377773703909
H 4.58080621257428 5.31111265074336 -3.59584215292138

IDPi-4

S	13.28331874090282	13.42007723343537	5.86866314996611
S	9.13601686420504	15.53253541249904	7.91229495073506
P	12.85735864918452	11.90257910436679	8.32698713350391
P	9.96205010567710	12.58794747711339	8.06205304398618
F	12.17050049843866	10.66669223400220	5.50008475422551
F	12.94373163712203	8.75868156093065	3.83934092765367
F	16.33437806744164	8.65549972182440	2.49224218380803
F	15.24339055963320	13.69219669908586	3.44401813385549
F	11.00303087982760	14.55001428091025	10.35053196898681
F	10.72654197839010	15.20059933484963	12.87025931360835
F	8.42954252981072	18.26025537221874	14.05969560755063
F	7.27692917458862	17.32779114000427	9.22615756588224
O	13.52092068674168	10.40697918259363	8.45895770921540
O	13.17710194672954	12.55280195292132	9.81105906299599
O	11.79678660993534	13.60781808506277	5.76631377252418
O	14.12241141507124	14.61526405133026	5.69094377991395
O	8.91867713973368	12.71255883428896	9.31843328227781
O	9.12022292216583	11.78613916607980	6.92087788949788

O	9.81651179113324	16.63500956350025	7.21365461460510
O	7.75621971604711	15.14722024018163	7.57936440379935
N	11.26444505782527	11.75913327397242	8.29567484801836
N	13.71885739169559	12.62353798087252	7.19756944821895
N	10.18550219344912	14.21069279859737	7.72889290539560
C	13.39902415979000	9.72618528207161	9.66985358034722
C	14.19865934122426	10.12283497690720	10.74720818805705
C	14.02001333713508	9.45528934714488	12.01570201975455
C	14.67655344695555	9.87182618642110	13.21312675732875
H	15.37163314473682	10.72048771185387	13.17764182104529
C	14.43497543872847	9.23568881777606	14.42208929171633
H	14.94706739125595	9.58557709738456	15.33106551721691
C	13.52924760710799	8.14574142361930	14.49913190536915
H	13.34996897534358	7.64674801565747	15.46312608499828
C	12.85867698084582	7.72938975453609	13.36093788558306
H	12.13471708988928	6.90124864749481	13.40789813316343
C	13.06771552060987	8.37593611842650	12.10677009217861
C	12.31176400525381	8.00679160199524	10.96059940686436
H	11.57953135161588	7.18939577659794	11.04496289426782
C	12.44235095275019	8.66809078087023	9.74439810238964
C	14.49083180609235	12.54531661818709	10.26612120717772
C	15.06968833676652	11.32796250804916	10.63941210029282
C	16.46331161666575	11.32620355935530	10.99760365801368
C	17.18815779229674	10.12191624033085	11.23683969162451
H	16.66545299634537	9.15977286496849	11.13889529100969
C	18.53322351385696	10.15542934956845	11.57510295482820
H	19.07658714084215	9.21385112369299	11.74577942854984
C	19.21598880319346	11.39480238135961	11.69498843568556
H	20.28206039722064	11.40888184054223	11.96709317331969
C	18.54238481933487	12.58290987652730	11.45668675251944
H	19.06602337039133	13.54838796389304	11.53348054544493
C	17.16280380423747	12.58513832067231	11.09511177062426
C	16.46460718443073	13.80133935104692	10.83927980085745
H	16.99175317440196	14.75882802332009	10.96891494562675
C	15.14347630121449	13.80771887580369	10.40971203602347
C	13.71234548814590	12.22953358864078	4.57426349503495
C	13.12218209641740	10.95647360199388	4.60962897754979
C	13.54344352270667	9.94231203028448	3.73817971688851
C	14.56103598701349	10.17504263791300	2.79456406755451
C	15.16216550318754	9.06628606296994	1.94768496660061
C	15.10965464147494	11.46856057376481	2.73446097014618
C	14.68181484605090	12.49723226369757	3.59160614883949
C	8.11073161293550	11.62618337972060	9.67804909203493
C	7.11348177355976	11.19373883623152	8.79842251074109

C	6.38406128098144	9.99838041487819	9.13703027356588
C	5.48914928608700	9.36619860047601	8.22282624450555
H	5.33642623733840	9.81362298230495	7.23102948891267
C	4.83248347190136	8.19316234392508	8.56598385094559
H	4.15698056803976	7.71519886597242	7.84071583457449
C	5.02816830042181	7.60160933546958	9.84241639663690
H	4.49699795753146	6.67420939829873	10.10368528229037
C	5.89805820613608	8.18561206824672	10.74917460405941
H	6.07092902626421	7.72802591678919	11.73540115204952
C	6.60499268421227	9.38039882485360	10.42056906923565
C	7.53840136238734	9.96139809962311	11.32235605188668
H	7.67756442900254	9.50836122711160	12.31565853881574
C	8.31761372989757	11.06102004896620	10.97266777808493
C	7.81980463617659	12.21939808641585	6.63115574797724
C	6.81122331016173	11.95315701314449	7.55518622749001
C	5.49693449835105	12.48706766227976	7.30078360131812
C	4.42515822748236	12.38815864619695	8.23644834987897
H	4.59774356432995	11.89000939712803	9.19985250497138
C	3.17725358898604	12.92358048210281	7.95190498579018
H	2.36806791132599	12.84059793882295	8.69304308986239
C	2.93795019034318	13.58421270923855	6.71844282826460
H	1.94238662217605	14.00003612547964	6.50255979083268
C	3.96524078359872	13.72001507353442	5.79860216174223
H	3.80037475088313	14.25061540686129	4.84820847901682
C	5.26390756182687	13.19429557431134	6.06534425364275
C	6.33455193356971	13.38444879213910	5.14905831536346
H	6.14675929378848	13.93682980807203	4.21596249263061
C	7.62123635377498	12.93016115908019	5.41281641876449
C	9.11755457684345	15.87768082235445	9.69212055015897
C	10.00665794945602	15.37647804749139	10.65613190542242
C	9.85138858264139	15.71945469435370	12.01567603301064
C	8.82128473723180	16.57519045889754	12.43973350787591
C	8.54233934611394	16.92273975931716	13.89382109690462
C	7.95797583996701	17.09790636733498	11.45892158886465
C	8.11114237529615	16.77449507935195	10.10577297871586
H	10.90607462371944	14.33822043277805	6.95896439328530
F	6.96416865361360	17.92142698562246	11.80167331518578
F	7.36218749943220	16.37571824624417	14.27870297160575
F	9.49377712028800	16.48346072491153	14.73178573453673
F	14.36292356249041	7.98552812251272	1.85434361421201
F	15.43008256484406	9.47955010760075	0.68922472381668
F	16.05863255373268	11.76309213898384	1.83984558642797
C	9.31385008664902	11.61363806843857	11.93348335804534
C	8.88174844355813	12.23888449663835	13.14069615685232

C	10.68992117483519	11.47533784875295	11.67627077592593
C	9.86720984524893	12.66295561055228	14.09898064147840
C	7.49495810547543	12.50390939545177	13.42788505655143
C	11.64645312649472	11.89837375806017	12.60115419066935
H	11.01669338598351	11.02023117048818	10.73289746633077
C	9.46367445697713	13.31473488581307	15.30683656467968
C	11.26393201374912	12.47299507274695	13.82849894929575
C	7.11064156828848	13.13440810341284	14.58658273245629
H	6.73767175872447	12.20666832191524	12.68832655466828
H	12.71330701392895	11.78247019752883	12.37481431317496
C	8.07299145338994	13.55580591600236	15.56725784188933
C	10.45220571057326	13.76147268680253	16.25029299210317
C	12.23120230620859	12.91140440359639	14.79713032169209
H	6.04624827332048	13.34297575952546	14.77567918701659
C	7.69929127043291	14.22746688885926	16.75250745087738
C	10.02914564696999	14.42567275770059	17.42273261501506
C	11.84214352110411	13.52880190784446	15.96006057618005
H	13.29674277409650	12.74728386075552	14.57515103336567
C	8.66851631755532	14.65483443748997	17.66842217864737
H	6.63226051244405	14.42141994034585	16.94129711811810
H	10.78776084304140	14.76863899746670	18.14342937833257
H	12.59251716856673	13.86791413220679	16.69146025132858
H	8.36024068871532	15.17998311654449	18.58514422599061
C	11.54472692797921	8.32155086574062	8.60587665400961
C	12.02526383578286	7.68163746898210	7.42922384564573
C	10.16602148516553	8.57844527451905	8.74359174361429
C	11.08752182171470	7.30349246229747	6.40626349100447
C	13.41262402979123	7.34718565206471	7.24222167973680
C	9.25642386373146	8.24661379407537	7.73663377768806
H	9.80477995203021	9.06338415280947	9.66200638517636
C	11.53721277788241	6.60058937900947	5.24397421234059
C	9.69149835410547	7.60648227727193	6.55742807470673
C	13.84147701262283	6.66823784006531	6.12754045197095
H	14.13333027708457	7.63778950596942	8.02011341227771
H	8.18843157841980	8.47452614729011	7.86435102503765
C	12.92596727013182	6.26707960251936	5.09453472655309
C	10.60375405192269	6.23684343870351	4.21343888729537
C	8.77665833600403	7.22244366573068	5.51620901705171
H	14.90772463671360	6.42144524971700	6.00581005882032
C	13.35216751150684	5.59781970220000	3.92640337810883
C	11.07924525526406	5.57375068779171	3.05924156555073
C	9.21366165584784	6.56672646021889	4.39046178885214
H	7.70989159304042	7.46312863802690	5.64731061934898
C	12.43873723248863	5.26277767078293	2.91886203343733

H	14.42065711501916	5.36193060524088	3.80659187185703
H	10.36306306254613	5.30101937669528	2.26827690294038
H	8.50220923445899	6.27689881580735	3.60120395723070
H	12.79162995074597	4.75344973072852	2.00947011782850
C	14.35313319129231	15.05042218302105	10.17186926629918
C	13.84504783630986	15.80120502374111	11.26978705093910
C	14.02423860426673	15.42619197513792	8.85708722208151
C	12.97989917920230	16.92073700410752	11.00638557234078
C	14.13937272485772	15.47431962409765	12.64100002288265
C	13.15427698800225	16.48865806390836	8.59835265481527
H	14.44591597558142	14.86232774872911	8.01596678090013
C	12.46082789568666	17.69648599458005	12.09204877462404
C	12.60697473879844	17.24595787058157	9.65692224124989
C	13.65324411429736	16.23008961212932	13.67961797112861
H	14.77195726410082	14.59901229554776	12.84688717501167
H	12.89178223840112	16.73220173295970	7.55759910694571
C	12.80942726889662	17.36936378551786	13.44687271862021
C	11.56428088633204	18.79080290568938	11.83351231935448
C	11.67991791971513	18.32290889560963	9.42934967796612
H	13.88835683011141	15.95818703053482	14.72008496341531
C	12.29073317491993	18.15412144021257	14.50036356353596
C	11.07481729197966	19.54808146424129	12.92103297866032
C	11.18148409198850	19.06416002615888	10.47319151228345
H	11.36653991802961	18.52667898601373	8.39495215112534
C	11.44067705869576	19.23533591140534	14.23690087200193
H	12.55243637509078	17.89299765415404	15.53724070074958
H	10.38448525413936	20.38191701163516	12.72138640080798
H	10.47456387413335	19.88731218110013	10.28452209528992
H	11.03775743449039	19.82974463186083	15.07037093286388
C	8.75306861882137	13.26850858915259	4.50683886577454
C	9.45819251585287	12.27623472016364	3.76978402576631
C	9.09816906081836	14.62760162350720	4.35887987385540
C	10.49228768191991	12.69067183176253	2.86106326675076
C	9.15307852926345	10.87279193393074	3.86273685848844
C	10.14722163163706	15.02440999588441	3.52640316762972
H	8.55001639674501	15.37899647306386	4.94527945406752
C	11.14137082210332	11.72645015485678	2.02573577282748
C	10.86106308184870	14.07566030278513	2.76462981973974
C	9.78638733846541	9.94648022124148	3.07126855251903
H	8.39238690309700	10.54077061786573	4.58369184838929
H	10.42776582742688	16.08678869839757	3.46614877833671
C	10.77019646437269	10.34075012225433	2.10085826555476
C	12.16054137991554	12.14083646490389	1.09945319523550
C	11.93658304820892	14.45000133282370	1.88613409622540

H	9.54364266749608	8.87863258548274	3.17173928851239
C	11.38072902534705	9.41395909084068	1.22625786868965
C	12.73625485410969	11.17936131809583	0.23936603644407
C	12.55948556007978	13.52349977353619	1.08513880598038
H	12.24434088667042	15.50657188739600	1.86021857831063
C	12.33886495029090	9.83613243408332	0.29601800625497
H	11.10626772464594	8.35063685925352	1.29994076988262
H	13.51840821530267	11.49530860409561	-0.46762383940959
H	13.37414581878512	13.82693068169121	0.40933413108799
H	12.80754523086610	9.10226204231551	-0.37531592764732

IDPi-4$^-$ (lowest free energy DFT conformer)

S	14.70372921444777	11.96953387905835	8.21986756853348
S	10.09047746288782	14.50158899082023	8.85644023280794
P	12.36296815283322	11.33600097805708	9.79649968875234
P	9.57134275345209	11.70036165476249	8.77445689591531
F	16.66235655811593	11.62841530213169	6.00447967913348
F	16.28911283016287	10.78003921389886	3.51586200555837
F	14.93123263144238	9.00361118887556	2.01244099723041
F	12.26919183217367	10.11844829367270	7.13850271959232
F	11.49672320513010	12.70884817476420	6.63071496852863
F	13.52744492820345	13.33731208954916	5.13401578377881
F	15.75977938916919	16.37902298949940	5.86368100384311
F	11.28424319241752	17.20606745200730	8.25982625318493
O	13.12497729353631	9.85701215705062	9.86371979627414
O	12.47693198063099	11.80998894345019	11.38354583927581
O	15.21681953311690	13.25316751221574	7.68683698310164
O	15.58978945891223	11.16325081877831	9.09207762119543
O	8.23151082574810	11.37115347289314	9.70997880935383
O	9.24861997055252	10.69243827341873	7.48442921741415
O	9.13953523602337	15.62780593837289	8.90192988602737
O	10.89574666191332	14.19382050998393	10.06541700079369
N	10.82094605005151	11.09392625399569	9.53558428141653
N	13.22121961496513	12.24169528723419	8.79550369749404
N	9.33510806804899	13.19445953381156	8.27317334350009
C	13.05779855313343	9.02410859731192	10.94681067161883
C	13.65064779774912	9.39141102627677	12.15813962662724
C	13.48697828427110	8.51603198729978	13.28918612975610
C	13.94348418897159	8.86144871561887	14.59718659240350
H	14.45844578983753	9.82128821767551	14.74048355683684
C	13.72513514391800	8.02011663532788	15.67923821812272
H	14.07798261276654	8.31628025590506	16.67892281946766
C	13.04292487377206	6.78642859616145	15.50822524137461
H	12.87526878559938	6.12607741018869	16.37262397632143

C	12.58074171882034	6.42382384469972	14.25213499468413
H	12.04103095147881	5.47474994092016	14.10657753765701
C	12.77863611641622	7.26911087577597	13.12059188042288
C	12.28982695450939	6.90853615788442	11.83143705669126
H	11.80308444768439	5.93021195633598	11.69566967858891
C	12.42778083074192	7.75628272711859	10.73993697245409
C	13.76663736964313	11.86978904810136	11.89570684361460
C	14.39886464652808	10.67349641139214	12.25000901615260
C	15.78513072671411	10.70926141630798	12.63337268554279
C	16.55641830500958	9.53135985549062	12.86189170360397
H	16.07984058356052	8.54949444902626	12.73566529398835
C	17.89525008440778	9.60977626258390	13.21823610918062
H	18.47086252171069	8.68530931701273	13.37904738539008
C	18.53058095973187	10.87136151211654	13.36384979300348
H	19.59256392896341	10.92225077332331	13.64907878993499
C	17.81572722029434	12.03421711457408	13.12350368393735
H	18.30075914970246	13.01934291865690	13.20731837921991
C	16.44217001757908	11.98779066807888	12.74153510595820
C	15.71433639720267	13.17462373308591	12.45973456176217
H	16.21450052708597	14.14715073479998	12.58256392267660
C	14.39262740343024	13.15032944381879	12.02204343760810
C	14.45392831147884	10.99051046167293	6.66501283439762
C	15.48833856134450	11.06679175165135	5.71261309292794
C	15.29754352177133	10.60996702328741	4.40295153037991
C	14.08902744282917	10.00681045900911	4.00165926769789
C	13.88836794567127	9.68408853636183	2.53963626253462
C	13.09303543927361	9.85079750029665	4.97951798160820
C	13.27995006948178	10.32235005322866	6.29214036712036
C	7.87591181582725	10.07135953553951	9.96680615656494
C	7.25167480288936	9.34685876280813	8.94334209241089
C	6.93972694598912	7.96159535713976	9.16475293507834
C	6.44195270488877	7.10568091384859	8.13652861213635
H	6.28783415565496	7.52081841927850	7.13090300747541
C	6.17145799726575	5.76782741546759	8.38753151625520
H	5.79874362345037	5.12541702676045	7.57521927246836
C	6.38086403213295	5.21953859207478	9.68126686049815
H	6.16127547296121	4.15767253022358	9.86970254534290
C	6.87770301653266	6.02043161589654	10.69759191837648
H	7.06381295936647	5.60349210518255	11.69984778746169
C	7.18188502305726	7.39611706186163	10.46992462214772
C	7.74840405855930	8.20743959995022	11.49110710049126
H	7.90941450706269	7.76456089859997	12.48591292892739
C	8.12811441038856	9.53233277592367	11.27147890780920
C	7.96573586693184	10.70847521983516	6.96764083770335

C	6.94490060266916	10.04271001037188	7.66314094947920
C	5.59551872217581	10.11890485456492	7.16354568751404
C	4.47326998558661	9.59463148949240	7.87442335033262
H	4.63558463640442	9.11773089610669	8.85024418790191
C	3.18838223226368	9.69112933899522	7.35994789380660
H	2.34177004195728	9.28234887156881	7.93260886532418
C	2.95541854379178	10.32101498661684	6.10879282282363
H	1.93243473323973	10.38768841086310	5.70795853666510
C	4.01792931669756	10.86972047251268	5.40810858370575
H	3.85185183988435	11.38502782817508	4.44900407620744
C	5.34895177515129	10.79884238342226	5.91590667392753
C	6.43436759919414	11.41122904682012	5.23164930148237
H	6.23998466331706	11.93313217436833	4.28184252761054
C	7.72905169595416	11.40118107604306	5.73684177662264
C	11.33327894774520	14.91499594853252	7.57083386690307
C	11.93204852470884	13.96994668312823	6.72435028792569
C	13.01627103296533	14.30923232582997	5.89934469624065
C	13.51812489876574	15.61820916660091	5.86251249724454
C	14.72944611222962	16.02557437152938	5.05527622462604
C	12.87293141164175	16.57944026124504	6.66184652185630
C	11.81633233476211	16.23506601182462	7.51512362078010
F	13.26193511693986	17.85935453473959	6.61906242475066
F	15.17385530156031	15.05144119113782	4.23934988153899
F	14.46484845965377	17.10624089427499	4.27002919819477
F	12.77334365515300	8.96171782359674	2.30181706114035
F	13.77101947858533	10.83440583663203	1.81264449892246
F	11.92401259789933	9.26324287336318	4.70248361511722
C	8.85227897729017	10.28805623572256	12.32735330093406
C	8.54967508110658	11.63321044668862	12.70497495568393
C	9.90316622443536	9.61433602351871	12.98921636845892
C	9.36737128143517	12.28290007339649	13.69657579900520
C	7.44151440811565	12.37551361665461	12.16229551772626
C	10.65995815292757	10.22355648759399	13.98777849554083
H	10.15440755931935	8.58949380803093	12.67946561420789
C	9.12520831012941	13.64883187089489	14.05491613391869
C	10.42739795417087	11.56626178774285	14.34739400087084
C	7.22920952044304	13.69387247686460	12.48072188520782
H	6.75721543173578	11.87644357084832	11.46591855099993
H	11.46673142222080	9.66599824850496	14.48189033902577
C	8.06990541821537	14.38431543290299	13.41803141063091
C	9.93846339522346	14.29436778399956	15.04997895497958
C	11.22711007250095	12.23376378855253	15.33794699252581
H	6.39472577528639	14.24463580928901	12.02044584253423
C	7.87161421369608	15.74188121604877	13.75718636390946

C	9.69316656083045	15.64985039292292	15.36685393467555
C	10.99331986564124	13.54394131504266	15.67733965447998
H	12.04375690536270	11.66672456005855	15.81132789390080
C	8.67561934901291	16.36527175618526	14.72086879116851
H	7.07144306208462	16.30412324375960	13.25168083432982
H	10.32808520870954	16.14268947176771	16.11934424595229
H	11.62075660209132	14.04936704997120	16.42786964081115
H	8.50938722611427	17.42457817187363	14.96986843507323
C	11.96939307332933	7.38053418292179	9.37694578303153
C	12.90559645678542	7.25383031390716	8.30802421700502
C	10.60287972066893	7.14884048265843	9.13684807630119
C	12.43135468502336	6.87758328301786	7.00560741036147
C	14.31577466063017	7.48956251780245	8.47805067588713
C	10.13626367864962	6.79908099703313	7.86613942036752
H	9.88824284861883	7.28411928959608	9.96137489100891
C	13.35290756956110	6.73254739715046	5.91894683557819
C	11.02963300479829	6.65381495410946	6.78417798687280
C	15.19567599861073	7.37406427397258	7.43056312246231
H	14.69266337567710	7.79156464990041	9.46414471709994
H	9.05923769724421	6.64356183365098	7.70479074634327
C	14.75265690958922	6.98538476615521	6.12068480796687
C	12.88028870620086	6.35307092617766	4.61569843955351
C	10.58377655971906	6.29001888211728	5.46571120167345
H	16.26415731930708	7.58930048316898	7.58476652803002
C	15.64255633068640	6.85133679697570	5.03081193300258
C	13.80957243263675	6.22530261287296	3.56001123434848
C	11.46970857412945	6.14020003118564	4.42734334779187
H	9.50474440430119	6.13501132661151	5.30838912215413
C	15.17384688258665	6.47075656560251	3.76758807435793
H	16.71238271411686	7.05770738644894	5.18926901471965
H	13.44290096209349	5.95045819753645	2.55915720289240
H	11.11298789209483	5.86603959383197	3.42223017587214
H	15.87775528136169	6.38365877045699	2.92674020298210
C	13.65119514823811	14.41208024339201	11.76840694868440
C	14.15594070161555	15.40845241338499	10.88208324533346
C	12.46050890073458	14.65508624782687	12.47938676250792
C	13.47527625852032	16.67232780461952	10.79350391272594
C	15.30420529740011	15.19541301515592	10.04235743179226
C	11.77937869660668	15.86678097627339	12.36799506729380
H	12.06370231832414	13.86872578251762	13.13452521759062
C	13.99673469632658	17.71242858047298	9.95870445586475
C	12.27263169265342	16.89863026701099	11.54362074917915
C	15.78867207193480	16.18455206024759	9.22208332832714
H	15.76536837220054	14.20025031799566	10.01708410760833

H	10.84303318751676	16.02061171487061	12.92194608181894
C	15.18321393897277	17.48642023091736	9.17996529726246
C	13.33238808655217	18.98573493684533	9.89004192804025
C	11.61025390053681	18.16893200804185	11.42958805982448
H	16.63620657280413	15.97298984964907	8.55360224522403
C	15.69322515955880	18.53577355336584	8.38188544077627
C	13.88354080873171	20.00416572615218	9.08057243280540
C	12.12193070631712	19.17318713877706	10.64474495505460
H	10.67422230656803	18.31520290988383	11.99106029616662
C	15.05346067982936	19.78070131275600	8.34203314247278
H	16.59096937252327	18.35072636232508	7.77291196218547
H	13.37233009233579	20.97871424808467	9.03146359376574
H	11.60421023548822	20.14262305681359	10.56868059600181
H	15.46201182327089	20.58438073319646	7.70979409935188
C	8.82101708043256	12.08462823665243	4.98320047788213
C	8.93961170405297	13.50328279952150	4.99267570453981
C	9.71033720283477	11.31554438284809	4.21190121935846
C	9.97346103525458	14.12395215349012	4.20898766155455
C	8.10428915674986	14.34235984199535	5.81048110136256
C	10.69925283277663	11.91726299832882	3.42775677197225
H	9.62358597436937	10.22012183624973	4.23235166838562
C	10.16146340061135	15.54289969146957	4.26321600383576
C	10.85246430569365	13.32007692734292	3.40702274749498
C	8.30150476141591	15.69897853140741	5.88123661530270
H	7.33740476210327	13.86758194787199	6.43554427219860
H	11.37051998120736	11.29655148436397	2.81840883353044
C	9.33728941351389	16.34487319453444	5.12392915144207
C	11.21249180318244	16.16257504220642	3.50407372411932
C	11.88700949683061	13.96728704600693	2.64441501898772
H	7.69661602327880	16.30709185547360	6.56855348078873
C	9.59204761920015	17.73125138937144	5.22448606274294
C	11.41388274437133	17.55652546548698	3.62049821271997
C	12.05666262397219	15.32968238880112	2.68714428320442
H	12.56844692489456	13.33429850590259	2.05606352556146
C	10.61925842486312	18.32624712634941	4.48050339991445
H	8.98170228677166	18.33304554849143	5.91484704130465
H	12.23340781210030	18.02379905116836	3.05502310984061
H	12.87344168758686	15.80710640489739	2.12448146517147
H	10.81463693164705	19.40443367398418	4.58356355956530

IDPi-4$^-$ (lowest energy GFN2-xTB conformer, original parameters)

S 12.36721578890350592417 13.50376626322764117560 6.21941651327135591742

S 10.78192648175808976418 15.25909147194413684190 8.08079447469433276297

P 12.61126378327381658551 12.32728135651051992738 8.61033918888271543324

P 9.7428675962806021 7153 12.7947867038786320 3928 8.4381148672411399 0476

F 14.563990859928795 27396 13.621709599763738 29647 4.0557298019942900 0692

F 15.385786524105240 50254 11.696913212836081 54147 2.4126506788733048 6585

F 13.176670244675788 62357 8.2942469618509946 3719 1.8329988225840698 7567

F 11.111313899961251 29471 10.799930764658446 59140 5.6166706317449106 5780

F 11.204635121379704 18792 14.534884350246395 35698 10.938828822461182 71041

F 10.217643639738975 38074 15.647416014928982 26411 13.167994161963088 47304

F 8.1275921264938819 6326 19.168784224825305 52884 13.077533945937600 98985

F 9.3498519729315106 0194 18.027623566418299 10586 8.3750281124893639 6282

O 13.304189568977157 87591 10.850630641502037 93190 8.7396111626616743 7061

O 13.201064369060867 77583 13.005637771642014 39973 9.9384291597284359 9093

O 10.951541510459252 39316 13.350379384894367 26286 6.4353609505434947 7351

O 12.815860580483255 04899 14.755954831824039 88565 5.7184392130453369 7736

O 8.7556983801254304 4670 12.627248203190003 29505 9.7451024959012588 7664

O 8.9836885711414726 7851 11.896159165890107 80058 7.3588075222327988 8113

O 12.038224160928530 53726 14.621540184973621 73434 8.3914455877630196 3121

O 10.768228276632163 08736 16.193439629580140 60405 7.0121442487973375 5347

N 11.067660707068565 52874 12.020046514962404 06061 8.8440904623375313 7756

N 13.310124438836458 79339 12.767024988410527 79710 7.2345832768132787 2938

N 9.5036968722237240 5699 14.371772936119850 07260 8.2410573627727963 2418

C 13.029547769671292 91165 10.216943762665589 90096 9.9319156128017027 6405

C 13.715042945746830 58272 10.570279966452872 59980 11.076721789352669 48940

C 13.301295853667310 70906 10.053320225517820 67410 12.334587074690954 72220

C 13.876809456262900 96584 10.470723106436489 40243 13.552979632744044 38039

H 14.704530647938513 61003 11.163188432165224 38411 13.530209177671039 81078

C 13.384065280509277 68447 10.029363657020933 68668 14.749863299106680 25889

H 13.827142379841214 74767 10.365253562830261 55779 15.675416000911647 88466

C 12.293926267732707 91318 9.1485734939307263 8577 14.790094439818002 31427

H 11.910277859911142 07320 8.8205398174110971 6097 15.744670010624432 16597

C 11.721581046554181 42670 8.7167272904181043 4580 13.627107337683836 97395

H 10.880718984125572 03528 8.0385383813444928 3359 13.646665195191287 62283

C 12.202546520065856 93869 9.1543455933732520 4353 12.375718479812064 74287

C 11.611330175860967 27314 8.7301416389278365 6706 11.169823428386738 58409

H 10.818885770417617 56448 7.9960424930060884 9182 11.203507463254970 48872

C 12.018309781699231 91956 9.2283953252506965 0148 9.9569252332914519 5987

C 14.506500454503751 02973 12.734883286478337 89375 10.285742527949238 93362

C 14.808703221271375 83834 11.550877116510461 56192 10.920747987683810 72023

C 16.156263401095692 69998 11.280227025159488 71849 11.287566195151550 01920

C 16.554929751904964 51887 10.054109150819432 01699 11.860759556341884 39092

H 15.812537602056423 22489 9.2881395604093484 0530 12.024469212098102 48854

C 17.860781766037845 84131 9.8303231972878055 7000 12.200480059568564 07295

H 18.151216820670953 21145 8.8872061114219818 0232 12.639022033567618 66360

C 18.833154382861998 49927 10.816695452992728 83199 11.980554994671518 18749

H 19.857854281376472 01652 10.626741117210251 27508 12.263126002594100 68304

C 18.4845917669000418528 12.00261716617689700115 11.39727648392823411427
H 19.22972042303160478127 12.76081311822062325234 11.20474679959576924659
C 17.14940305203657544553 12.26185775008528189289 11.02300831354686927455
C 16.79307694761133973316 13.45506158849222977381 10.35898364605489874180
H 17.56179218164544408864 14.18021417223921964990 10.13451359969064746736
C 15.49807818255090374748 13.69498083768352714173 9.98210322669829963615
C 12.77208819813088958028 12.30368497722069420774 4.86347095473185664360
C 13.87140727670269768623 12.48444136668527093548 4.03726246712006986428
C 14.30675516261469581991 11.48581761609013973668 3.16882591666453983237
C 13.61769707324264189197 10.27823252624502714525 3.07440347280891179693
C 14.11132848004764817063 9.19125024835279447188 2.14984443036036720898
C 12.51584953690336554644 10.09173660653111070928 3.90402885589513148901
C 12.12982921406909042616 11.07925630407471828676 4.80781445577557953186
C 8.16216024889915026108 11.43005184243986249726 10.04829235580737112343
C 7.19460885154993068369 10.91204829260563080595 9.20483753495239653830
C 6.67303814468496625523 9.60728230841785268979 9.42426754909796748905
C 5.83039954286758455027 8.96026095704355007854 8.49557689049690445415
H 5.54661027534958872565 9.48223104374557124174 7.59427144781578267185
C 5.39024022075625897088 7.68445715201605317191 8.71947722576825690055
H 4.75168599140869840625 7.19851778191587587941 7.99668275660775407232
C 5.76466208423369241842 6.99300678833948285984 9.88124637225389967909
H 5.40186497710358626279 5.98899747818381733566 10.04295733328434359066
C 6.58991975680250430258 7.58706445242891458491 10.79393557346869236824
H 6.89319842994235187916 7.06167083497889880306 11.68805184295427856966
C 7.07002739105844124623 8.89715382148847488963 10.58647143348881947134
C 7.91759433907377285777 9.53263043058352010917 11.51189565624554766998
H 8.17518226833531436171 9.00920526674868860084 12.42176393817111623719
C 8.45954416239649908960 10.77764764994087975936 11.27900528851941608366
C 7.65067018855197389371 12.21395855597717172714 7.17384222242097724376
C 6.72533920634816784201 11.73285480853319562300 8.07047450849054825994
C 5.35335406773880251308 12.08700486825361331000 7.92297189165663429122
C 4.36455521101825105745 11.71861223390331119276 8.85972051904520796484
H 4.65216122736505521829 11.14993952746495864403 9.73028316927822700677
C 3.05812086841682528870 12.08241254060535041503 8.68199528892547078840
H 2.31341423433812920507 11.79355117640343308949 9.40868974250139800120
C 2.67347370615036572516 12.83550128122755218385 7.56344402088804912410
H 1.63623725103359540789 13.10636567873131141937 7.43439573161210720542
C 3.61233318592491636778 13.23586500564281465131 6.65469284930034010728
H 3.33166653472281426573 13.83435292477861366933 5.80028550528022979194
C 4.97012201298976563635 12.88809653393829357526 6.81449162031245858628
C 5.95288109573438450894 13.33775779444798814666 5.90858116784423881285
H 5.65257697792858238017 13.95171035072670662203 5.07186501711017800886
C 7.27624478101956828624 13.02312367233426293467 6.07839822866545453905
C 10.35403966155074328981 16.26045889128981158933 9.58589807812323435599

C	10.52562111910238940027	15.67050758298308288374	10.82793068277733361526
C	10.00461473449125726631	16.23274277163041645622	11.98654600734067798840
C	9.27166840351077325977	17.41911871591617355648	11.93007746511318245553
C	8.76608367755628847817	18.01074446741408152661	13.22482533101593382696
C	9.06382114606027222692	18.00255580379837638816	10.68423966674329150806
C	9.59459831882561609007	17.41792154218974175706	9.53292569192685590451
F	8.36328099323287688094	19.13083175489380138856	10.57000752714323077441
F	7.92166431406293636286	17.18452668449645059923	13.85699563282387813956
F	9.77779791114712004685	18.24641574313030290000	14.07708163787777877474
F	14.57318587202303916683	9.67046465678075684025	0.99103044799200668002
F	15.12385241827831094952	8.50266453200136851365	2.70544416521969077749
F	11.80468665026725538780	8.96906696193772923209	3.82851926028284506032
C	9.29946979267553430759	11.39698212079719041867	12.31566852004732304238
C	8.85633696823585125912	11.44996316830983751345	13.65833107868016860209
C	10.55379282474398294767	11.89884682179049946171	11.99064260542500193196
C	9.74204668784108385182	11.93053192258128269998	14.65690240936621790979
C	7.54141539511968961307	11.07592680832444465011	14.05956232728964394596
C	11.39002951752848957767	12.42076210251422274666	12.95345306619444869511
H	10.88142187042786979134	11.87254724752032686297	10.96591569080910666401
C	9.35247248123403807085	11.92921005514874011055	16.01871225462632963854
C	11.02186793821134536131	12.42139650502320513681	14.30008892183103341722
C	7.16539208116133252702	11.08063602639181688403	15.36324513815027970054
H	6.82689121348485539187	10.80155506326123671101	13.29813972337542615776
H	12.34677304749325443822	12.82356137722298150550	12.65993010782715089135
C	8.06316971114757485850	11.47763134909528659477	16.39157483944876858573
C	10.24910884326217441753	12.38269241686517752044	17.01853282608832884648
C	11.89195923517125308422	12.89610157414466584669	15.31947526206760024081
H	6.16246433574343566164	10.79156029648602377335	15.64403049522053201770
C	7.70422641904643601407	11.46607514671475414048	17.74302332061456155543
C	9.84987874980967426097	12.34900167293909412081	18.35756132878437085765
C	11.52809536334547324543	12.86819157445474282042	16.62461802817321654402
H	12.85808395776192369908	13.27687335834936455115	15.02200259768180501396
C	8.59324472853896459412	11.89207746347615390903	18.71169868980626915800
H	6.71905120777210473904	11.12170194123478594861	18.02287678928593095407
H	10.53634565252514576628	12.68985804470609757288	19.11925263955066611743
H	12.19815766935585266140	13.22290962154515092664	17.39494323325524760548
H	8.30244408991136673137	11.87511230583205978917	19.75188798790009769846
C	11.41516966181205994246	8.74061878558867455524	8.70728075434252524190
C	12.18983818172544708602	8.10006392818717380067	7.71890351189947931942
C	10.03965231195359208982	8.84985869670785163521	8.54050865773265144298
C	11.53623362578274758050	7.53946183849085294071	6.59147767776813964247
C	13.60041517972289781824	7.94248626414867597845	7.82878141598805044055
C	9.40910052194584345386	8.35561936664759841165	7.42031967785857826669
H	9.47020068563947248208	9.34972174405577582945	9.30487187644446223089

C 12.28003931439416795968 6.82823122958539929783 5.61798588253750441623
C 10.13415737498098856406 7.67469143159315958513 6.43728265554416090310
C 14.31685123495173428410 7.29515281512927860064 6.87501812992841809802
H 14.10076146061340018889 8.35680950953651624502 8.69013468081262807630
H 8.34093153355979133323 8.47184839558256541636 7.30794381559182948394
C 13.68600376279873387375 6.71236293910698034182 5.74145182684198118750
C 11.62500528085862860905 6.24217657918974300202 4.50553277106264271623
C 9.50484195016203514683 7.09261739616898001515 5.30294282131873373487
H 15.38920890066524371775 7.19761151095908058295 6.96766256321291965747
C 14.40548680527877500879 6.02676026053949076555 4.75669972670953988825
C 12.38316334684361308405 5.55824772569234593078 3.55113804070786098777
C 10.21608359245965402806 6.39608187058611310505 4.38181898355956267466
H 8.43543131426750036894 7.21267444011114822189 5.20073836012009227403
C 13.75675284680436760709 5.45761510146511508168 3.67725303687636317918
H 15.47907486921554465198 5.95117240768642918880 4.84716197811351889868
H 11.88384830767005517771 5.11671551791205647675 2.70139165086286237027
H 9.72835959311447773246 5.95073580798019996507 3.52654956083682824541
H 14.32770108047812840368 4.93523659796972236080 2.92421305845133217005
C 15.06621675521525105523 14.86660505495897588446 9.19779417254294884287
C 14.28563487804911602552 15.88326123462768180161 9.78224880127906004645
C 15.36821429179048692504 14.91134932619976005697 7.84323811449735242007
C 13.76359478639460398597 16.90559714316182748917 8.95516957180233674762
C 14.01468630154457173376 15.92667978185887101006 11.17582396449338055788
C 14.85383893145228384469 15.90228634633174209512 7.03267480758742902225
H 15.97634551830338089928 14.12758851382032254662 7.41710682253548192477
C 12.97826708976973186793 17.93774844134082613323 9.51894614881889111757
C 14.01675653456393888519 16.89272270803216002832 7.56282204316791251131
C 13.29411722748856128362 16.94046807429061374251 11.72437888623137780542
H 14.40899368901711596891 15.13803564950376667753 11.80027308479088077320
H 15.06673085471383188860 15.90107491281778884229 5.97593955679638355605
C 12.76071259347345865365 17.98366500548195290321 10.91853822604646317984
C 12.41723501261063233869 18.93775565084177969766 8.68548627965348529756
C 13.40495576497894170132 17.87690489565207840883 6.74584342444435591801
H 13.10858676609246842304 16.97189869961250963115 12.78928205107261462103
C 12.02860009597206136789 19.04725719766967273472 11.46135144072630751566
C 11.67451521196545805026 19.96994764233787833518 9.27120447818268011986
C 12.62884812090724295786 18.85568120207902609309 7.28257708782365664746
H 13.56388886424791095919 17.81578098752574845776 5.68072397408312745881
C 11.49727570930768116853 20.02720997471421426894 10.64243064451702558415
H 11.88753220825774015168 19.09681342774113232963 12.53167395210829582197
H 11.24146931812662764116 20.73023674124367943250 8.63857762249495841900
H 12.15765682461127106251 19.59625156678983515235 6.65342413851782410461
H 10.93362141030202394631 20.83980276336605186316 11.07664283246061387445
C 8.32602005226776142877 13.52202697727760671853 5.17081771312835147825

C 8.93461838663534813065 12.66200678519962430357 4.23679397193242479602
C 8.71699800261998269946 14.85136172637119145179 5.25298417513186244321
C 9.97342954562924788320 13.16591426258353081380 3.41756127138694365541
C 8.52720297228635359943 11.31052834615272217889 4.06558113999732384514
C 9.76113419453473163401 15.33209560718216124542 4.48982110814437263002
H 8.23735753081543364829 15.49971216982068078494 5.96920632198046519079
C 10.56329919584480769856 12.33787566862377182986 2.43287703586543324619
C 10.42093272182903973544 14.49995398002344337840 3.57890710534886213878
C 9.07909580180721142995 10.52117514727474656411 3.10784255625814243018
H 7.75541259703327323649 10.92250854692940364998 4.71350795281487400246
H 10.09341157613725314945 16.34859502586149915260 4.62175229347591187690
C 10.09841318440165025550 11.01187840047069421701 2.24560612008789073357
C 11.61790654173250203485 12.83420751903270051741 1.62546321250951253390
C 11.51471015227449434803 14.95437459093787424536 2.79433667720846390381
H 8.75991293162113926485 9.49636253822688303217 2.98462813950287753073
C 10.66068178211225081498 10.22865419653354024376 1.23089230937819915646
C 12.15501605657034112085 12.01179853153383625397 0.62889991136113165826
C 12.09218483111019182275 14.15586365712680816387 1.85947698120996007809
H 11.87498749060153713231 15.95684930801978573811 2.96400040687015753349
C 11.66886245366952401525 10.73165796583290543253 0.42945490553564025227
H 10.30331706649705303391 9.22028362171977455830 1.08525073881967148992
H 12.95530980682032939910 12.38801310229265872920 0.00878751894145914265
H 12.91945004312851708050 14.51219644688405630006 1.26252649772401359662
H 12.08736985427104215773 10.11785146533930124235 -0.35399393285223440531

IDPi-4$^-$ (lowest energy GFN2-xTB conformer, Round 1 parameters)
S 14.42165751528646921997 12.13891287895028803234 8.19120639154404095450
S 10.29719634315665999225 14.32086206788109272736 8.88800946718102657940
P 12.22514840963760818227 11.44975209188495846035 9.81400705389124894396
P 9.56652296264805812598 11.63224728217847925293 8.79988699296734289135
F 16.52686670054892914550 11.60638463612366422240 6.23637263147424292242
F 16.25924329278170432644 10.67475755494481504115 3.74271365504888953168
F 14.90155514442998629931 9.09156936988058284044 2.16459810261772167550
F 12.11187933489438073309 10.25950658082646782532 7.20414567108246295390
F 11.39751243191103391439 12.66330411056155469396 6.55058100859898750912
F 13.40561137158347193576 13.24940530179932274280 4.99563127386426586440
F 15.81336314446058466388 16.21956420088000072610 5.74415860815246670512
F 11.62559545778498737434 16.95922044706525610991 8.51135101723492937253
O 13.14757514720123943164 10.05429727815812235292 9.76399002943972860180
O 12.42580834048547444581 11.88395032850179333650 11.38998195866266627263
O 14.88896242535913572169 13.40743118385008614268 7.55241542136161392307
O 15.40948403954978651598 11.59460126157469517238 9.16037477809669198336
O 8.16920462108924105848 11.38306184707106893939 9.66851399571182668069
O 9.25332739591869213314 10.62931118057156787415 7.49989959752167667517

O 9.42451573295802802477 15.50230720095033376538 9.11973855042904446577
O 11.05296832981105659144 13.91316589353745136748 10.10763239663874735186
N 10.71826482810531011580 10.97344709352052127826 9.67100134869977168250
N 12.93409115977446610657 12.41920491909558244004 8.74203325826019650435
N 9.41063245943904647106 13.12650161479830757116 8.26469570489107496769
C 12.99174611001451040693 9.17431856302979298334 10.79857907698382746275
C 13.52826610083779890203 9.44735634479817676379 12.03961550334829055942
C 13.30956069435093347408 8.53604031506071159185 13.10842164816929589222
C 13.73588745028956914496 8.80388963976784388876 14.42779966719911577400
H 14.26031499541113589657 9.72615829090669770096 14.62817298254166153981
C 13.48243123176680491326 7.92208777558984156286 15.44181117614902021273
H 13.81110564426300335583 8.14380902550099783355 16.44697174922352544968
C 12.78885285046378150753 6.72892871555759519708 15.19104879817858666513
H 12.60029005056712492205 6.04141351324124542543 16.00312190685936997170
C 12.34857648053802314791 6.44743085037163243811 13.92861806758772402759
H 11.80283241604288235749 5.53635511178140582444 13.72579935097128611687
C 12.58707200894069977437 7.33854510464528164704 12.86139339208932419467
C 12.13431431650431591152 7.06211694589640082143 11.55566258832630666120
H 11.62867303142828312446 6.12645602610755179995 11.35983538515543322944
C 12.33542878191403602273 7.95028154099927153453 10.53113938075748023948
C 13.72283560910194566418 11.88836211595832992316 11.85750271641227193697
C 14.30956232818599538348 10.68826757837168983656 12.20576582210756555469
C 15.66313676788551312313 10.65736649289459059275 12.63713736428211475982
C 16.36490069318411499921 9.45572210332638363184 12.86975654080927533585
H 15.86555926930316132940 8.51278070439344780596 12.70419712934413425387
C 17.66789713555636609499 9.47226755126417785391 13.28401538070235154976
H 18.19338079424156973118 8.54312395586138606518 13.45379724967500578714
C 18.33678547519214774297 10.68885012929175104546 13.48328305695675766174
H 19.36453718927197797939 10.68299640355534663172 13.81773977823755927830
C 17.69504328546010540890 11.86870264049679590812 13.23392756821355220609
H 18.20765292068358576216 12.81222241058308952688 13.35700452088376799509
C 16.35551659702161231280 11.88592021784909746884 12.79148279229902840370
C 15.69994829042856920864 13.09040702408174716709 12.48159467616794771061
H 16.23145308364927075218 14.02194013780781389755 12.61616712547832719338
C 14.41544241502454859472 13.11533598299220315653 11.99185567378166084040
C 14.30122114086933748922 11.05564498263686346036 6.77538002232024183513
C 15.36783191653418789713 11.08553625201531112054 5.87489155195608070414
C 15.22996201411102923373 10.59372826799116218410 4.58241384589671962857
C 14.01986562419688731040 10.05234386871789098450 4.15216960059360395974
C 13.84084820004216176415 9.70983537524797313267 2.69138777143039886752
C 12.98683212185408208938 9.94126909953067361414 5.07509325914137754410
C 13.13364921403650242837 10.42157072095663927769 6.37789961674295913241
C 7.91212801490647521518 10.07687137716531644571 9.98054164088304496261
C 7.32923454862438727986 9.27430667175642575728 9.01931424695874461861

C 7.08548419133072560072 7.90254965102981543623 9.29476260368646656218
C 6.62085599021013937460 6.99855089566889176922 8.31576054085645388625
H 6.43672985863016311470 7.36210311777214432283 7.31564905077573524039
C 6.41201979990293846612 5.68141756104120432269 8.61897320502982999813
H 6.06034652828931541535 4.99925457887488278175 7.85831912141275434891
C 6.65638062350339687612 5.20219174685695051608 9.91448742336407029541
H 6.47816221855057339241 4.16028558584412788690 10.13886060960061996639
C 7.12907407741125709322 6.04716844984996093615 10.87855555508359906014
H 7.33825136673263056508 5.68385101868343500797 11.87507066155833435062
C 7.37053614630881881453 7.40845625724126932710 10.59559810255471035134
C 7.91362868462294422045 8.27689978608171550434 11.56203452957508659438
H 8.11423503479551300188 7.88850474594666462025 12.55097368024077830739
C 8.21131899206361026700 9.58963225955571907377 11.27785012151882071407
C 7.96274219576960007316 10.59857938269195010150 7.03643225780418557491
C 6.98671430213882249660 9.90146283522532755228 7.72767413796985547236
C 5.65010790958576158971 9.87458275928103290653 7.23984362806893777531
C 4.58211749608472285189 9.30977568377178599235 7.97094668378511705953
H 4.77465890559570560470 8.89682330892981987347 8.94907530818547947149
C 3.31230850851204206009 9.29357610207117446066 7.46430803095463701879
H 2.50717498304353458849 8.85878672529620558862 8.03904014683913459294
C 3.03936833712171505795 9.84448687768349195437 6.20374985457393890442
H 2.03190490245880761577 9.81343831923506471071 5.81429753770448343175
C 4.04237538747220437330 10.43473623763562407873 5.48822453507713259313
H 3.84220566168783861372 10.88806709959058416359 4.52757538579751628305
C 5.35935484059147260183 10.48096014690940513958 5.99046647497282602757
C 6.38879571167454063385 11.14608447545223768316 5.29815457202844619644
H 6.16208418960614601190 11.62356810488292602201 4.35492923631205108848
C 7.65636960581539227633 11.23883542100497834326 5.81049991336728233193
C 11.48567997561132969508 14.76572486912334269960 7.65532640476010506347
C 11.94105587560192205387 13.86591884974419919274 6.70417954370542723552
C 12.99375987326923187481 14.18740449265619751884 5.85276224231724118141
C 13.58655487964503727483 15.43939153542213382764 5.88986153574401605226
C 14.76498912140971597751 15.82001777950856613586 5.02534971531038632264
C 13.08127408542048719653 16.37117650034728555397 6.79032386226336459600
C 12.05865193759294484721 16.03544413958467274028 7.67293128996523154939
F 13.56903653389908548377 17.60850786450568605801 6.79875337762537501618
F 15.18750380926233845003 14.83819023644788970273 4.23190888963874378703
F 14.46466775497016854501 16.84856997094517794267 4.20701195562792484850
F 12.78915088424137813661 8.93067133569850568620 2.44182953717038131458
F 13.65793611839753474158 10.82499054215518974331 1.96217322744500566323
F 11.82310067087287919207 9.40877660669091930856 4.72555781311783285048
C 8.89652922017410929811 10.43286518275947649670 12.26824648840562304031
C 8.49534670620573706401 11.74515076966198812158 12.57807645584940381411
C 10.00974255505528809351 9.88873843676870833974 12.90674781460840314651

C	9.28148884698650356029	12.51042699240407607419	13.47747939902903802079
C	7.30475016724591608153	12.33705118798818922699	12.05973216037605943995
C	10.73832083638258794167	10.60977874386524355543	13.82553550454923652069
H	10.31643269793474360085	8.88283340661793907600	12.65862588776210451158
C	8.94297831340427862301	13.85771015921062421228	13.75292901938723311162
C	10.40498556375973038257	11.93196752757885370499	14.11551581752083706078
C	6.99930006022463579995	13.63336126026404571121	12.29814749295003117879
H	6.64092866531783254658	11.72971851038924739896	11.46338006386054075847
H	11.58608869367162341746	10.15822927875830927746	14.31947692292453311325
C	7.82014191255687673987	14.45033815138043031823	13.12677539910517054977
C	9.72604762164031022564	14.62131996868311922810	14.65430075107968121984
C	11.16093237073640942469	12.71252197262704441982	15.03724571070860882571
H	6.10961449553677482527	14.07409417569501997036	11.87091544784223628994
C	7.52929104591648012956	15.79200425649326788857	13.37612333002820186323
C	9.39315140334811360390	15.95817338404093810311	14.88129020447152939255
C	10.83720871142309860602	14.00074749241513316633	15.29665555189524894786
H	12.01294609842960880997	12.24760169571866974536	15.51511939381675908578
C	8.31357222793340433498	16.53570010459669603620	14.23861518263922221195
H	6.68263516897743947709	16.24745021632863739569	12.88288310494902155767
H	9.99934687048022219358	16.54354638605607874524	15.55922793849885898965
H	11.42386628506852019882	14.59199000999078954521	15.98657117640617997267
H	8.07903149050952684718	17.57635379505278550027	14.41554755788461328336
C	11.92558993093167352129	7.63243571619331540035	9.15356413889650966098
C	12.88809873515217852002	7.51270369801649540165	8.13558850481538264887
C	10.58348594650444240983	7.41764626994597708887	8.86917853152173663034
C	12.46761445323012296171	7.13260691616975250895	6.83624502490964314205
C	14.28023359002877867852	7.73724195553033755601	8.35840722634681476677
C	10.16878323947206119726	7.07545410805223973227	7.59897644815105177685
H	9.85873309540230380321	7.53395462567594798031	9.66051362848165773300
C	13.42111242503393953029	6.96817968841528578849	5.80033658480608860231
C	11.09429402981442791543	6.90815084631792242220	6.56861919555146211991
C	15.18333180878316923668	7.62761634976787128437	7.35643638592309390134
H	14.61134083121298132824	8.01413776361978058560	9.34706346903718987562
H	9.11814721884409351560	6.92174015735774617042	7.39959128655477549330
C	14.79269688166040985777	7.22738904533895354376	6.04598795973352043376
C	13.00816172719738972319	6.54029648445895617215	4.51341871866084520803
C	10.70600720001120365055	6.50653198158461876233	5.25729392517712224020
H	16.23057415135392744787	7.82171859109092348916	7.54044599840637452814
C	15.71484841854730163391	7.05993403754361459335	5.01136739539001396793
C	13.96925129513605945419	6.36156728263562598613	3.51733107873796457810
C	11.61967728369421770651	6.31775547642002166526	4.27794307371637572146
H	9.65325175754746567236	6.34961912500960323769	5.06901680190169123819
C	15.30475227259863224560	6.62185542852483344234	3.76589772859910754121
H	16.75890702022301326224	7.26642752408604941650	5.19854704688396829226

H 13.65557510001056051863 6.02702863099030228256 2.53910465935645257574
H 11.31761148714696396667 6.00836229387139475477 3.28780334332295209876
H 16.03190384768897658319 6.49016212978499584807 2.97834743174718230563
C 13.76264000958041222589 14.39640971181126616329 11.68259405359065361552
C 14.37414534642858576774 15.34425710334858905526 10.84138938707974908482
C 12.55484875708644487702 14.69769457031372184019 12.30229905340341645115
C 13.78132432792742534389 16.62619446159222746928 10.71221271391956442187
C 15.54946174087353760740 15.06147683240305212848 10.08331007350353480945
C 11.96998478242257668569 15.93799807605231499963 12.16565008644347400946
H 12.08051793085402536576 13.94453807865127892285 12.90897645244024438682
C 14.39597089397290474722 17.61510798017654622072 9.90453189358730767822
C 12.57792543440898747065 16.92907379064377693112 11.39643444842892883173
C 16.11633448571172166908 15.99694284685335077256 9.28595650890698465219
H 15.97020532739158049651 14.06833976337311575833 10.13016507852242042986
H 11.03014912731677910074 16.14804088061404030441 12.65346160469644409829
C 15.57870641334583083903 17.31207536951224312816 9.18589680435308686413
C 13.82770035233254013463 18.91010684502390759576 9.80673480581621959118
C 12.02366674445631211654 18.23568596065353020208 11.27243214612943766895
H 16.98803702556777039945 15.75547868699902842593 8.69641553679168310964
C 16.16976249054596337373 18.30141737689017134016 8.39796951234553468169
C 14.45853501380982031321 19.86940832801139578123 9.01396220229962885639
C 12.62417635697888051993 19.18557788831061827750 10.51985195012303897499
H 11.10071732162294644297 18.44187457147429398674 11.79547803830467778141
C 15.61462883942959400940 19.56444117715137664959 8.31916330557234573462
H 17.06360093407242217722 18.06164681372248992375 7.84075617206120512037
H 14.02750138386938516533 20.85874288291688927188 8.94250636978495272444
H 12.19690174407529781320 20.17533694317599213264 10.43132785077899171711
H 16.08490598782849190229 20.31947097785365841105 7.70431048916038907493
C 8.68793967995691751582 11.99894527904371166471 5.07080381159234150346
C 8.78972292833274870816 13.39000266545032324927 5.21966535023889210976
C 9.53004657133826249549 11.32871399432872117075 4.19368807703837465084
C 9.79056214533211743856 14.08551162157081115822 4.49543819105568065453
C 7.94536393516909722479 14.13012749899154485433 6.09985757851184029477
C 10.47068897797141850958 12.01096158265655766684 3.44729495891758341841
H 9.44071033028570205659 10.25670788032045166460 4.09608151266613695896
C 9.98404318628531051161 15.47254934006964433024 4.70883137233151582990
C 10.62419961840837245859 13.39073336369424360726 3.58369204555819687030
C 8.13645906397485596528 15.45263288626465580933 6.30932925913622177916
H 7.16450419311418951196 13.60033909250836003935 6.62284929345437145543
H 11.10401646047860424460 11.47721025278928053126 2.75338735511174448334
C 9.18138310058290407767 16.16313029800973311012 5.64989178958019522270
C 11.00319844618171316597 16.16508903892315274220 4.00825894981724939470
C 11.60584496826304246042 14.12271866021258048818 2.85211853494511924367
H 7.51924668630124859448 15.99396144309257827842 7.00888024864211001130

C 9.44509057055278766768 17.50946208451441066245 5.90680307020814510111
C 11.22121242482435299337 17.51548612356589273986 4.28630622228252722294
C 11.78593491976390161824 15.44870729058068548056 3.05387584353145236093
H 12.21873798321622750507 13.58132732082693117093 2.14533748808291324650
C 10.45880803616557841451 18.17007418022220477383 5.23717085849178065615
H 8.85454968158378363796 18.02383035388873011584 6.64968133904212432839
H 12.00597713938814337098 18.04028320699723053622 3.76117239423883287230
H 12.53876418592416541742 15.99445643885225898373 2.50314628313638909418
H 10.65802960238035090867 19.20897804172916423227 5.45628102801820791257

IDPi-4$^-$ (lowest energy GFN2-xTB conformer, Round 2 parameters)
S 14.50025684046652862946 11.81635534862668102107 8.49160273647816410403
S 10.49269144034935585807 14.28003458575470396852 8.15031074266861033095
P 12.09495608370120400821 11.67496803582829656420 9.95498843579200887177
P 9.56552882744504096024 11.69604724899308756392 8.61876467052061912000
F 16.74877566217694990769 10.69022817506414213540 7.01885339992483991978
F 16.67174591834949382019 9.18630108796675237670 4.81109711973685705289
F 14.28039892426261836533 9.02342532280954223722 2.73667692750208235708
F 12.15817504853189312541 9.86546981880120910091 7.72870651351674720075
F 12.03414094338162421138 16.67972549789243430496 7.27979536984763520024
F 14.18027359043586699272 16.77667612830988730366 5.70323687466615680819
F 15.27921041134482749158 15.34451403803698177342 3.50604126760523238104
F 11.67858620032169625347 12.04020763198491472679 6.42590945959052373126
O 12.90672312891682871339 10.29348752225627805501 10.35123683737716859810
O 12.15890241318824394057 12.48373958349621482000 11.36007995796420999568
O 15.14104813200002652707 12.83751139624707704456 7.63590971251356709359
O 15.32077716992957938658 11.48640487124920817052 9.66727137444630457708
O 8.09504461805652830719 11.75070173471250001285 9.34472936210971738547
O 9.32643693900168102573 10.43581483369685081186 7.58791395885809105692
O 9.71827989799356650735 15.52314054054481395895 8.01625546055514703880
O 11.09154207255803292753 14.12506085980410297509 9.48991484961244324836
N 10.57947351265932844910 11.22431979388813161336 9.75366945460684675595
N 12.98904246461718337002 12.31098021686642418615 8.76264403754604614960
N 9.57130081726102943662 13.02718043535607250760 7.72426993413653661946
C 12.57670215984343897730 9.70066613407163202965 11.54226044635361425605
C 12.99498665398852637054 10.25171825813745307698 12.73510173670639744614
C 12.59518138779777629566 9.64989209423714022762 13.95944505210472108558
C 12.89627718670030276371 10.22072446546630963837 15.21552559594235809470
H 13.46444671705021889352 11.13825851385358411960 15.24751211976450093744
C 12.46960657791377080628 9.63466239534935375843 16.37509835130930824221
H 12.70423191437984122842 10.08631149131066706559 17.32823569627606374866
C 11.71890133309806003581 8.45026594772459382909 16.34000897046743361329
H 11.39287341352774518555 7.99843671984157555954 17.26586928355389716216
C 11.39689310710325642617 7.88117686613981582866 15.14031297785516727572

H	10.80900720127903369416	6.97465329679842405142	15.10058018872122076459
C	11.81532471251224869491	8.46347180308746516175	13.92544159052022934020
C	11.48688113073667871333	7.88902905156163125611	12.68114571727599226847
H	10.93625938649436690753	6.95857603065142171772	12.65895656020856563373
C	11.86419978935121477548	8.48023132153381276055	11.50304866922364688264
C	13.39410636052237713045	12.55214537398595275874	11.97802312392809831465
C	13.84550965367043495746	11.45651947199148068535	12.68519960628270482061
C	15.13833664386001309765	11.47437357761788057076	13.27551087942218366322
C	15.71782453913914601173	10.34088177605439540230	13.88375629094904795124
H	15.16949973619541047754	9.41085491213357272500	13.89639247646363706679
C	16.96595459230497837666	10.40267407913150776722	14.43882878593720775484
H	17.39816956003958026145	9.52439239047430596941	14.89710924491724419738
C	17.69967760915445964542	11.59771073521772954962	14.41146608555865782364
H	18.68182938590357622388	11.63008285988801837618	14.86140871202573343623
C	17.18001578495018932813	12.70304262035481812632	13.79972699847665040807
H	17.74638114910966990578	13.62204449519070514896	13.74717720926780906154
C	15.90135846174004718989	12.66821281151716327429	13.20447977730080246772
C	15.37620429670728761096	13.78213207326333034075	12.52633098261762611969
H	15.95877664951461483156	14.69188162857727597554	12.49015418494687423845
C	14.15792305548699658857	13.73962920548886934569	11.88982248627583615530
C	14.43732795470611485200	10.42486604083813794830	7.40068819971285307702
C	15.59175721344383092060	10.16385962771491513479	6.65179463540393456356
C	15.55408354278750593380	9.37385627050769976165	5.51015203901176242596
C	14.35697152248336649905	8.80658526189039037035	5.07633455335372563155
C	14.30536440426161881589	8.11944383081669940339	3.73240089312006118050
C	13.22725447424098632609	8.98460273434918832436	5.86567439113477018253
C	13.27358997673959883912	9.76669044191199731131	7.02072884099345895237
C	7.70047463428179401745	10.58237268937158148674	9.94389233587381937696
C	7.16304065283360102967	9.58877106303230064555	9.15018809319200698837
C	6.79202177479906765711	8.34512633705110395965	9.72770841235878336306
C	6.36751403497361501138	7.24211784678123304815	8.95665117615678951779
H	6.31491451369073786282	7.34579812959207778533	7.88301332272349952746
C	6.03373528329219066535	6.05664913796283954639	9.55135151385494474141
H	5.71480075136116560941	5.21864239636480586881	8.94838578800364281562
C	6.10668139835382639546	5.91448801462453310762	10.94496320325635885240
H	5.83142542836803112749	4.97333927985548029937	11.39894997657774133870
C	6.53594966397109367051	6.95658347737483229167	11.71740866707129136159
H	6.61325211398107448701	6.85144233737921037886	12.79058014075558880052
C	6.90335792887099231052	8.18844766563662673775	11.13513492896260714815
C	7.40334203399602230888	9.25093535311635584151	11.91256096103611028525
H	7.46991405083052217861	9.12040805840822166317	12.98377159503391276019
C	7.82417372263325994197	10.43285384908666735271	11.34750218302986901620
C	8.09064012798222975675	10.33854521525823599859	6.99055648481098579339
C	7.00190473237715682586	9.88159747849375769135	7.71218085435406308648

C 5.72390631416792761144 9.79023528219405037021 7.09212011170635125978
C 4.54830706720711042124 9.47606257783077410295 7.80860783746504250047
H 4.60852876174644343621 9.31629708875775364163 8.87401903322506235838
C 3.33977707494783571107 9.38749293961939201836 7.17531782865476497335
H 2.45044924599268343357 9.14797127461453918329 7.74036754714719954507
C 3.23879753268790304688 9.61259886495117754635 5.79469604268928506485
H 2.27736978006959134291 9.52787494801630430175 5.30884643013465495898
C 4.35015415325920873357 9.95818274521687385459 5.07925732607769653271
H 4.28344179862906759837 10.16178765552290386154 4.01980472918439968311
C 5.60807707517349030013 10.07268733194817755816 5.70649443135095868485
C 6.74873831243287014559 10.49519304622501358892 4.99818137689659458545
H 6.65573928106776513403 10.72746409445844584241 3.94635075746418628739
C 7.96034909502228593681 10.65927094324631774214 5.61762677111940611496
C 11.80927156555372015134 14.34487254179532556009 6.99594038664595085919
C 12.47655470640938268900 15.54856038267840467881 6.75729338277338165142
C 13.60504941880185292291 15.60105661386199393803 5.94461741749300909277
C 14.12902645559361047845 14.44910680435022243273 5.36787692031924379421
C 15.41435874073266809603 14.54194855895724991512 4.58110516894653585496
C 13.44876810126131694290 13.25902417828557489088 5.57140284563516452465
C 12.29684021556128570296 13.21428815623322527983 6.35047508450242403910
F 13.87244077299320998975 12.11606820110480597918 5.02333809095513039722
F 16.41188553901451641082 15.05862148974537007007 5.29820868763898111808
F 15.83974917460042419748 13.37261486539460086931 4.10780065463363186495
F 15.36636905144799847278 7.34110886280313224006 3.50002110955386758206
F 13.23075467455111109416 7.35058976042019729391 3.55709301963864099605
F 12.06758673685691896083 8.44121979564300772836 5.51638848766347589248
C 8.46104030260836381672 11.47192049200045360635 12.16972032464802921936
C 8.12880792844034871791 12.83652014150048970009 12.08746059546841600252
C 9.45619859366598447536 11.06212229103411637254 13.05552617765093614821
C 8.87119304701248800882 13.77249178747112523524 12.85258562287106443023
C 7.04706378927336363205 13.32741067497429732214 11.29654346034967993262
C 10.13541355342135830142 11.96297873760688723621 13.84423953311861765769
H 9.71117440277975241258 10.01353983539106096146 13.10816469400229244968
C 8.60871729399384122416 15.15918196069873324916 12.73458436544161465065
C 9.87384567269658575128 13.32893998123096857000 13.74811298507587942197
C 6.81833975765767341670 14.65379576591808863384 11.15873617953506524714
H 6.40385306250111518978 12.61697248718557595737 10.79970193228563246635
H 10.88975716142450700374 11.61686478053163718016 14.53549773003491019097
C 7.60743298298433323623 15.61958908233525811227 11.84605122835902868417
C 9.34699892377953389655 16.09410770379248134532 13.50246238964449574382
C 10.58431313986024235874 14.28675108809446925306 14.52799410305665617216
H 6.01444845154140583787 15.00862979607917324643 10.52934151823989594732
C 7.39509993493181561064 16.99185927188858968861 11.70969243256606162618
C 9.09461207182319952835 17.45787188831605618589 13.34113270566145992291

C 10.33301061433788881061 15.61155630460756604805 14.41206173271609358721
H 11.34303581097283242229 13.92354230472660425733 15.20832800272720852774
C 8.13723398008570519835 17.89741045694264087729 12.44541666003125435225
H 6.64322508677500422891 17.34180101642476046209 11.01708227784268778748
H 9.66772781095555089337 18.17087990350612614066 13.91811278781628402612
H 10.88581321427459158713 16.33423151954265861718 14.99674731766236313035
H 7.96533030887705173484 18.95792533474742214139 12.32289160423412255341
C 11.58311813922987809633 7.84141243220881367648 10.20717400461362167619
C 12.63834616629969787027 7.42014053163553377601 9.37906469188862068620
C 10.26727274482860607918 7.62260482692793583936 9.82150131537177806251
C 12.33337690135962994020 6.74001583159073991425 8.17324040915424454568
C 14.01116006955924042643 7.62940417195966169572 9.71042631554391988402
C 9.96777287030150560554 6.98599389246216251337 8.63512107764499958762
H 9.47249823065946117140 7.97118908904697232032 10.46313536923544162960
C 13.37822814438227503331 6.27348824636301838353 7.33666979099827010202
C 10.98403032979644144973 6.51811396841976975480 7.80167906097993757442
C 15.00656933349144139811 7.22571146945307152265 8.88713405532012856725
H 14.25307625613987738689 8.13353683589396858622 10.63303728477585430312
H 8.93625061688632627011 6.83483067104486163856 8.35154893436938117190
C 14.73081789488551684997 6.52360176361604082729 7.67835613824699159125
C 13.07581857310002071415 5.55209847695169678161 6.15436843531775323868
C 10.71072800797888113777 5.81340257342167543442 6.59321831685014103641
H 16.03945089498512288628 7.41156545310477632427 9.14572214976850617063
C 15.74417821500045278071 6.05727412270470289002 6.83904242948812601810
C 14.12276451647577246717 5.08336991671212601318 5.35936643315853089575
C 11.70814235610396458753 5.34027726497036248077 5.81164416518444415516
H 9.67572624508242462582 5.66184939602780623602 6.32087164673659351877
C 15.43931696571214473579 5.33735094146732436116 5.69884824403279122151
H 16.77441289030770832369 6.25702439509826291442 7.09676237168388457377
H 13.89240238506115865391 4.52674854649413127561 4.46250913750204514230
H 11.49247000936327900433 4.80336920629063879318 4.89920268541187642342
H 16.23529732127761349147 4.97804962377263304774 5.06353767259628995134
C 13.64228083489811460538 14.92934507470576122046 11.19547931852945588105
C 14.40942553061750608379 15.59863610439350978254 10.22432297102641918229
C 12.40495975538901518576 15.43927703929177752684 11.57308413501115218480
C 13.93454493607448974046 16.83203625596125263542 9.70960156111415351177
C 15.63233093395480288734 15.07433309973320945119 9.70862953137554463012
C 11.93491198282801235564 16.62962476197069605632 11.06172012693935080563
H 11.81404844821304322977 14.89171693445643818166 12.28826040618628567813
C 14.70431799677801265602 17.54930731215784334154 8.76038715496214059897
C 12.69403554271829293043 17.35883596102448578335 10.14747536999406030134
C 16.34968053592442416289 15.74542949684365567009 8.77725971095195411920
H 15.96905212258525885716 14.10821846691516334715 10.05379929343334133307
H 10.96933574019346124828 17.00423840428247856948 11.36637279573923819953

C 15.92799466531286434190 17.01464982826970384622 8.28728595582443716694
C 14.25111867618498528998 18.80158785033799517805 8.27477524351770554745
C 12.25876758233230745532 18.61543408779779085194 9.63621735611708274405
H 17.25718336356509041707 15.31929112920145996668 8.37652746917011015171
C 16.67166520294991371998 17.73785382353019102197 7.35304057057563031918
C 15.03245476689998483266 19.49265573396309747523 7.34816640927010400475
C 13.00559660409722084751 19.30952726693919530021 8.74755473466339594779
H 11.30568600205975826611 18.99394546421388696444 9.97722086367975791177
C 16.22673027565687675633 18.96323179149795379317 6.89466131421080419273
H 17.59686124517856598004 17.31961004957128480441 6.98429457744378101580
H 14.68880746354578370472 20.44988450776772381801 6.98034071880475437410
H 12.66790433824712813760 20.26301024399955608146 8.36432555293151125397
H 16.81457170413674262477 19.51057025575136449902 6.17068666759832762381
C 9.11407274357868324444 11.16044570767356702845 4.83845314189903596969
C 9.30344538524153641390 12.53782055754081703469 4.65272497012860508647
C 9.98716537874078724712 10.25207191255753436110 4.25505418254171097203
C 10.42126659863436977105 12.98066465276613712376 3.90146929634790851082
C 8.43278238785476119688 13.51470873030973329776 5.22137430545328662390
C 11.04578401881266280782 10.67939332993369205838 3.47787212232832265713
H 9.82877949698397834766 9.19542356856886478056 4.41348132424544914443
C 10.69645114028058863198 14.36594461106780329374 3.79106050945821326792
C 11.28751399851724279699 12.04028100240484455696 3.28971764659897525362
C 8.70231049611196816329 14.83680490348151792546 5.12423194884149513229
H 7.56386451106551760404 13.17129333357529574755 5.76082983908908285997
H 11.70403143766951359339 9.95895927572764882996 3.01412066423660762737
C 9.85889604084629134206 15.30822919296383943788 4.43728184707923833940
C 11.82908521967422821319 14.81086981737552221716 3.06428024995568337729
C 12.38963182533605333902 12.51731433216540168019 2.52001076479712082801
H 8.05888173774635596658 15.56538549263972193160 5.59209985526948738510
C 10.19621898333916654167 16.66172890601092859697 4.38618182879265994245
C 12.11906996940243175231 16.17578835435305251167 3.02716434795222255616
C 12.64715460139577096754 13.84129979497334872462 2.41047564418655646534
H 13.02820134519341266355 11.78709068481783184268 2.04334565770518672423
C 11.31771538536136390007 17.08379173071504908421 3.69583046698291584065
H 9.57535680710125802761 17.37410635893136046093 4.90805171369159953798
H 12.98927656732236357584 16.51369685259399844313 2.48354560259025092250
H 13.49003468407465788914 14.19474284580001111067 1.83386314968770691713
H 11.57166964433336708851 18.13361323362424926131 3.67613538030769992915

RC-1

C -3.98115331837416 -3.07462987384636 -0.14895078974765
C -2.71955216774698 -3.64220653771830 -0.26766136189135
C -1.46006201031194 -3.04284403910834 -0.01297700979566
C -4.30146470331225 -1.74995352627695 0.24584877018965

C	-1.15415180746620	-1.75124993879805	0.38699661448013
C	-3.46583498530777	-0.70167464011651	0.58556784605568
C	-2.03544889678968	-0.65016407009441	0.63606458733000
H	-4.83780466053919	-3.72561987064045	-0.38609640906319
H	-2.68612226489898	-4.69590003647102	-0.58859120794530
H	-0.58119871263034	-3.68853275052124	-0.16237689026685
H	-5.37604277858823	-1.50580361739844	0.28162213693594
H	-0.09115241080665	-1.53097022581857	0.53965262340220
H	-3.94254484828793	0.25438511111307	0.84487431568632
O	-1.54414338419041	0.51974689709521	0.95108369117400
H	-0.50553326524845	0.57170282252142	0.81541757264037
S	5.53485295692700	2.42170359884670	1.37494175148797
S	1.09578833213482	0.46073832807559	-1.11070329051022
P	2.81348841210491	3.37447405828833	1.20851742073604
P	1.15537187988699	3.33108420764591	-1.31750456838567
F	4.55613296817176	4.42428278057833	-0.96393688213347
F	5.94260312278566	5.18461247221459	-3.03797075564456
F	10.00573511682959	4.83846260034793	-3.23744772110809
F	8.46135326122286	2.39387111590096	0.90741605150506
F	2.00824364792328	-2.33670670063518	-0.61248514782375
F	4.49057120521017	-3.29229029312930	-0.46196496072994
F	7.04393241349437	-2.81124136983132	-1.66418198619139
F	3.78116296024319	1.92121532942745	-1.89135170017268
O	3.50865543105732	4.76228316966875	1.77195943340792
O	1.90356246450832	2.90812413049485	2.51643068826824
O	6.07676429368993	1.04254294190580	1.27855267479975
O	5.75598945769364	3.17800683655026	2.62710296511613
O	-0.39056197057683	3.91390786736636	-1.44004676429768
O	1.92758542436454	4.21258502005328	-2.48283903260532
O	0.20954436761527	-0.54463954276077	-1.73930624225582
O	0.92331959007825	0.60280219864382	0.40164418456079
N	1.73742989631716	3.71391712565845	0.09284909862658
N	3.99743177530907	2.35967371451921	0.86408555930102
N	1.00759611214574	1.83006961679648	-1.88373400959192
C	2.80166446413988	5.70213721576776	2.48516577575897
C	2.33296260344885	5.39603729301659	3.76576068628540
C	1.47841838753893	6.35172807436160	4.42089910624576
C	0.83400021122021	6.07885260326180	5.66423963290531
H	1.02163571347121	5.11510254536293	6.15892346423548
C	-0.02630060410125	7.00178604867535	6.24204004529648
H	-0.51950457418773	6.76406578800084	7.19661382026640
C	-0.27702360450638	8.24826073413632	5.61005136371829
H	-0.95826284545378	8.97386953636497	6.07920276590882
C	0.33358903377027	8.54514190216912	4.40147041524617

H	0.14434541439222	9.50736280363857	3.90048296351751
C	1.21372327207337	7.61523604120612	3.77309308428646
C	1.84430651598548	7.91403902562997	2.53104297977813
H	1.70213485232929	8.90987303797075	2.08376365883089
C	2.65208792150783	6.98783877325827	1.87930660098484
C	2.55427081252613	2.90293942496471	3.74684952528850
C	2.76294338364572	4.12148560185386	4.40196705486580
C	3.51439369531046	4.13006513056898	5.63136621525125
C	3.89423065892482	5.33141881106213	6.30192472385567
H	3.61085352785043	6.30010644156795	5.86964658411812
C	4.62749321660769	5.29242178443011	7.47895020238344
H	4.91278751002026	6.23385127730519	7.97236565384884
C	5.02238886332767	4.05201715532261	8.04564718847721
H	5.60034948187362	4.03460824707615	8.98184108131437
C	4.69749822670699	2.86830573559713	7.40325406449853
H	5.01969544836234	1.89940752682516	7.81519107782175
C	3.95653006995368	2.87470231423309	6.18470688169638
C	3.67354392795313	1.66810265335298	5.49035244981962
H	4.01957217909838	0.71441242488374	5.91707461059288
C	2.99524166125543	1.65348111625909	4.27643397518235
C	6.43421328506126	3.30269921980980	0.03217356385316
C	5.84266182870887	4.06914473600652	-0.98216038424414
C	6.58910136394558	4.48732477192238	-2.09829663744744
C	7.95246845600880	4.17361723073664	-2.22898015266599
C	8.74163303680672	4.44597014982194	-3.49198043275668
C	8.55499975428496	3.46208974417535	-1.17297526943116
C	7.81814338140832	3.06000613420305	-0.05107756374819
C	-0.56139304587804	5.28875562266503	-1.43005523468744
C	-0.22166257724010	6.02081424083252	-2.57101190327009
C	-0.32044177734240	7.45595983998804	-2.51600687489308
C	0.12584507996397	8.29842776145062	-3.57772546236092
H	0.55533501579788	7.83666306391105	-4.47743374766874
C	0.04009020389152	9.67983057346302	-3.47810602675346
H	0.39988070762047	10.30909944213738	-4.30603225961282
C	-0.49821511619594	10.28830527153647	-2.31340189440023
H	-0.56205887398133	11.38469848277401	-2.24708495134717
C	-0.92889652868505	9.49936233586651	-1.25842149634176
H	-1.33414336955416	9.95945621031720	-0.34378283528698
C	-0.84490193294863	8.07690761985319	-1.32260261659186
C	-1.24667039947962	7.26309515372601	-0.22480166614022
H	-1.66994609064114	7.74194156747379	0.67145110804121
C	-1.11119095494030	5.88017883891445	-0.25381194096900
C	1.29532740070049	4.39161009328284	-3.71040928348856
C	0.22194845129420	5.28855229605811	-3.78845117917791

C	-0.48465656677829	5.42124727060389	-5.03711348056927
C	-1.67363961171709	6.19925886226283	-5.17647917422789
H	-2.07921343904359	6.72013913410002	-4.29873419451750
C	-2.32595710159054	6.29642376502681	-6.39698615680268
H	-3.24312718683009	6.89950219393391	-6.47672068761527
C	-1.82599966947873	5.62006079765765	-7.54105455997175
H	-2.34849575314044	5.71111582208952	-8.50518494435583
C	-0.69084851275800	4.83263651683024	-7.43354620479151
H	-0.30342689994901	4.28438239530570	-8.30630487377861
C	-0.00478995704042	4.70034549757910	-6.18995612221293
C	1.13026516333183	3.85379276743213	-6.05933093930925
H	1.49690346260048	3.31234710639082	-6.94492355387264
C	1.78035523478627	3.66707321429102	-4.84471328628538
C	2.79344046331603	-0.15738927107254	-1.22567363180060
C	3.02145634971576	-1.50507097434401	-0.89158745517650
C	4.32328075882171	-2.00566568745169	-0.80247924457748
C	5.44210109160688	-1.18508745696902	-1.02992731517203
C	6.82474427513727	-1.76859124057388	-0.81815370800924
C	5.21020515369419	0.14312036976932	-1.40938455262594
C	3.90311427908523	0.65188940938717	-1.50956149455597
F	6.20783005779206	0.97801141425555	-1.69678128392519
F	6.94395958161878	-2.25198410581945	0.44200748382917
F	7.80878912777031	-0.88040849806202	-1.01321417336282
F	8.17438092937927	5.38329676668303	-4.27702619744719
F	8.82655030888659	3.30546224605214	-4.24182640056991
F	9.85145737117873	3.13663070430151	-1.22634296046425
C	-1.54268857942414	5.03364373589195	0.89264989966644
C	-2.68181271822158	4.18404038668644	0.79152335520718
C	-0.83481742980304	5.10548067703622	2.10457906060589
C	-3.09867207074334	3.43433151537547	1.94368732329501
C	-3.45705133436880	4.06970669876010	-0.41541120201404
C	-1.22279813229355	4.35927976049528	3.22021510131705
H	0.05053200652534	5.75078046622227	2.16180293030846
C	-4.26441041732384	2.60681988056298	1.87998322059021
C	-2.35662186229710	3.52293687797440	3.16976618017699
C	-4.57263770560385	3.27104126737836	-0.47992441781102
H	-3.14219838225781	4.64135673896792	-1.29993672319619
H	-0.64336944052703	4.43484301579967	4.15159385380923
C	-5.01921540717700	2.51680894414174	0.65958711942431
C	-4.68100674372591	1.85276606301738	3.03245217233779
C	-2.80401916005524	2.77056332225242	4.31082523318346
H	-5.14894103511054	3.19883503240238	-1.41522822697431
C	-6.16236321928067	1.68322058357563	0.61621315270564
C	-5.82400910672856	1.02573116456292	2.93663359357720

C	-3.91736315906791	1.96795225540415	4.24721949864049
H	-2.22842811538507	2.85482552680340	5.24603315221360
C	-6.55592611882969	0.94666606187872	1.74254608252822
H	-6.74041971715449	1.62070033710095	-0.31885975743347
H	-6.13939505189282	0.44850528250411	3.81965192543234
H	-4.24935244651590	1.40014503104165	5.13058045924601
H	-7.45045190502011	0.30681102118646	1.69222786223470
C	3.36663128948358	7.30885077508907	0.61786146821353
C	4.78714406333188	7.18999307437482	0.52125707206722
C	2.63079110100791	7.76420288894618	-0.49149146855206
C	5.43225739413361	7.54786728132903	-0.71289337686474
C	5.61235135282199	6.73081125852859	1.60855123671161
C	3.25533843271858	8.10577968570763	-1.69353438009079
H	1.53715401513918	7.82457988207672	-0.41040889492198
C	6.85504478320635	7.43541249004671	-0.84040578303351
C	4.65647579151953	8.01412487822483	-1.82917706990760
C	6.97104034376236	6.58737839657894	1.47331592608574
H	5.14591788248196	6.46616250757804	2.56586813989263
H	2.64742643662195	8.44548034475618	-2.54530708181677
C	7.64148839744961	6.93302648930270	0.25177756394505
C	7.50271792527515	7.80595231183747	-2.06875940734249
C	5.33168186409850	8.37372682693109	-3.04730585680099
H	7.56591993620612	6.19550538769980	2.31220041100885
C	9.03995788272880	6.79975489119508	0.09334330449080
C	8.90485474863262	7.66941887093669	-2.17327635679322
C	6.69647839718281	8.28071097397183	-3.16168169064005
H	4.72246173151315	8.72725003011581	-3.89375354571348
C	9.66193713854624	7.16679343709588	-1.10620346973796
H	9.63450811404989	6.40189653060778	0.93017217665901
H	9.39641329733941	7.94184769200603	-3.11943313780758
H	7.19960345787395	8.55377617314283	-4.10200956014806
H	10.74995322107789	7.04873891021923	-1.21734279081219
C	2.79579290647198	0.34377359435632	3.59005397856203
C	1.52444198466328	-0.29308267177831	3.54727653571756
C	3.92801413232067	-0.33382273318943	3.09876961144265
C	1.44051281567857	-1.65473329529097	3.09281513009944
C	0.31656555467254	0.35838336921694	3.97643172684967
C	3.83716412331165	-1.63191945574414	2.58789914382669
H	4.89992953860928	0.17704871596691	3.09033146268744
C	0.19139810128603	-2.35201864388433	3.14153753083684
C	2.61103012323706	-2.33061872004019	2.60779213383707
C	-0.89188894576662	-0.29574853862535	3.97863955986633
H	0.36878545214786	1.40990489427335	4.29225988424433
H	4.73813550077880	-2.12111033245969	2.18781569912140

C	-0.99608098528693	-1.67233763640367	3.58311097931503
C	0.11508219111779	-3.73120954344174	2.74075275570625
C	2.49888851834936	-3.69896835161721	2.17850658799258
H	-1.80561499323667	0.23592336810303	4.28001594770816
C	-2.22514862718278	-2.37496842675255	3.60813096363259
C	-1.13198269272647	-4.39557563838288	2.80613578607403
C	1.30644625068407	-4.37683276640454	2.25722499733670
H	3.39883771944198	-4.19413953144686	1.78506884698376
C	-2.28675069660307	-3.72262344158995	3.23384891132754
H	-3.13301943347610	-1.84499337267304	3.93590034659509
H	-1.18536491900002	-5.45561279061477	2.51194735728672
H	1.24210681871213	-5.43010227580938	1.94187176648781
H	-3.24830853241009	-4.25699749773120	3.26923134127980
C	2.96904171189291	2.76646955722840	-4.76722060903706
C	2.82435497784862	1.35134750634067	-4.72582653218090
C	4.25565672464549	3.33498481326068	-4.77939460865470
C	4.00303432924788	0.52914195527980	-4.65613690712895
C	1.53872964961258	0.70665461054639	-4.68842404361714
C	5.40370169743550	2.53754249487576	-4.75968007075010
H	4.35388525993587	4.42908485601284	-4.79979863738335
C	3.88113509716060	-0.88902567324266	-4.49591484882198
C	5.30615694874267	1.13141557380597	-4.68862019108586
C	1.42326818699397	-0.65348335982101	-4.54036682322100
H	0.63751915582219	1.33255130264958	-4.73384695261017
H	6.39905055164398	3.00165082043801	-4.79136621756435
C	2.58136458584656	-1.49400116161479	-4.40952298672916
C	5.05723505493031	-1.70447281782910	-4.36114593036356
C	6.46775167729759	0.28705999387594	-4.59468308318589
H	0.42893287835990	-1.11593913990421	-4.46500524944109
C	2.48346807531164	-2.88252483463460	-4.16520891957049
C	4.90875233882894	-3.09015200436890	-4.12534786861868
C	6.34889750900946	-1.07171990859112	-4.43249166309256
H	7.45881920760281	0.76551564016600	-4.61391685754268
C	3.63561304615538	-3.66688799511150	-4.01938511478021
H	1.48419670541030	-3.33573947656360	-4.07665957771784
H	5.81025935337074	-3.70812711628335	-4.00095677346919
H	7.24546942700962	-1.70084988057321	-4.32548344826727
H	3.54187177005947	-4.74499823085248	-3.81866196933059

RC-2

C	1.08319679726213	-4.33708376632502	-1.50872448483440
C	2.13335788207981	-3.44198970230803	-1.32040143331399
C	2.09285856503977	-2.19586394672006	-0.64809504528171
C	-0.25717202984765	-4.21142402054121	-1.06247439577659

C	1.04477354614382	-1.54849832725154	-0.01092916840428
C	-0.86910537250879	-3.19628673013832	-0.34063409256807
C	-0.31460324785750	-1.97007922118505	0.15822476842355
H	1.31592040325589	-5.25778504263802	-2.06712754491674
H	3.11346618566009	-3.71999992978227	-1.74199347700046
H	3.04073365276403	-1.63663737661957	-0.63942469657524
H	-0.91746893968748	-5.05388804051683	-1.32672490825056
H	1.29756609158866	-0.56680136436763	0.42748977101443
H	-1.93605702353873	-3.31896711816335	-0.09472468818475
O	-1.15428939514299	-1.20988385854542	0.80316805758303
H	-0.69881915007340	-0.36259102431948	1.24793564671616
S	2.87115122265454	6.49815416782903	2.40654660401805
S	0.77298527414353	1.82762312629565	1.73469268106229
P	2.43452229789413	4.95812548324289	0.00391047597162
P	2.96020922152055	2.00892010378413	-0.10370196787110
F	4.49861085531272	7.88911857428452	4.45174181922034
F	6.76816046765343	7.14602210654543	5.60533810600569
F	9.12916877623766	4.10520852575703	4.06786317108133
F	5.08607018135255	4.47193050360622	1.16054270868427
F	-0.97174223257029	2.08419688020161	4.18119298774953
F	-0.43809879930440	3.31149483679114	6.47428858875088
F	1.64626852777924	3.94078421037560	8.15008257551215
F	3.52389573003293	2.89829639424324	2.76265114737952
O	3.46538447053926	6.08335443598649	-0.62762459706053
O	1.03184372519945	5.29851727388120	-0.80182390277889
O	2.24288703558379	6.42941142194457	3.74642541038666
O	2.81328181083744	7.77193369086902	1.65535611204762
O	2.66857765119500	1.04957709074999	-1.43286505349425
O	4.55939784049859	1.63998741761959	0.15957897841642
O	-0.08522486703564	0.63085642958048	2.08028385149175
O	0.11604856667779	2.92108901300542	0.99410748897960
N	2.84360714393071	3.51270581363978	-0.52984519524337
N	2.37408575533843	5.19448368175504	1.58122148277365
N	2.10431053624153	1.25908795957600	1.04503794143363
C	3.43403069053083	6.44773867160297	-1.95457475641024
C	2.33404165329456	7.14517080139498	-2.45872462902802
C	2.28917509423674	7.40936423340888	-3.87388142944763
C	1.15661943666609	8.00309529539619	-4.50792940888541
H	0.29677846212740	8.29792720507815	-3.89031487456597
C	1.12540879085663	8.19899695024166	-5.88158180369000
H	0.23690739918490	8.65150680183057	-6.34719608624663
C	2.22838271154937	7.81778504487289	-6.69013342660421
H	2.19436309081687	7.98161262247055	-7.77769217029650
C	3.34288826460284	7.23546395289075	-6.10664097596705

H	4.20308353067777	6.93179464292894	-6.72362167330946
C	3.40436943970433	7.00890833009481	-4.69966305228918
C	4.54591138856750	6.40745499696959	-4.09543284996278
H	5.42138969978042	6.17201253797958	-4.72015808435910
C	4.59163526109930	6.13516258299521	-2.73273227940919
C	0.60054058375730	6.62178270720466	-0.73902703796848
C	1.25131653922412	7.57200098055585	-1.53069380842602
C	0.90635571289911	8.95987416596730	-1.36598954267762
C	1.60568698666854	10.01456153603417	-2.02403414300721
H	2.44538589400198	9.76741553283797	-2.68781537629508
C	1.25129846347589	11.34102685002301	-1.82358958298651
H	1.81187933475725	12.13745716627616	-2.33606408021020
C	0.17984849958295	11.67961106480517	-0.95571186945928
H	-0.09387267236048	12.73504092015208	-0.80717528286225
C	-0.50528948639669	10.67992800391434	-0.28372722398016
H	-1.32447488000995	10.92911501623582	0.40847210832395
C	-0.15770610271101	9.30738253739770	-0.45659949644859
C	-0.83685477855403	8.28096316639432	0.25375594190291
H	-1.67241314273945	8.55521321932136	0.91537834794530
C	-0.47763904942663	6.94048301026318	0.14318773225973
C	4.64476069669541	6.16347868040088	2.80793972776497
C	5.16538001193318	6.87088011443828	3.90820023299800
C	6.36833844142159	6.48390999104618	4.51355126832632
C	7.12972104990862	5.40964851249071	4.01180852608848
C	8.33051367341970	4.91815092829002	4.79013779708457
C	6.65237099512808	4.76948786567464	2.85606075965872
C	5.43927728949230	5.15008007244019	2.25543521353283
C	3.50400173614718	1.20844038230071	-2.52628135518713
C	4.77714742093864	0.63611626705000	-2.46700557730991
C	5.69363160248234	0.87605570184276	-3.54813320439636
C	7.05166076780993	0.44114885016248	-3.51849032040293
H	7.41566084590982	-0.10664087591557	-2.63790730926333
C	7.91242156026801	0.71912243283745	-4.57107155318504
H	8.95950359889374	0.38433198861531	-4.52272706422888
C	7.45568265347723	1.44201405598947	-5.70503054263904
H	8.14763539231998	1.65589485545885	-6.53322576571018
C	6.14453272282723	1.88818899679535	-5.75954494028384
H	5.78438238131232	2.46330770592245	-6.62659427501113
C	5.23568462089646	1.63157893507508	-4.68987386905607
C	3.90209381670501	2.12827544972817	-4.71704588228644
H	3.56072777946194	2.68459852546485	-5.60338210521501
C	3.01894615010046	1.94109795042123	-3.65571438669280
C	4.97922586750552	0.32869820178591	0.01054376153285
C	5.11867330356818	-0.19863954159370	-1.28288446404525

C	5.46322685345290	-1.59097772684879	-1.43406227708355
C	5.47296854417257	-2.25150211311920	-2.70103223420541
H	5.23545862647703	-1.67619717032344	-3.60570564237600
C	5.76717232928376	-3.60484576387723	-2.80167586785717
H	5.76919800216891	-4.08817566218369	-3.79046217818227
C	6.06467042602502	-4.36923800022671	-1.64167330046420
H	6.30589780409898	-5.43880507958922	-1.73448064975937
C	6.03981461252971	-3.76430515635601	-0.39426627737388
H	6.25197989154562	-4.34714643067291	0.51547257144211
C	5.72920697983229	-2.37858028404156	-0.25489204579621
C	5.63604289866778	-1.76721651296810	1.02627395576690
H	5.84966788915649	-2.36929558921040	1.92266347792287
C	5.25391900560226	-0.43980060352295	1.18846897513753
C	1.23814102666249	2.48077263961271	3.35370703772527
C	0.24510724673510	2.59955844005582	4.34485122710415
C	0.52117628499132	3.26379307002319	5.54706199317994
C	1.76602439935532	3.87358169264036	5.78264841802494
C	1.95638786045323	4.68065173998751	7.05107096714186
C	2.75622475979034	3.73224915580077	4.80090939586968
C	2.50384382735428	3.02772849107687	3.61324304196097
F	3.98142128908030	4.23885676688142	4.95532498342342
F	1.14210384860047	5.76043454257170	7.05904234064674
F	3.21737806024574	5.11664218387174	7.21167699279120
F	7.92145683666103	4.19749000238568	5.87786126866026
F	9.09317663946333	5.92624323544216	5.25803653198400
F	7.32236355107950	3.76018951214031	2.28830452206411
C	1.65672053043021	2.52889035645200	-3.66223153828369
C	0.49389119805164	1.76087169083228	-3.35403826268485
C	1.51754575657017	3.89908637620787	-3.96258658675905
C	-0.77995620322462	2.42060879828386	-3.26775538862713
C	0.52748053587543	0.33572888448161	-3.16075100058312
C	0.27301298579379	4.52947079545170	-3.93646862052870
H	2.41810277278641	4.49020190061412	-4.18218297963952
C	-1.94827532636962	1.68796062216616	-2.88246790228443
C	-0.88964081729887	3.81939147519280	-3.57179991879883
C	-0.59786093000024	-0.37214687414771	-2.81381094615633
H	1.47709178756002	-0.19670845404887	-3.31032323251426
H	0.20159170921141	5.60158525146053	-4.16826316744569
C	-1.86360191444635	0.27897059464665	-2.61778156108793
C	-3.21444574534954	2.35759997752888	-2.76201780298697
C	-2.17583401349483	4.45535546817297	-3.48169836028742
H	-0.54538230814678	-1.46476375280032	-2.69233148419938
C	-3.02057735469952	-0.41658484731715	-2.20068421739057
C	-4.34232347330395	1.62133788684188	-2.33126665168638

C	-3.29075598488714	3.75759488853694	-3.08440067551528
H	-2.24396974600280	5.52803813368408	-3.72019631319041
C	-4.24187452074942	0.25296355212358	-2.04598597265676
H	-2.95098916868415	-1.49749130679845	-2.00188601854804
H	-5.30762067810581	2.14007342322838	-2.22536561586251
H	-4.26430407417310	4.26398951576608	-2.99758919630076
H	-5.13145259158228	-0.30138748119943	-1.71068815241113
C	5.79962476340667	5.56992309075304	-2.07957763493923
C	6.45697882564622	6.27007198671422	-1.02274268498090
C	6.31421697400391	4.33759495350747	-2.52238614228467
C	7.63775500028727	5.69723166708476	-0.43648818865869
C	5.99286282118556	7.53586221342117	-0.51620183522470
C	7.45559487591029	3.77337021109467	-1.94608655029555
H	5.78593968698353	3.79999379472040	-3.32182704948489
C	8.31835129484492	6.38004252335441	0.62322582204185
C	8.13958694120999	4.43438558772686	-0.90412785603983
C	6.62776760422276	8.17393187226707	0.52051782097626
H	5.09772346698196	7.99660722667625	-0.95353790797156
H	7.82397669126414	2.80090730442136	-2.30575474287540
C	7.80952615449718	7.62640582157823	1.12474269056426
C	9.50480274629001	5.81362882652229	1.20383676216607
C	9.32404594045654	3.88780314591626	-0.29778947048352
H	6.22687395717113	9.12374957862843	0.90603875107181
C	8.48372254599536	8.27157274676277	2.18643516574064
C	10.15072542471791	6.50271516005328	2.25405534211704
C	9.98204041196345	4.54980665666495	0.70902123670554
H	9.69479830248089	2.91718202392392	-0.66299360590046
C	9.64317410668941	7.71532385940909	2.74016487714041
H	8.08277512410858	9.22118223882707	2.57318302226798
H	11.05201588763682	6.06113449946456	2.70554056376676
H	10.88484619435656	4.11674351583420	1.16671532299292
H	10.15173159278842	8.22539148144865	3.57152438334015
C	-1.24250579820812	5.88097591059264	0.85098727198457
C	-1.41229760344394	5.89419753013732	2.26643892359249
C	-1.87664787898526	4.88332689150285	0.08621957716976
C	-2.28372785014446	4.92328506261427	2.87294917497494
C	-0.72416574738894	6.82281199532317	3.12124560361747
C	-2.70550006957742	3.92776503050311	0.67309208605785
H	-1.71547090552340	4.86292118990537	-0.99885235601991
C	-2.50973652278732	4.94760877920588	4.28626229639210
C	-2.93621564425881	3.93091968068085	2.06576324470942
C	-0.92543972194736	6.83069858549631	4.48085407130445
H	0.01375708370559	7.50340671804873	2.67705537719845
H	-3.18184667171284	3.15772306395020	0.04720602289288

C	-1.84525089243161	5.92275082246960	5.10616613778954
C	-3.40143761832178	3.99708168602312	4.89257756939901
C	-3.80108136835913	2.97598000077578	2.70166485014857
H	-0.34950619795069	7.52070317615710	5.11486404446760
C	-2.10087872196687	5.94162060193400	6.49706437789479
C	-3.62838330524119	4.06048087120108	6.28627080645851
C	-4.03054982578672	3.01104147500643	4.05638997231252
H	-4.28149443754341	2.21102506040979	2.07146319500384
C	-2.98854979766117	5.02579864395614	7.07507159163528
H	-1.57825859645129	6.68072779458713	7.12260247647968
H	-4.31586961618246	3.33370581774543	6.74670137861308
H	-4.70110224667275	2.27608396755766	4.52883823001727
H	-3.17429387664300	5.05564871360456	8.15953185337202
C	5.15470311875837	0.15216122719566	2.55463237383461
C	4.06207977119484	-0.15724412174421	3.41172937224762
C	6.17522377700088	1.00905222315448	3.00590413622457
C	4.00562952659673	0.44207773284021	4.71742182826891
C	2.97282235676259	-1.00506868392165	3.00617530976284
C	6.15194145496565	1.54839036964395	4.29504876642365
H	7.00239368610360	1.25898523247675	2.32718505816060
C	2.85701456500366	0.24299352601307	5.54970742315893
C	5.07637795452200	1.28451463560066	5.17082817039280
C	1.86520979280408	-1.18214551489762	3.79645674490190
H	3.03101853864372	-1.49024988485787	2.02353168252235
H	6.96960713436009	2.19850136998988	4.63489108382154
C	1.75153155439884	-0.54395602314287	5.07895567691722
C	2.78083001523313	0.87745834941673	6.83661087527240
C	4.99421352968540	1.87206391795179	6.48189636819927
H	1.02335424014371	-1.79710354072182	3.44428394397498
C	0.58966721370111	-0.64713922335965	5.87564695024852
C	1.60247108750619	0.73456766452907	7.60404268716491
C	3.89383647162770	1.67640388452014	7.28038425345263
H	5.81812029484891	2.52269098622994	6.81140081387845
C	0.51676676591453	-0.00751652620952	7.12023751825672
H	-0.26611808690258	-1.22617734150100	5.49644307356036
H	1.53644999638329	1.24527376611042	8.57574034924486
H	3.82795468509195	2.16195482760492	8.26543050768456
H	-0.40192848583902	-0.08362818672147	7.72086225390941

TS-3a-1

C	2.34553846783834	5.91718857502767	-3.45448270121464
H	3.44010461040276	5.81931484672370	-3.54452902542311
H	1.98691113789321	6.65239986995623	-2.71895345035458
C	1.62514456804247	4.71764412669897	-3.52956557269935

H	0.57897628458950	4.62486950025172	-3.18710923527223
O	2.18753320685645	3.67681418065573	-4.09892472250135
C	1.35253417653140	2.50066363604576	-4.36755046616172
H	0.54487415043452	2.82491169019839	-5.05486538865159
H	2.02561532063344	1.80748042408267	-4.89668001805572
C	0.79682536312680	1.90549289171748	-3.10466702000305
C	1.57326666962062	1.01559665111607	-2.33805579772710
C	-0.48580333506291	2.26895075536452	-2.64652819077462
C	1.08694755560423	0.50283100332887	-1.12931568768199
H	2.56738321733838	0.71110682365721	-2.69883859016368
C	-0.97214614944621	1.76927725066332	-1.43356210823056
H	-1.12290685079672	2.90692765096793	-3.27547023980617
C	-0.18662572303217	0.88500365005108	-0.67478732352763
H	1.69132927309828	-0.20229538635092	-0.54220500995223
H	-1.97373102110915	2.05031969906611	-1.07777114506293
C	2.01952320879097	6.92187951167290	-5.10383940139956
C	2.35311839089440	6.06654338159142	-6.21634814745856
C	1.53216350983992	5.10915896471133	-6.78839522477048
C	0.67600164094646	7.49018997913699	-4.98199178780019
C	0.19553410575444	4.77476384427139	-6.46941556222330
C	-0.51184928620997	6.86415711493698	-5.22198335737007
C	-0.72536186201708	5.51289225175867	-5.69767987556938
H	2.80854480112782	7.66357538303434	-4.89219247438268
H	3.38727365803023	6.12471280779087	-6.58380935489906
H	1.99007683218626	4.48634463668860	-7.57416769395299
H	0.62541499432201	8.52180347414568	-4.59499772360567
H	-0.21056535984437	3.88213308536985	-6.97413716522936
H	-1.44625936488907	7.40286703345268	-4.99920398543567
O	-1.94999629391378	5.05823584053341	-5.46406332366383
C	-0.76459474668587	0.29659989827152	0.58948960545448
F	0.19128833458357	-0.17169971338809	1.42458590219590
F	-1.59909731046895	-0.74376883192983	0.31174864617687
F	-1.49667696404828	1.20747655163457	1.27512857479281
H	-2.02025306034822	4.06332214002031	-5.66851451339431
S	-2.53479772884073	-1.72933259939402	-10.79332556181827
S	-1.63474829913425	1.43307119695501	-6.50381821727931
P	-0.12949091916469	-0.89148883042254	-9.44187743813279
P	0.21596729192778	-0.75440548471488	-6.43780745331033
F	-1.20580111390813	-3.46037806607252	-8.40628067486467
F	-2.22610494372788	-5.57163333311259	-7.26752505441630
F	-5.19777858176389	-7.78569370188720	-8.96425495351506
F	-4.63417356039333	-3.59247915918736	-11.73882041738492
F	-2.59872451603731	-1.51248386388692	-7.03683609491085
F	-4.80511995716397	-2.24745552886279	-8.23675271152548

F	-8.03571686788979	-0.04490589478603	-8.39346100044764
F	-4.17393945263438	2.99743506430199	-6.88012970749404
O	0.52313803549671	-2.11488806539372	-10.34162309948556
O	0.56122330799567	0.44282266269769	-10.14180209976107
O	-3.86873343268043	-1.08236832399573	-10.86934040561071
O	-1.78407340736006	-1.95404623697534	-12.04754012104215
O	1.64494591445649	-0.34725499557311	-5.68103243463983
O	-0.05599110404688	-2.24956904333181	-5.78733601267771
O	-1.91287540434846	2.49172380081831	-5.46709470395617
O	-1.03012591898433	1.93121950655626	-7.76842934891643
N	0.48437685621479	-0.91457176999228	-7.97634948699309
N	-1.71305974624993	-0.96862511699683	-9.61908288448547
N	-0.83013039401486	0.27642688199353	-5.76780508837472
C	1.84777418407313	-2.11755096551603	-10.71404099865207
C	2.31585284906405	-1.16486347489173	-11.62295616642716
C	3.73347759421276	-1.09426892750068	-11.86074772962881
C	4.32517356454069	-0.06938170768643	-12.65714046159496
H	3.67161232143733	0.67288531690176	-13.13726187294055
C	5.70137518956194	0.00601292599465	-12.81735174163027
H	6.13630782432947	0.81162051238531	-13.42799010798909
C	6.55268947456770	-0.94682513132013	-12.19797172645871
H	7.64238417177468	-0.87950994382998	-12.33512516521296
C	6.00908934751569	-1.95658393688536	-11.41896833834097
H	6.66004829640264	-2.69987096916563	-10.93258753755358
C	4.60004437222758	-2.05663204950011	-11.22198499276139
C	4.03320971032137	-3.09244883373156	-10.42481943268809
H	4.69149923346653	-3.87098787153593	-10.00936347686410
C	2.66646890146384	-3.15720128069356	-10.17339635362872
C	0.46996918921142	0.51407532370340	-11.52965101816278
C	1.33536777169627	-0.27068603415026	-12.29623427302123
C	1.17597545146888	-0.28023354598939	-13.72710261985346
C	1.92907774806712	-1.13741760820306	-14.58380512962562
H	2.66677489196261	-1.82074147916180	-14.14205208831044
C	1.72949707924207	-1.12897033200710	-15.95670513574268
H	2.31755381034034	-1.80462108736926	-16.59613365086597
C	0.76676277653012	-0.26517577457874	-16.54202230864949
H	0.61928355550457	-0.26617055083386	-17.63249183618984
C	0.00198150656029	0.56244190549089	-15.73531849402142
H	-0.76341832178966	1.22144454663392	-16.17364966166215
C	0.17386287190140	0.57047265137806	-14.31988813883862
C	-0.63475295096039	1.38902786877510	-13.48367973864724
H	-1.38672823794797	2.04619227667417	-13.94632035632137
C	-0.51306496977587	1.37619031691973	-12.09971760975786
C	-2.92441655221359	-3.38467899364007	-10.08570471939391

C	-2.30815697778041	-3.96331873262865	-8.96687480693728
C	-2.85187224731438	-5.10649052140932	-8.35397153755685
C	-4.01181707311089	-5.72355390983593	-8.85181485205556
C	-4.72899272112632	-6.84083060399436	-8.12465472698909
C	-4.58390724217275	-5.17775294794008	-10.01767222201582
C	-4.03477624133574	-4.04879839859327	-10.63977570560673
C	2.68018961253814	-1.26915599775172	-5.66722698823639
C	2.58745243792035	-2.38401825111688	-4.82847379024753
C	3.63957260974639	-3.36716441772919	-4.88055221478633
C	3.56502629995315	-4.60965281267293	-4.18215686311739
H	2.67228343784805	-4.82776106208475	-3.58040553753789
C	4.58878789560660	-5.54191636242614	-4.27090028696536
H	4.50313380449396	-6.49750365972832	-3.73208910325773
C	5.73949613109311	-5.27891990189914	-5.06033294398263
H	6.54480677637797	-6.02603260536968	-5.12174570180515
C	5.83671308888463	-4.08884831951806	-5.76377723379312
H	6.71529061694398	-3.88081431868539	-6.39400975868727
C	4.79759911619358	-3.11371999534450	-5.70363342992944
C	4.87100726352983	-1.90770376730040	-6.45791947502592
H	5.76834334063698	-1.70496197950792	-7.06223514840409
C	3.82894765333239	-0.98914370352118	-6.46506742383991
C	0.11993929101628	-2.44405481789005	-4.42257504819780
C	1.42293688262852	-2.51141956588380	-3.91336363447483
C	1.60558071939925	-2.64842456805779	-2.49025669944399
C	2.88472773907753	-2.56665680083201	-1.86142796645596
H	3.77511946904711	-2.39039545626304	-2.47983283713814
C	3.01603408821574	-2.69618350578629	-0.48531158553368
H	4.01301733335055	-2.62256076882423	-0.02500850888371
C	1.87636334409230	-2.92002356475583	0.33182399144458
H	1.99140781067499	-3.02370751958284	1.42087753419533
C	0.61715737467766	-2.97613772438421	-0.24438383138105
H	-0.27782806496873	-3.10876247657740	0.38065718351296
C	0.44619603174826	-2.82209299741903	-1.65160682470416
C	-0.84879362426257	-2.81137930026228	-2.23999081835593
H	-1.72859410200980	-2.95025159796129	-1.59433967030579
C	-1.03980381839035	-2.61225574907384	-3.60206483615957
C	-3.25921513153718	0.78649695642802	-6.97674483624505
C	-3.48371092591251	-0.55562652812750	-7.31521267025170
C	-4.67127525247598	-0.95210776221280	-7.95354443588664
C	-5.66694743511984	-0.01934625176396	-8.27209111900144
C	-6.90415726681957	-0.35247539299565	-9.08266513718050
C	-5.46910803948870	1.30972263212224	-7.86010569042646
C	-4.29088820722145	1.71060771216783	-7.22270452658227
F	-6.39937815047628	2.24184404772492	-8.11464327702624

F	-6.92263473325798	0.37838418363633	-10.22078511064270
F	-6.98141001111199	-1.64825530441083	-9.41917097453524
F	-3.95436139575944	-7.45719301609580	-7.20931155385401
F	-5.80861967338023	-6.34073295561888	-7.45186108556839
F	-5.68584841387303	-5.71911917012171	-10.54914749455212
C	3.92443317910237	0.27715745477302	-7.24821559964744
C	4.40627728559396	1.46670653591228	-6.63337651635843
C	3.57451842004845	0.28714171534564	-8.60816773093391
C	4.56791835769043	2.65044156862157	-7.43205296204363
C	4.75750775050466	1.52894717889738	-5.23810098343488
C	3.70148367013980	1.44385287466300	-9.38191258935749
H	3.19269702968668	-0.63491068295875	-9.06163607135121
C	5.09985600933621	3.84373094526630	-6.84782477064342
C	4.21598582289185	2.63237991222479	-8.82442116144408
C	5.22730466722116	2.68755303481622	-4.66770965821361
H	4.64248444177785	0.61904087442682	-4.62995219434663
H	3.41567675077545	1.42101845250750	-10.44310533506335
C	5.42243819108589	3.87980826781886	-5.44792982653161
C	5.31669803159293	5.01041990745267	-7.66167213748844
C	4.42279169218561	3.82058792781006	-9.60837475392807
H	5.47528774407912	2.71724395681753	-3.59545549130712
C	5.93397962042448	5.07509260352479	-4.89109710011185
C	5.85180266220060	6.17508878320236	-7.06259612343117
C	4.95872916348803	4.95846945379499	-9.05529691887194
H	4.15395373857006	3.78948475851992	-10.67561254467427
C	6.14885172849031	6.20592475065954	-5.69234453425732
H	6.18428945790882	5.09897317699503	-3.81874334231527
H	6.02553395969934	7.06444186142634	-7.68797401513637
H	5.12398264663834	5.85521802964073	-9.67256246067244
H	6.56346336549143	7.12206338722892	-5.24468725869399
C	2.05145896857906	-4.26348794232423	-9.39754212863005
C	1.00802672765865	-5.06453294410389	-9.95536944728434
C	2.52328661417581	-4.54239780843866	-8.10146592206588
C	0.46491205120190	-6.14233479515372	-9.17398105554788
C	0.47040915518292	-4.84663284287833	-11.27364513552437
C	1.99353088876883	-5.58629886349010	-7.33913147206726
H	3.30897869602234	-3.90343337524708	-7.67577975086413
C	-0.58675803145071	-6.95818545099351	-9.70448390173801
C	0.96636926634437	-6.40540114406583	-7.85302572944000
C	-0.56123373170575	-5.60599774445198	-11.76745083110953
H	0.87822663253683	-4.04047398143898	-11.89648145215131
H	2.37838965689511	-5.77091256266855	-6.32516430952844
C	-1.12591681369565	-6.68564691928831	-11.00782460396758
C	-1.12327241081668	-8.04219297628343	-8.92832023622552

C	0.40622632633279	-7.49362325967690	-7.09731507092424
H	-0.97313482343306	-5.38692674757123	-12.76423221361921
C	-2.18333775271896	-7.48445402322315	-11.49987095609395
C	-2.16999518966694	-8.82148788590409	-9.46909563478867
C	-0.59169525306446	-8.28245492976201	-7.61324393740278
H	0.79956602240001	-7.67983065023515	-6.08570563595288
C	-2.69559981611448	-8.54159118462012	-10.73782601064829
H	-2.59889442366066	-7.26282330043912	-12.49497485945011
H	-2.58682139972843	-9.64272415517632	-8.86670682860940
H	-1.01621796907603	-9.10804506751119	-7.02155923386383
H	-3.52449015761436	-9.14842191694273	-11.13108301211368
C	-1.38119690880578	2.25223395124583	-11.26079046692309
C	-0.86252979041120	3.44840585042131	-10.69162735811282
C	-2.74297155334432	1.93789355111529	-11.10324800905532
C	-1.74862000026676	4.33005946724070	-9.98404121311875
C	0.51674619779414	3.83394090180013	-10.83158071857925
C	-3.60187619149208	2.78071296249315	-10.39013197224704
H	-3.13736721527236	1.00125336815354	-11.52071648627689
C	-1.26055264076129	5.56833444405049	-9.45873959778958
C	-3.13357631779567	3.98610142208633	-9.82509809038709
C	0.98387827526965	5.02597434297582	-10.33261046298056
H	1.19848378828753	3.15468031987378	-11.36394720730055
H	-4.66189336892880	2.50483033175603	-10.28199488581696
C	0.11496292638538	5.93896703334001	-9.64245511477731
C	-2.14900948232593	6.45525443352962	-8.75653931915094
C	-3.99710722539571	4.88738391545523	-9.10952640006121
H	2.04188646383906	5.30389667044769	-10.45417466357602
C	0.57098595759594	7.17631010608435	-9.13314232758055
C	-1.64429258698553	7.67867152969425	-8.26201715540101
C	-3.52410268924661	6.06685667269533	-8.58955908435148
H	-5.04966107712582	4.59790409497153	-8.97285697588149
C	-0.30095040565862	8.03326996669468	-8.45131846563949
H	1.62747206836798	7.45295984317506	-9.27270789704701
H	-2.32742789208242	8.35771535379436	-7.72798779373587
H	-4.19589338570888	6.74270712894604	-8.03822139907195
H	0.07153575565807	8.99063114058749	-8.05612425645876
C	-2.41571723553684	-2.66517885747658	-4.17939433977363
C	-3.31219692990450	-1.56572018187342	-4.07877335918027
C	-2.84051105725793	-3.85669921795827	-4.79515281759400
C	-4.63699446962245	-1.68980638190493	-4.62609076428800
C	-2.92624811369765	-0.30665977959914	-3.50025541693203
C	-4.13813143850622	-3.99314218889155	-5.29650015083951
H	-2.13341911731190	-4.69324084411289	-4.88427781098738
C	-5.52593797053624	-0.56687297724168	-4.61918789133412

C	-5.05659196124843	-2.92410340445300	-5.22783500428712
C	-3.77844132235045	0.77026544573352	-3.50274835107792
H	-1.91393765153712	-0.20682314988240	-3.08800451671546
H	-4.45479700186621	-4.93959620473652	-5.75575822744360
C	-5.09348780536778	0.68971572132364	-4.07485606313173
C	-6.83295680445679	-0.67628940972729	-5.20621294543123
C	-6.38070501364883	-3.01563961474639	-5.78377019829474
H	-3.45047066911402	1.73595213586850	-3.09246340259048
C	-5.95659398572084	1.80633124977697	-4.14647536673929
C	-7.66500335192441	0.46538035055500	-5.24531364754650
C	-7.23220428149719	-1.93799501874239	-5.77413609538510
H	-6.68126134067299	-3.96462108639187	-6.25304325375199
C	-7.22565966920595	1.69213900973016	-4.72920424239934
H	-5.61062547741663	2.77278920390616	-3.74887672749469
H	-8.65760728040583	0.38481577682075	-5.71238042614574
H	-8.23043629242472	-2.01240727462063	-6.23126100153900
H	-7.88191398182958	2.57355247810230	-4.78719938264326

TS-3a-1-RRR

C	-4.98975148936123	-2.57185343905197	-1.58108886124948
H	-5.01086705336253	-1.62907539525853	-1.01075536433028
H	-5.02424592230188	-2.47399946742550	-2.67671900421716
C	-4.13252801738266	-3.57105916492826	-1.09913593626772
H	-3.82503375409440	-4.41465038138475	-1.74428706461216
O	-3.76680732255144	-3.53744863048187	0.16812916856340
C	-3.09760103618898	-4.71871018177045	0.70477008721925
H	-3.76729877760748	-5.58259739727781	0.50256128044389
H	-3.07028928801191	-4.52839091708365	1.79307058478416
C	-1.71344698212698	-4.96345033178881	0.16358962174404
C	-1.51351175490315	-5.73660874565026	-0.99888894134641
C	-0.59317537717050	-4.44361222685117	0.84506417820296
C	-0.21607360665799	-6.00163615549322	-1.45685617195208
H	-2.36620468559588	-6.15546844029637	-1.55409024243440
C	0.70493805666836	-4.72224372760284	0.40225570245469
H	-0.74057515001358	-3.83211576553223	1.74893554511925
C	0.89141293781871	-5.51572905273060	-0.74318343810387
H	-0.07063316506381	-6.60350523208627	-2.36287577266033
H	1.57687908212281	-4.33541240946138	0.94809678832072
C	-6.82747353138550	-3.16510593458729	-1.27338191376439
C	-6.98369232065382	-3.53001645905618	0.11725628318831
C	-6.64895494293020	-4.75111121589322	0.67969297118705
C	-7.11798616678530	-4.14478915368764	-2.32140004231539
C	-6.11555830827951	-5.89879400350884	0.05016011294126
C	-6.76458725855836	-5.46102556213215	-2.34854830136540

C	-6.07644602893650	-6.20880398623975	-1.32442627437629
H	-7.26014027192809	-2.17695479060225	-1.50389872529866
H	-7.33479183655447	-2.74051484501779	0.80068641560806
H	-6.76699587439309	-4.83083409836670	1.77175473259877
H	-7.60815434708516	-3.76441569252645	-3.23158801053893
H	-5.76846948604222	-6.71811445740531	0.69988556713007
H	-7.01428829678070	-6.02662067711366	-3.25677556999775
O	-5.48678437512845	-7.34948985054411	-1.64925245384170
C	2.27934855314901	-5.93916952913250	-1.16256502627767
F	3.23753557295273	-5.12988642842082	-0.65810216547037
F	2.55030009657176	-7.20114661876393	-0.72500281688230
F	2.42185739302890	-5.95755844180233	-2.50995308885549
H	-5.35017577030560	-7.44364439024290	-2.65655609357320
S	-4.06469249474235	-9.35551204583587	-8.76340267209950
S	-4.35463883888771	-6.05908028628085	-4.61134551361700
P	-6.27791315602836	-8.47952465683766	-7.12539171015808
P	-6.65624315406455	-5.61520432509333	-6.25500328764776
F	-2.52014375770041	-9.64643532143204	-11.27019115906082
F	-2.03857579664179	-7.86606100211990	-13.17800346586464
F	-4.22316416577252	-4.36307605932275	-13.52404409001079
F	-5.90302540203742	-6.77758286591627	-9.48669702712730
F	-1.71067094830782	-7.12984195914577	-3.82689916151754
F	0.45192345828406	-7.57754065685882	-5.30943617801782
F	1.62578337484638	-6.96072894854022	-7.69499894505885
F	-4.06458514621359	-5.76200650656777	-7.75258888565494
O	-7.15765166155626	-9.16951410505581	-8.34046189108950
O	-6.61914029980440	-9.47195523007902	-5.84295120683007
O	-2.60580317353914	-9.37620085467835	-8.50680962266572
O	-4.75093385463216	-10.62638818606542	-9.08463328975349
O	-7.93440711823220	-5.09282061589751	-5.31210729104246
O	-6.80158635277894	-4.59676748596667	-7.54714063852728
O	-3.76624645850943	-5.29700271217498	-3.47872424951943
O	-4.93263458659885	-7.39985202994241	-4.22649219499232
N	-6.94001150499397	-7.09009402539885	-6.71345099073403
N	-4.74534615062550	-8.49927943993040	-7.56564136851771
N	-5.37850853132364	-5.13093020348871	-5.39443840725221
C	-8.45270152009985	-9.58886793495667	-8.11925055262825
C	-8.68108218053907	-10.69524344979436	-7.29942184355396
C	-10.04084349278032	-11.01216008903874	-6.94833106842764
C	-10.37163294808286	-12.02751363781834	-6.00317576577509
H	-9.56057698968506	-12.61390065061556	-5.54899130919943
C	-11.69086502304570	-12.26949632133764	-5.64755459748319
H	-11.92169838563147	-13.05137351457195	-4.90845190696860
C	-12.74435944293629	-11.51373966245754	-6.22638884720857

H	-13.78701192140888	-11.71777148663256	-5.93986964949239
C	-12.45632135673760	-10.51653992559111	-7.14528313744362
H	-13.26493551661170	-9.92057461930328	-7.59676574044712
C	-11.11090539407827	-10.23127518821525	-7.52337655262708
C	-10.80616549857938	-9.20026197631613	-8.45740277569937
H	-11.63173207105324	-8.65755487987764	-8.94309022669534
C	-9.49414838511841	-8.86946086677530	-8.78321733009140
C	-6.52114145911109	-10.84466415729117	-6.05147708602958
C	-7.51990295244108	-11.47887847322315	-6.79636711035497
C	-7.36420046605669	-12.87207262085612	-7.11993746939557
C	-8.26664769140744	-13.57128842410968	-7.97532124997387
H	-9.12170991567723	-13.03126682828660	-8.40443490542500
C	-8.06712037772276	-14.90981780578198	-8.28072367463428
H	-8.77184624483670	-15.42656982017345	-8.94965128420087
C	-6.95689837025094	-15.61546750875648	-7.74592002415997
H	-6.81068130281230	-16.67744596557236	-7.99434663316676
C	-6.05241021105577	-14.96039165407915	-6.92537175358971
H	-5.17847762812491	-15.49138627390020	-6.51727119245624
C	-6.22198916962720	-13.58218001423015	-6.59987653357201
C	-5.29386004251832	-12.89952371158933	-5.76745963556236
H	-4.45351141593678	-13.46228433531502	-5.33314679922663
C	-5.41701611615928	-11.54427100670773	-5.47560981636579
C	-4.17047184563696	-8.25889678516161	-10.24569851234424
C	-3.23928686307436	-8.52389326759357	-11.26770089321297
C	-2.98904104700712	-7.58705940720462	-12.27898058060724
C	-3.69572364292203	-6.36983695200159	-12.34142324343886
C	-3.28545877720551	-5.31606808605780	-13.34723576672181
C	-4.68520819847213	-6.15059225426820	-11.36882031101098
C	-4.93180081609330	-7.08657856897649	-10.34919571080665
C	-9.18264539342676	-4.97023677158386	-5.90535154896443
C	-9.41296925593718	-3.90103790009444	-6.77314424800186
C	-10.68135378051038	-3.83666336300990	-7.45092878280776
C	-10.96065390185802	-2.86879956999983	-8.46128233588652
H	-10.18382976199020	-2.13737805409353	-8.72446985260569
C	-12.18210668963719	-2.85619985141133	-9.11958362029682
H	-12.37087814866352	-2.10849326154891	-9.90450836266795
C	-13.18587116006248	-3.80699002501344	-8.79650905878806
H	-14.15036895962655	-3.78694374068430	-9.32549218497823
C	-12.94389225865103	-4.76530318073392	-7.82446468060918
H	-13.71017507596556	-5.51554659466121	-7.57482641267931
C	-11.69583290361988	-4.81509426440323	-7.13627829401938
C	-11.42818570794357	-5.81855014969499	-6.15890686239427
H	-12.21965825905274	-6.53579588273723	-5.89315935190867
C	-10.18845637158495	-5.91590540797809	-5.54196468243387

C	-7.05756102836332	-3.24947174921199	-7.30992349143584
C	-8.35245639667033	-2.86987718975536	-6.93279712716511
C	-8.60528252624786	-1.48947246388944	-6.60395769677635
C	-9.84944196057437	-1.03356239925447	-6.07184881746505
H	-10.65734506564158	-1.75802190614693	-5.90224774438503
C	-10.04748837617056	0.30390366275496	-5.76014144856694
H	-11.01569439503680	0.62875451050261	-5.34986412637246
C	-9.01299693372297	1.25543564179902	-5.96229988322575
H	-9.18583362146131	2.31492607273776	-5.72049337969732
C	-7.78355451468775	0.84176980424567	-6.45001473694984
H	-6.96498913848587	1.56407727443476	-6.59391844282954
C	-7.54242151655034	-0.52827912073803	-6.76610698297617
C	-6.26767870468501	-0.96878908247508	-7.21674457846337
H	-5.46314470358793	-0.22906311012134	-7.34877855251233
C	-5.99335682953669	-2.30739325642619	-7.47459531756535
C	-2.97138443506297	-6.41532716583727	-5.72196910870038
C	-1.79190218504203	-6.91513526520812	-5.14506353232039
C	-0.66741410019099	-7.19341172852335	-5.92821960700052
C	-0.71245119004573	-7.08474278102385	-7.32965041274804
C	0.48326956909529	-7.54601256800552	-8.14052084527756
C	-1.90169384366742	-6.61971423371399	-7.90669980332729
C	-3.00401277657532	-6.25535766354375	-7.11449675452394
F	-2.03873887396735	-6.48898167182784	-9.22725031589385
F	0.65150568688412	-8.88261645596977	-8.02707931838833
F	0.37354055420422	-7.25648428253751	-9.44865580148258
F	-2.15381775159135	-4.67829690247481	-12.92114200682383
F	-3.00429176929546	-5.83826704644323	-14.55791044100044
F	-5.42572428345448	-5.03709880055680	-11.36642120189381
C	-9.87176119154117	-6.91708251280819	-4.48448894257117
C	-9.88824351141885	-6.52697092283986	-3.11473027719715
C	-9.48674042935414	-8.21988391469771	-4.83547048236688
C	-9.44267629786246	-7.46027571968583	-2.11779680946142
C	-10.33120982659317	-5.22651810626401	-2.68748901993907
C	-9.06363368786266	-9.13580524944885	-3.86669000909500
H	-9.48666340973532	-8.50396583902087	-5.89431728402044
C	-9.42458774164838	-7.08090625587750	-0.73803814320125
C	-9.00598254292315	-8.77340694515889	-2.50510944970813
C	-10.33708782844515	-4.87175665334972	-1.35929959693566
H	-10.67794898674820	-4.51464762882582	-3.45108107583998
H	-8.75172518095966	-10.14474791800891	-4.17331237636732
C	-9.88576223192936	-5.77927219471255	-0.34059201765998
C	-8.94070590718919	-8.00048360372870	0.25561948663781
C	-8.52459475057421	-9.67123153333833	-1.48857173764992
H	-10.69039030521925	-3.87472711839581	-1.05299463322888

C	-9.87766995505655	-5.43569351853944	1.03049446417471
C	-8.93802866454553	-7.60401640215438	1.61243118720360
C	-8.48371426545314	-9.29854466092773	-0.16741724479606
H	-8.18628857145654	-10.67325107473157	-1.79490265737365
C	-9.41058561846110	-6.34012663346506	1.99315093698180
H	-10.24228784390349	-4.44156828289787	1.33223515520334
H	-8.56983832174543	-8.31071404592071	2.37242733945680
H	-8.10866691988951	-9.99662971934147	0.59697496305237
H	-9.41457328983337	-6.05684293469000	3.05693494807416
C	-9.17129206737199	-7.82194746933466	-9.78369089232057
C	-8.33615968408963	-8.09895070564631	-10.90900154669653
C	-9.72102406165585	-6.53534051914226	-9.62880347430251
C	-8.07170315626503	-7.04861242050473	-11.85499740156920
C	-7.74210304185987	-9.38914569898083	-11.14600568035030
C	-9.46909367262918	-5.51513534557326	-10.54886815078085
H	-10.34011247746675	-6.33003234045188	-8.74492849303006
C	-7.21854951276559	-7.29187484736971	-12.98011981025747
C	-8.64986658357758	-5.74542079799254	-11.67411550662631
C	-6.89613012579911	-9.61144094177350	-12.20425958360504
H	-7.95167102677278	-10.21401761902994	-10.45334189565913
H	-9.90651842989197	-4.51822549191500	-10.38913130857903
C	-6.59929869019714	-8.57631834998469	-13.15384771508801
C	-6.95732686160505	-6.24464319821377	-13.92928846413311
C	-8.37347121825935	-4.71799192480849	-12.64208445769088
H	-6.42636669065077	-10.59842799755824	-12.33203942525674
C	-5.72788779596991	-8.78198167376895	-14.24784234643639
C	-6.09150791839725	-6.50350615398515	-15.01508723018312
C	-7.56621609365111	-4.95706994262737	-13.72631126511267
H	-8.82438279390580	-3.72518784196951	-12.48829976582867
C	-5.47946822753710	-7.75515068669203	-15.16679204768577
H	-5.24533721707921	-9.76441780649446	-14.36613874918407
H	-5.88145903988081	-5.69581285577954	-15.73239896828444
H	-7.35443123018985	-4.15908682064725	-14.45445232272178
H	-4.79105523766348	-7.92723653119291	-16.00738160386661
C	-4.49097173119699	-10.89911928075578	-4.50465899075687
C	-3.08918436224758	-10.81906377884321	-4.74078214400952
C	-5.01131022897492	-10.46694127340362	-3.26899803714175
C	-2.22880966731595	-10.33607654824377	-3.69295384551928
C	-2.49760772702669	-11.18033471378097	-6.00107220318388
C	-4.17981912907132	-10.00408715066431	-2.24776615190501
H	-6.09731655962819	-10.51684046219754	-3.10371891463619
C	-0.81412388159990	-10.26879371797526	-3.89995824813218
C	-2.78299511747060	-9.92613347954477	-2.43338285340865
C	-1.14116747918136	-11.10295472138119	-6.20179344382558

H	-3.15679028166737	-11.47670428095140	-6.82815420375503
H	-4.61426078366407	-9.68485413322889	-1.28989064183526
C	-0.25144306385681	-10.67106298066446	-5.15919114179968
C	0.04899877993218	-9.78802907282073	-2.85478143696289
C	-1.89745846423266	-9.44784382825381	-1.40608440701955
H	-0.72245676108610	-11.33795234225531	-7.19112975866652
C	1.14927288570576	-10.60216327211149	-5.33711148682456
C	1.44191827047973	-9.72506975782008	-3.08503453900527
C	-0.53950487328307	-9.38072940472337	-1.60655159418469
H	-2.33699714379019	-9.12978954313653	-0.44735483445186
C	1.98127628609503	-10.13510199060898	-4.31195438842121
H	1.57611695514549	-10.90032423922117	-6.30630992266197
H	2.09668606738551	-9.33638654910144	-2.29032282406185
H	0.12648037544336	-9.00407970077691	-0.81473256767859
H	3.06806154933685	-10.07680690294967	-4.47582768557186
C	-4.63526301505621	-2.71345456154381	-7.94137718136318
C	-3.54020414975221	-2.79834497454271	-7.03650991905491
C	-4.43389485736876	-2.97847806617987	-9.30825247994269
C	-2.24945036528693	-3.19020308940884	-7.53646255725743
C	-3.67783505530482	-2.55726537592641	-5.62435335373904
C	-3.16973695407599	-3.30675261601280	-9.80582756896830
H	-5.29109160773733	-2.92671101782274	-9.99378817891993
C	-1.16105835692192	-3.40374588436577	-6.62974453356434
C	-2.06038248120961	-3.42289415675680	-8.94072619274337
C	-2.63202062484840	-2.75756940656114	-4.75859314800952
H	-4.66116232079366	-2.24589341598390	-5.24490811801699
H	-3.03027277172871	-3.49262353894434	-10.87987460286705
C	-1.35384320369635	-3.22417039613180	-5.21840136779093
C	0.11358783734163	-3.84786211747747	-7.12261122289278
C	-0.75987002697357	-3.81776589330991	-9.41331776054354
H	-2.76487314134155	-2.60029013237218	-3.67846465665788
C	-0.29897433957617	-3.53896055582584	-4.33255073168702
C	1.14281349623443	-4.13863486567704	-6.19871916884805
C	0.28173042722707	-4.02330911000154	-8.54203660652518
H	-0.63258697048673	-3.99772088179050	-10.49154164978329
C	0.93101424065500	-4.00098938235514	-4.82005821974670
H	-0.46120981984753	-3.43077065033221	-3.24888729368594
H	2.10797370153472	-4.50726871138043	-6.57580344887337
H	1.26009315536434	-4.36139692067485	-8.91494844528541
H	1.73124302267699	-4.26792122208339	-4.11569417187991

TS-3a-4

C	-0.09465019078598	2.02365516721371	-6.31594585170908
H	0.17384097357339	2.89523926216021	-5.69533868087868

C	-1.03927124797059	1.07761177270061	-5.89403375251714
H	-1.44481426738634	0.38239214290650	-6.64574604743660
O	0.66511561359896	1.94037776594505	-7.38244794936169
C	0.37609315767997	1.01054115849169	-8.47864226246675
H	0.90505707991005	0.06338432988210	-8.26406609135011
H	-0.71278241937322	0.81201137600135	-8.52394019162800
C	0.87708016363114	1.65673461415235	-9.73901892669598
C	2.17758982405680	2.19664720951682	-9.77881250086279
C	0.05387322191126	1.75423266393321	-10.87408050881319
C	2.63942008116724	2.83524007463843	-10.93270247391289
H	2.82832204257354	2.10007769158321	-8.89978179639743
C	0.51625407368939	2.38549266231656	-12.03654290881311
H	-0.96283486663937	1.33306303571074	-10.85406475962247
C	1.81121875880044	2.92720870962467	-12.06541302461899
H	3.65083011341777	3.26413778072624	-10.96470600241214
H	-0.13555607088143	2.46979357677212	-12.91617103334917
C	2.04496722183299	1.69030203514853	-3.74987530321726
C	0.91289778536285	1.25983600226377	-3.01011659420112
C	-0.03734038368563	0.34326564188374	-3.41632673149666
C	2.64506987002563	1.02331883020879	-4.82393457446039
C	-0.17111302884394	-0.23389368360770	-4.73495804531766
C	2.25366216038796	-0.14823663302753	-5.49816898907464
C	0.98843164536371	-0.75430092402977	-5.47300871393244
H	2.59099408385353	2.56064576665992	-3.35190995720532
H	0.76830741804237	1.72310197424437	-2.01968655927683
H	-0.86919064956272	0.12480458124967	-2.72627318277307
H	3.61158504310811	1.43458581017787	-5.15993365420341
H	-1.00071478261073	-0.95614775976561	-4.80170082076860
H	2.99216698950532	-0.58203360192652	-6.18629399520593
O	0.68992181640325	-1.79965210658411	-6.22127487622651
C	2.34843282330997	3.54823939754020	-13.33070373015833
F	2.96322058312363	2.61817283777534	-14.11538331169098
F	3.26876210705766	4.50794104822368	-13.07449435245258
F	1.37067907908941	4.11029916415303	-14.08099705622489
H	-1.78613263071293	1.44413182855976	-5.17532773788176
H	1.49404315372838	-2.14934959430628	-6.76382022388988
S	3.40196323848403	-7.02436796033421	-9.09216494822426
S	3.12736234736650	-1.71586769569978	-8.79508308017848
P	1.08713849723868	-5.31668988042158	-8.99330876820746
P	0.89055285364140	-2.63978217821517	-10.39786188984559
F	5.12882067526816	-9.14407673061721	-10.17340762979309
F	6.09408891294500	-9.45195900410527	-12.63073315418442
F	4.61185712593219	-7.32254677804515	-15.86511043155860
F	2.06981925971216	-5.85662511715191	-11.81768278206366

F	5.90979909907385	-1.32727713563227	-7.68185550560877
F	8.08455487030881	-2.79595266629551	-8.12300219002308
F	9.29504074375242	-4.43709248335919	-9.98979617909527
F	3.60785320461372	-3.83241716436429	-11.04124554280508
O	0.29554804352465	-6.64340966968954	-9.58517259617978
O	0.45856510565577	-5.15922029036556	-7.45607969177926
O	4.80959532501156	-6.79388010121747	-8.68557450812250
O	2.63321741333383	-8.11315136958681	-8.44838252985767
O	-0.42339589339532	-1.61607164700091	-10.25111884671190
O	0.92704544017487	-2.90092085611463	-12.02619054919834
O	3.49889228204233	-0.33116036982393	-8.41605284054933
O	2.64833666306463	-2.59239504408250	-7.65422888758811
N	0.54042461817852	-3.99961344956212	-9.69579246913372
N	2.65382948209021	-5.58708708269632	-9.07598184379930
N	2.11698723926507	-1.66647146434062	-10.01934300869532
C	-1.09042559992645	-6.64177627146943	-9.56412292153699
C	-1.73353474184697	-6.79327855963969	-8.33746213387845
C	-3.15907013083384	-6.57723773397684	-8.27765687428386
C	-3.88753618945569	-6.54416685257886	-7.05231351597467
H	-3.35709298242939	-6.73531252525711	-6.10997563852205
C	-5.24391214683356	-6.25145457980070	-7.03339494136898
H	-5.77954012966208	-6.21706358301428	-6.07280337673005
C	-5.94416732066163	-5.98964339437013	-8.24010962122503
H	-7.02079544467729	-5.76372190407721	-8.21226131182542
C	-5.26619084389098	-6.01550299626619	-9.44898523337600
H	-5.79356180229083	-5.81292084971844	-10.39448944455546
C	-3.86856159280308	-6.29577169524789	-9.50123312691981
C	-3.16578156551033	-6.30511142417230	-10.73732109125819
H	-3.73149823293357	-6.17155346902951	-11.67209253202981
C	-1.78726703587665	-6.48850274528335	-10.80366816372499
C	0.04304326520422	-6.21057330027826	-6.65910696803869
C	-0.95243566383462	-7.08798393425469	-7.10649041934620
C	-1.27444628181801	-8.24056571416198	-6.30604535884625
C	-2.13282364404350	-9.27910236319556	-6.77341507314910
H	-2.56080414021245	-9.20381062954992	-7.78303497354996
C	-2.41041492749122	-10.38387835419697	-5.98105715958094
H	-3.06292587471537	-11.18132010830592	-6.36753095734490
C	-1.85167471580914	-10.49941494041341	-4.68074992408436
H	-2.08020493440004	-11.38017271526004	-4.06197294615581
C	-1.01063367704254	-9.50796948387299	-4.19915440524073
H	-0.56314538325860	-9.58997777633753	-3.19644544616070
C	-0.69738042203409	-8.36592551032990	-4.99261164583686
C	0.13861070912231	-7.32193279182688	-4.50964412574239
H	0.49743178567241	-7.36114783697238	-3.46977509096101

C	0.52212202563087	-6.24932526266580	-5.30831228684349
C	3.60895195777743	-7.44311912679947	-10.87336054536106
C	4.59702060084949	-8.40813177899198	-11.14894472139827
C	5.10119073985263	-8.57551812758480	-12.44479728463127
C	4.60498402901886	-7.82075584976647	-13.52593153882889
C	5.31074752823785	-7.89795844011239	-14.86365848861987
C	3.55383263989276	-6.92872773962553	-13.25894041807560
C	3.05393970815501	-6.74692273411196	-11.95612653201651
C	-1.60475068279981	-1.94847900833849	-10.89974340210100
C	-1.66733519407561	-1.85531233229168	-12.29597213243678
C	-2.85749429934197	-2.31772485025930	-12.96487372009484
C	-2.94268018781998	-2.43861240506178	-14.38469811995677
H	-2.07878229227620	-2.14503657383224	-14.99662422403878
C	-4.08401966334738	-2.94210938364865	-14.99142608790112
H	-4.11830938876749	-3.04294101418462	-16.08656108808081
C	-5.20340800016234	-3.33850860639503	-14.21240558570494
H	-6.10301983164593	-3.73452292036323	-14.70673382265131
C	-5.15301535383501	-3.23644703514077	-12.83118084472269
H	-6.00883523895012	-3.54950195560559	-12.21280989603457
C	-3.98760849663227	-2.74012263039316	-12.17531854678803
C	-3.90704040318644	-2.67687452717225	-10.75736802556296
H	-4.78119338772539	-2.96932587831591	-10.15594079171648
C	-2.73676678695643	-2.30522842818900	-10.10522316625261
C	0.76063421987491	-1.83449339877139	-12.90108442112945
C	-0.51994547583215	-1.29164725704489	-13.05497647185637
C	-0.69329112499772	-0.15777780061547	-13.92707006146764
C	-1.92303090005488	0.55975702010769	-14.03104339132404
H	-2.78320395953856	0.23901278473705	-13.42804526773181
C	-2.03971202361603	1.66081529962147	-14.86869923890271
H	-2.99543185715960	2.20379243015447	-14.92278329790875
C	-0.93719426371899	2.09528961815605	-15.65124398939856
H	-1.04138668527569	2.96944694270946	-16.31077194141501
C	0.27657077185063	1.43309830415300	-15.55782419864538
H	1.15070154993703	1.78366782236789	-16.12578609199229
C	0.43588922035901	0.31420043932875	-14.68833936132207
C	1.69883120137498	-0.31892377016820	-14.52837480330911
H	2.55706789857950	0.04901754946961	-15.10926023305266
C	1.89233849742959	-1.36931940768796	-13.63918009923828
C	4.64390663542547	-2.56354087560549	-9.30077538254892
C	5.82938312576639	-2.30521516029534	-8.58319311784030
C	6.96793473873746	-3.09178833012627	-8.80021720426424
C	6.95062788430177	-4.19092841117681	-9.67747219377131
C	8.15284995726417	-5.11421411687780	-9.71116333530066
C	5.78385475494713	-4.41134256627267	-10.42266160177070

C	4.65400650048682	-3.59334028481877	-10.25317310284175
F	5.70088146834874	-5.38690533072574	-11.32831631168878
F	8.32300537116495	-5.69252318465300	-8.49514834735069
F	8.03665658404701	-6.09571344284715	-10.61840395548505
F	6.50665880027247	-7.24631391885806	-14.79472451113839
F	5.57756001139822	-9.16760322240414	-15.23150536844750
F	3.00174498789680	-6.19510853058806	-14.22978474300764
C	-2.71253297793393	-2.28632530041835	-8.61381316625149
C	-3.32361385887151	-1.21964217508033	-7.89538198806022
C	-2.16393608613131	-3.37120634832686	-7.91297835794627
C	-3.41889067186685	-1.30550933184058	-6.46229559832032
C	-3.83025705932458	-0.03799728771262	-8.54517637997981
C	-2.24958818669989	-3.45853738891329	-6.52187037024007
H	-1.66527639412743	-4.16110228625290	-8.48237553362493
C	-4.00762086832330	-0.23533563102041	-5.71511968866603
C	-2.89200003687744	-2.45165283611064	-5.77407193932256
C	-4.38092768962371	0.99605579417937	-7.82541852016817
H	-3.75997523625785	0.02545248627223	-9.64120637547354
H	-1.81119806989595	-4.32142892253140	-6.00134588883892
C	-4.48853534551669	0.93812848022573	-6.39210551426939
C	-4.07739827951254	-0.30945264333809	-4.28011801399977
C	-3.00623650617064	-2.51370307632205	-4.34035745337020
H	-4.74933163391506	1.89621866226444	-8.34153261035593
C	-5.02232453747728	2.00203565590530	-5.62953593657211
C	-4.62022479490945	0.78066320516198	-3.56247865946850
C	-3.57359170309545	-1.48795010711056	-3.62290940636379
H	-2.61322976091894	-3.40385304794634	-3.82653351342888
C	-5.08462580485655	1.92171335905813	-4.23185228850528
H	-5.38799632219440	2.90040186917702	-6.15044507094420
H	-4.67469388088460	0.72174184813242	-2.46431672155712
H	-3.64794433117056	-1.54848815186090	-2.52586097247042
H	-5.50360867648634	2.76000590408609	-3.65495462439144
C	-1.10307157837458	-6.54671074462767	-12.11846177096670
C	-0.27901466914280	-7.65148320433310	-12.49190170562976
C	-1.34745379788886	-5.51965483556174	-13.05035889579301
C	0.27274842857645	-7.68978178567937	-13.82024834935736
C	0.02461069558896	-8.74191415381962	-11.60221095954592
C	-0.81845827218175	-5.55807974696969	-14.34114259832523
H	-1.95751748386885	-4.66212099016835	-12.73939063629037
C	1.12663080536533	-8.77067374902607	-14.21393501546822
C	-0.01263665137630	-6.63834635591720	-14.75770888435344
C	0.87270191424390	-9.75779596193172	-11.96836252979961
H	-0.41379047440738	-8.75428897756305	-10.59665339471048
H	-1.02770210616541	-4.73614636035788	-15.04299401781853

C	1.46223080045266	-9.80556366021017	-13.27648218430118
C	1.68012146327837	-8.81032616189263	-15.53997985038480
C	0.53975854618929	-6.71404514137138	-16.08341313106119
H	1.11440109521514	-10.55639023463656	-11.25048685610429
C	2.35264882196921	-10.83169700161677	-13.66677646267217
C	2.55311139285527	-9.86456409559854	-15.88805137661049
C	1.34870192986696	-7.75645281526767	-16.46125759406154
H	0.30571136309861	-5.90342305529302	-16.79111588097608
C	2.89176658912677	-10.85715646116863	-14.95857373018053
H	2.61639453945777	-11.61264821226280	-12.93702049902238
H	2.98754448374989	-9.88301760643551	-16.89907078569721
H	1.77657962432661	-7.79447919814933	-17.47480476144140
H	3.59184157963546	-11.65609882067132	-15.24451195944112
C	1.22119112995715	-5.08889385465209	-4.68357109294874
C	2.57326355676978	-4.74770266870003	-4.95219906933876
C	0.49052026022767	-4.33717761033442	-3.73926734717178
C	3.15473752515409	-3.61830540929421	-4.27490963064767
C	3.39553917024150	-5.52152577920936	-5.83915942136382
C	1.05209807970922	-3.24543284644272	-3.07162583216683
H	-0.55016950560009	-4.62956552215813	-3.53173892251599
C	4.51886623852879	-3.26739008650295	-4.52074986971467
C	2.38750970546211	-2.86299167440540	-3.32322653959372
C	4.70981384035915	-5.19305746602919	-6.06149371347310
H	2.96076011893812	-6.40222994080387	-6.33303701859079
H	0.45672809602062	-2.67578449283858	-2.34133870837197
C	5.31187601228912	-4.06166852762931	-5.41545963079132
C	5.11000891036343	-2.13972871982220	-3.85406358329468
C	3.00804030247944	-1.75125280414013	-2.65513249463774
H	5.30213068643341	-5.79438099025575	-6.76585398238727
C	6.66641334006413	-3.71844977742588	-5.62093658206479
C	6.45910072977543	-1.81379840396915	-4.12227687580353
C	4.30907143607598	-1.39706798431282	-2.91798212019875
H	2.40900355263304	-1.17865650332824	-1.93052391741022
C	7.22596578640372	-2.59697480377667	-4.99507936899833
H	7.27633722448747	-4.34736944200059	-6.28517016015289
H	6.90647637139977	-0.94129103210153	-3.62072825407076
H	4.76579982719312	-0.53608694574026	-2.40476515876414
H	8.27577232575737	-2.33323699968711	-5.19125384396050
C	3.23837004611058	-2.00701372675633	-13.53933140290005
C	4.28984080277675	-1.40213269847642	-12.79611892518735
C	3.48464637918219	-3.18831864392872	-14.26161577584485
C	5.59341932318446	-2.01064575521157	-12.80677773469830
C	4.08737648840820	-0.23087813352909	-11.98540473046876
C	4.75649458457400	-3.76856817370211	-14.29337332050213

H	2.65821514724359	-3.65816013952308	-14.81372862342394
C	6.65327364316460	-1.45412898888055	-12.02110647467281
C	5.82931458346096	-3.20036235492717	-13.57574713980479
C	5.09830231474777	0.28232480453699	-11.20999191083416
H	3.09085475811539	0.22874897605517	-11.96396426326680
H	4.92605744516080	-4.68066890166901	-14.88235835807015
C	6.40781373003071	-0.30718369398628	-11.19286240429728
C	7.95077684435669	-2.07154130866640	-12.01613437465450
C	7.13990978588992	-3.79492075936335	-13.56011075509591
H	4.90789231859255	1.14686899947079	-10.55914475212040
C	7.44797168604793	0.18734288140215	-10.37384594325589
C	8.96462969934216	-1.53008574807058	-11.19408501529738
C	8.15868964628404	-3.25027099680995	-12.81736357602881
H	7.29637278287350	-4.72024996662434	-14.13480559844730
C	8.71046455097445	-0.41966418431504	-10.37775372832418
H	7.24668917396132	1.05202702784382	-9.72320078834002
H	9.95307509746982	-2.01238363402921	-11.18107400069105
H	9.14974587076133	-3.72800397522957	-12.79606931518600
H	9.50761624970985	-0.02756140914988	-9.72847879662321

TS-3a-4-SRR

C	-5.80967895369293	-0.05671291349679	3.55923773733135
H	-6.69582038191535	-0.22263133046401	4.19883946286881
C	-5.78481591810319	-0.28452486343729	2.17307067522521
H	-4.77654086777130	-0.44364347158172	1.75515086539726
O	-4.75537022723313	0.52330133052275	4.08431135265929
C	-4.78912276253221	0.93387886720188	5.48851419160432
H	-5.68419492381952	1.56967815427767	5.64364142841122
H	-3.87658231098167	1.54043593886827	5.60007807198881
C	-4.77906720258115	-0.25179188030043	6.40835779343994
C	-3.58244357756295	-0.96845520130659	6.61365354459275
C	-5.95606988368867	-0.66956375425686	7.05501209858869
C	-3.56340971988055	-2.08487243004132	7.45516664620677
H	-2.65734131910499	-0.62759712411020	6.12477780687352
C	-5.94077336448175	-1.78019880940127	7.90811348699532
H	-6.88940157123554	-0.10222238040576	6.91784280644720
C	-4.74326092227100	-2.48363946317279	8.11218498829641
H	-2.63081902055277	-2.64075113380231	7.62072243644652
H	-6.85875189483639	-2.09240104836243	8.42497150653625
C	-7.89155156898656	2.53239008031049	3.51773789376022
C	-8.33419491178073	2.03584338995955	2.26121647176541
C	-7.53985324296435	1.62041143314877	1.21482732722922
C	-6.63349543182045	3.07011857560620	3.80687906390231
C	-6.10887696424576	1.40119909984886	1.25807965008218

C	-5.45037329118919	3.09317008847192	3.04270967697021
C	-5.16645513983682	2.32180870736296	1.90765826290231
H	-8.65743273923133	2.66414554252878	4.29969380603144
H	-9.42587398916261	1.97320836821909	2.11771346409408
H	-8.04529041297142	1.27381503564339	0.29915923997715
H	-6.55591178826849	3.58861609192597	4.77536954645625
H	-5.68928631845692	1.07456514618210	0.29140791308504
H	-4.62696064603019	3.70717327108673	3.44109768328457
O	-3.94848341760060	2.26403097177316	1.39461859366809
C	-4.71214263657882	-3.61784427175933	9.10684580388119
F	-5.90255848600829	-4.25936533683865	9.18181140741779
F	-3.76755070798301	-4.53863069002957	8.80746293509438
F	-4.43709454470204	-3.16141212137744	10.36348045663650
H	-6.57880297248567	-0.92591687965826	1.76416424580079
H	-3.25372718349945	2.55116251224725	2.08370589592781
S	-0.39737093187606	8.05598245337969	6.51718781461220
S	-1.97514856843976	3.38220600405574	4.42215091431561
P	-2.29360006619041	6.03974322077740	7.39206614410330
P	-1.80337644015976	3.04613120776057	7.26323735469122
F	0.49785457845809	5.63140781318060	8.45044656455512
F	2.93909272927909	5.05477048425716	9.17004670304704
F	6.00853794263635	7.75903853634275	8.92202294458763
F	2.02346227744625	9.75258448182774	6.56436833762374
F	-1.28636444497972	4.17571200070386	1.59217892575900
F	0.52750662755884	5.94566001108466	0.78046435773093
F	3.10377529059238	6.85879421027243	1.43743878494466
F	0.51644424080221	4.42327743995287	6.01263541752365
O	-2.36834937454190	6.88329226934624	8.81049662264163
O	-3.83140162773630	6.24862189564482	6.81189965639397
O	0.06307432093017	8.36512531396485	5.14129985969606
O	-1.11563435934884	9.09966185030649	7.28234246577983
O	-2.93617286151811	1.93669080697360	7.78910722989314
O	-0.48823493715820	2.61733155095561	8.15279570879400
O	-2.27735521359267	2.37229768309584	3.35297753312816
O	-3.00879665528054	4.43538357929828	4.62770776788059
N	-2.22439800921271	4.48520981764155	7.72416482597712
N	-1.15825205463109	6.63094508145726	6.45320263046521
N	-1.58460360790611	2.56629765483049	5.73492300702354
C	-3.32289717057467	6.49565894528168	9.73999036246187
C	-4.66991356955327	6.76947082002076	9.47830738993523
C	-5.66636784436672	6.15739361105857	10.31875457167537
C	-7.05586073964770	6.17102589073464	9.99796264096468
H	-7.38607710416823	6.68876524191353	9.08721298010271
C	-7.97933597343062	5.50765387418029	10.79245880547769

H	-9.04148712579052	5.50301717444973	10.50530323858084
C	-7.56306899485761	4.82836155140456	11.96859877806969
H	-8.30716348539786	4.31876631290232	12.59906244411589
C	-6.22005831431229	4.80071412845270	12.31089555539056
H	-5.88123172760301	4.27344110496074	13.21661306945790
C	-5.23908841123620	5.43035424721744	11.48697056736976
C	-3.85231936583017	5.33273098527703	11.78137851118525
H	-3.54123159281665	4.82123824439285	12.70486855798025
C	-2.87168702791031	5.82802596186547	10.92244722609910
C	-4.58012801258389	7.38797134612853	7.05113366727905
C	-5.03774140320489	7.65787036790411	8.34351681081936
C	-5.84222749449632	8.83673188018548	8.55247918694196
C	-6.24479214664039	9.27698308717872	9.84875550912540
H	-5.91770592085384	8.71087009424557	10.73107809005270
C	-7.02748623188956	10.41124632529781	10.00854267615870
H	-7.31772799489958	10.73303563560282	11.02012857817575
C	-7.44757885725021	11.16533929680186	8.88154791829494
H	-8.07114771383084	12.06119053166345	9.02110795351898
C	-7.05251494524159	10.78084586122527	7.61010933572270
H	-7.35037922929958	11.36919370284913	6.72831478062394
C	-6.23813602042234	9.62707967685158	7.41164532318554
C	-5.79039833875340	9.25653793353402	6.11218231702924
H	-6.09503515980588	9.86482401247053	5.24676428665425
C	-4.94970025311273	8.16981102069325	5.91412524289925
C	1.16761890150669	7.69042254606928	7.40483805898719
C	1.43915393102708	6.52111121547601	8.13027208794384
C	2.75054583762453	6.21424591239171	8.53085656949451
C	3.82328984546959	7.08146288758343	8.26455236211890
C	5.26130327052076	6.70186140845610	8.54758511526230
C	3.53011385917847	8.28695278616943	7.59719690804688
C	2.22370020928164	8.59599486918589	7.19545544791329
C	-3.12554003792763	1.71543338391650	9.14319936315450
C	-2.11231744500218	1.07242799186680	9.86487524043851
C	-2.24131647262966	0.97604010162294	11.29568511684752
C	-1.17910956601397	0.52765843604083	12.13537045902178
H	-0.23244138814819	0.21302557741021	11.67470395711710
C	-1.32161614341803	0.50646324415087	13.51568918414010
H	-0.48284637249576	0.17200191810550	14.14443680639712
C	-2.53563178005001	0.92196190556969	14.12455838013533
H	-2.63559635972063	0.89850967552048	15.21993712631966
C	-3.58588204965398	1.36601455990875	13.33687626067111
H	-4.52986443054545	1.69925685008094	13.79582652060963
C	-3.46627345667791	1.41548580220734	11.91602373902883
C	-4.52175889876209	1.90806022162815	11.10300952672587

H	-5.46122135783490	2.21801560955236	11.58296073710437
C	-4.38710429079285	2.07407464291725	9.72601579387638
C	-0.17034888366982	1.27196884787917	8.29057835097998
C	-0.94952854818816	0.48343552382302	9.14354062956803
C	-0.64021554845561	-0.92133380560914	9.25573825815523
C	-1.43075076829283	-1.83317306655680	10.01813423561548
H	-2.32997722450525	-1.46921731096471	10.53071082708182
C	-1.10050255644487	-3.17863331804534	10.09591938477052
H	-1.73927828742640	-3.85767948635544	10.67838856133248
C	0.03231951096203	-3.68581948142081	9.40704606456412
H	0.28432819748419	-4.75461546375967	9.47655856280695
C	0.80619559678592	-2.83418079122361	8.63439037884334
H	1.67741491672185	-3.21586581899556	8.07970558853294
C	0.49252107979225	-1.44603111992019	8.53179443963852
C	1.27099996965618	-0.57512728280362	7.71940074259585
H	2.14309668754100	-0.98051799245921	7.18381202984811
C	0.96168508193274	0.77203847454928	7.57641339324135
C	-0.50846527576214	4.28520649414652	3.85650767701585
C	-0.44200637300460	4.66903707674218	2.50383184785128
C	0.51338417355103	5.59683978237968	2.07548348981903
C	1.42503029284575	6.18156250134072	2.97175215598658
C	2.32834180556963	7.29001422613148	2.46803424009613
C	1.40180082583833	5.74242293884212	4.30145922857094
C	0.45143629465565	4.80366423988071	4.73712928129159
F	2.27711604804267	6.18749906349684	5.20267542011282
F	1.58185088665720	8.31648286098496	2.00091082183354
F	3.15088645676335	7.77053963852525	3.41248713782762
F	5.37970925112114	5.76323151689839	9.50878324332709
F	5.83742546069825	6.18372165805542	7.42208317737029
F	4.49922478872379	9.16428367391964	7.31611844455321
C	-5.52396938181594	2.57729873562769	8.90903922774215
C	-6.82633915897987	1.99173143168324	9.00547397710413
C	-5.33018612493658	3.66809883004786	8.03947157561655
C	-7.90972911822127	2.56038633961301	8.24241460918956
C	-7.10737463999236	0.81988423946088	9.79553639358505
C	-6.37429439704001	4.21701155645469	7.29687953634226
H	-4.33650504560910	4.11915282857734	7.96827718017697
C	-9.22633813750928	1.99926176596670	8.32305661493719
C	-7.68005065776797	3.69200842300342	7.38726061974504
C	-8.36885883386279	0.27960490529778	9.86851645339672
H	-6.28016152651925	0.33454556642459	10.33017228879482
H	-6.17284538305576	5.08348124718821	6.64959447027281
C	-9.47480270827365	0.85197522995561	9.15159422544813
C	-10.31012302387503	2.57914297190227	7.57434516570538

C	-8.78064572220463	4.25595303879226	6.65317743830154
H	-8.54796576403654	-0.62060766011475	10.47736750100328
C	-10.78140433037115	0.31723222297210	9.21981490449381
C	-11.59993755145172	2.01026727006402	7.67763382719654
C	-10.04485424191117	3.72440862981937	6.74414115251784
H	-8.58330753585350	5.12994502324365	6.01272358509476
C	-11.83040516593432	0.89301868546424	8.49135996024231
H	-10.96448109796800	-0.56179267306932	9.85704987318626
H	-12.42725666646727	2.46095529669418	7.10770861470778
H	-10.88096728641288	4.16963301363261	6.18239742011309
H	-12.84222084234612	0.46550978382909	8.55874528157320
C	-1.43441249868014	5.60371640883287	11.22516380041717
C	-0.44419280621775	6.63331357283098	11.18339434927274
C	-1.05222017104958	4.30048068053194	11.60798433368112
C	0.91726579039567	6.30556067283166	11.52192615212711
C	-0.73534731046541	7.99491058155414	10.81720025556845
C	0.26009757479404	3.98657260526720	11.95903995138491
H	-1.81196917286206	3.50806260125625	11.60733570564123
C	1.94235044310397	7.30373114862922	11.43876169540972
C	1.26735452143986	4.97353217432374	11.93203384079326
C	0.25483696634799	8.93779015409056	10.68882501979209
H	-1.77142501810247	8.28596172332678	10.60785479207727
H	0.51549985781139	2.95611657682212	12.24990409648694
C	1.62630774126329	8.62716160741905	10.97830757096479
C	3.30019609209533	6.98015381426807	11.78305391681392
C	2.62832966429595	4.68423439133068	12.29374041518063
H	0.00150853729166	9.95382380305267	10.35087971282968
C	2.66433126692025	9.57610172589396	10.83424606090366
C	4.29978078567559	7.96876332088048	11.64150707538742
C	3.60404919676416	5.64711334450512	12.22944561576358
H	2.87372531895601	3.66020904913498	12.61585015001783
C	3.98611719588730	9.24713833740876	11.16079195144464
H	2.41610985709710	10.58184153706112	10.46145113170129
H	5.34035063904296	7.71143906652177	11.89024378815114
H	4.64541360632022	5.40888113970010	12.49449234852197
H	4.78433698989720	9.99330715467201	11.03265649866236
C	-4.42955858037196	7.79197778390352	4.57044247935309
C	-5.24704167519860	7.09976830916935	3.63581764672801
C	-3.09664812126453	8.09660160560462	4.24332067821937
C	-4.68394131677189	6.70662561953567	2.37350690413047
C	-6.62598534857269	6.77920446355810	3.90090675688448
C	-2.54042783256557	7.71046296724027	3.02164770091269
H	-2.48474174572108	8.65616430526319	4.96182627261705
C	-5.48856093127740	6.00969266396588	1.41636758328347

C	-3.31344862187034	7.01139079841856	2.06854860895265
C	-7.40589627089490	6.13526574357198	2.96952958095014
H	-7.05282651465027	7.08334655623924	4.86849332409320
H	-1.48606537681442	7.95100142859236	2.81563580090861
C	-6.86956468432221	5.72681135633221	1.69970586827710
C	-4.91825593802970	5.58985197500505	0.16509228490325
C	-2.78054982860048	6.60510566572032	0.79633600152787
H	-8.46223307408868	5.91122874959839	3.18701041057120
C	-7.64365274742200	5.04267306807271	0.73714390771048
C	-5.72845537690026	4.88685831816213	-0.75605198099691
C	-3.54415019698457	5.91166088923369	-0.11003765333254
H	-1.73269744891870	6.84732233221226	0.56783713710957
C	-7.07470452194970	4.62246055559689	-0.47275057410893
H	-8.70215830098169	4.83433003581890	0.95531065509524
H	-5.28681235107128	4.56096545466472	-1.71057911622129
H	-3.11301468999703	5.59224622714283	-1.07130965346653
H	-7.69175427533027	4.08350182204505	-1.20815371579808
C	1.82303716512390	1.66689618594934	6.74803544397031
C	1.73996294522995	1.66870642521203	5.32773411332283
C	2.75299808250886	2.50265287034074	7.39381894399516
C	2.61246021420961	2.53149053977738	4.57660341669014
C	0.76794084758732	0.88836739922967	4.60842645224469
C	3.62707778683819	3.31522320302870	6.66593134311681
H	2.78588206374456	2.51601585207668	8.49253413188610
C	2.48301995497926	2.62093208493671	3.15296356312903
C	3.57930706460953	3.34752298128873	5.25591981870496
C	0.63340471997015	0.98738892968591	3.24542568474474
H	0.09571701931544	0.23448947108279	5.18022292017191
H	4.35771064748993	3.94719744003699	7.19009645730670
C	1.46933778561094	1.86426444581903	2.47210486777408
C	3.32746401887900	3.51276204118844	2.40642002088679
C	4.43227947034531	4.20973937151878	4.48146465745644
H	-0.15158080405166	0.41779356447529	2.72691318963090
C	1.30738050426201	2.02780188269255	1.07779653979569
C	3.13447997391472	3.63464570024574	1.01174727346507
C	4.31276391917713	4.28773111550230	3.11540652109415
H	5.15934218479743	4.83786202528637	5.01831149550182
C	2.12981639332326	2.90650787798392	0.36012707399546
H	0.51353920387914	1.46347202856577	0.56507308895851
H	3.76838926887614	4.33352183164500	0.44640237877608
H	4.95149162839861	4.97405465150140	2.53931998755473
H	1.98271885875492	3.03115938564694	-0.72325527745591

3a-1

C 1.37501744882924 1.75890621150157 0.1640754149779
H 1.00380522392469 1.03281230106326 -0.58512494006312
H 0.97996393387175 1.45435260093119 1.15270803554453
C 0.82327259596346 3.13890772803446 -0.24001529386573
H 0.3265248528411 3.63728969709213 0.62734093863368
O -0.15303625444897 2.90974369247382 -1.24799616828902
C -1.02691666104287 3.98693990941899 -1.49637541073752
H -1.40599579085218 4.40772562993928 -0.52911291590301
H -0.5094000876792 4.83159856573467 -2.01436780832318
C -2.19707938205674 3.53212251287365 -2.33697259827654
C -2.49025140439798 2.16551642947253 -2.4958946788189
C -3.03257868967681 4.48567217177122 -2.94965741796706
C -3.59788203546214 1.75993266553466 -3.25023863758985
H -1.83378127281148 1.42481638834732 -2.0196110434769
C -4.14388800091674 4.08568923700537 -3.70072240734955
H -2.81161238568173 5.5591545248353 -2.83694328572753
C -4.4294780130947 2.71826521894109 -3.85464952766818
H -3.8260374938474 0.69018901657679 -3.36569572682297
H -4.79696002608645 4.83446996483592 -4.17074921873421
C 2.93103558050731 1.69897908662903 0.16713863279672
C 3.40887690064482 2.18222779854994 -1.19372261877288
C 2.90970501844516 3.3468478652208 -1.643730456006
C 3.50033699612039 2.53707322816908 1.29284697784859
C 1.95227373219655 4.10262765814503 -0.75473689946947
C 3.3799476241117 3.88362667648807 1.40085736117507
C 2.65626933077763 4.75726437998367 0.44831692050368
H 3.23477504329846 0.64667318192933 0.32545561948013
H 4.11302924190754 1.57045585055002 -1.77780372362369
H 3.1817000800395 3.76373450059791 -2.62516032737289
H 4.04928321046325 2.00139758855565 2.08694041510934
H 1.48098741015353 4.94988434496482 -1.2867784910612
H 3.83128179942575 4.4197642470773 2.2513054029156
O 2.58204283418244 5.9687095161091 0.63434133285263
C -5.59102567256622 2.26612357375978 -4.70491525788059
F -6.21584108505151 1.19042401030117 -4.1644024212224
F -5.18551360546323 1.89859675133004 -5.94801384510522
F -6.5185925891687 3.2407856756418 -4.86227323076291

3a-4

C 2.94870458812144 1.30580330231106 -1.69213320506494
H 2.28736580518219 0.79063613305063 -2.42829242690735
C 2.24653087409651 1.39826088579767 -0.32676081326048
H 1.41935931324175 0.66346443803526 -0.28790285803891
O 4.13360180259989 0.54746746135541 -1.51293618127687

C 4.69115256446809 0.01726426410995 -2.69324218153797
H 3.98118743376624 -0.6954212018399 -3.18307592208431
H 4.8896230150407 0.82105618733892 -3.44750039423108
C 5.99487743190442 -0.68098721720255 -2.3873741682266
C 6.54928363111545 -1.57635797626896 -3.32322155505108
C 6.68452510698251 -0.43181858062313 -1.1875882041383
C 7.77417660656941 -2.20329383313971 -3.07232135238557
H 6.01077711635044 -1.79286571859573 -4.2597483857655
C 7.91075613757467 -1.05999059994703 -0.93064764308981
H 6.24159616545656 0.25547101046852 -0.45380024898929
C 8.46135053772869 -1.94399024369311 -1.87249182022454
H 8.19632635344569 -2.90956382106477 -3.80177827257623
H 8.44301248947478 -0.87029948513701 0.01238711423001
C 3.2282958054402 2.73744266612876 -2.28292864592855
C 1.88830718516553 3.44718887582369 -2.35721024197525
C 1.12782651002026 3.46384530071951 -1.24751498933998
C 4.23451680361312 3.46411710619105 -1.42023596510062
C 1.67026241239493 2.80523981615446 0.00186480846365
C 4.01900986308051 3.8847968619455 -0.14907633478975
C 2.77045516149885 3.68823256691193 0.62817871758118
H 3.64887360113564 2.62158875207892 -3.30036324338131
H 1.56861849540303 3.91348168528787 -3.30149854295638
H 0.15234181345355 3.97264547007593 -1.21290893002607
H 5.22566925731567 3.65565411910733 -1.86597596622791
H 0.89209161675816 2.72014966523163 0.78247130614077
H 4.80557941766363 4.43193381857921 0.3955000973209
O 2.63387010184113 4.18003309281119 1.7429022275295
C 9.80653434095945 -2.58297021665256 -1.62862083451585
F 10.8048322023108 -1.88491404289495 -2.23011301659037
F 9.85948329307425 -3.84458046820047 -2.12330354723236
F 10.10700892590567 -2.64730333035713 -0.30984363034197
H 2.97768869889529 1.09529928849143 0.44877515179747

2nd lowest energy conformer of **IDPi-4**$^-$, computed at the GFN2-xTB level
S 12.32775303326669025239 13.58304523154448517630 6.35112560532338754626
S 10.66812994704799955059 15.26444606378530188806 8.24924475259337697253
P 12.41743928407764130384 12.26501612330165613685 8.70226738855457071509
P 9.57636795146585484417 12.83066195428349409724 8.41479721420532378318
F 14.52723521419845020830 13.51987414084289262917 4.14181990906113206563
F 15.38429402745571650257 11.43524995128271548595 2.75115529932952007641
F 14.39448018148195274080 7.79445150508953865653 3.65259503748258840261
F 11.08187944728520868409 10.89336319159068722229 5.99711478187115787364
F 10.93927255442767254578 14.81583508394832726651 11.25027759122144921378
F 9.96866494810659986570 16.24672650545813468170 13.27303257269637626337

F 7.03212274456749586449 18.21952850764085241053 13.05619052681728931020
F 9.15235958153776429924 17.94821389747398399095 8.19821618115349615152
O 13.00836200720108770668 10.73432075971949117843 8.54504598538588666656
O 13.06410533298803677837 12.70880427968198134181 10.09100501473270661279
O 10.90202400251033409972 13.50323288452928593983 6.54907750777039421308
O 12.85605147014355864599 14.79354302735961645965 5.83106448327834137046
O 8.45836166200160199935 12.50945787235441031271 9.56375178772632672519
O 8.94640528749403074471 11.99982879396088009116 7.19560005324550022721
O 11.85997645838329717094 14.60603489692147149981 8.71412102807651933745
O 10.80323385514359024739 16.16637138276971086270 7.15857454200368437114
N 10.86285759628042946190 12.05567517155866497092 8.94169044975148352705
N 13.20083440402116004009 12.87989269184070018071 7.43877397128617268152
N 9.34895863265330362424 14.41677018705085266959 8.32896153742074218940
C 13.19139805889300909314 9.89715603846611635674 9.61317410798413796158
C 14.12287319746610592119 10.20907805838787396624 10.58796852345711414500
C 14.22929534757821556923 9.40837356128892743357 11.75894390711224701818
C 15.01929303911673585503 9.78622467055897971022 12.86615379644568157858
H 15.57863003073429197798 10.70843721197517517396 12.81901141593084503256
C 15.06618204068720601185 9.00982424146389249131 13.99135162469363002913
H 15.66866401525841467901 9.31528454474453226908 14.83395223325337219933
C 14.33275213876838094507 7.81625093705053419768 14.06654400659762416126
H 14.38864232395959952271 7.21300986115835573997 14.96027254732108602298
C 13.54725903948757448347 7.42977547852430397768 13.01745728248741329480
H 12.96875158030617747329 6.51862271339526078151 13.06716494511290349578
C 13.46664566220248993034 8.21579505761302009148 11.84887794019204854123
C 12.66292049900266292184 7.84176633603374373394 10.75640003920637788326
H 12.12453594858619077002 6.90567702441708330952 10.80406127505318636395
C 12.50904832273509015295 8.64872143572168106118 9.65175695395582344815
C 14.44491934008386024857 12.60292613940761086155 10.12662966713170398236
C 15.00696909871003725812 11.37424448587714920222 10.38597136038599799690
C 16.42549847913875282757 11.23391840746911363169 10.38013141854315080082
C 17.07255310186318553178 9.98840648796952379485 10.52654708799385474549
H 16.47691775342744691102 9.09605458828065316368 10.63875833078634158824
C 18.43737600182559788209 9.89929714914941882853 10.51573085206542934600
H 18.91626371741794088166 8.93801589549114616773 10.62845332247573004736
C 19.22617259494068875370 11.04709797427472039999 10.35297308000339278067
H 20.30235244293542962168 10.95954008809468760433 10.35733827488481040291
C 18.63023247096215229135 12.26318514696337835801 10.17091200345912049841
H 19.22589424502717037058 13.15159836452900421477 10.01984413521026162641
C 17.22490914586147425780 12.38756582957227969644 10.16313515404085698890
C 16.60171584824769297484 13.62697024898755060462 9.91339090724391169829
H 17.21768265492475791234 14.49552876114622534942 9.73065494829695509793
C 15.23603710946518319247 13.74572909991739422253 9.87708716516301699073
C 12.74224904580276174215 12.28975142027834088765 5.08861376441263502102

C	13.8587177764795619339 1	12.3758699083468783896 9	4.27216011081862667709
C	14.32361289720844510498	11.27976495295649250750	3.54341037366623545779
C	13.68180603791664573521	10.04901572036434487245	3.63733572248729153387
C	14.09454149831007185867	8.82647383897900539296	2.85230181122847348263
C	12.57800878943794664622	9.94570201527076491743	4.48553209658215390476
C	12.13009369741610754545	11.05064922212017286540	5.19764957046511888450
C	8.20863448259315653388	11.16729153089019277445	9.74793063098776180198
C	7.39233401632602316056	10.49406716957561158665	8.86138522890990643077
C	7.27185781483814608350	9.08037099466928232516	8.94378520891269879201
C	6.56599820808568157560	8.32274466895238518305	7.98538401799145436399
H	6.04671636692817848768	8.83749124981665623579	7.19105068078948228560
C	6.55599820714911984965	6.95649000935668393453	8.04010204628095159762
H	6.02260557081765490750	6.38798266182853247130	7.29290666635516338090
C	7.24264121869886423610	6.27941607802891521573	9.05815186572166020085
H	7.22780372706332485677	5.20002998141219130446	9.08236170017435640034
C	7.92889946167790249376	6.98462308342229043490	10.00586592392178708621
H	8.46468160154572757392	6.47273872490095136811	10.79207626583625057037
C	7.97018550693809579855	8.39405556160990684589	9.97208947893716590727
C	8.70434109478858530906	9.13267357755546171916	10.91981840155339611442
H	9.19054878427414223552	8.60783659438287607202	11.72988406853498766225
C	8.82324021907256295094	10.49835321703739410282	10.83284498707966569953
C	7.58088927046874427873	12.07753327081873528925	7.01967859943290850566
C	6.75481569878293441889	11.29800418856380872512	7.79849870496553609911
C	5.34588781176903804493	11.37657933449802705184	7.62100395792469953449
C	4.43761355576740790241	10.67000455147650228582	8.43733688823746419416
H	4.82290233639698318058	10.04677685088474348163	9.22972123852961701118
C	3.08852679286289788507	10.77310186435694561169	8.23714482403483039263
H	2.40487555121799978508	10.22606055161303295620	8.86919821638515415430
C	2.58013205918883414824	11.58959586797657692614	7.21635863927020082542
H	1.51259760716861890728	11.65253554110900502394	7.06690627665962622928
C	3.43285763230701501669	12.31081872877280858347	6.42851247637749523278
H	3.05038665263944874795	12.95762205268751010578	5.65232903620571391912
C	4.82937437845149197813	12.23715051891570304576	6.61487377194317716089
C	5.71430911925537454721	13.02446304999339332653	5.84851403883201204081
H	5.30926794296837911702	13.69500277451992786837	5.10451810882575962580
C	7.06985025181182891174	12.96085580613420340512	6.04192446896630031006
C	10.13385374078318612590	16.35773052766487722920	9.65291446304471101314
C	10.27697057344874842499	15.93357323396358182777	10.96244006915291357984
C	9.73904206657788940049	16.65410231698576382087	12.02700929438447019493
C	8.98299348339530290275	17.80025394398438010057	11.80007707454286070003
C	8.32665737645729819860	18.56001147361117631363	12.92815521818661039788
C	8.78595369813852755669	18.20046009752661575476	10.48012311780110294990
C	9.37025194321431698086	17.49435911237957697040	9.43097296229571568915
F	8.04259236017157519427	19.27674369356946471044	10.21522319457038818769

F 8.88838100991445045906 18.34502789010473478015 14.11820102094628737177
F 8.35346751475827531408 19.88281394476425845141 12.73884389837547104207
F 13.10051009161037072204 8.40970024373661040329 2.04951088943037262879
F 15.15159739575411634860 9.01810405015589111599 2.06721588980258808022
F 11.94177120308902573242 8.77639975754026480104 4.59274413340326059796
C 9.59629349173750156865 11.24723054798073995642 11.83457721811817542346
C 9.00890368185752699048 12.26169846200779289802 12.61670398296126371918
C 10.92331567170547934609 10.89178314214028731044 12.05081061176122325662
C 9.78585305325624510431 12.88804814492136685544 13.62480523574829049949
C 7.64597729556448335586 12.64759804832100087424 12.47749652845600998319
C 11.69533888613221961350 11.53568363043400424317 12.99187175996550891455
H 11.35009570116714705534 10.10613532050828844433 11.44984715106538075702
C 9.20285459700070163080 13.86098344618576305720 14.47187605144668864909
C 11.14608700092641235813 12.53419432241696895858 13.80162040332519879371
C 7.09718628573176424368 13.60436343658934177370 13.26862972876057966687
H 7.04668287638615709056 12.16480567039856985900 11.72132977048809721055
H 12.72978183208250868574 11.25528675069014994392 13.12546499716026815463
C 7.85026057008923849878 14.24126549446398470877 14.29303546338690367179
C 9.97364328700945534933 14.47719478291319994412 15.48969291663159353334
C 11.90291933316189165737 13.18135539941425982136 14.81617172473929855414
H 6.06379187092033333784 13.89431766765886244741 13.14046484322309282788
C 7.30021896765940336849 15.22434200832377015900 15.12265497608279396502
C 9.37861491003653213738 15.44661863612610730456 16.30209534024920259299
C 11.34011564706849739537 14.10821741296122233678 15.63126190636164025705
H 12.94235485729791790277 12.90549273456851508968 14.92705891958172159661
C 8.05922524045718091656 15.81534366701954041901 16.11489949172219127149
H 6.27231718882008060234 15.52219177686027862251 14.97428158239598339208
H 9.96673583704350463108 15.91754648603261657058 17.07616356466646578838
H 11.92083279396914008430 14.58990028034142483193 16.40560851583708767976
H 7.62072103418440249811 16.57557239853469965851 16.74373145176866373163
C 11.66091064938288290875 8.18847263904273603430 8.53995450492313068480
C 11.87504702934829659000 6.92016975339891526886 7.95346199468918602093
C 10.61753772018593799942 8.98620777449807661696 8.08734954259212202032
C 10.96511842217716115044 6.45434311978395758302 6.97040252711121244289
C 12.99081751207448576224 6.09541096044607133564 8.27627747079939979358
C 9.76308453747485138763 8.55189891833426329981 7.09673227897041858370
H 10.48086606149631450080 9.96589987980272340451 8.51272237826407085493
C 11.12823260740440822758 5.16932473497631939097 6.39811501938398219380
C 9.89285305784557067454 7.27621280776159728987 6.54465762008305773634
C 13.15158228833949394243 4.86739417357607795367 7.72216370285152287067
H 13.72992335169019462171 6.47385751424585897951 8.96646400220392436609
H 8.98511762747113706951 9.20962025855499888394 6.74309532767943053955
C 12.21780119475824122333 4.35063425295740469068 6.78271233267762241326
C 10.20507135844080082165 4.69589279172190021683 5.43238269033652709794

C 8.98957869274410370508 6.78343172021841045449 5.56327259003323515429
H 14.00684207524051672067 4.25888524297319648326 7.97986497904908986811
C 12.35543744424388989955 3.08085430108426550078 6.21331130519838659154
C 10.37908668221692742861 3.41811714063276106401 4.89320349492448425366
C 9.13150096065136906986 5.54291049192078322250 5.03610033852761240780
H 8.18106014132284897755 7.42876927436585710041 5.25176688030703076038
C 11.44133855713409708699 2.62263142638727053679 5.28333828879346967256
H 13.18941980121422830052 2.45998006500857080425 6.50729186984787055792
H 9.67259086371057641429 3.05631240713747054372 4.15999389446463752051
H 8.43732578979615510661 5.17291707065263395293 4.29478925079322326752
H 11.56030849437764906895 1.63875178588356895659 4.85356974711546929058
C 14.58115297383146469201 15.02217020833463934082 9.53759717978615206846
C 13.92582291151264684004 15.78835269533811036524 10.51946770787284535231
C 14.62329729654352661328 15.46315996234046430402 8.22100607533993965603
C 13.29677825608212060615 16.99778203938657838989 10.13619278334142137510
C 13.89389382192321065190 15.40858404030898576309 11.88922050146239151047
C 13.95845435518698707256 16.60782780021920856939 7.83444534645656176508
H 15.13105108070469384529 14.86556689560866750810 7.48056062846781966158
C 12.69006199898694831063 17.82082874779812442512 11.11481837948648987435
C 13.27616263982511668473 17.38779748304282790627 8.77485469000175832832
C 13.32111192582041070409 16.20283495070241031044 12.83045635837679832036
H 14.34403539863477838878 14.46829014487461328997 12.17026568629960792123
H 13.94069018829656769753 16.88784729705735543348 6.79389773628650672777
C 12.72312825111822576218 17.44532316605437216595 12.48156573265794833105
C 12.05164707569803894671 19.02783468804240030181 10.73228651731676919212
C 12.58174673818570354911 18.57297285967323929867 8.41219511990910362442
H 13.30199226461166972513 15.90286168594752425065 13.86819003721583776212
C 12.16088720581255344655 18.29969827731905596124 13.43707177315595302503
C 11.49991727885733006076 19.84826163801545106935 11.72250062499074552136
C 11.99369036384625353264 19.36077939042005269243 9.34945943649388588881
H 12.53672704531780901505 18.83121729817879952407 7.36573320694070687864
C 11.56883879543670090584 19.49008396744437732195 13.05738583240842309863
H 12.18683219527507688440 18.01467332862280912309 14.47805185071343103687
H 11.01904801388482901814 20.77182963343756227914 11.43506369647952070068
H 11.47676119605048583594 20.26646686942972408474 9.06580709044477472958
H 11.14768261901467916175 20.14187606594979484953 13.80802898846673798516
C 8.03836298257651193921 13.81901110503725327305 5.33799383673795713179
C 8.95954588092767423291 13.27223175558863133006 4.42212806107752953722
C 8.08336630325335647740 15.17207341168561818279 5.64743116598326988509
C 9.96378259213524763993 14.10871367444577018091 3.87998029831562352143
C 8.90803081988565281790 11.91342782355225260460 4.01246269166923941185
C 9.06739610062444789662 15.98763665417453516682 5.12846220575349853732
H 7.36434966923008893502 15.57224689876786349885 6.34641523860419631831
C 10.90381076274337246446 13.58102995264367329753 2.96454440119338080706

C 10.0397028985410674550815.4691627943843243997404.2629348150996895228
C 9.7892314343400421705611.4141796574840626021803.1057812316692938736
H 8.1405046469052724234011.2787019144554214733504.4307272379054047917
H 9.1129528763399356705617.0265464164802153845805.4126964152954180065
C 10.8112862264739835183012.2296770603006912381302.5472869585005213366
C 11.9369415939430858486514.4065061317476086344402.4539460458453241642
C 11.1054376745926539626916.2622571579065677838103.7677879087237551303
H 9.7323570591793089334910.3803309480037651013602.7936183065471986708
C 11.7263828197575517009511.7406366007244873372901.6063475516948910737
C 12.8387527306230140311513.8690418116371834145201.5277396722654996708
C 12.0223841624177509857015.7516877554759791024702.9035669774152594868
H 11.1746694388940532860517.2832108587759627482704.1093076663990011354
C 12.7243280288594853288912.5564258266450874401701.1047247923718099826
H 11.6412029378622072783910.7163716131866948444401.2719879208944497861
H 13.6299834697077830725214.4938535379712636341801.1412760223618869659
H 12.8379182261931283903816.3587084485056344362902.5393302476715255977
H 13.4228292280806762448712.1639531670052303269400.3803088929845355181

3rd lowest energy conformer of **IDPi-4$^-$**, computed at the GFN2-xTB level
S 12.3689255475831352271113.5071320663221623448206.2230334599588257305
S 10.7836784858593137670415.2615257174078955415608.0875420896789957936
P 12.6146681016069326375412.3316468959177978348408.6142771160537900954
P 9.7455574428594342606412.7974326430001177357108.4439062874656016788
F 14.5642935874788470584913.6253676044236478048804.0581648354473784934
F 15.3848042355098399980311.7007656310384646758402.4141954280714603697
F 14.5710246085818226902109.6746775455681515154500.9928582268947740985
F 11.1128858900167255541210.8034128543792622423305.6215150092700021389
F 11.2148891218032300543014.5567606247769454341810.954331397110321333
F 10.2095050553811024229815.6311831311707010172513.166322277517931382
F 9.0624513295443094307319.3560995988407071877213.238933629031768290
F 9.3217897578705386507618.0113975743871321810708.3661617350588901587
O 13.3062759739385771951010.8538511634541929851108.7411348078810053863
O 13.2082233940986917275513.0081980271492465561809.9416653358178859178
O 10.9536084888214197974313.3534118126255787473206.4407908948928920267
O 12.8167704972523797124514.7596104290091982846905.7221074200249208985
O 8.7597775076550856709912.6305929487369361652309.7522204710640387759
O 8.9859353501326673097111.8977834544367926383807.3660294690857837096
O 12.0389934499160986547414.6235920604723510507508.4008003617356141745
O 10.7730166306834771461416.1958395981649445616307.0188913336512319673
N 11.0713091958200635645012.0245099865516724690908.8500997135334280585
N 13.3127608921099032102112.7721582769737889151407.2383626501257136709
N 9.5051274386117245995814.3739959385456774754208.2450692146259125792
C 13.0329312477394676506010.2199046365217718346209.9335777941450995598
C 13.7204637292461946174210.5723001290780604932711.077469724481479929

C 13.30804426062434409062 10.05566826995275064860 12.33580831471753036510
C 13.88628908601212685880 10.47183362560957320397 13.55331282043543161819
H 14.71486989522501254157 11.16323696404179166564 13.52920358436810843727
C 13.39518117432791832755 10.03049075847272852968 14.75087202952861353822
H 13.84044704477983245283 10.36518046598508746570 15.67580403527361454508
C 12.30381274127166335575 9.15128384370746772447 14.79262606240681066083
H 11.92133440556387213860 8.82346727453591483936 15.74774615148655243502
C 11.72881249608941267581 8.72069293314181770427 13.63048007045629361755
H 10.88707449873522747907 8.04362760956482247821 13.65130188812772260576
C 12.20831941615210070040 9.15793245380757525709 12.37840148656395378168
C 11.61526419863742276561 8.73413700989394037322 11.17324981501016090135
H 10.82244184538985720678 8.00049210555192757965 11.20792254975599533395
C 12.02120594598909875117 9.23190884880194495565 9.95980265734727332472
C 14.51470311117361688957 12.73465328834295817728 10.28363121682744107943
C 14.81595309377760827374 11.55042055999775030273 10.91867329489512350449
C 16.16410132101812280325 11.27563450455497573444 11.28023788036180263816
C 16.56098673713034941102 10.04928634959028599383 11.85415284248434630854
H 15.81669217711375807767 9.28624054056128400703 12.02273617906157632262
C 17.86753251909171069656 9.82162431193042095856 12.18864007814627647974
H 18.15655076676695145466 8.87841744243224617605 12.62792204891290737123
C 18.84242372389653041864 10.80406858647930512518 11.96236605794703145023
H 19.86766848033609988988 10.61108462039550026645 12.24087499426418013115
C 18.49551120916538593519 11.98994616983322281101 11.37803222394938984507
H 19.24244918932882342233 12.74508212809500662388 11.18057858245556701604
C 17.15957483723014576071 12.25312090871836900874 11.00914011891741317584
C 16.80457501122683439121 13.44585043025479542678 10.34367903487045659006
H 17.57498146451325382600 14.16740594530274144347 10.11346578858989708749
C 15.50859280442229071184 13.69020389979721841200 9.97277196607943672291
C 12.77313311275346308094 12.30712091930392304562 4.86717356966977909849
C 13.87167990000540029882 12.48812184594030050278 4.04000921747949259810
C 14.30637548145141657585 11.48956505381736903360 3.17116293216809896904
C 13.61715338378125395025 10.28204369561762732133 3.07707477061596623713
C 14.10993487165297466390 9.19519779363838551944 2.15185060640907277474
C 12.51596147407854076050 10.09540710222176684852 3.90753532291368443907
C 12.13067808455851093186 11.08278769569106003701 4.81180878555975777289
C 8.16784875358626649700 11.43267474079286216693 10.05647450861172664816
C 7.19941397878874411731 10.91419135983536037315 9.21431567337272205975
C 6.67916615411782554901 9.60897228534146385925 9.43396228472372300189
C 5.83503357752522333612 8.96191418383453708429 8.50665515311459152770
H 5.54874236403625520353 9.48433125289416878445 7.60640490900603616353
C 5.39649987742763848075 7.68556678394426739942 8.73061215429346582084
H 4.75671631364745106652 7.19961018903459848417 8.00892046179927241667
C 5.77421669373525769942 6.99354461754026601739 9.89098416822936599146
H 5.41270134163009419836 5.98907963364610917978 10.05272939158803424675

C	6.60100871396653232637	7.58762205119262311825	10.80225717638836080425
H	6.90678184978274511963	7.06181969335543602284	11.69528473618452046878
C	7.07939295334430784834	8.89834758396166947136	10.59475098126157632805
C	7.92847366755059113075	9.53381709151211609310	11.51875290836903431568
H	8.18895512145757464850	9.00951005897555212698	12.42728032970189566697
C	8.46840526010232252929	10.77985371102889367023	11.28619831855365163165
C	7.65256761102533022978	12.21598854830797620252	7.18274269053416514197
C	6.72841973593252973274	11.73500238785515392692	8.08061631997771989688
C	5.35633703271126737633	12.08957963236688115671	7.93518628803889480849
C	4.36870628935915750191	11.72085167463308685853	8.87302829655797964392
H	4.65730451392472577510	11.15135125836487617335	9.74273160753178402160
C	3.06216547539623817897	12.08526177907565468672	8.69736412751128895593
H	2.31834762314444375875	11.79616444002080655196	9.42486971146573182523
C	2.67626602643901811973	12.83932247895359957113	7.57990199219714888557
H	1.63897972630214638912	13.11076280597888654711	7.45256244932861733332
C	3.61398865268940561180	13.23991822999904854896	6.67008812392129257773
H	3.33236200100230206544	13.83914302922090300285	5.81652599853623719639
C	4.97186586890871051736	12.89150486175283205625	6.82773412551469327525
C	5.95353534519689642224	13.34153470411924402583	5.92083621148880734353
H	5.65232497256749244485	13.95645857928021982275	5.08516934879638959899
C	7.27702215004621599803	13.02617602783814909628	6.08847736720047194581
C	10.34594398775029056026	16.26202477948181979173	9.59018370560924182655
C	10.51738691255610724795	15.68115778281370609193	10.83511471123967595531
C	9.98136295175464560714	16.23122818513120080297	11.99669500392606202865
C	9.23083372619822917216	17.40229211035437018040	11.92886044450635374403
C	8.64009274279009176212	18.08557353318420268806	13.13996524701138568503
C	9.02443867968329627161	17.97793511628824703052	10.67563410096633269575
C	9.56800801486201990542	17.40846023206746906453	9.52680666312315693744
F	8.30200269315800198910	19.09594078056023391810	10.58150492353028226944
F	7.30229222835902547217	18.13240060836618994244	13.08457881056776805906
F	8.95326393773325079906	17.49979047390156594588	14.29435016609455288972
F	15.12272893240791660219	8.50632116345757971487	2.70657438014778151114
F	13.17487789392049535309	8.29847359131727380088	1.83546873977768232322
F	11.80482351432359244825	8.97273531670696478102	3.83249591145160195538
C	9.30955546020885904568	11.39866535654099166663	12.32221098554320803942
C	8.87073482415968861403	11.44491308950178343196	13.66657062720924820098
C	10.56126861167333608194	11.90595179170165529570	11.99535406568120698978
C	9.75870912392163880611	11.92291894119234285654	14.66440035529531371594
C	7.55785359925784128166	11.06640874050858336375	14.07061376372153560510
C	11.39938638211398114208	12.42569893747260678651	12.95769551779907757805
H	10.88595228036899698054	11.88487297432566869304	10.96961353517883708264
C	9.37409857926320455590	11.91348389043763944528	16.02761909673200690918
C	11.03600868138702573162	12.41879072678286277664	14.30555136627789991621
C	7.18649186893246927355	11.06358444166370169626	15.37559466182590028893

H 6.84101250165890384380 10.79480265268274941093 13.31034641938769880198
H 12.35404331480577866387 12.83218848504608189387 12.66253087239656238694
C 8.08728556468156689618 11.45675821390326376559 16.40284009199664083667
C 10.27334235609213841656 12.36363402599878646981 17.02658481961903191859
C 11.90863502781645522077 12.89015514570293596819 15.32433848649846730439
H 6.18511720606654513688 10.77107961811160485865 15.65843232773998749963
C 7.73341709798993548475 11.43679606058063313867 17.75549117018290345982
C 9.87925875900629080206 12.32144706140370615799 18.36683966217418628730
C 11.54970203641529380434 12.85433316014606575095 16.63057607190287967569
H 12.87282573711297395391 13.27456802053514550721 15.02529418744600775426
C 8.62508844331210333678 11.85948119438362624578 18.72316985487582030601
H 6.75015001512507240022 11.08840159859008167587 18.03711145845853280889
H 10.56777035830150524021 12.65963822936658544904 19.12786174996493571143
H 12.22173243922184937560 13.20632949820941703933 17.40042992198898730294
H 8.33827098296732316385 11.83591477194965158048 19.76433444817423179529
C 11.41737807027658746506 8.74374094050302552716 8.71064553382560902151
C 12.19166903469581697550 8.10302708860821141457 7.72205828275082062362
C 10.04182700348527390588 8.85293041396319324576 8.54435147849344289739
C 11.53763659708427269379 7.54234175603956202849 6.59491701052673118255
C 13.60229660186206501749 7.94544763240978380026 7.83138192754543194241
C 9.41081144641001721141 8.35849926679218668824 7.42450723983199090128
H 9.47276994196306532103 9.35304898270022277984 9.30879485962640806918
C 12.28109708794412568977 6.83120837873698594223 5.62108142135929256256
C 10.13548877182133622910 7.67749394024725262398 6.44125816939238937664
C 14.31837767085281143409 7.29815863191423730427 6.87733024897439548795
H 14.10298694968133403904 8.35967803745571558238 8.69258363717094084677
H 8.34257959726273234935 8.47463061370372372494 7.31261451074085222501
C 13.68712295132094247663 6.71544754570403945593 5.74394710450645451516
C 11.62566817665909013613 6.24523515388243666280 4.50881577907620467727
C 9.50575670200248801223 7.09541841300257836167 5.30714174013206552161
H 15.39077502444442302476 7.20065270659213219062 6.96956959063322756265
C 14.40626482491760818050 6.03003995083571187052 4.75881626594647677564
C 12.38349726661202154787 5.56151899708581609616 3.55401222830480101322
C 10.21668011960201560839 6.39902650348994050944 4.38567486022171948434
H 8.43629203405189542764 7.21537164846225120840 5.20539609418928783668
C 13.75714672920397063649 5.46099019504271065983 3.67955041350707201531
H 15.47989499576641669876 5.95451725148916466424 4.84884631324835702060
H 11.88386813275860021122 5.12003826139135043150 2.70442445997551850212
H 9.72865094853386835894 5.95368501253955795249 3.53057835310984913235
H 14.32783577438057065478 4.93876914736348826551 2.92620550372600884970
C 15.07819050106640901276 14.86172934627182762313 9.18793263189403397462
C 14.29489075657180663370 15.87684762991998610460 9.77146354422371388182
C 15.38258680160008573523 14.90790018353088797198 7.83388230044414068942
C 13.76928422650247263448 16.89669177752134388015 8.94366037541925962273

C 14.0243174143670596976 15.92030730331645216324 11.1649222420225921553
C 14.8670991777462528915 15.89795835454841643752 7.0230415197187916476
H 15.9929485948737273304 14.12551878865130561280 7.4083799055706940350
C 12.9753629769074692035 17.92311280145241170203 9.5057157529111364624
C 14.0252597905313720616 16.88526071082095825204 7.5518380468540300398
C 13.2968065192356377224 16.92979734435003891235 11.7120210606907804162
H 14.4239519562546405495 15.13530806821820462460 11.7905326756281745304
H 15.0815581532887872384 15.89773873144687144077 5.9665914893874081315
C 12.7544456480455572489 17.96736894967913755750 10.9049420916894543864
C 12.4075227702442028032 18.91804816153603496787 8.6708604604228955992
C 13.4099561718222375361 17.86613370277143886256 6.7337408434043801719
H 13.1120157703424027317 16.96015960904465202930 12.7771616916237782390
C 12.0093901613139735218 19.02334598597617798532 11.4445914455071680038
C 11.6519935711100952602 19.94244099395444536071 9.2542890628605682934
C 12.6247167580435792899 18.83868819863164389972 7.2687344941874183490
H 13.5725751767438609363 17.80695214868061881930 5.6690591759350006384
C 11.4691486341184756270 19.99743844607666432012 10.6248001565881917684
H 11.8630822807398743634 19.07477825428493645177 12.5140710998986719459
H 11.2132130624513468575 20.69811660490053739636 8.6200662962726006810
H 12.1498205230690743405 19.57594048778961592916 6.6384699182168063202
H 10.8940283665074186814 20.80095692175525812218 11.0601914098858689072
C 8.3258178142113905551 13.52575395719292750130 5.1801681669277783726
C 8.9342099106434815780 12.66627479597314120952 4.2455011256436945288
C 8.7160033973271282548 14.85535576621857600799 5.2622329748791427661
C 9.9722359026716027585 13.17091037589951341147 3.4257173238047160523
C 8.5271111353595614446 11.31474715464443825397 4.0739546543580047455
C 9.7594334552522141734 15.33677565924605978864 4.4985509995936787675
H 8.2361733478453267309 15.50338665536669147116 5.9786183404728401313
C 10.5616956900760783355 12.34350344740265903454 2.4402606919400371410
C 10.4191908145796396922 14.50512307052224336701 3.5871359221593142052
C 9.0785526552232305164 10.52601699136652158018 3.1154352539752609985
H 7.7558284457060260308 10.92621410477016929974 4.7221745906214120935
H 10.0912236932589500781 16.35345155653334359158 4.6303575283738176438
C 10.0970886202658647334 11.01744785854872432651 2.2526936203220762067
C 11.6155673911776204931 12.84055127644391980368 1.6323220498989821081
C 11.5123270751861603855 14.96022880747653260869 2.8020870208550618940
H 8.7596073702720111953 9.50116080783618421890 2.9919658763707044357
C 10.6587958218688054756 10.23491651422981085773 1.2371283296851087740
C 12.1521766153626504802 12.01879779853123686451 0.6349432142442790194
C 12.0895398199189276766 14.16227123487028194404 1.8665841518333810000
H 11.8722765225587583159 15.96279922378461257892 2.9718783366617129715
C 11.6662220823899751565 10.73862875883160405976 0.4351840137917868389
H 10.3015807174498696952 9.22653284041397725446 1.0912092340350527663
H 12.9518553002884768687 12.39558072582727987765 0.0143802976595530651

H 12.91627803271031993404 14.51914433186408537324 1.26922493362752408785
H 12.08427360627162983064 10.12536965568319935471 -0.34893677233954889516

4th lowest energy conformer of **IDPi-4⁻**, computed at the GFN2-xTB level
S 12.31444887407615418340 13.54845664578952657564 6.37579312366389494571
S 10.63989127738423334790 15.24547096831899040126 8.27237465592938470138
P 12.38880643756125721211 12.26138748555859869782 8.74273975562326199906
P 9.54809568958195598043 12.81333118468954701541 8.43986222865052937436
F 11.05531443366175281540 10.84706143784244147810 6.07333036196616049551
F 11.95262455444902371937 8.68532213635863392653 4.76232107468724574773
F 14.41656152684235792094 7.66432462274507830813 3.94462086054535365776
F 14.55599135846215474999 13.40855990617928839015 4.22761176518537062918
F 10.85021403303028009191 14.85504109023015750779 11.28972525811848548472
F 9.84229463604709842173 16.32869316432585549137 13.26425928791130814943
F 8.74140184481613147227 18.43878559191648136562 14.04698920260406325156
F 9.12870645318846740679 17.92645313876218793325 8.13971823345297984531
O 12.98516736750359790165 10.73322104450302738599 8.56954229288161961620
O 13.03373387722852605464 12.69073443722754568341 10.13612869718869546887
O 10.88959513541208323772 13.47189494358448236255 6.57753772838218964125
O 12.84167473926622093927 14.74364350728210659724 5.82020087739695934914
O 8.43078861374326393729 12.50663162903336100840 9.59292974958514577111
O 8.91189071694443413207 11.97775530814249123068 7.22714276746726014267
O 11.81744496745546690875 14.58951153555102386861 8.77444629275280973957
O 10.80324942658218212443 16.13001987812311455173 7.17105636326711426420
N 10.83389324160446243184 12.03829324815650458902 8.96960603259053357306
N 13.18441030401743674361 12.89277295770954992 80 7.49368964234953605086
N 9.31969477333915108375 14.39869346316207554537 8.33631991981448905449
C 13.19780699542663526813 9.88746806108211551134 9.62447895905585326659
C 14.13453970982904195353 10.19894903775127303902 10.59405846229744341258
C 14.27147110266725249517 9.37408156952015403363 11.74535253728145534069
C 15.06225162914737403241 9.74721366979368575301 12.85383419285119899200
H 15.59352065466723047393 10.68663190554960884526 12.82421458202551178829
C 15.14426206660585627617 8.94478230831793119648 13.95846950395884888962
H 15.74649015702401122496 9.24695513699836268984 14.80241214865613308405
C 14.44757595327786425798 7.72817267114878703183 14.01043906492975033018
H 14.53155621282951415196 7.10414293009454578254 14.88753666094418193211
C 13.66149995444956033452 7.34558102944939506074 12.96031857356782879265
H 13.11011274088269473737 6.41703489783474978481 12.99296861799092894785
C 13.54357742859665059143 8.15863312659172557062 11.81365962838654937173
C 12.73443711410313028409 7.79124785232629335496 10.72266247925575655131
H 12.22398979787262618402 6.83874767402714045517 10.74989333762828991325
C 12.54161475204314868392 8.62576578353877110317 9.64596271475153699271
C 14.41707287924767655340 12.60379885095462526579 10.15889254509950490046
C 14.99834564043325713101 11.38054040493440766113 10.40247763401685610063

C 16.4189844367625319421 2 11.2596220995527716013 4 10.3845435912892405383 4

C 3.41382041462647478625 12.47437877818759233151 6.43311396327959350572
H 3.05694508863016167766 13.12862050314867623513 5.65100980453692791627
C 4.80631494816758308986 12.35195611678217098017 6.62412382051595471921
C 5.72118483310589276414 13.10052555449886213523 5.85461701486889207757
H 5.34277479009179323555 13.77840384134179352316 5.10326579181493578119
C 7.07349380706042030198 12.99086924207985127566 6.05254948132895265189
C 10.07898191983711377873 16.36469113363876459744 9.64570798673969598269
C 10.19663664006542269647 15.96819689384333429416 10.96648074599285216379
C 9.64088966109000722327 16.71220551143280630413 12.00577861201105811517
C 8.89614979827591945138 17.85741691478322223929 11.74030594935451077276
C 8.22337633623265418237 18.64883766782773477644 12.83660962024481833055
C 8.72538346369863049290 18.23041358710528569986 10.40875916109556342803
C 9.32408872024418045044 17.49903211721633056186 9.38546764596536853276
F 7.99333542881249137224 19.30526015715692267349 10.10859335676327752651
F 8.28841473615492496663 19.96770282433218568485 12.62856397482751447114
F 6.91766966477456879403 18.34124279887247865872 12.92788323184701937407
F 13.19468079231839396925 8.23232346122976821334 2.26926235239391438370
F 15.24857875283218611173 8.82702539080913517466 2.35092315653560568478
F 15.45268125293256389341 11.27612903211596417918 2.93744669455971951777
C 9.55176708256996320756 11.20304051949946533284 11.84072351610224416163
C 9.01580769446455398963 12.24098240638032564220 12.62827270203766971690
C 10.87102665863231543142 10.80500092343592832833 12.02840798128632116004
C 9.83822508105695270331 12.85567938325853454273 13.60648919667834988445
C 7.65998959111407184963 12.66133470173438801964 12.52439266653642846450
C 11.68673692729024971015 11.43565126522274510990 12.94158167911260193250
H 11.25775999785360781402 9.99739040556042546370 11.42826183029658437817
C 9.30681068184382809250 13.85251337961706852298 14.45915451052271194499
C 11.19185942355256102587 12.46385579619142092156 13.74900845821926864687
C 7.15780014792884777819 13.63431578637616681249 13.32660015334457170866
H 7.02769857623095273169 12.18943179307899349340 11.78830431341168782922
H 12.71437781643888342842 11.12389515311111942708 13.05466394656111361883
C 7.95689804243268916650 14.25965534236873111240 14.32288520657423092075
C 10.12481793713736877294 14.46103334912643489929 15.44391205698145697056
C 11.99693874320479913820 13.10329279606677133074 14.73074440770304605053
H 6.12788406470035074136 13.94763914523261050249 13.22839099129052442549
C 7.45562914884702543361 15.25909885685154421253 15.16357932666161367763
C 9.57839916953869874305 15.45027365462824597842 16.26667466560427754985
C 11.48516804402351532133 14.05787174301875630533 15.54761481090772434754
H 13.03095992084376320008 12.79903619547012638691 14.81517900799484976915
C 8.26063324652637476220 15.84319571285620398271 16.12311711530436042494
H 6.42875709669275074987 15.57537390140917921144 15.04974905637203619335
H 10.20234797981942698186 15.91528805411909885947 17.01606885720291728603
H 12.10216903739514648919 14.53280789269825135079 16.29793273328792935217
H 7.85946781889433410129 16.61685410875035628919 16.76031312111215498817

C 11.67615244246015393514 8.18335309955259404546 8.53926367530614349732
C 11.93347398158796401901 6.96413847614302294886 7.87370704870715076851
C 10.57690524934835352155 8.94926085448199337691 8.17206213861576635793
C 11.01491787794039822757 6.50752200448803108657 6.89485508970718807831
C 13.10218850072428331544 6.18554793718967932392 8.11211664502597784576
C 9.70528006407197807448 8.52213661696463020689 7.19307602192519457418
H 10.41366340854209582290 9.90015447466363696094 8.65065935033619481942
C 11.23307728650759074185 5.27624348380488950028 6.23100855563603772680
C 9.88094711634827760349 7.28878252854722408216 6.56273861045583117857
C 13.31558528877840075211 5.00992135416363648659 7.46939544573677860484
H 13.83673848406663964283 6.55893758343593624716 8.81000758244037740496
H 8.88095427465036557635 9.15426889482482764038 6.90470215652480945323
C 12.38402972302262483595 4.50422540720487063481 6.52168923609399975305
C 10.30452202122647697990 4.81231387151689737891 5.26596021898429356156
C 8.96807880538564639039 6.80368883959430537089 5.58630623018788874390
H 14.21131767887034946796 4.43641149824525360401 7.66115231088939641069
C 12.57860085881214473602 3.28889148556989141525 5.85801578513667209336
C 10.53714861998389018538 3.58913641530021898518 4.63085753487402573825
C 9.16485439111758815045 5.61269310188857328825 4.96978899115019689958
H 8.10809356658750601810 7.41426794472674455250 5.35152600236843234427
C 11.66098022276553614063 2.83921108785764175764 4.92732555454961929087
H 13.45950836862848731812 2.70371416969685895992 6.07923878299949116411
H 9.82742953840205046845 3.23411047824677666540 3.89742338593793746782
H 8.46454407125339969298 5.24880594862727622996 4.23118159165175544700
H 11.82485180286597348243 1.89758834311603941103 4.42393968742074505940
C 14.52352896049829311664 15.02776114630263037952 9.56884597234153844170
C 13.86200953700256377488 15.79207314704418863016 10.54763808155776594333
C 14.56605919622780831446 15.46646570618701588273 8.25109178597474013372
C 13.23254212971853505110 17.00051963566143342632 10.16148395830960815545
C 13.82438208977643867570 15.41200474879081383506 11.91708531338571930291
C 13.89675886085581879570 16.60706936448011816765 7.86098630208067916669
H 15.07779483323887248503 14.86958232845673144595 7.51288479575973600078
C 12.62284577839427868184 17.82402713411101657925 11.13784848397875393289
C 13.21421833699034031895 17.38906367040628708764 8.79968473139194173882
C 13.24680036393908189041 16.20575546744449013659 12.85579256927418079215
H 14.27322045763269464658 14.47163053661341614031 12.19999811748650841992
H 13.87776849941648471543 16.88347194894381075869 6.81941403631065945490
C 12.65011037829175322145 17.44816120618256860553 12.50467328126203447880
C 11.98760487543101582730 19.03205658770841779415 10.75321473804254779338
C 12.52306487376630528274 18.57547724505467812151 8.43477661233036712929
H 13.22293288735479244167 15.90534084975658579708 13.89323697928378820166
C 12.08392156448307019900 18.30231132928975057439 13.45800221234742011234
C 11.43328336481803297886 19.85305334878388450193 11.74149256014411868421
C 11.93487716062627690405 19.36527681332441019890 9.37025164453561920652

H 12.4810746808133181673418.8332610802889099943 5 7.38804845536834342568

H 12.48107468081331816734 18.83326108028890999435 7.38804845536834342568
C 11.49514631091095395732 19.49367742010481663328 13.07643995874695619364
H 12.10477641274223969958 18.01656586997924947013 14.49888621285863621324
H 10.95556314008722509357 20.77776437216235194683 11.45240466632384013224
H 11.42132184952435203229 20.27244785093822443400 9.08508770936638576643
H 11.07117488972565588767 20.14539413544986246052 13.82554961408798277489
C 8.06709730629805221724 13.81062589359562053914 5.33899911752536393550
C 8.98632460420614265217 13.22369369875690914284 4.44596412393662809848
C 8.13194214183991803679 15.17099096571783789500 5.61101855601336474422
C 10.00756358847826810177 14.02893973621430134813 3.88807266685474317569
C 8.91711207771574621006 11.85470361713332287934 4.07491280888111528924
C 9.13234282537731445473 15.95600084129956286461 5.07687086748415872250
H 7.41559482425648930359 15.60181136422796122076 6.29428889709897010363
C 10.94891916671952891704 13.45993967282035441713 2.99904542666556750419
C 10.10056687227291938314 15.39910665285865931651 4.23089107290896215829
C 9.80194679537376778455 11.31440057107660202007 3.19570666812103620913
H 8.13507736972094086525 11.24424577261873281486 4.50203189991768759626
H 9.19272129604639687273 17.00131347257894987024 5.33355870614369464988
C 10.84361652111656226793 12.09652715435563052893 2.62604779769976826032
C 11.99792967543451993606 14.25483310129230929419 2.47245462168647023304
C 11.17825436766721658444 16.16309545948923798164 3.71618180108448914467
H 9.73323536431714941841 10.27208697781861701515 2.91570333246308521780
C 11.76635116590760787858 11.56310562181789869385 1.71723270336935485147
C 12.90504230925139772523 13.67440273222681668130 1.57792783596727215212
C 12.09456011992755719575 15.61387478843508347381 2.87540435066357691340
H 11.25744716331638350937 17.19438656218393646213 4.02298641582362215274
C 12.78157335324408450106 12.34840120446352784711 1.20188674072733103770
H 11.67410974362316089525 10.52833704525405167374 1.41892957698636834607
H 13.70843670016754423102 14.27604928786799831641 1.17994551265878389046
H 12.91940512301266963391 16.19920756426611418988 2.49693729052897328557
H 13.48570687892847175249 11.92177640083489897904 0.50262800626580350638

5th lowest energy conformer of **IDPi-4⁻**, computed at the GFN2-xTB level
S 12.40816747912836248702 13.78493181333950090561 6.05021230017758071540
S 10.79705381064060887297 15.51328417069115417348 7.93083067162988974985
P 12.61941730573401798665 12.56812765948305532504 8.43198872571605306803
P 9.73478777544472073657 13.06328452710341636589 8.18989170671343025276
F 14.51555519399021676463 13.89748188294628405970 3.76128025268419374783
F 15.30033897396877762276 11.94972656952170098066 2.15240051336367610446
F 14.97616406242261177795 9.63951842368241784698 1.20538122545401371610
F 11.16648643779166150125 11.09906308856608347924 5.54882679199424977412
F 11.26757608190735915343 14.87600395082479565190 10.82561176173285844015
F 10.30449512943567036416 16.06492093277543986574 13.02109846808648185856
F 8.21037910718852437242 19.58159835976979579186 12.83076609463044093218

F 9.35370667233504882176 18.26240539800643603030 8.15873708530199515110
O 13.21751511439754089849 11.04429859218648957153 8.58909495013813995001
O 13.24535802086931290944 13.25853341382369876555 9.73185432929420635162
O 10.98441879690192202190 13.69826180848068197804 6.26119423751041281889
O 12.91085297602120718352 15.02372885945421820963 5.57018106876394902827
O 8.69444930084980782681 12.68170290173264547207 9.40502388950988965632
O 9.07920812714575831137 12.24505631897857860224 6.98205453135054643354
O 12.05283721386047979252 14.87622612199430882640 8.24107278008422383664
O 10.79651694482813972797 16.43809703469490202110 6.85265339445183307987
N 11.06683436292570554826 12.34061871212344030369 8.65312565417216816854
N 13.31992408825141538387 12.98924172220040951231 7.05201025845478035592
N 9.49990968889727938063 14.64852682473044609424 8.12487289270722357060
C 13.06565830634336045080 10.44442971848043022476 9.82290933811752253746
C 13.85259789908315752882 10.87210034411224945927 10.87948009276913197141
C 13.60842868563191210285 10.41659251346426984242 12.20284870389621367792
C 14.27159921050082047600 10.94657236192121452234 13.32917293136415004540
H 15.02496126773608509097 11.70597134177995357618 13.18528987959461318269
C 13.95474779707739187984 10.52218731777499272084 14.59050673057864067061
H 14.46681323494245319239 10.93799972555326149859 15.44532315061528393585
C 12.96041487287191529276 9.55317381364538675825 14.79152893256238954223
H 12.71726670184360585836 9.24006762614410348533 15.79584838558768211669
C 12.29861303205983169562 9.02241501162848891227 13.72123967828710711103
H 11.52493468616476057775 8.28162260853123299853 13.86329184527562574658
C 12.60612036660258183929 9.43581925278085975606 12.40801003737148633377
C 11.96893672740621994421 8.88418723484336325669 11.28552270456139083876
H 11.26878672461260677551 8.07652276764262389008 11.44071176399664224732
C 12.18070331748537071803 9.34493264904992315678 10.00296461352914612064
C 14.58159958476111484060 13.01811496652900679294 9.97589879466011097975
C 14.92969785339794164258 11.84082561732856220260 10.59389092640532936684
C 16.30015768139042009466 11.56236952049274080423 10.85622976335962874828
C 16.73764318879168655485 10.34354352803905641167 11.41573945301398040897
H 16.00762846318761134512 9.58541579485353167911 11.65325677436297802103
C 18.06583780419753892943 10.11670136686488596922 11.65059229730979240003
H 18.38635395780799797194 9.17915427866125810397 12.07994936987643619375
C 19.02255502775801687676 11.09205909863539574189 11.33400819460334219002
H 20.06601771780498921771 10.89906617102063357549 11.53358809686733721378
C 18.63374214178749355142 12.27110548062193551289 10.76271660489368109381
H 19.36432652775410900858 13.02131910901599454178 10.49726656882478970090
C 17.27320592885677541517 12.53355517737783664245 10.49706643229317393207
C 16.86808519064091527184 13.72217643381688922943 9.85501902650226391245
H 17.61902076329078425942 14.44225529530673490797 9.56375780284937349052
C 15.54701678735516345853 13.97252723603461888047 9.58489382362802189164
C 12.77942033864340665161 12.58144283886528569383 4.68538647485892312261
C 13.85096133930361261832 12.74475573431696595605 3.82298094018351886092

C	14.2773133123137299094	11.7208452960754065941	2.9755722142615335101
C	13.6372564149130823807	10.4856910147868056526	2.9874515865792616864
C	14.0128190949291671074	9.3423129572878735871	2.0737791549493209686
C	12.5805295383173003642	10.3021833733929604193	3.8800362027351802396
C	12.1716109170846529252	11.3366280893138426932	4.7133087478044757290
C	8.3965495054569174726	11.3437023499913163249	9.5668812431643530658
C	7.5651545897744165714	10.7244499598895881575	8.6482825288263516228
C	7.3768085462747716718	9.3164046430850433466	8.6670642746708708159
C	6.6760707602383426717	8.6245328217879215770	7.6573326289416137058
H	6.2290727873660918234	9.1851075270720716758	6.8505540403642086744
C	6.5777948548433418096	7.2603178489286044339	7.6841697500017644983
H	6.0418240687137165778	6.7415039225402235700	6.9035358137221090046
C	7.1752339369426465509	6.5193041393177848519	8.7147332142345010908
H	7.0957900165415557225	5.4425071261990503046	8.7114413764263556316
C	7.8625599605907536826	7.1600429670290175465	9.7058142145924204413
H	8.3343667005497046318	6.6002074514641133617	10.5004923700254266805
C	7.9773289855374400758	8.5660510394875952755	9.7086833249072554963
C	8.6466551565275864277	9.2561934433899804020	10.7314169570747832693
H	9.0274420597412152744	8.6878535393838873801	11.5672443756732370445
C	8.8580512922825533905	10.6187140981319778632	10.7008436500139101355
C	7.7116671878130738093	12.3505690283863440726	6.8351193865573858232
C	6.9085208983969863538	11.5672653328331342947	7.6291543412310494076
C	5.4939859544750460074	11.6557973609248595181	7.5035533980648967400
C	4.6077451813600136532	10.9411801032876816464	8.3365484377505598701
H	5.0134605324122256675	10.3021107393521784700	9.1053650545601971799
C	3.2536599969501507345	11.0564389796482878125	8.1820084994461304006
H	2.5870734927278409287	10.5029621489208668805	8.8265226349806749084
C	2.7177066548483796992	11.8938825515856851922	7.1929187327982244681
H	1.6463362022881700141	11.9666111346033563478	7.0799192354239250946
C	3.5493711491910007183	12.6228472678493375269	6.3900217077770378182
H	3.1467265589347261922	13.2855251063716117698	5.6378427272067703768
C	4.9506406565934835128	12.5359342927922252641	6.5293063156590065432
C	5.8153621049757591166	13.3275028394616761318	5.7451871175951065851
H	5.3903510624120061223	14.0116368410794827070	5.0250634446072997008
C	7.1772458325620149821	13.2530604270260461419	5.8886922873890110707
C	10.3860969803627209984	16.5458263715203770516	9.4187608718245474648
C	10.5789411251277858383	16.0025013089770311580	10.6786762062763251890
C	10.0697726036747230438	16.6052299247888832667	11.8228344825550308883
C	9.3292333044883299919	17.7845265154802660845	11.7338666468073462567
C	8.8360686942509154562	18.4214081260437225751	13.0122749778410025101
C	9.1001289171551498213	18.3200698189219295386	10.4704886130660348442
C	9.6194236415411644713	17.6963893956493301118	9.3348719422024668546
F	8.3896848584789740499	19.4382140940635821380	10.3235058271721094058
F	7.9848005603956551112	17.6245235269699769276	13.6721113078358911252

F 9.85337177987069523510 18.67169360776952657943 13.85283543128626249086
F 14.43098263285781612808 8.27024818718036414111 2.75998419000099826093
F 12.96019129563052985077 8.94028135437302040600 1.34272781693488507671
F 11.94772433118574106459 9.12700872524013817610 3.91731704736598995709
C 9.51675242146362165840 11.23607853959105007391 11.86386380889271130457
C 9.08094561481052409135 10.92105214768320387009 13.17412932216416976416
C 10.61428869322959656074 12.07316976349805592861 11.70047680089125918812
C 9.83699464388005040405 11.38092527684581156677 14.28346064336830067987
C 7.88830924283782941586 10.18842489774885606835 13.44262713959269461839
C 11.32152414739970680557 12.55757882225041122126 12.77884559378754580905
H 10.93507504100447214057 12.32487546444282422442 10.70669311460622097343
C 9.46846078184175254933 11.01590396480836986370 15.60185550194561088233
C 10.97004395308164603762 12.20746811197647474501 14.08383515863537382984
C 7.53072060848933411137 9.84446846046965262644 14.70528524210220311375
H 7.24896730958803914291 9.92172582990429319239 12.61458065471073908270
H 12.17089887433258965643 13.20062778603820241585 12.60803562304288938378
C 8.32184360830298786027 10.21614128483602712549 15.82669675942644005318
C 10.24356321822943272082 11.44971515243821258423 16.70674607491546481697
C 11.71268898942892278114 12.65443051285589959321 15.21071452221693576234
H 6.62186391759822612357 9.28716770415417514073 14.88337662264213534513
C 7.98677393147983138988 9.84788529930438016891 17.13329829093346745594
C 9.87439474084655977038 11.05381054689576103556 17.99519352935774918478
C 11.37143937922357039838 12.28518278288790632757 16.46918346656586606969
H 12.56010571544739917726 13.30002684667557488751 15.03328101066330191316
C 8.76121271178259242163 10.25905308261565984651 18.20156134611255893674
H 7.11045906428424423495 9.23806658427410098966 17.29975527603211560290
H 10.46967194576058979294 11.38026693639965003513 18.83584982135221252975
H 11.94398535930650595560 12.62356011882882178554 17.32106186781239998140
H 8.49134025450644536193 9.96446804303359456867 19.20518020451487473110
C 11.50209903793557764118 8.64188692517118539627 8.90166448281769184803
C 11.53649248986320507981 7.22746650641836652085 8.83697040118630638972
C 10.76158009029064643869 9.34304232352015340268 7.95720206595805734651
C 10.73213973353197836502 6.55952773100644659365 7.87750224229741746740
C 12.37408210433630095793 6.43035165562769872594 9.67020202392818895021
C 10.01652882585831960682 8.69572457494496831032 6.99615957803767951617
H 10.74833700102347933125 10.41641848753775789760 7.99664653137486602930
C 10.68737769930652881101 5.14392606280825237519 7.84154121337879850273
C 9.96313698789104584819 7.30149023157576948506 6.94747476233129646062
C 12.33719832126521787075 5.07476318122006642142 9.63158301369511526957
H 13.06694745078030628349 6.92759975106415737400 10.33219113616547524259
H 9.45147118144437392573 9.27769014163845540111 6.28471553070565214227
C 11.47156313173767827607 4.38213827854020721020 8.74134008100741688452
C 9.85852547931370537526 4.47859543051873387753 6.90340795485631630868
C 9.16372538479339837636 6.61000680577387100811 5.99670594995773420521

H 12.98142136283079928205 4.49188066787651685274 10.27465830051680129031
C 11.39718439372286162836 2.98634139494679118343 8.70524139257045526108
C 9.81160978009273421208 3.08184626050308452250 6.90605803434206411140
C 9.10504473565386085454 5.25620495071802551479 5.97920873939935759722
H 8.60266732363411712470 7.19815599239273229415 5.28558967160431159016
C 10.57005149101562757608 2.34820994516535996866 7.80045429347628971328
H 11.99806287393287185239 2.40944815469355866355 9.39346668494795089543
H 9.17474672753058051455 2.57628195531180281819 6.19441043373345223699
H 8.49048641665773118348 4.73688575999930971960 5.25766228620729769716
H 10.52110809725066431497 1.26920073577092118455 7.78611514450166275481
C 15.09122639334400517441 15.15515877361532481871 8.83502270321818805598
C 14.32015854943290023016 16.16066881731616788898 9.45055663460436967682
C 15.40021223479497791686 15.24459591287261162051 7.48404905220506577734
C 13.79356745056215238776 17.20310675005861611453 8.65138959126705309188
C 14.07630267831381054577 16.18340513118002377269 10.84950108004978375220
C 14.88388221951623968664 16.25643279954513431562 6.70159610847598941064
H 16.01219889457431833080 14.47575274558268176861 7.03654976523152519974
C 13.01802592675999292737 18.22584722070982721220 9.24517292091992892722
C 14.03921696023777343498 17.22549991625601606415 7.25828027963709132564
C 13.37689663124875849576 17.19508950257655399696 11.42817337350018647157
H 14.47715234780632798106 15.38270280801584277697 11.45390414098508102825
H 15.10439079437209031198 16.29053294792684170034 5.64691158047828523081
C 12.82940927177356726929 18.25175176749961991618 10.64939893950075244788
C 12.44293616829068049867 19.23939062937596489178 8.43845350047194386889
C 13.41511880672235434986 18.22360424179575133508 6.46767036331270617211
H 13.21759259159106925097 17.21393184920520980086 12.49764409722241609302
C 12.11254401450955775488 19.30924479724746589682 11.22268291198236411788
C 11.71008341481266690209 20.26113168905838080036 9.05352457785496866904
C 12.63642556384572657180 19.18406263942233636044 7.03126230803119156576
H 13.56826218443568343730 18.18683199733433042411 5.40025080239079713351
C 11.56267688743884214375 20.29958364362206424403 10.42879691615492987466
H 11.99724829309806750643 19.34643572905633845949 12.29657858607133213980
H 11.26440291066955801114 21.03103437484776350175 8.44141702404497529244
H 12.15515764183339264548 19.93606827199869258038 6.42303978542868136969
H 11.00941022795666768275 21.10662920191596825248 10.88627577290167103286
C 8.11032519772881599351 14.12681833563535249709 5.15753553036443879165
C 8.99449874680401961768 13.60870513025181871569 4.19095942019069855888
C 8.13614243075852172637 15.47977317063369717687 5.46969535075619361919
C 9.96260845754978952016 14.46861000474444125530 3.62001916318074012935
C 8.93095644924126474962 12.26151731671807709745 3.74590153244044810066
C 9.08211358485865360990 16.31907905865682195667 4.91892288443693104227
H 7.43427803152294153222 15.86133862170300190542 6.19612291535540826715
C 10.86063782567770630294 13.97379589517960951639 2.64598452284911900279
C 10.03658218820866387944 15.82324326566123318116 4.02138865724646077382

C 9.75802408062843085190 11.80000415429071480844 2.77096613315592232851
H 8.19275895603362336317 11.60857522070310388074 4.18844021021131140969
H 9.11101999779743287888 17.35948479902127061791 5.19975180422989424756
C 10.74543269062785100232 12.63894366293612492314 2.18446578030170091012
C 11.86698218360695911144 14.81845814181438925061 2.11436669230179496992
C 11.08060406092639027520 16.63448492936375444629 3.50885715047165547276
H 9.68461250954421970505 10.77850332623597395809 2.42362436741802511619
C 11.60340180003822396770 12.18967515689533165357 1.17308785379706304042
C 12.72040344659660959792 14.31647933012420992327 1.12491119762835989349
C 11.96960077267750222063 16.14946848854840766307 2.60281459392229930216
H 11.15404953619817618460 17.64741134210260753434 3.87324335808788511315
C 12.57666149873130478909 13.02372496679564584099 0.65298830814748143325
H 11.49345014882380766608 11.18210518718819734829 0.79806606151359105183
H 13.49291939333152257063 14.95485812051524554533 0.72273130034891153173
H 12.76915936229904069421 16.76892007857377819846 2.22327461584407082285
H 13.23295016404833468471 12.66076215998889686887 -0.12435126356355752364

6th lowest energy conformer of **IDPi-4**$^-$, computed at the GFN2-xTB level
S 12.41261423316009171458 13.78136488273900894796 6.05297205413044636657
S 10.80327991866349535144 15.51300866989511817451 7.93514941913658145012
P 12.61996441740352636884 12.56389355113313577306 8.43440738963458080946
P 9.73626978744753124317 13.06493897554229377533 8.19058239711275781758
F 14.53954240904230132969 13.89266885212279412087 3.78548196267470249410
F 15.33495425744522222544 11.91164916545857366259 2.18652755299907397557
F 15.41429033187938557603 9.04168285642905544819 2.32586963072324603985
F 11.16674861994520995268 11.09788084950770858939 5.53437290092358313842
F 11.27248791621272516750 14.88679472958885163791 10.83454031014536056432
F 10.30485814325868965113 16.04412542711862954548 13.01764663247895725817
F 7.39757966718128301409 18.53201481417053031464 12.89950571436788884228
F 9.34553586047078788113 18.25316795544600978474 8.15139240100660344979
O 13.21352717513511088043 11.03765450291224681223 8.58814925435750176064
O 13.24862877826655349622 13.24969937847045642343 9.73532803418982695121
O 10.98894940258188235305 13.69450983592428627844 6.26357517127381413502
O 12.91515776247337754512 15.02046479206782514382 5.57370079054345346492
O 8.69616335175869892282 12.68625810134143527819 9.40724816318413559202
O 9.07672129143093897596 12.24883648899906951613 6.98365199544793391340
O 12.05627441930549714755 14.87217343187468365784 8.24818807323389791009
O 10.80806947238062321048 16.43773859987874885746 6.85695863420244933195
N 11.06669767981327012762 12.33908046618204323863 8.65374896344844302121
N 13.32327110364961342270 12.98652344073772191280 7.05632667695588811085
N 9.50408014172038129175 14.65049598853626200423 8.12470378136888093934
C 13.05989270377442679205 10.43617530139755089635 9.82101545441581791351
C 13.84848136423124209671 10.85984251041660364479 10.87797232194020935481
C 13.60335160941162868653 10.40337882972975513951 12.20071575605225788763

C 14.26902972947162773210 10.92936391050349698162 13.32742308107423312435
H 15.02501400180894286507 11.68626914792882232064 13.18409791355706239813
C 13.95140873382955604143 10.50413441978286499534 14.58826240583518085714
H 14.46538955103073575970 10.91678880722643718570 15.44345648217290545290
C 12.95383601474371282336 9.53822860414958739739 14.78830403009355975996
H 12.71017190185015977022 9.22440515001297001163 15.79227438529858673633
C 12.28960780086184456650 9.01137275665688441961 13.71759220681684965371
H 11.51350183504510837906 8.27299656517894810293 13.85899453766452893433
C 12.59783518967032023284 9.42568614250149927614 12.40481138431309027226
C 11.95856363847440562154 8.87767652196466627856 11.28173627252666655352
H 11.25593347069742122812 8.07203506437017637154 11.43609681490954699257
C 12.17148223880803215025 9.33928201054105322498 9.99963452729476820480
C 14.58485252842996615641 13.00368800329085239298 9.97567717998734337925
C 14.92912916982823290368 11.82459464393110870617 10.59229464545490095873
C 16.29885902591131596751 11.53958097182898612232 10.85141437792822571851
C 16.73186112067816821991 10.31910828055080742160 11.41079158735903575916
H 15.99880417385370989791 9.56478300580287310595 11.65102893143427742473
C 18.05956545807303825768 10.08597189941179905759 11.64228151105317010661
H 18.37662546354551551531 9.14725531264577895740 12.07164995103461180292
C 19.02021933059171843183 11.05631534517990743893 11.32222086618474499176
H 20.06325576685696887580 10.85832734281423306300 11.51908387596480309867
C 18.63566640513155903136 12.23676689248896920503 10.75096415057685206307
H 19.36920680111433767934 12.98311429271553407716 10.48281573556211654363
C 17.27570187176528548889 12.50567635099827157319 10.48881686185002592993
C 16.87465054523794094621 13.69565668912782996358 9.84682973142002282430
H 17.62835418569846268610 14.41156597501751157608 9.55247491275112636799
C 15.55398300446953996357 13.95285305090193439526 9.58086934972414994149
C 12.78991625335223858428 12.57745626515142944868 4.69056908628557245322
C 13.87365370198311431693 12.74089585209019936940 3.84109522263181268897
C 14.30237186562237283738 11.71121942363505219475 3.00748402772374134884
C 13.66200604441201527095 10.47267420356675238224 3.01048967823352775497
C 14.15313438696803771677 9.40648089315921076548 2.05880583333331479423
C 12.59482621675524782745 10.28932324680896925884 3.88577004195937725228
C 12.18267634182540781751 11.33372774391476234257 4.71048662812173724035
C 8.39517074229293669418 11.34891696414397443959 9.56908365052295906139
C 7.56119783380153798191 10.73219949973368159135 8.65109836483580174615
C 7.36947657327859495524 9.32470942969876404050 8.66917098960104937078
C 6.66564106822482305148 8.63527530566269518886 7.65993424562685376600
H 6.21893494859718298784 9.19760136554880780579 6.85420205720789965653
C 6.56390912110800783807 7.27131572641539936086 7.68611904675280754873
H 6.02554579606336293551 6.75427462283895785333 6.90595919288820958570
C 7.16084995820852832082 6.52820469474037778923 8.71548469511798096221
H 7.07862744851293790305 5.45161825961505286386 8.71169159825962857724
C 7.85115593544198731024 7.16658862517291694871 9.70601124144672944283

H 8.32253790662892178887 6.60504886185490658335 10.49974406420734673873
C 7.96947644985281922203 8.57230767455689957046 9.70959146216399737739
C 8.64162748462962326812 9.26023062692788911932 10.73196869266560682377
H 9.02188837441717517152 8.69041826294062147440 11.56700470348552300948
C 8.85626039753437410695 10.62229875809612700266 10.70213227594577531931
C 7.70901676633507371150 12.36024628150099502477 6.83995908021483334238
C 6.90501185702443986969 11.57809087370408995810 7.63421744696207582592
C 5.49045264727890547363 11.67204933872588767940 7.51276667694853550472
C 4.60383293463979370586 10.95787279880137532473 8.34570778125564416428
H 5.00915604591582308558 10.31458200036165706592 9.11120182136526324257
C 3.24975698784360478300 11.07863766698168639380 8.19521027231507837030
H 2.58289905057645263753 10.52533700424737439505 8.83959586795176832652
C 2.71419673466424349328 11.92150858190696816052 7.21053361585701235725
H 1.64280487552336573742 11.99860550185768737208 7.10074897933953330664
C 3.54637361328199141042 12.65019267804443714454 6.40791868172395595593
H 3.14416741066544602745 13.31701095141408686118 5.65918467293648497218
C 4.94768841877979781430 12.55754560460199797944 6.54305013273638458315
C 5.81318928696241332688 13.34875281678753466963 5.75949975925072621408
H 5.38875384201001939033 14.03733960582188444732 5.04330121831204714056
C 7.17527054707320210980 13.26834513486458178022 5.89841956226611152658
C 10.38575717595976577456 16.54551422192202281281 9.42096187273906515713
C 10.57500099527857884141 16.00791123949256089531 10.68241130008001249507
C 10.05728907658118131963 16.60162652779071379427 11.83144440787994255970
C 9.30756002361045098326 17.77072967182857610169 11.73379510580398310537
C 8.73664252121681705887 18.49852802872364776476 12.92878289070607422673
C 9.08224630027132562304 18.30132041485894234256 10.46419698569688883083
C 9.60907081960105102780 17.69054682963000146856 9.32913788959270995349
F 8.35803013082074031104 19.41508323688549353392 10.33999399033765875799
F 9.07969466357713272942 17.96261741885876972447 14.09849051138279563133
F 9.14965032133465783204 19.77504096913664355384 12.96658662218667146249
F 13.42207795035561801456 8.29357374523350365791 2.07545067776231739742
F 14.14259099641986061613 9.84208727966589336233 0.78933884763859119005
F 11.94375762297067211648 9.12629664923507277763 3.93742777709734248504
C 9.51763415969075055045 11.23741977054702090300 11.86487055047590288837
C 9.08443320576372492781 10.92022957189013609991 13.17547935810083004071
C 10.61500719369126954916 12.07471102729665268782 11.70091748564003708566
C 9.84334152900819425724 11.37718851263821129294 14.28409608207050318640
C 7.89160598458569850777 10.18824666337695816765 13.44525668450422983824
C 11.32498559268318949478 12.55637919670036950492 12.77871930561591895525
H 10.93382851035955916075 12.32870208014143997843 10.70708835755985077753
C 9.47769141318906704896 11.00935711601523081526 15.60254319113174759082
C 10.97647926723625211309 12.20342861819844948457 14.08369498213675186093
C 7.53673292716058895735 9.84173675367117084534 14.70793539979157316111
H 7.24988131387902257075 9.92426439404543359046 12.61817429950762914359

H 12.17411353705806753567 13.19953065742649833680 12.60718271332986617494
C 8.33104714150053027311 10.20988575927333208426 15.82830762965456372626
C 10.25580594212387985920 11.43996101465200787572 16.70657651850636682411
C 11.72220010229860598372 12.64710829084889098795 15.20986653450907688523
H 6.62770855954399529253 9.28499802987751721162 14.88695011503652665397
C 7.99894093403856754065 9.83868484471228654797 17.13479680084388689920
C 9.88954788859663658229 11.04117405566240250891 17.99493424489053339244
C 11.38372034382443054312 12.27519550826417216172 16.46824184969226223529
H 12.56967482402731306479 13.29243717660494716881 15.03185520551025788905
C 8.77634410877920601024 10.24666063682116501354 18.20213546043192920365
H 7.12264231290499783000 9.22901936692213276103 17.30192418333010095921
H 10.48718546448320765307 11.36509959258374991009 18.83489204531338145898
H 11.95861240470952502335 12.61101331186303120546 17.31954369668955706629
H 8.50875564349097146533 9.94980483223777234514 19.20570032072337696150
C 11.49053031186587325863 8.63974226627253472088 8.89750172418252738282
C 11.51840914381324232352 7.22518855567237494597 8.83245265337339802159
C 10.75433587097707999192 9.34441852480518519997 7.95220227459516681989
C 10.71123385588583332151 6.56116153885025887860 7.87260228917027404805
C 12.35232993902124043473 6.42391009807491641936 9.66547695055095168470
C 10.00659866186315660741 8.70073932153845674975 6.99079901596443598066
H 10.74630344116040348013 10.41785814467396953376 7.99119667916421327902
C 10.65960638780532576675 5.14577400172251131494 7.83659102655456951680
C 9.94617973248394982022 7.30683251304240144464 6.94228579055638750361
C 12.30897113725773017734 5.06856036493752792893 9.62672549132144439454
H 13.04779595551391757624 6.91772760131097541603 10.32731682562599218045
H 9.44498733882807428586 9.28557308105906287210 6.27901293470171317779
C 11.43988529944016896422 4.38014349587402307407 8.73652642158643999437
C 9.82771388344067098330 4.48450691606032947334 6.89827443306206511409
C 9.14354866629994589289 6.61923142379605344843 5.99138920925489060210
H 12.95051138270665447294 4.48254848026879848533 10.26964515889264362158
C 11.35867841354112250940 2.98476523324839071449 8.70046006609479860572
C 9.77394432632836007713 3.08803531203606240751 6.90096270803086220269
C 9.07823530099576991859 5.26578771207498874674 5.97388627716009246171
H 8.58550044145918533900 7.21019954083397074385 5.28027210559659465616
C 10.52857858910116206630 2.35070260200935265971 7.79553228349469318204
H 11.95655292044296125198 2.40494460404053711500 9.38884506573800337037
H 9.13471935449710414900 2.58559540475332205745 6.18922460141576280535
H 8.46128543981459912970 4.74943189633085882662 5.25226529053269075575
H 10.47433143363982566143 1.27196060505070995816 7.78122028863926917808
C 15.10225225643363700101 15.13764365159169322794 8.83236024662941510144
C 14.33171217479144843310 16.14286513358669949980 9.44903776495923786172
C 15.41300932336584850191 15.22963976490439996780 7.48189723040039211810
C 13.80531566335772453158 17.18644543036658944857 8.65131229813320601352
C 14.08787518398426286126 16.16343227374309066136 10.84785959978092861888

C 14.8983645195813956263516.2435735999512935734 46.7011292218779310303 5
H 16.0246278777526605097114.4610843456675652873 97.0334086765806453911 4

H 13.25679129221624563684 12.64461140187845167304 -0.08219148554919186722

IRC end point of **TS-3a-1**

C	2.32184097589668	5.97536658206415	-3.57357614363946
H	3.41679783515815	5.81429888254104	-3.56774486200264
H	2.00141937722293	6.64124059759873	-2.75183745389128
C	1.60229618921965	4.73036739998427	-3.57237106248800
H	0.57328714424527	4.62409079748931	-3.18567059403810
O	2.17879980655729	3.69131700024710	-4.09750547054446
C	1.35373192093128	2.49276623964755	-4.36511865327649
H	0.55354129569696	2.82482241743029	-5.05799912155198
H	2.03772333676361	1.80815514340408	-4.89047140401188
C	0.79734939513915	1.90454313138166	-3.10298798271222
C	1.57365885113728	1.01419346041162	-2.33701232802713
C	-0.48599817409551	2.26979754156863	-2.64719347159802
C	1.08677355374157	0.50215041179040	-1.12867841865900
H	2.56712474960175	0.70905152076900	-2.69880419271421
C	-0.97211473214159	1.76981389618770	-1.43405817470305
H	-1.12370920840211	2.90551342354556	-3.27792837250605
C	-0.18688711042909	0.88528736060088	-0.67515060178547
H	1.69080537649254	-0.20338151863484	-0.54195047961130
H	-1.97406423794941	2.05001430144539	-1.07876458619199
C	2.03882103685831	6.85617617559712	-4.99634786081191
C	2.35610708186038	6.05981136493234	-6.19904226917045
C	1.54026045649599	5.12009040626973	-6.78458673541167
C	0.68361239264406	7.47836885083280	-4.96239874583719
C	0.20198553976184	4.76811845651054	-6.45138865370980
C	-0.50133177198341	6.86653527688770	-5.21742927337567
C	-0.71789624917681	5.50828491560047	-5.68669472934841
H	2.80147695983636	7.64715501608899	-4.87910584189195
H	3.37336591806313	6.16201807501241	-6.60153184345805
H	1.98379565521510	4.51733284935260	-7.59533373403429
H	0.63939245057851	8.52948787744606	-4.63032965860916
H	-0.20532652602281	3.87960805193626	-6.96424226735558
H	-1.43914101939942	7.41717673035389	-5.03400964498260
O	-1.95169791174157	5.06266048094859	-5.46108593088543
C	-0.76509386060634	0.29649553620692	0.58970650800629
F	0.19133630136060	-0.17121824675203	1.42415298109488
F	-1.59895466636228	-0.74334268961085	0.31120798970719
F	-1.49657361610408	1.20789481791280	1.27480717646590
H	-2.02379367924800	4.07122695144442	-5.66982154548766
S	-2.53480534327867	-1.72921721804371	-10.79332044305424
S	-1.63517223637233	1.43288164670921	-6.50359026018087
P	-0.12947169548849	-0.89118935344755	-9.44235997689333

P	0.21546459194058	-0.75440670695411	-6.43857846700523
F	-1.20576986452718	-3.46047498270915	-8.40625756701214
F	-2.22611896097059	-5.57165480482315	-7.26747455662548
F	-5.19778517826945	-7.78570415510844	-8.96425789902280
F	-4.63414326499309	-3.59251824530528	-11.73882311039268
F	-2.59871432868663	-1.51262028443685	-7.03675995226138
F	-4.80524436755174	-2.24766360659908	-8.23678818084878
F	-8.03573109304728	-0.04494226366726	-8.39342504989677
F	-4.17385394770575	2.99709264413753	-6.87983584654430
O	0.52322102247601	-2.11501429714291	-10.34150436673579
O	0.56150209461543	0.44264830677850	-10.14174239582711
O	-3.86880833941881	-1.08263628188812	-10.86922981338587
O	-1.78399853267185	-1.95414783108187	-12.04744837256203
O	1.64517165079346	-0.34689863161637	-5.68032397287465
O	-0.05533361291653	-2.24993758838957	-5.78720528881954
O	-1.91205224329903	2.48892625314734	-5.46477921311543
O	-1.03027729523632	1.92872496337357	-7.76839101117743
N	0.48491224046403	-0.91513237364105	-7.97663389352619
N	-1.71295963801832	-0.96897148741277	-9.61881956354382
N	-0.82913752396435	0.27610571644608	-5.76670102889020
C	1.84778999024142	-2.11753936786518	-10.71402700130373
C	2.31583236834705	-1.16484379205516	-11.62295555165227
C	3.73348208522831	-1.09428047445059	-11.86068548023976
C	4.32516001973492	-0.06937150608063	-12.65708618140215
H	3.67157039151314	0.67285955427658	-13.13721453744926
C	5.70136192695611	0.00603611207842	-12.81731693665744
H	6.13628311943581	0.81164040660026	-13.42797358227320
C	6.55270015066082	-0.94680696739362	-12.19796349036588
H	7.64239506634031	-0.87949974088777	-12.33515311207214
C	6.00912356747672	-1.95658318924649	-11.41896748800720
H	6.66008683023565	-2.69989821535249	-10.93263149173397
C	4.60008374872552	-2.05664113231251	-11.22195781224910
C	4.03325451069227	-3.09246739384240	-10.42480141250592
H	4.69152936682185	-3.87105044904116	-10.00941685117209
C	2.66651430906807	-3.15716667440415	-10.17339182847070
C	0.46994717689071	0.51414754679289	-11.52977441719517
C	1.33538988369951	-0.27063215595448	-12.29624799026874
C	1.17605145973298	-0.28025369439656	-13.72712078963943
C	1.92913317410741	-1.13742231408933	-14.58384320397032
H	2.66682684706022	-1.82076932224382	-14.14211495202597
C	1.72954403042939	-1.12897448608535	-15.95674611121700
H	2.31761071704634	-1.80461231355690	-16.59617639554159
C	0.76680245477806	-0.26517092796914	-16.54202803443810
H	0.61932274523874	-0.26612928209603	-17.63250110650207

C	0.00202066240958	0.56241100608322	-15.73527704262374
H	-0.76337199947899	1.22144640591941	-16.17358391247171
C	0.17389035962246	0.57043908209163	-14.31983331314305
C	-0.63466952723118	1.38900344684151	-13.48359345771807
H	-1.38661704259104	2.04624155644577	-13.94618297686359
C	-0.51315149696701	1.37629908683413	-12.09958362623152
C	-2.92436558692102	-3.38470050017578	-10.08571793316706
C	-2.30816261865974	-3.96334944334537	-8.96688309017314
C	-2.85188075812916	-5.10648533245631	-8.35399133908183
C	-4.01182042098604	-5.72355948047582	-8.85183105947926
C	-4.72897545502483	-6.84081413667371	-8.12472123229980
C	-4.58389947123697	-5.17774666327238	-10.01769523586024
C	-4.03473768538602	-4.04880370127655	-10.63979280277051
C	2.68032223323257	-1.26901886971551	-5.66705378935193
C	2.58757331005785	-2.38401401720548	-4.82841639487941
C	3.63960087544177	-3.36723504935553	-4.88051849689332
C	3.56503892701141	-4.60970412841776	-4.18213468969183
H	2.67223321882126	-4.82790402720863	-3.58051286146575
C	4.58881423050956	-5.54194309927516	-4.27086784900021
H	4.50311980582655	-6.49756594762320	-3.73214573686869
C	5.73951109311837	-5.27894140714024	-5.06030300622874
H	6.54478246989151	-6.02607728514610	-5.12178198371913
C	5.83673050344554	-4.08888001008306	-5.76375710793395
H	6.71525444252020	-3.88087289947342	-6.39406234175939
C	4.79762365776367	-3.11376723238253	-5.70359953251493
C	4.87102920820636	-1.90778691974093	-6.45793448202177
H	5.76829160104107	-1.70509165220841	-7.06236762066967
C	3.82901613870040	-0.98918655921217	-6.46507503980185
C	0.12001915252301	-2.44404217303437	-4.42280069528052
C	1.42300332551524	-2.51136305194331	-3.91333211948007
C	1.60555309598275	-2.64825235699371	-2.49024264984707
C	2.88472442980646	-2.56648734302159	-1.86134947330630
H	3.77514845091238	-2.39074938904568	-2.47988758780540
C	3.01607723308303	-2.69599455107212	-0.48520389521294
H	4.01311123879635	-2.62279732859980	-0.02495758097383
C	1.87639071795232	-2.91993365114491	0.33192564762398
H	1.99145086097342	-3.02403023051126	1.42093271619241
C	0.61718592354815	-2.97615480108914	-0.24436665300114
H	-0.27777129777330	-3.10931283227791	0.38063503913845
C	0.44613292306265	-2.82196813148699	-1.65158735961184
C	-0.84884148834313	-2.81141043964606	-2.24009136917869
H	-1.72867286979834	-2.95056410883376	-1.59451981830362
C	-1.03981928645925	-2.61229475602672	-3.60218475938981
C	-3.25939952213941	0.78621395629695	-6.97671435083251

C	-3.48382599057442	-0.55574483618298	-7.31542519119883
C	-4.67129486364655	-0.95220270287110	-7.95370889934585
C	-5.66693313996197	-0.01934998538777	-8.27219153097824
C	-6.90412089198452	-0.35240108717192	-9.08276090753558
C	-5.46905465541177	1.30970242322930	-7.86021079549729
C	-4.29082944211785	1.71059667302303	-7.22269668266717
F	-6.39939505837732	2.24169952367454	-8.11457529974520
F	-6.92275438285468	0.37832868477860	-10.22083078438910
F	-6.98142522334837	-1.64827819578661	-9.41917285377357
F	-3.95436350000097	-7.45719618428742	-7.20931888060074
F	-5.80862509081365	-6.34075674231129	-7.45184343938790
F	-5.68585337507176	-5.71913610413324	-10.54915724392985
C	3.92435740346046	0.27706178888885	-7.24825971635525
C	4.40603017679863	1.46668269493264	-6.63344608911277
C	3.57452527488226	0.28713820683293	-8.60819077286114
C	4.56768375977100	2.65063875147521	-7.43184954569266
C	4.75747876774828	1.52878461890838	-5.23811880926950
C	3.70157844962096	1.44399603378389	-9.38170907436432
H	3.19267989000652	-0.63485533509738	-9.06173481562169
C	5.09943376105486	3.84386020229469	-6.84761561856091
C	4.21606059106666	2.63255689506868	-8.82423329310822
C	5.22763335275521	2.68751039231816	-4.66774998543187
H	4.64366494062197	0.61839217278994	-4.63033553341583
H	3.41555790754973	1.42123421596930	-10.44285667127925
C	5.42194704431195	3.88023049867671	-5.44774384610767
C	5.31643673043885	5.01036467216495	-7.66143039707362
C	4.42287174794018	3.82065083155351	-9.60825066674054
H	5.47746336867849	2.71651697783872	-3.59573798345106
C	5.93371789197336	5.07544048059954	-4.89087886530744
C	5.85194867752646	6.17481223276499	-7.06248853753584
C	4.95879660768537	4.95843356234702	-9.05506957830891
H	4.15366225714052	3.78961421597243	-10.67538254137019
C	6.14890183023113	6.20605989597817	-5.69234145096227
H	6.18560119757925	5.09886793087126	-3.81880379631254
H	6.02563913630726	7.06417426488068	-7.68789123362785
H	5.12368884494422	5.85528472737404	-9.67225976534145
H	6.56378837468422	7.12216227711849	-5.24491158877211
C	2.05148028236553	-4.26347641150149	-9.39756747531218
C	1.00804480171697	-5.06453019420890	-9.95537811446562
C	2.52328158088027	-4.54242081839499	-8.10149340073825
C	0.46492384626238	-6.14232639868362	-9.17398320113741
C	0.47042111823212	-4.84659954582729	-11.27364613216225
C	1.99353261169858	-5.58631363557486	-7.33914952866040
H	3.30900573096973	-3.90346101003152	-7.67587665822283

C	-0.58674037873988	-6.95818317835355	-9.70448598389460
C	0.96636587097676	-6.40541116594329	-7.85302660270893
C	-0.56121333754560	-5.60596892324813	-11.76745119706387
H	0.87819509839140	-4.04041200206548	-11.89645232259208
H	2.37839418944139	-5.77099306260496	-6.32519699247774
C	-1.12588007773072	-6.68563416582855	-11.00783209282949
C	-1.12326764215270	-8.04219617619654	-8.92833964614660
C	0.40621935734640	-7.49363669553495	-7.09733252095996
H	-0.97313802610547	-5.38687105646908	-12.76421415477577
C	-2.18330536025853	-7.48444261147059	-11.49988310071493
C	-2.16998524591241	-8.82149363778143	-9.46911711716979
C	-0.59170190924148	-8.28246088802339	-7.61326312203279
H	0.79956924714196	-7.67986184506459	-6.08572732040543
C	-2.69558024928057	-8.54158941042973	-10.73784950269018
H	-2.59887996275358	-7.26279084432665	-12.49497815834487
H	-2.58682391562655	-9.64272582100257	-8.86673014818965
H	-1.01622818706057	-9.10805606497944	-7.02159059823782
H	-3.52448624929850	-9.14840533347262	-11.13110947778208
C	-1.38127936866476	2.25231890071754	-11.26071239166436
C	-0.86243608556276	3.44846306065613	-10.69157924602011
C	-2.74302269696573	1.93799798889445	-11.10342321480564
C	-1.74859570420551	4.33015472031257	-9.98411798742281
C	0.51685495140092	3.83400907170163	-10.83149258629070
C	-3.60190644920151	2.78077306048791	-10.39018710755290
H	-3.13738614817302	1.00149476399541	-11.52114231518154
C	-1.26067538386868	5.56849190835703	-9.45914083759561
C	-3.13361631627850	3.98611900843687	-9.82513078064210
C	0.98362882004557	5.02620459549013	-10.33269709529832
H	1.19861685482313	3.15481357314006	-11.36395499796058
H	-4.66196100404309	2.50495847484080	-10.28216190573067
C	0.11453005728572	5.93957079073572	-9.64326271186998
C	-2.14900561182794	6.45531038640494	-8.75678549750697
C	-3.99712968854727	4.88745931835485	-9.10959236764908
H	2.04157431387336	5.30437991885880	-10.45446028342782
C	0.57038764874587	7.17676823144952	-9.13422567150056
C	-1.64438370405571	7.67892073440835	-8.26282287836588
C	-3.52392919459418	6.06680204225317	-8.58955144313305
H	-5.04971740212690	4.59795873328255	-8.97288132600488
C	-0.30138539889649	8.03362884459050	-8.45236373187749
H	1.62725679412093	7.45266166673855	-9.27229491432404
H	-2.32726513622275	8.35739877561578	-7.72775317022744
H	-4.19559158086290	6.74258146274324	-8.03790138177532
H	0.07148004600937	8.99022151487194	-8.05578715043670
C	-2.41575604381161	-2.66522627604832	-4.17943640581292

C	-3.31222552380892	-1.56579885102136	-4.07872510210661
C	-2.84049020068550	-3.85673541311955	-4.79522261816727
C	-4.63701664287702	-1.68984137869376	-4.62602040389174
C	-2.92639273641206	-0.30668232280803	-3.50024955255102
C	-4.13811587586996	-3.99314115039974	-5.29652211714743
H	-2.13340776529361	-4.69325223275897	-4.88446893157928
C	-5.52595058668515	-0.56691122869352	-4.61909843835192
C	-5.05658875707661	-2.92411576484262	-5.22781260339743
C	-3.77853283927829	0.77025634998076	-3.50275324857241
H	-1.91388547275723	-0.20714896944801	-3.08844630158268
H	-4.45474782411429	-4.93955062021359	-5.75587207091369
C	-5.09359388317867	0.68970904699684	-4.07482219320597
C	-6.83294080949206	-0.67632998087282	-5.20616331735348
C	-6.38068519188027	-3.01565267095212	-5.78379807968014
H	-3.45078409956047	1.73624940544155	-3.09295762638824
C	-5.95659674326404	1.80636118464437	-4.14649125042574
C	-7.66496668346774	0.46535064108707	-5.24528879646970
C	-7.23217097018630	-1.93800825620558	-5.77414905465031
H	-6.68120588794415	-3.96459541745872	-6.25316003279453
C	-7.22564981976103	1.69213639142823	-4.72922450159011
H	-5.61054332961054	2.77291331646262	-3.74921951139209
H	-8.65751821256796	0.38482681787200	-5.71248800541887
H	-8.23035976850731	-2.01236485689869	-6.23138378478613
H	-7.88181674446485	2.57359063523538	-4.78739329507280

IRC end point of **TS-3a-1-RRR**

C	-5.10001607101462	-2.61556949474086	-1.55415200266287
H	-5.02382725858952	-1.66008870637008	-1.00194034060319
H	-5.03882387204077	-2.47457846090730	-2.64872043814168
C	-4.16216072267025	-3.59881311244013	-1.09408688676670
H	-3.82590231207634	-4.42140386583362	-1.75356284249195
O	-3.77613360949851	-3.54138493469465	0.15258593014936
C	-3.09187728615965	-4.71856521685828	0.70712784767603
H	-3.77101520715400	-5.57597592137159	0.51015984570685
H	-3.06722829126801	-4.51188168582750	1.79193825096495
C	-1.71196993977909	-4.96263046890877	0.16296637417165
C	-1.51403736484620	-5.73692694273622	-0.99945021540923
C	-0.59178960628934	-4.44333516848873	0.84531843011563
C	-0.21643783006278	-6.00186213410014	-1.45721342694078
H	-2.36676157280397	-6.15707730097641	-1.55385650593421
C	0.70572616267363	-4.72249585609550	0.40213768036683
H	-0.73932681040565	-3.83247147123940	1.74953958933968
C	0.89112175792684	-5.51612948281328	-0.74341820255506
H	-0.07142466613178	-6.60416135434874	-2.36302198713633

H	1.57771937483049	-4.33582677585725	0.94784184004156
C	-6.71957779273507	-3.13356673862877	-1.29126899844873
C	-6.96703549048645	-3.52250440375548	0.11204109628690
C	-6.65111711452047	-4.73971131896124	0.67516946318091
C	-7.10051596184038	-4.13770563974745	-2.32178858569573
C	-6.09747562098769	-5.88813500262241	0.04771781379415
C	-6.76182838315568	-5.45312253049875	-2.34629972385104
C	-6.06613932346600	-6.20179133678826	-1.32255031358059
H	-7.24043112850780	-2.18187181368044	-1.50703592570963
H	-7.37510233801946	-2.74772317689249	0.78077534734971
H	-6.80674159037495	-4.83374810795648	1.76191861334815
H	-7.63716135841308	-3.76278612995331	-3.20786975706738
H	-5.75769809100264	-6.70939731308419	0.69971166465007
H	-7.04371116273878	-6.02926215303598	-3.23927768995718
O	-5.48804605010672	-7.35139547051839	-1.64870472718862
C	2.27967808512522	-5.93957886752624	-1.16296378504555
F	3.23700229982685	-5.12965765158050	-0.65810913883628
F	2.54983404628798	-7.20091768404012	-0.72478590464985
F	2.42160114106168	-5.95745674771947	-2.50996204415391
H	-5.35455866668319	-7.44421749570383	-2.65346417348844
S	-4.06481940231504	-9.35548794067569	-8.76320602626068
S	-4.35533272485742	-6.05848524718635	-4.61134596828705
P	-6.27800069534594	-8.47918322989836	-7.12511973195689
P	-6.65664534420938	-5.61487158492527	-6.25343421966655
F	-2.52013579615729	-9.64641884444877	-11.27024946844141
F	-2.03854095935818	-7.86602186579715	-13.17804421873748
F	-4.22314363044239	-4.36302899333742	-13.52402993661390
F	-5.90297156527838	-6.77756238062362	-9.48672255721502
F	-1.71020254161728	-7.12940860218856	-3.82664449204356
F	0.45207463476752	-7.57753713509391	-5.30934232248804
F	1.62579330255033	-6.96064355055526	-7.69497644043816
F	-4.06420799871663	-5.76174908171852	-7.75262120664667
O	-7.15752525575192	-9.16937805704818	-8.34035641416378
O	-6.61874143247036	-9.47201174110313	-5.84302070076703
O	-2.60576711821568	-9.37606767253197	-8.50692753159414
O	-4.75073338946653	-10.62646841465597	-9.08475212970798
O	-7.93461699137353	-5.09289953993887	-5.31222022901640
O	-6.80106422940458	-4.59670222886952	-7.54669242101616
O	-3.76458592067558	-5.29586952768794	-3.47864254086506
O	-4.93086310851251	-7.39953371900016	-4.22955256936747
N	-6.93930160381466	-7.08962117739172	-6.71351830861959
N	-4.74522818406990	-8.49932757372402	-7.56566434760083
N	-5.37704339298182	-5.12978384642488	-5.39565301628104
C	-8.45263175454378	-9.58885377504555	-8.11923738183303

C	-8.68104367940690	-10.69524093279128	-7.29945666908628
C	-10.04080852837505	-11.01220729138531	-6.94839130869026
C	-10.37160086479276	-12.02752758848109	-6.00322001456485
H	-9.56056001751701	-12.61375665788365	-5.54882795905563
C	-11.69082713813121	-12.26950540219623	-5.64760725967757
H	-11.92164175081147	-13.05126223174102	-4.90837095699361
C	-12.74432064522600	-11.51375300616587	-6.22643213760506
H	-13.78696254482097	-11.71769741484888	-5.93980282053585
C	-12.45628510998343	-10.51656286352764	-7.14532923538592
H	-13.26488750807504	-9.92050539666079	-7.59670888807958
C	-11.11086842893827	-10.23133835362376	-7.52343502174662
C	-10.80611961024903	-9.20030650244161	-8.45742719126548
H	-11.63169506083324	-8.65752902643169	-8.94302601883984
C	-9.49410199823452	-8.86950116786779	-8.78318900756730
C	-6.52103863938357	-10.84458923941075	-6.05155841081562
C	-7.51984301718124	-11.47883708827495	-6.79637894577443
C	-7.36414759386308	-12.87204854526520	-7.11991561242129
C	-8.26659961599018	-13.57127072163596	-7.97530984444218
H	-9.12168268528150	-13.03123920419919	-8.40436668954633
C	-8.06709088505119	-14.90980269869610	-8.28073016353916
H	-8.77184532410560	-15.42654949632251	-8.94963852497767
C	-6.95687602636966	-15.61547236373855	-7.74594073928143
H	-6.81067349037816	-16.67745847605612	-7.99435562497470
C	-6.05239448173855	-14.96039962948993	-6.92537700193150
H	-5.17847615734225	-15.49140493312265	-6.51725243560446
C	-6.22196349845956	-13.58219717448760	-6.59986363537569
C	-5.29381076687222	-12.89955547445289	-5.76744713137136
H	-4.45351039386188	-13.46235481388207	-5.33309709775718
C	-5.41696895103155	-11.54430858196114	-5.47565590000894
C	-4.17049535097295	-8.25891918128362	-10.24572448751970
C	-3.23932484097543	-8.52389126242204	-11.26772583521394
C	-2.98906135363401	-7.58706964220846	-12.27900325307653
C	-3.69575891894626	-6.36984497954352	-12.34146780052961
C	-3.28554803894451	-5.31611957094608	-13.34725168601969
C	-4.68524093878527	-6.15062890300945	-11.36884559719609
C	-4.93185326360904	-7.08661754248509	-10.34922190413898
C	-9.18263086357477	-4.97050942147364	-5.90553830578888
C	-9.41301616182993	-3.90116097848720	-6.77320290844867
C	-10.68137542511862	-3.83660921045728	-7.45096075978673
C	-10.96073184100081	-2.86876491167156	-8.46131733372522
H	-10.18392259309458	-2.13733457035941	-8.72455646319612
C	-12.18218841250394	-2.85616941835344	-9.11963548943631
H	-12.37095768108775	-2.10845439451845	-9.90455562228712
C	-13.18592333106277	-3.80697720492949	-8.79655197611421

H	-14.15044460883134	-3.78694734400714	-9.32549390107602
C	-12.94385985413512	-4.76528362276904	-7.82450574452900
H	-13.71011430055379	-5.51554039875613	-7.57479793911725
C	-11.69581065082248	-4.81508248338752	-7.13631985488672
C	-11.42813963403562	-5.81846628464095	-6.15887690887357
H	-12.21962434656154	-6.53563616972682	-5.89297504059292
C	-10.18852260712141	-5.91604715598068	-5.54177879052541
C	-7.05758607646239	-3.24972719983043	-7.30981795851181
C	-8.35247808549738	-2.86999135232285	-6.93279002431929
C	-8.60519839116976	-1.48953335210268	-6.60399361075278
C	-9.84936168072099	-1.03363576025070	-6.07185879209322
H	-10.65715625522135	-1.75817585697657	-5.90216385171137
C	-10.04747244630006	0.30381753697425	-5.76014082419149
H	-11.01567027238482	0.62860784032903	-5.34979723888029
C	-9.01304458751453	1.25542037758414	-5.96230358966160
H	-9.18594601832569	2.31491549849930	-5.72053010154989
C	-7.78359415794508	0.84180657587537	-6.45004691293516
H	-6.96508618202984	1.56417823985320	-6.59408117877704
C	-7.54242871564692	-0.52823494139307	-6.76612019396143
C	-6.26766741187910	-0.96870704139013	-7.21675148513350
H	-5.46321479804448	-0.22891163455721	-7.34901297260553
C	-5.99356833498595	-2.30735181309447	-7.47445092848035
C	-2.97085884935005	-6.41391109275957	-5.72159544091068
C	-1.79175889311282	-6.91479942754659	-5.14516914010918
C	-0.66741869344678	-7.19352850338781	-5.92823047449567
C	-0.71242779943109	-7.08493070776600	-7.32966946769439
C	0.48318549621716	-7.54614363515625	-8.14054645942333
C	-1.90172767724955	-6.61992606424468	-7.90657405928728
C	-3.00389609560218	-6.25524590232277	-7.11423866773811
F	-2.03872429780942	-6.48893425554807	-9.22712766807616
F	0.65169162739421	-8.88263243318111	-8.02716267221512
F	0.37356638174763	-7.25644239998963	-9.44865265269252
F	-2.15381821062376	-4.67821482065323	-12.92106053199717
F	-3.00422222611206	-5.83817340496171	-14.55790622262214
F	-5.42564244295870	-5.03699541268765	-11.36637001123392
C	-9.87223675502736	-6.91726961972643	-4.48417162170285
C	-9.88931619757974	-6.52729874831496	-3.11452947551384
C	-9.48673920431269	-8.21995849171700	-4.83539000411720
C	-9.44329723036240	-7.46047969370113	-2.11768383235039
C	-10.33204092988288	-5.22676693114170	-2.68717002595973
C	-9.06363789026666	-9.13582368640809	-3.86671221743440
H	-9.48649956293272	-8.50380089928213	-5.89429449880507
C	-9.42490956839842	-7.08107832127316	-0.73795278444862
C	-9.00618426266615	-8.77343274866219	-2.50504763782683

C	-10.33755407443480	-4.87212533435412	-1.35909617292143
H	-10.67818488239125	-4.51456775373635	-3.45074509163606
H	-8.75161148885846	-10.14477223149179	-4.17322044574716
C	-9.88639460715749	-5.77973195055900	-0.34042354366168
C	-8.94105805586602	-8.00068972244187	0.25568367136524
C	-8.52467950532729	-9.67118277051448	-1.48859669086140
H	-10.69023280823277	-3.87487699348760	-1.05271905957914
C	-9.87909517251313	-5.43659200178312	1.03061227609446
C	-8.93859673697034	-7.60439192297784	1.61244880478753
C	-8.48386767816364	-9.29848339452135	-0.16741916065751
H	-8.18624942512779	-10.67318274012007	-1.79495867001128
C	-9.41193765363353	-6.34081012076489	1.99320484818525
H	-10.24236607274074	-4.44191805206670	1.33215748864564
H	-8.56967712559381	-8.31084137237173	2.37234127820878
H	-8.10856393544843	-9.99649281804345	0.59695708857632
H	-9.41506570108507	-6.05709632979478	3.05689770767191
C	-9.17128562756128	-7.82195016343531	-9.78366619234811
C	-8.33617858888076	-8.09893113591711	-10.90900040985828
C	-9.72100315558750	-6.53534474790348	-9.62874903067888
C	-8.07170991208293	-7.04860100773144	-11.85499336949650
C	-7.74209956909819	-9.38912337370864	-11.14599699369679
C	-9.46910649424049	-5.51514452750673	-10.54884470316683
H	-10.34003957464841	-6.32999999324712	-8.74484613610871
C	-7.21854558325780	-7.29185529130275	-12.98011727565040
C	-8.64988803296219	-5.74541745000661	-11.67409648827390
C	-6.89613044252021	-9.61143111224445	-12.20425203194861
H	-7.95167444691168	-10.21396018749603	-10.45329742793687
H	-9.90657030392887	-4.51825268112134	-10.38905809994803
C	-6.59929371724754	-8.57631112432816	-13.15384602773756
C	-6.95732363005733	-6.24461863752435	-13.92929439061204
C	-8.37349401269838	-4.71799229986899	-12.64207772449707
H	-6.42636713070685	-10.59842014397576	-12.33202947170167
C	-5.72788192120878	-8.78196748109865	-14.24784765946676
C	-6.09150833612627	-6.50348822566651	-15.01509994770587
C	-7.56623889795921	-4.95705096887346	-13.72631424103662
H	-8.82445469657859	-3.72520560126906	-12.48829457541709
C	-5.47946990762611	-7.75513714698657	-15.16680190720841
H	-5.24534539022096	-9.76441179261138	-14.36614690137388
H	-5.88149456326050	-5.69579830664749	-15.73243251363247
H	-7.35448528318833	-4.15907539240501	-14.45448094810174
H	-4.79107572745918	-7.92723508794806	-16.00740974958187
C	-4.49092855266389	-10.89919175352941	-4.50461547864739
C	-3.08913683880703	-10.81912515259716	-4.74076681370408
C	-5.01132559889185	-10.46669500601205	-3.26908809959327

C	-2.22878938040701	-10.33609600780397	-3.69295898163461
C	-2.49755946790850	-11.18031010015458	-6.00107388753587
C	-4.17991767744633	-10.00368753886883	-2.24787045192089
H	-6.09732614706392	-10.51598380651046	-3.10379093540594
C	-0.81410297536072	-10.26873385792334	-3.89997206030381
C	-2.78312690995197	-9.92607779454714	-2.43349225218709
C	-1.14112431502695	-11.10294589727457	-6.20182957567130
H	-3.15677550394026	-11.47667284517794	-6.82813662776712
H	-4.61478080432671	-9.68310475512164	-1.29069843677754
C	-0.25141537653788	-10.67102861818664	-5.15922417239140
C	0.04907021722399	-9.78793926654921	-2.85479719621296
C	-1.89758440114419	-9.44776781363248	-1.40617522509420
H	-0.72243245635794	-11.33792669698612	-7.19117265156546
C	1.14932222502470	-10.60212398113530	-5.33713238777678
C	1.44199294913106	-9.72513450396717	-3.08504365681379
C	-0.53955537038475	-9.38071535798003	-1.60654959585352
H	-2.33736386045919	-9.13030366561821	-0.44733131864496
C	1.98135144976592	-10.13512445628462	-4.31199017798611
H	1.57615420060535	-10.90033614010425	-6.30631913182752
H	2.09689105495061	-9.33695809949349	-2.29017161976988
H	0.12657044531195	-9.00485968646933	-0.81439529412994
H	3.06815252227713	-10.07698650054392	-4.47584192013372
C	-4.63546733158232	-2.71338701488623	-7.94127407214463
C	-3.54032945181087	-2.79835404143625	-7.03655279999414
C	-4.43393022647132	-2.97849075019223	-9.30811128747948
C	-2.24951779395924	-3.19005723304294	-7.53629691678767
C	-3.67837277228925	-2.55740661469430	-5.62435650266758
C	-3.16974281906666	-3.30675237700191	-9.80561716988085
H	-5.29111400836418	-2.92690693576689	-9.99365637055307
C	-1.16114818500716	-3.40361791758462	-6.62955953519220
C	-2.06037789444444	-3.42281928320609	-8.94054013647555
C	-2.63229485250643	-2.75750665563835	-4.75897717933968
H	-4.66197826478707	-2.24670035371093	-5.24526347379584
H	-3.03028780205951	-3.49279927571924	-10.87965458165102
C	-1.35383184781266	-3.22401132019281	-5.21812663879136
C	0.11348957262652	-3.84781359340674	-7.12248625490609
C	-0.75991468689127	-3.81775307911131	-9.41316972195128
H	-2.76447195264657	-2.60007737765840	-3.67845587735540
C	-0.29884498061553	-3.53881626376649	-4.33253955377567
C	1.14278926237632	-4.13862854910234	-6.19869007694859
C	0.28168734330565	-4.02329658623644	-8.54192062580013
H	-0.63269310949823	-3.99780533644226	-10.49138981654137
C	0.93110856066470	-4.00087868426888	-4.82006664702521
H	-0.46075752185960	-3.43052578246363	-3.24878585577110

H	2.10786105568248	-4.50741806050932	-6.57580043653650
H	1.26000017370307	-4.36152680277935	-8.91480427852341
H	1.73149384521978	-4.26769902232819	-4.11585445072909

IRC end point of **TS-3a-4**

C	-0.07413133830129	2.01150590956313	-6.29682421251974
H	0.17610442384935	2.89879717283121	-5.69075611441314
C	-0.98048070955673	1.00766769016342	-5.83163039301908
H	-1.41772058700387	0.38049761064396	-6.63036417248381
O	0.64655880667065	1.94720649200054	-7.37893446416742
C	0.37789002122605	1.00354296709865	-8.48023204438550
H	0.92083703715917	0.06680076369050	-8.25621078896537
H	-0.70866037613358	0.79538340623969	-8.52276898738622
C	0.87686100263335	1.65705306353223	-9.73736141163535
C	2.17787764807767	2.19593316542162	-9.77783115948353
C	0.05288506128138	1.75483706650890	-10.87237451821273
C	2.63916868987155	2.83493809930001	-10.93193901065131
H	2.83051319042744	2.09743586287878	-8.90030782055605
C	0.51582488343263	2.38522446450810	-12.03538360694086
H	-0.96331310187400	1.33257499952618	-10.85370132133660
C	1.81088482744012	2.92675482388314	-12.06461027270834
H	3.65085293826708	3.26312800420718	-10.96427734320309
H	-0.13574490212732	2.46845088442319	-12.91539941792305
C	2.03820097214053	1.69778646769801	-3.75980706680494
C	0.90569974415172	1.25310503166880	-3.01454615584205
C	-0.04355162895379	0.35263544441981	-3.43172218060904
C	2.64046594342440	1.02879683686189	-4.82709661313633
C	-0.21013762782132	-0.16357523423052	-4.80179138833955
C	2.24872358376369	-0.15247215969224	-5.49544202506819
C	0.98235018147961	-0.73964579948871	-5.48609783366215
H	2.58379335172056	2.56667492156635	-3.35749244050913
H	0.78242696210261	1.68807734707753	-2.00724450267163
H	-0.85257289339862	0.08993489900110	-2.72917494426073
H	3.60857340339922	1.43746703985831	-5.16279261849606
H	-0.99804814757418	-0.93729706279097	-4.81923459617721
H	2.99711852898026	-0.60165376067369	-6.16284806096399
O	0.68300428059504	-1.80234627325036	-6.21491573473869
C	2.34843966850266	3.54808090289735	-13.33070557826946
F	2.96289490663897	2.61794125845691	-14.11456389445121
F	3.26841281438941	4.50768663016460	-13.07379656858179
F	1.37044441183096	4.11011939580849	-14.08028687628844
H	-1.77029768671767	1.42504878344585	-5.18310110727501
H	1.48901738658509	-2.15850531670616	-6.74457581382686
S	3.40198813673431	-7.02435208785918	-9.09206148925301

S	3.12765121218649	-1.71635678370082	-8.79346265743380
P	1.08721068973269	-5.31679613368498	-8.99288299635795
P	0.89146390083929	-2.64013829655754	-10.39715417223676
F	5.12878644261899	-9.14408325123903	-10.17345112464962
F	6.09409256304976	-9.45196012257598	-12.63075687031142
F	4.61184244638598	-7.32254587709065	-15.86512733834546
F	2.06977080293453	-5.85663873121149	-11.81777958792538
F	5.90974455956860	-1.32710650997235	-7.68214706143148
F	8.08465556666608	-2.79594613496561	-8.12316507671331
F	9.29503157900909	-4.43707372552259	-9.98987018992800
F	3.60780586227041	-3.83221824227710	-11.04132519320890
O	0.29545699461107	-6.64335031885214	-9.58532053779599
O	0.45816995330013	-5.15915816778909	-7.45626472554247
O	4.80960498239812	-6.79397370927912	-8.68581842469920
O	2.63307350441367	-8.11314786964579	-8.44857582009171
O	-0.42359786713829	-1.61557899139923	-10.25110855790196
O	0.92665570543746	-2.90068573308811	-12.02616215756303
O	3.49770213897369	-0.33040652156516	-8.41762665858774
O	2.64914559675393	-2.59399838425017	-7.65524085733644
N	0.53983375772651	-3.99991612546162	-9.69607028007096
N	2.65372109985198	-5.58705992323955	-9.07635734080891
N	2.11635372984114	-1.66525934241435	-10.01901941263771
C	-1.09039314673986	-6.64172152753781	-9.56411399348106
C	-1.73349811293985	-6.79324416841521	-8.33742180262648
C	-3.15904493693726	-6.57716899126261	-8.27769784002774
C	-3.88751382646466	-6.54412518638120	-7.05233176140631
H	-3.35702625256414	-6.73545691794101	-6.11005119305576
C	-5.24390287086178	-6.25142213706745	-7.03337865161504
H	-5.77953784019521	-6.21717307835747	-6.07278240280084
C	-5.94418783315104	-5.98961283740253	-8.24008547222825
H	-7.02084920694878	-5.76382257049393	-8.21223898515917
C	-5.26622047320711	-6.01547345533840	-9.44897587283104
H	-5.79364141834794	-5.81304378830219	-10.39449759630713
C	-3.86858238625687	-6.29571743826102	-9.50126061874881
C	-3.16581639780069	-6.30509924451528	-10.73736499021220
H	-3.73154903958905	-6.17169437900069	-11.67215100375487
C	-1.78730807498944	-6.48848512406893	-10.80366099865691
C	0.04304207528530	-6.21065900770862	-6.65893335268174
C	-0.95242123137548	-7.08801365137162	-7.10645104023110
C	-1.27455108135787	-8.24060559777419	-6.30605615219088
C	-2.13288835692396	-9.27915105270389	-6.77342065723389
H	-2.56083919049785	-9.20392120647794	-7.78306687973077
C	-2.41047660821168	-10.38393726520391	-5.98105725145067
H	-3.06296685571509	-11.18139299078537	-6.36753593113852

C	-1.85170576322871	-10.49944112308380	-4.68076953937289
H	-2.08018977434900	-11.38020680915945	-4.06197923061732
C	-1.01068379213178	-9.50795408929732	-4.19919781341069
H	-0.56314712377412	-9.58993442969547	-3.19650509217986
C	-0.69745577134501	-8.36588775817757	-4.99263911100769
C	0.13864650729263	-7.32194970226199	-4.50974453512877
H	0.49760009881487	-7.36114525145763	-3.46992550102778
C	0.52222737365549	-6.24916289673604	-5.30817573917520
C	3.60890065656218	-7.44312706506612	-10.87338015532571
C	4.59697915117111	-8.40813553233784	-11.14895148125247
C	5.10117490334878	-8.57552814371033	-12.44479647954149
C	4.60497759853002	-7.82076825113199	-13.52594775747300
C	5.31073355020186	-7.89797482734286	-14.86362168351059
C	3.55384990303301	-6.92871973161066	-13.25893627572132
C	3.05395428501682	-6.74692970621351	-11.95615304373022
C	-1.60474536938917	-1.94835135399402	-10.89973898458872
C	-1.66740348366994	-1.85526885098254	-12.29599273393233
C	-2.85753834352073	-2.31770782260613	-12.96491555046956
C	-2.94269379990378	-2.43860597867081	-14.38472683843305
H	-2.07874099581513	-2.14519905968712	-14.99665758552556
C	-4.08404358585341	-2.94208794545233	-14.99144027977694
H	-4.11830009357135	-3.04301442673041	-16.08656131936221
C	-5.20342991472187	-3.33848340264802	-14.21242484285054
H	-6.10299876501970	-3.73457949861264	-14.70674672584595
C	-5.15305713395448	-3.23644221053162	-12.83120397615663
H	-6.00881220258882	-3.54962844896417	-12.21282359483830
C	-3.98766263699581	-2.74010684854700	-12.17536735370550
C	-3.90711928834423	-2.67694691158197	-10.75740907996033
H	-4.78119820425323	-2.96966577206919	-10.15599838323277
C	-2.73681776033070	-2.30529739190208	-10.10533884514796
C	0.76062124448088	-1.83459569205803	-12.90082436753923
C	-0.51996634895753	-1.29157117181312	-13.05490569274147
C	-0.69326114591121	-0.15770302829284	-13.92696809367638
C	-1.92302173722462	0.55987255452709	-14.03098484293472
H	-2.78332363018521	0.23879867654706	-13.42831497608131
C	-2.03972712114638	1.66097503511084	-14.86862073209098
H	-2.99554465648302	2.20375302136053	-14.92296417447101
C	-0.93719849541362	2.09542851846472	-15.65119697312601
H	-1.04146835571906	2.96938182131434	-16.31097958188016
C	0.27654734096227	1.43313800567220	-15.55780374631070
H	1.15056260189205	1.78343230179354	-16.12614431359310
C	0.43593822260483	0.31426630761256	-14.68827338910482
C	1.69884126384457	-0.31901487854616	-14.52835856436022
H	2.55706249113323	0.04868377693231	-15.10945508992536

C	1.89234814841185	-1.36940025223725	-13.63913677074049
C	4.64424787005146	-2.56302979508860	-9.30119925824110
C	5.82951572801704	-2.30507165397147	-8.58325668546017
C	6.96799597598579	-3.09186141371030	-8.80011898664034
C	6.95061798924122	-4.19099463937832	-9.67736958638768
C	8.15279022865259	-5.11426949670920	-9.71103102759396
C	5.78381361289811	-4.41136128489565	-10.42256699549528
C	4.65414178514006	-3.59320484718621	-10.25315493897371
F	5.70085052237977	-5.38693506020695	-11.32837249935774
F	8.32313182395170	-5.69262278884754	-8.49522753070002
F	8.03664213590843	-6.09571898292912	-10.61845350500943
F	6.50666306553738	-7.24630727087345	-14.79477728500431
F	5.57754061715317	-9.16760668152479	-15.23154164080486
F	3.00173836641438	-6.19506778003577	-14.22984506548947
C	-2.71231001876390	-2.28642532046159	-8.61395936199839
C	-3.32257750987476	-1.21934942678298	-7.89543016197549
C	-2.16385293732573	-3.37125458278209	-7.91296936234057
C	-3.41758550305191	-1.30490790116043	-6.46226708425370
C	-3.82934375344819	-0.03764725928577	-8.54521030727219
C	-2.24950899163899	-3.45847665984840	-6.52185057701825
H	-1.66511976351484	-4.16120483621258	-8.48224958809212
C	-4.00620108005781	-0.23456112881881	-5.71502225712921
C	-2.89151561847890	-2.45143172016959	-5.77397491483539
C	-4.38003215196273	0.99664074438059	-7.82547411275263
H	-3.76060515884111	0.02497464542212	-9.64144080281064
H	-1.81086426340446	-4.32119614843644	-6.00131418140327
C	-4.48741788376420	0.93895971465662	-6.39201031533258
C	-4.07699584980027	-0.30934134086583	-4.27999952037502
C	-3.00592673617235	-2.51355170907920	-4.34031549908253
H	-4.75008137196536	1.89605086985010	-8.34183202714642
C	-5.02192949410453	2.00255335162555	-5.62937688495515
C	-4.62034865899603	0.78056937308793	-3.56246683939128
C	-3.57350671679897	-1.48800609087132	-3.62289606271748
H	-2.61255728268838	-3.40350186460470	-3.82653906769100
C	-5.08466844604808	1.92172116574977	-4.23168499522401
H	-5.38858393474408	2.90054573106481	-6.15028491331479
H	-4.67468437056292	0.72165949760563	-2.46435369690833
H	-3.64748484766146	-1.54834481010039	-2.52587883066594
H	-5.50395675065798	2.75977868833428	-3.65470538081821
C	-1.10310111353987	-6.54672975114764	-12.11844859709245
C	-0.27902264388595	-7.65148388508816	-12.49189996037091
C	-1.34745656368789	-5.51967958976873	-13.05036542564507
C	0.27273795654224	-7.68976974340419	-13.82025281060435
C	0.02460184238096	-8.74190588134350	-11.60219674078343

C	-0.81846460472190	-5.55807829691389	-14.34115166755192
H	-1.95755052148788	-4.66219130792152	-12.73933882226492
C	1.12661588292249	-8.77066184681695	-14.21394440067022
C	-0.01263911528866	-6.63834101295080	-14.75772420388668
C	0.87268770251900	-9.75778310219379	-11.96836329917332
H	-0.41372860095459	-8.75425606845933	-10.59661155194077
H	-1.02770293898834	-4.73617464424520	-15.04305322055757
C	1.46220492217361	-9.80554778310871	-13.27648865521304
C	1.68011345950137	-8.81032158201668	-15.53998288962011
C	0.53975437903939	-6.71404650051035	-16.08342461045264
H	1.11442349023451	-10.55634396410491	-11.25046592134570
C	2.35262239554507	-10.83168203066343	-13.66678183414052
C	2.55309838712336	-9.86455924903289	-15.88805920504059
C	1.34870161016584	-7.75645187513706	-16.46126222763857
H	0.30570496184775	-5.90342727729716	-16.79113770252745
C	2.89175047604766	-10.85714687184035	-14.95857731877692
H	2.61638839266416	-11.61261894170740	-12.93701702026766
H	2.98755738135206	-9.88300392046678	-16.89906844012933
H	1.77658557340901	-7.79448556892827	-17.47480668198955
H	3.59183753562890	-11.65608420293694	-15.24450151025784
C	1.22128972172543	-5.08883740010496	-4.68331867415683
C	2.57330876959236	-4.74771847230664	-4.95201378780861
C	0.49064808526307	-4.33708055399464	-3.73898076120332
C	3.15483432234489	-3.61852688210382	-4.27462523439023
C	3.39563676598511	-5.52155046894658	-5.83908704273962
C	1.05219525333498	-3.24507974477080	-3.07178441320455
H	-0.55008391857771	-4.62955830140043	-3.53143568031886
C	4.51890045758506	-3.26747923418127	-4.52064874578587
C	2.38766698833162	-2.86281391124612	-3.32330116658849
C	4.70985867866432	-5.19310897606687	-6.06138392905227
H	2.96087588708186	-6.40235919248001	-6.33284472967665
H	0.45684327410411	-2.67462231073768	-2.34209913520972
C	5.31194056275327	-4.06164053817603	-5.41542088513545
C	5.10990110541037	-2.13978299298642	-3.85391618674711
C	3.00816293286884	-1.75105184039517	-2.65512393930857
H	5.30240753960385	-5.79447375176490	-6.76548318457103
C	6.66648690046027	-3.71849598906322	-5.62098181544474
C	6.45893572433310	-1.81385759205661	-4.12233229006384
C	4.30909538033347	-1.39724957287065	-2.91752958060598
H	2.40823641347897	-1.17716995320812	-1.93248302679298
C	7.22593573844196	-2.59701810945391	-4.99508368635744
H	7.27641108570830	-4.34755346494188	-6.28508960249328
H	6.90619057442020	-0.94122650350040	-3.62087254837383
H	4.76550293919915	-0.53581428573178	-2.40490703005291

H	8.27577967929923	-2.33322796286826	-5.19116374921323
C	3.23839278149422	-2.00705791569115	-13.53933684894032
C	4.28986064043385	-1.40213831959777	-12.79619046386466
C	3.48462251183394	-3.18836579504922	-14.26161688244590
C	5.59344797432587	-2.01059177151675	-12.80685016125342
C	4.08748349827373	-0.23091836449069	-11.98541976713435
C	4.75648769871914	-3.76856502357435	-14.29336363587580
H	2.65819856035736	-3.65832019887357	-14.81363839877874
C	6.65329432050152	-1.45406406219352	-12.02119408718038
C	5.82932107360642	-3.20033503476359	-13.57577037256792
C	5.09839307185740	0.28230531329849	-11.21005205165531
H	3.09071490873479	0.22811063048621	-11.96385978503957
H	4.92600045987780	-4.68073234503326	-14.88224976963650
C	6.40792092869403	-0.30714956028998	-11.19291984727269
C	7.95076477731690	-2.07151778404894	-12.01618424397241
C	7.13990033561274	-3.79493325380479	-13.56011011955099
H	4.90831372496767	1.14657510803794	-10.55873135420451
C	7.44800931199115	0.18735197055523	-10.37385670347063
C	8.96460086037559	-1.53008127497129	-11.19411629251561
C	8.15866729908449	-3.25027113945270	-12.81737044327185
H	7.29631858284261	-4.72032739139270	-14.13470215873747
C	8.71047395819811	-0.41968283828963	-10.37775512247073
H	7.24670685725334	1.05184022300225	-9.72296355917746
H	9.95298800845833	-2.01247943888261	-11.18097712197382
H	9.14967617948704	-3.72808199574819	-12.79593983730314
H	9.50754814942583	-0.02772286075938	-9.72832427247320

IRC end point of **TS-3a-4-SRR**

C	-5.82055854257689	-0.03639687658250	3.55552994032897
H	-6.69478553095616	-0.23965109249068	4.20104457850062
C	-5.80537244919588	-0.19316055565949	2.13154230077985
H	-4.78684926387469	-0.43363105265850	1.76757000259619
O	-4.76704143472448	0.52322492511523	4.07875583183048
C	-4.78609254352874	0.93402200468771	5.49176198694783
H	-5.67785295642808	1.57456032484770	5.64125305483949
H	-3.86827508458448	1.53469057213034	5.59226796150795
C	-4.77855021884570	-0.25286106139005	6.40773204833483
C	-3.58162329103049	-0.96869636205033	6.61402613683347
C	-5.95643631201844	-0.66941693863208	7.05426613957190
C	-3.56316613146999	-2.08509505471250	7.45544647305715
H	-2.65594179625047	-0.62677477859443	6.12694175444259
C	-5.94086910100575	-1.77994406338769	7.90768953459752
H	-6.88930139031258	-0.10098947146702	6.91852531432065
C	-4.74336810741265	-2.48343425173719	8.11203717015074

H	-2.63049570381587	-2.64051644835784	7.62180016080942
H	-6.85872960536317	-2.09133986749685	8.42520013490263
C	-7.88590568008499	2.51899789875319	3.52358203219986
C	-8.32926409242264	2.03209091413196	2.25468986584665
C	-7.53479395234770	1.60574323485551	1.22414930621448
C	-6.63306322749854	3.06366524454959	3.80821042975143
C	-6.09065500751371	1.31930878351686	1.30973105210628
C	-5.45039352915301	3.09583367733834	3.03477651910598
C	-5.16234505789098	2.31220850249547	1.91698145063429
H	-8.65376996694599	2.65087782209461	4.30382445086168
H	-9.42197850638244	2.00842642758676	2.09756065320114
H	-8.02799727646639	1.30192553215943	0.28606290766693
H	-6.55408762394669	3.58267828869974	4.77670683407142
H	-5.68464530377949	1.06256421848718	0.31344017150862
H	-4.63394721581922	3.72494376835432	3.42370962054297
O	-3.94905517983303	2.26588135092507	1.38624517142611
C	-4.71221916530700	-3.61797696769222	9.10717074344614
F	-5.90265415845754	-4.25918611902151	9.18154660156234
F	-3.76770358244980	-4.53841242109504	8.80716750997247
F	-4.43722078282345	-3.16112340744860	10.36314004367212
H	-6.56873449100944	-0.89314209127517	1.75231249892524
H	-3.25466391539925	2.56783774496501	2.06620907792330
S	-0.39743745548688	8.05600742262743	6.51714760996984
S	-1.97554464919888	3.38249911036094	4.42166803142284
P	-2.29379604227918	6.03996559630073	7.39187238307966
P	-1.80326857942414	3.04658485980424	7.26284707859746
F	0.49789249213183	5.63138555509512	8.45049851033814
F	2.93912407980790	5.05473323560165	9.17004077361930
F	6.00855428868176	7.75903412977725	8.92201129126540
F	2.02346826866072	9.75257734793895	6.56438625141875
F	-1.28616188465223	4.17567599850974	1.59210278286048
F	0.52758910839752	5.94561466008261	0.78049850050071
F	3.10379418117156	6.85876804949957	1.43744277973230
F	0.51640844854727	4.42317928209478	6.01266486737089
O	-2.36827337347840	6.88320503539447	8.81058281526792
O	-3.83144774062997	6.24847087201495	6.81215539488666
O	0.06324233027451	8.36509851922875	5.14137063874694
O	-1.11556853187084	9.09959444814479	7.28245147148058
O	-2.93635826237265	1.93621957000918	7.78901402800290
O	-0.48834917572435	2.61714657396793	8.15302540630179
O	-2.27778959481335	2.37242168294172	3.35304316605711
O	-3.00778983874940	4.43595042835937	4.62925003523518
N	-2.22438928591775	4.48522110712762	7.72447438052771
N	-1.15804853759046	6.63080228993735	6.45340946883604

N	-1.58536061368551	2.56506861243411	5.73499873460673
C	-3.32289405923685	6.49565441812105	9.73997999553051
C	-4.66991454749212	6.76948063162760	9.47828425396487
C	-5.66633187857930	6.15735636962936	10.31875917150828
C	-7.05583779596028	6.17100885123980	9.99795971037785
H	-7.38599769125573	6.68881050309028	9.08722057042305
C	-7.97933081449857	5.50764458923500	10.79244953837658
H	-9.04147707384231	5.50305799510473	10.50528363230490
C	-7.56307774848642	4.82835462652552	11.96860071038829
H	-8.30719206272470	4.31882765223034	12.59909889567174
C	-6.22006741097722	4.80070761122202	12.31091559585806
H	-5.88125693939349	4.27352363715466	13.21669682735784
C	-5.23907298729916	5.43032563231839	11.48699670887263
C	-3.85230701336596	5.33271788436031	11.78142458597067
H	-3.54121337005459	4.82130696734535	12.70496195061235
C	-2.87170947149355	5.82800597621345	10.92245676779351
C	-4.58020098241068	7.38804033541112	7.05110134220737
C	-5.03777103523364	7.65787799537377	8.34350079960114
C	-5.84225870076412	8.83671801799628	8.55252736566890
C	-6.24481663170957	9.27699827704527	9.84879272436311
H	-5.91772500739835	8.71092009795271	10.73114556409338
C	-7.02750682074913	10.41126903423780	10.00857205109228
H	-7.31775362135870	10.73306238446238	11.02015803237080
C	-7.44757934794994	11.16533623083857	8.88155409850540
H	-8.07116590232116	12.06118340807264	9.02108936089413
C	-7.05249796394357	10.78080572524453	7.61012671283996
H	-7.35037126897079	11.36911106559464	6.72830676106131
C	-6.23811181994142	9.62703347448217	7.41165409308182
C	-5.79031630070739	9.25647776902878	6.11222097518464
H	-6.09501879252070	9.86464801026493	5.24674628539484
C	-4.94958973757069	8.16977966138092	5.91398878848419
C	1.16761133364017	7.69042614976984	7.40485509936150
C	1.43917217939053	6.52112244788253	8.13027266013010
C	2.75055297518170	6.21425742919020	8.53083688937968
C	3.82330125627600	7.08147363955560	8.26455029641814
C	5.26127894990123	6.70188837398832	8.54757668088520
C	3.53010861055159	8.28696059953804	7.59718440761734
C	2.22368881908897	8.59599130553412	7.19546180666752
C	-3.12561861758789	1.71533244366694	9.14319709653024
C	-2.11233085343154	1.07241568578245	9.86492000007341
C	-2.24128345804178	0.97603248873349	11.29572878497769
C	-1.17907594532196	0.52765717693805	12.13540071759707
H	-0.23234072419728	0.21317048791968	11.67476458195833
C	-1.32159684394632	0.50644458557172	13.51571571875683

H	-0.48279943723987	0.17207812259116	14.14446875742117
C	-2.53561480812012	0.92194521113073	14.12457142549208
H	-2.63555212526716	0.89857639141207	15.21995021252780
C	-3.58586565964041	1.36600520960585	13.33690061783014
H	-4.52980506253290	1.69935593716129	13.79584588330178
C	-3.46626124792829	1.41546520737450	11.91604982310579
C	-4.52174067822647	1.90810638805374	11.10307084949472
H	-5.46113196596873	2.21825685123793	11.58303932977411
C	-4.38712409310707	2.07409788091753	9.72607027343005
C	-0.17041675258579	1.27206841753005	8.29056586729444
C	-0.94957142857130	0.48341728156807	9.14353638233572
C	-0.64025348205872	-0.92133240934979	9.25565181465439
C	-1.43072099827151	-1.83320018355048	10.01815017252948
H	-2.32956873807045	-1.46897592339160	10.53123871640814
C	-1.10049086588856	-3.17868856511989	10.09592152592391
H	-1.73892315996465	-3.85770090056624	10.67882941186583
C	0.03230152785228	-3.68589534114424	9.40699335888360
H	0.28447100392337	-4.75463706179330	9.47669069010535
C	0.80615760947455	-2.83420604369533	8.63434431630630
H	1.67749638762443	-3.21584945785729	8.07981503584291
C	0.49248569784859	-1.44605309253315	8.53170183711672
C	1.27100637842696	-0.57510244177492	7.71937060880518
H	2.14316285271232	-0.98044946469381	7.18383935576679
C	0.96170333476634	0.77207265560184	7.57641691675449
C	-0.50834429483513	4.28511847378631	3.85649749480473
C	-0.44196027244060	4.66904207320593	2.50386958125241
C	0.51335808920496	5.59692257632159	2.07548409740515
C	1.42499991126942	6.18162609478619	2.97174842615921
C	2.32826038237755	7.29007036874646	2.46802675648177
C	1.40177446335669	5.74250458275021	4.30147727945316
C	0.45142428503620	4.80376779362557	4.73709718726882
F	2.27719802156657	6.18751538411108	5.20270228304188
F	1.58192104080850	8.31652188551358	2.00088162701361
F	3.15089958564857	7.77054105350187	3.41248897003773
F	5.37971318647496	5.76323044219829	9.50877352589639
F	5.83744577992104	6.18370702386903	7.42207173557637
F	4.49923764613248	9.16428445047871	7.31610401455570
C	-5.52393385665952	2.57724469183866	8.90900281855771
C	-6.82629097749387	1.99165347281152	9.00547675952235
C	-5.33020771113986	3.66809815477337	8.03940364146151
C	-7.90974729488650	2.56035341791157	8.24247113012822
C	-7.10737385910243	0.81981840984044	9.79558081898714
C	-6.37438922303766	4.21700707693192	7.29687946414248
H	-4.33653937084861	4.11923893488057	7.96816706696619

C	-9.22634180374156	1.99918221475629	8.32310700041798
C	-7.68016105893000	3.69204056563131	7.38741277021618
C	-8.36888038159324	0.27946717053387	9.86847300373122
H	-6.28025962454872	0.33487799398444	10.33079116560174
H	-6.17291535454175	5.08301145515077	6.64904351874897
C	-9.47485652803519	0.85184006260375	9.15155415062456
C	-10.31006657822134	2.57923128639548	7.57445159861696
C	-8.78063328038543	4.25594817658113	6.65319864489408
H	-8.54812380785993	-0.62047589293142	10.47771679889728
C	-10.78145577337227	0.31712841735687	9.21984778648261
C	-11.59986134890032	2.01026894475684	7.67769410198335
C	-10.04477704024188	3.72448732833272	6.74428492347846
H	-8.58297541250840	5.12919163073229	6.01194062214022
C	-11.83040042545245	0.89300215094033	8.49137852866615
H	-10.96458817301803	-0.56182328779221	9.85717264164985
H	-12.42713106376040	2.46093808278813	7.10769632138091
H	-10.88070524751013	4.16933050773035	6.18201728716968
H	-12.84224635069412	0.46555589751224	8.55872987835820
C	-1.43442309229748	5.60372731533572	11.22516721696324
C	-0.44418792179530	6.63331578279555	11.18340037126186
C	-1.05220425092049	4.30050092289151	11.60799212416521
C	0.91727416400041	6.30556007879321	11.52192720931212
C	-0.73535464925625	7.99490957318888	10.81718841769769
C	0.26011294800842	3.98657850230041	11.95904687156227
H	-1.81200350368477	3.50813212191698	11.60737642829897
C	1.94235751860571	7.30373262424937	11.43876486400549
C	1.26737469872055	4.97353422998749	11.93203223758701
C	0.25483530671196	8.93778461936412	10.68881382597507
H	-1.77142383899459	8.28594267727210	10.60778117262349
H	0.51555124842972	2.95613622251250	12.24994844664058
C	1.62630283513976	8.62715827913482	10.97831031387140
C	3.30020351276224	6.98016349345838	11.78305120894865
C	2.62834672199349	4.68424329221673	12.29373834632526
H	0.00150033245389	9.95379844759093	10.35081730156265
C	2.66432584575981	9.57609666485976	10.83424235367847
C	4.29978588738598	7.96877230560429	11.64150495175155
C	3.60406342057420	5.64712527392740	12.22943787684108
H	2.87374719521884	3.66021669607707	12.61585803534279
C	3.98611300053335	9.24714127768254	11.16078697175062
H	2.41610295324061	10.58182476042242	10.46142422576292
H	5.34035829879851	7.71144673175664	11.89022821201448
H	4.64543105411866	5.40889900868478	12.49447931413582
H	4.78432828658520	9.99330972721485	11.03263119414125
C	-4.42954250087960	7.79203879297066	4.57024741213924

C	-5.24719545917955	7.09997527110790	3.63568153823173
C	-3.09663666324220	8.09666395458026	4.24325294017243
C	-4.68413973051488	6.70709643059985	2.37321928554328
C	-6.62606989619834	6.77910559088268	3.90092045747449
C	-2.54037698307976	7.71051262691260	3.02158650111131
H	-2.48484368501302	8.65631577558444	4.96177903677408
C	-5.48876761857892	6.01045467550442	1.41604431438771
C	-3.31344645400093	7.01155637284291	2.06851445117687
C	-7.40576717813780	6.13517119802024	2.96952440904498
H	-7.05291025527051	7.08291140686877	4.86864795626482
H	-1.48604134889468	7.95109707830030	2.81548896433165
C	-6.86954109763580	5.72730020191529	1.69946733550353
C	-4.91836693991473	5.59012136723615	0.16506091855951
C	-2.78053833553860	6.60520638029077	0.79630382097797
H	-8.46189195293037	5.91023928160056	3.18710397092723
C	-7.64331485253323	5.04265606339197	0.73741227632765
C	-5.72835982694267	4.88695949503028	-0.75599693881875
C	-3.54421307903705	5.91179198552513	-0.10999954834698
H	-1.73269295239363	6.84749305091381	0.56780439714872
C	-7.07439999727077	4.62200149252192	-0.47241799250585
H	-8.70126980191280	4.83257795345055	0.95629254883995
H	-5.28659559272118	4.56085576727006	-1.71041916754593
H	-3.11309669298398	5.59225170005508	-1.07127597734734
H	-7.69154571587844	4.08265544094442	-1.20745244581061
C	1.82306236668862	1.66690719011421	6.74803005019693
C	1.74000073975312	1.66868289989845	5.32774077655680
C	2.75298500462909	2.50268264346273	7.39383887282437
C	2.61247730701440	2.53145890287269	4.57659329397185
C	0.76800671291688	0.88836765034819	4.60838196594859
C	3.62706318410826	3.31522871671861	6.66592367077046
H	2.78583824631097	2.51616124037169	8.49255544225709
C	2.48303800727673	2.62089785471874	3.15295870187588
C	3.57930880455174	3.34750355275234	5.25590918722980
C	0.63342263864062	0.98744508215964	3.24540732328900
H	0.09586997174632	0.23453038595812	5.18033321668822
H	4.35762751188030	3.94727133192900	7.19009959079082
C	1.46935476746169	1.86428311862910	2.47206477752397
C	3.32747090777377	3.51273683972637	2.40642795632787
C	4.43227063813515	4.20974490415274	4.48146313169806
H	-0.15168000057345	0.41814814519583	2.72673132347243
C	1.30737529999946	2.02782269639239	1.07778037398579
C	3.13447608014850	3.63463146168804	1.01176308518851
C	4.31274926183193	4.28772914684547	3.11541310334301
H	5.15926429859765	4.83793016477981	5.01832501908756

C	2.12981538424048	2.90652016535647	0.36012120493291
H	0.51341304282212	1.46366549942971	0.56505048487837
H	3.76829944837201	4.33359445770134	0.44643991421410
H	4.95139132882236	4.97412810221037	2.53933010795242
H	1.98262136852891	3.03128561863818	-0.72322574425209

Optimized product complex of **TS-3a-1**

C	2.50188440580923	5.50255470183337	-4.72749931073907
H	3.55978501455534	5.17881891113667	-4.75553225374733
H	2.38802131232741	6.22916438222380	-3.90045213827031
C	1.65788151858855	4.24222254630933	-4.47888079868711
H	1.06862571504654	4.31983597829985	-3.53554452906756
O	2.58158515636740	3.17516368920004	-4.38916269770680
C	2.05722856370750	1.88172616864543	-4.11422080715616
H	1.34904986064750	1.54870898914168	-4.89697855521402
H	2.93739725560275	1.21436586796462	-4.20151036542233
C	1.43366332795256	1.71583722916853	-2.74830966083264
C	2.12041425891549	2.14002196283801	-1.59211603783209
C	0.19265928495769	1.06827640294674	-2.61222144810241
C	1.58823293150641	1.89989442056693	-0.32178518567738
H	3.09014580100141	2.64957307001419	-1.70231886471802
C	-0.34499465197469	0.82690498869160	-1.34053256308149
H	-0.34812069996803	0.74372507806734	-3.51451161698146
C	0.35419869735568	1.23337503632380	-0.19438099259857
H	2.13633401716514	2.21071308338857	0.57990793087971
H	-1.30626633305723	0.30548688824975	-1.23356933914278
C	2.18570866961836	6.20498925018098	-6.09191998232830
C	2.14249079018916	5.14036289977578	-7.17611315043438
C	1.37644916427955	4.06067240496061	-6.97482701667868
C	0.88244165861603	6.93886215930607	-5.97842417792852
C	0.62681773259908	3.97079521372397	-5.65949049751193
C	-0.33279113832895	6.35203108034466	-5.76794317688231
C	-0.52004079005762	4.93194391267984	-5.63668471859417
H	2.99284371918513	6.93087080790949	-6.30212192362346
H	2.75078772136428	5.27151135675379	-8.07960582390420
H	1.26470578132750	3.23879795667847	-7.69777269251883
H	0.90649164860243	8.03901661842469	-6.03707928336917
H	0.20755194601897	2.96329876295743	-5.51741910707045
H	-1.24560739928919	6.96081240314344	-5.70869989486361
O	-1.72931550674275	4.53462698444525	-5.46860307278650
C	-0.21343118726454	0.99268347223545	1.18125888949872
F	0.73169748741339	0.52327981002105	2.03923420912695
F	-1.22965033240632	0.09514530321573	1.17082433039376
F	-0.69872639793702	2.13588093138753	1.72997151018895

H	-1.84927282438691	3.46015950687811	-5.38729969867627
S	-2.73993353517046	-1.60740446023471	-10.95168880749790
S	-1.73958024899068	1.17789925205855	-6.37502783727675
P	-0.30977440353955	-0.95099144286458	-9.53564059021822
P	0.09364863075346	-1.02901641795621	-6.52790188321865
F	-1.45394574290012	-3.51687550615560	-8.69879011958805
F	-2.52595511843223	-5.65051872128067	-7.67042079427114
F	-5.32088133504943	-7.81171961659475	-9.54753384373906
F	-4.91070802729624	-3.34465075850548	-12.00025716723810
F	-2.79142669839125	-1.67196968899689	-7.16398734368536
F	-5.01943690720194	-2.23129519646662	-8.40603608860651
F	-8.17703139564211	0.15217725779536	-8.39851247847491
F	-4.18722717267222	2.87273548853595	-6.67180533823734
O	0.36687664145949	-2.06773175579508	-10.54342879042711
O	0.39774167939540	0.45421316462051	-10.06482519171037
O	-4.04772454108075	-0.90448683189583	-10.97146973716630
O	-1.99333128560647	-1.76473879241282	-12.21619988981913
O	1.51735543746214	-0.52425916469310	-5.84709255983481
O	-0.09666055106455	-2.53160042867613	-5.87910781020927
O	-1.99691573434700	2.10013263697057	-5.18620771713200
O	-1.11993091866419	1.84889497116323	-7.54526343201089
N	0.28105130578352	-1.14937797977860	-8.07538890437717
N	-1.89062655844902	-0.96633222303353	-9.72763351153347
N	-0.98422623681700	-0.07445062425589	-5.78627639982614
C	1.72523653664998	-2.00781979972292	-10.79958737383092
C	2.21148219673685	-0.99319031946697	-11.62682912625047
C	3.63695539854129	-0.85555400869492	-11.77291515397081
C	4.23599608989641	0.22257583889045	-12.48962852167645
H	3.58718803705627	0.95849779901270	-12.98468716904694
C	5.61501125114444	0.36127375709331	-12.55078110247644
H	6.05395542959463	1.20790155352500	-13.09995203712310
C	6.46410895730358	-0.57663026393465	-11.90632062161915
H	7.55642255002752	-0.45708800384473	-11.96324189147335
C	5.91386088217107	-1.63454235699894	-11.20002321959275
H	6.56191435080444	-2.36376767218380	-10.68929258140128
C	4.50031420714270	-1.79936790449897	-11.10408329221909
C	3.93093913058922	-2.86452808576797	-10.35049525595702
H	4.59832638388328	-3.60274703029934	-9.87990428858686
C	2.55534756650285	-2.99774923138204	-10.18806576896992
C	0.36654478813117	0.67214155944299	-11.43942919271785
C	1.24525101501597	-0.04953982909930	-12.25400192689893
C	1.14745077580380	0.10669106123586	-13.68006273090586
C	1.91545618257144	-0.67410540664770	-14.59417074638510
H	2.61106695128291	-1.42737131230537	-14.19930638969367

C	1.78090916972919	-0.50384651168130	-15.96421950772921
H	2.37799779375572	-1.12262745323336	-16.65102161916636
C	0.87211992683585	0.45346437824356	-16.48799289870188
H	0.77518053454498	0.57896995370930	-17.57688368569224
C	0.09738011231298	1.21422674278020	-15.62683256382164
H	-0.62478177871879	1.94597754549499	-16.02082450821382
C	0.20408161777401	1.05847216556895	-14.21348752841025
C	-0.60535490104990	1.81769206836873	-13.32290219790327
H	-1.30255297637602	2.56139998500305	-13.73788625668965
C	-0.54974236114750	1.63918351985881	-11.94596635740617
C	-3.17874167812206	-3.29383537626513	-10.35871123015881
C	-2.56834471447778	-3.95907139318663	-9.28563217854065
C	-3.13702571193081	-5.12155956686412	-8.73418508436374
C	-4.30908218137814	-5.68031559665797	-9.26810361524990
C	-5.05242035474100	-6.84348879090247	-8.64704635215133
C	-4.87246128745762	-5.05225052717095	-10.39665694055535
C	-4.30998299449557	-3.89243180555754	-10.94523249551069
C	2.59541157116266	-1.37108572136347	-5.67598438428851
C	2.48816593857650	-2.42642771828586	-4.76634468214177
C	3.58687551942572	-3.34812803376714	-4.65581192407429
C	3.51062697672614	-4.53904447510450	-3.87382464204025
H	2.58359560810426	-4.75465338091598	-3.32423005247623
C	4.57814582108284	-5.42331636848848	-3.81758625351444
H	4.49341940499629	-6.34244323693652	-3.21859282441698
C	5.77648308452139	-5.15556731999226	-4.53134700955131
H	6.61632836132892	-5.86422912085029	-4.47678386887711
C	5.88148687711113	-4.00834200363624	-5.30202769774962
H	6.80257218914791	-3.79555961867275	-5.86652463485143
C	4.79937087628389	-3.08378718934369	-5.39251924557822
C	4.89086988026060	-1.90753056950798	-6.19073860857838
H	5.83699599328221	-1.68077395071441	-6.70561197464832
C	3.81052491173711	-1.04701233163802	-6.35433287061204
C	0.00359917112666	-2.61735798174150	-4.48692101400702
C	1.27536091092303	-2.53589485567255	-3.91009348237024
C	1.38301696355409	-2.45691585057509	-2.47518909679421
C	2.61179265811483	-2.17748376407363	-1.80581996781302
H	3.52490461380024	-2.03505780781129	-2.39962158358415
C	2.66023634876707	-2.05773392931969	-0.42451172177247
H	3.61573247331332	-1.82097815393842	0.06726577446726
C	1.48833021433915	-2.22326704973612	0.35946522976840
H	1.53739495063593	-2.11210331924824	1.45216488113955
C	0.27740532694789	-2.48566656386449	-0.25995341109752
H	-0.64498786532829	-2.57967647339873	0.33251910410092
C	0.18820723602843	-2.58672712090719	-1.67959711010430

C	-1.06401318172782	-2.76330117124427	-2.32862955935059
H	-1.97012441315649	-2.87722367046980	-1.71374949956798
C	-1.19246369051188	-2.75430841757105	-3.71457702455238
C	-3.37600593282204	0.63732454820711	-6.91197770342435
C	-3.64335539369274	-0.66707688441197	-7.35492163872483
C	-4.84582391796777	-0.96942009422388	-8.01675067429772
C	-5.81427794130976	0.01643280626009	-8.24840473330221
C	-7.06193510698310	-0.21354003903516	-9.08103708056252
C	-5.56715228502190	1.30439260389647	-7.74133058603649
C	-4.36984023082896	1.61580343372605	-7.09019865943745
F	-6.45621151483067	2.28957589438944	-7.93392694265693
F	-7.01491359133268	0.54104220951830	-10.20145718849263
F	-7.21909677360300	-1.49510596922122	-9.44651418636449
F	-4.38316731846664	-7.41214447790823	-7.62244163532062
F	-6.24926189178172	-6.42073297760279	-8.15067912433038
F	-5.98425059865986	-5.54793679296255	-10.95196736219219
C	3.92350633429423	0.20526376297743	-7.15657613689338
C	4.64342785946162	1.32441419381528	-6.64520203628287
C	3.30881347794346	0.29166660779634	-8.41782546900401
C	4.75486871063551	2.51010163324993	-7.45074180840507
C	5.23643435334164	1.33532487561376	-5.33221869717118
C	3.44429513113382	1.43003144206978	-9.21564527768437
H	2.71956553509994	-0.55906161658781	-8.77996911034889
C	5.41994123476203	3.66880929293322	-6.93884030416177
C	4.18435210334227	2.54299332917224	-8.76762075044456
C	5.84246090033979	2.45982690640621	-4.82761969700831
H	5.17932901995098	0.42091694258064	-4.72405890808198
H	2.97343953622361	1.45690841494813	-10.20727907359201
C	5.94614965555823	3.66630152200763	-5.60212403742349
C	5.55024832793234	4.84645201336902	-7.75429307206954
C	4.37867346015971	3.71190382866521	-9.58345539934227
H	6.26295399145598	2.45348966760383	-3.81004192046902
C	6.54271494600216	4.84345459285593	-5.09596938327707
C	6.16129487188741	5.99706144054958	-7.20550455217321
C	5.04273160830559	4.81471718694521	-9.10207417917566
H	3.97611898929167	3.70086596129643	-10.60825802207195
C	6.64125174055434	5.99580699940274	-5.88797814668049
H	6.93619420553009	4.84209539516738	-4.06757731096218
H	6.25998201751958	6.89852274243599	-7.83040550120680
H	5.18274311177547	5.70484053942432	-9.73560419474861
H	7.11032074969157	6.90259721803160	-5.47673361128319
C	1.96214090117238	-4.09411018819877	-9.38159845104959
C	0.99011776305911	-4.98387670721650	-9.92935406545444
C	2.38016205866131	-4.25865017453631	-8.04637326553017

C	0.45590118475774	-6.02911911756854	-9.09974316352044
C	0.51907884244534	-4.88669630953733	-11.28645903554950
C	1.85631541783059	-5.26812767075903	-7.23587571073891
H	3.11629807435577	-3.55498031450220	-7.63213552175037
C	-0.52636328849523	-6.93153433518374	-9.62225304324861
C	0.89370817255772	-6.16918548388264	-7.73842205339981
C	-0.44432051442306	-5.73277226503375	-11.77738106705007
H	0.93053387102936	-4.10766489357571	-11.94147758050302
H	2.19174281319181	-5.35941467686531	-6.19196228735130
C	-1.00302801386295	-6.78025035406263	-10.96863747096703
C	-1.06011072224869	-7.97850061132049	-8.79485919268789
C	0.33924117247113	-7.22370588991279	-6.93241769273622
H	-0.80374254062834	-5.61593968355484	-12.81119393734059
C	-1.99763942624671	-7.65993487851253	-11.45320351171531
C	-2.04871836315066	-8.83625939927804	-9.32544307215620
C	-0.59249423084926	-8.09583800117206	-7.43949843723607
H	0.68430876758441	-7.31691330507211	-5.89071891897357
C	-2.51224458145589	-8.67596907264145	-10.63850004915299
H	-2.36465365864782	-7.53291109367020	-12.48337142173128
H	-2.46775771605157	-9.62634332340266	-8.68391017781632
H	-1.01125092048578	-8.89622049695642	-6.80998021698513
H	-3.29389893340995	-9.34538135208716	-11.02655903441302
C	-1.39100400454435	2.47870519636219	-11.04074683578515
C	-0.86838157861727	3.69732838392575	-10.52242316639354
C	-2.72643763296986	2.12543025565099	-10.77896588600823
C	-1.72565265287913	4.56559720928966	-9.76417149637700
C	0.48402519328313	4.12255909543719	-10.77223266318629
C	-3.55531503267533	2.95817779033779	-10.01915928099853
H	-3.13316556612238	1.18235286908306	-11.16984296164472
C	-1.24853688763039	5.84259039333671	-9.32513960832923
C	-3.08419931134022	4.18363081348876	-9.50203385851631
C	0.94558026499207	5.34260296969241	-10.34077087054777
H	1.14512214158418	3.45162144913957	-11.34013542026690
H	-4.60287907721613	2.66672213897389	-9.85268130488244
C	0.09583828058081	6.25218751239867	-9.62156238408926
C	-2.13198613243734	6.74296685174527	-8.63113903602171
C	-3.92710285863311	5.07969843678127	-8.75623939882128
H	1.98147821228107	5.64936108993521	-10.55348523050799
C	0.52674696970648	7.53920820509708	-9.22320669423172
C	-1.66624643457541	8.03369088941978	-8.28695077450805
C	-3.47158621242842	6.30672184359354	-8.33905253190339
H	-4.95443384956694	4.75697306826043	-8.53039611026393
C	-0.35422329151406	8.42489820830506	-8.58216257978474
H	1.55604931897850	7.85309337421708	-9.45845136942406

H	-2.35482080707910	8.73199495779783	-7.78566482565678
H	-4.13551343755294	6.98795663560786	-7.78452475743380
H	-0.01189924203425	9.43694590095612	-8.31538655208336
C	-2.54157836263402	-2.89405974723634	-4.33486022391753
C	-3.51227668040538	-1.85839193238179	-4.20507784258707
C	-2.88112116260778	-4.07956721976756	-5.01239407588790
C	-4.81966164351677	-2.04152564974833	-4.77519322270739
C	-3.21706298069659	-0.59666306712530	-3.58127322554157
C	-4.16542932866177	-4.27782537788902	-5.52936723888479
H	-2.12168381594184	-4.86585357154838	-5.13062668466790
C	-5.78066046374156	-0.97996467313336	-4.72964257271052
C	-5.15209912940493	-3.27374425155519	-5.43318663849705
C	-4.13510876761355	0.42395701414802	-3.54236895516630
H	-2.21271422258814	-0.44634972726522	-3.16890328379127
H	-4.41147647809978	-5.22596021148679	-6.02700104362257
C	-5.44064651172572	0.27683015834504	-4.12195791806524
C	-7.07379180175194	-1.15093857431870	-5.33269781523371
C	-6.46112146027102	-3.42616893638419	-6.01160275188325
H	-3.86183624132070	1.39082932307388	-3.09478141901854
C	-6.38290161773335	1.33027117477274	-4.14013846274537
C	-7.98814930951342	-0.07347797880920	-5.31221680762615
C	-7.38145454970287	-2.40745970474971	-5.96565339894719
H	-6.69278332103622	-4.36880591355557	-6.52999911111683
C	-7.64094930683078	1.15347482411671	-4.73073823838534
H	-6.11006851398396	2.29602373864473	-3.68757466662980
H	-8.97359204424495	-0.20369363192753	-5.78297316021388
H	-8.36784069969256	-2.52596997887474	-6.43961345117889
H	-8.36191831346681	1.98468679733933	-4.74143018285235

Optimized product complex of **TS-3a-1-RRR**

C	-4.94087576528091	-3.32646400347224	-1.08335387155487
H	-4.64194766330739	-2.51733740850648	-0.39035933571519
H	-4.49708521482554	-3.12961287965679	-2.07697027071872
C	-4.36710069425323	-4.63260481817559	-0.52347498956889
H	-3.70988983798059	-5.10910239942673	-1.27592372069765
O	-3.66821861346281	-4.35979771845077	0.66767794859717
C	-2.77478439933035	-5.38519525899464	1.11224826193482
H	-3.26679715516161	-6.38368253816097	1.04706417155403
H	-2.60930830784417	-5.17247405228397	2.18648082688728
C	-1.43934493426725	-5.44267768812411	0.39627838558207
C	-1.29133909523364	-6.07397870012043	-0.85721765390750
C	-0.29409525082165	-4.90371904800572	1.01746140840293
C	-0.03047508735991	-6.18974388317005	-1.45679183384742
H	-2.15446718732743	-6.51915190033626	-1.37256988925362

C	0.97410832162876	-5.04103938926705	0.43986971989384
H	-0.39608991413011	-4.39545813905058	1.98913685404360
C	1.10721663657547	-5.70260751638203	-0.79281343862204
H	0.06818520066423	-6.68770199696127	-2.42993855109708
H	1.86571420049028	-4.64968308167869	0.94986413515053
C	-6.49978015760261	-3.29679689128491	-1.20676820724816
C	-7.09754673710996	-3.80410760724640	0.09661192026154
C	-6.62457211942574	-4.96688188272863	0.57380251282987
C	-7.00706621635777	-4.16328099652948	-2.32505373039222
C	-5.55811232683201	-5.68426165263052	-0.20404004403404
C	-6.73606903463932	-5.49484207882216	-2.46724232352067
C	-5.92507561606175	-6.22547252380529	-1.53989948140770
H	-6.81232821494971	-2.25350801709124	-1.39754661566769
H	-7.88779493448580	-3.23114527116989	0.60245167381642
H	-6.97879178107282	-5.40326422598629	1.51837580464380
H	-7.61030972365883	-3.69942838418630	-3.12300387463092
H	-5.11210757217562	-6.52339755923456	0.36256572601500
H	-7.10693194693990	-6.02744554502816	-3.35185615359816
O	-5.37157419946200	-7.34171400384669	-1.86470540374303
C	2.47367552740463	-6.00620558843518	-1.35547590478937
F	3.42940954898307	-5.18669656172010	-0.86286120807197
F	2.84718589956592	-7.28246424852878	-1.05103154273528
F	2.50585098198656	-5.90921927864940	-2.70822752751321
H	-5.26287172735657	-7.45495459468631	-2.93644861234778
S	-3.98952604399892	-9.25716713010505	-8.82033657538828
S	-4.31944715186249	-5.97149461901550	-4.67174682612699
P	-6.21477613581897	-8.41892097813805	-7.17395966176774
P	-6.62724182815881	-5.55728769459573	-6.31617067568739
F	-2.44123555962760	-9.53117359205169	-11.32207423110964
F	-1.98606042795319	-7.75855667349174	-13.23957375513668
F	-4.24501493368533	-4.30975622156733	-13.62757844988379
F	-5.83971686933762	-6.68716156077992	-9.53033618263578
F	-1.71731494250630	-7.11037758313796	-3.85351269748876
F	0.46642528921180	-7.55692911165860	-5.30956997122055
F	1.69016931698469	-6.85040704892706	-7.61577021644052
F	-3.99768671769699	-5.66621140381076	-7.79784434918636
O	-7.09369157271939	-9.12932332227245	-8.37713372788868
O	-6.54147641644596	-9.39644484063766	-5.87535415631310
O	-2.53227669485911	-9.26884200754943	-8.55565136364329
O	-4.66739753734152	-10.52947235196463	-9.15462269875591
O	-7.89059497534968	-5.03577829884368	-5.35370431666059
O	-6.80319024953779	-4.56071296692371	-7.61420430321491
O	-3.75837183910158	-5.22704568990487	-3.51923907636456
O	-4.90400073042143	-7.33665792234529	-4.30361243867566

N	-6.89725156988244	-7.03842163378344	-6.76104329119200
N	-4.68467380770405	-8.41752786652562	-7.61780129931259
N	-5.33902696439903	-5.06745511321247	-5.47405480078442
C	-8.41275355822398	-9.46908094716326	-8.14811937797998
C	-8.69683500801334	-10.52461091886074	-7.28000283494279
C	-10.06590028484774	-10.73130912432534	-6.88645950846914
C	-10.44042402639892	-11.67082951289424	-5.88194395028784
H	-9.65954953854685	-12.28855755322773	-5.41637402519176
C	-11.76246634868020	-11.79536123733721	-5.47889310489175
H	-12.02706711887118	-12.51852945166974	-4.69286959476750
C	-12.77485636565163	-10.99264786935371	-6.06797680403833
H	-13.82016194473462	-11.10310729559206	-5.74236022551347
C	-12.44313695786093	-10.06819826415625	-7.04677397229716
H	-13.21949423104861	-9.43771613337865	-7.50834147964336
C	-11.09237572408568	-9.90383304762227	-7.47497668514296
C	-10.74032632992835	-8.94777793000841	-8.46892995728142
H	-11.54033604154463	-8.37758506184013	-8.96584818837530
C	-9.41670938587165	-8.71891359893207	-8.83659247874656
C	-6.53029084684009	-10.77445442521900	-6.05521600921298
C	-7.57993588748431	-11.36510846824841	-6.76539148823001
C	-7.52263589737723	-12.77638094756891	-7.03918994300938
C	-8.48850317560789	-13.44548240159927	-7.84843319459521
H	-9.31802268701967	-12.86721540500477	-8.27791214820722
C	-8.38271666529258	-14.80421267594473	-8.10792491342912
H	-9.13557867723457	-15.29784622808071	-8.74087996299919
C	-7.30720539779796	-15.56119282406743	-7.57232309852265
H	-7.23461921059414	-16.63822869193005	-7.78576727172198
C	-6.34457307478521	-14.93828637087062	-6.79393847327960
H	-5.49784053210444	-15.51051569410122	-6.38422047711697
C	-6.41880251836677	-13.54193138452086	-6.51365311653122
C	-5.43429876919881	-12.89424274215222	-5.71794037961069
H	-4.62381232373125	-13.49494162783445	-5.27749794469564
C	-5.46851027118602	-11.52631501504159	-5.46699778835171
C	-4.09561449671330	-8.15267308458957	-10.29272720977413
C	-3.16637461412518	-8.41238618598946	-11.31743786161839
C	-2.93014692752586	-7.47835802452271	-12.33506778950775
C	-3.64953758331257	-6.26887288716245	-12.39905989283681
C	-3.26353925665083	-5.20759603171590	-13.40739021753845
C	-4.63441381798823	-6.05458695998538	-11.42027290513340
C	-4.86775106569377	-6.98735438512961	-10.39525632859021
C	-9.15891259092765	-5.01207569581985	-5.91878493130453
C	-9.46752818218514	-4.01065362835007	-6.84199420385883
C	-10.74861944298718	-4.07050302690033	-7.49830256793542
C	-11.10339016561600	-3.19292538591627	-8.56520862726640

H	-10.38095152790794	-2.42907713025066	-8.88444761394332
C	-12.32711148443701	-3.30818030638971	-9.20897156413914
H	-12.57217957186118	-2.62838736195157	-10.03874111456389
C	-13.26018302904139	-4.30264349235509	-8.81377677075295
H	-14.22736284733198	-4.38336334464882	-9.33192077879406
C	-12.94498481830192	-5.17564084451076	-7.78407477798409
H	-13.65520029148483	-5.95891382477177	-7.47634660028361
C	-11.69109597463752	-5.09408161510711	-7.10963072604362
C	-11.35000060820988	-6.01194857405115	-6.07373879898205
H	-12.08905263673578	-6.76174230382278	-5.75269369356920
C	-10.09957700401328	-5.98861349726391	-5.47287772623586
C	-7.16025173065354	-3.22764898357125	-7.43683496159146
C	-8.48029375965427	-2.92182408528312	-7.07921402092075
C	-8.83267289521871	-1.54190189560971	-6.85088649245842
C	-10.10357054243591	-1.13914945709255	-6.33950164787811
H	-10.85232452169642	-1.90579809721618	-6.09899285069620
C	-10.40201300447955	0.20039263336991	-6.13560187619255
H	-11.38866398908633	0.48283934003457	-5.73812439920408
C	-9.44630569433382	1.20832496440520	-6.42984943075917
H	-9.69859802195208	2.26784902251972	-6.27334325179338
C	-8.19164481468885	0.85034241339892	-6.89644301828028
H	-7.43111782791682	1.61818567843161	-7.10705792196704
C	-7.84750934109797	-0.51873700112047	-7.10133582812272
C	-6.54010279802502	-0.89513780115115	-7.51569363428884
H	-5.79097368418951	-0.10950609438812	-7.69830578317518
C	-6.16467517460211	-2.22510168203940	-7.66236948299011
C	-2.93384937084572	-6.35054527595120	-5.76030404625684
C	-1.77191400251533	-6.87322732845165	-5.16852323995352
C	-0.63775746199177	-7.14814922530727	-5.93950727999789
C	-0.65493397357182	-7.00063402878147	-7.33778918324184
C	0.56710609298568	-7.41041623875650	-8.13713149480018
C	-1.83354957993547	-6.52563178241900	-7.92970688104588
C	-2.94681336475019	-6.16819443401533	-7.15141223106019
F	-1.94717759789348	-6.37528534432125	-9.25013423037193
F	0.73633234562630	-8.75032608858037	-8.10875676326476
F	0.49920336579676	-7.03290829335256	-9.42611701566340
F	-2.18242484158048	-4.50472576636149	-12.95444746564624
F	-2.91674779302553	-5.72905902768012	-14.60093973701784
F	-5.38784497621667	-4.94977314329413	-11.42388932152726
C	-9.72062041386829	-6.89076654246006	-4.34742474251529
C	-9.85047215222592	-6.43984229946768	-3.00289162680355
C	-9.18009442075929	-8.15922908952836	-4.60813668332305
C	-9.38645149797824	-7.28108146272147	-1.93384897422363
C	-10.40776396581954	-5.15556306561433	-2.66969289349480

C	-8.73154689183156	-8.98308217499944	-3.57109863143399
H	-9.08109412802093	-8.49149725823479	-5.64695932946984
C	-9.49038815933743	-6.84028736833884	-0.57551548383235
C	-8.80495831651646	-8.56212794903198	-2.22683617514797
C	-10.52385690187873	-4.74133732728996	-1.36339294594834
H	-10.75671074335006	-4.50729832146062	-3.48700691151957
H	-8.30362029008709	-9.96778717944435	-3.80949631859807
C	-10.08400391877997	-5.56783395044985	-0.27254512418714
C	-9.00625249223908	-7.67326599463201	0.49273538425165
C	-8.31242445374963	-9.36563748087438	-1.13913045245307
H	-10.96916251657408	-3.76108164511580	-1.13145372528614
C	-10.21124895645851	-5.17100435343723	1.07814339039612
C	-9.14651961067154	-7.22774486618506	1.82695370463939
C	-8.40495708014037	-8.93935974944211	0.16346121421869
H	-7.85920168303276	-10.34117375862296	-1.37433621311055
C	-9.75030030835291	-5.99472245392211	2.11346832099354
H	-10.68113202457110	-4.20181824464781	1.30739030502924
H	-8.78202358698331	-7.86980750299184	2.64389660182112
H	-8.02738860810191	-9.56938306203185	0.98376838473283
H	-9.86177707573209	-5.67169965633302	3.15965170932667
C	-9.06064212219495	-7.73836524309313	-9.89210993623436
C	-8.20865972317051	-8.07495026856827	-10.99012494615514
C	-9.61465021854655	-6.44519841304694	-9.82060527210191
C	-7.92985637254501	-7.07477163727387	-11.98672363809600
C	-7.61332520312440	-9.37554899544070	-11.15719297092481
C	-9.35288805542023	-5.47770406499930	-10.79151500390549
H	-10.24894740336371	-6.19089958925989	-8.96186264953220
C	-7.05477556919908	-7.37164877140153	-13.08212153035619
C	-8.51603001218904	-5.76630805234929	-11.88921434977561
C	-6.74364783834397	-9.64718128759385	-12.18476489824811
H	-7.84036550884228	-10.16967959976622	-10.43543974894796
H	-9.79664029133354	-4.47539072764974	-10.69677724375660
C	-6.42554410454762	-8.65939151004973	-13.17653583148420
C	-6.78061543945679	-6.37493430078788	-14.08066782478702
C	-8.23198054080158	-4.79231052881571	-12.90798727624591
H	-6.27144699837546	-10.63879119472105	-12.25337948926092
C	-5.52481406007822	-8.91357623362665	-14.23649982442294
C	-5.88705406821205	-6.68231642671230	-15.13081318270979
C	-7.40414063477601	-5.08396419638979	-13.96287560590712
H	-8.69278971354357	-3.79634528208051	-12.81740046802143
C	-5.26005844929400	-7.93367200434603	-15.20109834701425
H	-5.03311466290062	-9.89705625829308	-14.29237269276359
H	-5.66779819999461	-5.91132267121289	-15.88477467054069
H	-7.18454044341357	-4.32585096555058	-14.73002583904031

H	-4.54905766551534	-8.14295071987161	-16.01403685015100
C	-4.49393803308495	-10.91277079499332	-4.52028522441851
C	-3.10352182012088	-10.83756548207825	-4.81199503196933
C	-4.95889160831659	-10.50467440653774	-3.25483227359757
C	-2.19671928682181	-10.38474924202925	-3.78971490574675
C	-2.56883235155293	-11.17850443903320	-6.10278371591991
C	-4.08312572761878	-10.06778384306814	-2.25846176175201
H	-6.03718240443237	-10.55820464657206	-3.04579402454235
C	-0.79188092755245	-10.32322611165925	-4.05461138718692
C	-2.69367075320355	-10.00222434888268	-2.49754013040271
C	-1.22194036914383	-11.10184683611116	-6.35952426762317
H	-3.26530825021697	-11.45855150775845	-6.90544422991117
H	-4.47522465672042	-9.76950815223689	-1.27512084606535
C	-0.28661239463047	-10.69678951142952	-5.34654465529853
C	0.11927355698595	-9.88058843797365	-3.03394471613258
C	-1.75962227921879	-9.56920915869937	-1.49332435555205
H	-0.84718663237730	-11.31490051579317	-7.37112823901612
C	1.10558357551170	-10.63229542466392	-5.58219325596369
C	1.50145510651798	-9.82004222894778	-3.32165387981602
C	-0.41104131931096	-9.50948933416977	-1.74959276347762
H	-2.15172426394055	-9.27950882135602	-0.50540468075377
C	1.98439627015663	-10.19835067747861	-4.58171150285203
H	1.48872292465870	-10.90927030422858	-6.57545984033764
H	2.19214885797592	-9.45463687115603	-2.54644781664015
H	0.29172162791161	-9.16443807770342	-0.97565893125437
H	3.06329001214400	-10.14132143326396	-4.79129762480795
C	-4.76503909087908	-2.57692463435018	-8.04645065814855
C	-3.73455017668667	-2.62014861308765	-7.06646749627995
C	-4.46191621397063	-2.85442106881319	-9.39181082570127
C	-2.40895794474778	-3.01073374548088	-7.46430089323824
C	-3.97357413705327	-2.33756480533741	-5.67533394484777
C	-3.16020777800436	-3.17028051604473	-9.79273021094215
H	-5.26901216909425	-2.82942171437091	-10.13680382142785
C	-1.39495381441230	-3.21761896748681	-6.47392484981309
C	-2.11597005783843	-3.26408807841275	-8.84706690004181
C	-2.99425615095386	-2.51770443581377	-4.73168622466200
H	-4.97789360350147	-2.00963549095600	-5.37353643840949
H	-2.94208000835451	-3.37509415982897	-10.85062464023984
C	-1.69252991986286	-3.01382877298860	-5.08425645172542
C	-0.09493140856909	-3.69219650355415	-6.86169090106914
C	-0.78300527971646	-3.66296439917436	-9.21493502348122
H	-3.20461564980907	-2.32267894381174	-3.66971464592868
C	-0.71952472034635	-3.34569006142075	-4.11539679358172
C	0.84734453215422	-4.00764322692685	-5.85667333849905

C	0.18391239554810	-3.87510783506193	-8.26258271159476
H	-0.57150720546837	-3.84782706573003	-10.27909897235805
C	0.52984181682623	-3.85012459410775	-4.50023176651671
H	-0.96384688367576	-3.22488301990303	-3.04881511121917
H	1.82780149589658	-4.41054565311165	-6.15177092413447
H	1.18612143496352	-4.22281869801301	-8.55457233819308
H	1.26448736459466	-4.13562854513399	-3.73489344943685

Optimized product complex of **TS-3a-4**

C	1.46613314116832	2.28432779043571	-5.30986857955563
H	1.00539039181387	3.06343148166843	-4.66970695163518
C	0.35544416776350	1.35297636611343	-5.82559589721359
H	0.48309873855082	1.11525136098118	-6.89817597847431
O	2.18688829588422	3.02186787436769	-6.27197850825568
C	2.56835468657438	2.39029919666133	-7.49270430831650
H	3.49782489756008	2.91335993385129	-7.80848574834246
H	2.84677698020065	1.32379831009110	-7.35866964643500
C	1.52727177177094	2.53588962813349	-8.58964370768598
C	0.70381698646500	3.67638449039575	-8.63585965696405
C	1.38658831879309	1.54693675829815	-9.58439041898854
C	-0.23197445691975	3.83629011898781	-9.66558655256511
H	0.79860446932898	4.43653688200797	-7.84643774067786
C	0.44888613053062	1.69964523327849	-10.61219699995893
H	1.99906742308954	0.63598604799388	-9.54104673897198
C	-0.36049219812675	2.84834782229179	-10.65679867389837
H	-0.87393576386065	4.72789966038452	-9.70049969391102
H	0.32871881383539	0.90364009397396	-11.36146908927171
C	2.43038805018711	1.51111648049570	-4.29574418428982
C	1.52479322713626	1.00888961325378	-3.18454430125819
C	0.45242768647553	0.28363795774815	-3.54134696179361
C	3.17441120703091	0.38380580182843	-4.93655393114475
C	0.22700453775514	0.01749047699706	-5.01341662836460
C	2.62727327084678	-0.74566359966468	-5.48339418250299
C	1.21392271575412	-0.94691879378713	-5.59947518564891
H	3.15154370139116	2.25919837565621	-3.91957820209373
H	1.75470602805288	1.26796350034548	-2.13997531778536
H	-0.27693093019814	-0.11170858640498	-2.81918567541500
H	4.27222707053901	0.46757078069389	-4.99570449412274
H	-0.77004054845157	-0.41226267183240	-5.19344133222035
H	3.29008194860215	-1.50576484717829	-5.91307382336473
O	0.71642254014671	-1.89045131350004	-6.32182631601619
C	-1.36561663111188	2.99190893051835	-11.76952018904101
F	-0.77181087447749	3.09821478535026	-12.98089801374667
F	-2.16268619921076	4.07743064870469	-11.60918146189577

F	-2.19325209512541	1.90669096561121	-11.83456658729959
H	-0.62034826306845	1.86646943685967	-5.74443915866401
H	1.46277299244088	-2.39680913684151	-6.88870839489630
S	3.16876496812052	-7.04892768515665	-9.35013162189324
S	3.08392527444686	-1.97325833187439	-8.79685934470787
P	0.86878741256360	-5.29934489774982	-9.15784227274950
P	0.89239410797809	-2.65512675401626	-10.57356226226041
F	4.90618860720655	-9.05217889784726	-10.67510878512397
F	5.88104670273315	-9.04814705818761	-13.15262957979804
F	4.39019973758123	-6.52960423487957	-16.09369367904044
F	1.77604604543091	-5.67507260710263	-11.93591675147092
F	5.73720963832796	-1.85233129282815	-7.38776635110535
F	7.96399114504702	-3.21019171772463	-7.90936954388203
F	9.30860215978254	-4.50872837719329	-9.86890575838734
F	3.66584641419999	-3.91051321294090	-11.17934982859066
O	0.09331989199530	-6.64255777021336	-9.71445710203856
O	0.19563445891589	-5.06985800140231	-7.64893274605877
O	4.58271775922418	-6.86000934247373	-8.93531113867018
O	2.41024918312161	-8.19140473783223	-8.79500262721958
O	-0.34459111113328	-1.54553914774155	-10.51502866552761
O	1.06866091724869	-2.86901237103599	-12.19880605270852
O	3.50554523266113	-0.64068963950002	-8.30264987177176
O	2.49475341973589	-2.89942708788428	-7.73702689136978
N	0.38051080806177	-3.99167339548522	-9.92025605318826
N	2.43233303682016	-5.61515761883823	-9.18662467697170
N	2.16994857782522	-1.80790757814448	-10.06997946603175
C	-1.28411332325378	-6.70976627491074	-9.77457947627678
C	-2.00719819450002	-6.75306953738183	-8.58388718404154
C	-3.44569818434628	-6.63853953409503	-8.65807024558226
C	-4.26989686535604	-6.48750054863023	-7.50427491925567
H	-3.80169314533451	-6.48623246237296	-6.51068786885860
C	-5.64304804210276	-6.32362214267133	-7.62003842986979
H	-6.25378249936268	-6.19229445200007	-6.71425959566555
C	-6.26495233625080	-6.32374727745434	-8.89593622360145
H	-7.35617356542952	-6.20677728445395	-8.97538686831668
C	-5.49232860852793	-6.46765288648123	-10.03818601255089
H	-5.96106727767922	-6.47307802071885	-11.03452671425265
C	-4.07535087199605	-6.60925738278358	-9.95540587909332
C	-3.27747622967096	-6.74172491634168	-11.12554151602020
H	-3.77357808116991	-6.80699989127849	-12.10554498377580
C	-1.88893929646149	-6.80588987608676	-11.06718927517878
C	-0.31102016274119	-6.05912423350030	-6.82914770141681
C	-1.31386400967328	-6.93220148614931	-7.27877197249384
C	-1.71233994467505	-8.02826226807973	-6.42709350523397

C	-2.55232308367109	-9.08847638256070	-6.88204737000639
H	-2.90153243985207	-9.08670880417343	-7.92345810270733
C	-2.91178623734156	-10.12852466165556	-6.03688490207078
H	-3.54784612578015	-10.94196749851456	-6.41727073000964
C	-2.45913585505349	-10.15888622664884	-4.69165753006555
H	-2.75585385414609	-10.98682434997614	-4.03048810596295
C	-1.62897932585489	-9.15313531683369	-4.22284964210091
H	-1.25281651497521	-9.17125459156269	-3.18817886275598
C	-1.22865894317187	-8.08069152004995	-5.07280884791967
C	-0.37037765454514	-7.04667786658102	-4.61342219099180
H	-0.05533609568666	-7.04269167445208	-3.55879006948329
C	0.11229239057260	-6.05247607057665	-5.45823075922213
C	3.37591745179480	-7.28938075174815	-11.16768772686643
C	4.37790336944060	-8.20071819712488	-11.55404595967327
C	4.88561019474029	-8.20447582452272	-12.85901308311586
C	4.38249752122885	-7.33042539478891	-13.84338465254753
C	5.08051595954683	-7.24812045924272	-15.18381015233767
C	3.31349531500694	-6.49661555406650	-13.47919196142739
C	2.80194248450574	-6.49539515528170	-12.16923639750586
C	-1.51900239688359	-1.99504559195779	-11.11828581509380
C	-1.57938714045799	-2.03293821355059	-12.51343331941095
C	-2.69169299040888	-2.70167053170433	-13.13219002233499
C	-2.75820636436500	-2.93404864185055	-14.53856217437171
H	-1.94579394387361	-2.55985849539754	-15.17694387716386
C	-3.81500873733506	-3.64129914565719	-15.09392238544078
H	-3.83945263900690	-3.82340647177869	-16.17882838933005
C	-4.86237570548127	-4.13728632296342	-14.27237790906154
H	-5.69849039882315	-4.69037439696483	-14.72602657538736
C	-4.82514897754680	-3.92934311647150	-12.90203129266543
H	-5.62711396533602	-4.31286443014040	-12.25309050593059
C	-3.74241384050442	-3.22671196698606	-12.29535393565557
C	-3.67606821902432	-3.04284373366933	-10.88512358415086
H	-4.50504921036905	-3.40733550104992	-10.25945039681110
C	-2.58539824763956	-2.43057016450224	-10.27710944743937
C	0.83763128703821	-1.81748991489635	-13.07999001374995
C	-0.48091198845172	-1.40161464006835	-13.29273354983562
C	-0.73422317141220	-0.31006022364427	-14.19766131152507
C	-2.03677896161530	0.22824603062978	-14.41931651311342
H	-2.89023165409613	-0.18973912361324	-13.86979423358366
C	-2.23263414372899	1.28156867765090	-15.29905432953996
H	-3.24600074307675	1.68414656793246	-15.44664837171734
C	-1.13775273311350	1.85807380880215	-15.99416247480337
H	-1.30718601217265	2.69634817842645	-16.68639589003248
C	0.14360083064853	1.37555899052873	-15.78392348212441

H	1.00481904544963	1.82356891087611	-16.30328408057887
C	0.38011232703632	0.29258062951336	-14.88681817847762
C	1.69381568619428	-0.20096792067148	-14.65403200434961
H	2.53785275952829	0.25425510617097	-15.19452727686117
C	1.95029698519055	-1.23917956307411	-13.76534290511192
C	4.59662084992990	-2.84921151858979	-9.24987352679109
C	5.73084426936341	-2.68729820470587	-8.42896007781095
C	6.89182161375837	-3.42709703045835	-8.68079161796139
C	6.94330079596416	-4.39466059007732	-9.69979923184860
C	8.18125302244238	-5.26311314267075	-9.80989040041733
C	5.81511987580250	-4.54347622982994	-10.51874354955814
C	4.66748170574600	-3.75808171740646	-10.31517955690444
F	5.79060891812857	-5.40858336623710	-11.53283139734572
F	8.29199381449426	-6.05483535564889	-8.71447882127574
F	8.17328079105674	-6.05672739234531	-10.89174682116482
F	6.29520838437773	-6.64440125436486	-15.04199610707317
F	5.31035472796092	-8.46426723350502	-15.72254128287528
F	2.73597132209261	-5.66789682438005	-14.35492750231475
C	-2.57008090706624	-2.22122418030557	-8.80250769850194
C	-2.68933453465950	-0.91584503698304	-8.24361866093687
C	-2.54720605487299	-3.33903865688780	-7.95174673018874
C	-2.84168808262271	-0.77846113627517	-6.81990686919433
C	-2.70141850137170	0.27502263448944	-9.05055366619292
C	-2.66462151771330	-3.20945619475448	-6.56623595845141
H	-2.42417915113089	-4.33116921129985	-8.39858928458533
C	-2.99992075255677	0.52075984241941	-6.23756152383380
C	-2.83594417979268	-1.94130290112726	-5.97416097897538
C	-2.84011121414797	1.52109922736648	-8.49024136762340
H	-2.59011632318514	0.18413130326708	-10.13886105016886
H	-2.63014092387863	-4.10676725316277	-5.92974871144489
C	-2.98516891087590	1.69142268186964	-7.07140498466159
C	-3.14704688433145	0.66413858115738	-4.81314386870321
C	-2.99952452624713	-1.76955677899153	-4.55481776127828
H	-2.83809504767819	2.41561297332537	-9.12922597633675
C	-3.09546201206739	2.96684100871464	-6.47098925063910
C	-3.25226528846434	1.96057856635718	-4.25976805712591
C	-3.15359214642398	-0.52244572914862	-3.99684610008326
H	-3.00133108738899	-2.66874365402491	-3.91914929008417
C	-3.22239183460783	3.09685959887227	-5.08134966136097
H	-3.07313086593431	3.85969983024015	-7.11450947912938
H	-3.35752096811913	2.06759630254514	-3.16900536725441
H	-3.28057273921353	-0.40869253525733	-2.90874951642478
H	-3.30318545862039	4.09781695223239	-4.63107809444444
C	-1.08700995628416	-7.02485169090475	-12.29641378927980

C	-0.16003168810295	-8.10754145742360	-12.39900630101330
C	-1.30011726037640	-6.18407860742859	-13.40679107697741
C	0.53972101034593	-8.30949612631486	-13.63993711893223
C	0.11012900921269	-9.01842403665122	-11.31744471858726
C	-0.63007007143870	-6.38665222762695	-14.61473394824028
H	-1.99475214445883	-5.33889069753299	-13.30371244494305
C	1.50732205710453	-9.36029800513389	-13.75823565469111
C	0.28913339361560	-7.44735804279017	-14.76267234277355
C	1.06736909195531	-9.99725898310971	-11.41712331884925
H	-0.44136038714545	-8.91504333565193	-10.37527737561535
H	-0.81076823650248	-5.70669836845777	-15.46150742955851
C	1.80819764343408	-10.19594863858867	-12.62971858218830
C	2.20976115620916	-9.56287869554759	-14.99498027198229
C	0.99841348375911	-7.68140771459242	-15.99159243757467
H	1.28632380630657	-10.63882907719642	-10.55082593162439
C	2.81423836259735	-11.18223681685742	-12.74656554804767
C	3.18979929742543	-10.57784746026467	-15.07168769661157
C	1.91392126980229	-8.69815386998190	-16.10678170442239
H	0.80113709400522	-7.01069629065103	-16.84198160344033
C	3.49634752035107	-11.36745391270324	-13.95548482719675
H	3.05624823658018	-11.80082892442231	-11.86894885560868
H	3.73250815180877	-10.72194320688912	-16.01756230986489
H	2.46046597049989	-8.85292162729585	-17.04951452456616
H	4.27983100024931	-12.13640735301301	-14.02946807673090
C	0.92898836951272	-4.96358717318739	-4.84347462515562
C	2.33291165487279	-4.84265358883140	-5.04625208064763
C	0.28793028338943	-4.11934718429313	-3.91298652428720
C	3.06524550724205	-3.86022343595241	-4.29093885130266
C	3.05319280957434	-5.69949733925949	-5.94496381076832
C	0.99586407561382	-3.17037156968295	-3.16925832688264
H	-0.79700906262902	-4.22789446061435	-3.76696084961969
C	4.47807905626711	-3.73051504458323	-4.47217685068954
C	2.39335812150673	-3.02845258955024	-3.32910976760630
C	4.40887007560888	-5.57089806781667	-6.12225385646120
H	2.50429397236724	-6.47086375128344	-6.50253992203878
H	0.47303543463044	-2.54326336534408	-2.43168538032271
C	5.16207725404400	-4.58645756717194	-5.39936659425192
C	5.21980401222158	-2.75529704890920	-3.72038937805213
C	3.16428251351491	-2.09103679702219	-2.55964915854704
H	4.91835335977244	-6.21331440947767	-6.85502412647622
C	6.55883340460707	-4.44544151329067	-5.56239953333024
C	6.61131477522713	-2.63757386100227	-3.93522224895861
C	4.51722525779212	-1.94806419906688	-2.75775831520396
H	2.64073177864123	-1.47331724713739	-1.81497320923147

C	7.26943859239816	-3.47264659377895	-4.84859265579469
H	7.07913615381598	-5.10803463245097	-6.26990780352484
H	7.17754057741879	-1.88493095261565	-3.36452953087759
H	5.09282828009634	-1.21888358650507	-2.16580676228308
H	8.35267183019103	-3.36597752696985	-5.00669768591009
C	3.33729476131850	-1.76864430786589	-13.60542460880479
C	4.27000710461054	-1.17431328065261	-12.71252953257628
C	3.73055074084360	-2.86298897205130	-14.39669676865124
C	5.59151303161283	-1.73247222598400	-12.60925410950554
C	3.93506931185027	-0.04923752473832	-11.88065676987805
C	5.02228195545614	-3.39099403039178	-14.32168754505320
H	2.99922798352439	-3.31238646485439	-15.08215931357502
C	6.52013553522839	-1.20434405020858	-11.65578257923571
C	5.97222916596873	-2.84665498705955	-13.43168010779411
C	4.82239526899792	0.44910122066983	-10.95971935990701
H	2.93429799158559	0.39341195028710	-11.97539662722450
H	5.30535924016520	-4.24315628288367	-14.95518557642284
C	6.12977706323044	-0.12448579644924	-10.79248006051654
C	7.82900090682170	-1.78380081927596	-11.52631017474757
C	7.29838012028167	-3.38943107836498	-13.30217210858653
H	4.53081514630634	1.28860537093615	-10.31149050267546
C	7.03139455232425	0.32165726000282	-9.80029111836185
C	8.70028959232325	-1.29533106278822	-10.52665201833127
C	8.19054956205973	-2.87629542830955	-12.39261153278198
H	7.56932348220019	-4.25234663741458	-13.92906676445276
C	8.29800089705833	-0.26311665101900	-9.66796177728353
H	6.71849235552804	1.13154414931759	-9.12362142764167
H	9.69179012726974	-1.75787682565176	-10.41235014439842
H	9.19339605107527	-3.31601699738921	-12.28779531287540
H	8.98275482773281	0.08846934855586	-8.88153541797465

Optimized product complex of **TS-3a-4-SRR**

C	-5.75179988820933	-0.34337252170736	4.22406642296319
H	-5.98371006724618	-1.34866802742285	4.64395057075174
C	-6.03175071714354	-0.29156630410921	2.72391850467467
H	-5.08884864364204	-0.53045945348129	2.19646245328080
O	-4.36192922405202	-0.08087005602743	4.30720835059384
C	-3.78880472438863	0.05103929513461	5.60319579622024
H	-4.22457845475341	0.92005043169830	6.14255715422766
H	-2.72720428123481	0.30881499337391	5.41890229088822
C	-3.93182236701324	-1.20007178570865	6.43597423669767
C	-3.28882458916288	-2.39185365469014	6.04757403035504
C	-4.74835897979819	-1.21013736313801	7.58065842380314
C	-3.46032996437955	-3.56698190208242	6.78672557220867

H	-2.63846446709273	-2.39061953719155	5.15947735704122
C	-4.93090138604394	-2.38250613569595	8.32352304553985
H	-5.24141899946972	-0.27948691985044	7.89644525352658
C	-4.28699405361482	-3.56498497364559	7.92431308457837
H	-2.93729676316569	-4.48887469665142	6.49671152401313
H	-5.56126520716609	-2.37686840920389	9.22495480384635
C	-6.66540955008878	0.69165886481718	5.03983736602843
C	-7.96812613665428	0.97093718326271	4.29727894774223
C	-7.88827537493313	1.26623861267252	2.98636766537205
C	-6.01427887975113	2.03602472858732	5.16327497017998
C	-6.53688942615869	1.14088982735440	2.32287524154959
C	-5.45716098753268	2.68342843857146	4.09753752289905
C	-5.47216796885376	2.11194212221662	2.78120673883928
H	-6.87624839268074	0.28799117765072	6.04727389202805
H	-8.90814569332406	1.00499774420909	4.86744140670965
H	-8.77284170068000	1.50824861612493	2.37899853869141
H	-5.94051019016292	2.50869395412378	6.15432670865709
H	-6.58809408343876	1.21567247528910	1.22068830387612
H	-4.90728105701238	3.62532658077404	4.24985519904986
O	-4.51976802369077	2.33841847010619	1.94139089713033
C	-4.48841800189728	-4.82982758568640	8.72031005559234
F	-5.62707244510985	-5.47595128855317	8.36565148811251
F	-3.46650151058613	-5.70484134777549	8.55599376346036
F	-4.58659333160614	-4.57073852670247	10.05283381433185
H	-6.81115413044747	-1.01440130890309	2.42070656083284
H	-3.65960616190550	2.66565739895329	2.45883677419251
S	-0.51517237349778	8.25961811725383	6.44441783795886
S	-2.13507897461224	3.63878724983893	4.40484416367135
P	-2.41021747892142	6.27597481025482	7.36810497885351
P	-2.01409632980140	3.27492380957095	7.24538233077829
F	0.31848190037225	5.83089418114428	8.39967678026689
F	2.74068001400929	5.17484989274582	9.09353607035480
F	5.83880266688601	7.79049784131028	8.98192934747673
F	1.95024348472091	9.90356909433207	6.49717519475657
F	-1.41876560269706	4.35561322578200	1.55323332493319
F	0.37478297126623	6.14234512906040	0.73623727251649
F	2.96863451368670	7.05138534588311	1.39557927473199
F	0.38029283184046	4.62536365007415	5.97052725728357
O	-2.39653670988310	7.12390671313917	8.78908406538089
O	-3.96372127466864	6.55359340904121	6.85549874013211
O	-0.02646796102489	8.54545678722932	5.07330896005465
O	-1.22476504211782	9.32421741684717	7.18626656106963
O	-3.18752563197225	2.20117279410898	7.76205539449484
O	-0.71698272005517	2.78027531593273	8.13280446213042

O	-2.46733561474287	2.63408493395498	3.32753685807653
O	-3.12934145439497	4.73083918073768	4.59274119468322
N	-2.40018367773508	4.72185123968363	7.70911224602077
N	-1.29668716701182	6.84361062707027	6.38645511838917
N	-1.77069199673331	2.81783913421079	5.71468433484285
C	-3.41031176502501	6.86527863817274	9.69877346050264
C	-4.69535918564272	7.34943770978308	9.43337193907699
C	-5.78238020332364	6.89622063769957	10.25994753486213
C	-7.14602651537842	7.19124001599973	9.96527309693419
H	-7.37703781089193	7.83107870224642	9.10230628311488
C	-8.17339375806115	6.66206288440036	10.73282700224494
H	-9.21891858087968	6.88852831463735	10.47517683285943
C	-7.88687683651274	5.82252014038718	11.84176888967695
H	-8.71020264528293	5.41009533833017	12.44414050754095
C	-6.57146439489792	5.51942754480115	12.15719983569253
H	-6.33591049597943	4.86604758565251	13.01210651772331
C	-5.49203284476993	6.03134088404690	11.37740330826319
C	-4.14243636658270	5.69113690019331	11.66992641060494
H	-3.93030082087747	5.08128667414947	12.56147746550277
C	-3.08374512150884	6.09273055211807	10.85821476998489
C	-4.49487015551214	7.82853982151557	6.99799576633566
C	-4.89728158327930	8.25140440223212	8.26690543039751
C	-5.42680363777116	9.58196208722176	8.41222694802402
C	-5.77304522101245	10.13992236290462	9.67880400213282
H	-5.62725004424368	9.53698291852740	10.58548068831998
C	-6.27853569385554	11.42810940927420	9.77701588902952
H	-6.53570712242850	11.83767321117690	10.76548954446130
C	-6.45889123012446	12.22422238174368	8.61533572129474
H	-6.86271834532139	13.24379287618724	8.70609897785685
C	-6.10975056622986	11.72053793670790	7.37221579155809
H	-6.22618519167750	12.33532028356319	6.46619927050279
C	-5.57996034929785	10.40368746408480	7.23578207544773
C	-5.18591173607285	9.89449020395833	5.96602268798492
H	-5.30742893161590	10.52801605533300	5.07421734049522
C	-4.63833982775050	8.62617909489625	5.82473916189501
C	1.03810746531795	7.87115846557460	7.35242829417815
C	1.28270356297922	6.69458521284809	8.07444710476535
C	2.58695230026149	6.34501105150683	8.46596351319432
C	3.68143948082815	7.17962927658164	8.18877414453550
C	5.11697358487312	6.78032269173497	8.45451705636760
C	3.41827121926103	8.39505370181749	7.52607480202315
C	2.11994567183368	8.74433531205980	7.13200803866201
C	-3.34277381600606	1.89830132439496	9.10453947926789
C	-2.34527006722319	1.16899003861191	9.76249613178838

C	-2.46875779511502	0.96595056189137	11.18322430357476
C	-1.41906989155119	0.40541113837505	11.97046585497363
H	-0.48824941386101	0.09892218809111	11.47350442987637
C	-1.55453097703129	0.26349027973703	13.34399347192371
H	-0.72580180372282	-0.15840835789498	13.93207286678004
C	-2.74845097532876	0.66693005262724	13.99864294648587
H	-2.84298848700756	0.54837787123643	15.08833075667684
C	-3.78559326045154	1.21974962699891	13.26437033212084
H	-4.71197265040795	1.54825223380152	13.76081592046383
C	-3.67419106280480	1.39045639171190	11.85260543273663
C	-4.72177259146677	1.97950769385058	11.09167997066121
H	-5.65915235382469	2.26249015241292	11.59483240415750
C	-4.58119608851650	2.24754356898817	9.73380301402062
C	-0.42930955872304	1.41799532501921	8.17117678277689
C	-1.21953668993114	0.59285116607637	8.97809779404819
C	-0.96758938275972	-0.82824464115488	8.97598039861547
C	-1.77271997155035	-1.76438680676175	9.69044394773334
H	-2.63959166686364	-1.40467185889715	10.25848901953532
C	-1.48823905605998	-3.12133119683357	9.66719149221967
H	-2.12741920606571	-3.81666419921018	10.22898079573340
C	-0.39472705817402	-3.61869361949794	8.91193624098126
H	-0.18687643576959	-4.69904934711354	8.89756411487498
C	0.39113659044474	-2.74203711796689	8.18131846697008
H	1.23417423755036	-3.11259601656278	7.57766279083473
C	0.12790676447042	-1.33964828914897	8.18900576120126
C	0.92443226281861	-0.43718979602089	7.43231490106820
H	1.77067422562684	-0.83208271810525	6.84942180859134
C	0.67009195104700	0.92956645391567	7.40000404008207
C	-0.64828899750283	4.48709725318192	3.81811354622638
C	-0.58063836139981	4.85908517363848	2.46160098224577
C	0.36853142777637	5.79254229571018	2.03040494943409
C	1.28140421275940	6.37989956756147	2.92357538241153
C	2.18317978047180	7.48780182132653	2.41589073293273
C	1.26873781941028	5.93668156039864	4.25265541898467
C	0.31757580135535	5.00179556807158	4.69410459838930
F	2.14905210472621	6.38077098522313	5.14963025572115
F	1.43549215960725	8.50705524126739	1.93117763937759
F	2.99491699549670	7.98302677984481	3.36150733267523
F	5.22388478642186	5.72562008951996	9.28905437175567
F	5.72723076122506	6.42287320073200	7.28788043044536
F	4.41719175512762	9.23899173692933	7.24417133176446
C	-5.70217855161987	2.83256852569026	8.94830593542824
C	-6.91366199691387	2.10608555118466	8.73860084489107
C	-5.55899708622111	4.11476489754032	8.39036006596194

C	-7.95970836568030	2.70512082257336	7.94993498684058
C	-7.12404613057713	0.77031586929540	9.23701592221419
C	-6.58875070567068	4.71498499198251	7.66493748726899
H	-4.61208292503282	4.64779890531151	8.52831420555347
C	-9.14886585727938	1.96648227551065	7.64753020757626
C	-7.80199111397179	4.03569252345323	7.43156325962705
C	-8.26095835479863	0.05812122190863	8.93718907376060
H	-6.34402718261075	0.31599422422464	9.86412317924830
H	-6.43891817391298	5.72461249032594	7.26019175690830
C	-9.30585149723720	0.62030917595551	8.12523581084779
C	-10.18439914316979	2.56075379189142	6.84418432306735
C	-8.86996189577290	4.61880217647170	6.66556156611286
H	-8.38812253295306	-0.96696748081963	9.31898672212790
C	-10.46968141657382	-0.10384939682669	7.77998499911597
C	-11.33104960004942	1.79701078095374	6.52826284374755
C	-10.01237090595737	3.91219426733156	6.38022165982188
H	-8.74635766762182	5.65239313922402	6.30771543319234
C	-11.46753807153391	0.47969094509296	6.98813152120687
H	-10.58355629468706	-1.13634735380479	8.14473751746113
H	-12.12202189465099	2.25422037516408	5.91355929152519
H	-10.81852557067014	4.36899832573060	5.78549218588792
H	-12.36722351456554	-0.09858701063337	6.72909966628868
C	-1.68773565796428	5.69507906532996	11.16797583125180
C	-0.61063770694204	6.63258174859415	11.20587559653326
C	-1.43067452829932	4.33969889350219	11.45782276881847
C	0.71306649050618	6.16250063898631	11.51950123341137
C	-0.78222521915185	8.03697520087307	10.93942433935773
C	-0.15170405520534	3.88428716819362	11.77863914888855
H	-2.26121381868058	3.62342762244531	11.40556980616156
C	1.82038465719912	7.07194454805718	11.51693000920264
C	0.94127718856349	4.77557132311593	11.81836831540028
C	0.28444573039151	8.90054337084455	10.89982950082024
H	-1.78854855826359	8.42675673310802	10.74131275223242
H	0.00853936605719	2.81679864171208	11.99317236675852
C	1.62107387639776	8.45209746811912	11.17270413882592
C	3.14367983139163	6.60374322525838	11.82754964726786
C	2.27263281056260	4.33867363342649	12.14217132552313
H	0.12234808219699	9.96088166737729	10.65343826817807
C	2.73576915849653	9.31959880898334	11.11813138162079
C	4.22410025162031	7.51238666567456	11.77417704874604
C	3.32946679298741	5.21478055533243	12.15114114449696
H	2.42724470206544	3.27408356175910	12.37791284282183
C	4.02195336650740	8.85188698169885	11.41539333462798
H	2.57670434419342	10.37314968220412	10.84100757012419

H	5.23796694399550	7.14665727006504	11.99632914703245
H	4.34590283272466	4.86469781877597	12.38811236742901
H	4.88116961451226	9.53630069102083	11.35647004200060
C	-4.19976542685070	8.10452471737425	4.49911688154255
C	-4.99009632214858	7.15754656212385	3.79212666208073
C	-2.98711790859723	8.55494812286898	3.94810820907223
C	-4.54954762965464	6.69932927490954	2.50597045059239
C	-6.22692167184663	6.64354711156451	4.31394527618864
C	-2.53957618936527	8.09042359863937	2.70714899563359
H	-2.36701040803478	9.26065065241054	4.51696261674777
C	-5.34223352576355	5.76521463132643	1.76806925826190
C	-3.31414110667177	7.18049999674489	1.95447618593902
C	-6.98240903283928	5.73277075979475	3.61468870330387
H	-6.56714088565894	7.00393095119211	5.29587727387105
H	-1.56657860026458	8.43101395332096	2.32074034188798
C	-6.57387842841367	5.27001617403269	2.31802690681531
C	-4.91540706793615	5.32376859372764	0.46771443858932
C	-2.91596062083725	6.72665528874568	0.64988195180517
H	-7.92155750008378	5.34366832056113	4.03694511788237
C	-7.35064327622887	4.35895162801883	1.56320158009027
C	-5.72728010656287	4.41163175903937	-0.24878798773240
C	-3.68390622709962	5.83869615798876	-0.06457925204291
H	-1.96511031126477	7.09478673989713	0.23685545792158
C	-6.93263125258097	3.94563092962409	0.28928967226364
H	-8.30333166051130	4.00225722210766	1.98372302881803
H	-5.39921724849900	4.07870733518540	-1.24542823251533
H	-3.35842070980866	5.49754127005028	-1.05935264692995
H	-7.55798231928072	3.25134302492389	-0.29344251296963
C	1.56066658575182	1.83630757158875	6.61976230042834
C	1.51748446924652	1.86358368543147	5.19744500225804
C	2.48884056883185	2.64155715242286	7.30546258064150
C	2.43001733298848	2.71797289868640	4.48583904555707
C	0.54605623798835	1.12037579419530	4.43820666513303
C	3.40247488838165	3.44342734595624	6.61568557272900
H	2.49240128105666	2.63487317115429	8.40451358769453
C	2.34291689115300	2.83378407296907	3.06090349899272
C	3.39551596341599	3.50068618765453	5.20574730723352
C	0.45357277141904	1.24331103775877	3.07352759753555
H	-0.15885054850540	0.47544780095729	4.98045539113658
H	4.13348069686280	4.04585390166182	7.17290930785156
C	1.33316201194996	2.11058292371875	2.33886252513589
C	3.22650717807883	3.71974935525805	2.35400836503897
C	4.28778597303231	4.35733201177726	4.47061879833087
H	-0.33073420469866	0.70222146556827	2.52401511712741

C	1.21575792041485	2.29877370762618	0.94325057701166
C	3.07713107950182	3.86692829380248	0.95649775076717
C	4.20728346845314	4.46177741577450	3.10347106341107
H	5.01228900609401	4.96070706019519	5.03848918568433
C	2.07705146268875	3.17079536739478	0.26424737724233
H	0.42489108599996	1.76062183507645	0.39886352129301
H	3.74136465365943	4.56123600304730	0.42111994130663
H	4.87497985050285	5.14506000569584	2.55734503127507
H	1.96562548330751	3.31411688265040	-0.82106326009819

References

1 Monaco, M.R., Poladura, B., Diaz de Los Bernardos, M., Leutzsch, M., Goddard, R. and List, B. Activation of Carboxylic Acids in Asymmetric Organocatalysis. *Angew. Chem. Int. Ed.* 53, 7063-7067 (2014).

2 Domzalska-Pieczykolan, A., Funes-Ardoiz, B. Furman, I., Bolm, C. Selective Approaches to α - and β - Arylated Vinyl Ethers. *Angew. Chem. Int. Ed.* **61**, e202109801 (2021).

3 Kleemiss, F., Dolomanov, O.V., Bodensteiner, M., Peyerimhoff, N., Midgley, M.,Bourhis, L.J., Genoni, A., Malaspina, L.A., Jayatilaka, D., Spencer, J.L., White, F., Grundkoetter-Stock, B, Steinhauer, S., Lentz, D., Puschmann, H., Grabowsky, S. Accurate crystal structures and chemical properties from NoSpherA2. *Chem. Sci.*, **12**, 1675-1692 (2021).

4 Neese, F. Software update: The ORCA program system—Version 5.0. *WIREs Comp. Mol. Sci.* **12**, e1606, doi:https://doi.org/10.1002/wcms.1606 (2022).

5 Perdew, J. P., Burke, K. & Ernzerhof, M. Generalized gradient approximation made simple. *Phys. Rev. Lett.* **77**, 3865 (1996).

6 Grimme, S., Antony, J., Ehrlich, S. & Krieg, H. A consistent and accurate ab initio parametrization of density functional dispersion correction (DFT-D) for the 94 elements H-Pu. *J. Chem. Phys.* **132**, 154104, doi:10.1063/1.3382344 (2010).

7 Weigend, F. & Ahlrichs, R. Balanced basis sets of split valence, triple zeta valence and quadruple zeta valence quality for H to Rn: Design and assessment of accuracy. *Phys. Chem. Chem. Phys.* **7**, 3297-3305 (2005).

8 Vahtras, O., Almlöf, J. & Feyereisen, M. Integral approximations for LCAO-SCF calculations. *Chem. Phys. Lett.* **213**, 514-518 (1993).

9 Weigend, F. Accurate Coulomb-fitting basis sets for H to Rn. *Phys. Chem. Chem. Phys.* **8**, 1057-1065 (2006).

10 Bannwarth, C., Ehlert, S. & Grimme, S. GFN2-xTB—An Accurate and Broadly Parametrized Self-Consistent Tight-Binding Quantum Chemical Method with Multipole Electrostatics and Density-Dependent Dispersion Contributions. *J. Chem. Theory Comput.* **15**, 1652-1671, doi:10.1021/acs.jctc.8b01176 (2019).

11 Pracht, P., Bohle, F. & Grimme, S. Automated exploration of the low-energy chemical space with fast quantum chemical methods. *Phys. Chem. Chem. Phys.* **22**, 7169-7192, doi:10.1039/C9CP06869D (2020).

12 Spicher, S. & Grimme, S. Robust Atomistic Modeling of Materials, Organometallic, and Biochemical Systems. **59**, 15665-15673, doi:https://doi.org/10.1002/anie.202004239 (2020).

13 Qiu, Z. *et al.* 3-center-3-electron σ-Adduct Enables Silyl Radical Transfer Below the Minimum Barrier for Silyl Radical Formation. *ChemRxiv*, doi:10.26434/chemrxiv-22024-dpqvv-v26432 (2024).

14 Yepes, D., Neese, F., List, B. & Bistoni, G. Unveiling the Delicate Balance of Steric and Dispersion Interactions in Organocatalysis Using High-Level Computational Methods. *J. Am. Chem. Soc.* **142**, 3613-3625, doi:10.1021/jacs.9b13725 (2020).

15 Harden, I., Neese, F. & Bistoni, G. An induced-fit model for asymmetric organocatalytic reactions: a case study of the activation of olefins via chiral Brønsted acid catalysts. *Chem. Sci.* **13**, 8848-8859, doi:10.1039/D2SC02274E (2022).

16 Ghosh, S. *et al.* Strong and Confined Acids Control Five Stereogenic Centers in Catalytic Asymmetric Diels–Alder Reactions of Cyclohexadienones with Cyclopentadiene. *Angew. Chem. Int. Ed.* **59**, 12347-12351, doi:https://doi.org/10.1002/anie.202000307 (2020).

17 Mardirossian, N. & Head-Gordon, M. ωB97M-V: A combinatorially optimized, range-separated hybrid, meta-GGA density functional with VV10 nonlocal correlation. *J. Chem. Phys.* **144**, doi:10.1063/1.4952647 (2016).

18 Neese, F., Wennmohs, F., Hansen, A. & Becker, U. Efficient, approximate and parallel Hartree–Fock and hybrid DFT calculations. A 'chain-of-spheres' algorithm for the Hartree–Fock exchange. *Chem. Phys.* **356**, 98-109, doi:https://doi.org/10.1016/j.chemphys.2008.10.036 (2009).

19 Izsák, R. & Neese, F. An overlap fitted chain of spheres exchange method. *J. Chem. Phys.* **135**, 144105, doi:10.1063/1.3646921 (2011).

20 Izsák, R., Neese, F. & Klopper, W. Robust fitting techniques in the chain of spheres approximation to the Fock exchange: The role of the complementary space. *J. Chem. Phys.* **139**, 094111, doi:10.1063/1.4819264 (2013).

21 Helmich-Paris, B., de Souza, B., Neese, F. & Izsák, R. An improved chain of spheres for exchange algorithm. *J. Chem. Phys.* **155**, 104109, doi:10.1063/5.0058766 (2021).

22 Marenich, A. V., Cramer, C. J. & Truhlar, D. G. Universal solvation model based on solute electron density and on a continuum model of the solvent defined by the bulk dielectric constant and atomic surface tensions. *J. Phys. Chem. B* **113**, 6378-6396 (2009).

23 Pracht, P. & Grimme, S. Calculation of absolute molecular entropies and heat capacities made simple. *Chem. Sci.* **12**, 6551-6568, doi:10.1039/D1SC00621E (2021).

24 Altun, A., Neese, F. & Bistoni, G. HFLD: A Nonempirical London Dispersion-Corrected Hartree–Fock Method for the Quantification and Analysis of Noncovalent Interaction Energies of Large Molecular Systems. *Journal of Chemical Theory and Computation* **15**, 5894-5907, doi:10.1021/acs.jctc.9b00425 (2019).

25 Hellweg, A., Hättig, C., Höfener, S. & Klopper, W. Optimized accurate auxiliary basis sets for RI-MP2 and RI-CC2 calculations for the atoms Rb to Rn. *Theor. Chem. Acc.* **117**, 587-597 (2007).

26 Chai, J.-D. & Head-Gordon, M. Systematic optimization of long-range corrected hybrid density functionals. *J. Chem. Phys.* **128**, doi:10.1063/1.2834918 (2008).

27 Zheng, J., Xu, X. & Truhlar, D. G. Minimally augmented Karlsruhe basis sets. *Theor. Chem. Acc.* **128**, 295-305 (2011).

28 Stoychev, G. L., Auer, A. A. & Neese, F. Automatic Generation of Auxiliary Basis Sets. *J. Chem. Theory Comput.* **13**, 554-562, doi:10.1021/acs.jctc.6b01041 (2017).
